# Borylated 5-Membered Ring Iminosugars: Detailed Nuclear Magnetic Resonance Spectroscopic Characterisation, and Method for Analysis of Anomeric and Boron Equilibria

**DOI:** 10.3390/molecules30071402

**Published:** 2025-03-21

**Authors:** Michela Simone

**Affiliations:** 1 Discipline of Chemistry, University of Newcastle, Callaghan, NSW 2308, Australia; michela_simone@yahoo.co.uk; 2 Newcastle CSIRO Energy Centre, 10 Murray Dwyer Circuit, Mayfield West, NSW 2304, Australia

**Keywords:** monosaccharide, iminosugar, boronic acid, boronate, boron, BNCT, glycosidase, cancer, Fsp^3^ index, NMR, mutarotation, borarotation, induced fit

## Abstract

This paper describes the first detailed NMR analysis of the borylated intermediates and target compounds for a small library of pyrrolidine iminosugars of l-gulose absolute stereochemical configuration. The iminosugars were functionalised via *N*-alkylation to bear a boronate ester or boronic acid groups. The addition of the organic boron pharmacophore allows to further explore the chemical space around and in the active sites, where the boron atom has the capability to make reversible covalent bonds with enzyme nucleophiles and other nucleophiles. We discuss the concurrent complex equilibrium processes of mutarotation and borarotation as studied by NMR.

## 1. Introduction

The development of functionalisation of drug leads (of high and low Fsp^3^) with organic boron moieties has been previously studied in our research group [1,2,3,4]. Organic boron pharmacophores present a brilliant opportunity to manage cancer [5,6,7,8,9] and modulate carbohydrate-active enzymes via additional drug–enzyme interaction strategies. In fact, organic boron atoms expand the traditional medicinal chemistry space to reversible intermolecular interactions between the drugs and the target enzymes, introduce adaptations related to conformational preferences for the B atoms ranging from trigonal planar to tetrahedral, and modified electronics at and around the B atoms.

One important aspect must be considered for these borylated drugs. When examining the induced fit model for enzymes and their interactions with specific drugs, it is acknowledged that enzymes possess conformational plasticity, meaning that during the ligand-free and ligand-bound states of an enzyme, the active site conformations are different [10]. Organic B atoms introduce a further refinement to the induced fit model by providing drug molecules that are also conformationally flexible and can adapt to the enzyme active sites and neighbouring sites. Thus, by working in unison, drugs and enzymes can team up to adapt to each other. Future investigations around the kinetics of these systems will likely present additional features.

Additionally, organic boron in its acidic form displays low intrinsic toxicity; hence, drug leads containing B pharmacophores are highly desirable in medicinal chemistry.

Therapeutic applications encompass modulation of carbohydrate-active enzymes [1,2,3,4]—traditionally achieved via carbohydrate analogues [11,12,13,14,15,16,17,18,19]—and radiotherapeutic agents for boron neutron capture therapy (BNCT) that are more selective for cancer over healthy cells [20,21], and whose activation is completely within clinical control [3].

By virtue of its Lewis acidity, organic B atoms can be found in equilibrium between several forms. When considering boronic acids (R-B(OH)_2_) and their corresponding boronate species (R-B(OR′)(OH), R-B(OR′)_2_, R-B^−^(OH)_3_, and R-B^−^(OR’)_3_), it is of paramount importance to develop robust methodologies for their installation and purification and to study the resulting B atom equilibria [1,2,3,4,22].

An organic B atom (e.g., boronic acid or boronate ester) in a molecule is expected to engage with nucleophilic atoms (e.g., oxygen and nitrogen), both intramolecularly and intermolecularly, to potentially give a plethora of boron adducts of trigonal planar and tetrahedral geometries. The final equilibrium mixture depends on many factors, including pH, solvent, temperature, concentration, arrangement of atoms in space, and intramolecular proximity of nucleophilic atoms. This equilibrium process has been designated borarotation [3,4]. In its fundamental mechanism of action, borarotation is comparable to mutarotation [23] but it can present additional complexities due to interactions between solvent molecules and B atoms. To comprehensively elucidate the solution (and eventual physiological) behaviour, borarotation equilibria exploration requires a separate study. In the future, machine learning and molecular modelling [24,25,26,27] could allow refinement of our understanding and modulation of these equilibria and of enzyme–drug interactions.

In this article, mutarotation and borarotation equilibria in aqueous/protic solutions for two small libraries of iminosugar drug leads are studied utilising NMR analysis. A detailed discussion of the divulged equilibria is of crucial importance when studying borylated iminosugars and—by extension—borylated organic molecules. This paper provides an NMR methodology to study complex equilibria and advances the comprehensive utilisation of borylated carbohydrate derivatives in clinical applications (e.g., for BNCT and glycosidase modulation).

## 2. Results and Discussion

At its core, analysis of ^11^B-NMR spectra provides the B atom environments of a given chemical species and insights into its borarotation profile. When coupled with analysis of the ^1^H-, ^13^C-, and 2D NMR spectra, it is possible to discern the equilibria of a molecule involved in borarotation of the B atom and mutarotation of lactol intermediates. Delineation of such equilibria tends to be complex, especially when borarotation and mutarotation [23,28] occur concomitantly.

NMR analysis of the borylated intermediates, target compounds, and side-products (structures shown in Table 1) revealed the appearance of more than one signal in most ^11^B-NMR spectra. ^11^B-NMR studies conducted in the previous literature [22,29] are very limited for heterocyclic high Fsp^3^ index species and, as such, the novel boron-containing species in this study and those published [1,2,4,30] provide greater insights into intramolecular and intermolecular (e.g., solvent molecules) interactions of B atoms covalently bound to heterocyclic high and low Fsp^3^ index compounds. A vast range of reversible covalent interactions can occur via the empty *p*-orbital of boron, acting as an effective Lewis acid.

We compared the relevant literature ^11^B-NMR data [31,32,33,34,35] of reference compounds purchased from chemical companies [36], those obtained from ongoing projects in our research laboratory, and from borylated systems recently investigated [1,2,4] (Table S1). This allows to build a more comprehensive hypothesis around the equilibria observed in the NMR spectra via, primarily, analysis and assignment of the observed ^11^B-NMR signals.

The synthesis and biological evaluation will shortly be published. All spectra are available in the Supplementary Information.

### 2.1. ^11^B-NMR Data Analysis

**Figure 1:**
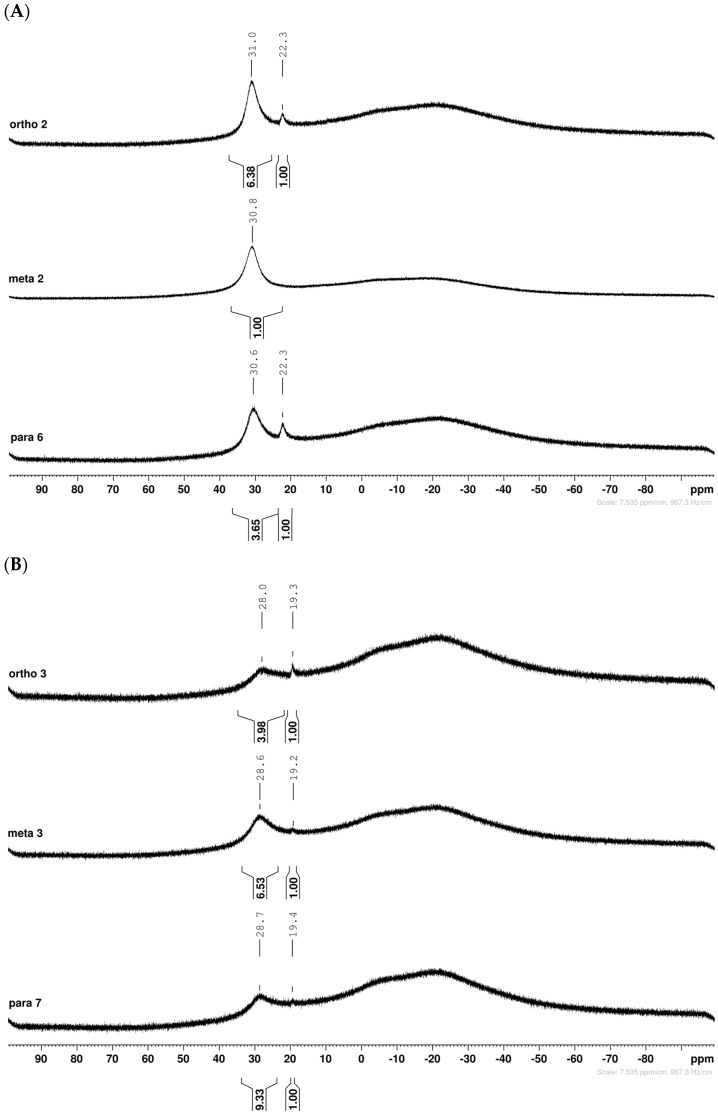

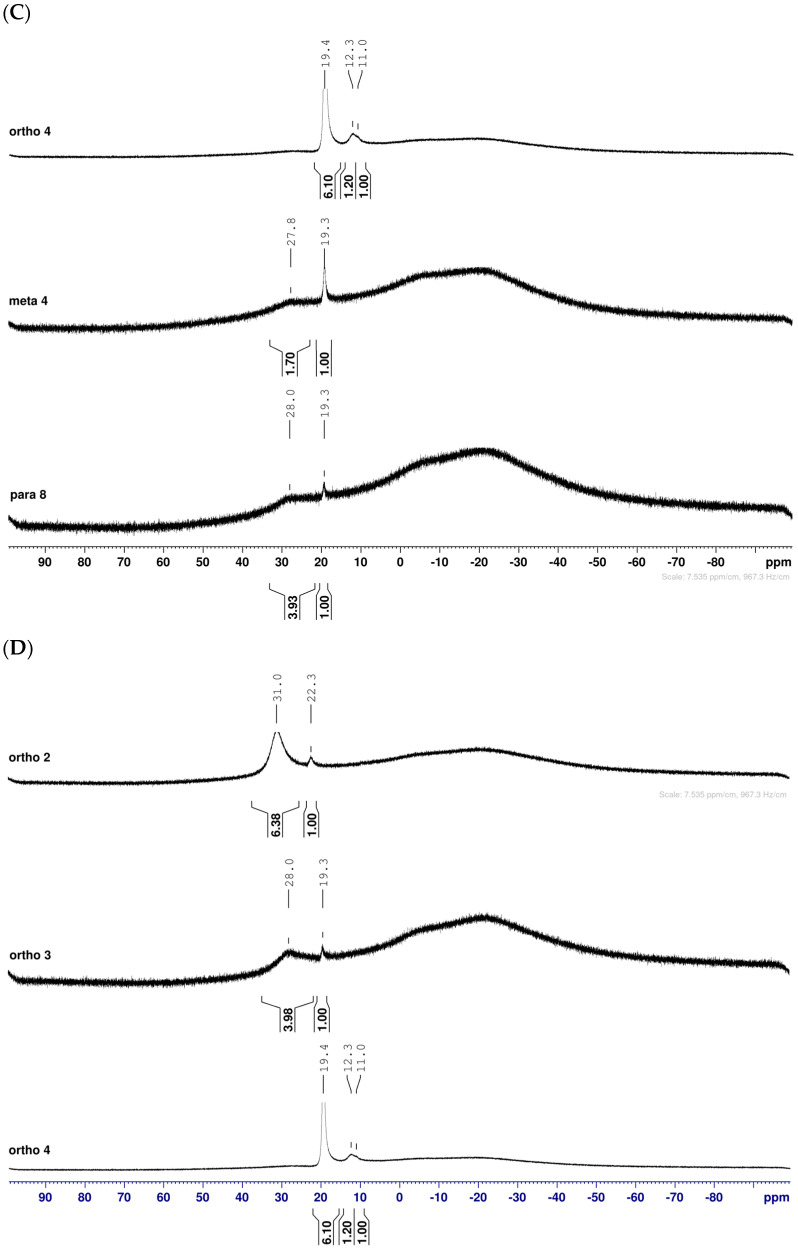

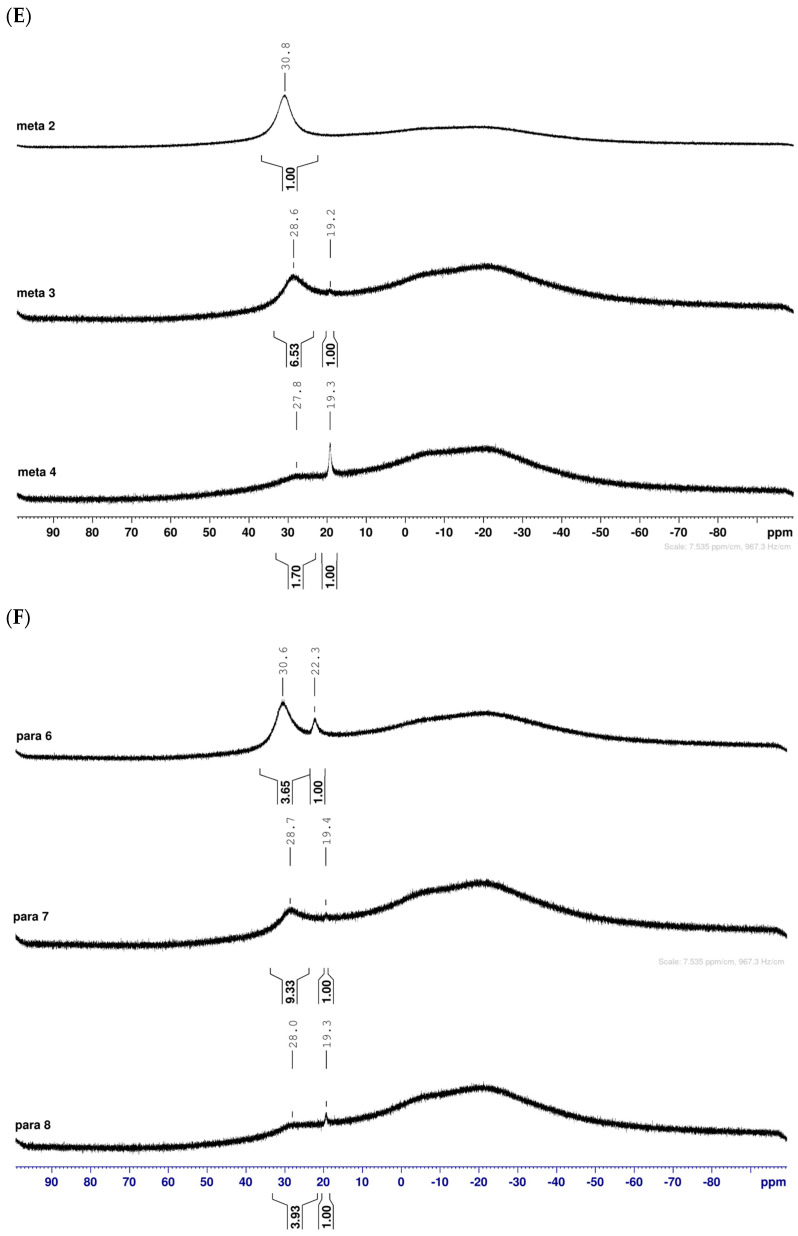

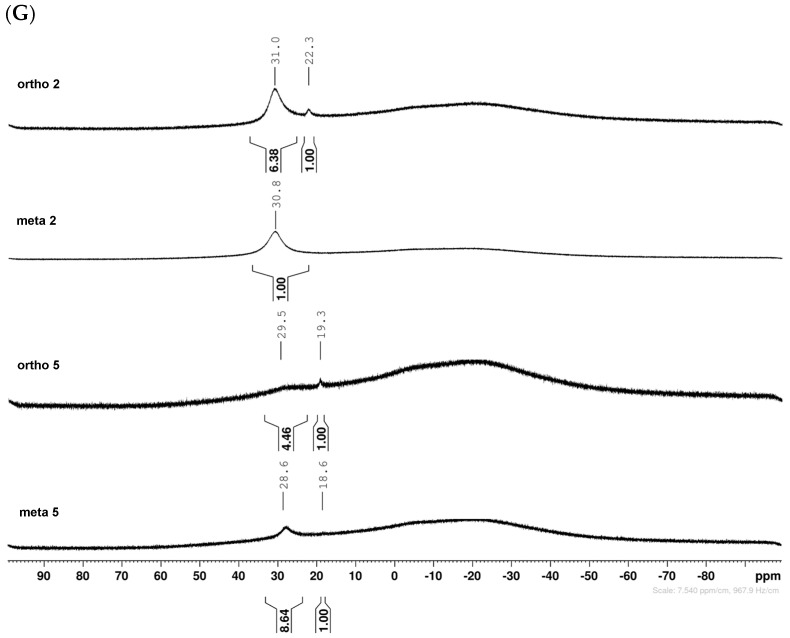
^11^B-NMR spectral comparisons: (**A**) for intermediates **ortho 2**, **meta 2**, and **para 6**; (**B**) for intermediates **ortho 3**, **meta 3**, and **para 7**; (**C**) for target compounds **ortho 4**, **meta 4**, and **para 8**; (**D**) for library **ortho 2**, **ortho 3**, and **ortho 4**; (**E**) for library **meta 2**, **meta 3**, and **meta 4**; (**F**) for library **para 6**, **para 7**, and **para 8**; (**G**) for intermediates **ortho 2** and **meta 2**, and side-products **ortho 5** and **meta 5**.

The borarotation [4] process occurs for all molecules under investigation [borylated iminosugars **para 8**, **meta 4**, and **ortho 4**, intermediates **para 6**, **ortho 2**, **para 7**, **meta 3**, and **ortho 3**, and side products **meta 5** and **ortho 5**], except for **meta 2**. Figure 1 shows stacked plots of the obtained spectra and how boron signals change across the intermediates **ortho 2**, **meta 2** and **para 6** (Figure 1A), intermediates **ortho 3**, **meta 3** and **para 7** (Figure 1B), target compounds **ortho 4**, **meta 4** and **para 8** (Figure 1C), the **ortho** family (Figure 1D), the **meta** family (Figure 1E), the **para** family (Figure 1F), and side-products **ortho 5** and **meta 5** (Figure 1G). The interplay between borarotation and mutarotation occurs for intermediates **para 7**, **meta 3**, and **ortho 3** (with postulated equilibria shown in Table 2). The boron hump is visible between ~10 and ~−40 ppm in the ^11^B-NMR spectra, corresponding to borosilicate compounds contained in NMR tubes and the NMR probe [37,38,39].

#### 2.1.1. Analysis and Comparison of Pinacol Boronates **ortho 2**, **meta 2** and **para 6** (Figure 1A)

The ^11^B-NMR spectra show signals at 30.6 and 22.3 ppm (**para 6**), 30.8 ppm (**meta 2**), and 31.0 and 22.3 ppm (**ortho 2**). In the case of **para 6**, these peaks correspond to the boronate esters of the trigonal planar and partially tetrahedral geometries. The latter likely occurs via a CDCl_3_ molecule datively bonding to a hindered B atom (a slight pink colour occurs which is related to the molecular system). In the case of **meta 2**, the pinacol boronate ester is likely less accessible to nearby CDCl_3_ molecules due to the greater proximity of the iminosugar portion of the molecule, and the partially tetrahedral form is abrogated. In **ortho 2**, the trigonal planar pinacol boronate can be discerned (31.0 ppm) as well as a partially tetrahedral species (22.3 ppm). The latter likely arises from intramolecular interactions between the iminosugar N atom datively bonding into the B atom empty *p*-orbital. This dative bond allows for the formation of an intramolecular 5-membered ring; however, the pinacol carbon backbone sterically hinders the formation of a complete dative interaction and tetrahedral geometry around the B atom (for which a chemical shift of ~10 ppm would be expected) [1,2,4,34,40,41].

#### 2.1.2. Analysis and Comparison of Lactols **ortho 3**, **meta 3** and **para 7** (Figure 1B)

The spectra are further complicated by the presence of a mutarotation equilibrium arising at C-1, where both the α-furanose (*α-fur*) and β-furanose (*β-fur*) anomers arise via the open-chain carbonyl form. Careful examination of the 1D and 2D spectra is important in discerning the mutarotation and borarotation processes [4]. At equilibrium, **para 7**, **meta 3**, and **ortho 3** exist in the respective mutarotation ratios of the *α-fur* anomer: *β-fur* anomer = 1.0:0.4, 1.0:0.5, and 1.0:0.7. Hence, the B atom in both anomers is found primarily in the trigonal planar boronic acid form, as indicated by the respective chemical shifts at 28.7 ppm, 28.6 ppm, and 28.0 ppm. The partially tetrahedral form for both anomers is found at 19.4 ppm, 19.2 ppm, and 19.3 ppm for **para 7**, **meta 3**, and **ortho 3**, respectively. These partially tetrahedral species likely arise intermolecularly from a partial dative bond between the less hindered boronic acid B atom and the O atom lone pair of a D_2_O molecule in **para 7** and **meta 3**, and intramolecularly form a partial dative bond between the less hindered boronic acid group and the N atom lone pair in **ortho 3**. The occurrence of an upfield shift (of ~3 ppm from ~22 ppm to ~19 ppm for **para 7** and **ortho 3**) indicates that the dative bonds are inferred to be slightly shorter and stronger than their corresponding precursors (22.3 ppm for both **para 6** and **ortho 2**), and the quaternisation of the B atom more pronounced.

#### 2.1.3. Analysis and Comparison of Final Iminosugars **ortho 4**, **meta 4** and **para 8** (Figure 1C)

The B atom is found predominantly in the trigonal planar boronic acid form, as indicated by the respective chemical shifts at 28.7 ppm and 27.8 ppm for **para 8** and **meta 4**, respectively. In **ortho 4**, there may be the trigonal planar boronic acid form, but as a minor component of the equilibrium mixture (28.3 ppm, ~10%).

The partially tetrahedral species likely arise intermolecularly from a partial dative bond between the less hindered boronic acid B atom and the O atom lone pair of a D_2_O molecule in **para 8** and **meta 4** and is represented by the chemical shifts at 19.4 ppm and 19.3 ppm. In **ortho 4**, the B atom is found at 19.4 ppm and 12.3/11.0 ppm. The 19.4 ppm signal likely arises intramolecularly from a partial dative bond between the less hindered boronic acid group and the N atom lone pair. The signals at 12.3/11.0 ppm indicate the formation of a stronger dative bond, significantly greater quaternisation of the B atom geometry, and the formation of a more defined intramolecular 5-membered ring.

#### 2.1.4. Analysis of the Signal Integration Ratios

It is important to keep in mind that the signals for the trigonal planar B atom tend to be broad and those for partially tetrahedral/tetrahedral B atoms are significantly sharper. The broadness of the trigonal planar B atoms promotes a certain degree of error in the estimation of the borarotated composition. However, it is crucial to estimate these ratios for application purposes (e.g., drug leads).

For precursors **para 6** and **ortho 2** the ratios between the trigonal planar B species and partially tetrahedral B species are comparable (4.0:1.0 and 6.4:1.0, respectively).

For lactols **para 7**, **meta 3**, and **ortho 3**, the trigonal planar B species is significantly favoured with ratios of 9.3:1.0, 6.5:1.0, and 4.0:1.0, respectively. This is likely due to the higher degree of molecular tumbling in solution upon removal of the pinacol and acetonide protecting groups, which reduces the efficacy of the interaction between nucleophilic atoms and the B empty *p*-orbital. However, intramolecular dative bonding giving rise to a 5-membered ring structure (**ortho 3**) is more favourable over intermolecular dative bonding (**meta 3**, **para 3** with several water molecules passing nearby).

For final iminosugars **para 8**, **meta 4**, and **ortho 4**, the ratio between the trigonal planar B atoms and the partially tetrahedral B atoms is 3.9:1.0 and 1.7:1.0 for **para 8** and **meta 4**, respectively. In the case of **ortho 3**, there may be the trigonal planar B atom (integration ratio: 1.1), and varying degrees of B quarterisation with the B atom spending ~25% of its time in a defined intramolecular 5-membered ring.

#### 2.1.5. Boronic Acid Species **ortho 5** and **meta 5** (Figure 1G)

These species have lost their pinacol protecting group but retain the acetonide protecting group. In both cases, the trigonal planar B species is predominant, as indicated by chemical shifts at 28.6 ppm and 29.5 ppm, respectively. The partially tetrahedral species likely arise from an intermolecular partial dative bond between the less hindered (due to the absence of the pinacol group) boronic acid B atom and the O atom lone pair of a D_2_O molecule in **meta 5**, and an intramolecular partial dative bond between the less hindered boronic acid group and the N atom lone pair in **ortho 5**. The species ratios stand at 8.4:1.0 and 4.5:1.0, respectively. The ratio for **ortho 5** (4.5:1.0) is comparable to that of **ortho 2** (6.4:1.0) and **ortho 3** (4.0:1.0). This is likely due to the proximity in space of the B and N atom in all three molecules, which favours their dative interaction regardless of the protecting groups presence/absence and impact of mutarotation on the degree of molecular tumbling.

Mutarotation seems to impact the degree of molecular tumbling in **meta 3** compared to **meta 5**, mirrored in the relative broadness/sharpness of the boronic acid signals.

### 2.2. ^1^H-, ^13^C- and 2D NMR Data Analysis

NMR data for compounds **meta 3**, **meta 4**, **ortho 3**, **ortho 4**, **meta 5**, and **ortho 5** were analysed in detail. Spectra for protected precursors **meta 2** and **ortho 2** are relatively more straightforward to assign and hence, we did not discuss in detail (all spectra are presented in Figures S1–S20, structures in Table 1, and their postulated equilibria in Table 2).

Table 3 and Table 4 contain ^1^H- and ^13^C-NMR, optical rotation, and melting point data for all 1,4-dideoxy-1,4-iminohexitols available from the chemical literature for stereoisomers (*allo*, *altro*, *galacto*, *gluco*, *ido*, *manno,* and *talo*) of the gulo systems investigated. The selected, relevant *N*-benzylated derivatives are also included. Comparison of these data provide an important insight into three-dimensional shapes around the pyrrolidine ring and how *N*-benzylation impacts conformational states in aqueous solution.

#### 2.2.1. *N*-(3-Methylphenyl boronic acid)-3,6-dideoxy-3,6-imino-d-gulofuranose **meta 3** (Figures S2–S4)

This intermediate is an equilibrium mixture of two predominant conformations, the *α-fur* and the *β-fur* anomers with the boron in a trigonal planar geometry in its acidic form (^11^B-NMR signal at 28.6 ppm, integration 6.5). It is also possible to see minor signals in the ^1^H-NMR, likely arising from the same anomers where the boron is in the boronate partially tetrahedral form (^11^B-NMR signal at 19.2 ppm, integration 1.0). The anomeric ratio for the *α-fur* anomer–*β-fur* anomer is 1.0:0.5. The open-chain form can also be seen via the aldehyde H at 9.86 ppm (integration 0.002). Only the boronic acid *α-fur* and *β-fur* anomers data have been reported. The minor species are visible in the spectra provided in the Supporting Information.

**Aromatic region of the ^1^H-NMR spectrum**. This region is characterised by the presence of two sets of four signals. The signals belonging to the *β-fur* anomer are partially or completely obscured by the signals belonging to the *α-fur* anomer.

The *α-fur* anomer displays COSY correlations between the doublet of doublets at 7.80 ppm (ArH^C^) with the triplet at 7.48 ppm (ArH^D^) and the doublet at 7.78 ppm (ArH^A^). The doublet of triplets at 7.58 ppm (ArH^E^) is COSY correlated to ArH^D^ and ArH^A^.

HSQC correlations are observed for the doublet of doublets at 7.80 ppm to a carbon signal at 135.4 ppm (ArC^C^), the doublet at 7.78 ppm to the carbon signal at 136.2 ppm (ArC^A^), the doublet of triplets at 7.58 ppm to the carbon signal at 133.5 ppm (ArC^E^), and the triplet at 7.48 ppm to the carbon at 128.9 ppm (ArC^D^). The aromatic carbon directly bonded to the B atom is not discernible. The other quaternary aromatic carbon is at 142.4 ppm, which is HMBC correlated with 7.58 ppm (ArH^E^).

Several β-anomer correlations are obscured by the correlations for the α-anomer. The *β-fur* anomer displays COSY correlations between the peaks at 7.82 and 7.76 ppm (obscured, ArH^A^) and a doublet of doublets at 7.60 ppm (ArH^C^). The triplet at 7.50 ppm (ArH^D^) is COSY correlated to the signals at 7.60 ppm and 7.27 ppm (ArH^E^). Also, ArH^E^ is correlated to ArH^A^.

HSQC correlations occur from obscured signals at 7.82–7.76 ppm and a partially obscured signal at 7.60 ppm to carbon signals at 135.9 (ArC^A^) and 135.5 ppm (ArC^C^), respectively. The aromatic carbon directly bonded to the B atom is not discernible. The other quaternary aromatic carbon is at 139.6 ppm, which is HMBC correlated with 7.27 ppm (ArH^E^).

**Sugar region of the ^1^H-NMR spectrum**. This region has many overlapping signals, with assignments made using the correlations found in the 2D spectra (HSQC, HMBC, and COSY), and comparison to analogous molecules if the NMR data were not sufficient.

The *α-fur* anomer was assigned based on the *J* coupling between H-1 and H-2 (4.3 Hz), which indicates a dihedral angle in the range of 50° between these atoms and points to a relative *cis* arrangement. H-1 is a highly deshielded atom at 5.44 ppm as a doublet, which is COSY correlated to a multiplet in the range of 4.42–4.35, indicating the presence of H-2. This area is heavily crowded with H-5, *β-fur* H-2, and flanked by ArCH_2_ doublets. A multiplet at 4.27–4.20 ppm is COSY correlated to an apparent doublet at 4.93 ppm. These are assigned as H-3 and H-4, respectively. H-4 is obscured by the *β-fur* H-4, an apparent doublet at 4.91 ppm. The integrations for these signals are not reliable, hindering the assignment of a signal of the anomers, as the degree of overlap significantly impacts the assignment process. H-4 correlates to a signal located at 4.42–4.35 ppm, which corresponds to H-5. The H-5 signal is correlated to a multiplet at 3.60–3.52 ppm, assigned to H-6, and to a doublet of doublets at 3.49 ppm, assigned to H-6′. The doublet at 4.51 ppm is COSY correlated to the doublet at 4.44 ppm, with a *J* coupling value of 13.1 Hz, which corresponds to ArC*H^a^*H^b^ and ArCH^a^*H^b^*.

HSQC correlations: H-1 correlates to a broad signal at 99.0 ppm (width 89 Hz), H-2 and H-3 correlate to a broad signal at 72.7 ppm (width 68 Hz), H-4 correlates to a broad signal at 78.9 ppm (width 88 Hz), H-5 correlates to 67.4 ppm, H-6 and H-6′ correlate to a broad signal at 61.9–59.7 ppm (width 226 Hz), and ArC*H^a^*H^b^ and ArCH^a^*H^b^* correlate to a broad signal at 59.7–58.4 ppm (width 170 Hz).

The *β-fur* anomer was assigned based on the *J* coupling between H-1 and H-2 (2.1 Hz), which indicates a dihedral angle of 120° between these atoms and points to a relative trans arrangement. H-1 is a deshielded atom at 5.29 ppm as a doublet. H-1 does not COSY correlate to any other signal. Hence, locating H-2 was difficult and achieved by exclusion, once all other signals had been assigned. H-2 is likely located in the range of 4.42–4.35 ppm. H-3 is a doublet of doublets at 4.16 ppm, which is COSY correlated to an apparent doublet at 4.91 ppm. The *J* coupling between H-3 and H-4 is 5.6/5.8 Hz. H-4 is obscured by the *α-fur* H-4, an apparent doublet at 4.93 ppm. The integrations for these are not reliable enough to assign the signal to one of the anomers, as the degree of overlap significantly impacts the assignment process. H-4 correlates to an obscured signal located at 4.49–4.44 ppm, which is assigned to H-5. The H-5 signal is correlated to a doublet of doublets at 3.74 ppm and the obscured signal (presumably a doublet of doublets as well) at 3.52–3.47 ppm is assigned to H-6 and H-6′. The doublet at 4.57 ppm is COSY correlated to the doublet at 4.35 ppm, with a *J* coupling value of 13.0 Hz, which corresponds to ArC*H^a^*H^b^ and ArCH^a^*H^b^*.

HSQC correlations are as follows: H-1 correlates to a broad signal at 103.1 ppm (width 29 Hz), H-2 correlates to a broad signal at 72.7 ppm (width 68 Hz), H-3 correlates to a signal at 72.2 ppm, H-4 correlates to a slightly broad signal at 80.7 ppm (width 30 Hz), H-5 to 67.5 ppm, H-6 and H-6′ correlates to a broad signal at 61.9–59.7 ppm (width 226 Hz), and ArC*H^a^*H^b^ and ArCH^a^*H^b^* correlates to a broad signal at 59.7–58.4 ppm (width 170 Hz).

#### 2.2.2. *N*-(3-Methylphenyl boronic acid)-1,4-dideoxy-1,4-imino-l-gulitol **meta 4** (Figure S5)

This final compound is found as an equilibrium mixture between the trigonal planar boronic acid form and the partially tetrahedral boronate form where one D_2_O molecule datively bonds via an O atom lone pair into the B empty *p*-orbital, as indicated by the ^11^B-NMR signals at 27.8 ppm and 19.3 ppm. It is postulated the tetrahedral boronate form arises from the interaction with a D_2_O molecule because intramolecular interactions are unlikely due to geometry constraints, similar in the case of **para 8**. The only case where intramolecular dative bonds are geometrically possible is in the **ortho** series. For **meta 4**, it is possible to see two sets of signals in the ^1^H- and ^13^C-NMR spectra, reflecting the borarotated equilibrium mixture. The trigonal planar boronic acid species and the partially tetrahedral boronate species have an approximate ratio of 1.3:1.0, as indicated by the similar integration ratios in ^1^H- and ^11^B-NMR spectra.

**Aromatic region of the ^1^H-NMR spectrum**. The boronic acid species displays principal COSY correlations between the doublet at 7.66 ppm to an apparent triplet at 7.27 ppm with a coupling constant of 7.5/7.3 Hz. The signals corresponded to ArH^C^ and ArH^D^. ArH^D^ is also correlated to a doublet at 7.43 ppm with a coupling constant of 7.5 Hz (ArH^E^). ArH^A^, ArH^C^, and ArH^E^ are expected to be slightly deshielded compared to ArH^D^ due to a d^+^ arising from delocalisation of *p*-electrons into the C-B bond.

The signal at 7.72 ppm is a singlet and not correlated to any other signal and assigned to ArH^A^.

HSQC correlations occur from the signal at 7.72 ppm (ArH^A^) to a carbon signal at 137.6 ppm, from 7.66 ppm (ArH^C^) to a carbon at 136.5 ppm, from 7.43 ppm (ArH^E^) to a carbon signal at 134.2 ppm, and from 7.27 ppm (ArH^D^) to a carbon signal at 129.6 ppm. The aromatic C directly bonded to the B atom is not discernible.

The boronate species displays principal COSY correlations between the apparent triplet at 7.10 ppm to the doublets at 6.83 ppm and 6.71 ppm with coupling constants of 8.0 Hz. The triplet corresponded to ArH^D^, and the two doublets to ArH^C^ and ArH^E^, respectively. These would be expected to be slightly deshielded compared to ArH^D^ due to a d^+^ on ArH^A^, ArH^C^, and ArH^E^ arising from delocalisation of *p*-electrons onto the C-B bond. The signal at 6.81 ppm is a singlet and not correlated to any other signal and assigned to ArH^A^.

HSQC correlations occur for the signal at 6.81 ppm (ArH^A^) to the carbon signal at 118.8 ppm, from 6.83 ppm (ArH^C^) to the carbon signal at 118.1 ppm, from 6.71 ppm (ArH^E^) to the carbon signal at 123.3 ppm, and from 7.10 ppm (ArH^D^) to the carbon signal at 131.5 ppm. The two aromatic C_quat_ are not discernible.

**Sugar region of the ^1^H-NMR spectrum**. This region has many overlapping signals, with assignments made using the correlations in the 2D spectra (COSY and HSQC), and comparisons to the analogous molecules when the NMR data were not sufficient.

The boronic acid species was assigned based on the *J* coupling between the doublet at 4.55 ppm, which is COSY correlated to the doublet at 3.99 ppm, with a *J* coupling value of 12.9 Hz, which corresponds to ArC*H^a^*H^b^ and ArCH^a^*H^b^*. The signal at 3.31–3.20 ppm, appearing as a multiplet, is assigned to H-1 and COSY correlated to the multiplet at 3.09–2.97 ppm, assigned as H-1′. Both signals are HSQC correlated to a single slightly broad carbon signal at 54.3 ppm (width 27 Hz). H-1 and H-1′ both COSY correlate to a group of signals as a multiplet at 4.26–4.05 ppm. This indicates that H-2 is contained in this multiplet. The side signals at one end of this multiplet are correlated with those at the other end, which indicates that H-3 is also contained therein. From this multiplet, a COSY correlation occurs at 3.63–3.53 ppm and 3.52 ppm. This indicates that the 4.26–4.04 ppm multiplet contains also H-5, which correlates to two signals corresponding to H-6 and H-6′. The multiplet at 3.63–3.53 ppm integrates two H atoms and H-4 is placed in this multiplet as well. The signals at 3.63–3.53 ppm are COSY correlated to 3.52 ppm and both signals correlate to one single carbon signal at 64.7 ppm (C-6).

HSQC correlations occur from 4.55 ppm and 3.99 ppm to a single slightly broad carbon signal at 62.5 ppm (width 21 Hz), which is assigned to ArCH_2_. The multiplet at 3.63–3.53 ppm also correlates to 70.5 ppm and is assigned to C-4. The multiplet at 4.26–4.05 ppm correlates to the signals at 72.2 ppm, 71.6 ppm, and 70.1 ppm, which are assigned to C-3, C-2, and C-5, respectively.

The boronate species was assigned based on the *J* coupling between the doublet at 4.64 ppm which is COSY correlated to an obscured signal at 4.08 ppm, with a *J* coupling value of 12.9 Hz and corresponds to ArC*H^a^*H^b^ and ArCH^a^*H^b^*. The multiplet at 3.67–3.62 ppm is assigned to H-1 and it is COSY correlated to the multiplet at 3.09–2.97 ppm, assigned to H-1′. H-1 is HSQC correlated to a slightly broad carbon signal at 57.8 ppm (width 17 Hz). Other COSY correlations are complex to discern due to the extensive signal overlaps. The multiplet at 3.63–3.53 ppm is assigned to H-6 and H-4, and the apparent triplet at 3.49 ppm is assigned to H-6.

HSQC correlations occur from 4.64 ppm and 4.08 ppm to a single slightly broad carbon signal at 62.7 ppm, which is assigned to ArCH_2_. The multiplet at 3.63–3.53 ppm also correlates to 70.5 ppm and is assigned to C-4. The multiplet at 4.26–4.05 ppm is correlated to signals at 72.17 ppm, 71.62 ppm, and 70.10 ppm, which are assigned to C-3, C-2, and C-5, respectively. The two C_quat_ are not discernible.

#### 2.2.3. *N*-(2-Methylphenyl boronic acid)-3,6-dideoxy-3,6-imino-d-gulofuranose **ortho 3** (Figures S7–S9)

This intermediate is an equilibrium mixture of two predominant conformations, the *α-fur* and the *β-fur* anomers with the boron in trigonal planar geometry in its acidic form (^11^B-NMR signal at 28.0 ppm, integration 4.0). It is also possible to see minor signals in the ^1^H-NMR, likely arising from the same anomers where the boron is in the boronate form (^11^B-NMR signal at 19.3 ppm, integration 1.0). The partially tetrahedral boronate species likely arises from an intramolecular dative bond from the N lone pair to the B empty *p*-orbital, to give a 5-membered ring structure. The anomeric ratio for the *α-fur* anomer–*β-fur* anomer is 1.0:0.7.

**Aromatic region of the ^1^H-NMR spectrum**. This region is characterised by the overlapping of the four signals corresponding to ArH^B^ and ArH^C^, whereas ArH^A^ and ArH^D^ are relatively isolated, albeit ArH^D^ (*β-fur*) overlaps with ArH^B^ and ArH^C^.

The *α-fur* anomer displays the four aromatic H atoms at 7.85 (app-dd), 7.58–7.54 (m), 7.50 (app-dd) ppm as ArH^A^, ArH^B^ and ArH^C^, and ArH^D^, respectively,. COSY correlations only clearly show the correlation between ArH^A^ and ArH^D^. HSQC shows correlations between ArH^A^ (7.85 ppm) and the signal at 135.7 ppm (ArC^A^), from ArH^B^ and ArH^C^ (7.58–7.54 ppm range) to the signals at 131.3 and 129.6 ppm (ArC^B^ and ArC^C^, unclear assignment), and from ArH^D^ (7.50 ppm) to the signal at 132.1 ppm (ArC^D^).

The *β-fur* anomer displays the four aromatic H atoms at 7.88 (app-dd), 7.61–7.56 (m), and 7.54 (app-dd) ppm as ArH^A^, ArH^B^ and ArH^C^, and ArH^D^, respectively,. No discernible COSY correlations are observed for this anomer. HSQC shows correlations between ArH^A^ (7.88 ppm) to the signal at 135.9 ppm (ArC^A^), from ArH^B^ and ArH^C^ (7.61–7.56 ppm range) to the signals at 131.5 and 129.8 ppm (ArC^B^ and ArC^C^), and from ArH^D^ (7.54 ppm) to the signal at 132.2 ppm (ArC^D^).

**Sugar region of the ^1^H-NMR spectrum**. This region has many overlapping signals, with assignments made using the correlations in the 2D spectra (COSY and HSQC), the information provided in the DEPT spectrum, and comparison to analogous molecules where the NMR data were not sufficient.

The *α-fur* anomer was assigned based on the *J* coupling between H-1 and H-2 (4.3 Hz), which indicates a Karplus angle of 50° between these atoms and points to a relative *cis* arrangement. It is known that H-2 is *cis* to H-1. H-1 is a deshielded atom and is located at 5.48 ppm as a doublet. This signal has a (weak) COSY correlation to a signal centred at 4.33 ppm, which indicates H-2. H-2 appears as a multiplet in the range of 4.39–4.28 ppm. It has no discernible COSY correlations to H-3 which is adjacent located in the range of 4.31–4.22 ppm. H-3 is a multiplet and was assigned by exclusion, after all other signals were assigned, and through comparison to analogous molecules (e.g., H-3 of *α-fur* **meta 3** is a multiplet at 4.27–4.20 ppm). H-4 is the next most deshielded H atom and is a partially obscured multiplet in the range of 5.06–4.99 ppm. It is obscured by the H-4 (*β-fur*, apparent triplet at 5.06 ppm). The integrations for these signals are not reliable enough to assign the signal to one of the anomers, as the degree of overlap significantly impacts the assignment process. H-4 correlates to a signal located at 4.47 ppm and 4.27 ppm. The former is H-5, which is obscured by ArCH^a^*H^b^* (a doublet centred at 4.45 ppm) and is assigned as a multiplet in the range of 4.49–4.44 ppm. The latter is H-3. H-5 is COSY correlated to one apparent singlet in the range of 3.55–3.51 ppm, which integrates two H atoms and corresponds to the signal of H-6 and H-6′. The doublet at 4.45 ppm for ArCH^a^*H^b^* is COSY correlated to the signal that is partially obscured by H_2_O (4.79 ppm), which corresponds to the ArC*H^a^*H^b^, centred at 4.76 ppm.

HSQC correlations are as follows: H-1 at 5.48 ppm correlates to the broad signal at 99.5 ppm (width 59 Hz), H-2 at 4.39–4.28 ppm correlates to 73.4 ppm, H-3 at 4.31–4.22 ppm correlates to 72.0 ppm, H-4 at 5.06–4.99 ppm correlates to the broad signal at 79.4 ppm (width 65 Hz), H-5 at 4.49–4.44 ppm correlates to 67.2 ppm, H-6 and H-6′ at 3.55–3.51 ppm correlates to the broad signal at 60.1 ppm (width 105 Hz), and ArC*H^a^*H^b^ at 4.76 ppm and ArCH^a^*H^b^* at 4.45 ppm correlate to the broad signal at 60.7 ppm (width 32 Hz).

The ***β-fur* anomer** was assigned starting from the H-1 which appears as a singlet at 5.33 ppm. H-1 does not COSY correlate to any other signal. This made locating H-2 difficult and was achieved by exclusion once all other signals were assigned. The signal at 5.06 ppm corresponds to H-4 and appears as a partially obscured apparent triplet. This signal is COSY correlated to two signals: one at 4.18 ppm and one centred at 4.65 ppm. The latter is obscured due to overlapping with the signals related to ArC*H^a^*H^b^ and ArCH^a^*H^b^*. These H atoms appear as a multiplet in the range of 4.69–4.60 ppm. The H-5 signal is COSY correlated to two signals correlated to each other, namely H-6 and H-6′. These signals appear as two doublets of doublets at 3.73 and 3.59 ppm. The signal at 4.18 ppm is H-3, appearing as a doublet, and is correlated solely to H-4. H-2 is located in the range of 3.65–3.58 ppm, under the signal related to H-6′. This signal is significantly removed from the expected chemical shift at around 4.3 ppm. However, it is HSQC correlated to the carbon signal at 75.6 ppm, which is close to C-2 (*α-fur*) and appears at 73.5 ppm.

HSQC correlations are as follows: H-1 at 5.33 ppm correlates to the carbon at 102.9 ppm, H-2 at 3–65-3.58 ppm correlates to 75.6 ppm, H-3 at 4.18 ppm correlates to a slightly broad signal at 73.4 ppm (width 27 Hz), H-4 at 5.06 ppm correlates to a slightly broad signal at 81.8 ppm (width 29 Hz), H-5 at 4.65 ppm correlates to 67.4 ppm, H-6 and H-6′ at 3.73 ppm and 3.59 ppm correlate to a broad signal at 60.1 ppm (width 105 Hz), and ArC*H^a^*H^b^ and ArCH^a^*H^b^* at 4.69–4.60 ppm correlate to the broad signal at 60.1 ppm (width 105 Hz).

#### 2.2.4. *N*-(2-Methylphenyl boronic acid)-1,4-dideoxy-1,4-imino-l-gulitol **ortho 4** (Figures S10–S12)

This final compound is found as an equilibrium mixture between two boronate ammonium species, one partially tetrahedral and one tetrahedral. Both forms arise from intramolecular dative bonding of the N lone pair into the B empty *p*-orbital, as indicated by the ^11^B-NMR signals at 19.4 ppm and 12.3/11.0 ppm, with an integration ratio of 6.1:2.2 (combined). It is possible to see two main sets of signals in the ^1^H- and ^13^C-NMR spectra, reflecting the borarotated equilibrium mixture. There may be a boronic acid species as a minor component at 28.3 ppm, with an integration of 1.1.

**Aromatic region of the ^1^H-NMR spectrum**. The partially tetrahedral boronate ammonium species displays principal COSY correlations between the apparent doublet of doublet at 7.48 ppm and two doublets of doublets of doublets at 7.33 ppm and 7.31 ppm, which correspond to ArH^C^ and ArH^D^. The signal at 7.48 ppm is assigned to ArH^B^. The signal at 7.33 ppm is also COSY correlated to 7.31 ppm and 7.17 ppm. The former signal is correlated to 7.17 ppm (ArH^E^). ArH^B^ and ArH^D^ are expected to be slightly deshielded compared to the other aromatic H nuclei due to a d^+^ arising from delocalisation of *p*-electrons into the C-B bond.

HSQC correlations are observed between the signal at 7.48 ppm and the carbon signal at 129.4 ppm (ArC^B^), from 7.33 ppm to the carbon at 128.2 ppm (ArC^C^), from 7.31 ppm to the carbon signal at 127.6 ppm (ArC^D^), and from 7.17 ppm to the carbon signal at 122.7 ppm (ArC^E^). The aromatic C directly bonded to the B atom is not discernible. The other quaternary aromatic carbon is at 140.6 ppm.

The tetrahedral boronate ammonium species displays principal COSY correlations between the apparent doublet of triplets at 7.88 ppm and the apparent doublet at 7.52 ppm, and between the doublet of doublet at 7.40 ppm to the multiplet at 7.05–5.95 ppm.

HSQC correlations are observed between the signal at 7.40 ppm and the carbon signal at 132.2 ppm, from 7.52 ppm to the carbon at 130.2 ppm, from 7.88 ppm to the carbon signal at 127.9 ppm, and the signals between 7.05 and 5.95 ppm to the carbon signal at 115.9 ppm. The two C_quat_ are not discernible.

**Sugar region of the ^1^H-NMR spectrum**. This region has several overlapping signals, with assignments made using the correlations in the 2D spectra (HSQC, HMBC, and COSY), and comparison to analogous molecules if the NMR data are not sufficient.

The partially tetrahedral boronate ammonium species was assigned based on the *J* coupling between the doublet at 4.45 ppm which is COSY correlated to the partially obscured doublet at 4.29 ppm, with a *J* coupling value of 15.2 Hz, which corresponds to ArC*H^a^*H^b^ and ArCH^a^*H^b^*. The doublet of doublet at 3.20 ppm is assigned to H-1′ and COSY correlated to the multiplet at 3.55–3.45 ppm where the doublet of doublet is discernible and assigned to H-1. Both signals are COSY correlated to an apparent doublet of doublet at 4.60 ppm, assigned to H-2. This signal is correlated to the multiplet at 4.53–4.45 ppm, which is assigned to contain H-3 and H-5. This multiplet is COSY correlated to 3.55–3.45 ppm, which indicates that H-4 is contained therein (with H-1′). The multiplet at 4.53–4.45 ppm is also correlated to the apparent doublet at 3.25 ppm, which integrates to two hydrogens and is assigned to H-6 and H-6′.

HSQC correlations occur from 4.45 ppm to 4.29 ppm to a single slightly broad carbon signal at 67.3 ppm, which is assigned as ArCH_2_. The multiplet at 4.53–4.45 ppm correlates to the carbon signals at 72.9 ppm and 70.4 ppm, which are assigned to C-3 and C-5, respectively. The multiplet at 3.55–3.45 ppm is correlated to 72.7 ppm, which is assigned to C-4. The signal at 3.25 ppm also correlates to 63.8 ppm (C-6). The signal at 4.60 ppm correlates to 71.6 ppm (C-2) and the signals at 3.20 ppm and 3.55–3.45 ppm correlate to a single signal at 57.4 ppm (C-1).

The tetrahedral boronate ammonium species displays an obscured signal at 4.50–4.39 ppm and is HSQC correlated to a carbon signal at 55.2 ppm. The multiplet at 4.20–4.10 ppm is COSY correlated to 3.72–3.62 ppm and 4.50–4.39 ppm. The multiplet at 3.85–3.75 ppm is correlated to the multiplet at 3.72–3.62 ppm, assigned to the H-6 and H-6′ nuclei. The multiplet at 3.40–3.30 seems to be correlated to the multiplet at 1.45–1.28 ppm. The former is assigned as H-2 and the latter as H-1 and H-1′.

HSQC correlations occur from the multiplet at 1.45–1.28 ppm to the signal at 9.9 ppm, which is assigned to C-1. The signal at 4.20–4.10 ppm correlates to the carbon signal at 68.1 ppm, and the signals at 3.85–3.75 ppm and 3.72–3.62 ppm correlate to the carbon signal at 62.9 ppm, which is assigned to C-6.

#### 2.2.5. *N*-(3-Methylphenyl boronic acid)-3,6-dideoxy-3,6-imino-1,2-*O*-isopropylidene-α-d-glucofuranose **meta 5** (Figures S13–S17)

This final compound is found as an equilibrium mixture between the trigonal planar boronic acid form and the partially tetrahedral boronate form where one D_2_O molecule datively bonds via an O atom lone pair into the B empty *p*-orbital, as indicated by the ^11^B-NMR signals at 28.6 ppm and 18.6 ppm, with an integration ratio of 8.6:1.0. It is possible to see a major set of signals and a minor one in the ^1^H- and ^13^C-NMR spectrum, reflecting the borarotated equilibrium mixture.

**Aromatic region of the ^1^H-NMR spectrum**. The boronic acid species displays four aromatic signals at 7.57 ppm (doublet), 7.49 ppm (doublet), 7.37 ppm (doublet of triplets), and 7.32 ppm (app-t). These were assigned as ArH^A^, ArH^C^, ArH^E^, and ArH^D^ based on coupling constants, multiplicities, and expected deshielding effects arising from delocalisation of *p*-electrons into the C-B bond.

HSQC correlations occur between the signal at 7.57 ppm and the carbon signal at 135.2 ppm (ArC^A^), the signal at 7.49 ppm correlates to 133.4 ppm (ArC^C^), the signal at 7.37 ppm correlates to 131.4 ppm (ArH^E^), and the signal at 7.32 ppm correlates to 128.7 ppm (ArH^D^). The aromatic C directly bonded to the B atom is a broad signal at 134.2–135.3 ppm. The other quaternary aromatic carbon is at 138.5 ppm.

**Sugar region of the ^1^H-NMR spectrum**. This region is relatively unencumbered with assignments extrapolated from the 1D spectra and the correlations in 2D spectra (HSQC and HMBC), and comparison to analogous molecules if the NMR data were not sufficient.

The boronic acid species is assigned from the deshielded H-1, a doublet located at 5.93 ppm, which COSY correlated to a doublet at 4.46 ppm, assigned to H-2. The signals at 2.83 ppm and 2.53 ppm are two doublets of doublets are HSQC correlated to the single carbon signal at 60.3 ppm (C-6) and correspond to H-6 and H-6′. The geminal *J* coupling is 10.5 Hz and the vicinal ones to H-5 are 3.5 Hz and 5.7 Hz. The signal at 4.13 ppm displays three *J* coupling constants of 5.6 Hz, 5.5 Hz, and 3.7 Hz, assigned to H-5. The apparent triplet at 4.74 ppm displays a *J* coupling constant of 5.5 Hz, corresponds to H-4. The partially obscured doublet at 3.30 ppm is assigned to H-3. The two doublets at 3.91 ppm and 3.59 ppm, coupled to each other (*J* 13.2 Hz) and HSQC correlated to a single signal at 59.9 ppm, are assigned to the two ArCH_2_.

HSQC correlations occur between 5.93 ppm and 108.8 ppm (C-1), 4.46 ppm and 85.8 ppm (C-2), 4.74 ppm and 84.8 ppm (C-4), and 4.13 ppm and 71.2 ppm (C-5). The acetonide C_quat_ is at 113.4 ppm.

For the boronated species, not all signals are visible. The aromatic signals at 7.12 ppm are a triplet of a doublet which is assigned to ArH^C^. The signal at 6.79 ppm, a doublet of doublets, is assigned to ArH^E^. The signal at 7.32 ppm is assigned to ArH^D^. The sugar region shows a deshielded doublet at 5.83 ppm, which is assigned to H-1. The doublet at 4.49 ppm is assigned to H-2, and the doublet at 4.54 ppm is assigned to H-4. The signals at 1.61 ppm and 1.59 ppm are assigned to H-6 and H-6′.

#### 2.2.6. *N*-(2-Methylphenyl boronic acid)-3,6-dideoxy-3,6-imino-1,2-*O*-isopropylidene-α-d-gulofuranose **ortho 5** (Figures S18–S20)

The major–minor species ratio is ~4.2:1.0 as determined from the ^1^H-NMR spectrum, corresponding to the trigonal planar and partially tetrahedral boronate species formed via intramolecular interaction of the N with the B atom. This ratio is reflected in the ^11^B-NMR spectrum, which shows a 4.5:1.0 integration ratio.

**Aromatic region of the ^1^H-NMR spectrum**. The major boronic acid species displays four aromatic signals at 7.74 ppm (apparent singlet), 7.77–7.73 ppm (obscured), 7.53 ppm (doublet of doublets), and 7.48 ppm (apparent triplet). These are assigned to ArH^B^, ArH^E^, ArH^D^, and ArH^C^ based on coupling constants, multiplicities, and expected deshielding effects arising from delocalisation of π-electrons into the C-B bond. The main COSY correlations arise between signals at 7.75 ppm and 7.48 ppm, and at 7.53 ppm and 7.48 ppm.

HSQC correlations occur between the signal at 7.75 ppm and the carbon at 135.0 ppm (ArC^B^), 7.77–7.73 ppm and 133.1 ppm (ArC^E^), 7.53 ppm and 132.4 ppm (ArC^D^), and 7.48 ppm to 128.3 ppm (ArC^C^). The aromatic C directly bonded to the B atom is not discernible and the other ArC_quat_ is at 135.9 ppm.

**Sugar region of the ^1^H-NMR spectrum**. This region is relatively unencumbered with assignments made by analysing the 1D spectra, utilising the correlations in 2D spectra (COSY, HSQC and HMBC), and comparison to analogous molecules if the NMR data are not sufficient.

The boronic acid species is assigned from the deshielded H-1 signal, a doublet located at 5.96 ppm and is COSY correlated to a doublet at 4.36 ppm via a *J* coupling of 3.7 Hz. This is assigned to H-2.

The two doublets at 4.88 ppm and 3.48 ppm, coupled to each other (5.5/5.7 Hz) and HSQC correlated to a single signal at 71.1 ppm, are assigned to the two ArCH_2_. The signal at 3.83 ppm is assigned to H-3. The partially obscured signal at 4.89–4.83 ppm, whose multiplicity is not discernible, is assigned to H-4. H-4 is COSY correlated to the signal at 4.31 ppm (a doublet of doublet of doublets, H-5). H-5 is COSY correlated to the two signals at 2.93 ppm and 2.83 ppm, assigned to H-6 and H-6′, respectively. H-6 and H-6′ are also COSY correlated to each other and HSQC correlated to the single carbon signal at 58.8 ppm (C-6). The geminal *J* coupling is 11.4 Hz and the vicinal ones to H-5 are 4.2 Hz and 6.0 Hz. HSQC correlations occur between 5.96 ppm and 106.7 ppm (C-1), 4.36 ppm and 83.9 ppm (C-2), 4.89–4.83 ppm and 83.4 ppm (C-4), and 4.31 ppm and 69.1 ppm (C-5). The acetonide C_quat_ is at 113.0 ppm.

**Aromatic region of the ^1^H-NMR spectrum**. The minor boronate species displays four aromatic signals at 7.32 ppm (triplet), 6.97 ppm (doublet of triplets), 6.92–6.90 ppm, and 6.89 ppm (doublet of doublet of doublet). These were assigned as ArH^D^, ArH^E^, ArH^B^, and ArH^C^ based on coupling constants, multiplicities and expected deshielding effects arising from delocalisation of *p*-electrons into the C-B bond. The main COSY correlations arise between signals at 7.32 ppm and 6.97 ppm and 6.89 ppm.

HSQC correlations occur between the signal at 7.32 ppm and the carbon at 130.1 ppm (ArC^D^), 6.97 ppm and 122.0 ppm (ArC^E^), 6.92–6.90 ppm and 116.6 ppm (ArH^D^). The two aromatic C_quat_ directly bonded to the B atom and ArH^C^ are not discernible.

**Sugar region of the ^1^H-NMR spectrum**. This region relatively unencumbered with assignments made analysing 1D spectra, correlations in the 2D spectra (HSQC and HMBC), and comparison to analogous molecules if the NMR data are not sufficient.

The partially obscured, deshielded H-1, corresponding to the doublet located at 5.98 ppm, is COSY correlated to a doublet at 4.40 ppm via a *J* coupling of 3.7 Hz. This is assigned to H-2.

The signal at 3.75 ppm is assigned to H-3. The partially obscured signal at 2.96 ppm is assigned to H-5. H-6 and H-6′ are found at 2.95–2.90 ppm (obscured) and 2.84–2.79 ppm (obscured) with both signals being HSQC correlated to one single carbon atom at 58.6 ppm (C-6). The two doublets at 4.85–4.80 ppm (obscured) and 3.46 ppm (partially obscured doublet), coupled to each other (5.9 Hz) and HSQC correlated to a single signal at 71.2 ppm, are assigned as the two ArCH_2_.

HSQC correlations occur between 5.98 ppm and 106.7 ppm (C-1), and 3.75 ppm and 58.4 ppm (C-3). The acetonide C_quat_, C-2, H-4, C-4, and C-5 are not discernible.

## 3. Materials and Methods

### 3.1. Numbering System

Spectroscopic data for all compounds are assigned based on a numbering system derived from systematic naming of materials according to IUPAC recommendations on carbohydrate nomenclature [74]. The numbering is given in Table 2 by the red numbers and letters on selected structures.

### 3.2. General Nuclear Magnetic Resonance (NMR) Experimental

Spectra were recorded on a Bruker Ascend^TM^ 400 in deuterated solvent as stated. Chemical shifts (δ) are quoted in ppm and coupling constants (*J*) in Hz. Residual signals from the CDCl_3_ (7.26 ppm for ^1^H-NMR and 77.16 ppm for ^13^C-NMR), deuterated methanol (3.31 ppm for ^1^H-NMR and 49.00 ppm for ^13^C-NMR) and deuterium oxide (4.79 ppm for ^1^H-NMR) were used as an internal reference [75]. NMR spectra in the Supplementary Information were produced utilising TopSpin 4.2.0 [76].

The boron hump is visible between ~10 and ~−40 ppm in the ^11^B-NMR spectra. This arises from borosilicate compounds contained in the NMR tubes and the NMR probe.

## 4. Conclusions

An NMR methodology for the study of borylated high Fsp^3^ index drug leads such as iminosugars and their precursors is presented. Concurrent mutarotation and borarotation equilibria are identified. The delineation of these drug’s equilibria in aqueous/protic environments advances the introduction of borylated carbohydrate derivatives in clinical applications (e.g., for BNCT and glycosidase modulation). Further studies in this area will include AI and molecular modelling to refine our understanding and modulation of these equilibria and of enzyme–drug interactions.

## Figures and Tables

**Table 1:** Structures of borylated intermediates, target compounds, and side-products.

**Intermediates**	** 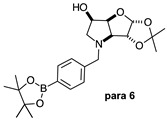 **	** 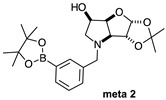 **	** 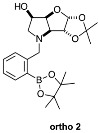 **
	** 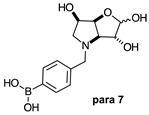 **	** 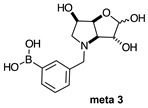 **	** 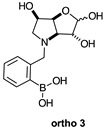 **
**Target** **Compounds**	** 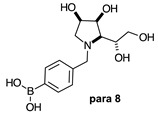 **	** 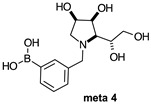 **	** 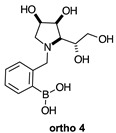 **
**Side-Products**		** 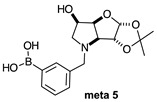 **	** 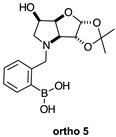 **

**Table 2:** ^11^B-NMR data (128 MHz) highlighting the borarotation process for all **para**, **meta**, and **ortho** species and the interplay between borarotation and mutarotation for intermediates **para 7**, **meta 3**, and **ortho 3**. The signal integration ratios are normalised to 1.0 for the partially tetrahedral/tetrahedral species. The most likely equilibria are shown for each chemical species. Chemical shifts in ppm.

Compound (Deuterated Solvent)	Chemical Shifts		Compound (Deuterated Solvent)	Chemical Shifts		Compound (Deuterated Solvent)	Chemical Shifts		
	Signal Integration Ratio			Signal Integration Ratio			Signal Integration Ratio		
	Signal Shape			Signal Shape			Signal Shape		
	Geometry			Geometry			Geometry		
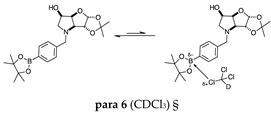	30.6	22.3	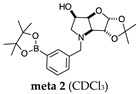	30.8		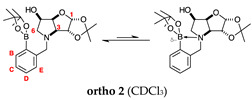	31.0	22.3	
	3.7	1.0		NA			6.4	1.0	
	broad	sharp		Broad			sharp	sharp	
	Boronate ester (trigonal planar)	Boronate ester (partially tetrahedral)		Boronate ester (trigonal planar)			Boronate ester (trigonal planar)	Boronate ammonium (partially tetrahedral)	
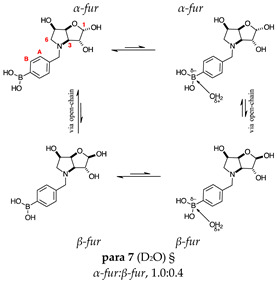	28.7	19.4	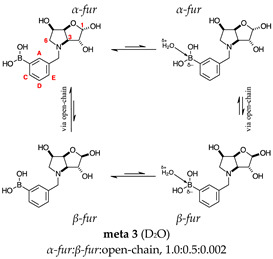	28.6	19.2	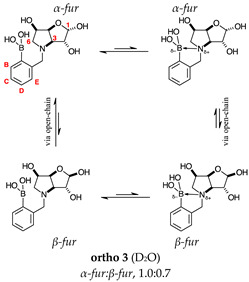	28.0	19.3	
	9.3	1.0		6.5	1.0		4.0	1.0	
	broad	sharp		broad	sharp		broad	sharp	
	Boronic acid (trigonal planar)	Boronate (partially tetrahedral)		Boronic acid (trigonal planar)	Boronate (partially tetrahedral)		Boronic acid (trigonal planar)	Boronate (partially tetrahedral)	
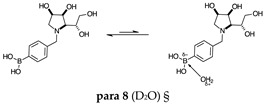	28.0	19.3	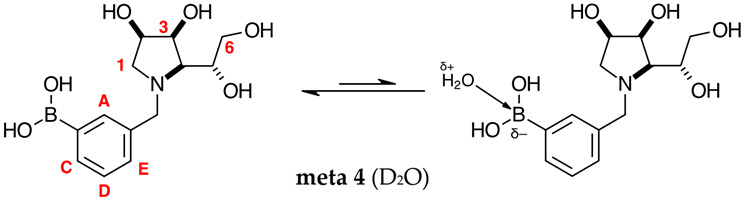	27.8	19.3	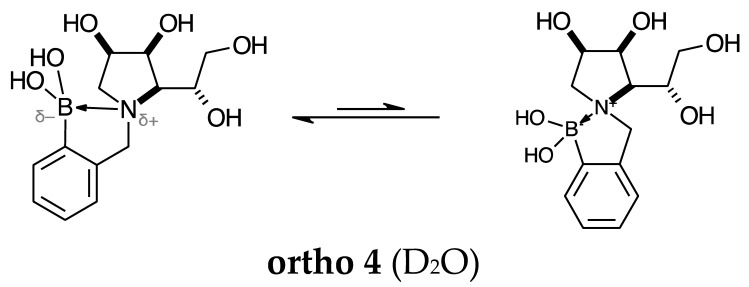	*28.3*	19.4	12.3, 11.0
	3.9	1.0		1.7	1.0		*1.1*	6.1	1.2, 1.0
	broad	sharp		broad	sharp		*broad*	sharp	Sharp and merging
	Boronic acid (trigonal planar)	Boronate (partially tetrahedral)		Boronic acid (trigonal planar)	Boronate (partially tetrahedral)		*Boronic acid* *(trigonal planar)*	Boronate ammonium (partially tetrahedral)	Boronate ammonium (tetrahedral)
			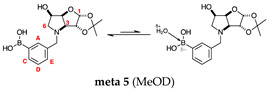	28.6	18.6	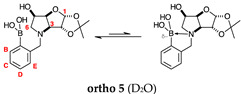	29.5	19.3	
				8.6	1.0		4.5	1.0	
				sharp	sharp		broad	sharp	
				Boronic acid (trigonal planar)	Boronate (partially tetrahedral)		Boronic acid (trigonal planar)	Boronate (partially tetrahedral)	

§ The following data will be shortly published.

**Table 3:** Comparison of ^1^H-NMR data for 1,4-dideoxy-1,4-imino-hexitols (see structures below) available from the chemical literature, including selected *N*-benzylated derivatives. The NMR spectra were acquired in D_2_O, unless stated otherwise. # Estimated assignment. On light blue background, relevant data for 1,4-dideoxy-1,4-imino-l-gulitol and its *N*-benzylated derivative. NA = not available, δ = chemical shifts (ppm), *J* = coupling constant (Hz).

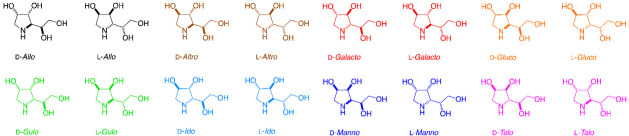								
**Compound:** **1,4-Dideoxy-1,4-imino-**	**^1^** **H-NMR Chemical Shifts (δ, ppm), Multiplicity, and Coupling Constants (*J*, Hz) for Nucleus:**							
	**H-1**	**H-1′**	**H-2**	**H-3**	**H-4**	**H-5**	**H-6**	**H-6′**
**d-Allitol.HCl** [42] #	δ 3.24, dd *J* 12.8, 1.6	δ 3.33, dd *J* 12.8, 3.6	δ 4.25, dd *J* 4.6, 2.4	δ 4.29, dd *J* 2.4, 4.4	δ 3.61, d *J* 4	δ 4.01, dd *J* 5, 3.6	δ 3.61, d *J* 4	δ 3.55, dd *J* 8, 3.2
[43]	δ 3.20, dd *J*_H-1,H-1′_ 12.6 *J*_H-1,H-2_ 2.1	δ 3.31, dd *J*_H-1′,H-1_ 12.6 *J*_H-1′,H-2_ 3.8	δ 4.21–4.26, m	δ 4.28, dd *J*_H-3,H-4_ 8.0 *J* 4.3	δ 3.50, dd *J*_H-4,H-3_ 8.0 *J* 3.6	δ 4.00–3.96, m	δ 3.64, dd *J*_H-6,H-6′_ 11.8 *J*_H-6,H-5_ 4.6	δ 3.60, dd *J*_H-6′,H-6_ 11.8 *J*_H-6′,H-5_ 6.5
[44]	δ 3.41, dd *J*_H-1,H-1′_ 12.8 *J*_H-1,H-2_ 2.1	δ 3.50, dd *J*_H-1′,H-1_ 12.8 *J*_H-1′,H-2_ 3.8	δ 4.41, dt	δ 4.47, dd *J*_H-3,H-2_ 4.2	δ 3.73, dd *J*_H-4,H-3_ 8.2 *J*_H-4,H-5_ 3.5	δ 4.18, m	δ 3.79, m, 2H	
[45] #	δ 3.30–3.50, m, 2H			δ 4.3–4.5, m, 2H	δ 3.60–3.80, m, 1H	δ 4.1–4.2, m, 1H		δ 3.60–3.80, m, 1H
[46]	δ 3.20, dd *J*_H-1,H-1′_ 12.8 *J*_H-1,H-2_ 2.0	δ 3.29, dd *J*_H-1′,H-1_ 12.8 *J*_H-1,H-2_ 3.7	δ 4.20, dt *J*_H-2,H-3_ 4.2	δ 4.26, dd	δ 3.51, dd *J*_H-4,H-3_ 8.2 *J*_H-4,H-5_ 3.5	δ 3.97, dt	δ 3.57, m, H-6, H-6′	
[47]	Spectra as for enantiomer as synthesised in [47]							
**l-Allitol.HCl** [48] #	δ 3.35, dd *J*_H-1,H-1′_ 12.9 *J*_H-1,H-2_ 2.1	δ 3.46, dd *J*_H-1′,H-1_ 12.9 *J*_H-1′,H-2_ 3.6	δ 4.33–4.40, m	δ 4.42, dd *J* 8.1, 4.2	δ 3.67, dd *J* 8.1, 3.3	NA	δ 3.72–3.79, m	
[47]	δ 3.33, dd *J*_H-1,H-1′_ 13 *J*_H-1,H-2_ 4.7	δ 3.41, dd *J* _H-1′,H-2_ 4	δ 4.33, ddd *J*_H-2,H-3_ 5	δ 4.38, dd	δ 3.64, dd *J*_H-4,H-3_ 8 *J*_H-4,H-5_ 3	δ 4.10, ddd *J*_H-5,H-6_ 6 *J*_H-5,H-6′_ 5	δ 3.69, d	δ 3.70, d
[44]	Spectra as for enantiomer							
**d-Altritol**	NA							
**l-Altritol**	NA							
**d-Galactitol.HCl** [49]	δ 3.25, dd *J* 12.4, 3.2	δ 3.49–3.43, m	δ 4.24, dt *J* 5.0, 3.2	δ 4.08, br t *J* 3.5	δ 3.49–3.43, m	δ 3.93–3.88, m	δ 3.69, dd *J* 12.2, 3.6	δ 3.58, dd *J* 12.2, 4.9
[50]	δ 3.52–3.37, m, 1H	δ 3.23, dd *J* 12.4, 2.6	δ 4.27–4.19, br t *J* 2.8	δ 4.06, br t *J* 3.5	δ 3.52–3.37, m, 1H	δ 3.98–3.82, m	δ 3.68, dd *J* 12.1, 3.7	δ 3.56, dd *J* 12.2, 4.9
[51] #	δ 3.42, dd *J* 12.0, 6.9	δ 3.56, dd *J* 12.0, 3.6	δ 3.91–3.94, m	δ 3.60–3.65, m	δ 2.82, br d *J* 6.0, 5.1	δ 3.98, dt *J* 5.1, 3.0	δ 2.97, dd *J* 12.6, 5.1	δ 2.77, dd *J* 12.6, 3.0
[52] (MeOD) #	δ 3.43, dd *J*_H-1,H-1’_ 11.9 *J*_H-1,H-2_ 4.6	δ 3.23, dd *J*_H-1’,H-1_ 11.9 *J*_H-1’,H-2_ 2.5	δ 4.18, ddd *J*_H-2,H-1’_ 4.5 *J*_H-2,H-1_ 2.6 *J*_H-2,H-3_ 2.6	δ 4.11, dd *J*_H-3,H-4_ 3.2 *J*_H-3,H-2_ 2.9	δ 3.47, dd *J*_H-4,H-5_ 7.0 *J*_H-4,H-3_ 3.6	δ 3.92, ddd *J*_H-5,H-4_ 7.1 *J*_H-5,H-6_ 4.3 *J*_H-5,H-6’_ 4.2	δ 3.72, dd *J*_H-6,H-6’_ 11.6 *J*_H-6,H-5_ 4.1	δ 3.65, dd *J*_H-6’,H-6_ 11.6 *J*_H-6’,H-5_ 4.4
[53]	δ 2.99, dd *J* 12.4, 4.9	δ 2.79, dd *J* 12.5, 2.8	δ 3.93–3.96, m	δ 4.01, dt	δ 2.85–2.83, m	δ 3.62–3.66, m	δ 3.58, dd *J* 11.8, 3.5 Hz	δ 3.45, dd, *J* 12.0, 6.6 Hz
[54] #	δ 3.35, dd *J* 12.7, 3.0	δ 3.60–3.53, m, 1H	δ 4.35, m	δ 4.18, t *J* 3.8	δ 4.01, m	δ 3.60–3.53, m, 1H	3.80, dd *J* 12.2, 3.6	δ 3.68, dd *J* 12.0, 5.0
[55]	Spectra as for enantiomer as synthesised in [55]							
**d-Galactitol** [56] #	δ 3.06, dd *J* 12.0, 5.6	δ 2.72, m	δ 4.05–3.99, m	δ 3.79, dd *J* 6.2, 3.9	δ 2.80, d *J* 8.4	δ 3.71–3.62, m	δ 3.51–3.45, m	δ 3.40–3.33, m
[52]	δ 3.00, dd *J*_H-1,H-1’_ 12.2 *J*_H-1,H-2_ 5.7	δ 2.78, dd *J*_H-1’,H-1_ 12.2 *J*_H-1’,H-2_ 3.9	δ 4.02, ddd *J*_H-2,H-1_ 5.7 *J*_H-2,H-1’_ 3.8 *J*_H-2,H-3_ 3.7	δ 3.82, dd *J*_H-3,H-4_ 6.0 *J*_H-3,H-2_ 3.7	δ 2.81, dd *J*_H-4,H-3_ 5.8 *J*_H-4,H-5_ 5.6	δ 3.73, ddd *J*_H-5,H-6’_ 7.4 *J*_H-5,H-4_ 5.1 *J*_H-5,H-6_ 4.0	δ 3.58, dd *J*_H-6,H-6’_ 11.8 *J*_H-6,H-5_ 3.9 Hz	δ 3.47, dd, *J*_H-6’,H-6_ 11.8 *J*_H-6’,H-5_ 7.4 Hz
[54] #	δ 2.87–2.81, m, 1H	δ 3.04, dd *J* 12.3, 5.1	δ 4.11, m	δ 4.04, m	δ 2.87–2.81, m, 1H	δ 3.77–3.70, m, 2H		δ 3.57, dd *J* 12.4, 7.9
*N*-Benzyl-1,4-dideoxy-1,4-imino-d-galactitol [57]	δ 2.86, m	δ 2.71, dd *J*_H-1’,H-1_ 10.7 *J*_H-1’,H-2_ 4.4	δ 3.95–3.84, m	δ 4.09, m	δ 2.91, dd *J*_H-4,H-5_ 4.6 *J*_H-4,H-3_ 2.7	δ 3.95–3.84, m	δ 3.72, dd *J*_H-6,H-6’_ 11.1 *J*_H-6,H-5_ 5.6	δ 3.68, dd *J*_H-6’,H-6_ 11.1 *J*_H-6,H-5_ 6.2
**l-Galactitol.HCl** [55]	δ 3.31, dd *J*_H-1,H-1’_ 13 *J*_H-1,H-2_ 3	δ 3.52, dd *J*_H-1’,H-2_ 5	δ 4.30, dt	δ 4.13, dd *J*_H-3,H-2_ 3	δ 3.51, dd *J*_H-4,H-3_ 4 *J*_H-4,H-5_ 7	δ 3.96, m	δ 3.64, dd *J*_H-6,H-6′_ 12 *J*_H-6,H-5_ 5 Hz	δ 3.75, dd, *J*_H-6′,H-5_ 4 Hz
**l-Galactitol** [CD_3_OD/D_2_O] [58]	δ 3.04, AB of ABX [∆δa-δb 72.0] *J*_H-1,H-1′_ 12.0 *J*_H-1′,H-2_ 4.4 *J*_H-1,H-2_ 2.4		δ 4.08–4.05, m	δ 4.10, ddd *J*_H-3,H-4_ 4.5 Hz *J*_H-3,H-2_ 4.5 Hz *J*_H-3,H-1_ 0.8 Hz	δ 3.02, t *J*_H-4,H-5/H-3_ 4.8	δ 3.80–3.76, m	δ 3.44, AB of ABX [∆δa-δb 22.8] *J*_H-6,H-6′_ 11.2 *J*_H-6,H-5_ 4.4 *J*_H-6′,H-5_ 6.4	
**d-Glucitol.HCl** [44]	δ 3.37, dd *J*_H-1,H-1′_ 13.1 *J*_H-1,H-2_ 0.7	δ 3.80, m, 1H	δ 4.45, m		δ 3.80, m, 1H	δ 4.18, dt *J* 8.8, 5.0	δ 3.80, m, 2H	
[46]	δ 3.14, d *J*_H-1,H-1′_ 13.1	δ 3.59, m, 1H	δ 4.21, m, 2H		δ 3.59, m, 1H	3.96, m	δ 3.59, m, 2H	
[CDCl_3_] [50]	δ 3.40, dd *J* 12.4, 2.7	δ 3.60, dd *J* 12.4, 4.5	δ 4.41–4.34, m		δ 3.63, dd *J* 10.1, 5.5	δ 4.09, ddd *J* = *J* = *J* 5.5	δ 3.75, dd *J* 11.9, 5.7	δ 3.79, dd *J* 11.9, 5.3
*N*-Benzyl-1,4-dideoxy-1,4-imino-d-glucitol.HCl [46]	δ 3.27, d *J*_H-1,H-1′_ 13.2	δ 3.71, dd *J*_H-1′,H-1_ 13.2 *J*_H-1′,H-2_ 4.2	δ 4.31, m	δ 4.16, d	δ 3.60, br s, 1H	δ 3.79, m	δ 3.60, br s, 2H	
**d-Glucitol** [46]	δ 2.69, d *J*_H-1,H-1′_ 12.8	δ 3.20, dd *J*_H-1’,H-1_ 12.8 *J*_H-1’,H-2_ 4.9	δ 4.03, m		δ 3.14, dd *J* 9.5, 3.6	δ 3.70, m	δ 3.43, dd *J*_H-6,H-6_ 12.0 *J*_H-6,H-5_ 6.5 Hz	δ 3.59, dd *J*_H-6’,H-6_ 12.0 *J*_H-6’,H-5_ 3.2
**l-Glucitol**	NA							
**d-Gulitol.HCl** [44]	δ 3.22, dd *J*_H-1,H-1’_ 12.0 *J*_H-1,H-2_ 8.4	δ 3.60, dd *J*_H-1’,H-1_ 12.0 *J*_H-1’,H-2_ 8.1	δ 4.55, dt	δ 4.34, t *J*_H-3,H-2=H-3,H-4_ 3.7	δ 3.69, m	δ 4.18, ddd *J*_H-5,H-4_ 8.6	δ 3.68, dd *J*_H-6,H-6’_ 12.2 *J*_H-6,H-5_ 5.1	δ 3.81, dd *J*_H-6’,H-6_ 12.2 *J*_H-6’,H-5_ 3.2
**l-Gulitol.HCl** [59]	δ 3.13	δ 3.52 *J*_H-1’,H-1_ 12	δ 4.47 *J*_H-2,H-1’_ 8 *J*_H-2,H-1_ 8 *J*_H-2,H-3_ 4	δ 4.25 *J*_H-3,H-4_ 4	δ 3.60	δ 4.10 *J*_H-5,H-4_ 9 *J*_H-5,H-6_ 5 *J*_H-5,H-6’_ 3	δ 3.60	δ 3.73 *J*_H-6’,H-6_ 12
[60]	δ 3.01, dd *J*_H-1,H-1’_ 12.0	δ 3.45, m, 1H	δ 4.35, dt *J*_H-2,H-1_ 8.3 *J*_H-2,H-3_ 4.0	δ 4.13, t	δ 3.45, m, 1H	δ 3.98, m	δ 3.62, dd *J*_H-6,H-6’_ 12.2	δ 3.45, m, 1H
§	δ 3.02, dd *J*_H-1,H-1’_ 11.5 *J*_H-1,H-2_ 8.4	δ 3.43, m, 1H	δ 4.35, dt *J*_H-2,H-1_ 8.1 *J*_H-2,H-3_ 3.9	δ 4.14, t *J* 3.6	δ 3.43, m, 1H	δ 3.98, m	δ 3.62, dd *J*_H-6,H-6′_ 12.2 *J*_H-6,H-5_ 3.1	δ 3.43, m, 1H
*N*-Benzyl-1,4-dideoxy-1,4-imino-l-gulitol[CDCl_3_] §	δ 3.28, dd *J*_H-1′,H-1_ 12.1 *J*_H-1′,H-2_ 9.1	δ 3.59, dd *J*_H-1,H-1′_ 12.0 *J*_H-1,H-2_ 7.0	δ 4.55, dddd *J*_H-2,H-1′_ 9.3 *J*_H-2,H-1/H-3_ 6.9 *J* 3.6	δ 4.42, ddd *J*_H-3,H-2_ 7.1 *J*_H-3,H-4_ 4.2 *J* 3.0	δ 3.84, dd *J*_H-4,H-5_ 9.3 *J*_H-4,H-3_ 4.0	δ 4.38, ddd *J*_H-5,H-4_ 8.5 *J*_H-5,H-6′_ 4.7 *J*_H-5,H-6_ 3.2	δ 3.85, dd *J*_H-6,H-6′_ 12.6 *J*_H-6,H-5_ 3.2	δ 3.70, dd *J*_H-6′,H-6_ 12.8 *J*_H-6′,H-5_ 4.8
**d-Iditol.HCl** [CDCl_3_] [49] #	δ 3.19, bd *J* 13.1	δ 3.58–3.55, m, 1H	δ 4.31, bd *J* 3.9	δ 4.20, bd *J* 2.5	δ 3.75–3.66, m, 1H	δ 4.04, ddd, *J* 8.5, 4.8, 3.3	δ 3.75–3.66, m, 1H	δ 3.58–3.55, m, 1H
[55]	δ 3.24, dd *J*_H-1′,H-1_ 13 *J*_H-1′,H-2_ 0	δ 3.62, dd *J*_H-1,H-2_ 4	δ 4.36, bd	δ 4.24, dd *J*_H-3,H-2_ 1	δ 3.77, dd *J*_H-4,H-5_ 9 *J*_H-4,H-3_ 3	δ 4.08, ddd	δ 3.74, dd *J*_H-6′,H-5_ 3	δ 3.62, dd *J*_H-6′,H-6_ 12 *J*_H-6′,H-5_ 5
**l-Iditol**	NA							
**d-Mannitol.HCl** #	δ 3.03, dd *J*_H-1,H-1′_ 11.9 *J*_H-1,H-2_ 8.9	δ 3.45, dd, 1H *J*_H-1′,H-1_ 11.8 *J*_H-1′,H-2_ 8.9	δ 4.35, dt *J*_H-2,H-1/H-1′_ 8.9 *J* 3.9	δ 4.23, t *J* 2.6	δ 3.45, 1H	δ 3.96, m	δ 3.55, m	
[61] #	δ 3.21, dd *J* 11.9, 8.6	δ 3.78–3.68, m, 1H	δ 4.14, dt *J* 8.5, 4.9	δ 3.78–3.68, m, 1H	δ 4.41, t *J* 3.4	δ 4.55–4.50, m	δ 3.66–3.60, m, 2H	
*N*-Benzyl-1,4-dideoxy-1,4-imino-d-mannitol.HCl [61] #	δ 3.89–3.78, m, 2H		δ 4.54–4.47, m		δ 3.89–3.78, m, 1H	δ 3.96, q *J* 5.0	δ 3.64, dd *J* 12.0, 7.2	δ 3.38, dd *J* 12.0, 7.2
**d-Mannitol** [62]	δ 2.58, dd	δ 2.97, dd *J*_H-1′,H-1_ 11.3	δ 4.13, dt *J*_H-2,H-1_ 8.1 *J*_H-2,H-3_ 5.0	δ 4.02, t *J*_H-3,H-4_ 5.0	δ 2.92, dd *J*_H-4,H-5_ 10.0	δ 3.66, ddd	δ 3.37, dd *J*_H-5,H-6_ 6.3	δ 3.56, dd *J*_H-6′,H-6_ 11.3 *J*_H-6′,H-5_ 3.8
[63] #	2.75, dd *J* 12, 7	3.15, dd *J* 11.5, 8	4.32, dt *J* 8, 4	4.20, t *J* 4	3.09, dd *J* 10, 4	3.85, ddd *J* 10, 7, 3.5	3.55, dd *J* 12, 7	3.75, dd *J* 12, 3.5
**l-Mannitol** [64]	δ 2.72, dd *J*_H-1,H-1′_ 11.2 *J*_H-1,H-2_ 3.5	δ 3.12, dd *J*_H-1′,H-1_ 11.2 *J*_H-1′,H-2_ 8.1	δ 4.29, dt *J*_H-1,H-1′_ 8.3 *J*_H-2,H-3_ 4.1	δ 4.16, app t *J* 3.9	δ 3.07, dd *J*_H-4,H-5_ 9.4 *J*_H-4,H-3_ 3.5	δ 3.81, ddd *J*_H-5,H-4_ 9.4 *J*_H-5,H-6′_ 6.4 *J*_H-5,H-6_ 2.8	δ 3.71, dd *J*_H-6,H-6′_ 12.0 *J*_H-6,H-5_ 2.8	δ 3.51, dd *J*_H-6’,H-6_ 12.0 *J*_H-6’,H-5_ 6.4
*N*-Benzyl-1,4-dideoxy-1,4-imino-l-mannitol [64]	δ 2.76, dd *J*_H-1,H-1’_ 11.4 *J*_H-1’,H-2_ 6.6	δ 2.83, dd *J*_H-1’,H-1_ 11.4 *J*_H-1’,H-2_ 6.6	δ 4.13, dt *J*_H-2,H-1_ 6.6 *J*_H-2,H-3_ 4.6	δ 4.34–4.29, m	δ 3.01–2.97, m	δ 3.93, dt *J*_H-5,H-6_ 6.3 *J*_H-5,H-6’_ 3.7	δ 3.79, dd *J*_H-6,H-6’_ 11.8 *J*_H-6,H-5_ 3.7	δ 3.72, dd *J*_H-6’,H-6_ 11.8 *J*_H-6’,H-5_ 6.3
**d-Talitol.HCl** [44]	δ 3.32, dd *J*_H-1,H-1’_ 13.0 *J*_H-1,H-2_ 1.7	δ 3.45, dd *J*_H-1’,H-1_ 13.0 *J*_H-1’,H-2_ 3.7	δ 4.33, dt	δ 4.24, dd *J*_H-3,H-2_ 3.9	δ 3.54, dd *J*_H-4,H-3_ 8.8 *J*_H-4,H-5_ 4.4	δ 3.98, m	δ 3.62, dd *J*_H-6,H-6′_ 12.1 *J*_H-6,H-5_ 5.0	δ 3.75, dd *J*_H-6′,H-6_ 12.1 *J*_H-6′,H-5_ 3.7
[45] #	δ 3.31, dd *J* 12.7, 1.3	δ 3.42, dd *J* 14, 3.8	δ 4.3, dt *J* 3.8, 1.3	δ 4.2, dd *J* 8.9, 3.8	δ 3.52, dd *J* 8.9, 3.8	δ 3.95, m	δ 3.6, dd *J* 11.4, 5.1	δ 3.73, dd *J* 11.4, 3.8
[65]	δ 3.19, dd *J*_H-1,H-1′_ 13.0 *J*_H-1,H-2_ 1.6	δ 3.30, dd *J*_H-1′,H-1_ 13.0 *J*_H-1′,H-2_ 3.8	δ 4.20, dt *J*_H-2,H-1′=H-2,H-3_ 4.0 *J*_H-2,H-1_ 1.6	δ 4.10, dd *J*_H-3,H-4_ 8.9 *J*_H-3,H-2_ 4.1	δ 3.41, dd *J*_H-4,H-3_ 8.9 *J*_H-4,H-5_ 4.3	δ 3.84, q	δ 3.50, dd *J*_H-6,H-6′_ 12.1 *J*_H-6,H-5_ 4.9	δ 3.61, dd *J*_H-6′,H-6_ 12.1 *J*_H-6′,H-5_ 3.7
[47]	δ 3.33, dd *J*_H-1,H-1′_ 13 *J*_H-1,H-2_ 2	δ 3.43, dd *J*_H-1′,H-2_ 4	δ 4.33, ddd	δ 4.23, dd *J*_H-3,H-2_ 4	δ 3.54, dd *J*_H-4,H-3_ 9 *J*_H-4,H-5_ 4	δ 3.97, dt	δ 3.63, dd *J*_H-6,H-6′_ 12 *J*_H-6,H-5_ 5	δ 3.74, dd *J*_H-6′,H-5_ 4
*N*-Benzyl-1,4-dideoxy-1,4-imino-d-talitol.HCl [65]	δ 3.20, dd *J*_H-1,H-1′_ 12.9 *J*_H-1,H-2_ 4.2	δ 3.28, dd *J*_H-1′,H-1_ 12.9 *J*_H-1′,H-2_ 3.9	δ 4.21, q	δ 4.11, dd *J*_H-3,H-4_ 6.3 *J*_H-3,H-2_ 4.2	δ 3.53, m	δ 3.80, m	δ 3.44, dd *J*_H-6,H-6′_ 12.3 *J*_H-6,H-5_ 4.9	δ 3.53, m
**d-Talitol** [65]	δ 2.62, dd *J*_H-1,H-1′_ 12.5 *J*_H-1,H-2_ 3.4	δ 3.02, dd *J*_H-1′,H-1_ 12.5 *J*_H-1’,H-2_ 5.1	δ 3.95, dt *J*_H-2,H-1’/H-3_ 5.2 *J*_H-2,H-1_ 3.4	δ 3.78, dd *J*_H-3,H-4_ 7.9 *J*_H-3,H-2_ 5.2	δ 2.78, dd *J*_H-4,H-3_ 7.9 *J*_H-4,H-5_ 4.2	δ 3.62, m	δ 3.40, dd *J*_H-6,H-6’_ 11.8 *J*_H-6,H-5_ 7.7	δ 3.51, dd *J*_H-6’,H-6_ 11.8 *J*_H-6’,H-5_ 4.1
[63] #	δ 2.80, dd *J*_H-1,H-1’_ 12 *J*_H-1,H-2_ 3.5	δ 3.21, dd *J*_H-1’,H-1_ 12 *J*_H-1’,H-2_ 5	δ 3.97, dd *J* 8, 5		δ 2.96, dd *J* 7.5, 4	δ 3.82, dd *J* 8, 4	δ 3.60, dd *J*_H-6,H-6’_ 12 *J*_H-6,H-5_ 8	δ 3.71, dd *J*_H-6’,H-6_ 12 *J*_H-6’,H-5_ 4
**l-Talitol.HCl** [47]	Spectra as for enantiomer as synthesised in [47]							

§ The following data will be shortly published.

**Table 4:** Comparison of ^13^C-NMR, melting point, and optical rotation data for 1,4-dideoxy-1,4-imino-hexitols available from the chemical literature, including selected *N*-benzylated derivatives. The NMR spectra were acquired in D_2_O, unless stated otherwise. # Estimated assignment. c = concentration (g/100 mL). On light blue background, relevant data for 1,4-dideoxy-1,4-imino-l-gulitol and its *N*-benzylated derivative. NA = not available.

Compound: 1,4-Dideoxy-1,4-imino-	^13^C-NMR Chemical Shifts (δ, ppm) (in D_2_O Unless Stated Otherwise) for Nucleus:						Melting Points (°C)	Optical Rotation	
	C-1	C-2	C-3	C-4	C-5	C-6			
								Temp (°C)	[α]_D_ (°)
**d-Allitol.HCl** [42] #	50.1	70.3	69.9	61.9	68.6	62.5	110–111 [42]	NA	+28.4 (c 0.6, H_2_O) [42]
[45]	50.9	71.1	70.8	62.9	69.4	63.3	109–110 [45]	25	+25.6 (c 0.9, H_2_O) [45]
[46]	50.9, t	71.1, d	70.9, d	62.8, d	69.4, d	63.3, t	110–111 [46]	20	+29.4 (c 0.53, H_2_O) [46]
[43]	NA						NA	25	+24.4 (c 1.0, H_2_O) [43]
[44]	NA						112–113 [44]	25	+25.6 (c 0.9, H_2_O) [44]
[66]	NA						NA	25	+25.0 (c 1, H_2_O) [66]
[47]	Spectra as for enantiomer as synthesised in [47]						110–111 [47]	20	+28 (c 4, H_2_O) [47]
*N*-Benzyl-1,4-dideoxy-1,4-imino-d-allitol.HCl [46] #	58.4, t	70.7, d	71.4, d	70.3, d	69.1, d	63.0, t	NA	20	+23.1 (c 0.72, H_2_O) [46]
**l-Allitol.HCl** [44]	Spectra as for enantiomer						110–112 [44]	NA	−24.0 (c 2.1, H_2_O) [44]
[48] #	50.0	70.3	70.0	61.8	68.6	62.4	110–112 [48]	20	−26.0 (c 1.0, H_2_O) [48]
[67]	NA						112–113 [67]	20	−24.6 (c 1.12, H_2_O) [67]
[47]	50.8	70.6	70.9	62.5	69.2	63.1	110–111 [47]	20	−28 (c 4, H_2_O) [47]
[46]	NA						110–111 [46]	25	−29.4 (c 0.53, H_2_O) [46]
**l-Allitol**	NA								
*N*-Benzyl-1,4-dideoxy-1,4-imino-l-allitol [67]							110–111 [67]	20	−25.5 (c 1.07, H**_2_**O) [67]
**d-Altritol**	NA								
**l-Altritol**	NA								
**d-Galactitol.HCl** [49] #	49.9	76.5	74.5	66.7	69.1	63.3	100–103 [49]	NA	−23 (c 1.5, H_2_O) [49]
[54] #	52.3	78.9	77.0	71.5	69.1	65.7	103–104 [54]	20	−20.4 (c 1.0, H_2_O) [54]
(MeOD) [52] #	51.5	78.1	76.1	70.3	69.2	65.0	102 [52]	22	−25.3 (c 1.0, MeOH) [52]
[50] #	49.6	76.2	74.2	66.4	68.7	62.9	100–102 [50]	NA	−22 (c 1.5, H_2_O) [50]
[57]	NA						98–101 [57]	NA	−24.1 (c 0.8, MeOH) [57]
[55]	Spectra as for enantiomer as synthesised in [55]						99–101 [55]	20	−23 (c 2, H_2_O) [55]
**d-Galactitol** [56] #	50.9	79.4	77.2	66.4	71.3	64.4	NA	25	+2.9 (c 1.0, H_2_O) [56]
[51] #	51.4	78.8	77.7	66.4	72.2	64.1	134–136 [51]	20	+3.0 (c 2.4, H_2_O) [51]
[52] #	51.3, t	77.8, d	79.7, d	66.1, d	71.8, d	64.3, t	NA	22	+3.0 (c 1.0, H**_2_**O) [52]
[53]	60.9	75.6	76.8	61.8	69.4	68.3	NA	20	+2.7 (c 1.8, H_2_O) [53]
[54] #	53.6	81.7	80.5	75.1	68.4	66.3	134–135 [54]	20	−1.4, (c 2.4, H_2_O) [54]
[68]	NA						NA	NA	−0.8 (c 2.0, H_2_O) [68]
*N*-Benzyl-1,4-dideoxy-1,4-imino-d-galactitol [57]	58.9	75.7	79.2	73.3	71.2	63.7	133–135 [57]	NA	−25.5 (c 1.0, CHCl_3_) [57]
**l-Galactitol.HCl** [55]	50.5	75.1	77.1	67.3	69.6	63.9	99–101 [55]	20	+24 (c 2, H_2_O) [55]
**l-Galactitol**[CD_3_OD/D_2_O] [58]	52.0	78.0	78.2	67.8	71.6	64.5	NA	25	−2.4 (c 3.8, H_2_O) [58]
**d-Glucitol.HCl**[CDCl_3_] [50] #	50.4	75.3	74.8	66.4	68.7	62.9	138–140 [50]	NA	−26 (c 2, H_2_O) [50]
[46] #	52.5, t	75.5, d	74.6, d	63.3, d	67.8, d	64.3, t	143–144 [46]	20	−28.1 (c 0.42, H_2_O) [46]
[44]	NA						142–144 [44]	NA	−25.0 (c 0.34, H**_2_**O) [44]
[69]	NA						140–142 [69]	20	−27 (H_2_O) [69]
*N*-Benzyl-1,4-dideoxy-1,4-imino-d-glucitol.HCl [46] #	59.9, t	74.8, d	76.9, d	70.0, d	68.8, d	63.6, t	NA	20	−31.9 (c 0.68, H**_2_**O) [46]
**d-Glucitol** [46] #	52.5, t	77.6, d	77.7, d	71.1, d	61.9, d	65.3, t	194–196 [46]	20	−10.1 (c 0.43, H_2_O) [46]
[68]	NA						200–203 [68]	20	−10.5 (c 1, H_2_O) [68]
[69]	NA						200–202 [69]	20	−11 (H_2_O) [69]
**l-Glucitol**	NA								
**d-Gulitol.HCl** [44]	46.3	70.1	69.5	62.7	67.8	62.7	180–182 [44]	NA	−4.9 (c 1.0, H_2_O) [44]
**l-Gulitol.HCl** [59]	47.2	71.2	70.5	63.8	69.0	63.8	182–183 [59]	20	+6.0 (c 4, H_2_O) [59]
§	47.6	71.5	70.9	64.2	69.3	64.2	168–170 §	NA	NA
[60]	47.5, t	69.1, d	70.7, d	64.0, d	71.3, d	64.0, t	170–173 [60]	20	+7.1 (c 0.48, H_2_O) [60]
*N*-Benzyl-1,4-dideoxy-1,4-imino-l-gulitol §	53.1	68.9	70.2	70.1	68.6	63.1	NA	25	−0.04 (c 0.08, MeOH) §
**d-Iditol.HCl**[CDCl_3_] [49] #	50.5	75.0	74.4	68.3	63.5	63.3	154–156 [49]	NA	+3.2 (c 1.5, H_2_O) [49]
[55]	51.1	75.6	75.0	68.8	64.1	63.8	157–158 [55] 161–162 [55]	20	+3.7 (c 3, H_2_O) [55]
**d-** **Iditol**	NA								
**l-Iditol.HCl** [55]	Spectra as for enantiomer as synthesised in [55]						152–155 [55] 157–158 [55]	20	−3.8 (c 3.1, H_2_O) [55]
**l-Iditol**	NA								
**d-Mannitol.HCl** [62] #	48.4, t	66.2, d	70.8, d	63.3, d	71.0, d	63.9, t	148–149 [62]	25	−16.3 (c 1, H_2_O) [62]
[61] #	47.2	67.1	69.8	62.2	69.9	62.8	147–148 [61]	29	−25.7 (c 0.94, MeOH) [61]
[70,71]	NA						149–151 [70]	25	−15.7 (c 1, H_2_O) [71]
[61] #	55.2	68.4	68.5	67.7	70.9	62.6	NA	26	−25.2 (c 0.27, MeOH) [61]
[72] #	48.4	68.4	71.0	64.1	71.2	63.4	146–148 §	NA	NA
[73]	NA						147–148 [73]	20	−15.8 (c 0.97, H_2_O) [73]
**d-Mannitol** [62]	48.5, t	70.3, d	71.4, d	60.7, d	72.1, d	63.6, t	137 [62]	20	−10.4 (c 0.12, H_2_O) [62]
[63]	NA						125–128 [63]	25	−12.4 (c 0.7, H_2_O) [63]
**l-Mannitol** [64]	48.8	71.8	72.5	61.0	70.7	64.0	NA	21	+10.3 (c 1.20, H_2_O) [64]
*N*-Benzyl-1,4-dideoxy-1,4-imino-l-mannitol [64]	55.8	70.0	72.8	66.3	71.2	63.7	108–109 [64]	21	+37.7 (c 1.20, H_2_O) [64]
**d-Talitol.HCl** [44]	49.7	71.9	69.0	61.7	67.6	63.2	152–154 [44]	NA	−50.5 (c 1.01, H_2_O) [44]
[45] #	49.9	72.2	67.8	61.9	69.3	63.1	150–152 [45]	32	−50.0 (c 0.5, H_2_O) [45]
[65] #	50.9, t	73.2, d	68.8, d	62.8, d	70.3, d	64.4, t	144–145 [65]	20	−56.3 (c 0.41, H_2_O) [65]
[47]	50.8	73.0	70.2	62.7	68.7	64.3	146–150 [47] 151–152 [47]	20	−54 (c 2, H_2_O) [47]
*N*-Benzyl-1,4-dideoxy-1,4-imino-d-talitol.HCl [65] #	56.0, t	73.4, d	70.6, d	NA	70.7, d	63.6, t	NA	20	−10.1 (c 0.94, H_2_O) [65]
**l-Talitol.HCl** [44]	Spectra as the enantiomer						148–152 [44]	NA	+46.7 (c 1.05, H**_2_**O) [44]
[47]	Spectra as for enantiomer as synthesised in [47]						151–152 [47]	20	+54 (c 2, H_2_O) [47]

§ The following data will be shortly published.

## Data Availability

Data of the compounds are available from the author.

## Supplementary Materials

The following supporting information can be downloaded at: https://www.mdpi.com/article/10.3390/molecules30071402/s1. Table S1. Structures of the reference compounds analysed by ^11^B-NMR and their corresponding signals at 128 MHz, dissolved in the deuterated solvent stated in brackets. Figure S1. ^1^H- (400 MHz), ^13^C-NMR (100 MHz), DEPT, ^11^B-NMR (128 MHz), COSY, HSQC and HMBC spectra of *N*-(3-methylphenyl boronic acid pinacol ester)-3,6-dideoxy-3,6-imino-1,2-*O*-isopropylidene-α-d-gulofuranose **meta 2** in CDCl_3_. Figure S2. ^1^H- (400 MHz), ^13^C-NMR (100 MHz), DEPT, ^11^B-NMR (128 MHz), COSY, HSQC and HMBC spectra of *N*-(3-methylphenyl boronic acid)-3,6-dideoxy-3,6-imino-d-gulofuranose **meta 3** in CDCl_3_. Figure S3. ^1^H-NMR spectrum (400 MHz, D_2_O) of intermediate **meta 3** with colour-coded signals, highlighting the furanose anomeric forms they belong to, with interpretation of the isolated signals and tentative interpretation of the overlapping ones. Namely, the orange designates the *α-fur* form and indigo designates the *β-fur* form. (A) section 7.87 ppm to 7.20 ppm; (B) section 5.50 ppm to 4.87 ppm; (C) section 4.61 ppm to 4.12 ppm; (D) section 4.00 ppm to 3.15 ppm. Highlighted are also the principal COSY correlations to hydrogen atoms within the same spin systems. Figure S4. ^13^C-NMR spectrum (100 MHz, D_2_O) sections of intermediate **meta 3** with colour-coded signals, highlighting the furanose anomeric forms they belong to, with interpretation of the isolated signals and tentative interpretation of the overlapping ones. Namely, the orange designates the *α-fur* form and indigo designates the *β-fur* form. (A) section 141.5 ppm to 127.2 ppm; (B) section 104.0 ppm to 76.0 ppm; (C) section 75.5 ppm to 56.7 ppm. Highlighted are also the principal HSQC and HMBC correlations to hydrogen atoms within the same spin systems. Figure S5. ^1^H- (400 MHz), ^13^C-NMR (100 MHz), DEPT, ^11^B-NMR (128 MHz), COSY and HSQC spectra of *N*-(3-methylphenyl boronic acid)-1,4-dideoxy-1,4-imino-l-gulitol **meta 4** in D_2_O. Figure S6. ^1^H- (400 MHz), ^13^C-NMR (100 MHz), DEPT, ^11^B-NMR (128 MHz), COSY, HSQC and HMBC spectra of *N*-(2-methylphenyl boronic acid pinacol ester)-3,6-dideoxy-3,6-imino-1,2-*O*-isopropylidene-α-d-gulofuranose **ortho 2** in CDCl_3._ Figure S7. ^1^H- (400 MHz), ^13^C-NMR (100 MHz), DEPT, ^11^B-NMR (128 MHz), COSY, HSQC and HMBC spectra of *N*-(2-methylphenyl boronic acid)-3,6-dideoxy-3,6-imino-d-gulofuranose **ortho 3** in D_2_O. Figure S8. ^1^H-NMR spectrum (400 MHz, D_2_O) of intermediate **ortho 3** with colour-coded signals, highlighting the furanose anomeric forms they belong to, with interpretation of the isolated signals and tentative interpretation of the overlapping ones. Namely, the orange designates the *α-fur* form and indigo designates the *β-fur* form. (A) section 7.93 ppm to 7.47 ppm; (B) section 5.57 ppm to 4.54 ppm; (C) section 4.54 ppm to 3.40 ppm. Highlighted are also the principal COSY correlations to hydrogen atoms within the same spin systems. Figure S9. ^13^C-NMR spectrum (100 MHz, D_2_O) sections of intermediate **ortho 3** with colour-coded signals, highlighting the furanose anomeric forms they belong to, with interpretation of the isolated signals and tentative interpretation of the overlapping ones. Namely, the orange designates the *α-fur* form and indigo designates the *β-fur* form. (A) section 100.5 ppm to 79 ppm; (B) section 105.0 ppm to 90.0 ppm; (C) section 78.0 ppm to 58.0 ppm. Highlighted are also the principal HSQC correlations to hydrogen atoms within the same spin systems. Figure S10. ^1^H- (400 MHz), ^13^C-NMR (100 MHz), DEPT, ^11^B-NMR (128 MHz), COSY, HSQC and HMBC spectra of *N*-(2-methylphenyl boronic acid)-1,4-dideoxy-1,4-imino-l-gulitol **ortho 4** in D_2_O. Figure S11. ^1^H-NMR spectrum (400 MHz, D_2_O) of final compound **ortho 4** with colour-coded signals, highlighting the boronic acid and boronate forms they belong to, with interpretation of the isolated signals and tentative interpretation of the overlapping ones. Namely, the orange designates the boronic acid form and indigo designates the boronate form. (A) section 8.00 ppm to 6.90 ppm; (B) section 4.65 ppm to 3.00 ppm; (C) section 1.80 ppm to 1.10 ppm. Highlighted are also the principal COSY correlations to hydrogen atoms within the same spin systems. Figure S12. ^13^C-NMR spectrum (100 MHz, D_2_O) sections of final compound **ortho 4** with colour-coded signals, highlighting the boronic acid and boronate forms they belong to, with interpretation of the isolated signals and tentative interpretation of the overlapping ones. Namely, the orange designates the boronic acid form and indigo designates the boronate form. (A) section 144 ppm to 109 ppm; (B) section 76 ppm to 44 ppm. Figure S13. ^1^H- (400 MHz), ^13^C-NMR (100 MHz), ^11^B-NMR (128 MHz), COSY and HSQC spectra of *N*-(3-methylphenyl boronic acid)-3,6-dideoxy-3,6-imino-1,2-*O*-isopropylidene-α-d-gulofuranose **meta 5** in MeOD. Figure S14. ^1^H-NMR spectrum (400 MHz, MeOD) of compound **meta 5** with colour-coded signals, highlighting the boronic acid form they belong to, with interpretation of the isolated signals and tentative interpretation of the overlapping ones. Namely, the orange designates the boronic acid form. (A) section 7.82 ppm to 7.00 ppm; (B) section 6.00 ppm to 4.00 ppm; (C) section 4.00 ppm to 2.50 ppm; (D) section 1.50 ppm to 1.00 ppm. Highlighted are also the principal COSY correlations to hydrogen atoms within the same spin systems. Figure S15. ^13^C-NMR spectrum (100 MHz, MeOD) of compound **meta 5** with colour-coded signals, highlighting the boronic acid form they belong to, with interpretation of the isolated signals and tentative interpretation of the overlapping ones. Namely, the orange designates the boronic acid form. (A) section 141 ppm to 25 ppm; (B) section 140 ppm to 120 ppm. Figure S16. ^1^H- (400 MHz) and COSY spectra of the minor species for *N*-(3-methylphenyl boronic acid)-3,6-dideoxy-3,6-imino-1,2-*O*-isopropylidene-α-d-gulofuranose **meta 5** in MeOD. Figure S17. ^1^H- (400 MHz), ^13^C-NMR (100 MHz), DEPT, HSQC and HMBC spectra of *N*-(3-methylphenyl boronic acid)-3,6-dideoxy-3,6-imino-1,2-*O*-isopropylidene-α-d-gulofuranose **meta 5** in MeOD. Figure S17. ^1^H- (400 MHz), ^13^C-NMR (100 MHz), DEPT, HSQC and HMBC spectra of *N*-(3-methylphenyl boronic acid)-3,6-dideoxy-3,6-imino-1,2-*O*-isopropylidene-α-d-gulofuranose **meta 5** in MeOD. Figure S18. ^1^H- (400 MHz), ^13^C-NMR (100 MHz), DEPT, ^11^B-NMR (128 MHz), COSY and HSQC spectra of *N*-(2-methylphenyl boronic acid)-3,6-dideoxy-3,6-imino-1,2-*O*-isopropylidene-α-d-gulofuranose **ortho 5** in D_2_O. Figure S19. ^1^H-NMR spectrum (400 MHz, D_2_O) of compound **ortho 5** with colour-coded signals, highlighting the boronic acid and boronate forms they belong to, with interpretation of the isolated signals and tentative interpretation of the overlapping ones. Namely, the orange designates the boronic acid form and indigo designates the boronate form. (A) section 7.80 ppm to 6.80 ppm; (B) section 6.10 ppm to 4.20 ppm; (C) section 3.90 ppm to 2.70 ppm; (D) section 1.60 ppm to 1.25 ppm. Highlighted are also the principal COSY correlations to hydrogen atoms within the same spin systems. Figure S20. ^13^C-NMR spectrum (100 MHz, D_2_O) of compound **ortho 5** with colour-coded signals, highlighting the boronic acid and boronate forms they belong to, with interpretation of the isolated signals and tentative interpretation of the overlapping ones. Namely, the orange designates the boronic acid form and indigo designates the boronate form. (A) section 140 ppm to 23 ppm; NMR Experimental details. References [1,2,3,4,36] are cited in the supplementary materials.
