# Supplementary material for: Borylated 5-Membered Ring Iminosugars: Detailed Nuclear Magnetic Resonance Spectroscopic Characterisation, and Method for Analysis of Anomeric and Boron Equilibria
Source: Molecules. 2025 Mar 21;30(7):1402. doi: 10.3390/molecules30071402 (PMC11990579; doi:10.3390/molecules30071402)
Supplement: Supplementary file 1 [file molecules-30-01402-s001.zip › molecules-3467788-supplementary-final_MS.pdf]

Supplementary Materials

Borylated 5-Membered Ring Iminosugars: Detailed Nuclear Magnetic Resonance Spectroscopic Characterisation, and Method for Analysis of Anomeric and Boron Equilibria

Michela Simone*[,1]

Index

Table S1. Structures of the reference compounds analysed by ^{11}B-NMR and their corresponding signals at 128 MHz, dissolved in the deuterated solvent stated in brackets. **4**

Figure S1. ^{1}H- (400 MHz), ^{13}C-NMR (100 MHz), DEPT, ^{11}B-NMR (128 MHz), COSY, HSQC and HMBC spectra of *N*-(3-methylphenyl boronic acid pinacol ester)-3,6-dideoxy-3,6-imino-1,2-*O*-isopropylidene-*α*-D-gulofuranose **meta 2** in $CDCl_3$. **6**

Figure S2. ^{1}H- (400 MHz), ^{13}C-NMR (100 MHz), DEPT, ^{11}B-NMR (128 MHz), COSY, HSQC and HMBC spectra of *N*-(3-methylphenyl boronic acid)-3,6-dideoxy-3,6-imino-D-gulofuranose **meta 3** in $CDCl_3$. **25**

Figure S3. ^{1}H-NMR spectrum (400 MHz, D_2O) of intermediate **meta 3** with colour-coded signals, highlighting the furanose anomeric forms they belong to, with interpretation of the isolated signals and tentative interpretation of the overlapping ones. Namely, the orange designates the *α-fur* form and indigo designates the *β-fur* form. A) section 7.87 ppm to 7.20 ppm; B) section 5.50 ppm to 4.87 ppm; C) section 4.61 ppm to 4.12 ppm; D) section 4.00 ppm to 3.15 ppm. Highlighted are also the principal COSY correlations to hydrogen atoms within the same spin systems. **57**

Figure S4. ^{13}C-NMR spectrum (100 MHz, D_2O) sections of intermediate **meta 3** with colour-coded signals, highlighting the furanose anomeric forms they belong to, with interpretation of the isolated signals and tentative interpretation of the overlapping ones. Namely, the orange designates the *α-fur* form and indigo designates the *β-fur* form. A) section 141.5 ppm to 127.2 ppm; B) section 104.0 ppm to 76.0 ppm; C) section 75.5 ppm to 56.7 ppm. Highlighted are also the principal HSQC and HMBC correlations to hydrogen atoms within the same spin systems. **61**

Figure S5. ^{1}H- (400 MHz), ^{13}C-NMR (100 MHz), DEPT, ^{11}B-NMR (128 MHz), COSY and HSQC spectra of *N*-(3-methylphenyl boronic acid)-1,4-dideoxy-1,4-imino-L-gulitol **meta 4** in D_2O. **64**

Figure S6. ^{1}H- (400 MHz), ^{13}C-NMR (100 MHz), DEPT, ^{11}B-NMR (128 MHz), COSY, HSQC and HMBC spectra of *N*-(2-methylphenyl boronic acid pinacol ester)-3,6-dideoxy-3,6-imino-1,2-*O*-isopropylidene-*α*-D-gulofuranose **ortho 2** in $CDCl_3$. **78**

Figure S7. ^{1}H- (400 MHz), ^{13}C-NMR (100 MHz), DEPT, ^{11}B-NMR (128 MHz), COSY, HSQC and HMBC spectra of *N*-(2-methylphenyl boronic acid)-3,6-dideoxy-3,6-imino-D-gulofuranose **ortho 3** in D_2O. **99**

Figure S8. ^{1}H-NMR spectrum (400 MHz, D_2O) of intermediate **ortho 3** with colour-coded signals, highlighting the furanose anomeric forms they belong to, with interpretation of the isolated signals and tentative interpretation of the overlapping ones. Namely, the orange designates the *α-fur* form and indigo designates the *β-fur* form. A) section 7.93 ppm to 7.47 ppm; B) section 5.57 ppm to 4.54 ppm; C) section 4.54 ppm to 3.40 ppm. Highlighted are also the principal COSY correlations to hydrogen atoms within the same spin systems. **122**

Figure S9. ^{13}C-NMR spectrum (100 MHz, D_2O) sections of intermediate **ortho 3** with colour-coded signals, highlighting the furanose anomeric forms they belong to, with interpretation of the isolated **125**

signals and tentative interpretation of the overlapping ones. Namely, the orange designates the *α-fur* form and indigo designates the *β-fur* form. A) section 100.5 ppm to 79 ppm; B) section 105.0 ppm to 90.0 ppm ; C) section 78.0 ppm to 58.0 ppm. Highlighted are also the principal HSQC correlations to hydrogen atoms within the same spin systems.

Figure S10. ^{1}H- (400 MHz), ^{13}C-NMR (100 MHz), DEPT, ^{11}B-NMR (128 MHz), COSY, HSQC and HMBC spectra of *N*-(2-methylphenyl boronic acid)-1,4-dideoxy-1,4-imino-L-gulitol **ortho 4** in D_2O. **127**

Figure S11. ^{1}H-NMR spectrum (400 MHz, D_2O) of final compound **ortho 4** with colour-coded signals, highlighting the boronic acid and boronate forms they belong to, with interpretation of the isolated signals and tentative interpretation of the overlapping ones. Namely, the orange designates the boronic acid form and indigo designates the boronate form. A) section 8.00 ppm to 6.90 ppm; B) section 4.65 ppm to 3.00 ppm; C) section 1.80 ppm to 1.10 ppm. Highlighted are also the principal COSY correlations to hydrogen atoms within the same spin systems. **154**

Figure S12. ^{13}C-NMR spectrum (100 MHz, D_2O) sections of final compound **ortho 4** with colour-coded signals, highlighting the boronic acid and boronate forms they belong to, with interpretation of the isolated signals and tentative interpretation of the overlapping ones. Namely, the orange designates the boronic acid form and indigo designates the boronate form. A) section 144 ppm to 109 ppm; B) section 76 ppm to 44 ppm. **157**

Figure S13. ^{1}H- (400 MHz), ^{13}C-NMR (100 MHz), ^{11}B-NMR (128 MHz), COSY and HSQC spectra of *N*-(3-methylphenyl boronic acid)-3,6-dideoxy-3,6-imino-1,2-*O*-isopropylidene-*α*-D-gulofuranose **meta 5** in MeOD. **159**

Figure S14. ^{1}H-NMR spectrum (400 MHz, MeOD) of compound **meta 5** with colour-coded signals, highlighting the boronic acid form they belong to, with interpretation of the isolated signals and tentative interpretation of the overlapping ones. Namely, the orange designates the boronic acid form. A) section 7.82 ppm to 7.00 ppm; B) section 6.00 ppm to 4.00 ppm; C) section 4.00 ppm to 2.50 ppm; D) section 1.50 ppm to 1.00 ppm. Highlighted are also the principal COSY correlations to hydrogen atoms within the same spin systems. **171**

Figure S15. ^{13}C-NMR spectrum (100 MHz, MeOD) of compound **meta 5** with colour-coded signals, highlighting the boronic acid form they belong to, with interpretation of the isolated signals and tentative interpretation of the overlapping ones. Namely, the orange designates the boronic acid form. A) section 141 ppm to 25 ppm; B) section 140 ppm to 120 ppm. **175**

Figure S16. ^{1}H- (400 MHz) and COSY spectra of the minor species for *N*-(3-methylphenyl boronic acid)-3,6-dideoxy-3,6-imino-1,2-*O*-isopropylidene-*α*-D-gulofuranose **meta 5** in MeOD. **177**

Figure S17. ^{1}H- (400 MHz), ^{13}C-NMR (100 MHz), DEPT, HSQC and HMBC spectra of *N*-(3-methylphenyl boronic acid)-3,6-dideoxy-3,6-imino-1,2-*O*-isopropylidene-*α*-D-gulofuranose **meta 5** in MeOD. **187**

Figure S18. ^{1}H- (400 MHz), ^{13}C-NMR (100 MHz), DEPT, ^{11}B-NMR (128 MHz), COSY and HSQC spectra of *N*-(2-methylphenyl boronic acid)-3,6-dideoxy-3,6-imino-1,2-*O*-isopropylidene-*α*-D-gulofuranose **ortho 5** in D_2O. **206**

Figure S19. ^{1}H-NMR spectrum (400 MHz, D_2O) of compound **ortho 5** with colour-coded signals, highlighting the boronic acid and boronate forms they belong to, with interpretation of the isolated signals and tentative interpretation of the overlapping ones. Namely, the orange designates the boronic acid form and indigo designates the boronate form. A) section 7.80 ppm to 6.80 ppm; B) section 6.10 ppm to 4.20 ppm; C) section 3.90 ppm to 2.70 ppm; D) section 1.60 ppm to 1.25 ppm. Highlighted are also the principal COSY correlations to hydrogen atoms within the same spin systems. **221**

Figure S20. ^{13}C-NMR spectrum (100 MHz, D_2O) of compound **ortho 5** with colour-coded signals, highlighting the boronic acid and boronate forms they belong to, with interpretation of the isolated signals and tentative interpretation of the overlapping ones. Namely, the orange designates the boronic acid form and indigo designates the boronate form. A) section 140 ppm to 23 ppm. **225**

NMR Experimental details **226**

References **259**

Table S1. Structures of the reference compounds analysed by ^{11}B-NMR and their corresponding signals at 128 MHz, dissolved in the deuterated solvent stated in brackets.

Structure	Chemical shift, ppm	Reference	Structure	Chemical shift, ppm	Ref
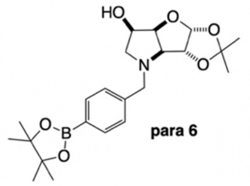 para 6	30.6, 22.3 (integration ratio 3.7 : 1.0) ($CDCl_3$)	§	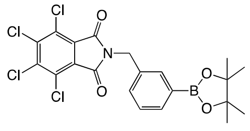 meta 13	31.5 ($CDCl_3$)	[1]
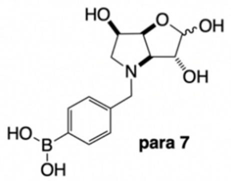 para 7	28.7, 19.4 (integration ratio 9.3 : 1.0) (D_2O)	§	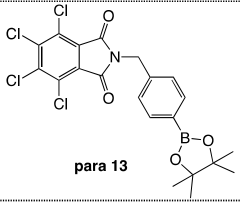 para 13	31.1 ($CDCl_3$)	[1]
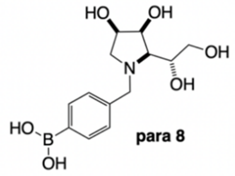 para 8	28.0, 19.3 (integration ratio 3.9 : 1.0) (D_2O)	§	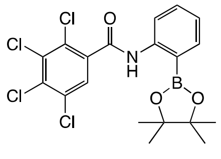 ortho 14	31.1 ($CDCl_3$)	[1]
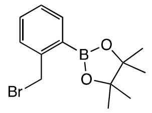 ortho 9	31 (D_2O)	Inventory (Boron Molecular)[2]	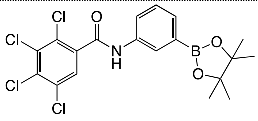 meta 14	31.7 ($CDCl_3$)	[1]
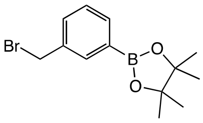 meta 9	31 (D_2O)	Inventory (Boron Molecular)[2]	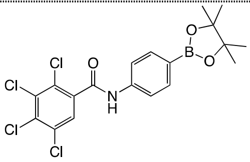 para 14	31.8 ($CDCl_3$)	[1]
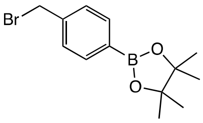 para 9	31 (D_2O)	Inventory (Boron Molecular)[2]	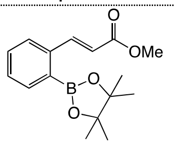 ortho 15	31.5 ($CDCl_3$)	[3]
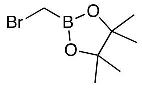 10	Rapidly decomposed (D_2O)	Inventory (Boron Molecular)[2]	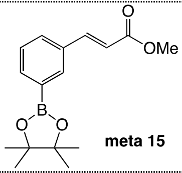 meta 15	30.7 ($CDCl_3$)	[3]
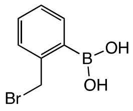 ortho 11	30 (D_2O)	Inventory (Boron Molecular)[2]	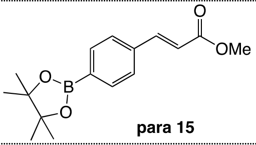 para 15	31.2 ($CDCl_3$)	[3]
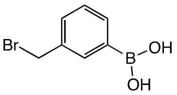 meta 11	29 (D_2O)	Inventory (Boron Molecular)[2]	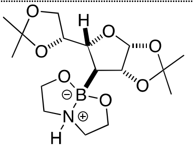 16 D-*Galacto*	30.9, 17.2, 10.3 (integration ratio: 0.02 : 0.03 : 1.0) (CD_3OD)	[4,5]
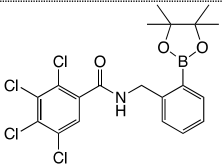 ortho 12	31.8 ($CDCl_3$)	[1]	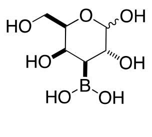 17 D-*Galacto*	31.7, 19.4 (integration ratio: 4.4 : 1.0) (D_2O)	[4,5]

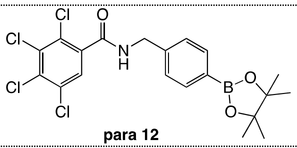 **para 12**	31.6 ($CDCl_3$)	[1]			

§ The following data will be shortly published.

Figure S1. ^{1}H- (400 MHz), ^{13}C-NMR (100 MHz), DEPT, ^{11}B-NMR (128 MHz), COSY, HSQC and HMBC spectra of *N*-(3-methylphenyl boronic acid pinacol ester)-3,6-dideoxy-3,6-imino-1,2-*O*-isopropylidene-*α*-D-gulofuranose **meta 2** in $CDCl_3$.

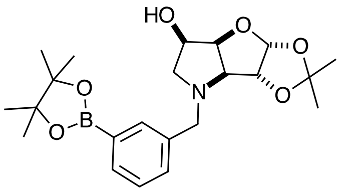

δH: 7.69 (1H, d, $J_{HC,HD}$ 7.2 Hz, ArH^{C}), 7.65 (1H, s, ArH^{A}), 7.39 (1H, d, $J_{HE,HD}$ 7.7 Hz, ArH^{E}), 7.31 (1H, app-t, $J_{HD,HC/HE}$ 7.4 Hz, ArH^{D}), 5.98 (1H, d, $J_{H-1,H-2}$ 3.4 Hz, H-1), 5.10-4.85 (1H, broad s, OH), 4.80 (1H, t, $J_{H-4,H-3/H-5}$ 5.7 Hz, H-4), 4.52 (1H, d, $J_{H-2,H-1}$ 3.3, H-2), 4.12 (1H, td, $J_{H-5,H-4}$ 5.7 Hz, $J_{H-5,H-6'}$ 2.1 Hz, H-5), 3.94 (1H, d, $J_{Ha,Hb}$ 13.2 Hz, ArCH^{a}H^{b}), 3.43 (1H, d, $J_{Hb,Ha}$ 13.1 Hz, ArCH^{a}H^{b}), 3.23 (1H, d, $J_{H-3,H-4}$ 5.5 Hz, H-3), 2.87 (1H, partially obscured dd, $J_{H-6',H-6}$ 11.0 Hz, $J_{H-6',H-5}$ 1.9 Hz, H-6′), 2.40 (1H, dd, $J_{H-6,H-6'}$ 10.8 Hz, $J_{H-6,H-5}$ 5.5 Hz, H-6), 1.49 (3H, s, CH_3 acetonide), 1.33 (12H, s, 4 x CH_3 pinacol), 1.32 (3H, s, CH_3 acetonide); **δC**: 137.0 (ArC$_{quat}$), 135.1 (ArC^{A}), 133.9 (ArC^{C}), 131.9 (ArC^{E}), 127.9 (ArC^{D}), 129.5-128.6 (broad, ArC^{B}-B), 112.6 (C$_{quat}$ acetonide), 107.6 (C-1), 84.0 (2 x C$_{quat}$ pinacol) 83.9 (C-2), 83.1 (C-4), 72.7 (C-3), 69.5 (C-5), 61.1 (C-6), 58.2 (CH_2Ar), 27.6, 26.9 (2 x CH_3, acetonide), 24.9 (4 x CH_3 pinacol); **δB**: 30.8.

^{1}H-NMR

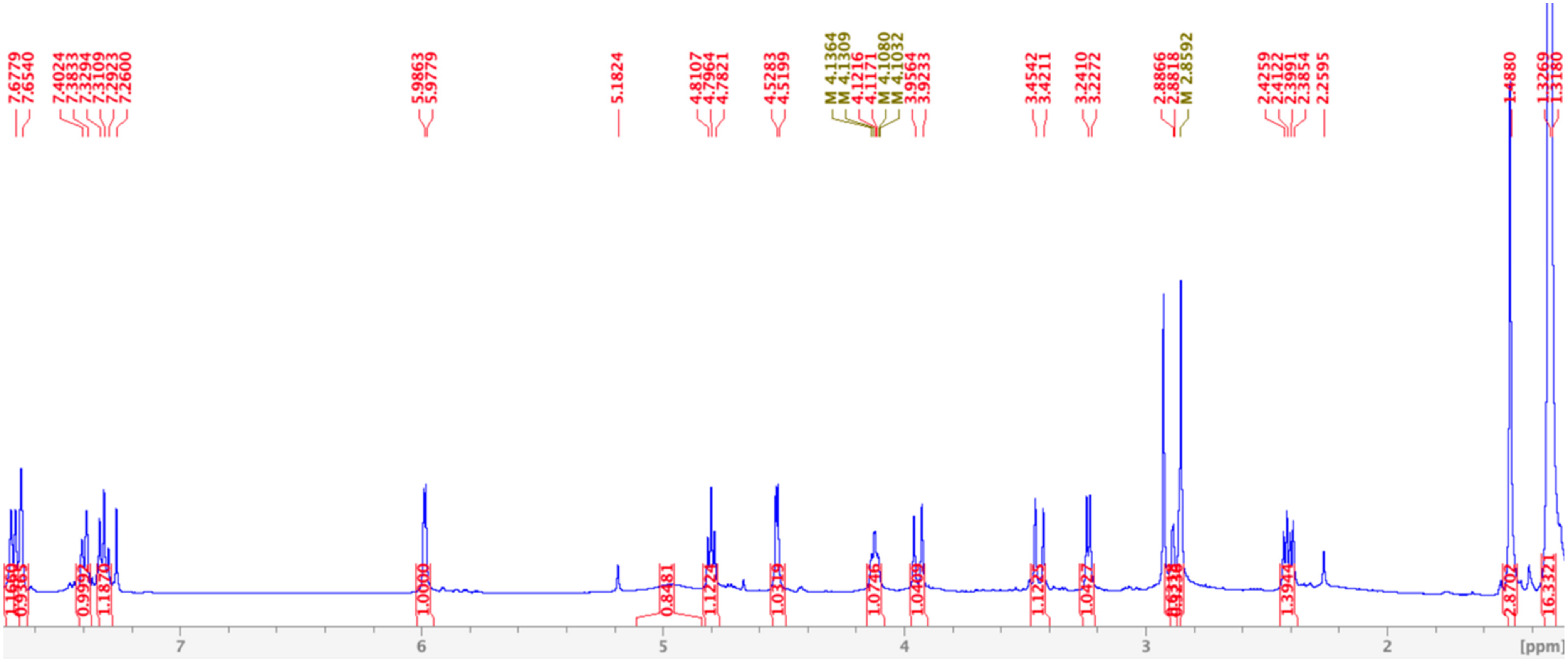
7.6779
7.6540
7.4024
7.3833
7.3294
7.3109
7.2923
7.2600
5.9863
5.9779
5.1824
4.8107
4.7964
4.7821
4.5283
4.5199
M 4.1364
M 4.1309
4.1216
4.1171
M 4.1080
M 4.1032
3.9564
3.9233
3.4542
3.4211
3.2410
3.2272
2.8866
2.8818
M 2.8592
2.4259
2.4122
2.3991
2.3854
2.2595
1.4880
1.3269
1.3180
0.9992
1.1870
1.0000
0.8481
1.1224
1.0319
1.0746
1.0409
1.1225
1.0427
1.3944
2.8702
16.3321
7
6
5
4
3
2
[ppm]

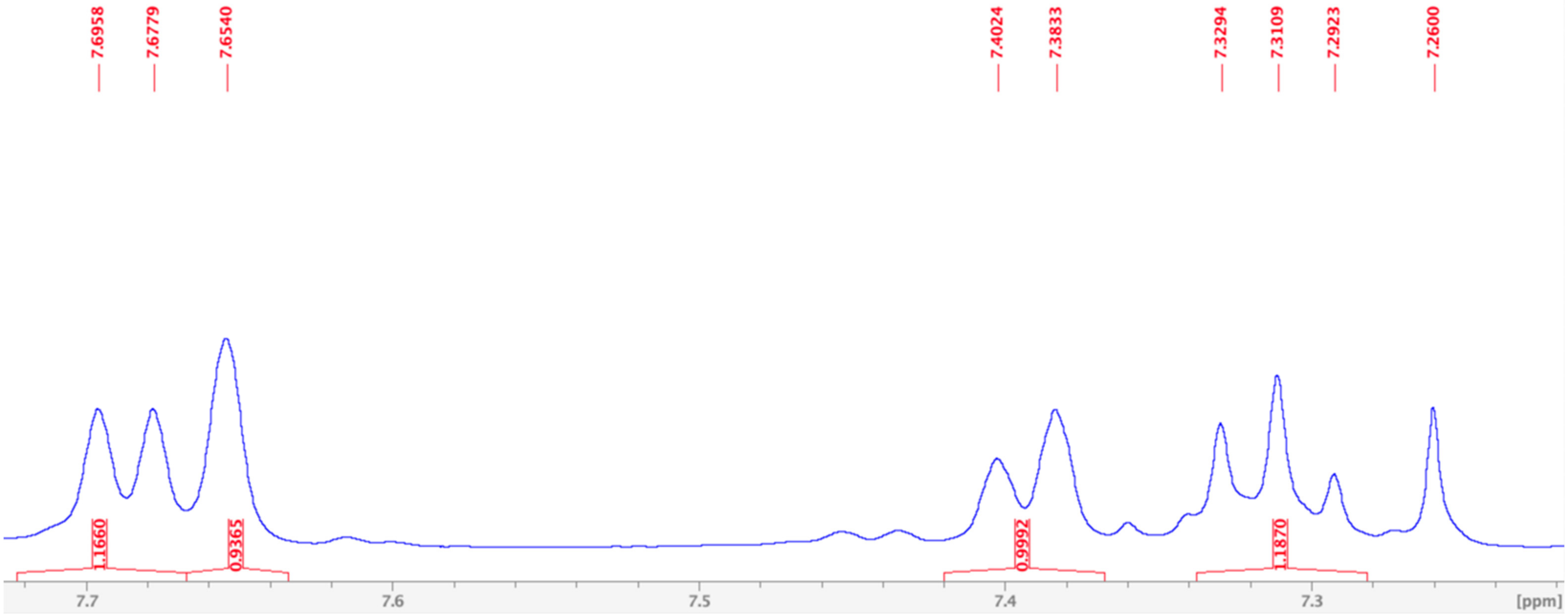
7.6958
7.6779
7.6540
7.4024
7.3833
7.3294
7.3109
7.2923
7.2600
1.1660
0.9365
0.9992
1.1870
7.7
7.6
7.5
7.4
7.3
[ppm]

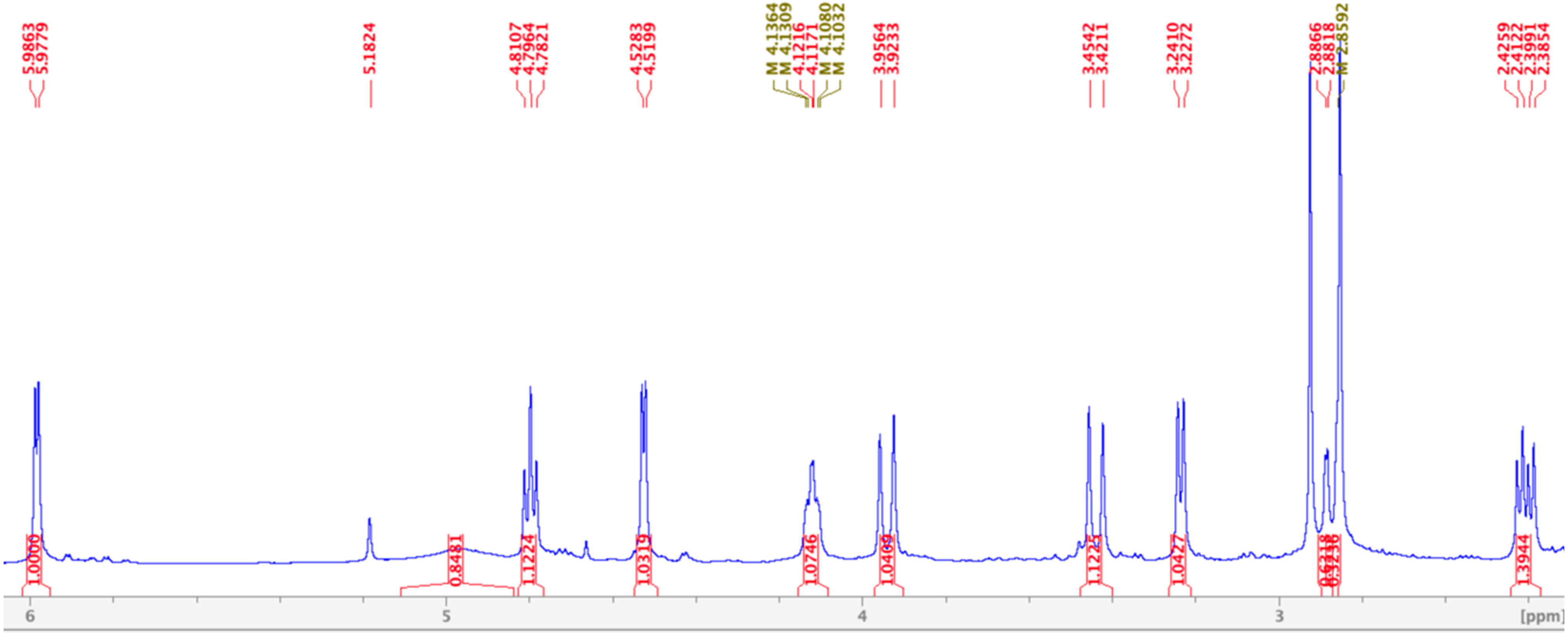
5.9863
5.9779
5.1824
4.8107
4.7964
4.7821
4.5283
4.5199
M 4.1364
M 4.1309
4.1216
4.1171
M 4.1080
M 4.1032
3.9564
3.9233
3.4542
3.4211
3.2410
3.2272
2.8866
2.8818
M 2.8592
2.4259
2.4122
2.3991
2.3854
1.0000
0.8481
1.1224
1.0319
1.0746
1.0469
1.1225
1.0427
1.3944
6
5
4
3
[ppm]

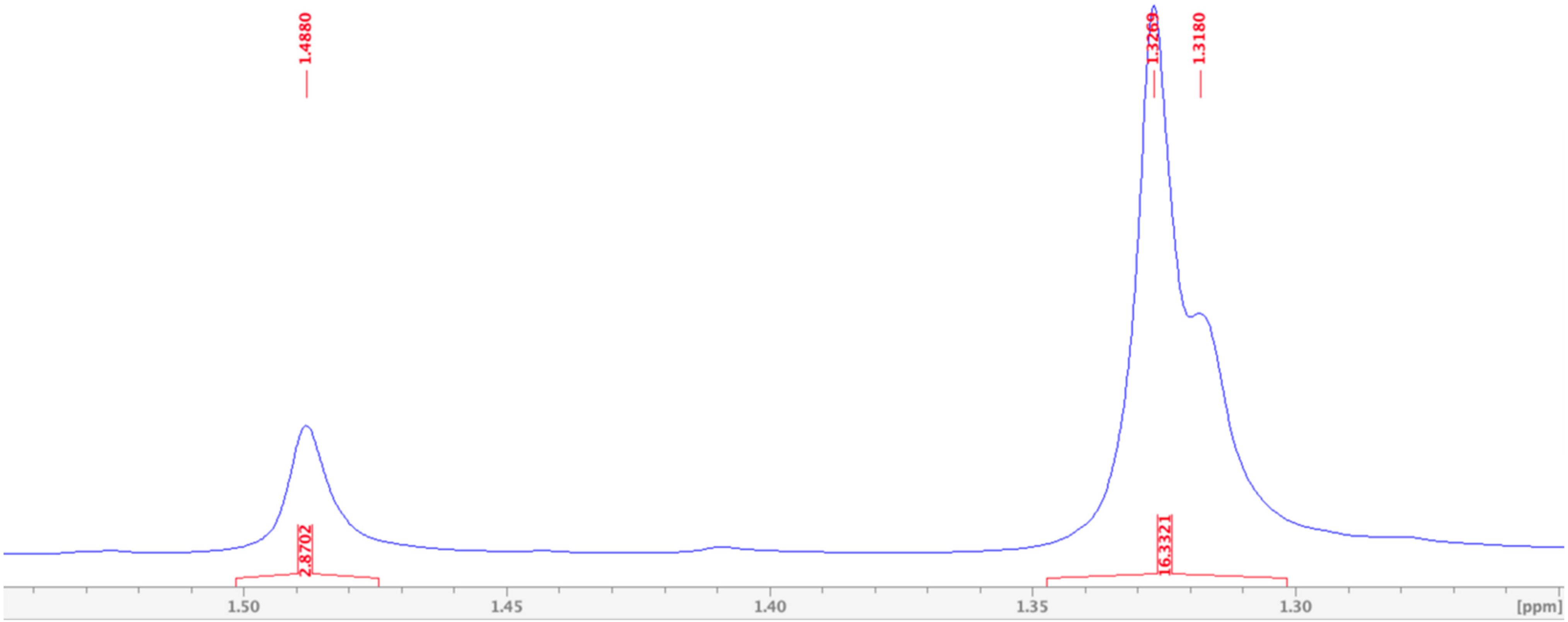

1.4880
1.3269
1.3180
2.8702
16.3321
1.50
1.45
1.40
1.35
1.30
[ppm]

^{13}C-NMR

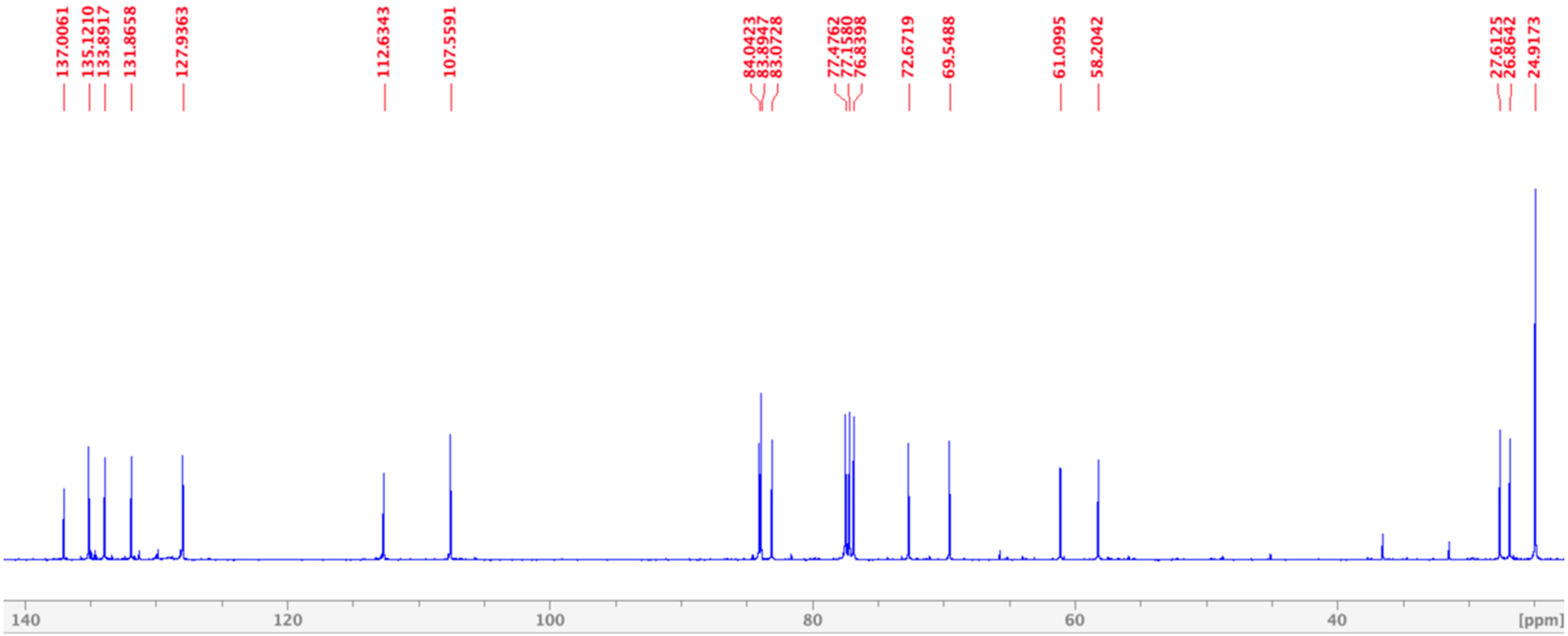
137.0061
135.1210
133.8917
131.8658
127.9363
112.6343
107.5591
84.0423
83.8947
83.0728
77.4762
77.1580
76.8398
72.6719
69.5488
61.0995
58.2042
27.6125
26.8642
24.9173
140
120
100
80
60
40
[ppm]

DEPT

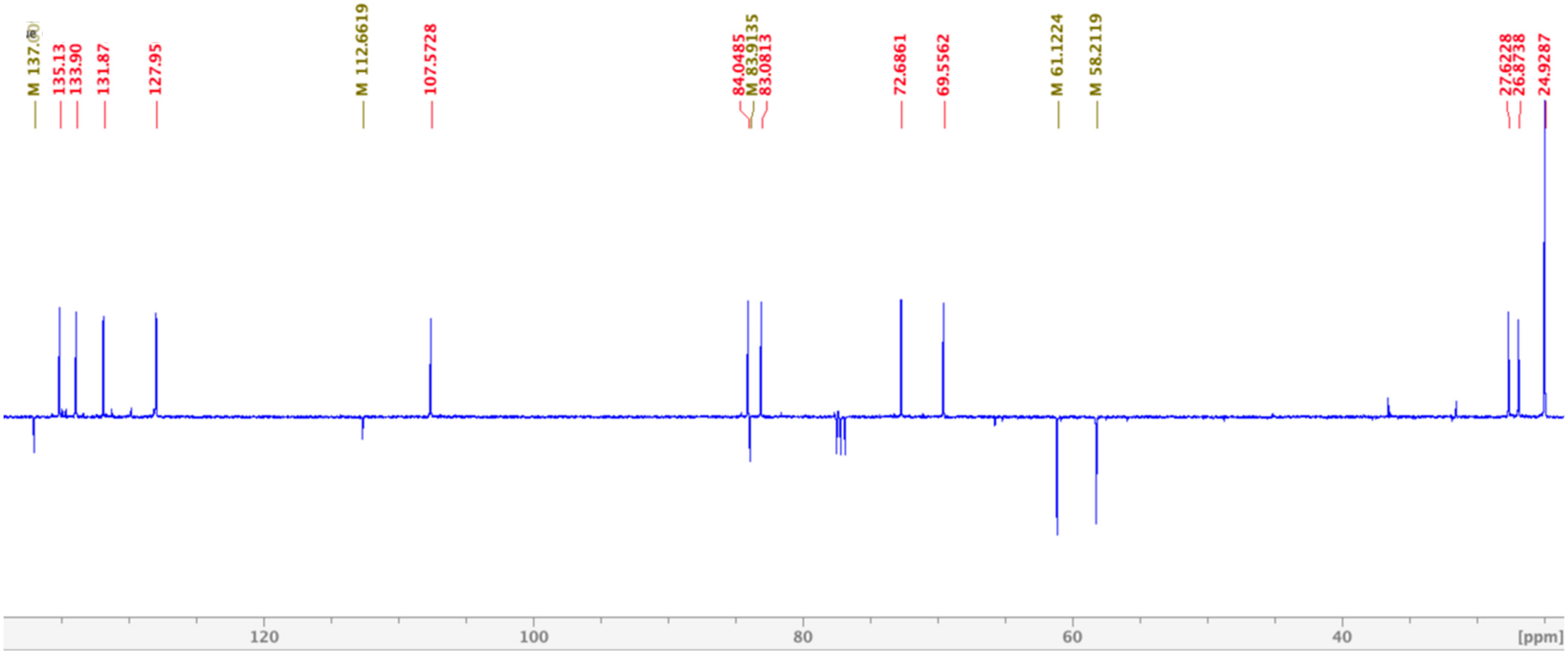
M 137.[illegible]
135.13
133.90
131.87
127.95
M 112.6619
107.5728
84.0485
M 83.9135
83.0813
72.6861
69.5562
M 61.1224
M 58.2119
27.6228
26.8738
24.9287
120
100
80
60
40
[ppm]

¹¹B-NMR

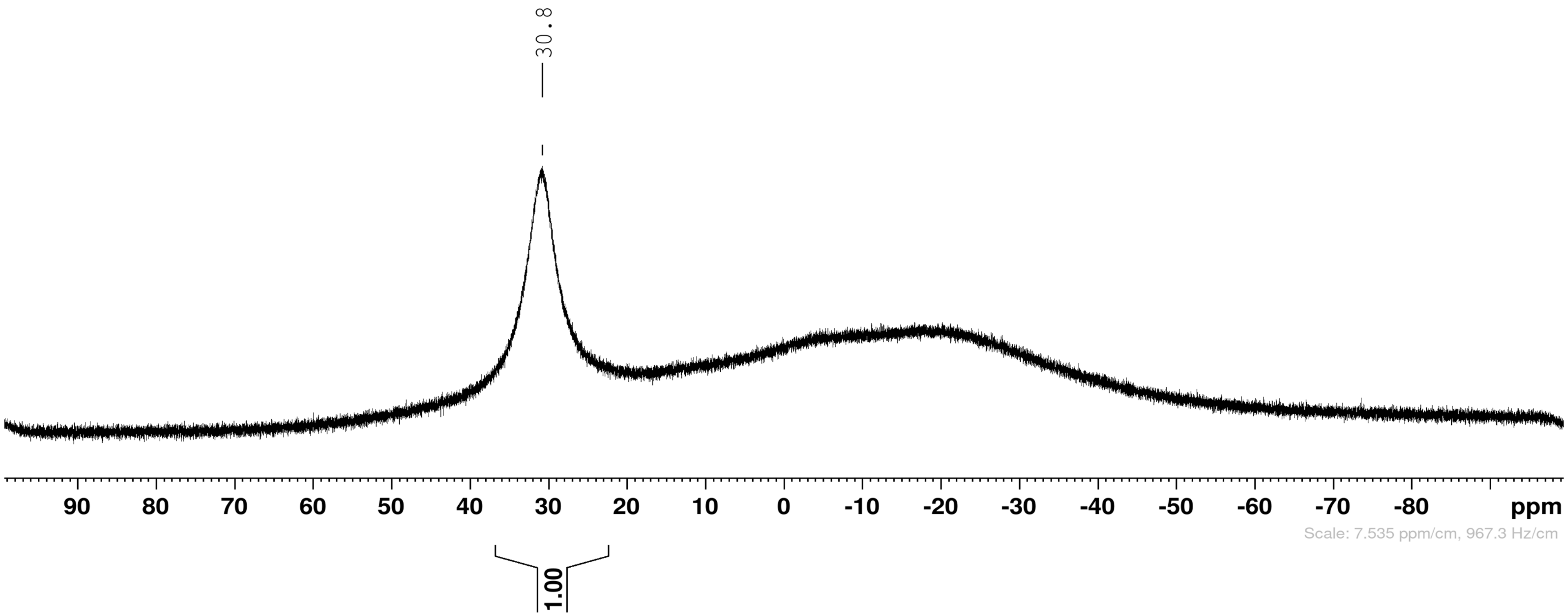

30.8
90
80
70
60
50
40
30
20
10
0
-10
-20
-30
-40
-50
-60
-70
-80
ppm
Scale: 7.535 ppm/cm, 967.3 Hz/cm
1.00

COSY

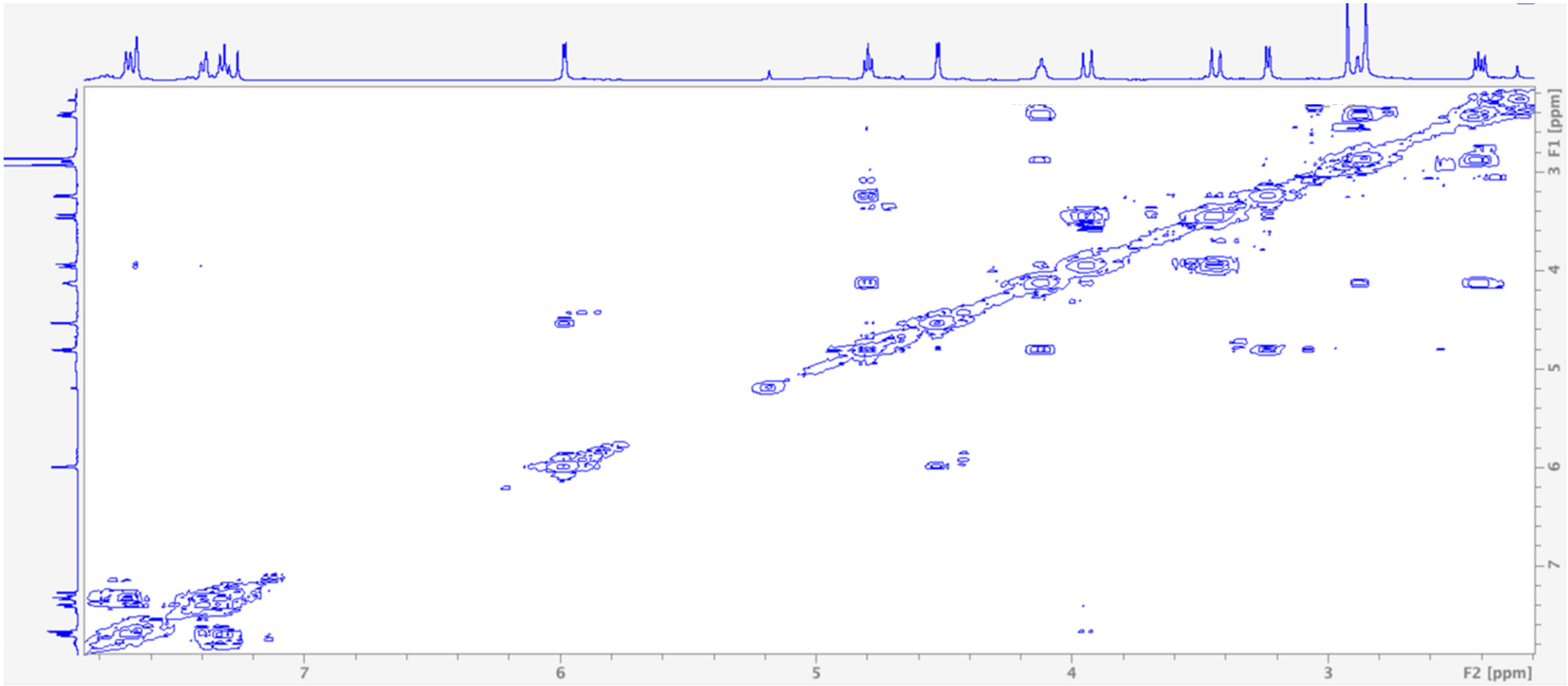

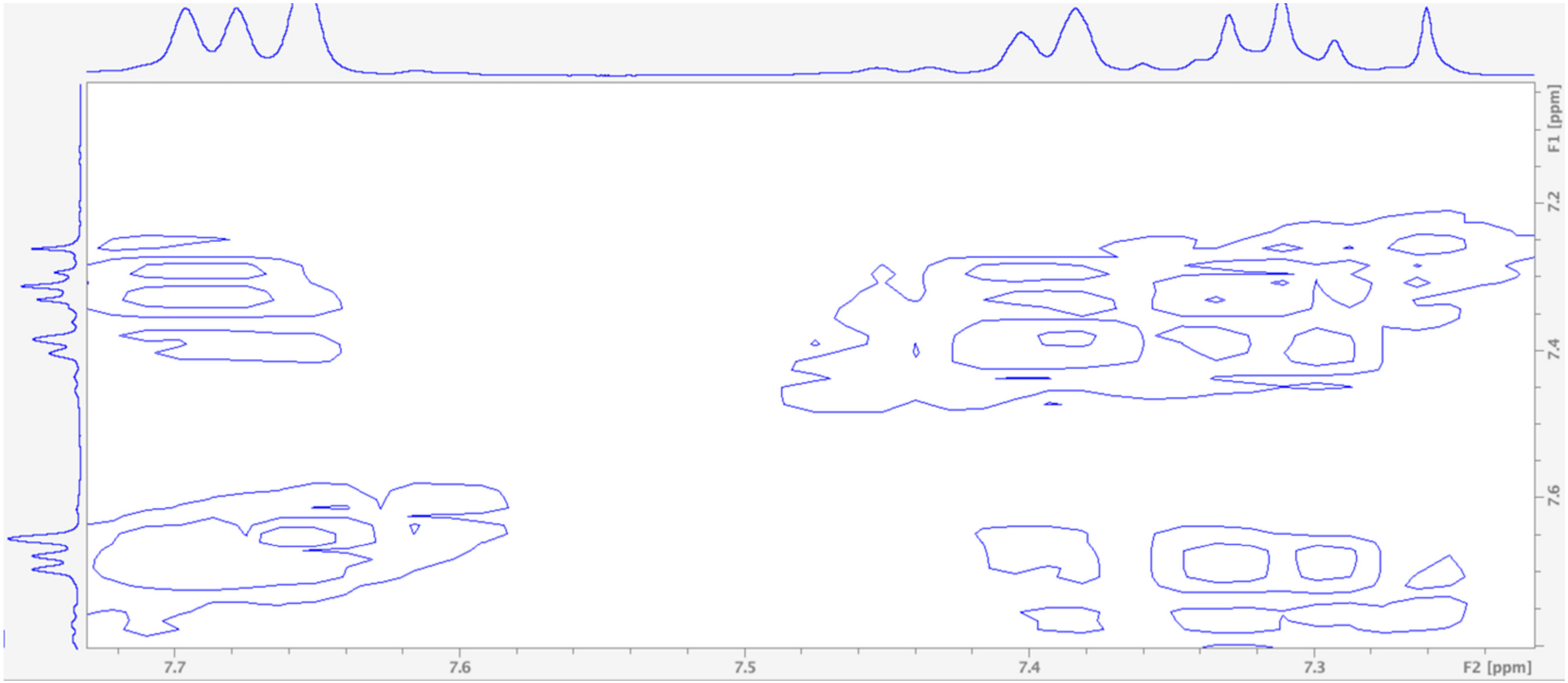
F1 [ppm]
7.2
7.4
7.6
7.7
7.6
7.5
7.4
7.3
F2 [ppm]

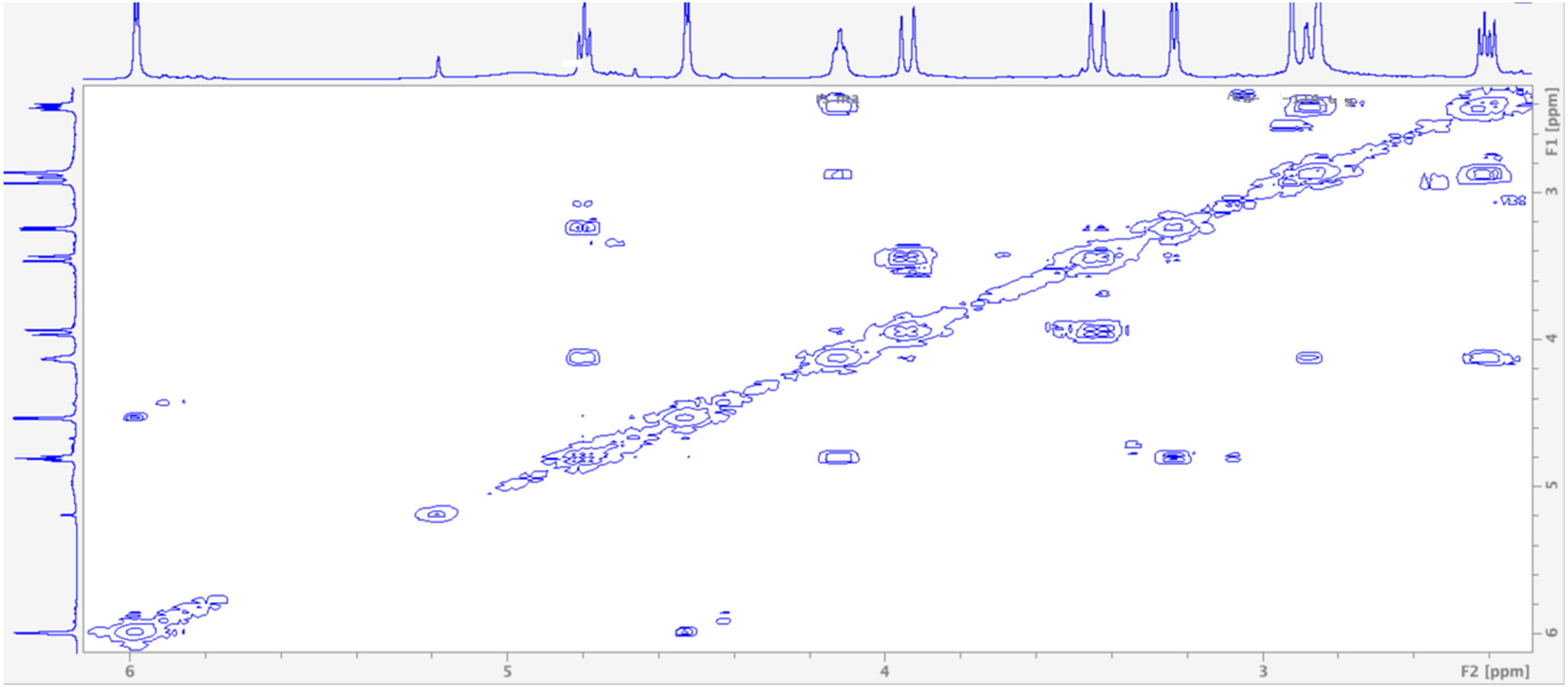

F1 [ppm]
3
4
5
6
6
5
4
3
F2 [ppm]

HSQC

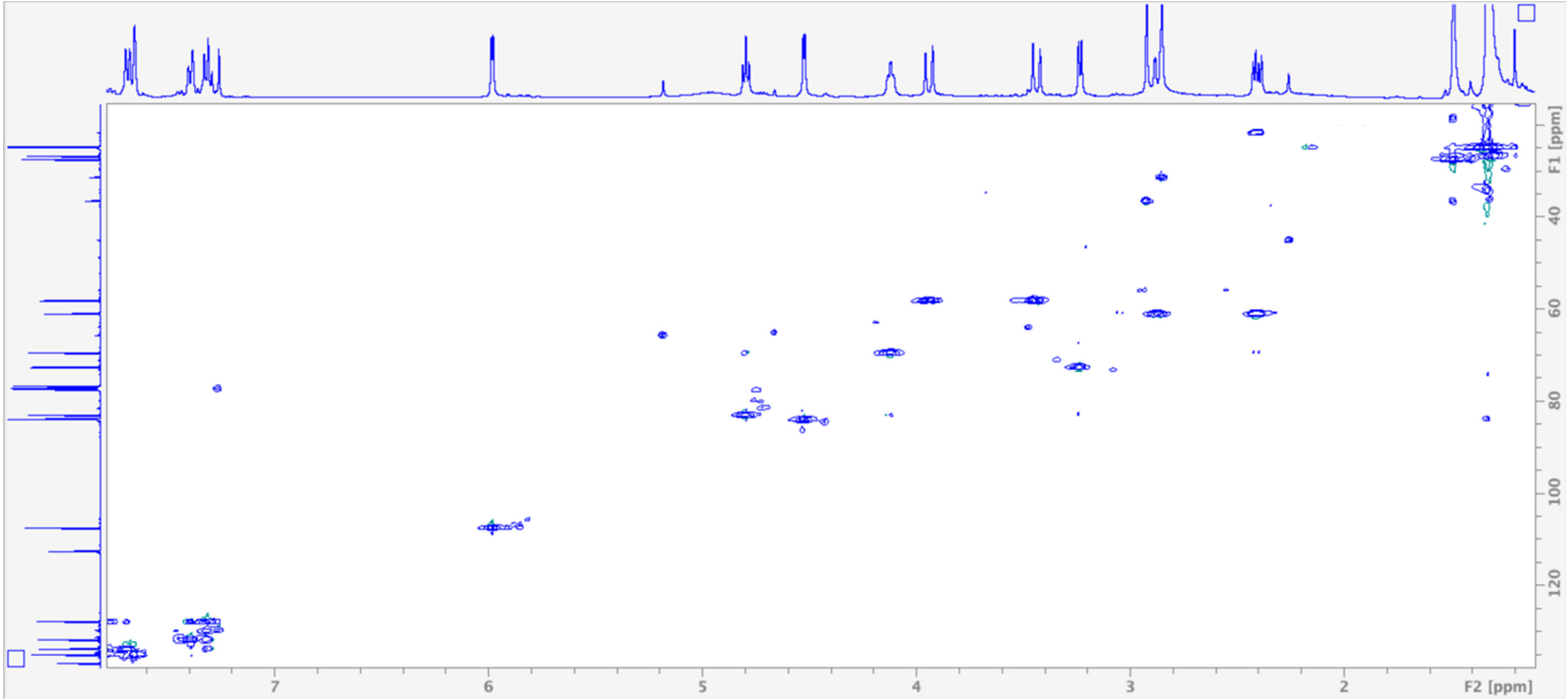

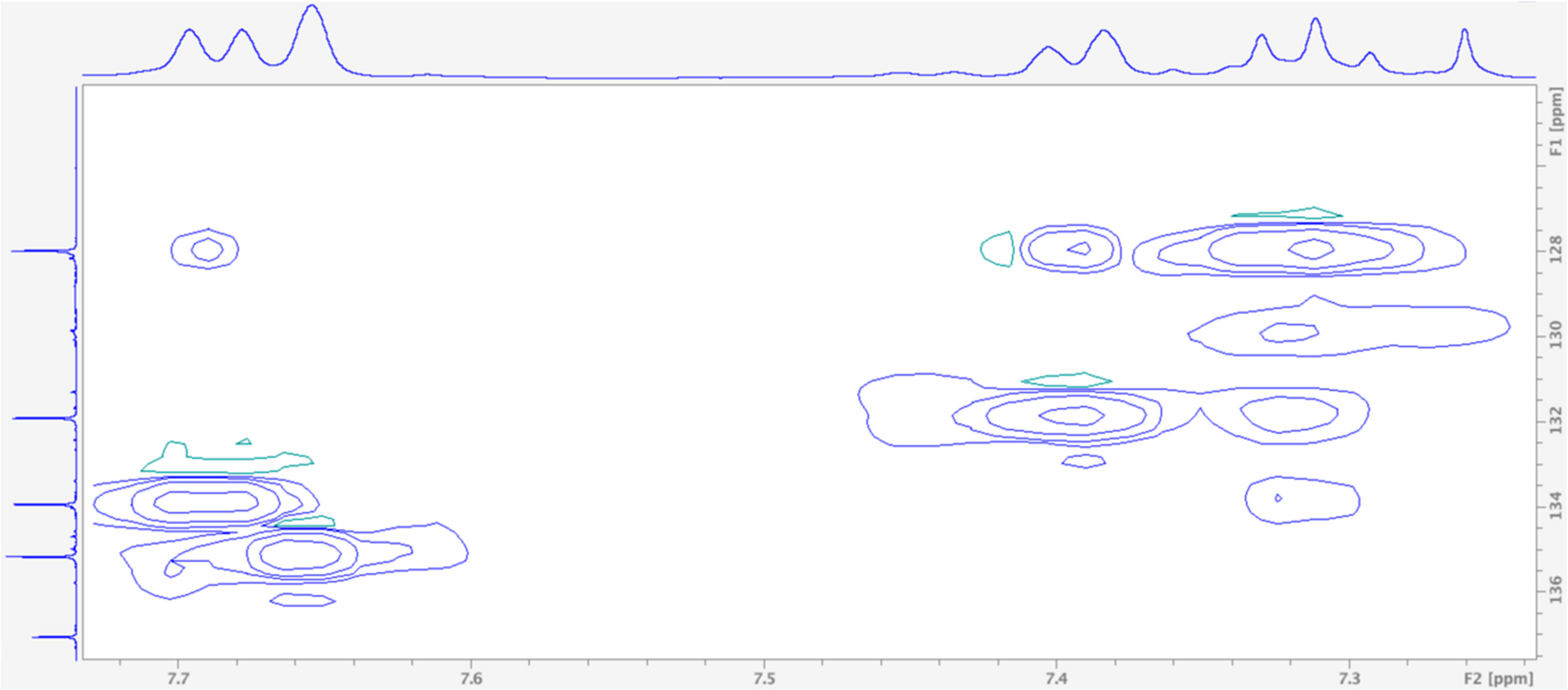

F1 [ppm]
128
130
132
134
136
7.7
7.6
7.5
7.4
7.3
F2 [ppm]

HMBC

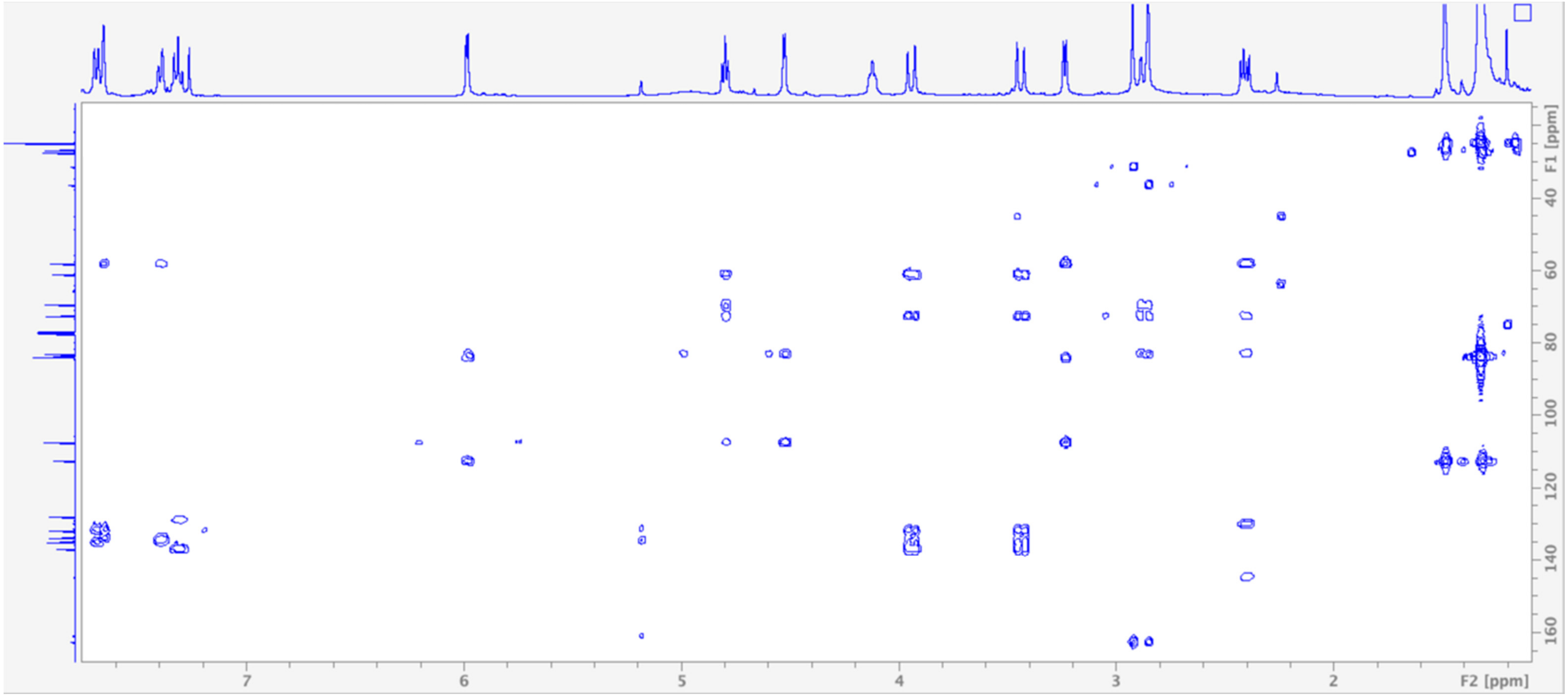

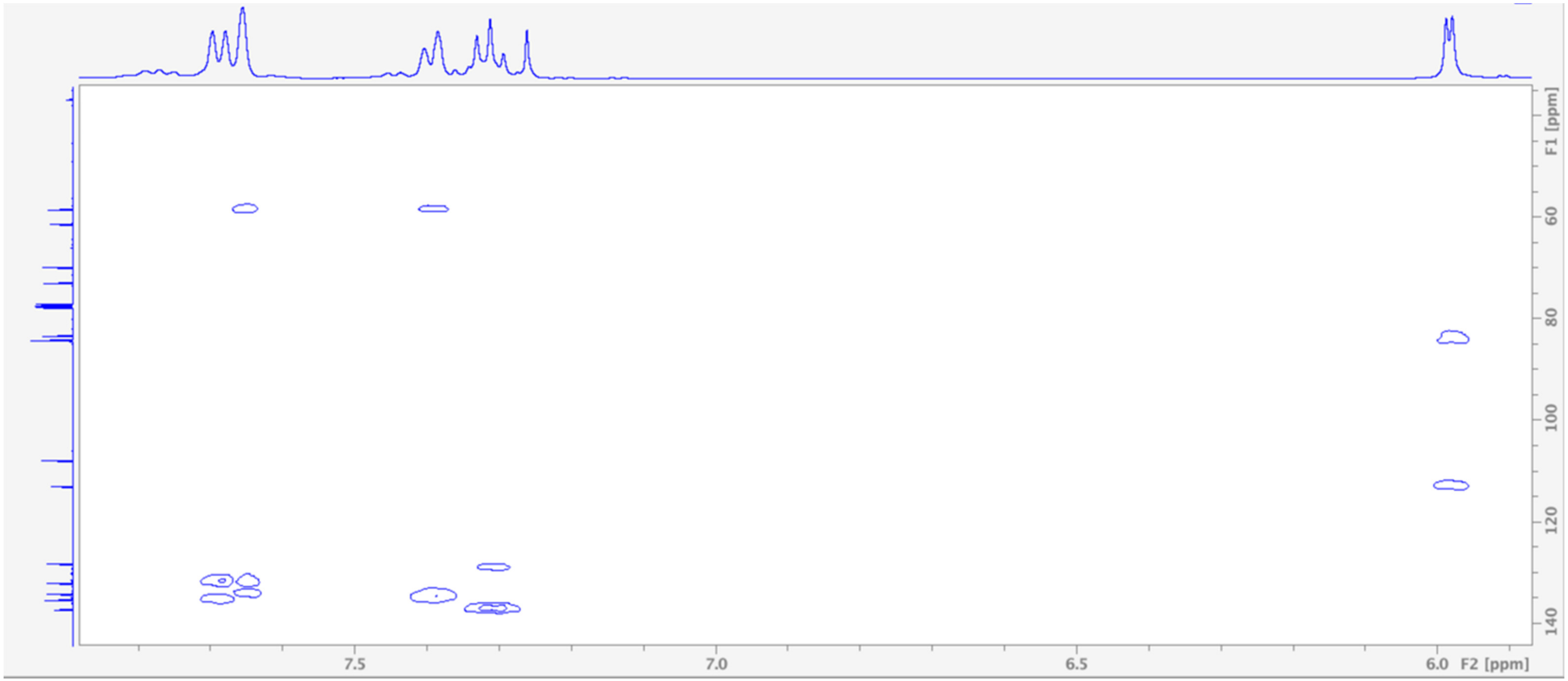

60
80
100
120
140
F1 [ppm]
7.5
7.0
6.5
6.0
F2 [ppm]

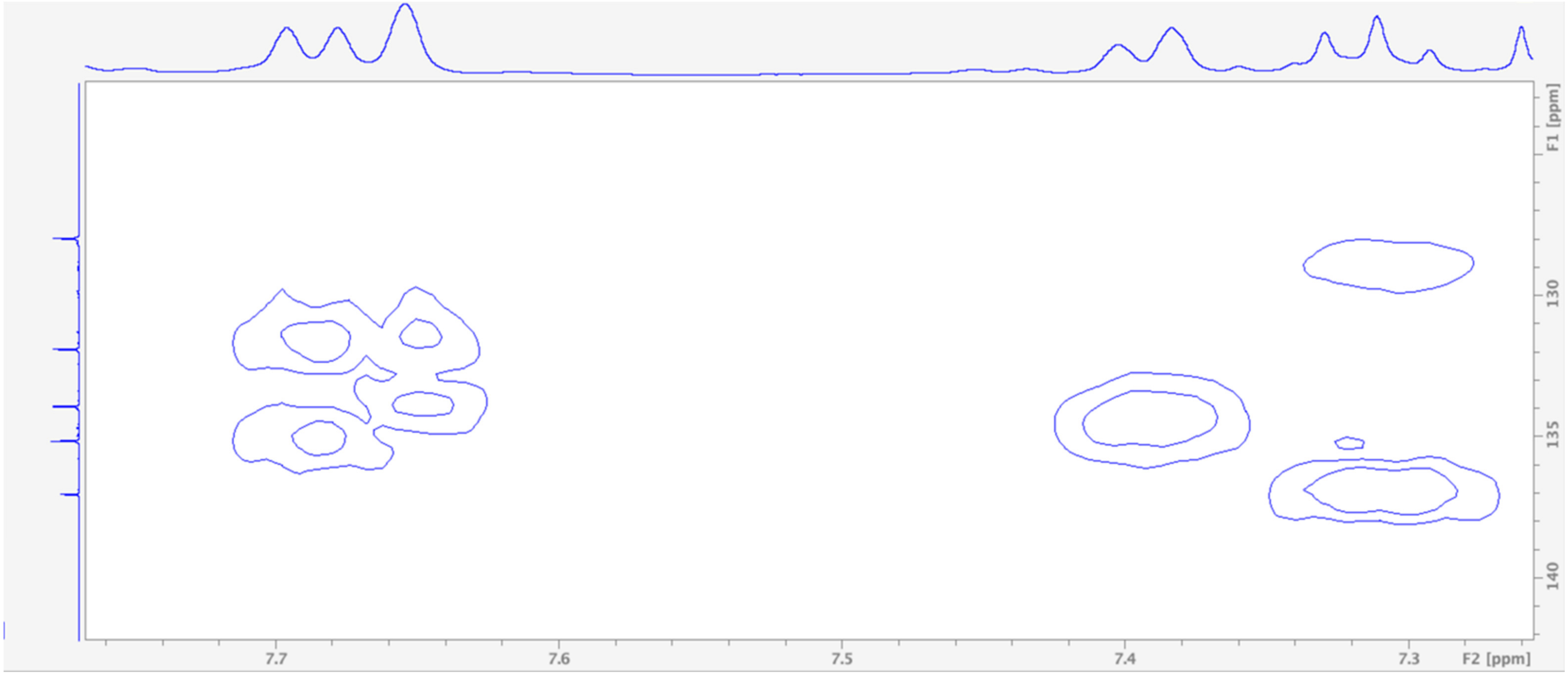

F1 [ppm]
130
135
140
7.7
7.6
7.5
7.4
7.3
F2 [ppm]

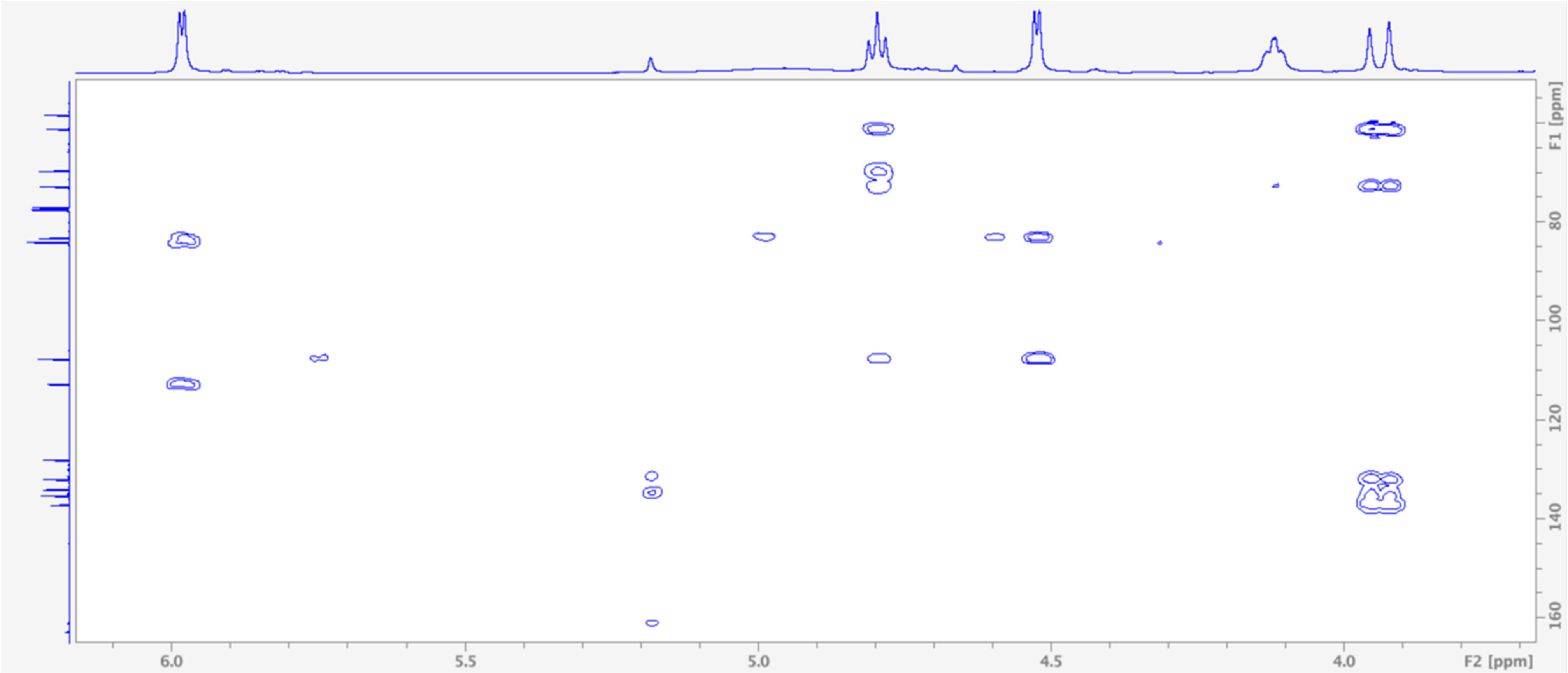

F1 [ppm]
80
100
120
140
160
6.0
5.5
5.0
4.5
4.0
F2 [ppm]

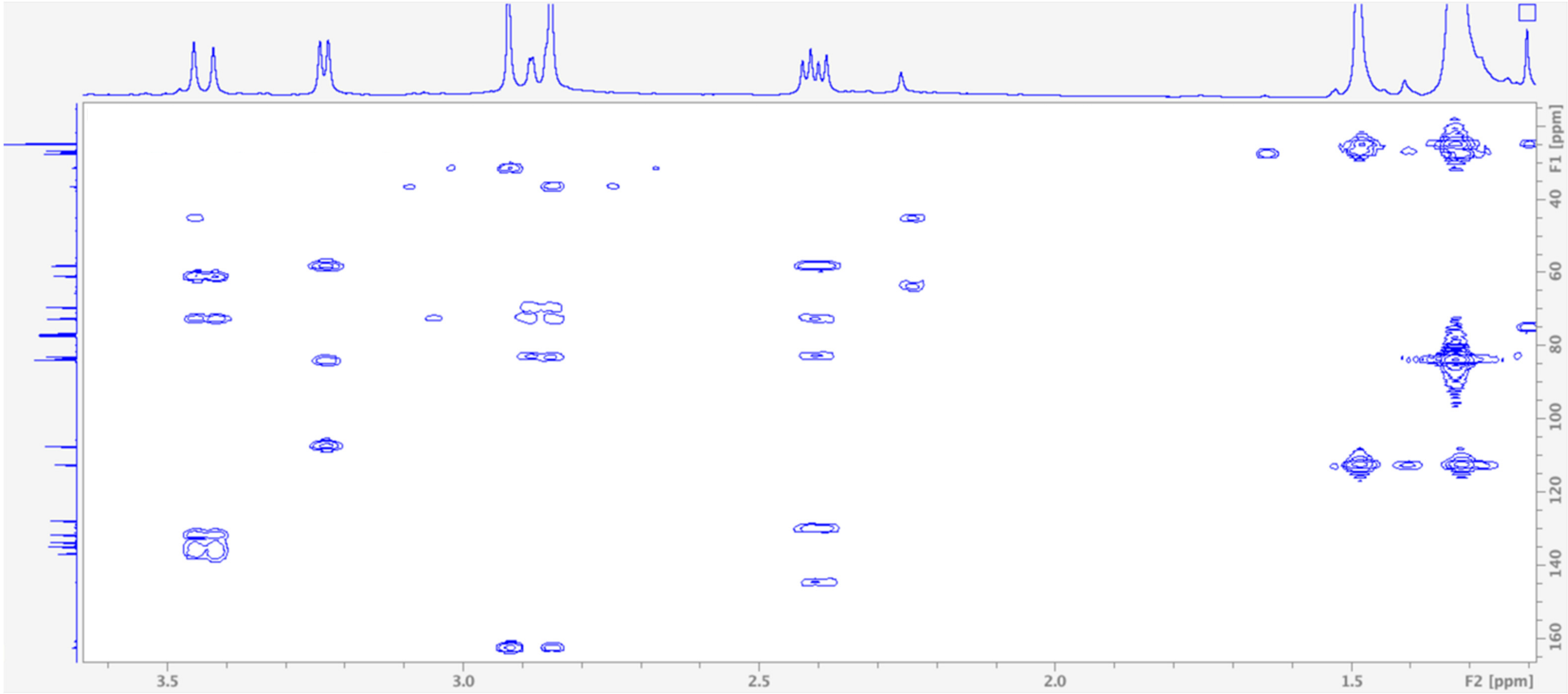

F1 [ppm]
40
60
80
100
120
140
160
3.5
3.0
2.5
2.0
1.5
F2 [ppm]

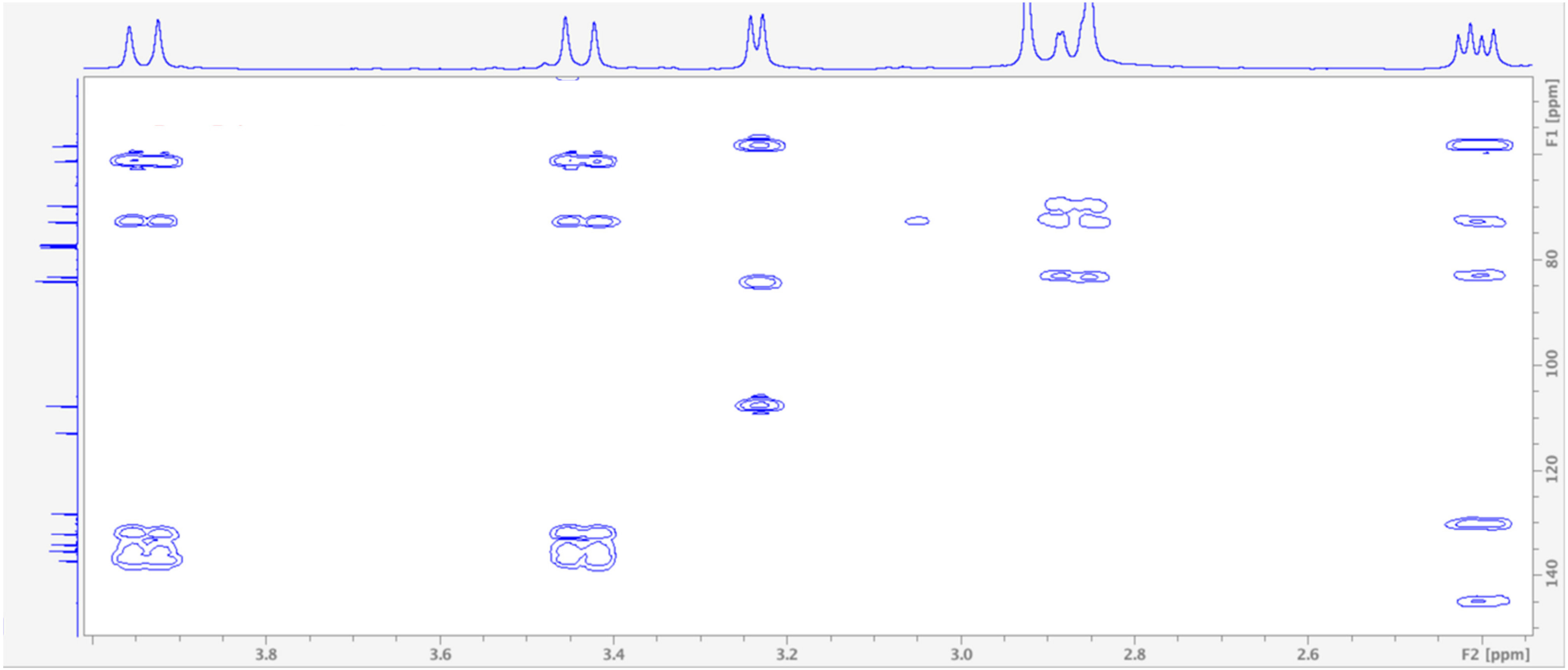

F1 [ppm]
80
100
120
140
3.8
3.6
3.4
3.2
3.0
2.8
2.6
F2 [ppm]

Figure S2. ^{1}H- (400 MHz), ^{13}C-NMR (100 MHz), DEPT, ^{11}B-NMR (128 MHz), COSY, HSQC and HMBC spectra of *N*-(3-methylphenyl boronic acid)-3,6-dideoxy-3,6-imino-D-gulofuranose **meta 3** in D_2O.

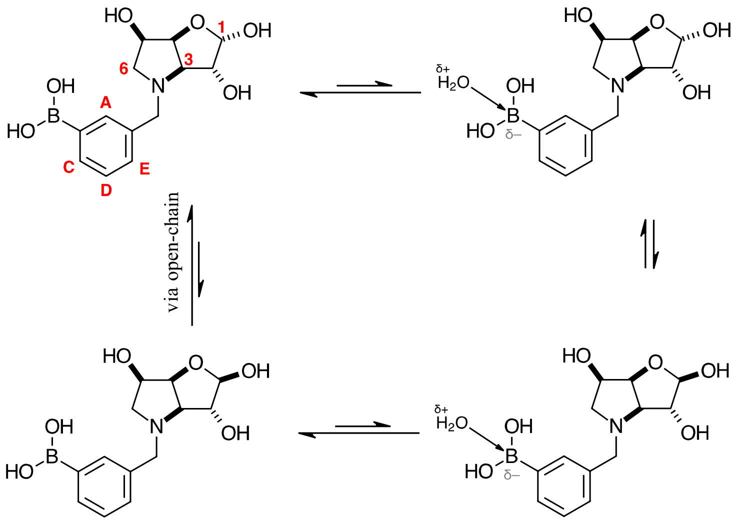

Ratio *α-fur* anomer : *β-fur* anomer : open-chain, 1.0 : 0.5 : 0.002.

***α-fur* anomer**. δ_H: 7.80 (1H, dd, $J_{ArHC,ArHD}$ 7.6 Hz, $J_{ArHC,ArHA}$ 1.2 Hz, ArH^C), 7.78 (1H, d, $J_{ArHA,ArHC}$ 1.6 Hz, ArH^A), 7.58 (1H, dt, $J_{ArHE,ArHD}$ 7.5 Hz, $J_{ArHE,ArHA}$ 1.9 Hz, ArH^E), 7.48 (1H, t, $J_{ArHD,ArHC/HE}$ 7.6 Hz, ArH^D), 5.44 (1H, d, $J_{H\text{-}1,H\text{-}2}$ 4.3 Hz, H-1), 4.93 (1H, app-d, J 5.6 Hz, H-4), 4.51 (1H, d, $J_{Ha,Hb}$ 13.1 Hz, H^a), 4.44 (1H, d, $J_{Hb,Ha}$ 13.1 Hz, H^b), 4.42-4.35 (2H, m, H-2 and H-5), 4.27-4.20 (1H, m, H-3), 3.60-3.52 (1H, m, H-6), 3.49 (1H, dd, $J_{H\text{-}6',H\text{-}6}$ 12.2 Hz, $J_{H\text{-}6',H\text{-}5}$ 3.5 Hz, H-6′); δ_C: 142.4 (ArC$_{quat}$), 136.2 (ArC^A), 135.4 (ArC^C), 133.5 (ArC^E), 128.9 (ArC^D), 99.0 (C-1, broadened, 89 Hz), 78.9 (C-4, broadened, 88 Hz), 72.8 (C-2 and C-3, broadened, 68 Hz), 67.4 (C-5), 61.9-59.7 (C-6, broadened, 226 Hz), 59.7-58.4 (CH_2Ar broadened, 170 Hz); Not discernible: ArC^B-B.

***β-fur* anomer**. δ_H: 7.82-7.76 (1H, obscured, ArH^A), 7.60 (1H, dd, $J_{ArHC,ArHD}$ 5.5 Hz, $J_{ArHC,ArHA}$ 1.9 Hz, ArH^C), 7.50 (1H, t, $J_{ArHD,ArHC/HE}$ 7.3 Hz, ArH^D), 7.27 (1H, dd, $J_{ArHE,ArHD}$ 8.5 Hz, $J_{ArHE,ArHA}$ 0.6 Hz, ArH^E), 5.29 (1H, d, $J_{H\text{-}1,H\text{-}2}$ 2.1 Hz, H-1), 4.91 (1H, app-d, $J_{H\text{-}4,H\text{-}3}$ 5.6 Hz, H-4), 4.57 (1H, d, $J_{Ha,Hb}$ 13.0 Hz, H^a), 4.35 (1H, d, $J_{Hb,Ha}$ 13.0 Hz, H^b), 4.49-4.44 (1H, obscured, H-5), 4.42-4.35 (2H, m, H-2), 4.16 (1H, dd, $J_{H\text{-}3,H\text{-}4}$ 5.8 Hz, $J_{H\text{-}3,H\text{-}2}$ 1.8 Hz, H-3), 3.74 (1H, app-dd, $J_{H\text{-}6,H\text{-}6'}$ 12.3 Hz, $J_{H\text{-}6,H\text{-}5}$ 4.4 Hz, H-6), 3.52-3.47 (1H, obscured, H-6′); δ_C: 139.6 (ArC$_{quat}$), 135.9 (ArC^A), 135.5 (ArC^C), 129.4 (ArC^E), 129.0 (ArC^D), 103.1 (C-1, broadened, 29 Hz), 80.7 (C-4, slightly broadened, 30 Hz), 72.7 (C-2 and C-3, broadened, 68 Hz), 67.5 (C-5), 61.9-59.7 (C-6, broadened, 226 Hz), 59.7-58.4 (CH_2Ar broadened, 170 Hz); Not discernible: ArC^B-B.

Open-chain. δ_H: 9.86 (1H, s, CHO).

δ_B: 28.6 (broad, integration: 6.5), 19.2 (sharp, integration: 1.0).

^{1}H-NMR

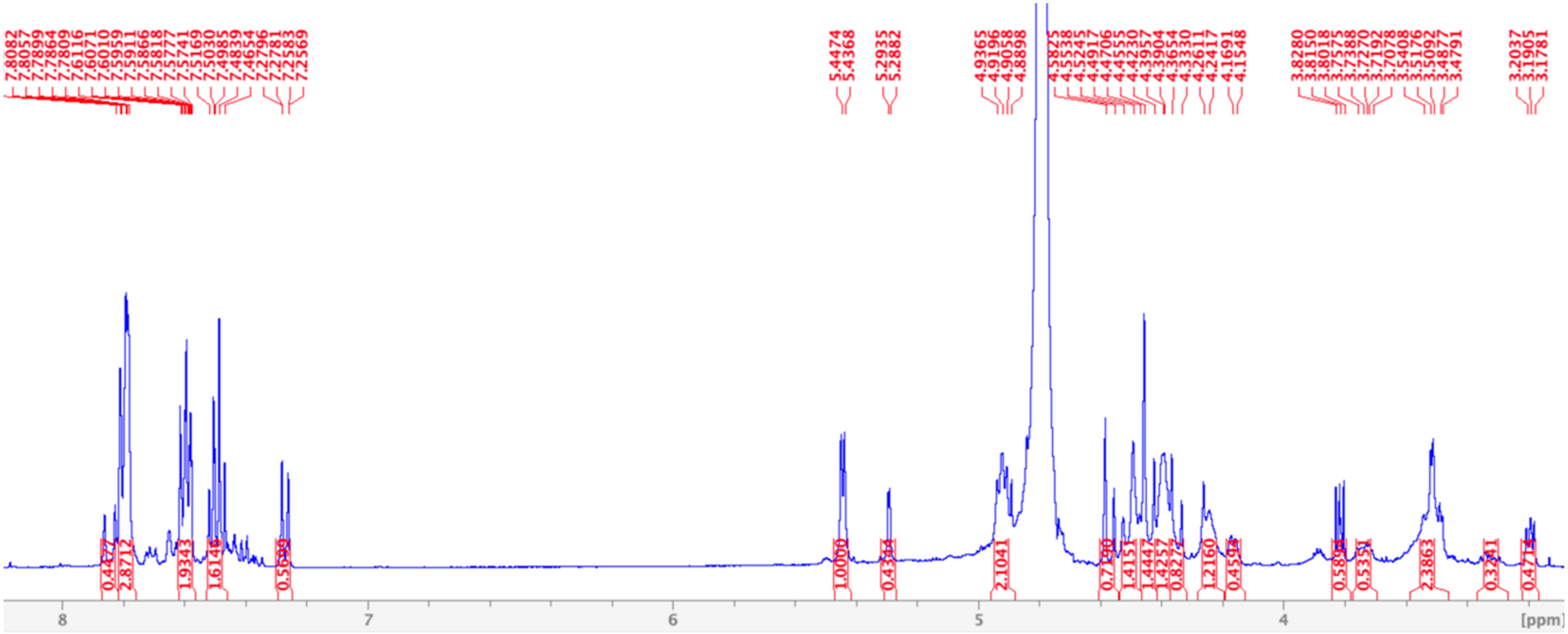

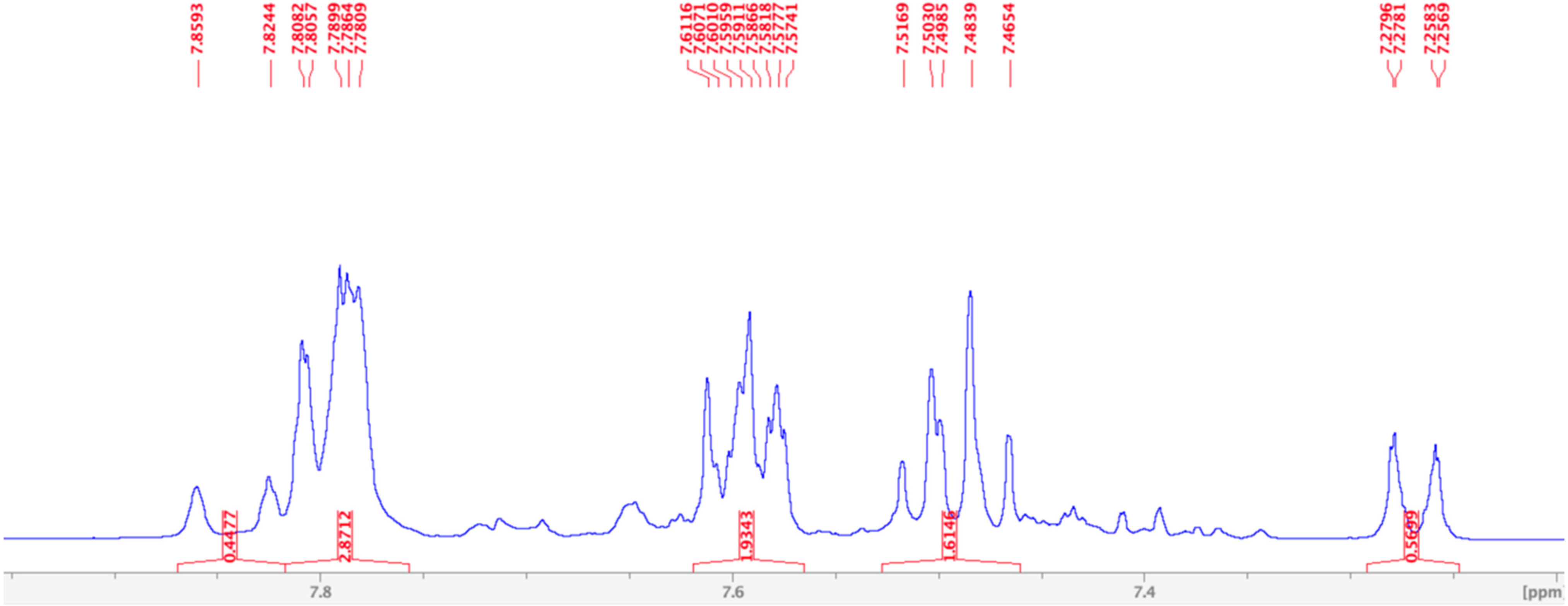
7.8593
7.8244
7.8082
7.8057
7.7899
7.7864
7.7809
7.6116
7.6071
7.6010
7.5959
7.5911
7.5866
7.5818
7.5777
7.5741
7.5169
7.5030
7.4985
7.4839
7.4654
7.2796
7.2781
7.2583
7.2569
0.4477
2.8712
1.9343
1.6146
0.5699
7.8
7.6
7.4
[ppm]

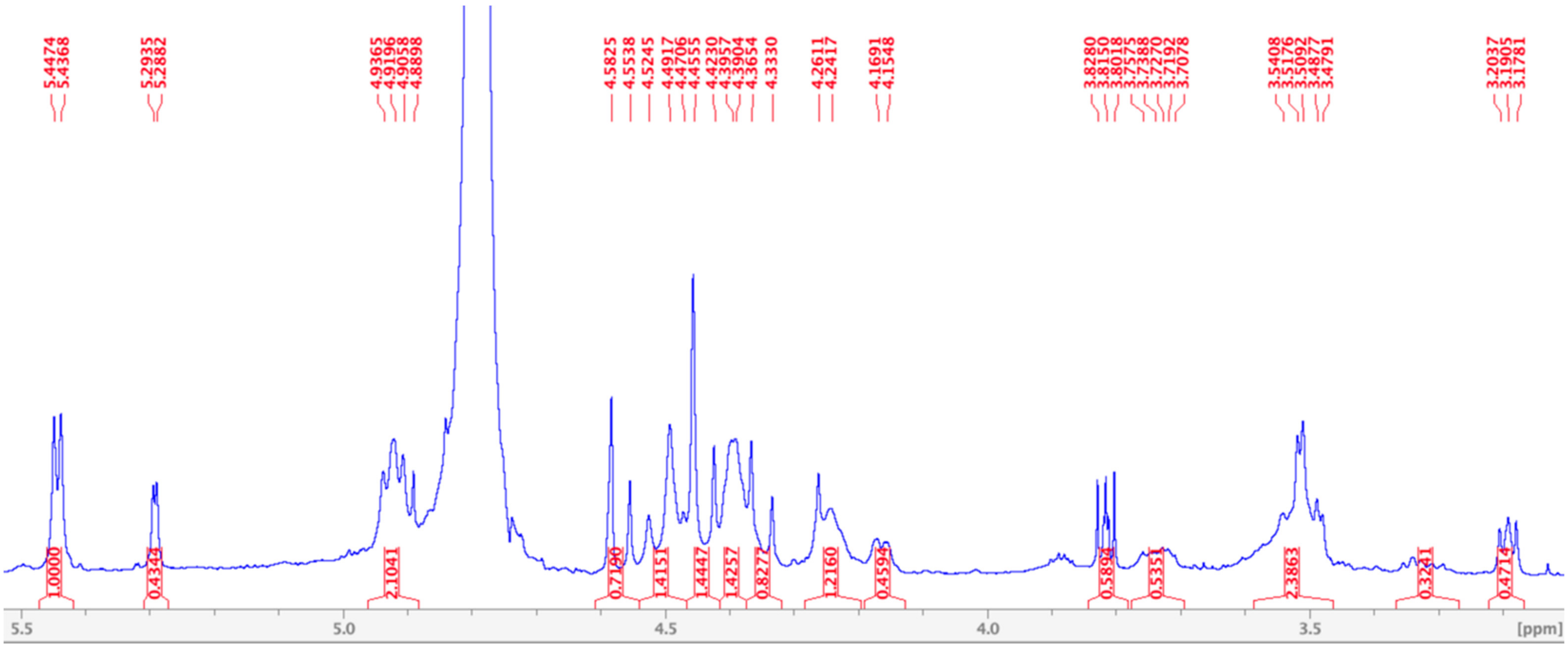
5.4474
5.4368
5.2935
5.2882
4.9365
4.9196
4.9058
4.8898
4.5825
4.5538
4.5245
4.4917
4.4706
4.4555
4.4230
4.3957
4.3904
4.3654
4.3330
4.2611
4.2417
4.1691
4.1548
3.8280
3.8150
3.8018
3.7575
3.7388
3.7270
3.7192
3.7078
3.5408
3.5176
3.5092
3.4877
3.4791
3.2037
3.1905
3.1781
1.0000
0.4344
2.1041
0.7190
1.4151
1.4447
1.4257
0.8277
1.2160
0.4594
0.5894
0.5351
2.3863
0.3241
0.4714
5.5
5.0
4.5
4.0
3.5
[ppm]

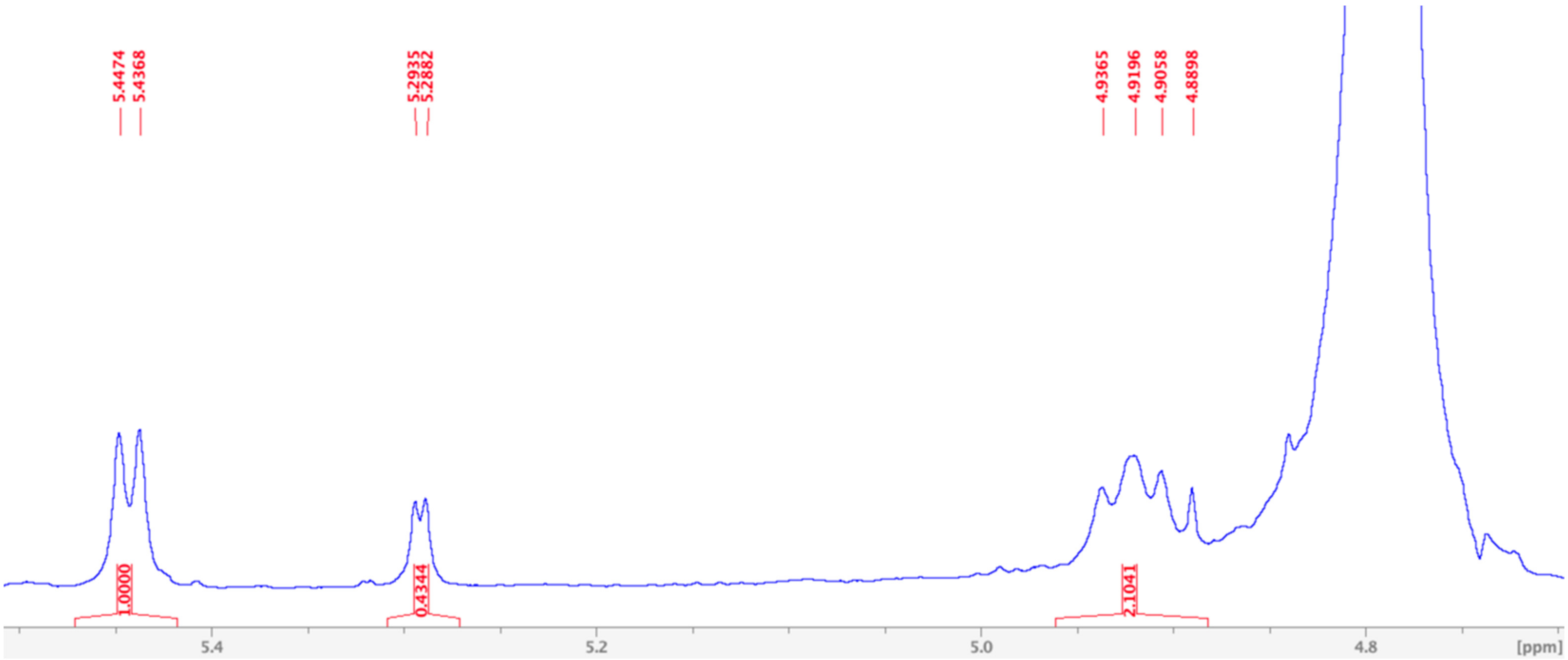
5.4474
5.4368
5.2935
5.2882
4.9365
4.9196
4.9058
4.8898
1.0000
0.4344
2.1041
5.4
5.2
5.0
4.8
[ppm]

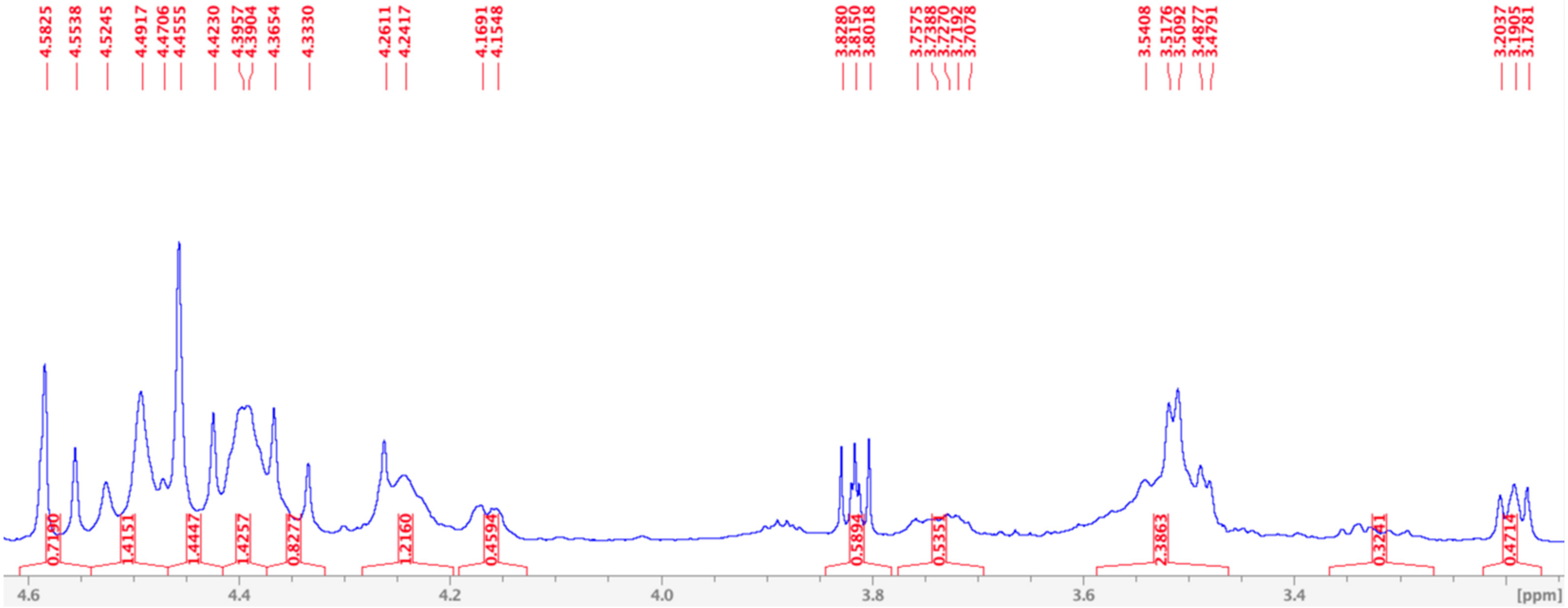
4.5825
4.5538
4.5245
4.4917
4.4706
4.4555
4.4230
4.3957
4.3904
4.3654
4.3330
4.2611
4.2417
4.1691
4.1548
3.8280
3.8150
3.8018
3.7575
3.7388
3.7270
3.7192
3.7078
3.5408
3.5176
3.5092
3.4877
3.4791
3.2037
3.1905
3.1781
0.7190
1.4151
1.4447
1.4257
0.8277
1.2160
0.4594
0.5894
0.5351
2.3863
0.3241
0.4714
4.6
4.4
4.2
4.0
3.8
3.6
3.4
[ppm]

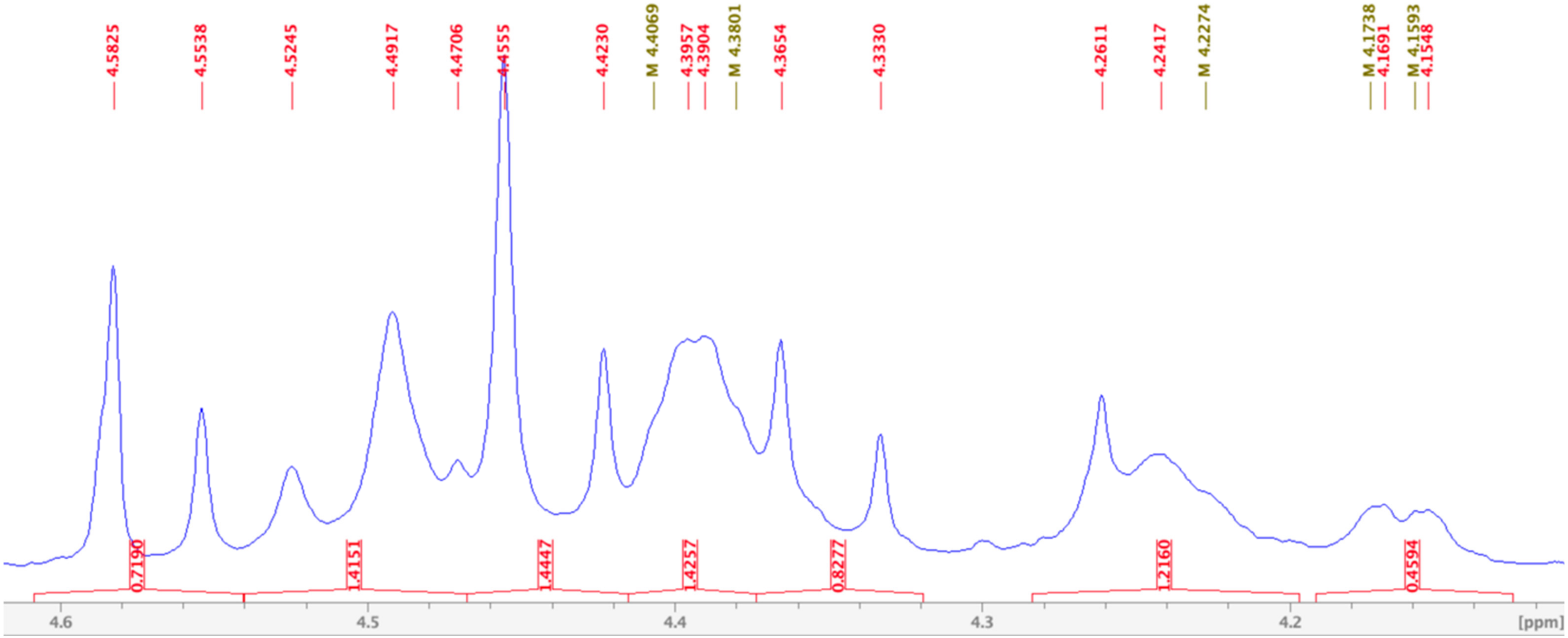
4.5825
4.5538
4.5245
4.4917
4.4706
4.4555
4.4230
M 4.4069
4.3957
4.3904
M 4.3801
4.3654
4.3330
4.2611
4.2417
M 4.2274
M 4.1738
4.1691
M 4.1593
4.1548
0.7190
1.4151
1.4447
1.4257
0.8277
1.2160
0.4594
4.6
4.5
4.4
4.3
4.2
[ppm]

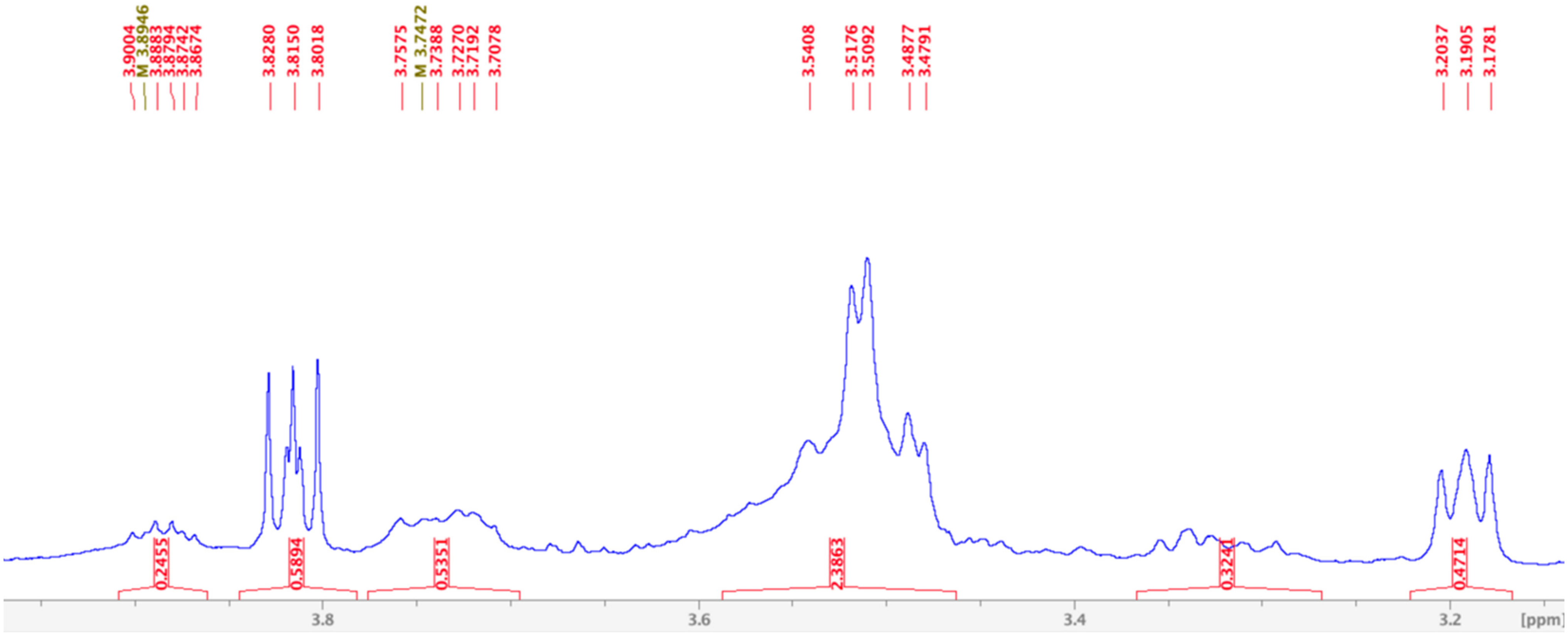
3.9004
M 3.8946
3.8883
3.8794
3.8742
3.8674
3.8280
3.8150
3.8018
3.7575
M 3.7472
3.7388
3.7270
3.7192
3.7078
3.5408
3.5176
3.5092
3.4877
3.4791
3.2037
3.1905
3.1781
0.2455
0.5894
0.5351
2.3863
0.3241
0.4714
3.8
3.6
3.4
3.2
[ppm]

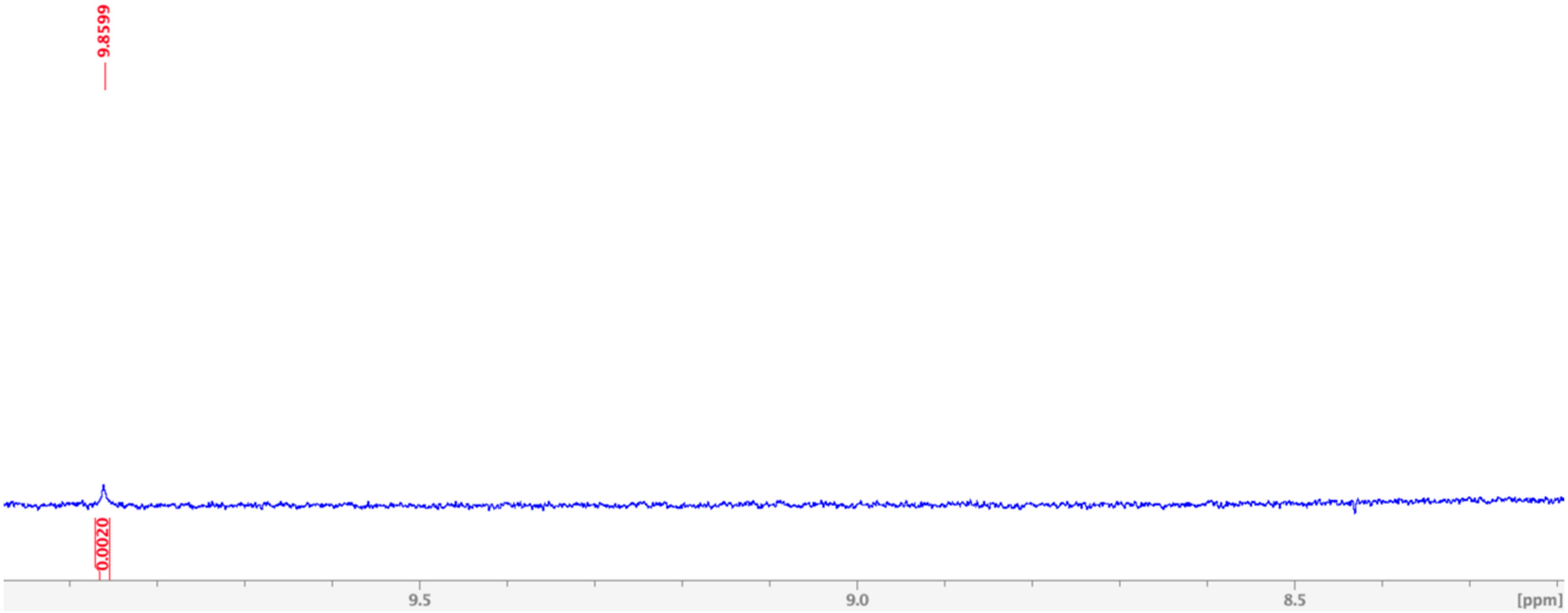
9.8599
0.0020
9.5
9.0
8.5
[ppm]

^{13}C-NMR

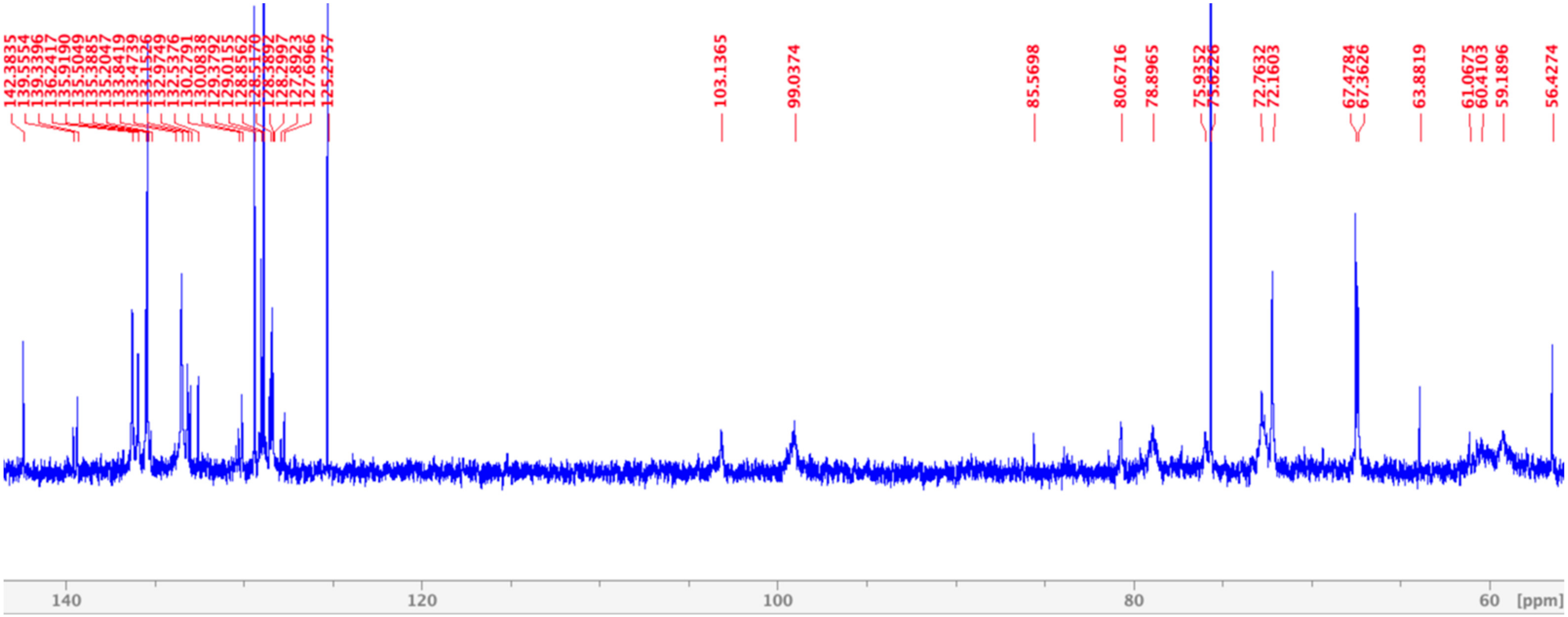
142.3835
139.5554
139.3396
136.2417
135.9190
135.5049
135.3885
135.2047
133.8419
133.4739
133.1526
132.9749
132.5376
130.2791
130.0838
129.3792
129.0155
128.8562
128.5170
128.3892
128.2997
127.8923
127.6966
125.2757
103.1365
99.0374
85.5698
80.6716
78.8965
75.9352
75.6226
72.7632
72.1603
67.4784
67.3626
63.8819
61.0675
60.4103
59.1896
56.4274
140
120
100
80
60
[ppm]

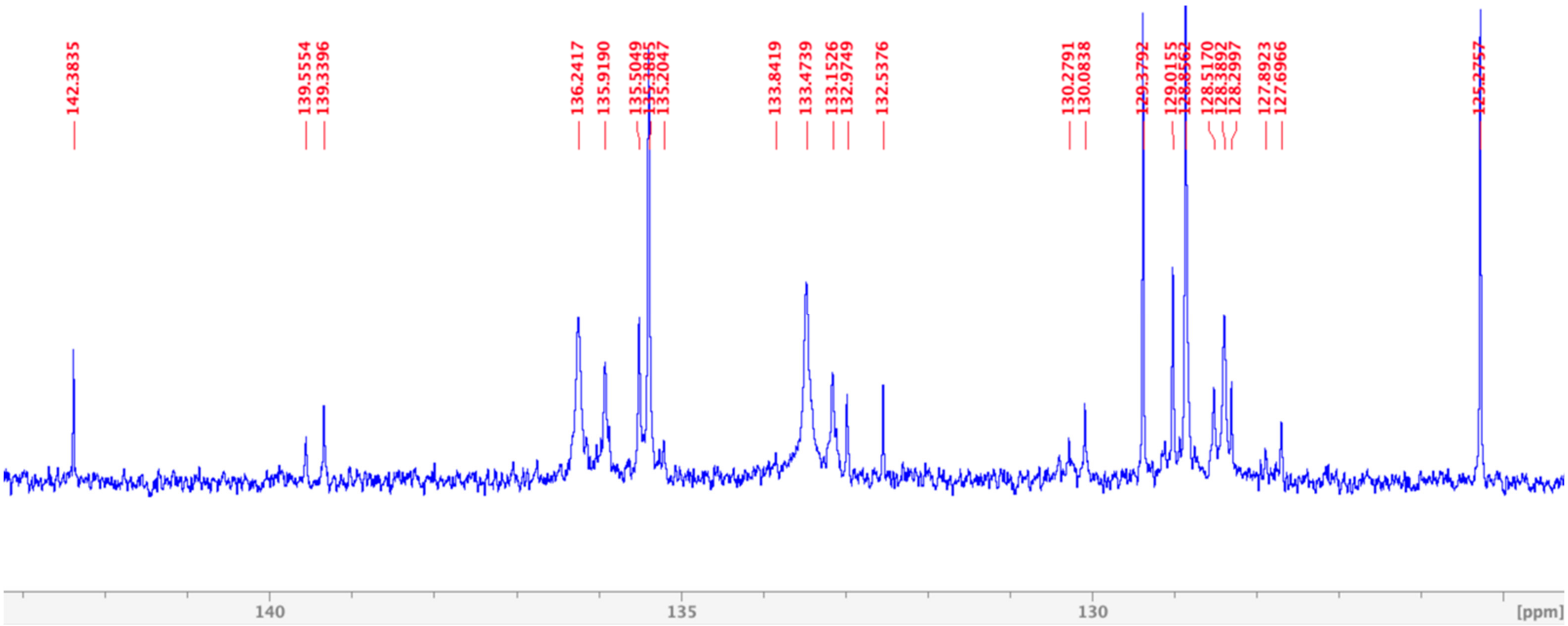
142.3835
139.5554
139.3396
136.2417
135.9190
135.5049
135.3885
135.2047
133.8419
133.4739
133.1526
132.9749
132.5376
130.2791
130.0838
129.3792
129.0155
128.8562
128.5170
128.3892
128.2997
127.8923
127.6966
125.2757
140
135
130
[ppm]

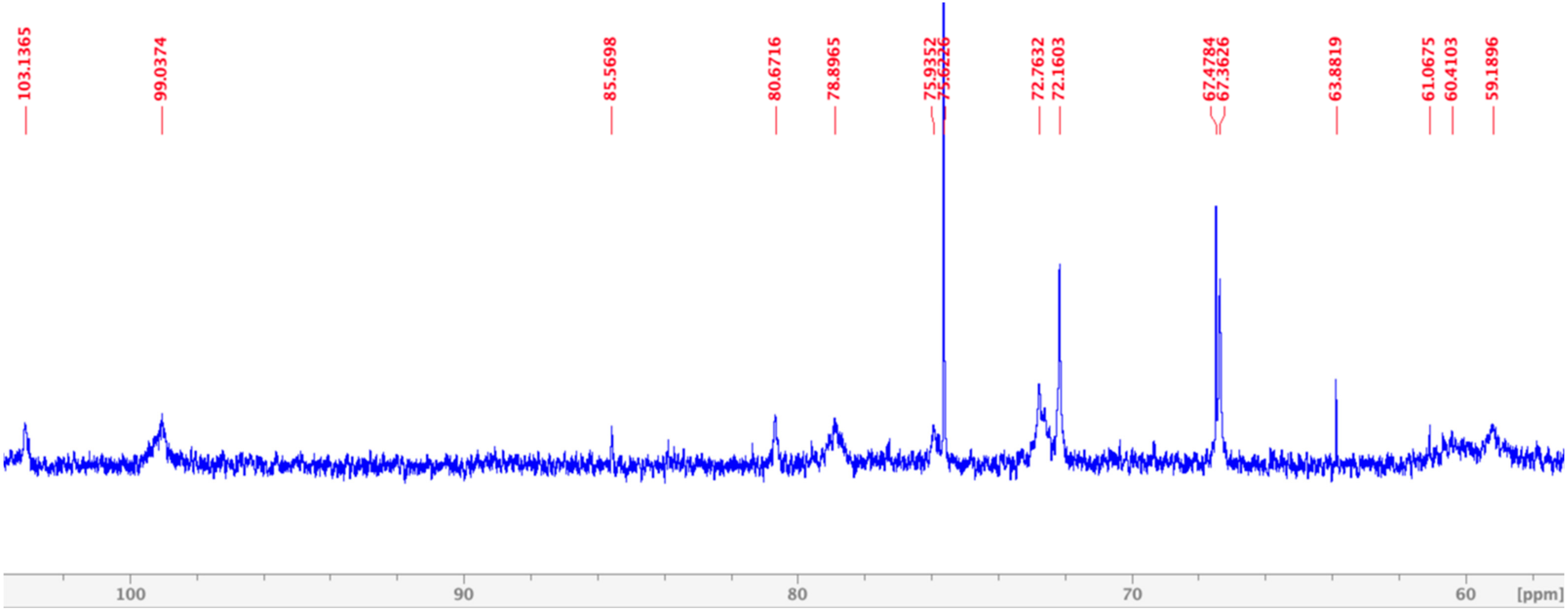
103.1365
99.0374
85.5698
80.6716
78.8965
75.9352
75.6226
72.7632
72.1603
67.4784
67.3626
63.8819
61.0675
60.4103
59.1896
100
90
80
70
60
[ppm]

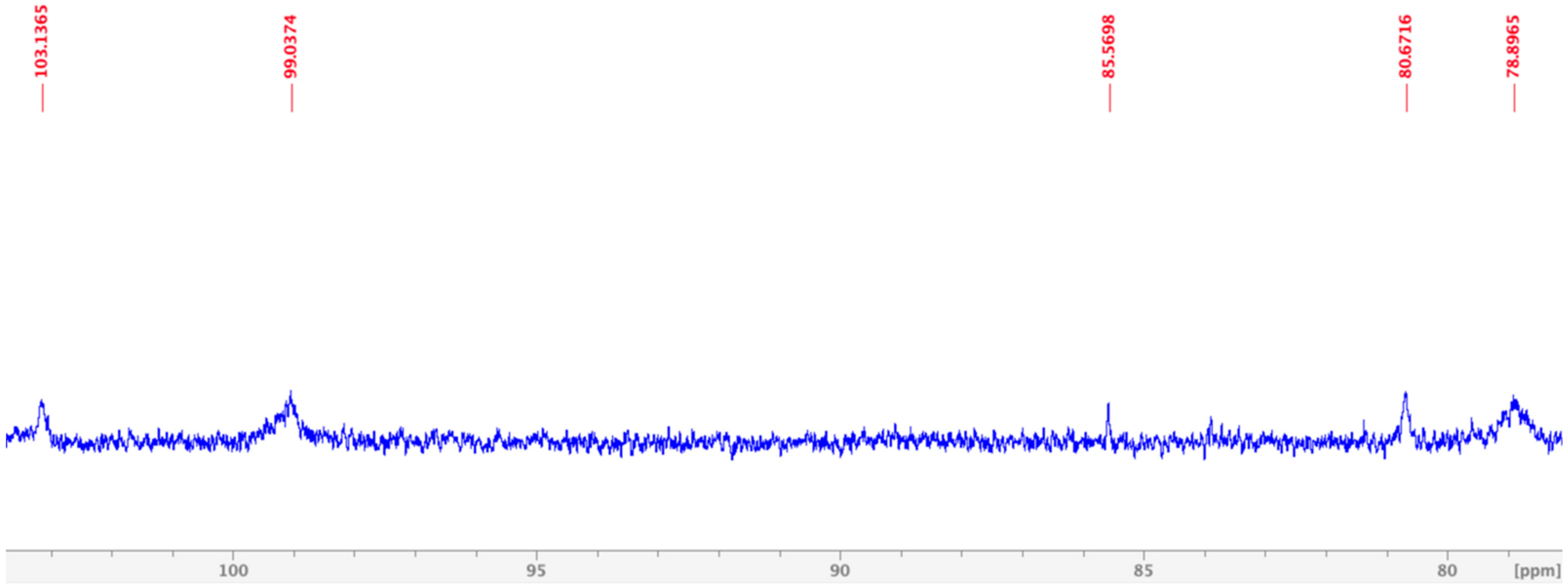
103.1365
99.0374
85.5698
80.6716
78.8965
100
95
90
85
80
[ppm]

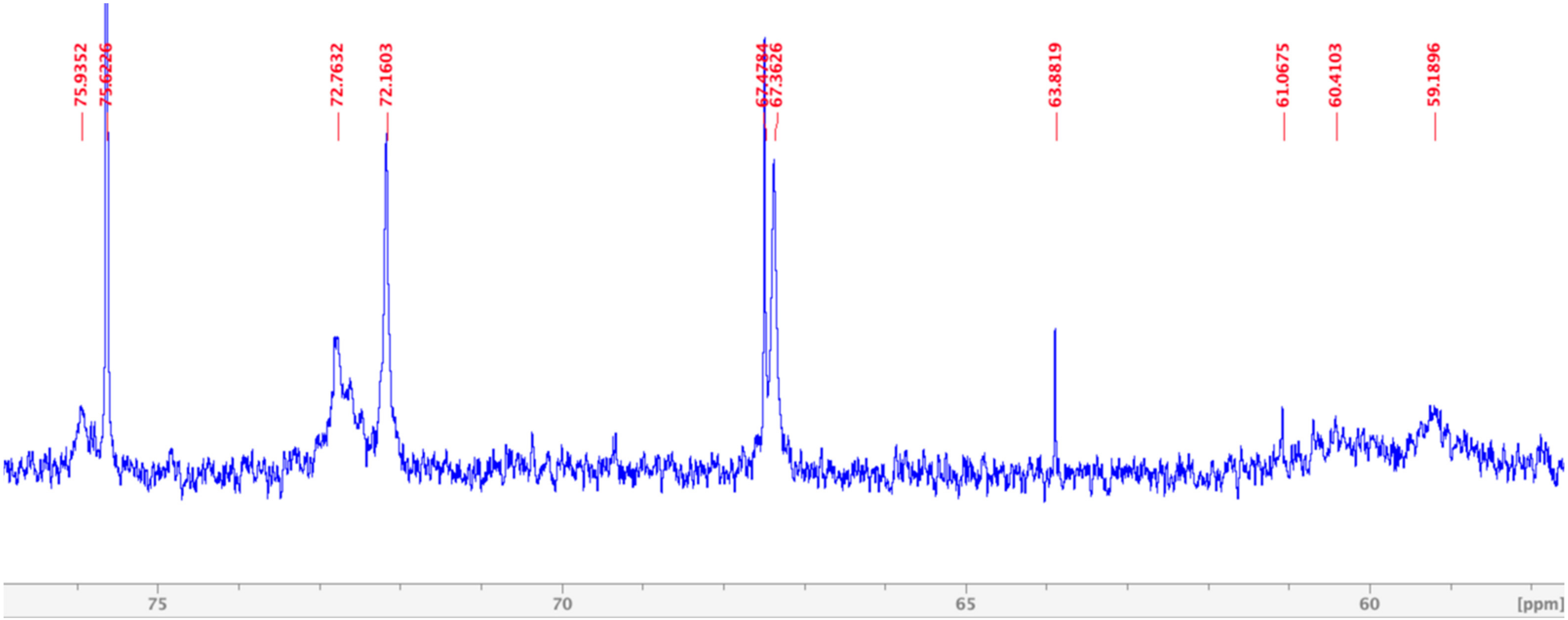

75.9352
75.6226
72.7632
72.1603
67.4784
67.3626
63.8819
61.0675
60.4103
59.1896
75
70
65
60
[ppm]

DEPT

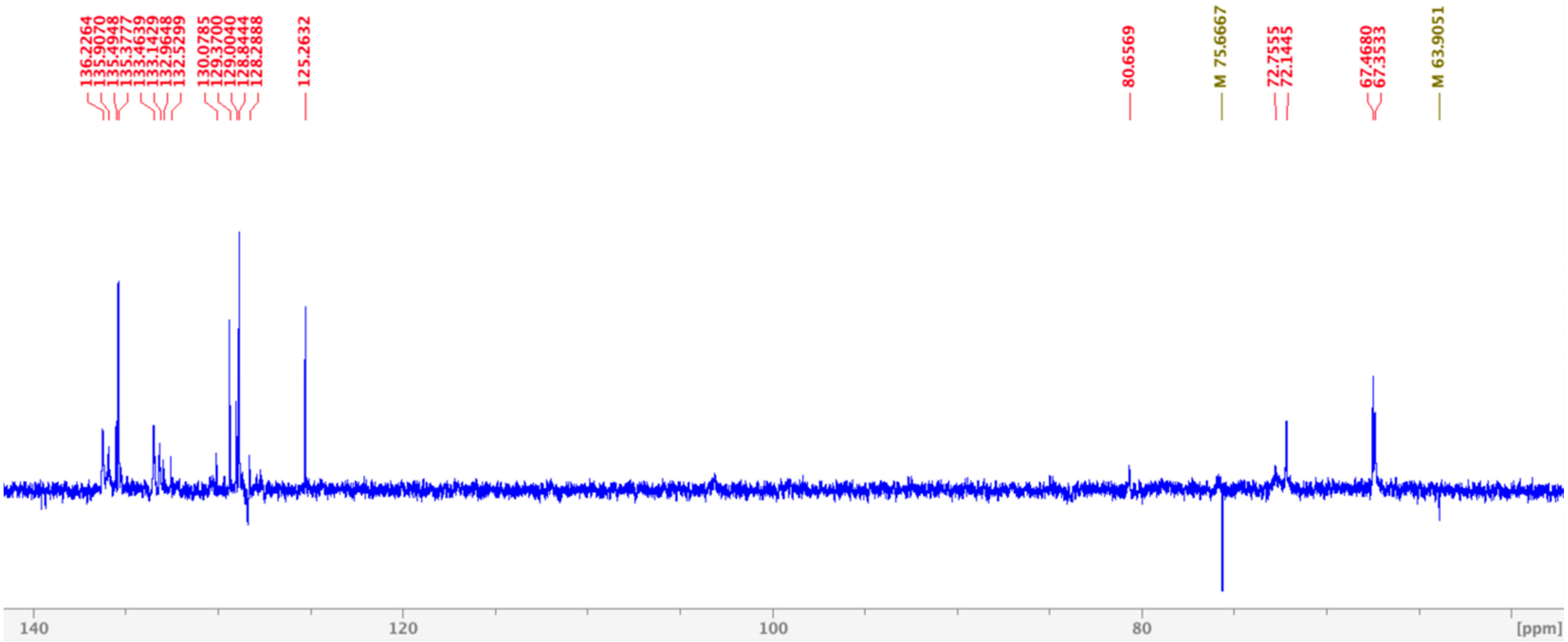

136.2264
135.9070
135.4948
135.3777
133.4639
133.1429
132.9648
132.5299
130.0785
129.3700
129.0040
128.8444
128.2888
125.2632
80.6569
M 75.6667
72.7555
72.1445
67.4680
67.3533
M 63.9051
140
120
100
80
[ppm]

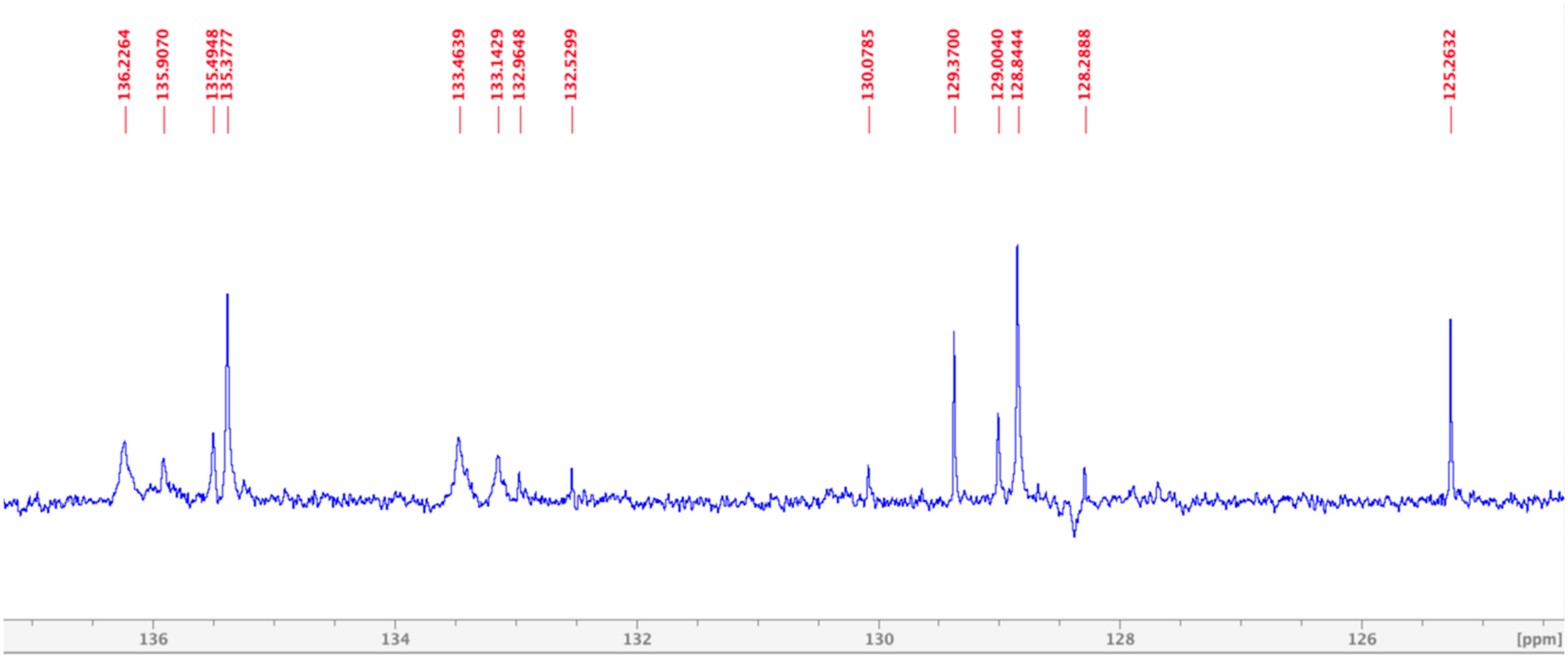
136.2264
135.9070
135.4948
135.3777
133.4639
133.1429
132.9648
132.5299
130.0785
129.3700
129.0040
128.8444
128.2888
125.2632
136
134
132
130
128
126
[ppm]

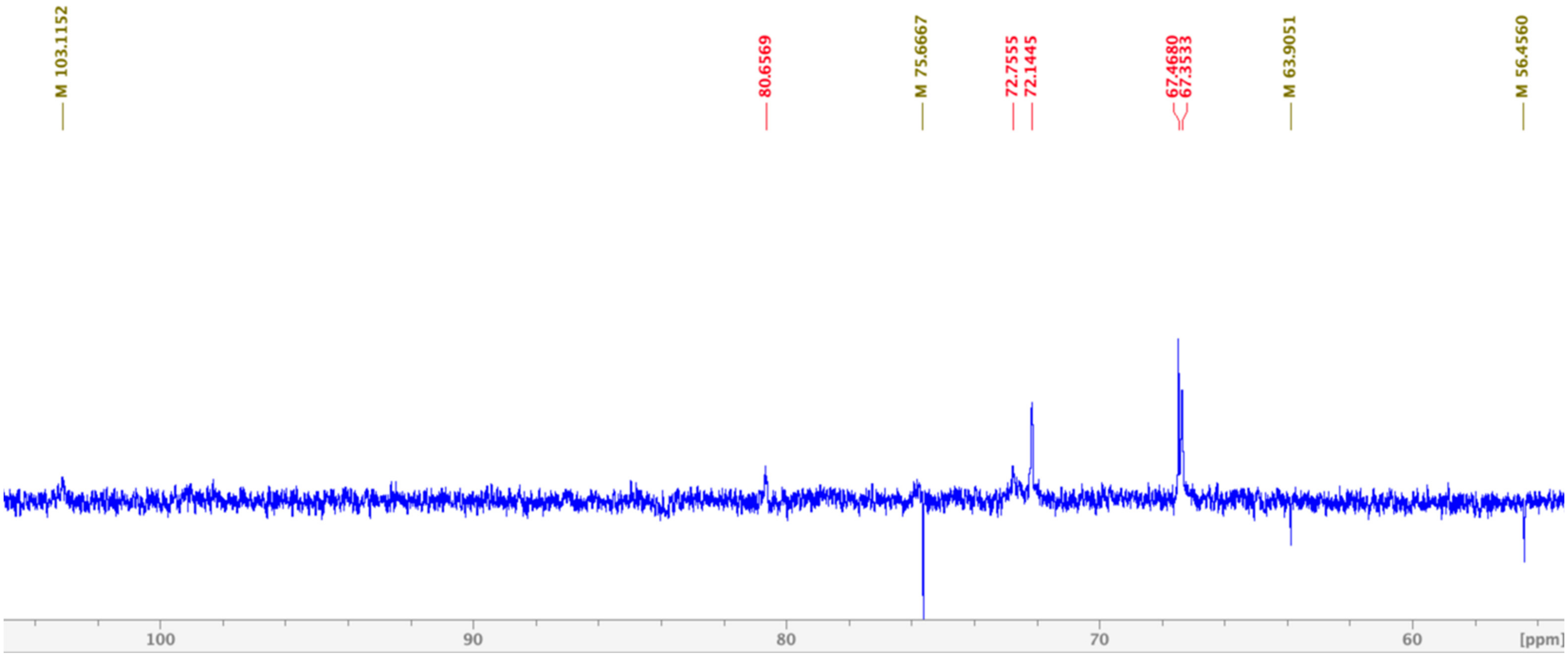
M 103.1152
80.6569
M 75.6667
72.7555
72.1445
67.4680
67.3533
M 63.9051
M 56.4560
100
90
80
70
60
[ppm]

^{11}B-NMR

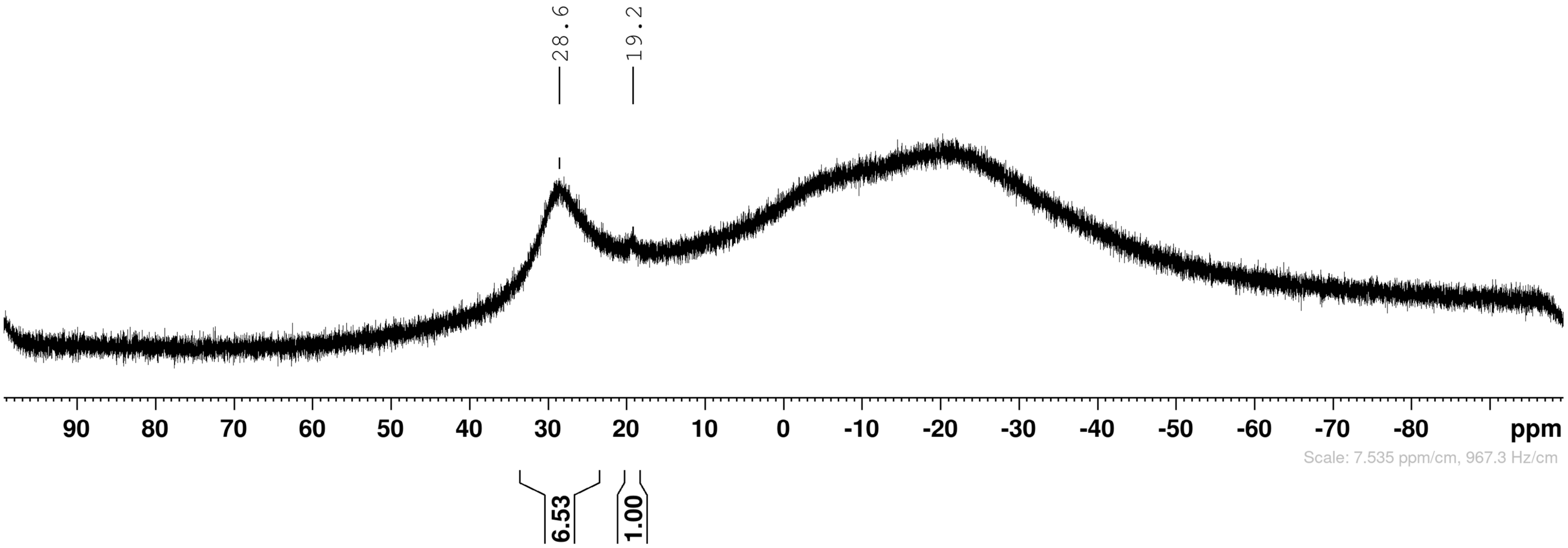

28.6
19.2
90
80
70
60
50
40
30
20
10
0
-10
-20
-30
-40
-50
-60
-70
-80
ppm
Scale: 7.535 ppm/cm, 967.3 Hz/cm
6.53
1.00

COSY

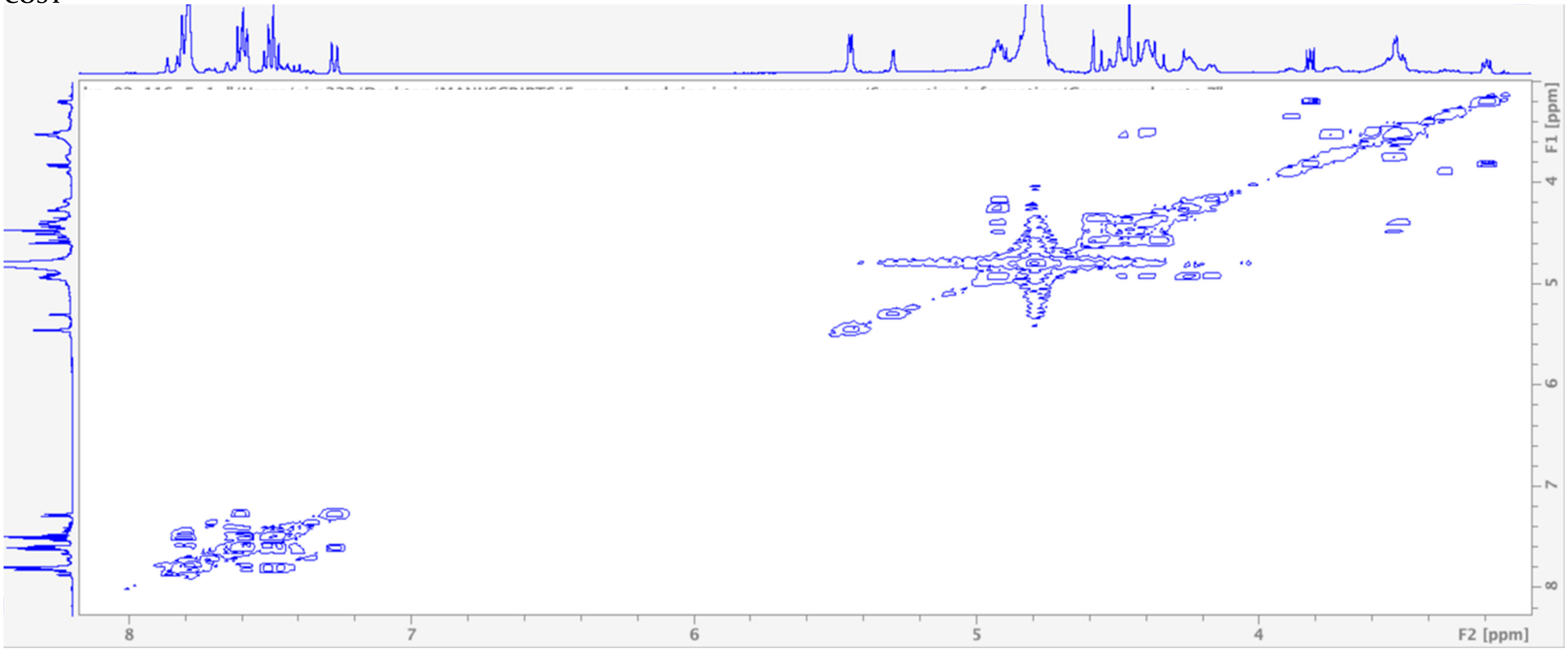

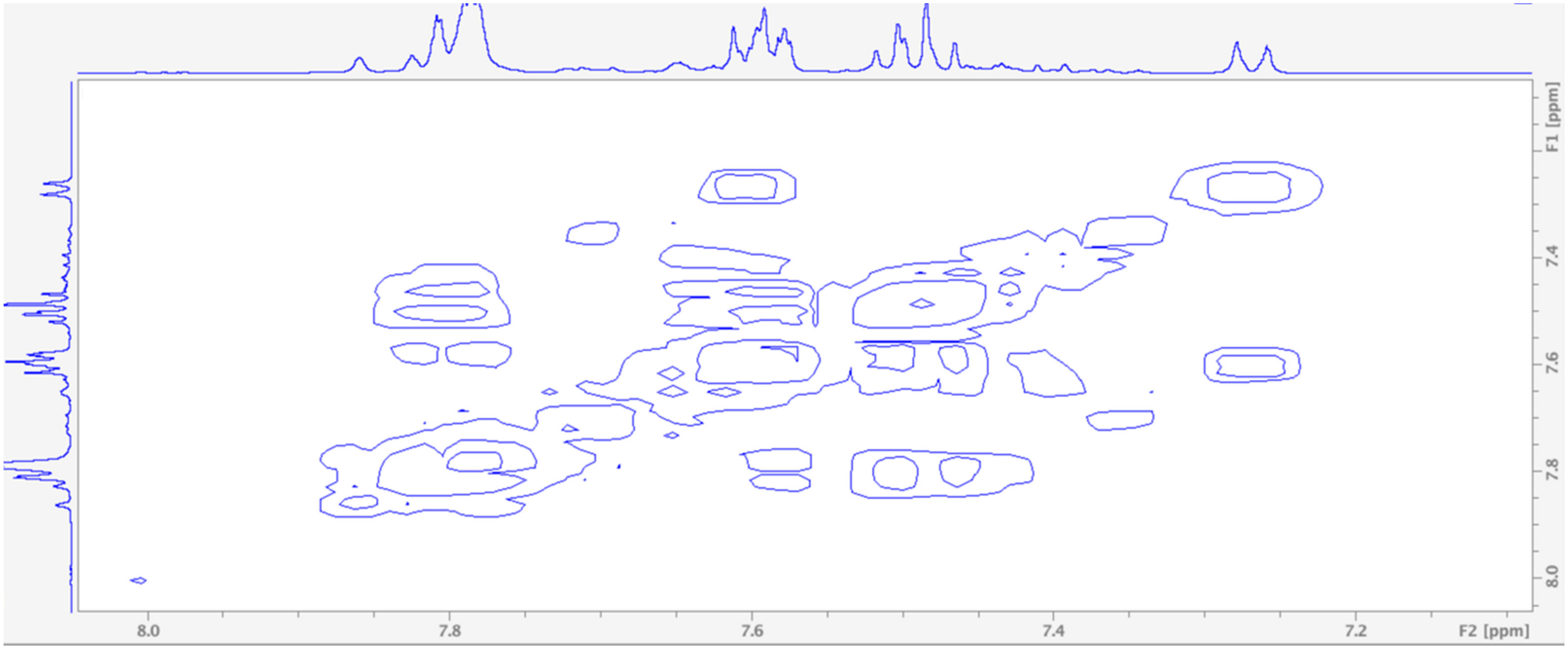
8.0
7.8
7.6
7.4
7.2
F2 [ppm]
F1 [ppm]
7.4
7.6
7.8
8.0

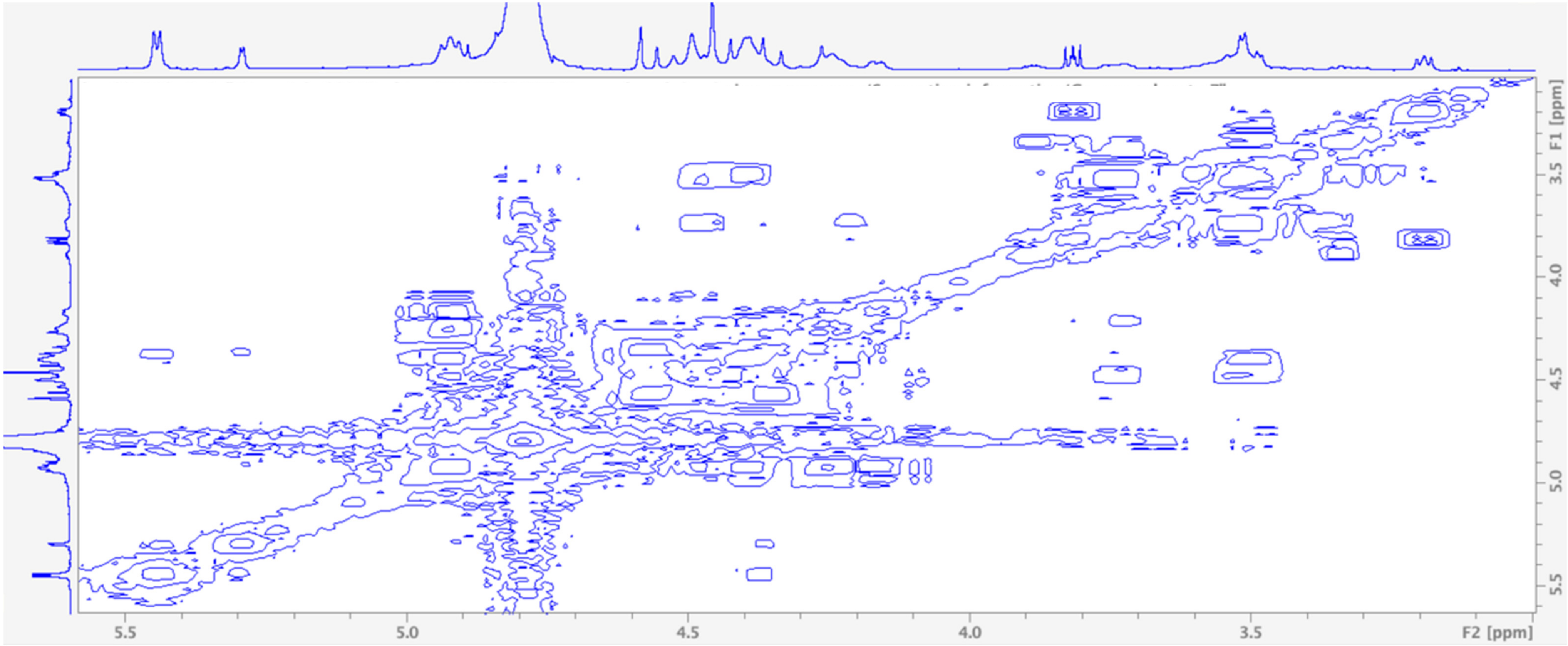

5.5
5.0
4.5
4.0
3.5
F2 [ppm]
3.5
4.0
4.5
5.0
5.5
F1 [ppm]

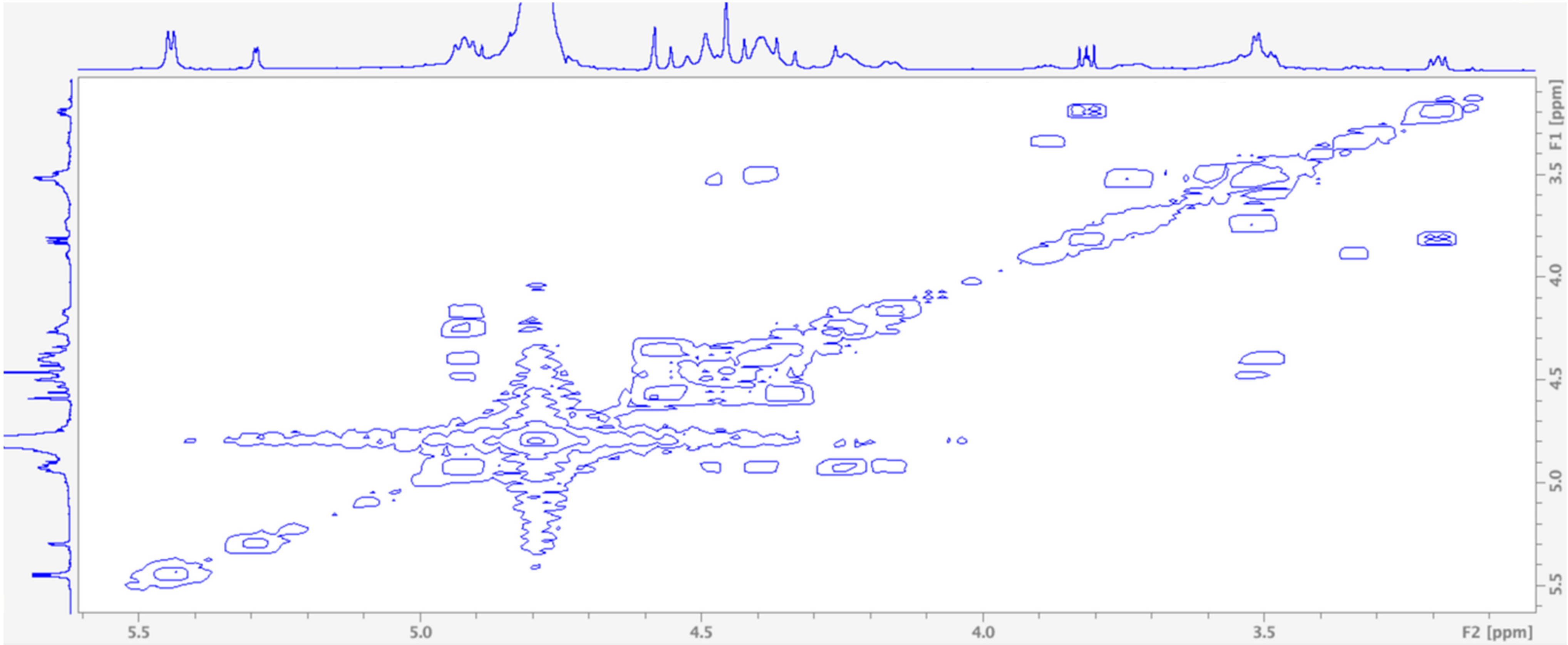
5.5
5.0
4.5
4.0
3.5
F2 [ppm]
F1 [ppm]

HSQC

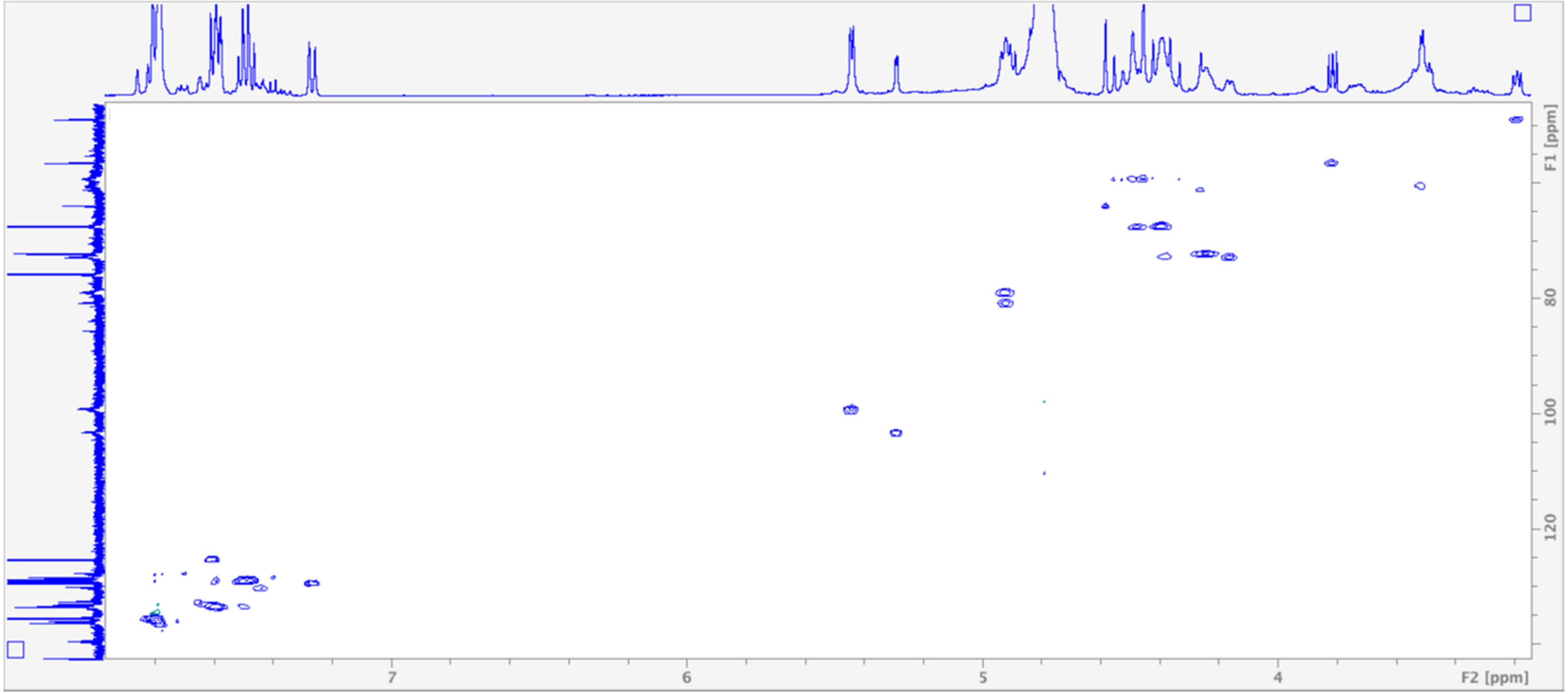

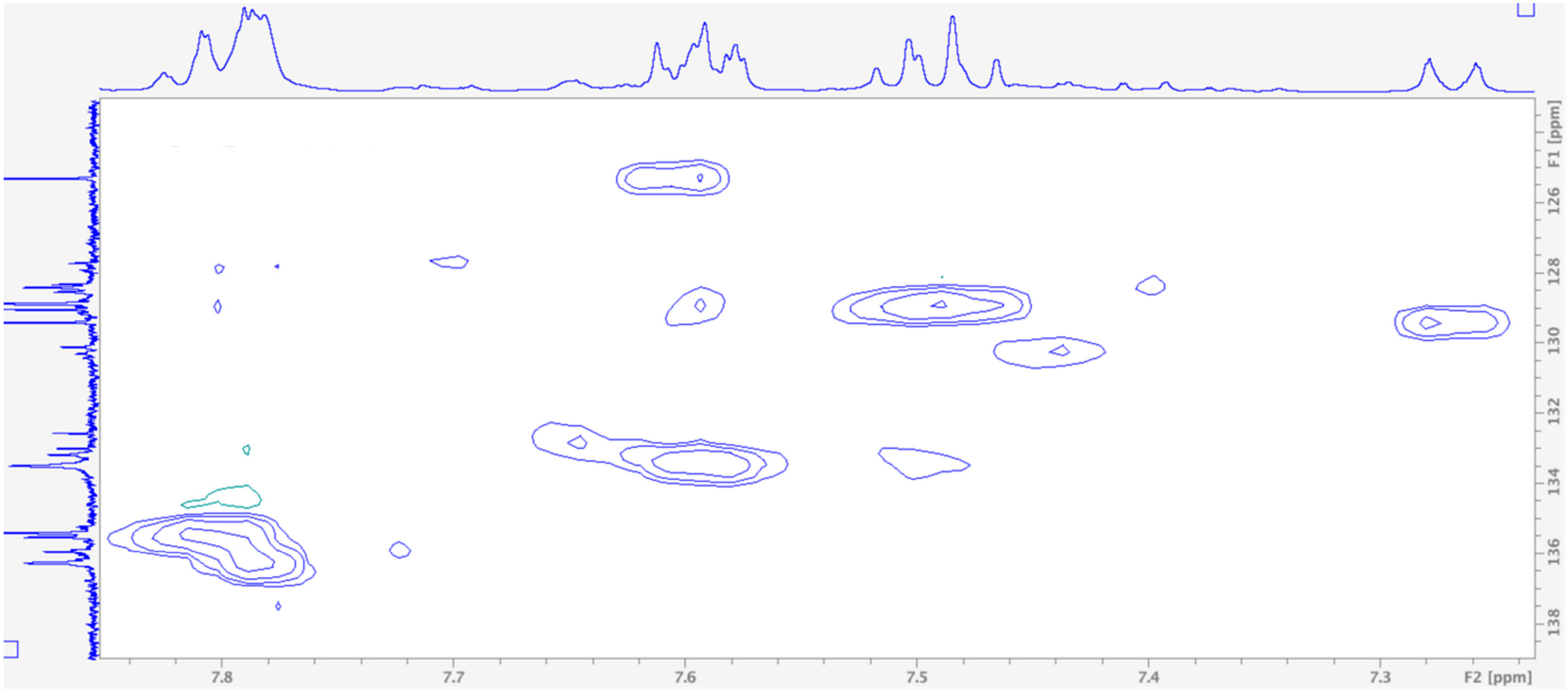

F1 [ppm]
126
128
130
132
134
136
138
7.8
7.7
7.6
7.5
7.4
7.3
F2 [ppm]

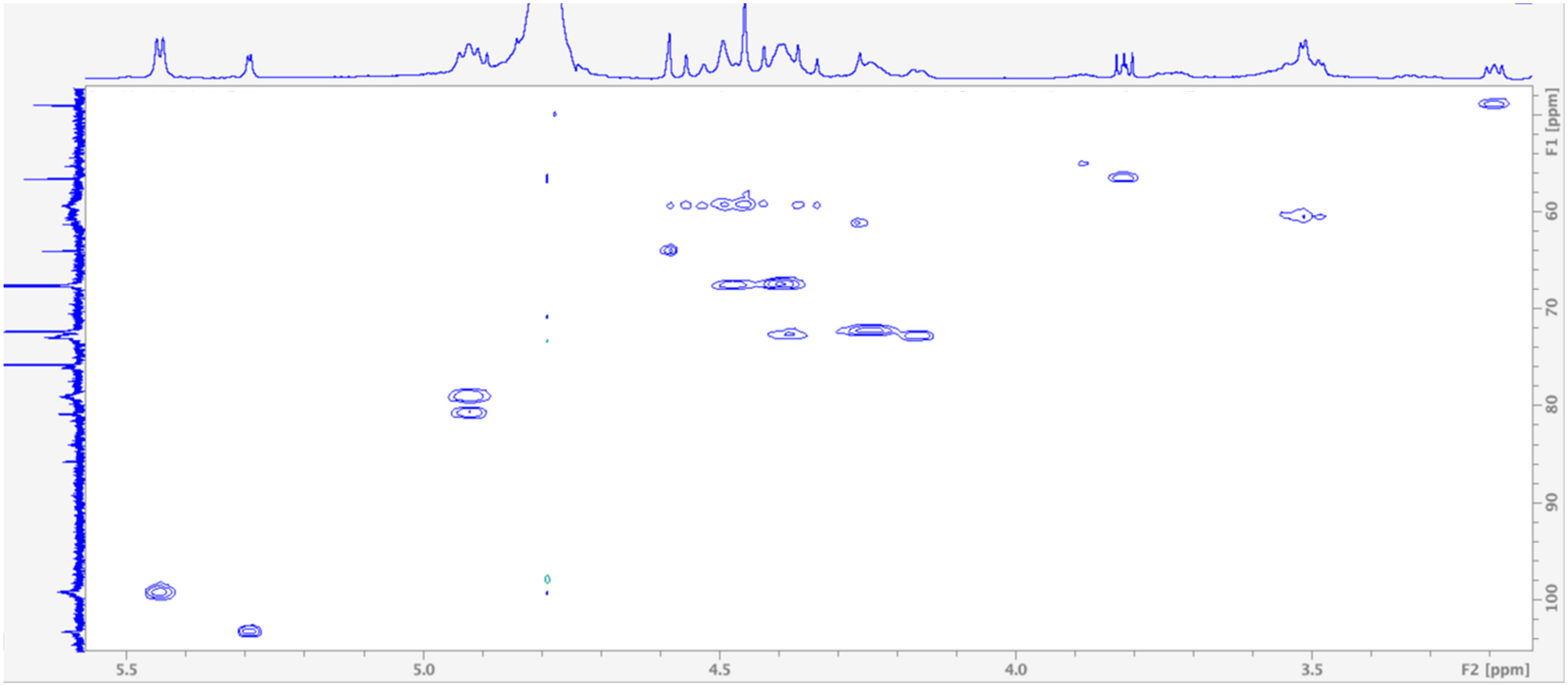

F1 [ppm]
60
70
80
90
100
5.5
5.0
4.5
4.0
3.5
F2 [ppm]

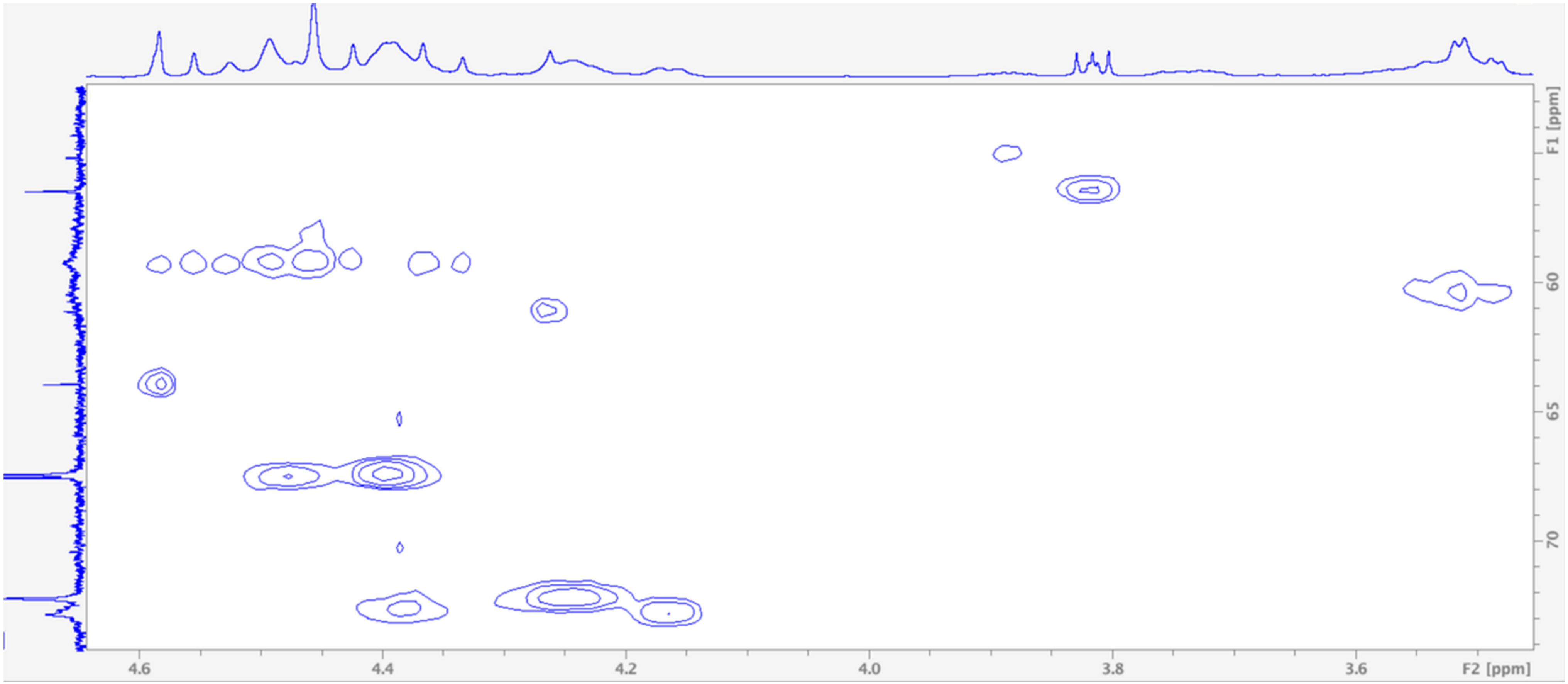

F1 [ppm]
60
65
70
4.6
4.4
4.2
4.0
3.8
3.6
F2 [ppm]

HMBC

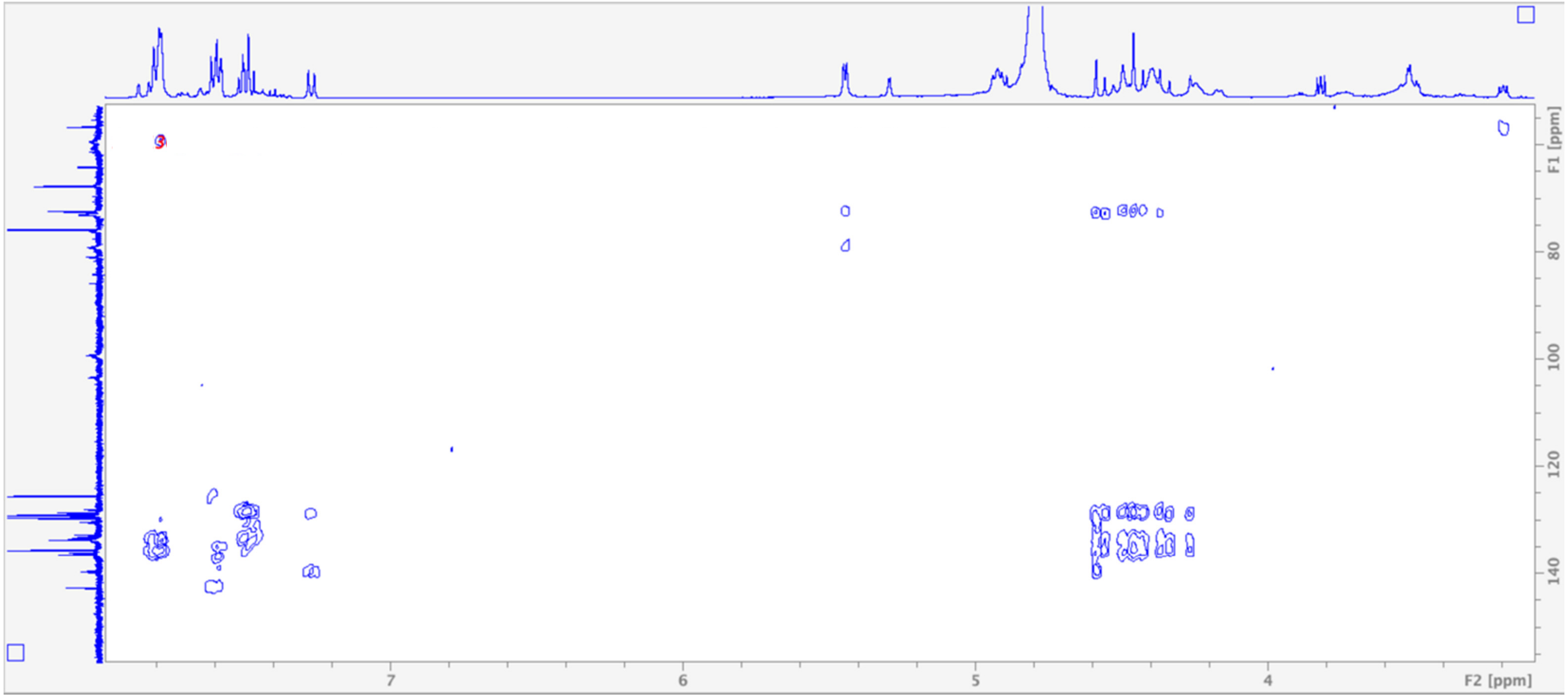

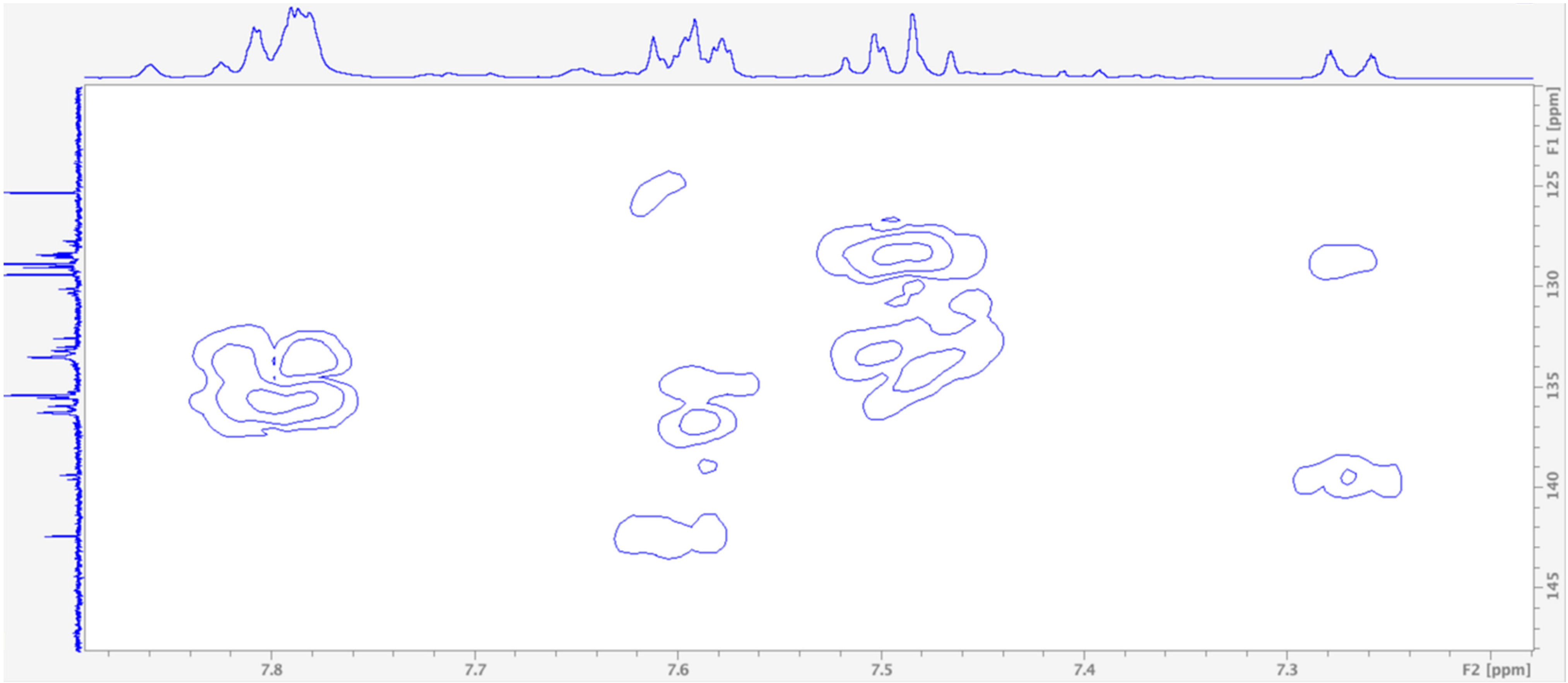
F1 [ppm]
125
130
135
140
145
7.8
7.7
7.6
7.5
7.4
7.3
F2 [ppm]

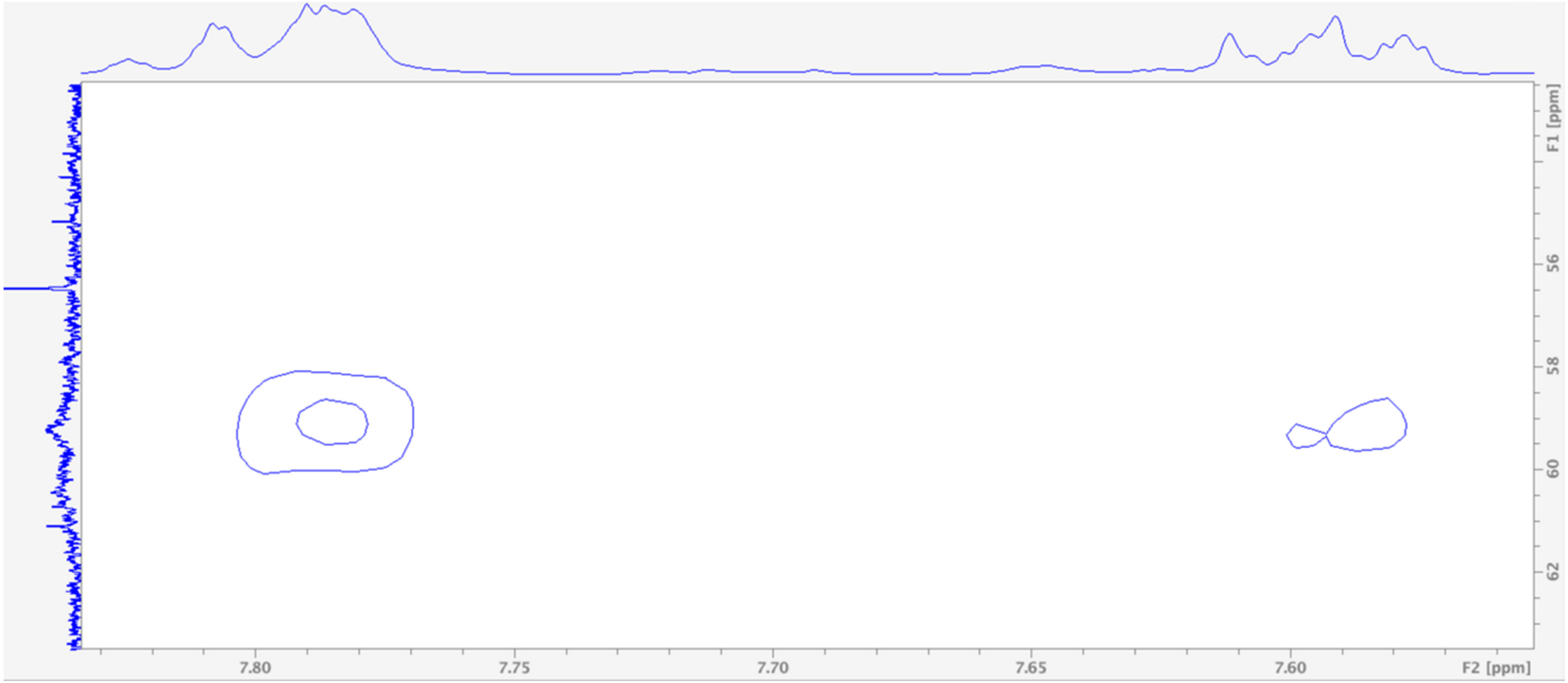

F1 [ppm]
56
58
60
62
7.80
7.75
7.70
7.65
7.60
F2 [ppm]

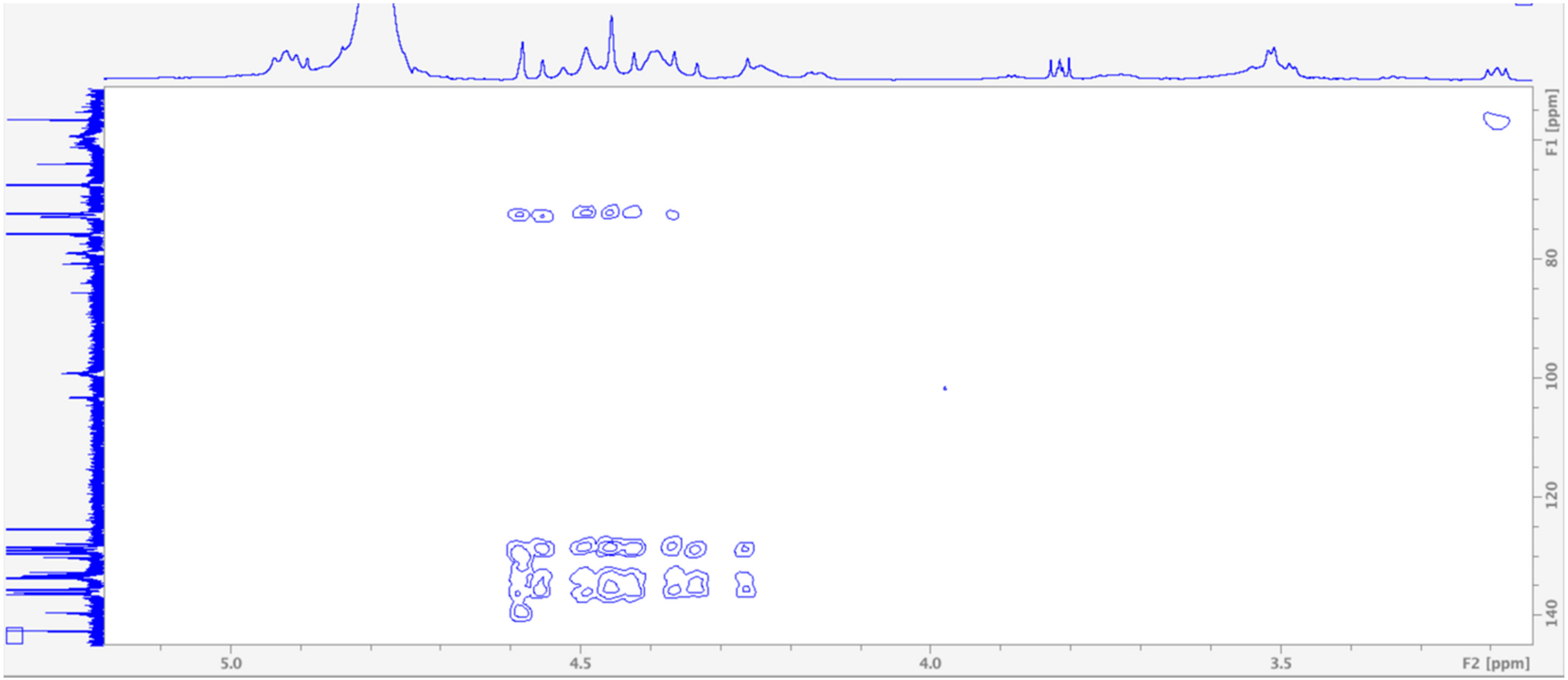

F1 [ppm]
80
100
120
140
5.0
4.5
4.0
3.5
F2 [ppm]

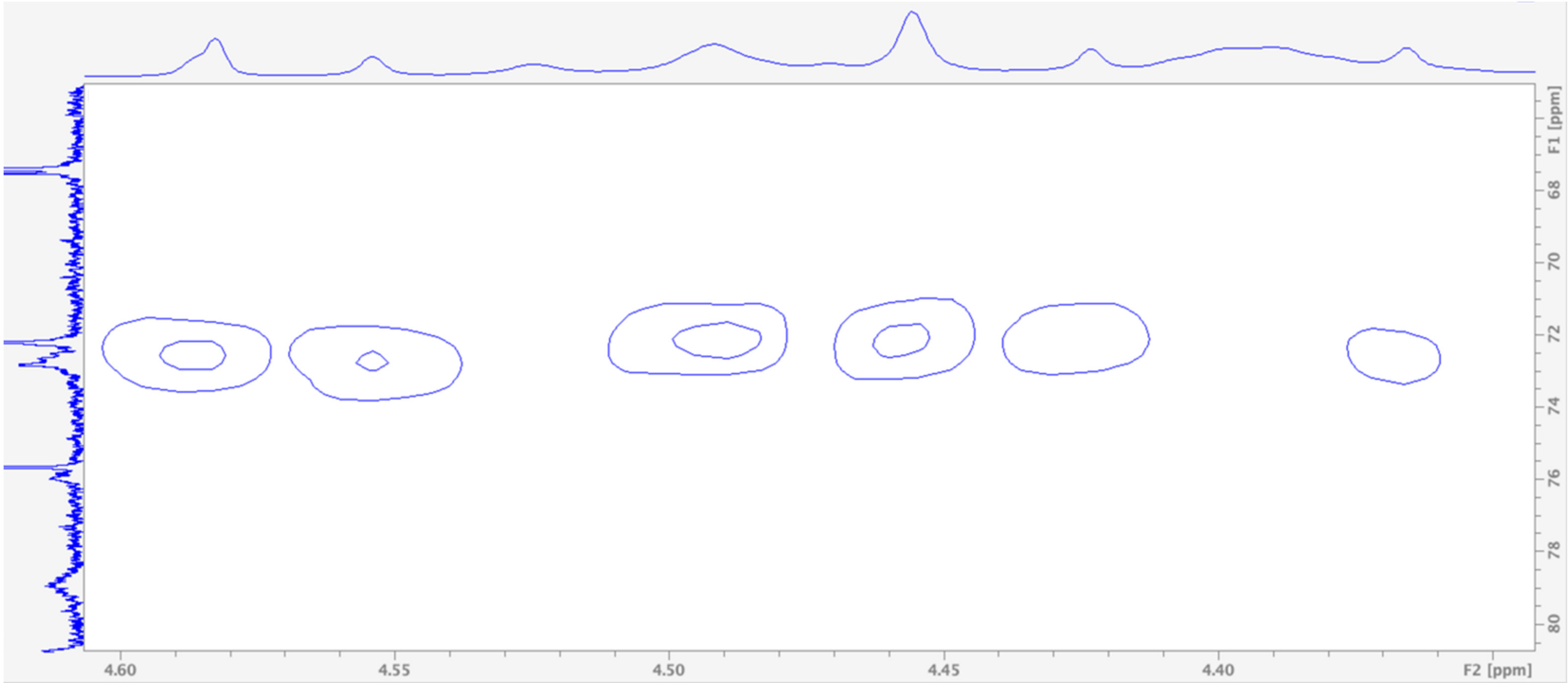

F1 [ppm]
68
70
72
74
76
78
80
4.60
4.55
4.50
4.45
4.40
F2 [ppm]

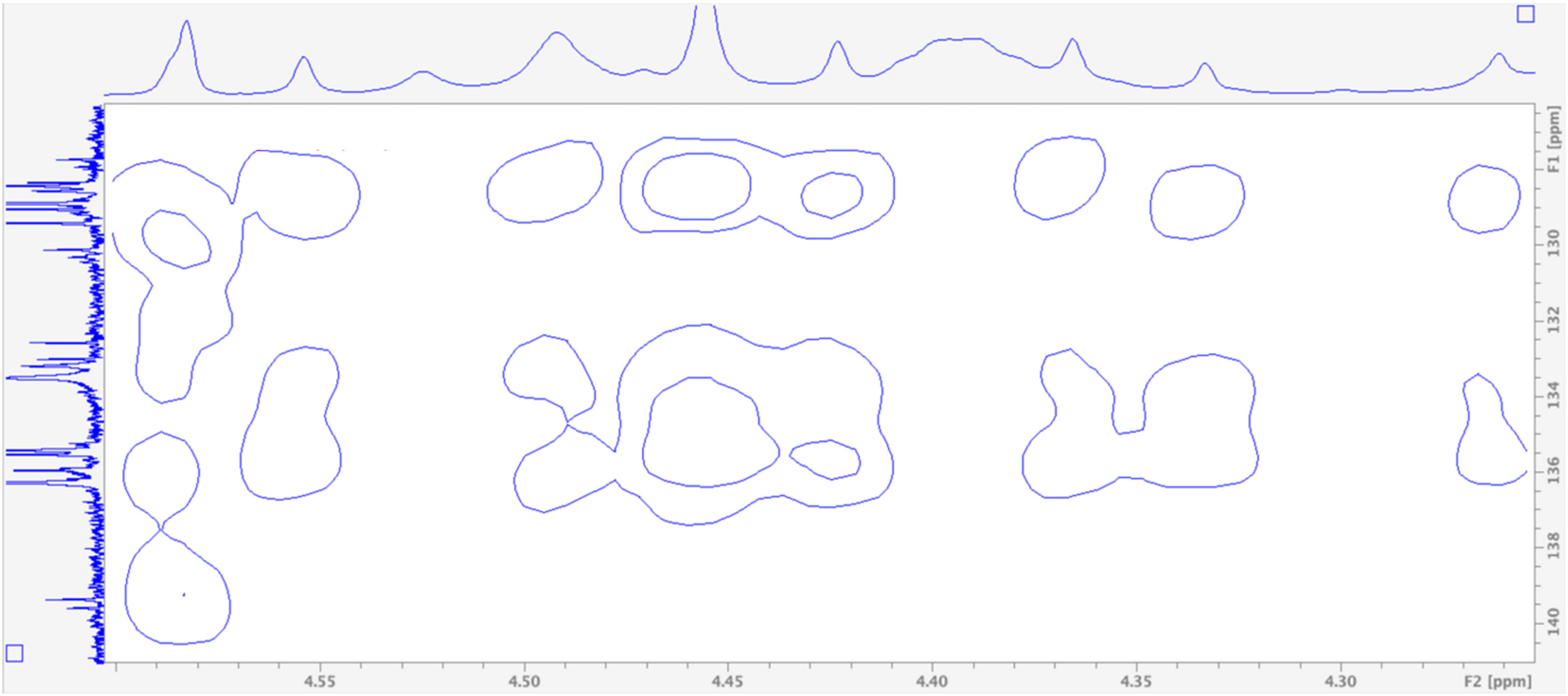

F1 [ppm]
130
132
134
136
138
140
4.55
4.50
4.45
4.40
4.35
4.30
F2 [ppm]

Figure S3. ^{1}H-NMR spectrum (400 MHz, D_2O) of intermediate **meta 3** with colour-coded signals, highlighting the furanose anomeric forms they belong to, with interpretation of the isolated signals and tentative interpretation of the overlapping ones. Namely, the orange designates the *α-fur* form and indigo designates the *β-fur* form. A) section 7.87 ppm to 7.20 ppm; B) section 5.50 ppm to 4.87 ppm; C) section 4.61 ppm to 4.12 ppm; D) section 4.00 ppm to 3.15 ppm. Highlighted are also the principal COSY correlations to hydrogen atoms within the same spin systems.

A

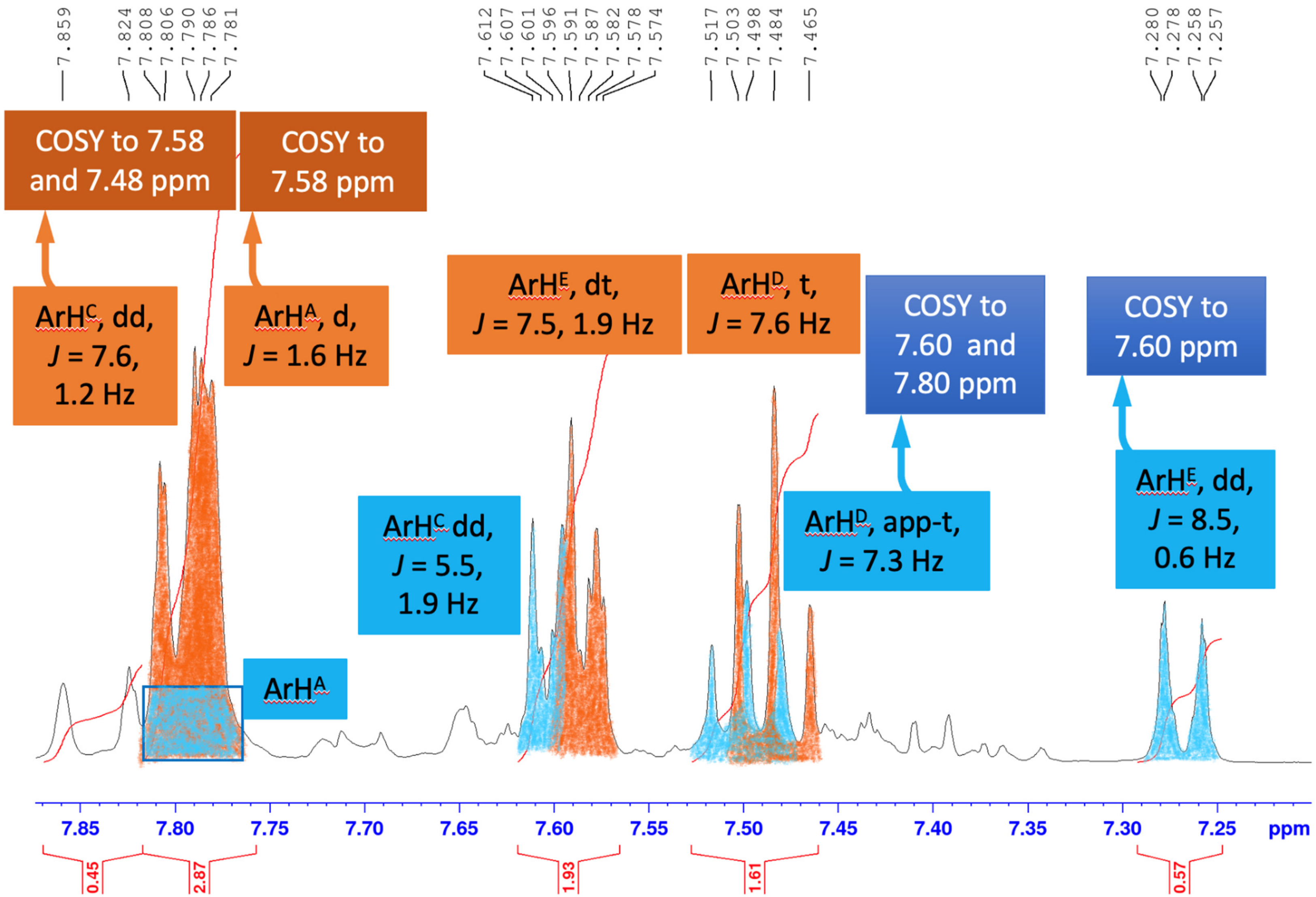

B

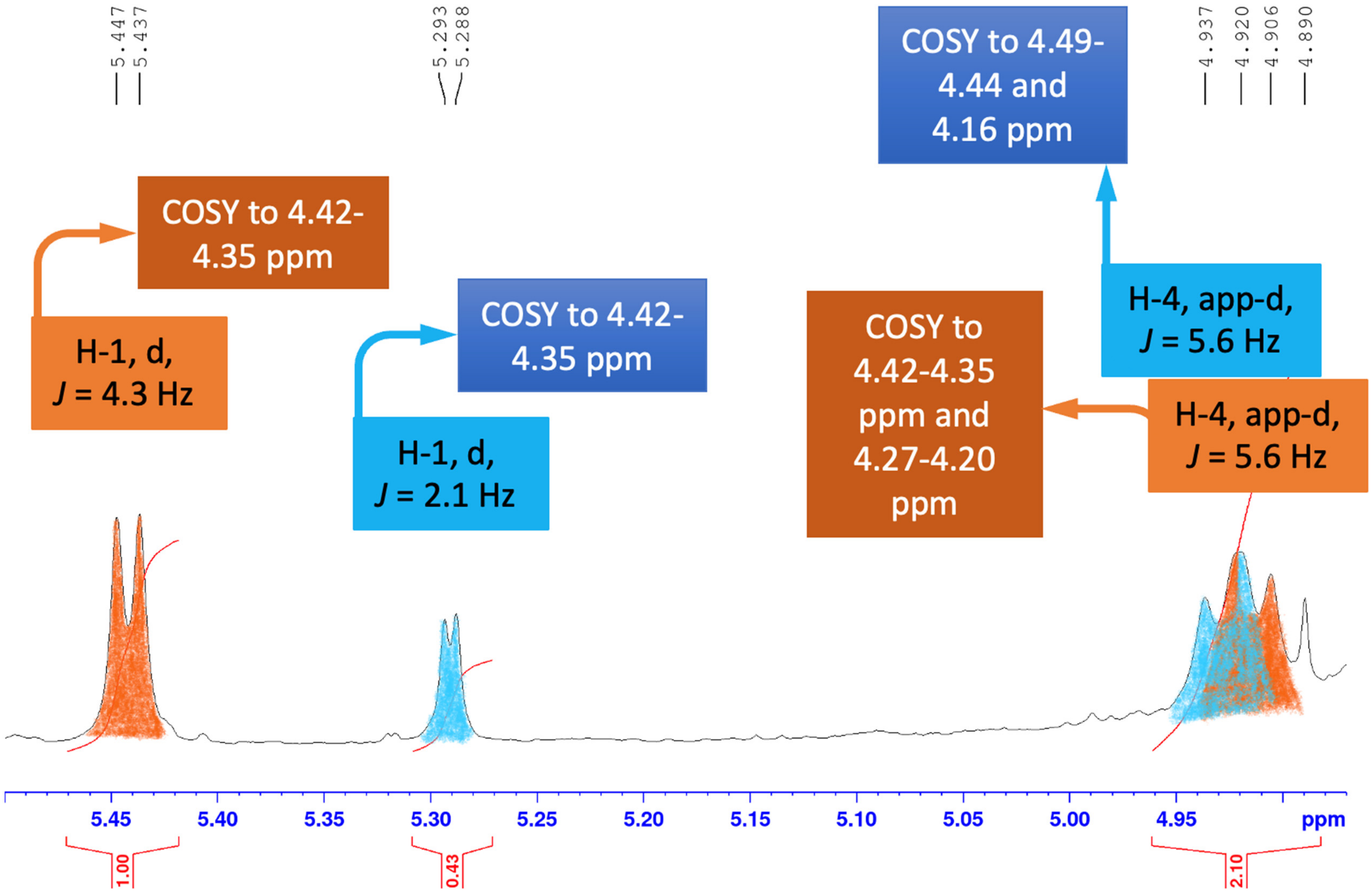
5.447
5.437
5.293
5.288
4.937
4.920
4.906
4.890
COSY to 4.49-4.44 and 4.16 ppm
COSY to 4.42-4.35 ppm
H-1, d, J = 4.3 Hz
COSY to 4.42-4.35 ppm
H-1, d, J = 2.1 Hz
COSY to 4.42-4.35 ppm and 4.27-4.20 ppm
H-4, app-d, J = 5.6 Hz
H-4, app-d, J = 5.6 Hz
5.45
5.40
5.35
5.30
5.25
5.20
5.15
5.10
5.05
5.00
4.95
ppm
1.00
0.43
2.10

c

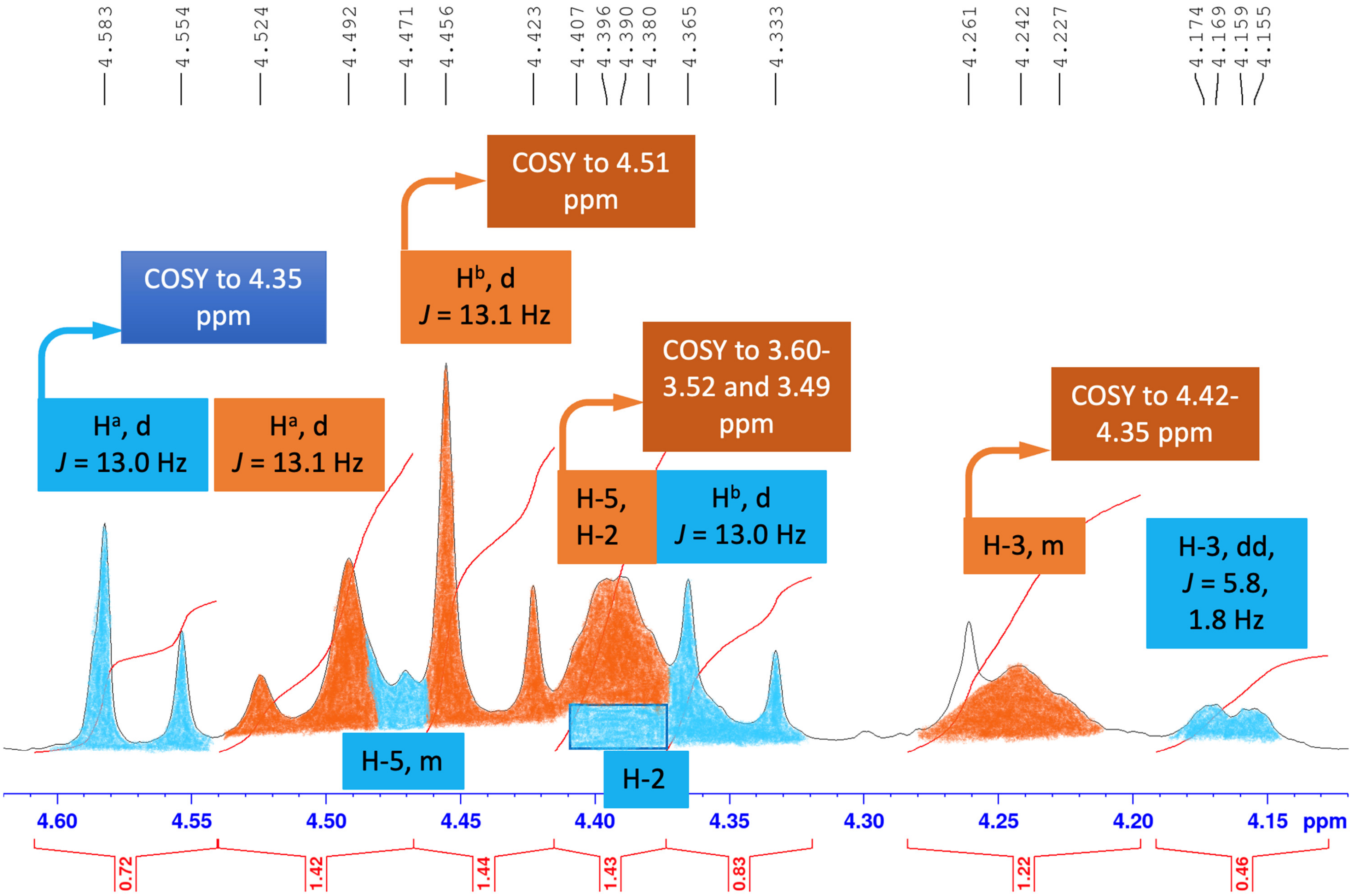
4.583
4.554
4.524
4.492
4.471
4.456
4.423
4.407
4.396
4.390
4.380
4.365
4.333
4.261
4.242
4.227
4.174
4.169
4.159
4.155
COSY to 4.51 ppm
COSY to 4.35 ppm
H^{b}, d
J = 13.1 Hz
COSY to 3.60-3.52 and 3.49 ppm
H^{a}, d
J = 13.0 Hz
H^{a}, d
J = 13.1 Hz
H-5, H-2
H^{b}, d
J = 13.0 Hz
COSY to 4.42-4.35 ppm
H-3, m
H-3, dd, J = 5.8, 1.8 Hz
H-5, m
H-2
4.60
4.55
4.50
4.45
4.40
4.35
4.30
4.25
4.20
4.15
ppm
0.72
1.42
1.44
1.43
0.83
1.22
0.46

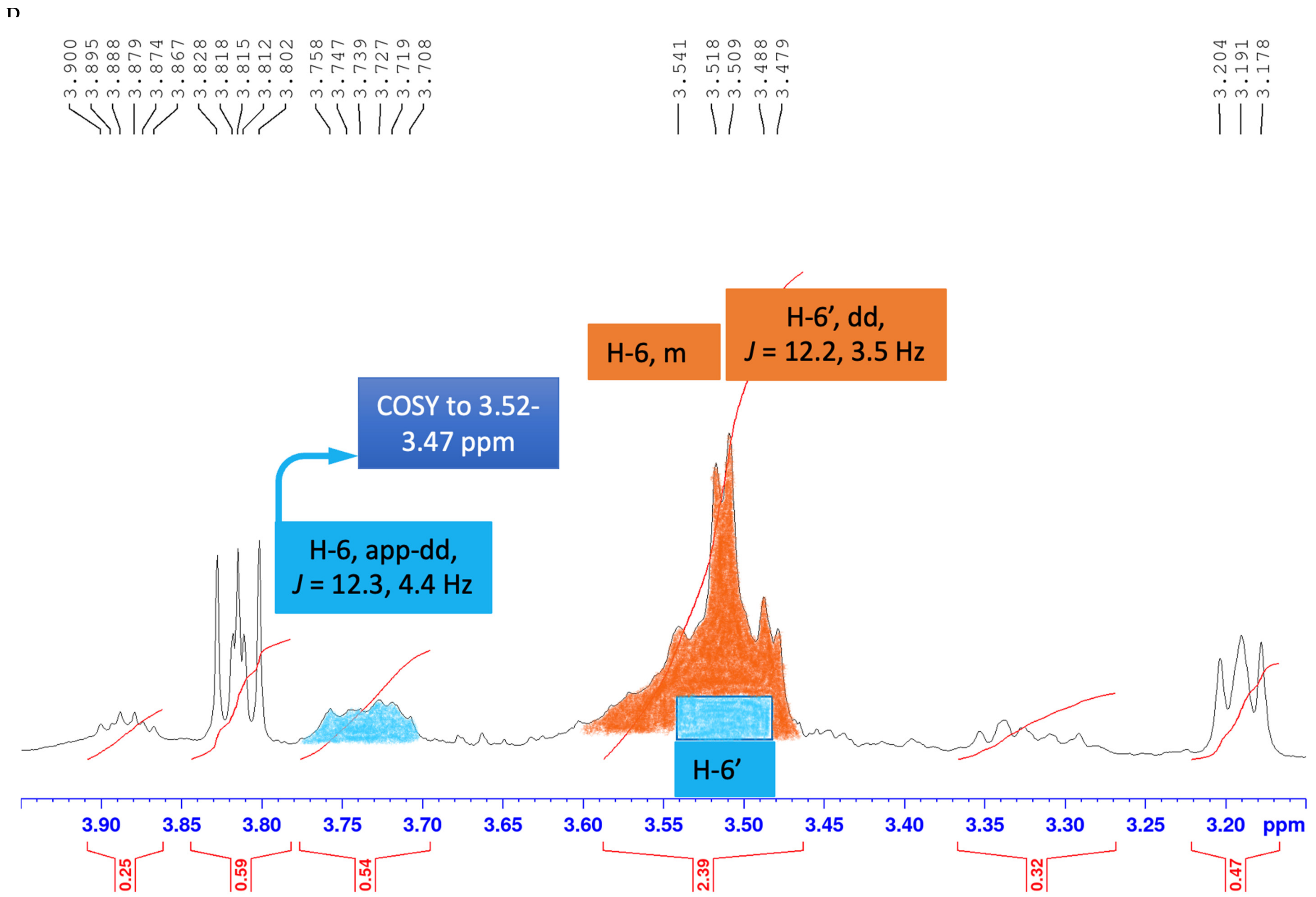
3.900
3.895
3.888
3.879
3.874
3.867
3.828
3.818
3.815
3.812
3.802
3.758
3.747
3.739
3.727
3.719
3.708
3.541
3.518
3.509
3.488
3.479
3.204
3.191
3.178
H-6, m
H-6', dd,
J = 12.2, 3.5 Hz
COSY to 3.52-3.47 ppm
H-6, app-dd,
J = 12.3, 4.4 Hz
H-6'
3.90 3.85 3.80 3.75 3.70 3.65 3.60 3.55 3.50 3.45 3.40 3.35 3.30 3.25 3.20 ppm
0.25
0.59
0.54
2.39
0.32
0.47

Figure S4. ^{13}C-NMR spectrum (100 MHz, D_2O) sections of intermediate **meta 3** with colour-coded signals, highlighting the furanose anomeric forms they belong to, with interpretation of the isolated signals and tentative interpretation of the overlapping ones. Namely, the orange designates the *α-fur* form and indigo designates the *β-fur* form. A) section 141.5 ppm to 127.2 ppm; B) section 104.0 ppm to 76.0 ppm; C) section 75.5 ppm to 56.7 ppm. Highlighted are also the principal HSQC and HMBC correlations to hydrogen atoms within the same spin systems.

A

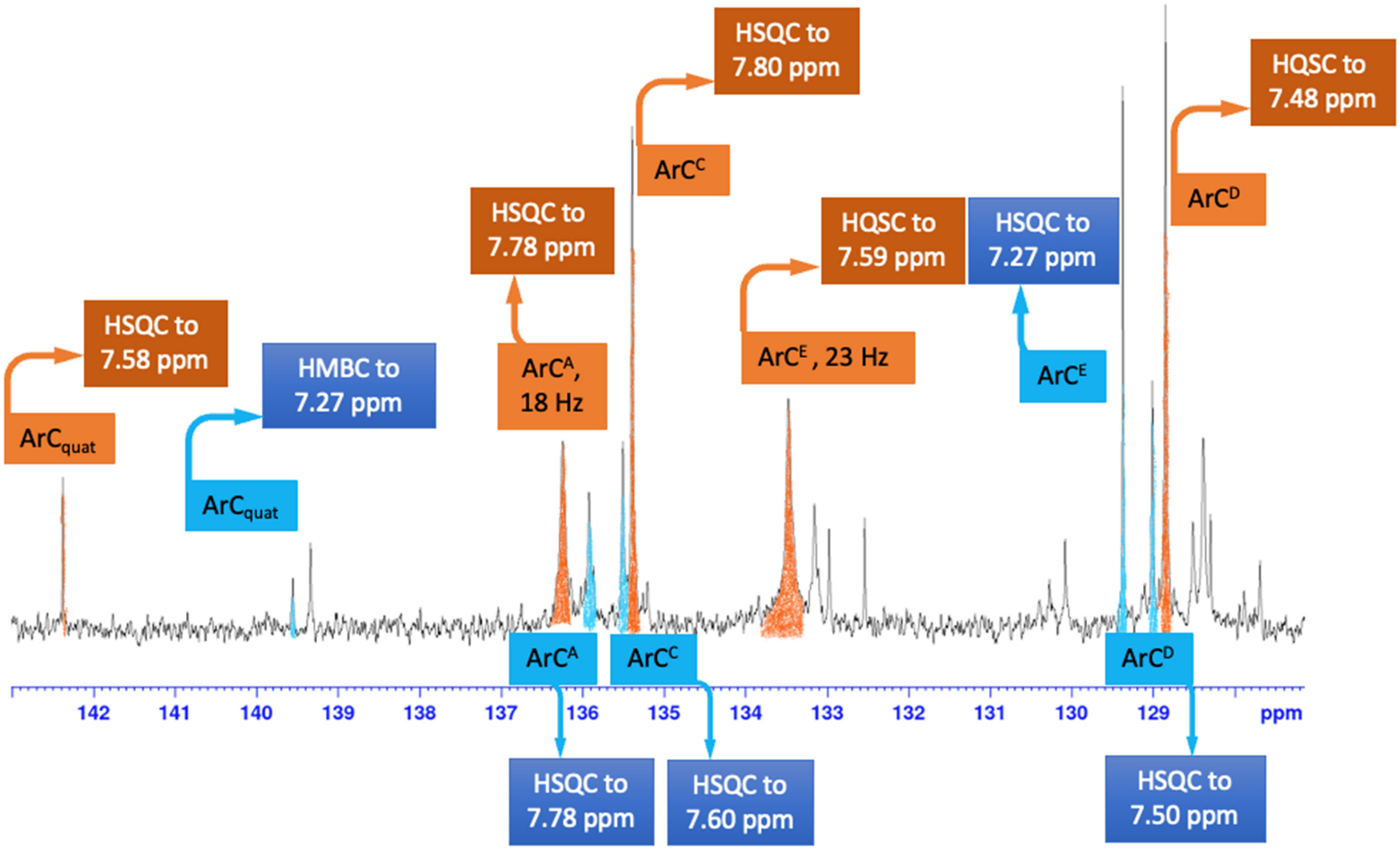

B

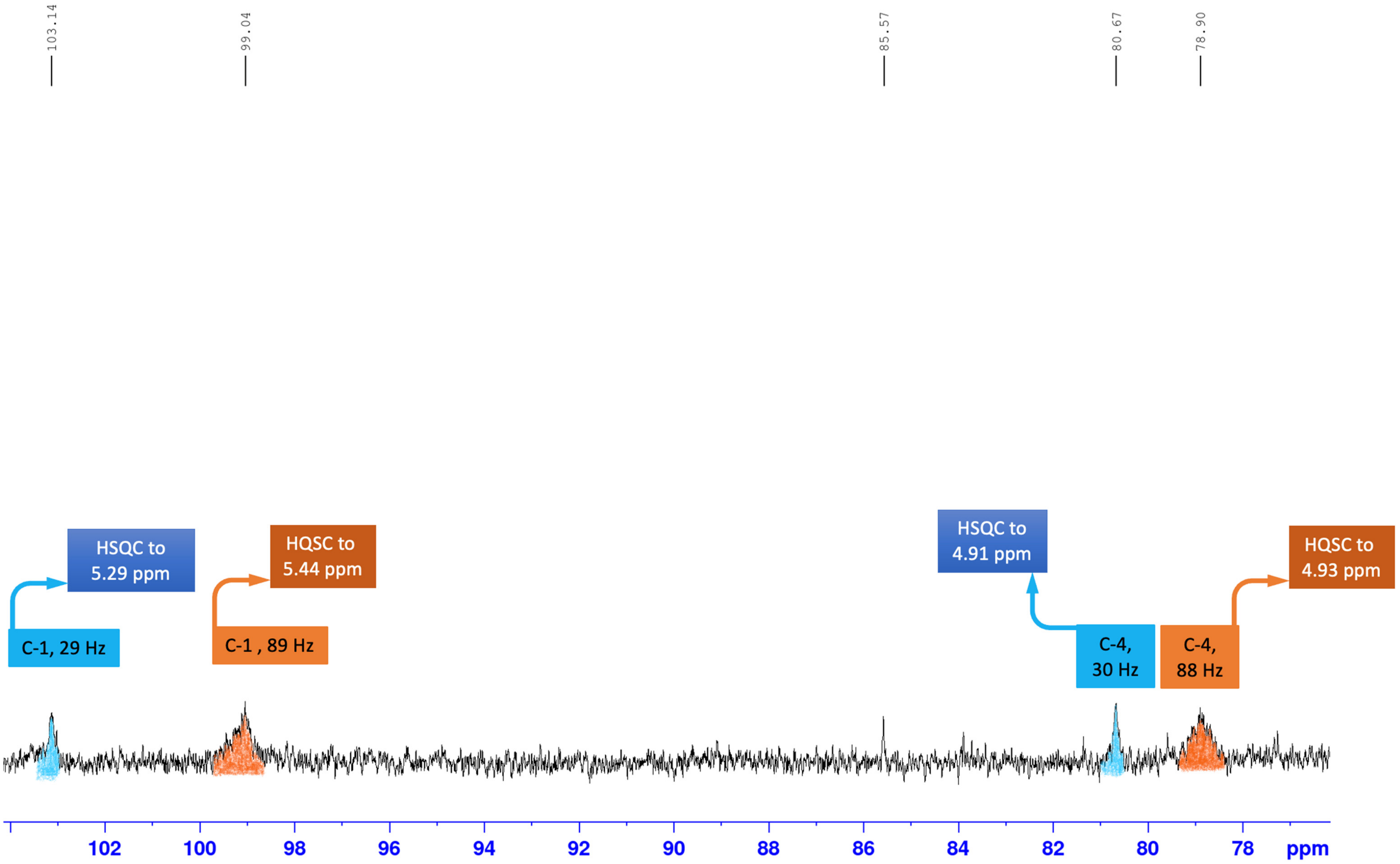

103.14
99.04
85.57
80.67
78.90
HSQC to
5.29 ppm
HQSC to
5.44 ppm
HSQC to
4.91 ppm
HQSC to
4.93 ppm
C-1, 29 Hz
C-1 , 89 Hz
C-4,
30 Hz
C-4,
88 Hz
102
100
98
96
94
92
90
88
86
84
82
80
78
ppm

C

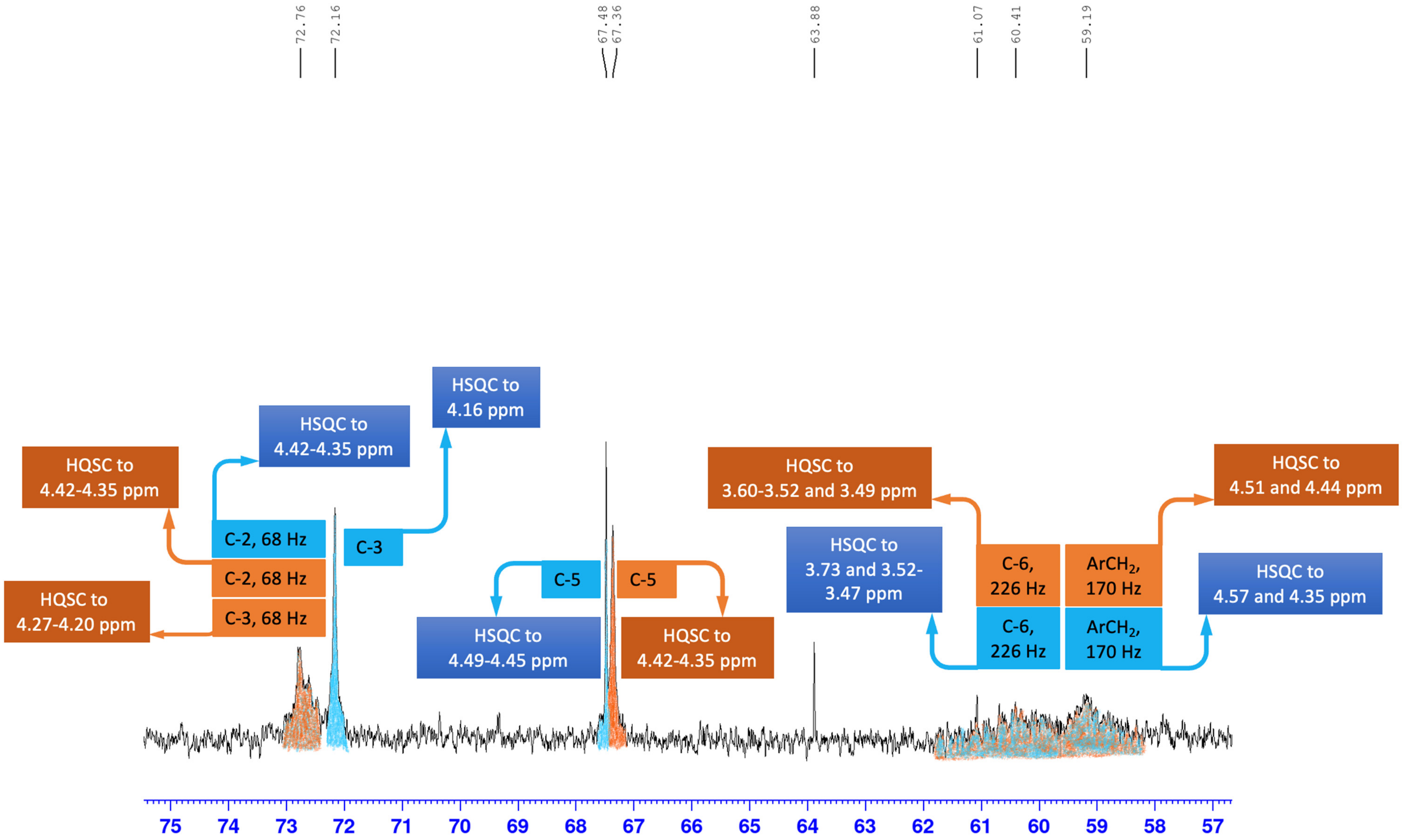

72.76
72.16
67.48
67.36
63.88
61.07
60.41
59.19
HSQC to 4.16 ppm
HSQC to 4.42-4.35 ppm
HQSC to 4.42-4.35 ppm
C-2, 68 Hz
C-3
C-2, 68 Hz
C-3, 68 Hz
HQSC to 4.27-4.20 ppm
C-5
C-5
HSQC to 4.49-4.45 ppm
HQSC to 4.42-4.35 ppm
HQSC to 3.60-3.52 and 3.49 ppm
HSQC to 3.73 and 3.52-3.47 ppm
C-6, 226 Hz
C-6, 226 Hz
ArCH$_2$, 170 Hz
ArCH$_2$, 170 Hz
HQSC to 4.51 and 4.44 ppm
HSQC to 4.57 and 4.35 ppm
75 74 73 72 71 70 69 68 67 66 65 64 63 62 61 60 59 58 57

Figure S5. ^{1}H- (400 MHz), ^{13}C-NMR (100 MHz), DEPT, ^{11}B-NMR (128 MHz), COSY and HSQC spectra of *N*-(3-methylphenyl boronic acid)-1,4-dideoxy-1,4-imino-L-gulitol **meta 4** in D_2O.

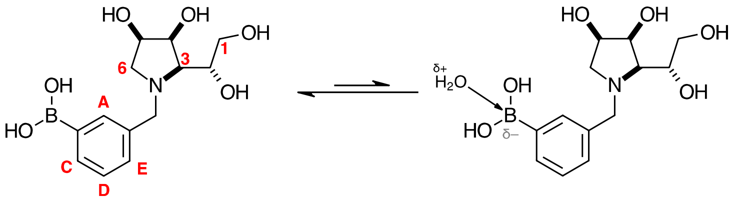

Boronic acid species. δ_H: 7.72 (1H, s, ArH^A), 7.66 (1H, d, $J_{HC,HD}$ 7.4 Hz, ArH^C), 7.43 (1H, d, $J_{HE,HD}$ 7.5 Hz, ArH^E), 7.27 (1H, app-t, $J_{HD,HC/HE}$ 7.3 Hz, ArH^D), 4.55 (1H, d, $J_{Ha,Hb}$ 12.9 Hz, ArCH^aH^b), 4.26-4.05 (m, H-2, H-3, H-5), 3.99 (1H, d, $J_{Hb,Ha}$ 12.9 Hz, ArCH^aH^b), 3.63-3.53 (m, H-6, H-4), 3.52 (1H, app-t, *J* 3.5 Hz, H-6'), 3.31-3.20 (m, H-1), 3.09-2.97 (m, H-1'); δ_C: 137.6 (ArC^A), 136.5 (ArC^C), 134.2 (ArC^E), 129.6 (ArC^D), 72.18 (C-3), 71.63 (C-2), 70.5 (C-4), 70.12 (C-5), 64.7 (C-6), 62.5 ($ArCH_2$,width 21 Hz), 54.3 (C-1, width 27 Hz). The two C_{quat} are not discernible.
Boronate species. δ_H: 7.10 (1H, app-t, $J_{HD,HC/HE}$ 8.0 Hz, ArH^D), 6.83 (1H, d, $J_{HC,HD}$ 8.0 Hz, ArH^C), 6.81 (1H, s, H^A), 6.71 (1H, d, $J_{HE,HD}$ 8.1 Hz, ArH^E), 4.64 (1H, d, $J_{Ha,Hb}$ 12.9 Hz, ArCH^aH^b), 4.08 (1H, obscured, ArCH^aH^b), 3.67-3.62 (m, H-1), 3.63-3.53 (m, H-6, H-4), 3.49 (1H, app-t, *J* 3.5 Hz, H-6'), 3.09-2.97 (m, H-1'). δ_C: 131.5 (ArC^D), 123.3 (ArC^E), 118.8 (ArC^A), 118.1 (ArC^C), 72.17 (C-3), 71.62 (C-2), 70.5 (C-4), 70.10 (C-5), 64.6 (C-6), 62.7 ($ArCH_2$), 57.8 (C-1, width 17 Hz). The two C_{quat} are not discernible.
δ_B: 27.8 (broad, integration: 1.7), 19.3 (sharp, integration: 1.0).

^{1}H-NMR

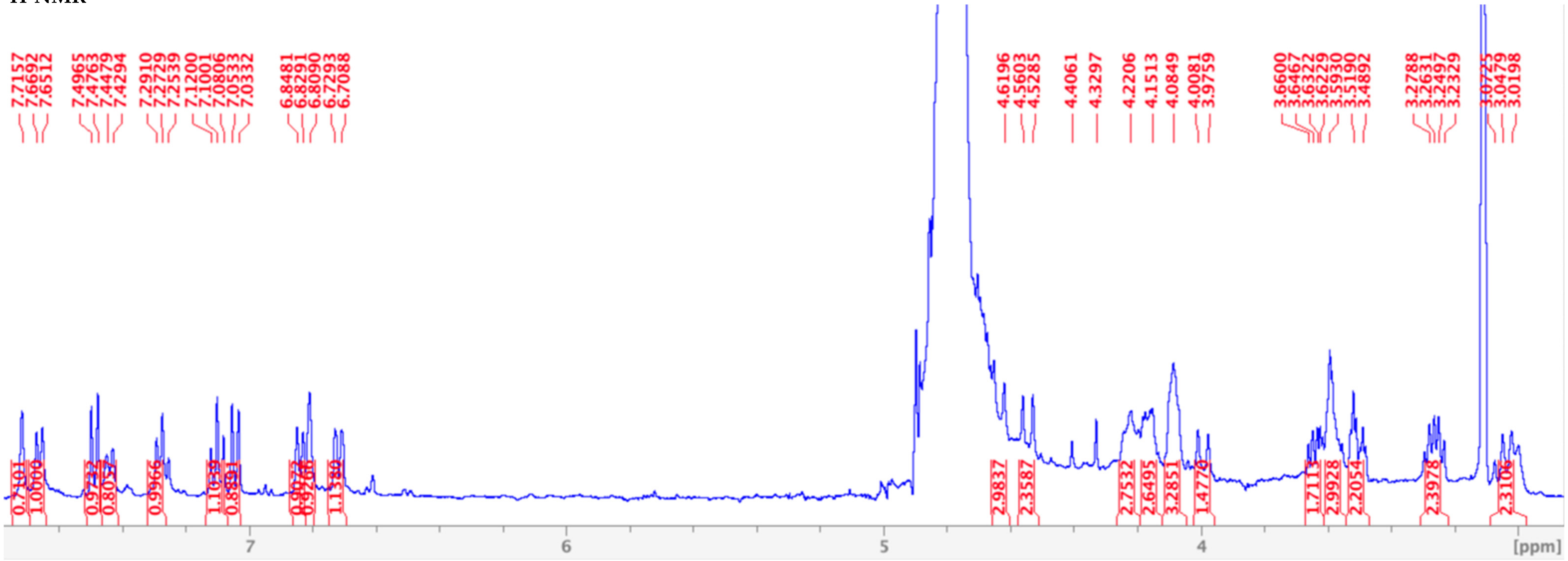

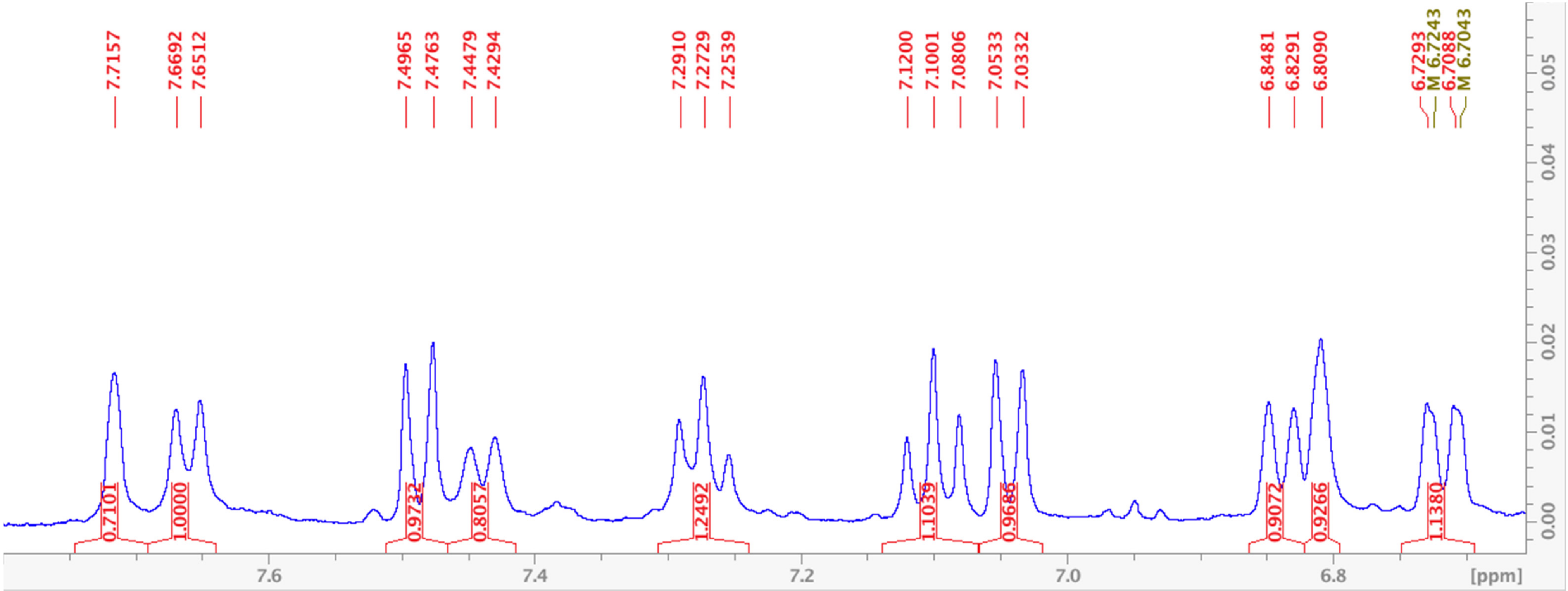
7.7157
7.6692
7.6512
7.4965
7.4763
7.4479
7.4294
7.2910
7.2729
7.2539
7.1200
7.1001
7.0806
7.0533
7.0332
6.8481
6.8291
6.8090
6.7293
M 6.7243
6.7088
M 6.7043
0.7101
1.0000
0.9732
0.8057
1.2492
1.1039
0.9686
0.9072
0.9266
1.1380
7.6
7.4
7.2
7.0
6.8
[ppm]
0.05
0.04
0.03
0.02
0.01
0.00

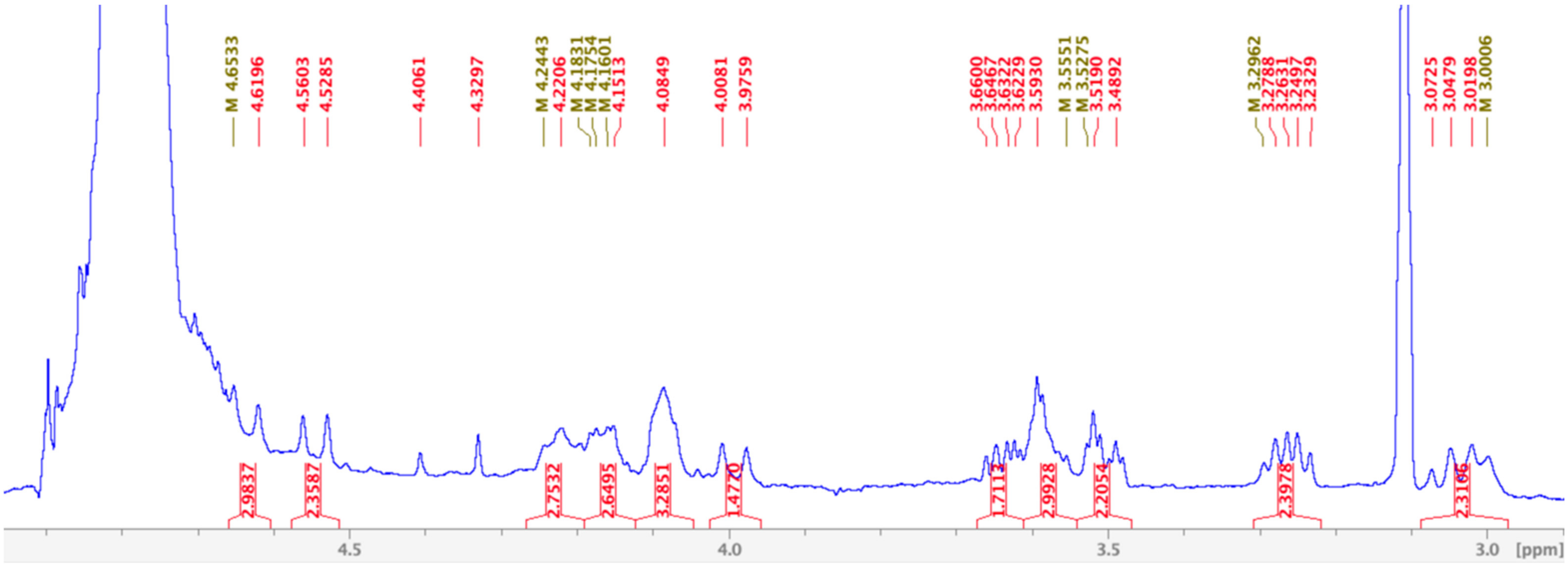
M 4.6533
4.6196
4.5603
4.5285
4.4061
4.3297
M 4.2443
4.2206
M 4.1831
M 4.1754
M 4.1601
4.1513
4.0849
4.0081
3.9759
3.6600
3.6467
3.6322
3.6229
3.5930
M 3.5551
M 3.5275
3.5190
3.4892
M 3.2962
3.2788
3.2631
3.2497
3.2329
3.0725
3.0479
3.0198
M 3.0006
2.9837
2.3587
2.7532
2.6495
3.2851
1.4770
1.7113
2.9928
2.2054
2.3978
2.3166
4.5
4.0
3.5
3.0
[ppm]

^{13}C-NMR

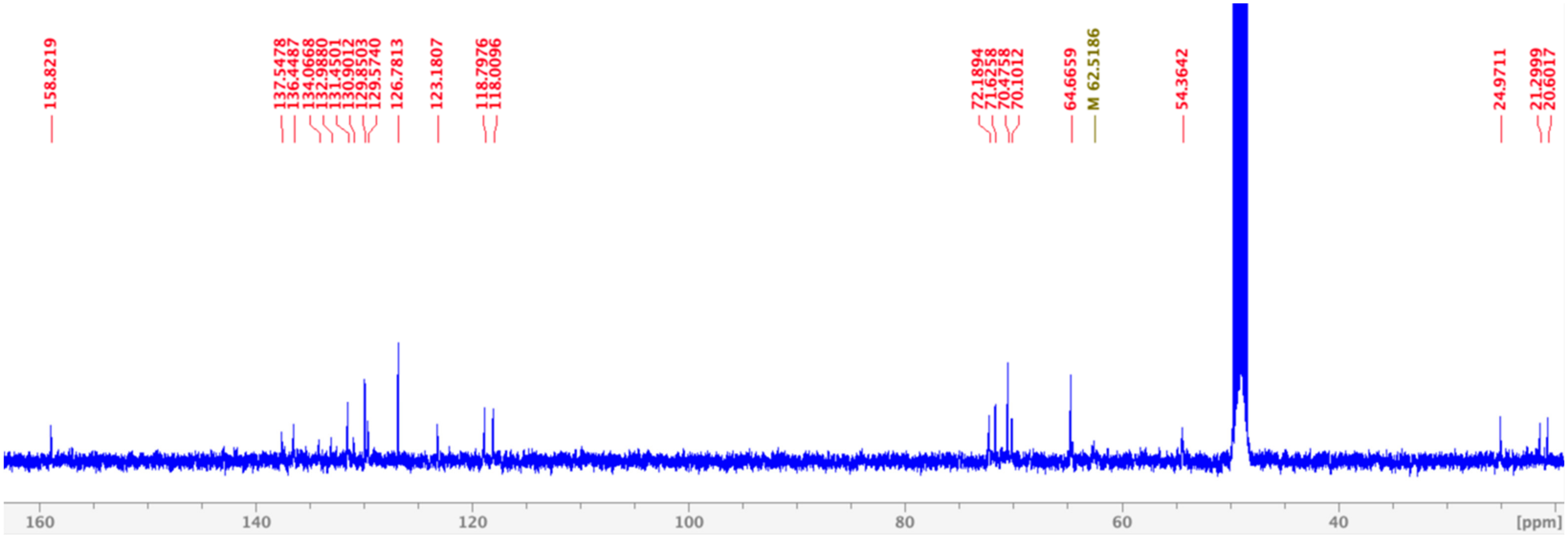

158.8219
137.5478
136.4487
134.0668
132.9880
131.4501
130.9012
129.8503
129.5740
126.7813
123.1807
118.7976
118.0096
72.1894
71.6258
70.4758
70.1012
64.6659
M 62.5186
54.3642
24.9711
21.2999
20.6017
160
140
120
100
80
60
40
[ppm]

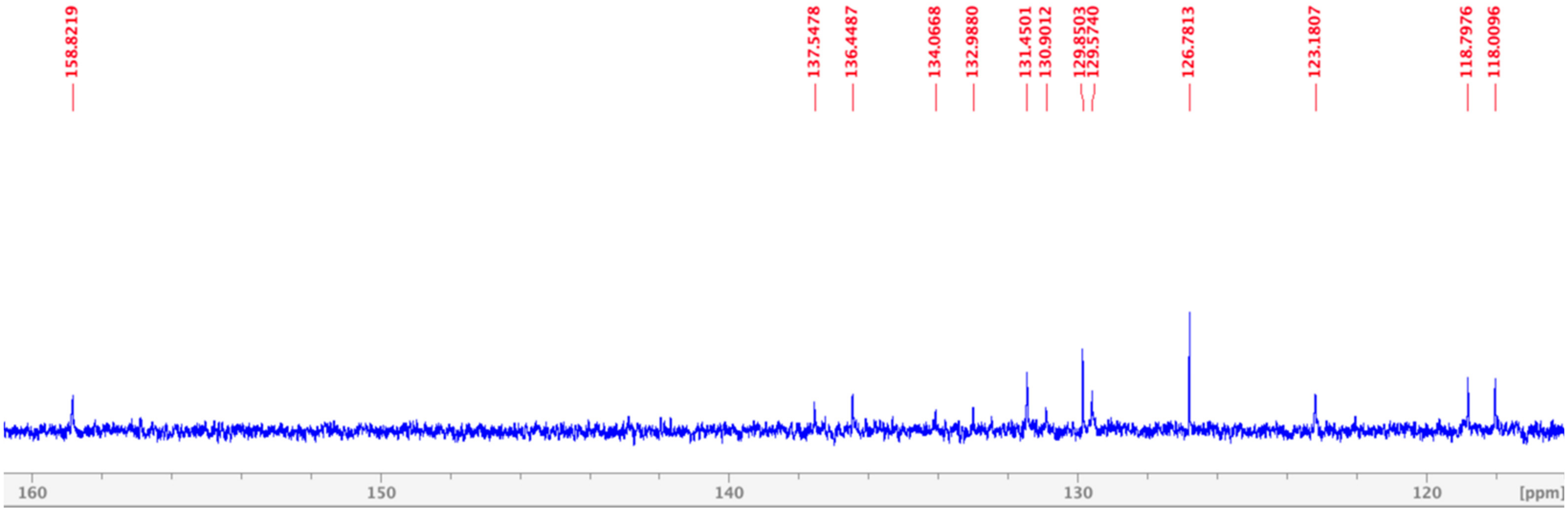
158.8219
137.5478
136.4487
134.0668
132.9880
131.4501
130.9012
129.8503
129.5740
126.7813
123.1807
118.7976
118.0096
160
150
140
130
120
[ppm]

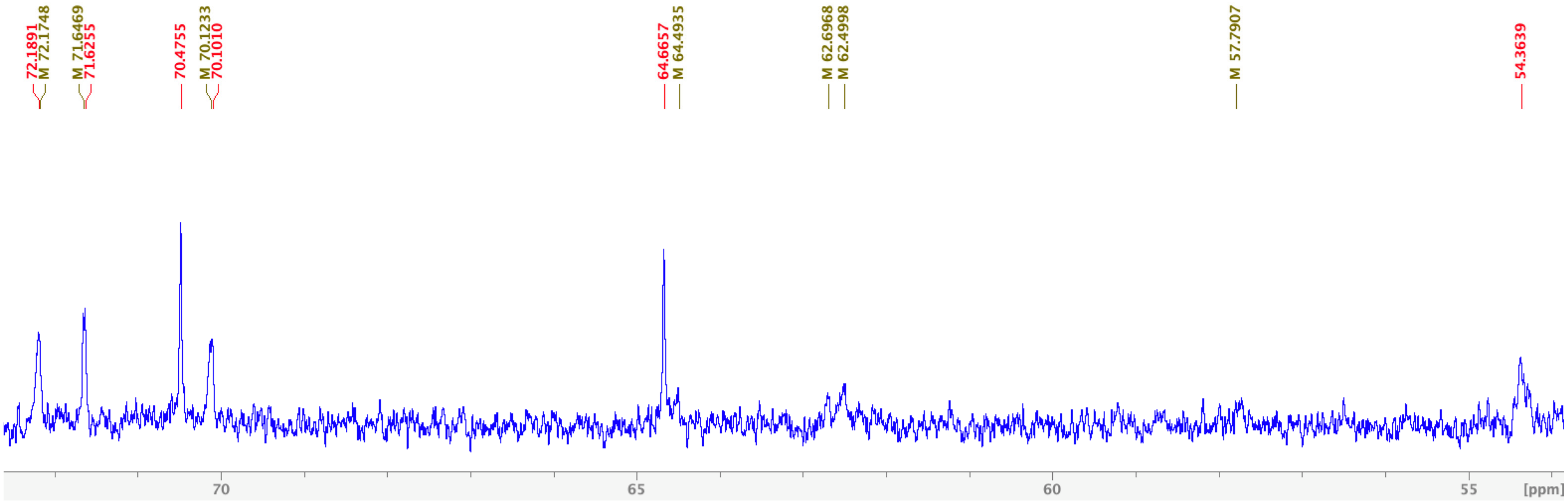
72.1891
M 72.1748
M 71.6469
71.6255
70.4755
M 70.1233
70.1010
64.6657
M 64.4935
M 62.6968
M 62.4998
M 57.7907
54.3639
70
65
60
55
[ppm]

DEPT

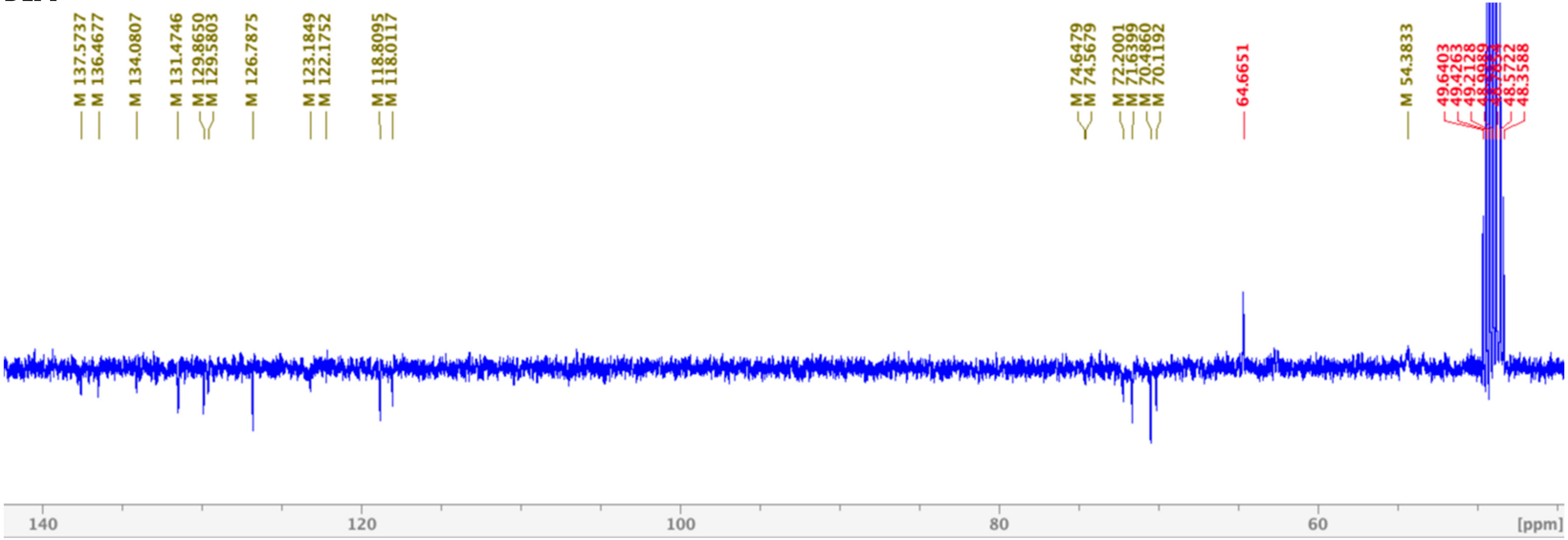
M 137.5737
M 136.4677
M 134.0807
M 131.4746
M 129.8650
M 129.5803
M 126.7875
M 123.1849
M 122.1752
M 118.8095
M 118.0117
M 74.6479
M 74.5679
M 72.2001
M 71.6399
M 70.4860
M 70.1192
64.6651
M 54.3833
49.6403
49.4263
49.2128
48.5722
48.3588
140
120
100
80
60
[ppm]

^{11}B-NMR

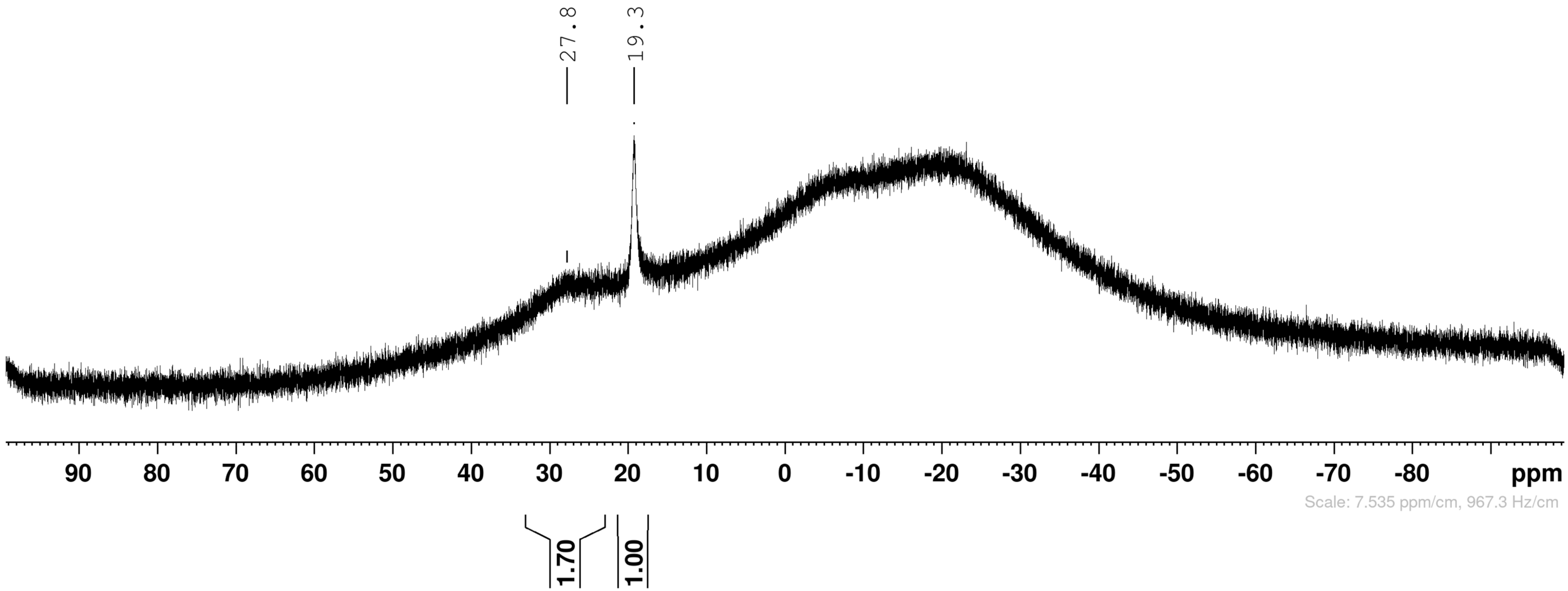

27.8
19.3
90
80
70
60
50
40
30
20
10
0
-10
-20
-30
-40
-50
-60
-70
-80
ppm
Scale: 7.535 ppm/cm, 967.3 Hz/cm
1.70
1.00

COSY

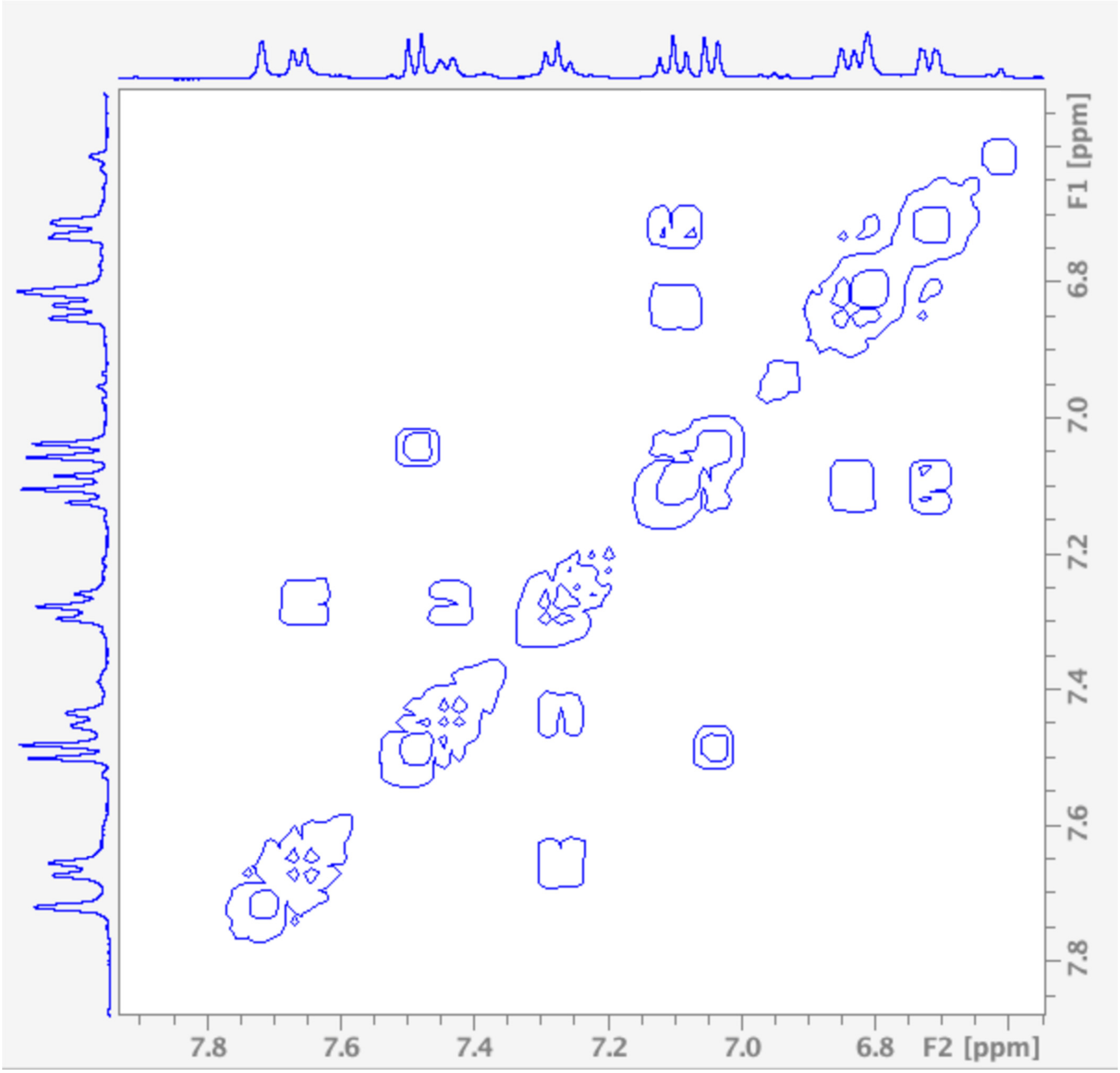

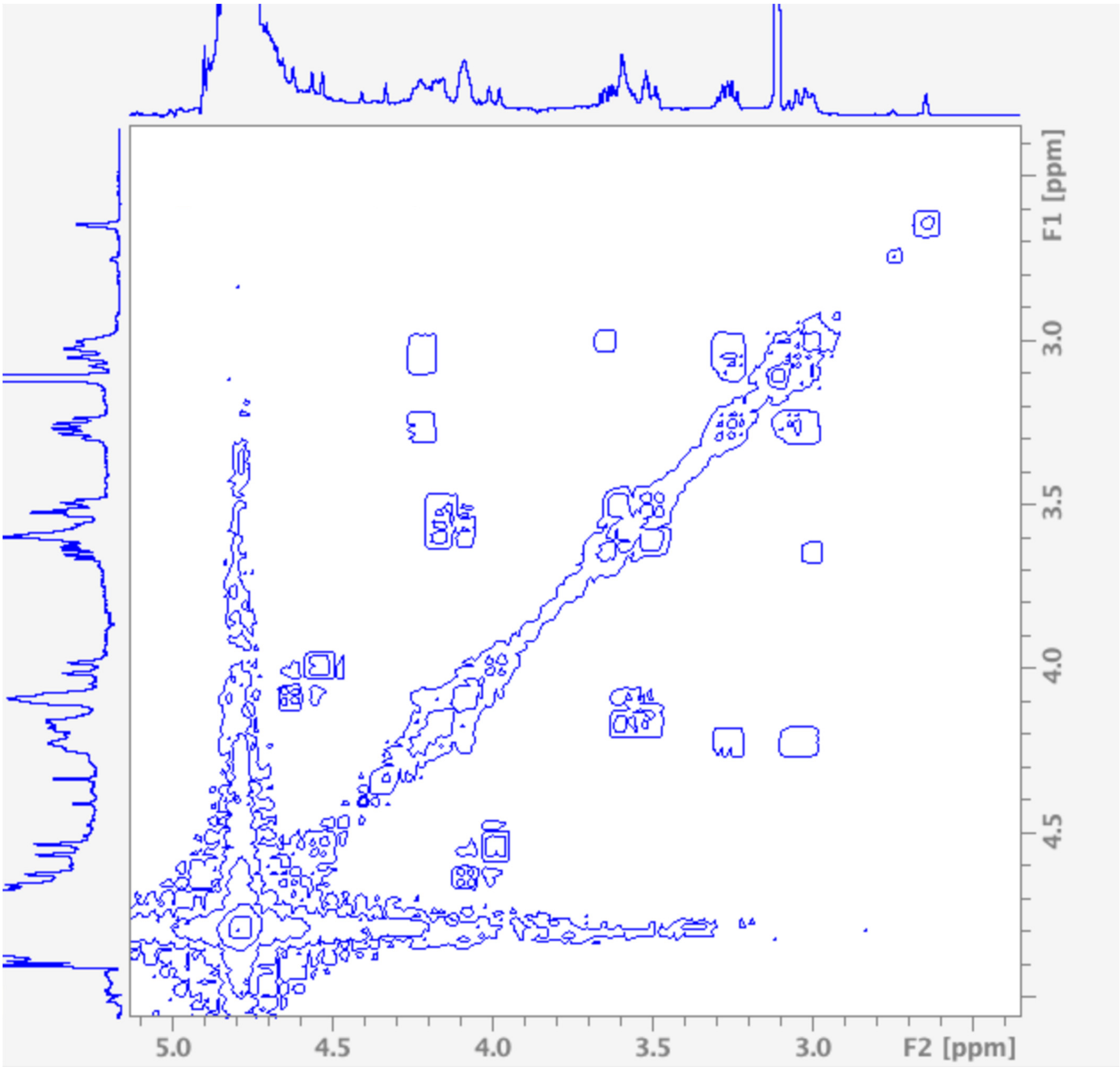
F1 [ppm]
3.0
3.5
4.0
4.5
5.0
4.5
4.0
3.5
3.0
F2 [ppm]

HSQC

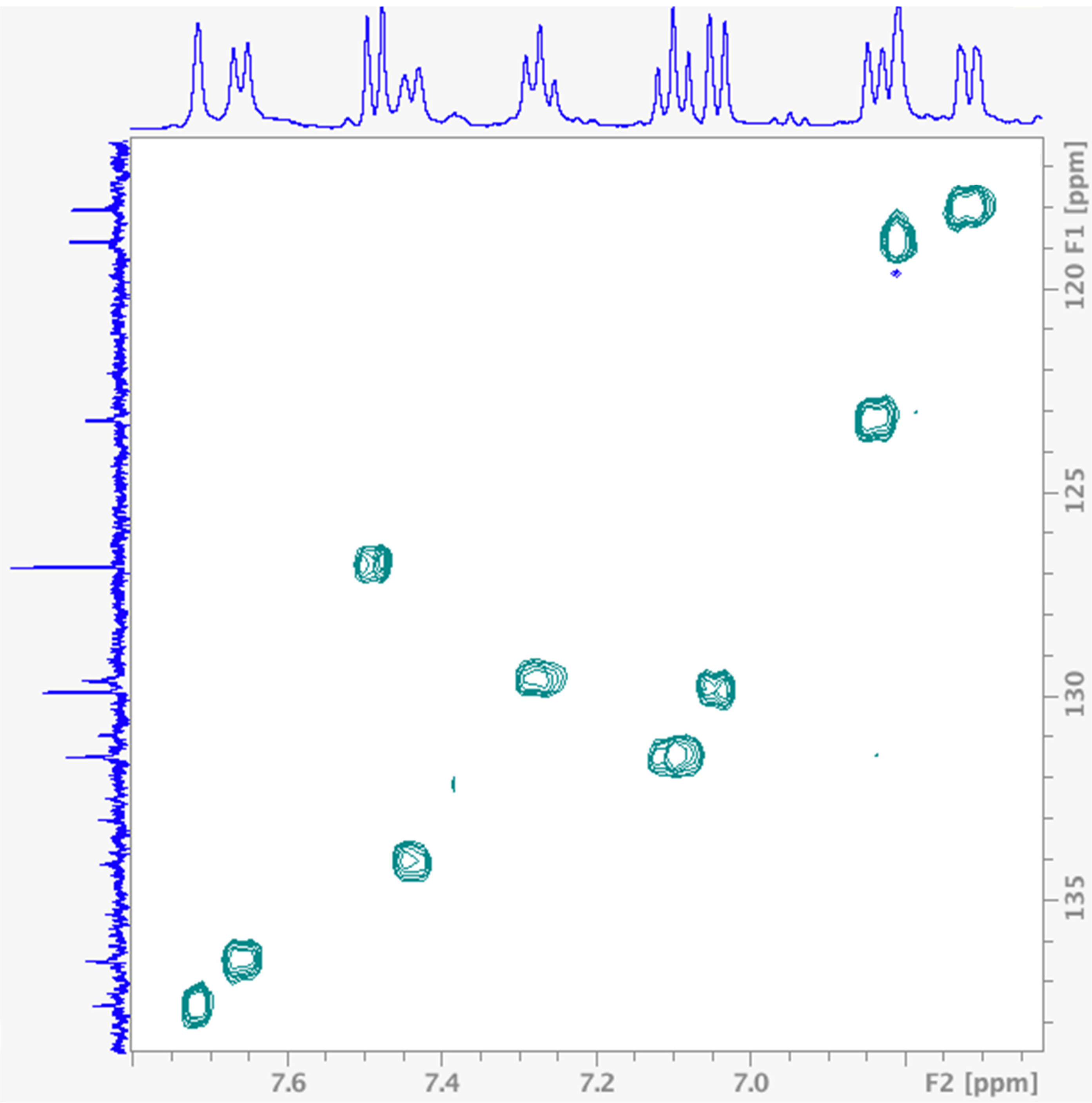

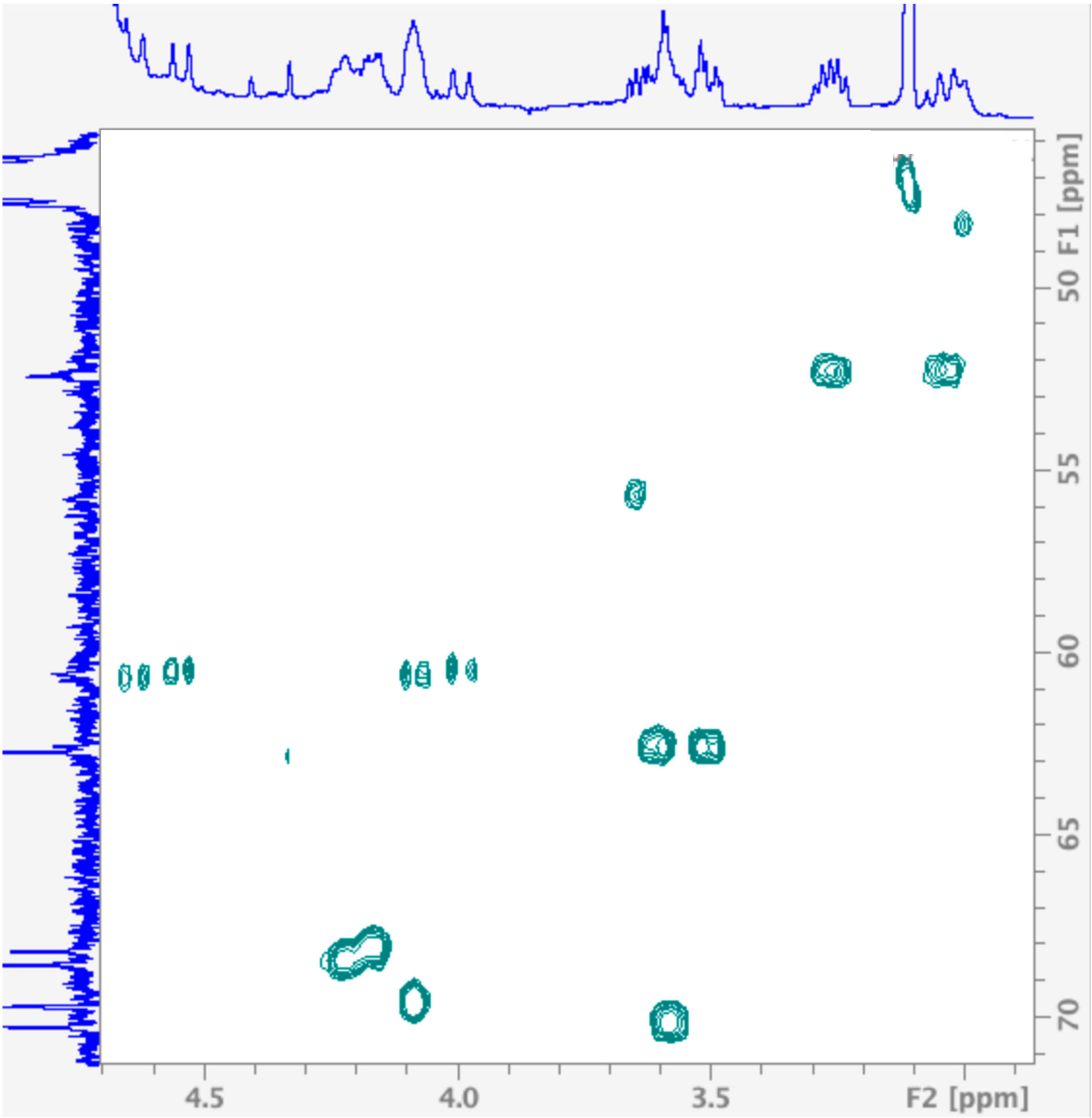

F1 [ppm]
50
55
60
65
70
4.5
4.0
3.5
F2 [ppm]

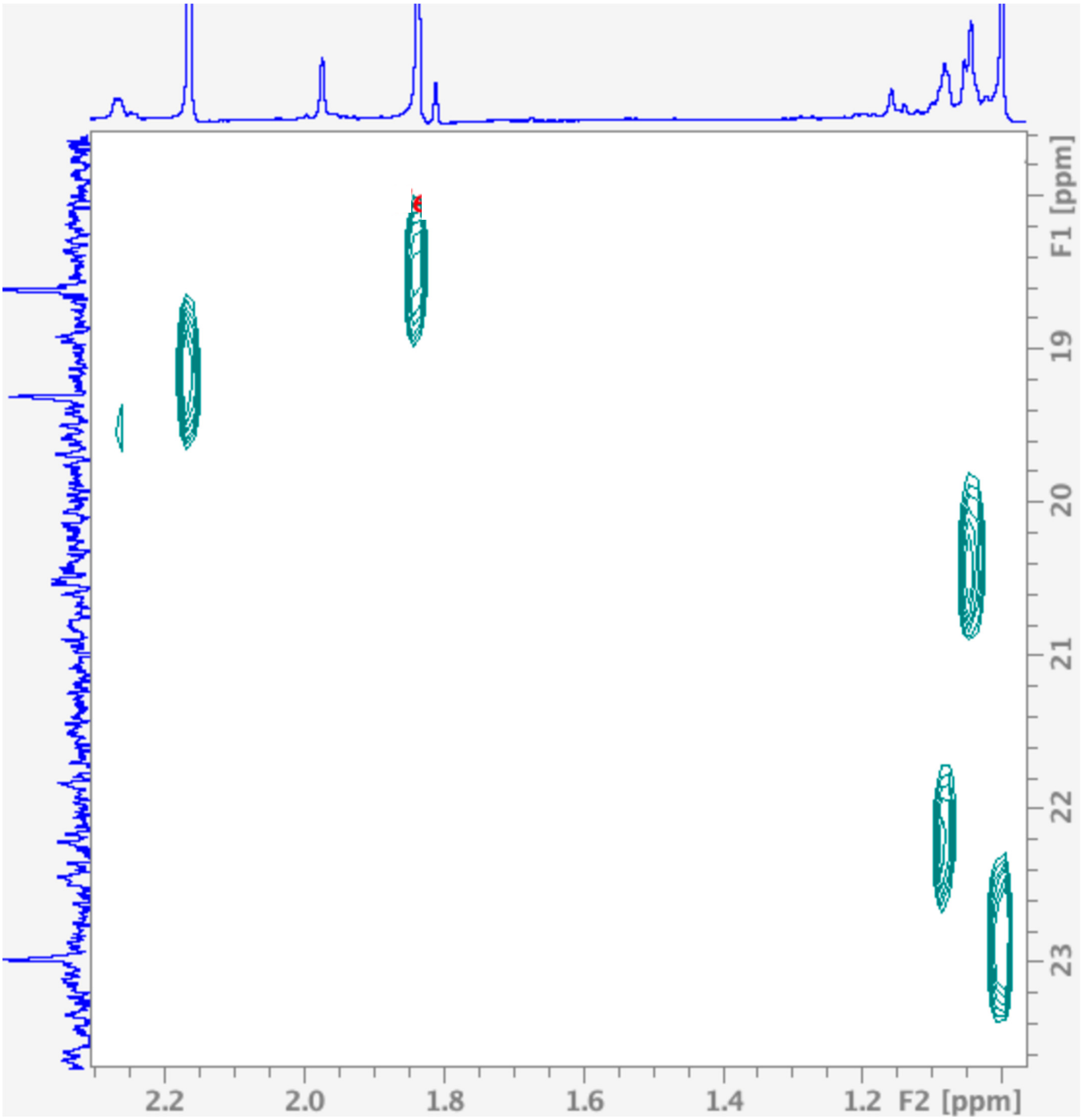

F1 [ppm]
19
20
21
22
23
2.2
2.0
1.8
1.6
1.4
1.2
F2 [ppm]

Figure S6. ^{1}H- (400 MHz), ^{13}C-NMR (100 MHz), DEPT, ^{11}B-NMR (128 MHz), COSY, HSQC and HMBC spectra of *N*-(2-methylphenyl boronic acid pinacol ester)-3,6-dideoxy-3,6-imino-1,2-*O*-isopropylidene-α-D-gulofuranose **ortho 2** in $CDCl_3$.

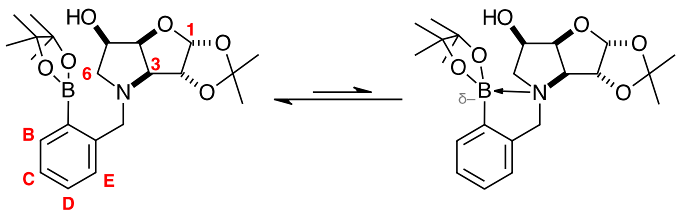

Boronic acid species. **δ_H**: 7.80 (1H, dd, $J_{HB,HC}$ 7.3 Hz, $J_{HB,HD}$ 1.1 Hz, ArH^B), 7.39 (1H, td, $J_{HD,HC/HE}$ 7.5 Hz, $J_{HD,HB}$ 1.4 Hz, ArH^D), 7.34 (1H, app-d, $J_{HE,HD}$ 7.6 Hz, ArH^E), 7.25 (1H, td partially obscured by $CDCl_3$ signal, $J_{HC,HB/HD}$ 7.2 Hz, $J_{HC,HE}$ 1.3 Hz, ArH^C), 5.89 (1H, d, $J_{H\text{-}1,H\text{-}2}$ 3.4 Hz, H-1), 4.81 (1H, app-t, $J_{H\text{-}4,H\text{-}3/H\text{-}5}$ 5.8 Hz, H-4), 4.40 (1H, d, $J_{H\text{-}2,H\text{-}1}$ 3.4 Hz, H-2), 4.14 (1H, app-dddd, $J_{H\text{-}5,H\text{-}4/H\text{-}6/OH}$ 6.0 Hz, $J_{H\text{-}5,H\text{-}6'}$ 2.4 Hz, H-5), 4.01 (1H, d, $J_{Ha,Hb}$ 13.2 Hz, CH^aH^{b}Ar), 3.91 (1H, d, $J_{Hb,Ha}$ 13.2 Hz, CH^aH^bAr), 3.27 (1H, app-d, $J_{H\text{-}3,H\text{-}4}$ 5.5 Hz, H-3), 2.96 (1H, dd, $J_{H\text{-}6',H\text{-}6}$ 10.8 Hz, $J_{H\text{-}6',H\text{-}5}$ 2.4 Hz, H-6′), 2.80 (1H, d, $J_{OH,H\text{-}5}$ 6.8 Hz, OH), 2.50 (1H, dd, $J_{H\text{-}6,H\text{-}6'}$ 10.8 Hz, $J_{H\text{-}6,H\text{-}5}$ 5.6 Hz, H-6), 1.49, 1.28 (6H, 2 x s, 2 x CH_3, acetonide), 1.352, 1.347 (12H, 2 x s, pinacol 4 x CH_3); **δ_C**: 144.2 (ArC$_{quat}$), 136.1 (ArC^B), 130.9 (ArC^D), 129.4 (ArC^E), 126.7 (ArC^C), 112.6 (C$_{quat}$ acetonide), 107.5 (C-1), 83.9 (C-2), 83.8 (2 x C$_{quat}$ pinacol), 83.2 (C-4), 72.9 (C-3), 69.7 (C-5), 61.6 (C-6), 57.7 (CH_2Ar), 27.7, 27.0 (2 x CH_3, acetonide), 25.1, 25.0 (4 x CH_3 pinacol). ArC$_{quat}$-B not discernible.
δ_B: 31.0 (sharp, integration: 6.4), 22.3 (sharp, integration: 1.0).

¹H-NMR

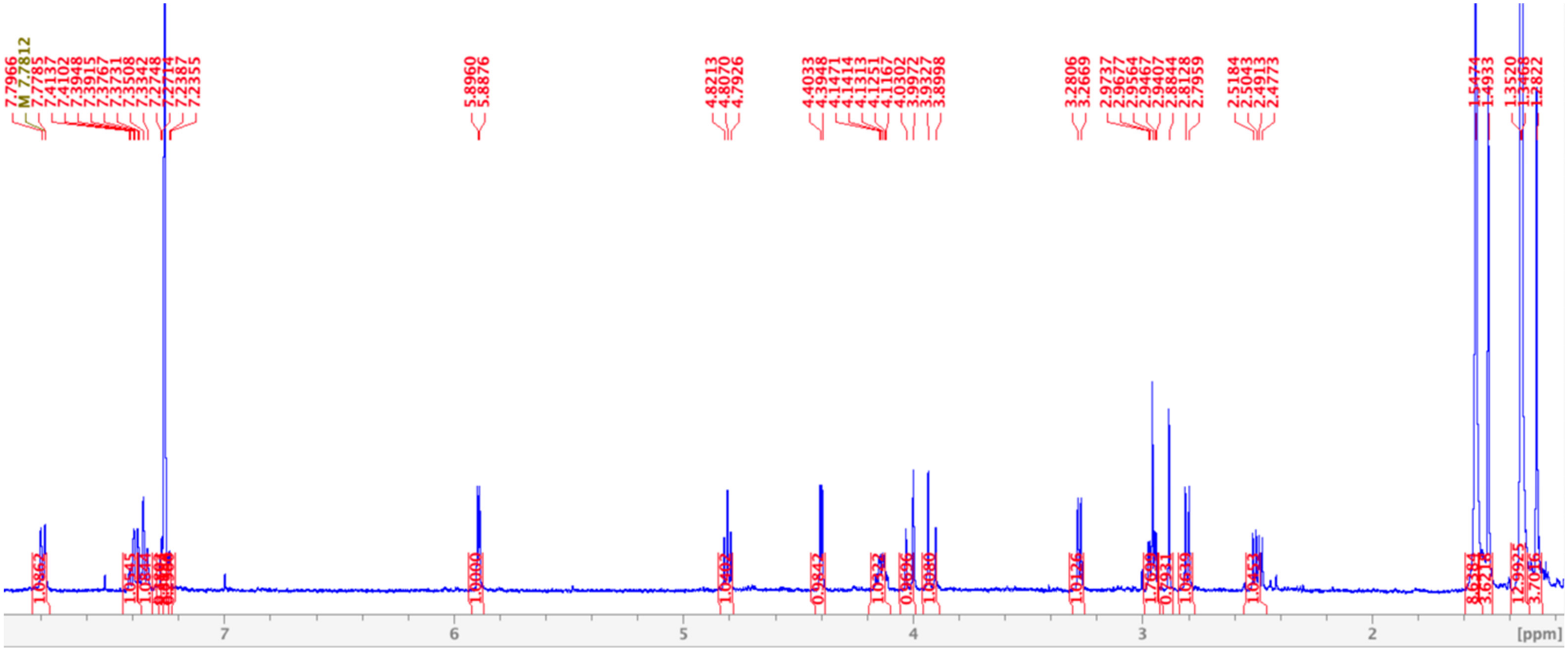

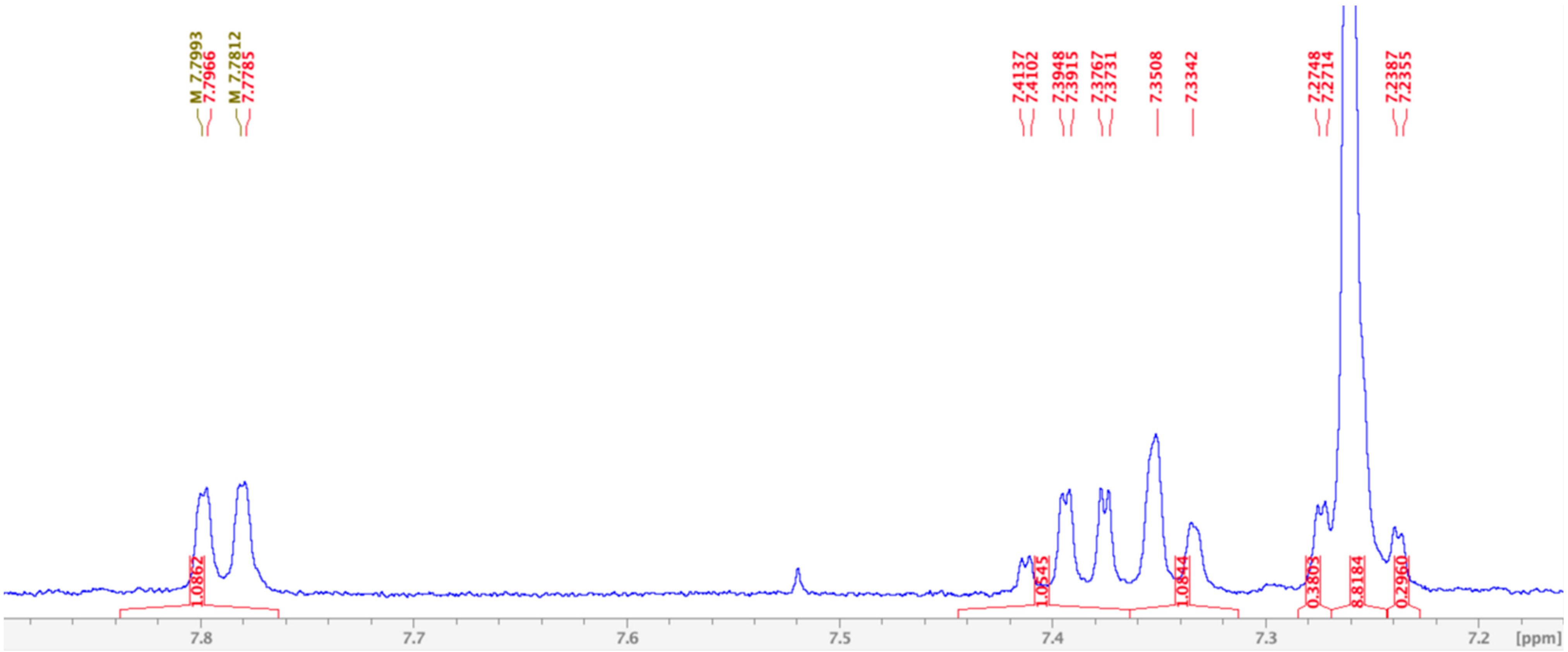

M 7.7993
7.7966
M 7.7812
7.7785
7.4137
7.4102
7.3948
7.3915
7.3767
7.3731
7.3508
7.3342
7.2748
7.2714
7.2387
7.2355
1.0862
1.0545
1.0844
0.3803
8.8184
0.2960
7.8
7.7
7.6
7.5
7.4
7.3
7.2
[ppm]

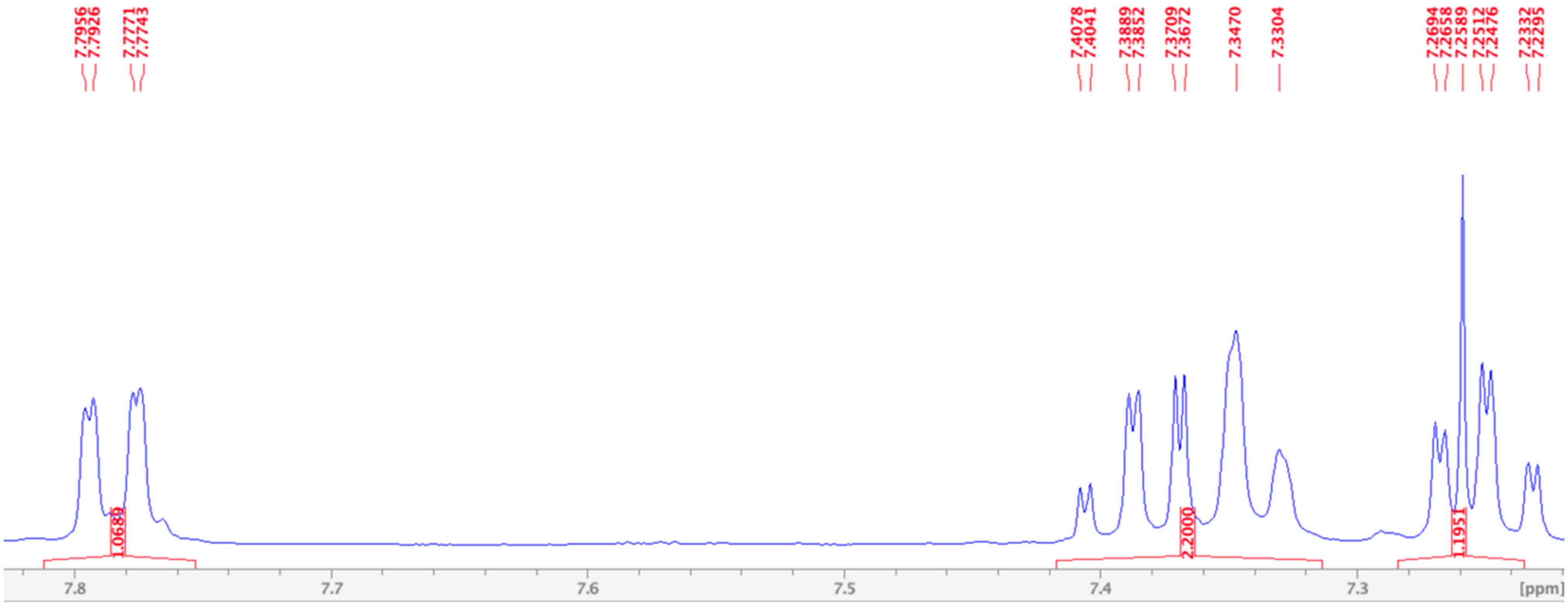
7.7956
7.7926
7.7771
7.7743
7.4078
7.4041
7.3889
7.3852
7.3709
7.3672
7.3470
7.3304
7.2694
7.2658
7.2589
7.2512
7.2476
7.2332
7.2295
1.0689
2.2000
1.1951
7.8
7.7
7.6
7.5
7.4
7.3
[ppm]

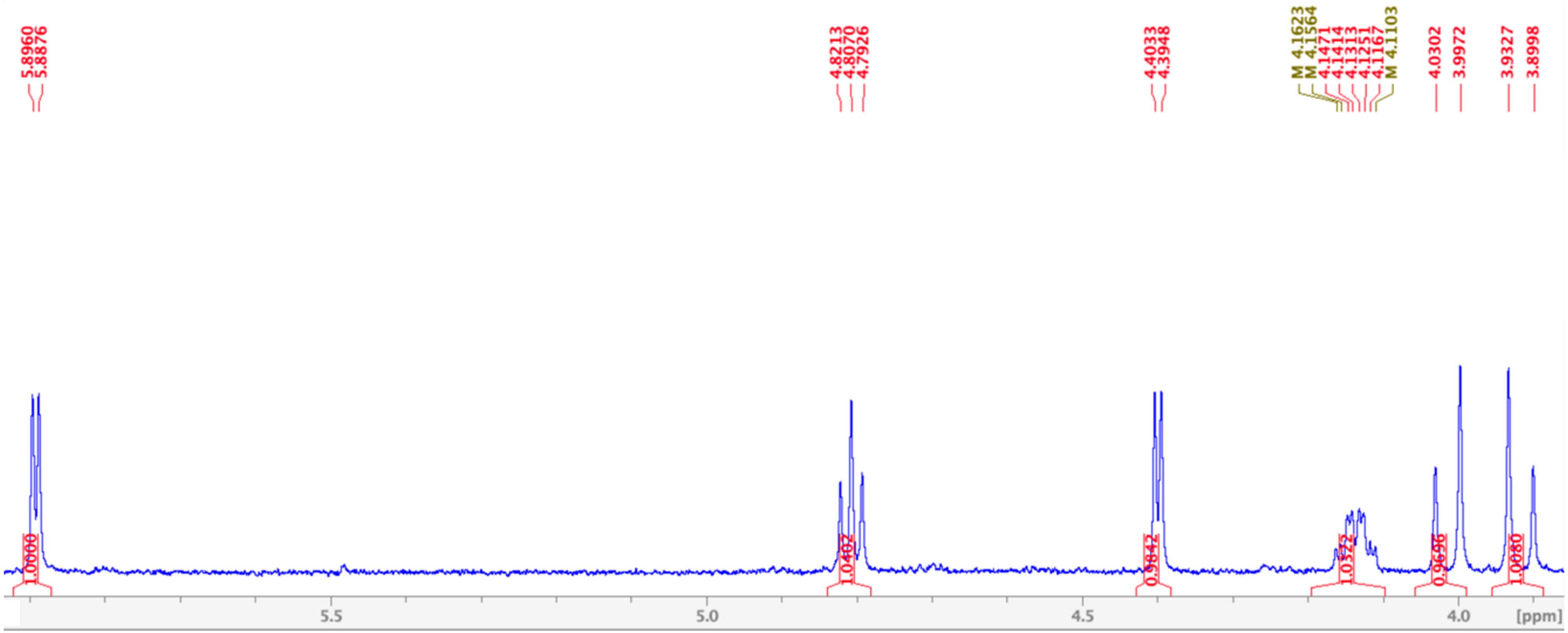
5.8960
5.8876
4.8213
4.8070
4.7926
4.4033
4.3948
M 4.1623
M 4.1564
4.1471
4.1414
4.1313
4.1251
4.1167
M 4.1103
4.0302
3.9972
3.9327
3.8998
1.0000
1.0402
0.9842
1.0372
0.9696
1.0080
5.5
5.0
4.5
4.0
[ppm]

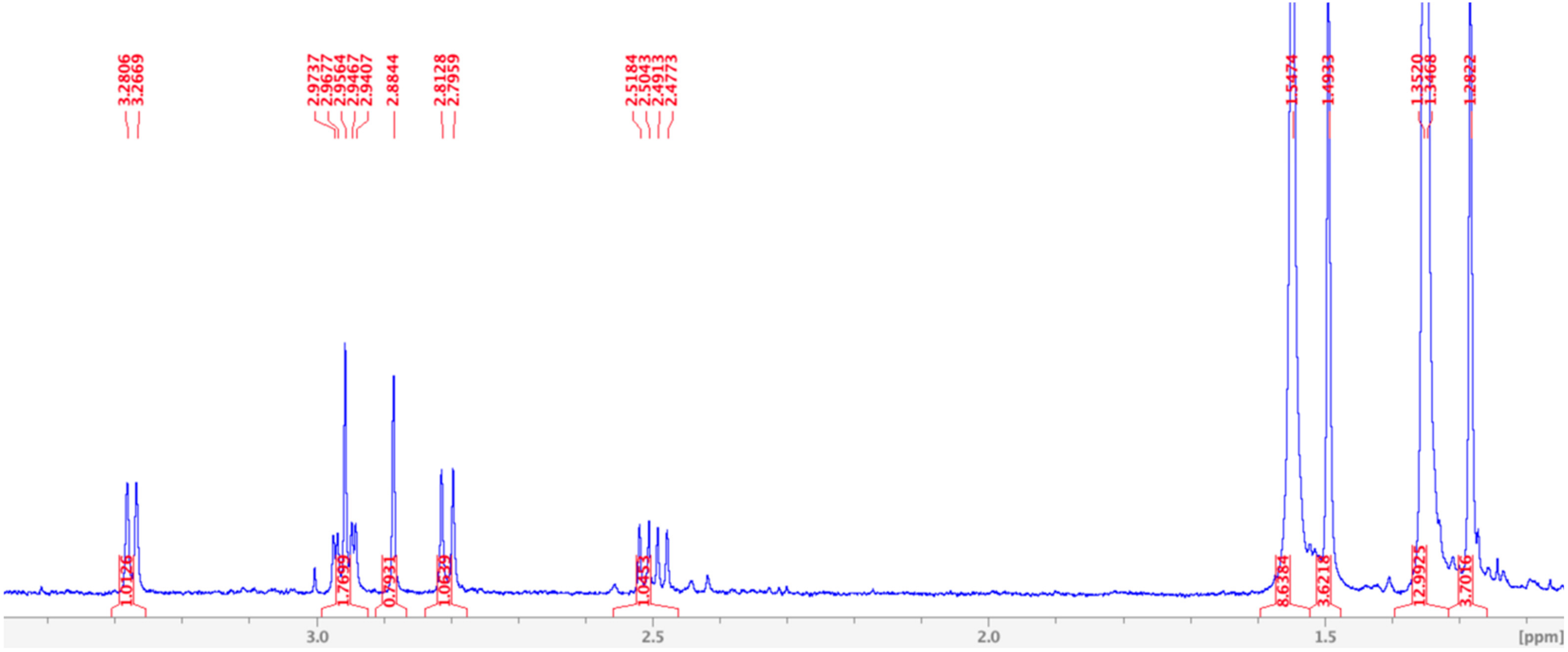
3.2806
3.2669
2.9737
2.9677
2.9564
2.9467
2.9407
2.8844
2.8128
2.7959
2.5184
2.5043
2.4913
2.4773
1.5474
1.4933
1.3520
1.3468
1.2822
1.0126
1.7699
0.7931
1.0629
1.0453
8.6384
3.6218
12.9925
3.7016
3.0
2.5
2.0
1.5
[ppm]

^{13}C-NMR

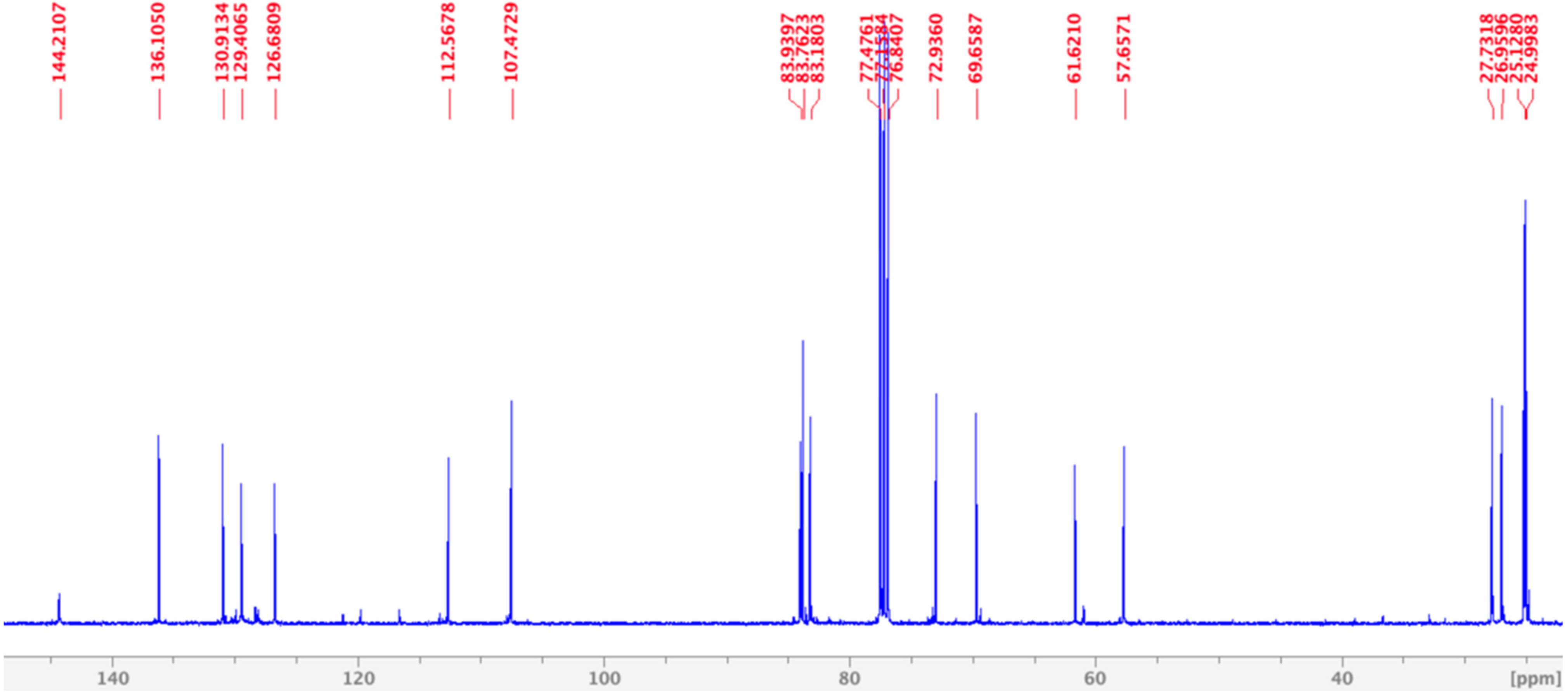
144.2107
136.1050
130.9134
129.4065
126.6809
112.5678
107.4729
83.9397
83.7623
83.1803
77.4761
76.8407
72.9360
69.6587
61.6210
57.6571
27.7318
26.9596
25.1280
24.9983
140
120
100
80
60
40
[ppm]

DEPT

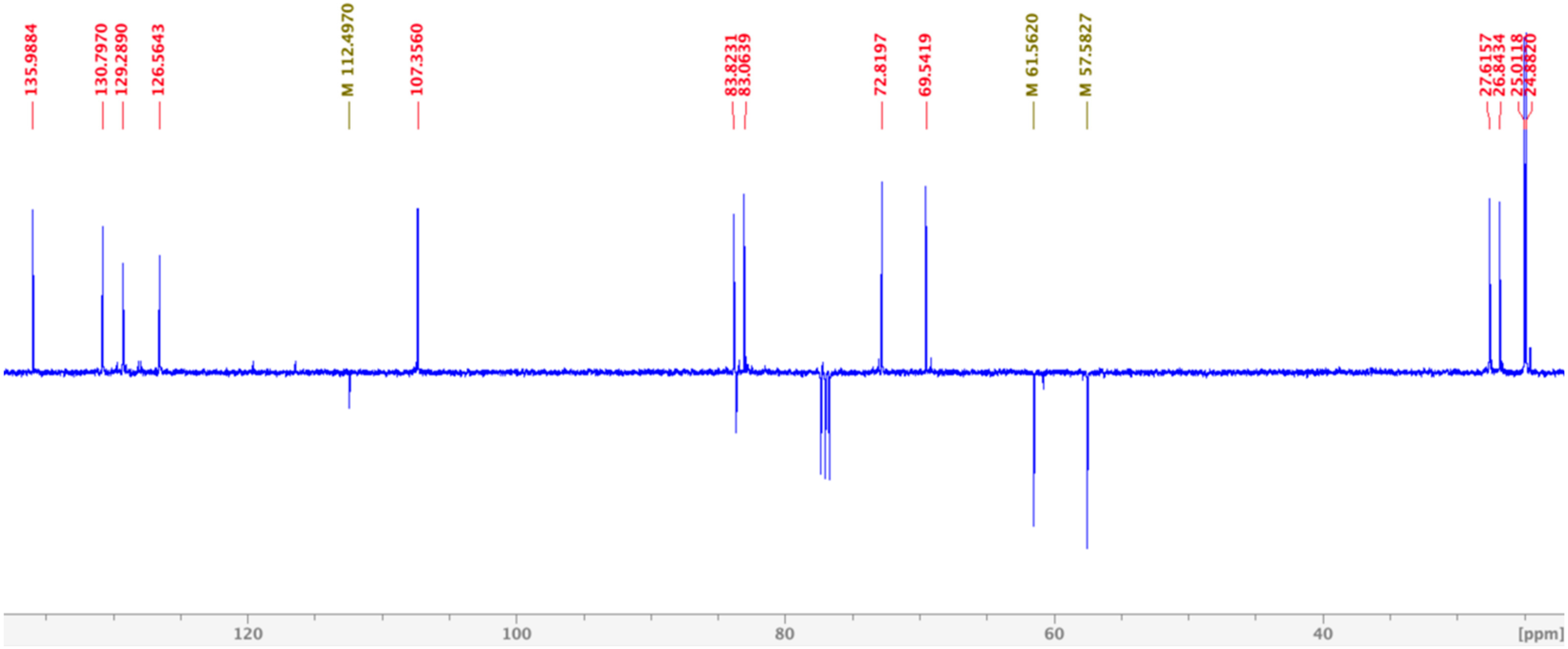

135.9884
130.7970
129.2890
126.5643
M 112.4970
107.3560
83.8231
83.0639
72.8197
69.5419
M 61.5620
M 57.5827
27.6157
26.8434
25.0118
24.8820
120
100
80
60
40
[ppm]

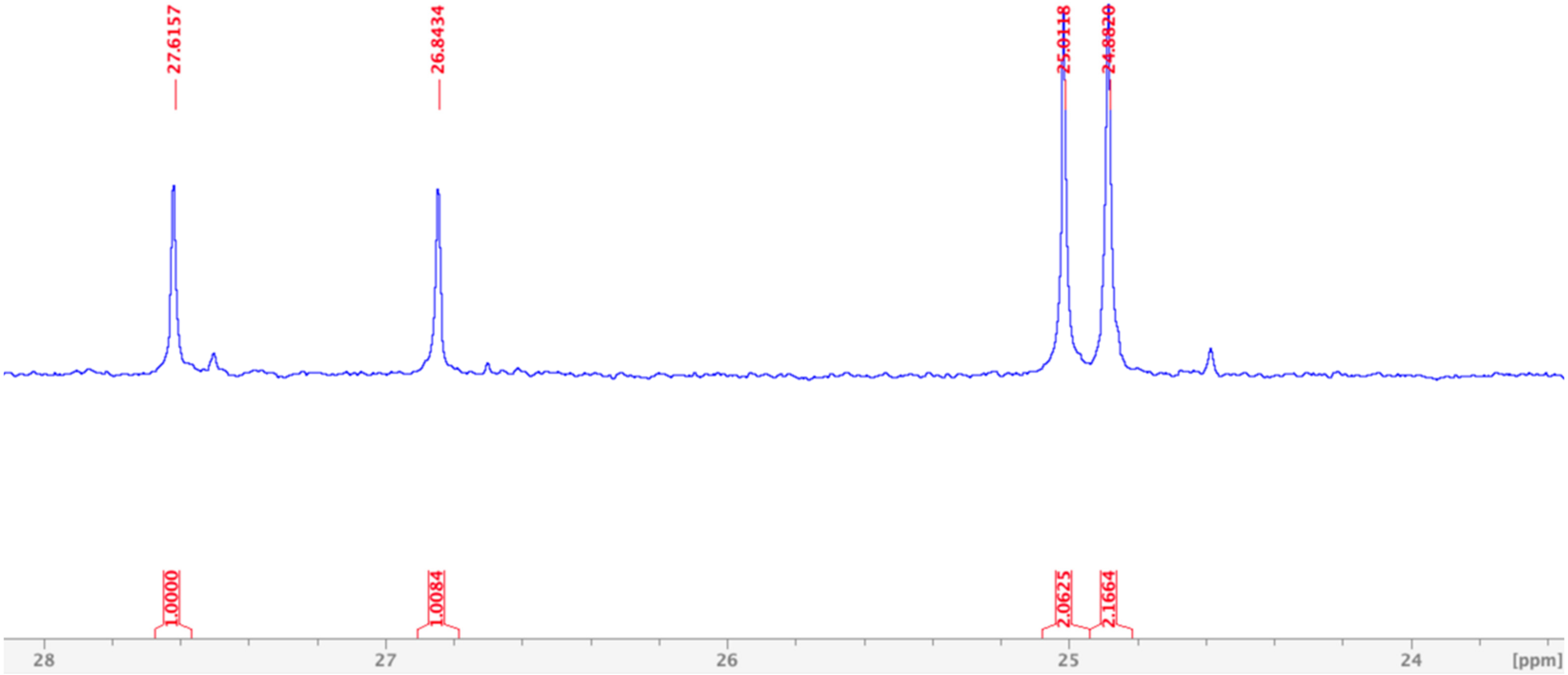
27.6157
26.8434
25.0118
24.8820
1.0000
1.0084
2.0625
2.1664
28
27
26
25
24
[ppm]

^{11}B-NMR

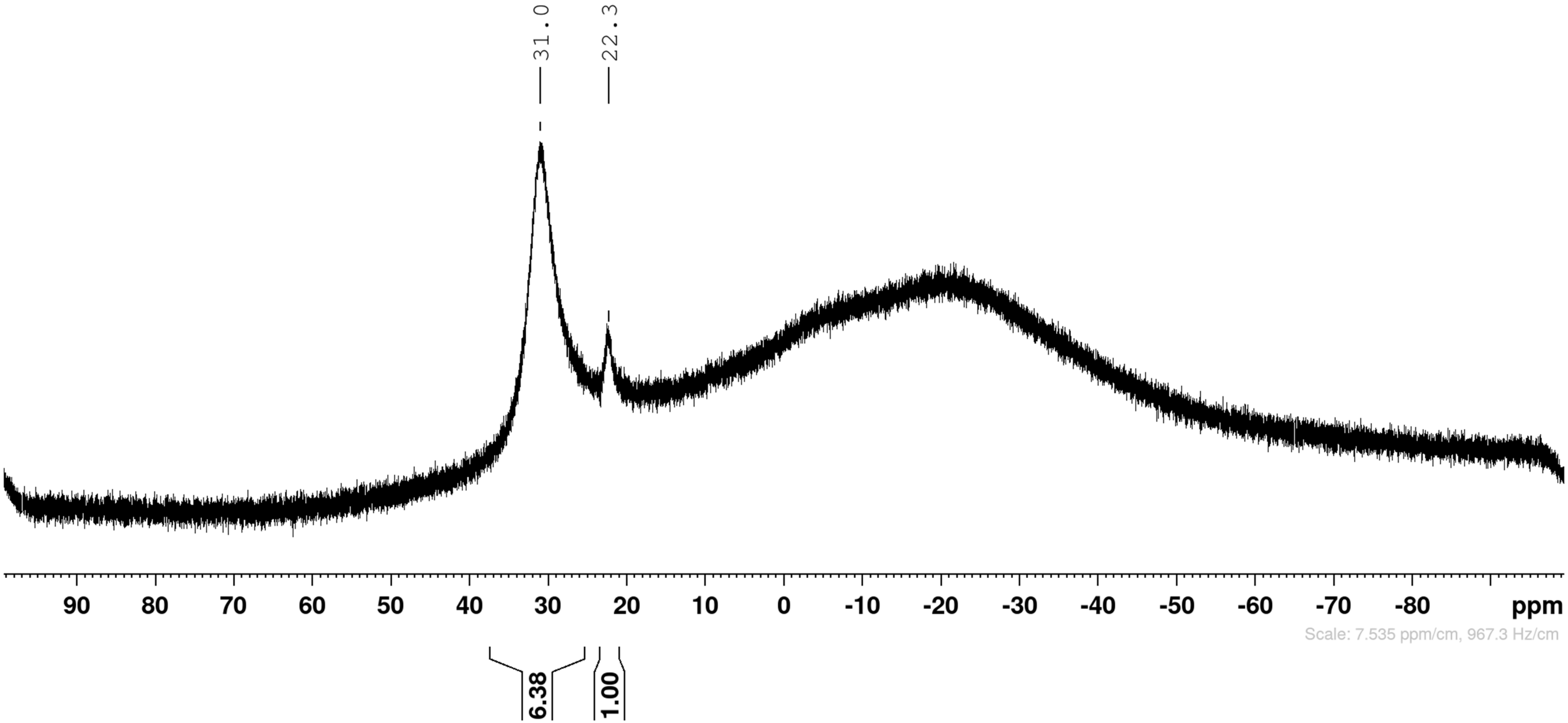

31.0
22.3
90
80
70
60
50
40
30
20
10
0
-10
-20
-30
-40
-50
-60
-70
-80
ppm
Scale: 7.535 ppm/cm, 967.3 Hz/cm
6.38
1.00

COSY

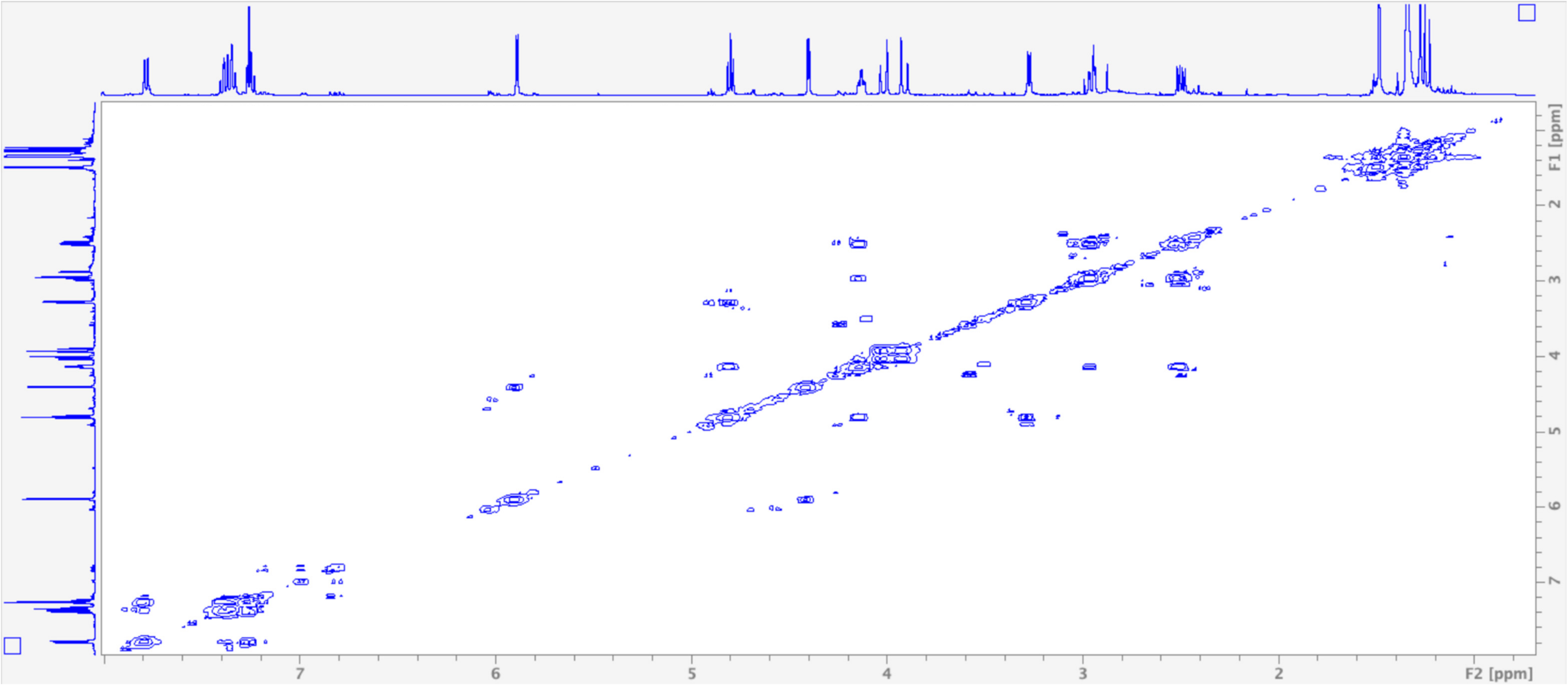

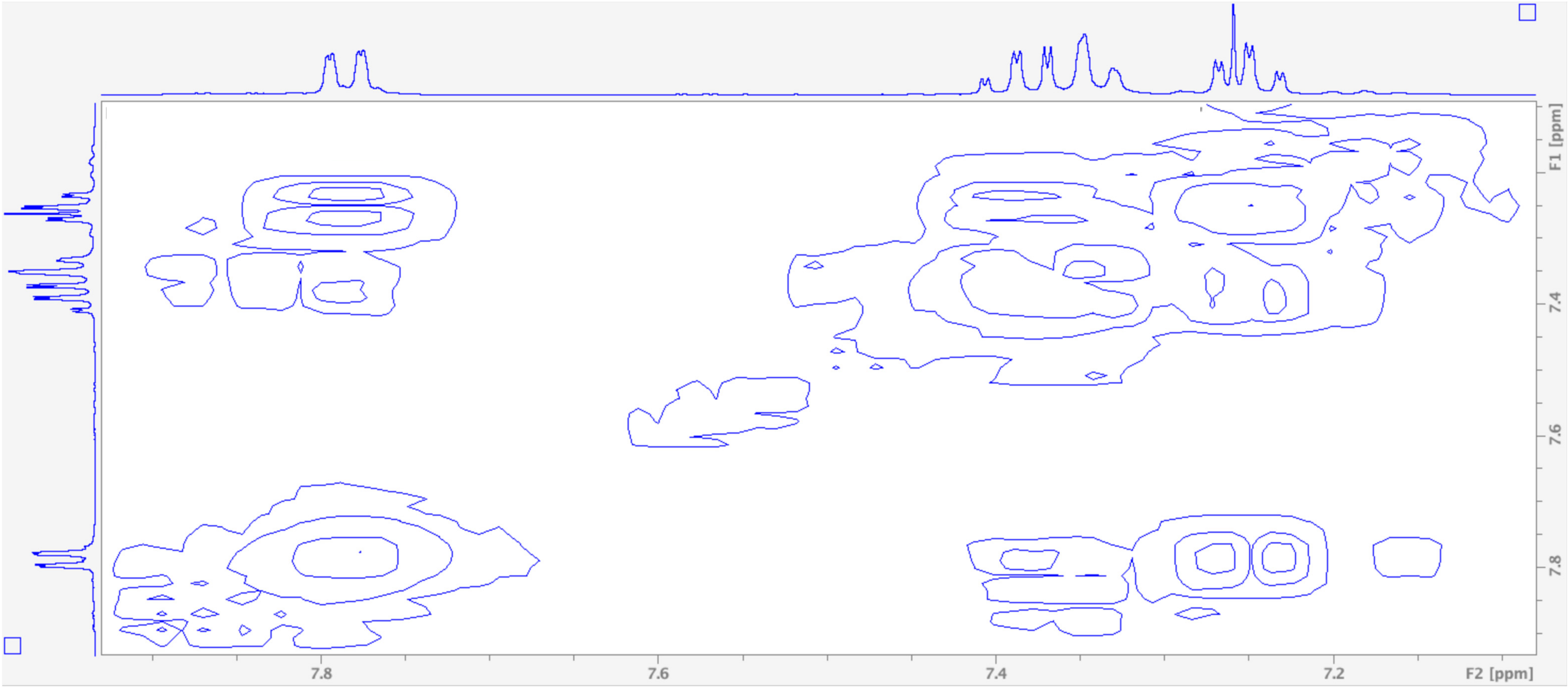
F1 [ppm]
7.4
7.6
7.8
7.8
7.6
7.4
7.2
F2 [ppm]

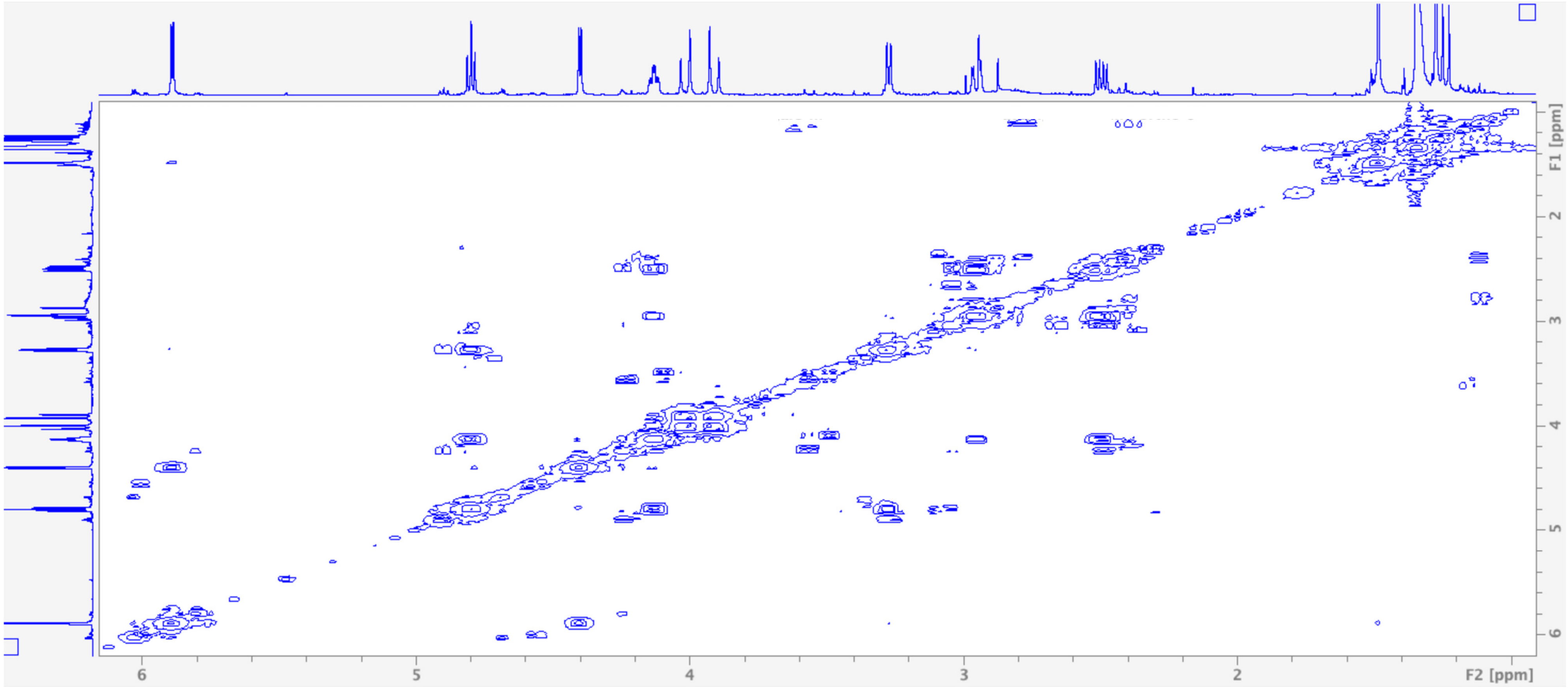

F1 [ppm]
F2 [ppm]
6
5
4
3
2

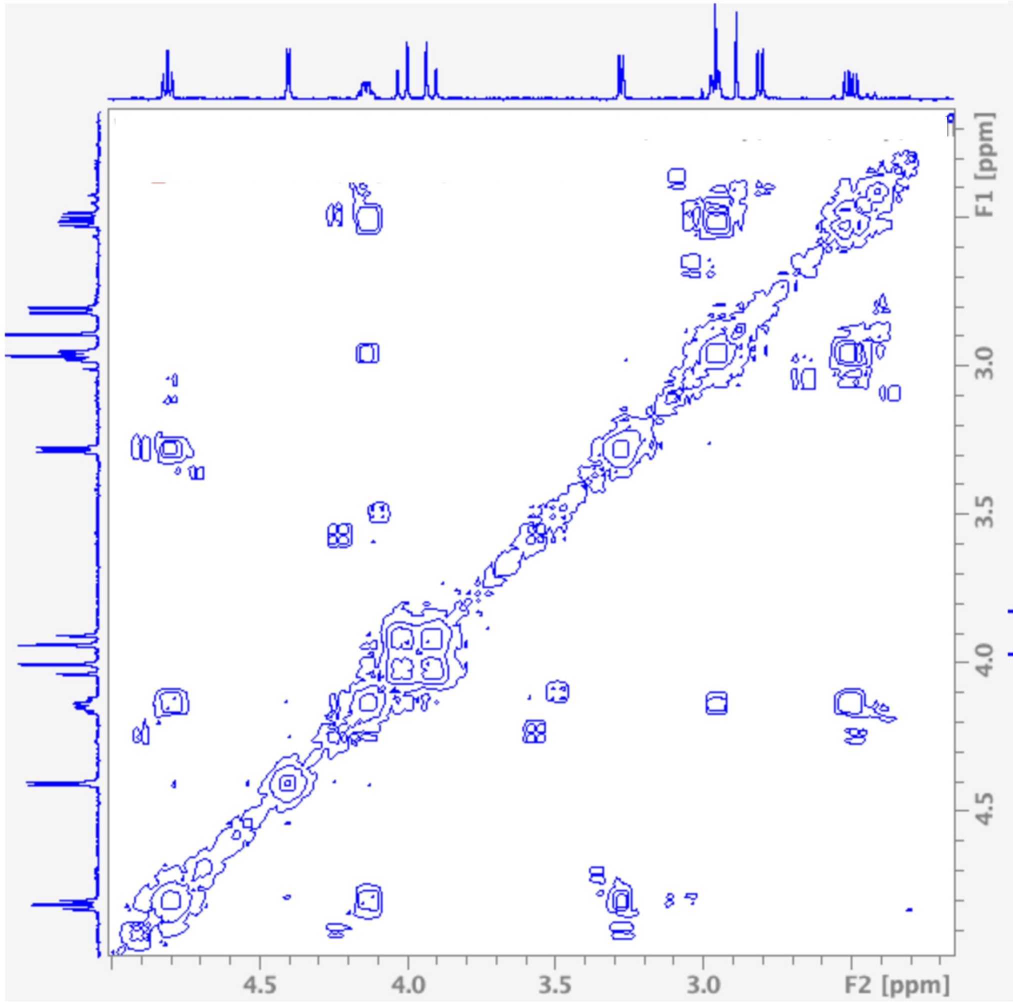
4.5
4.0
3.5
3.0
F2 [ppm]
F1 [ppm]

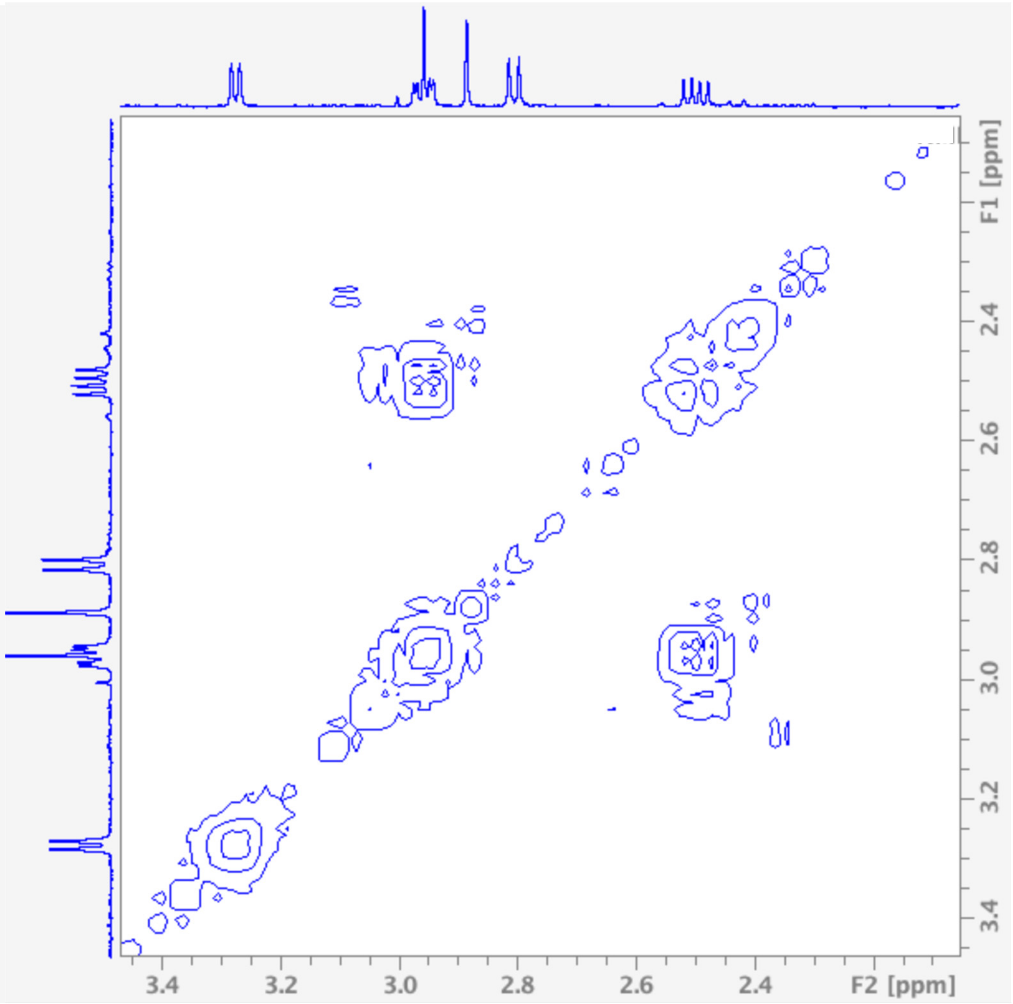
3.4
3.2
3.0
2.8
2.6
2.4
F2 [ppm]
F1 [ppm]

HSQC

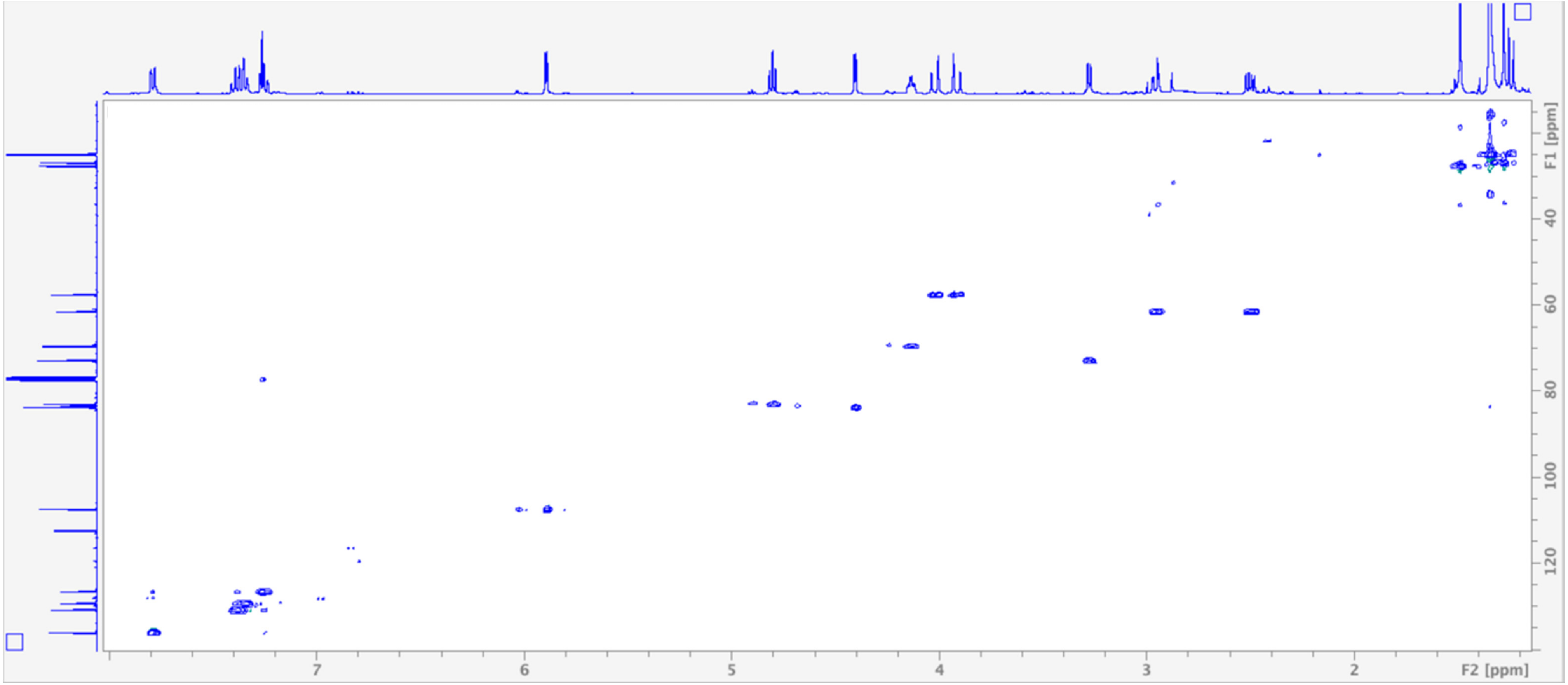

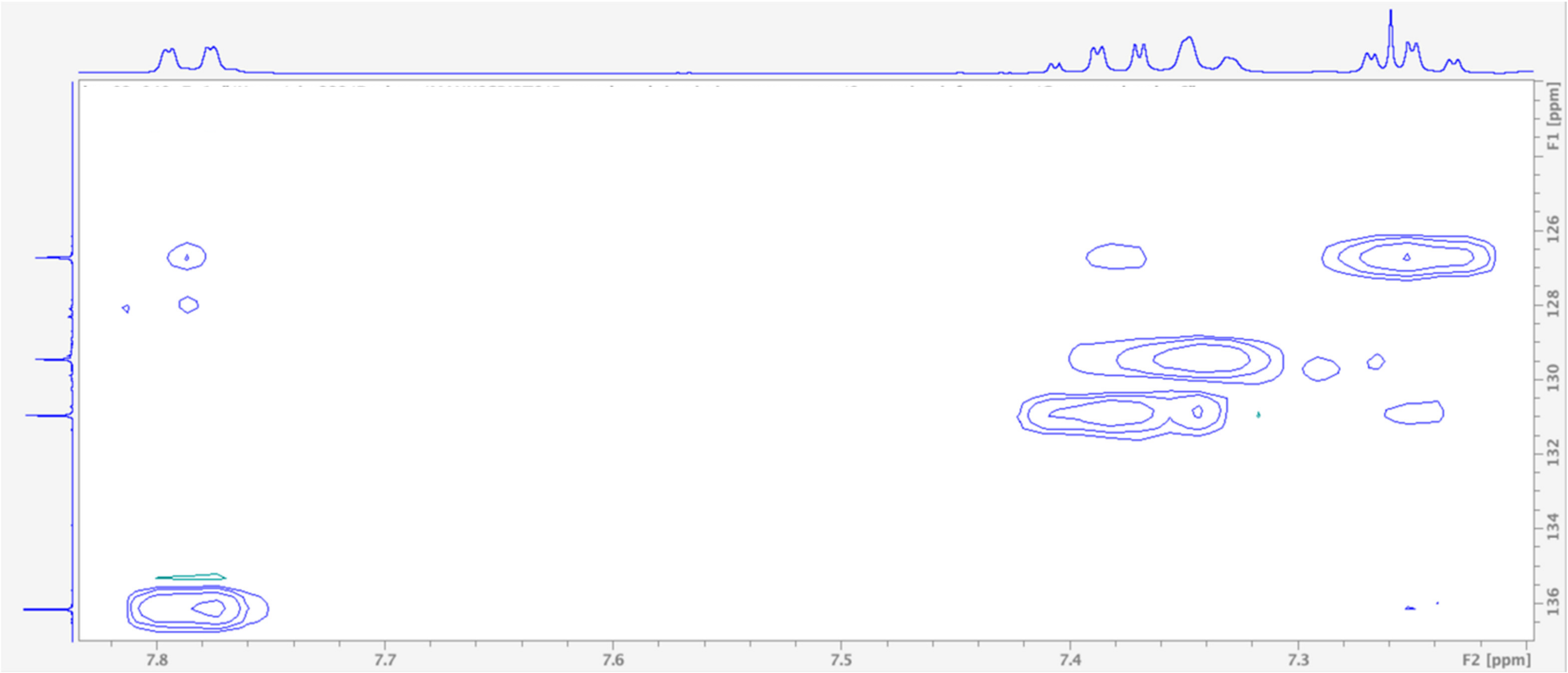
F1 [ppm]
126
128
130
132
134
136
7.8
7.7
7.6
7.5
7.4
7.3
F2 [ppm]

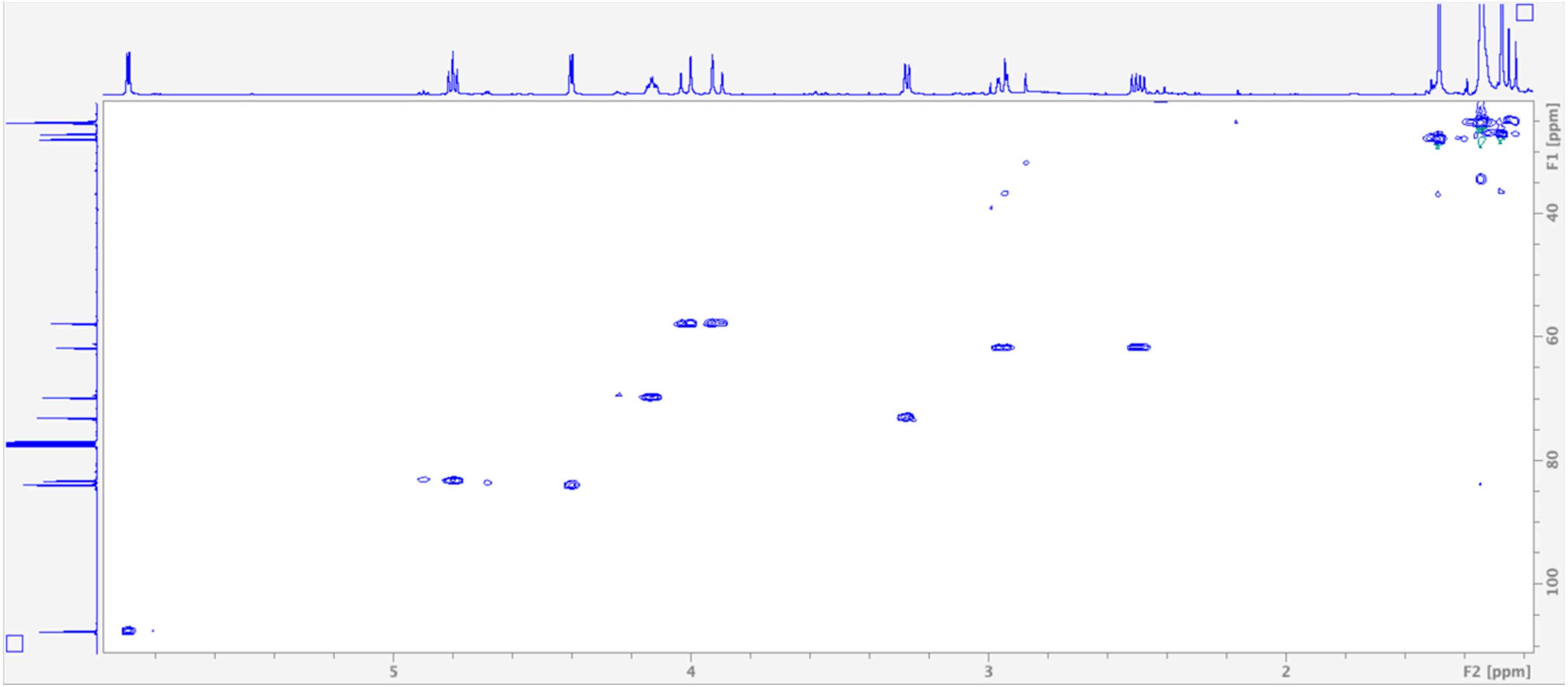
F1 [ppm]
40
60
80
100
5
4
3
2
F2 [ppm]

HMBC

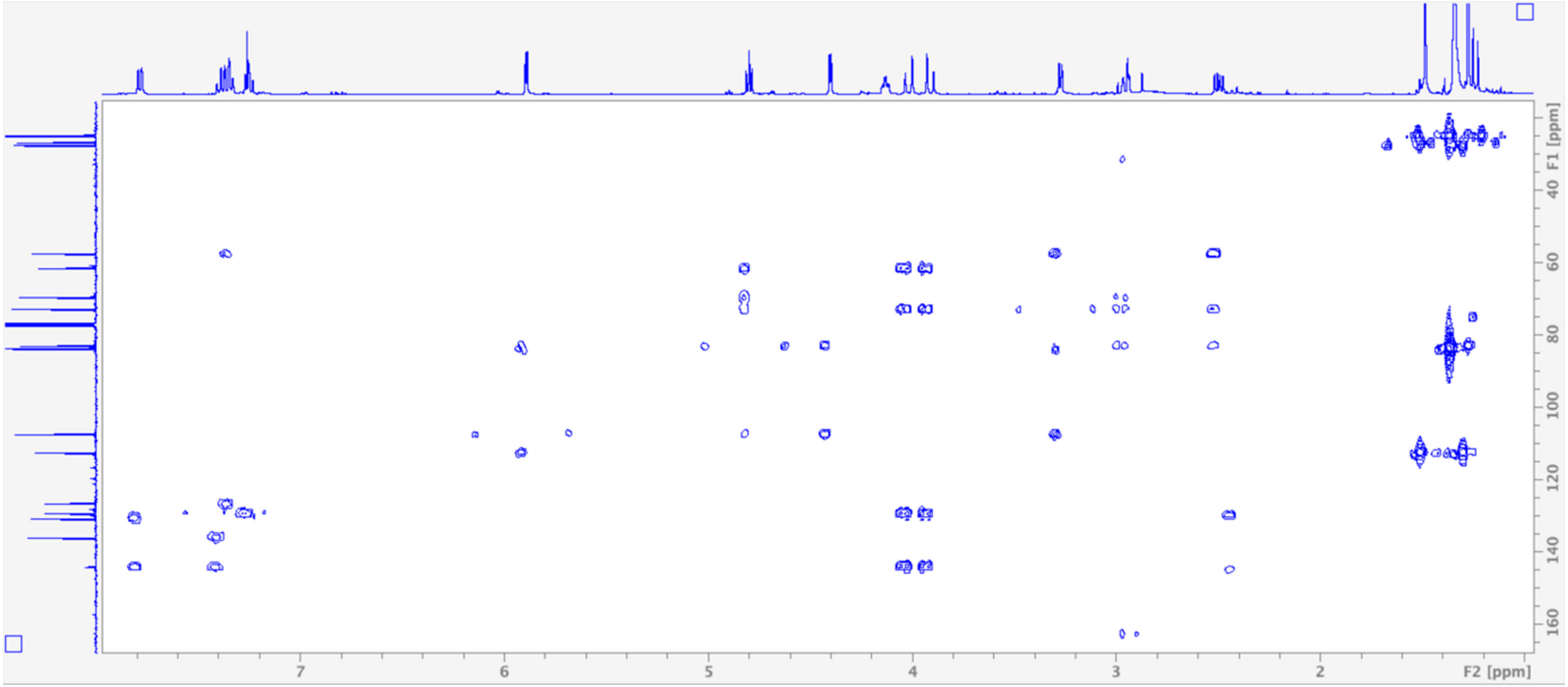

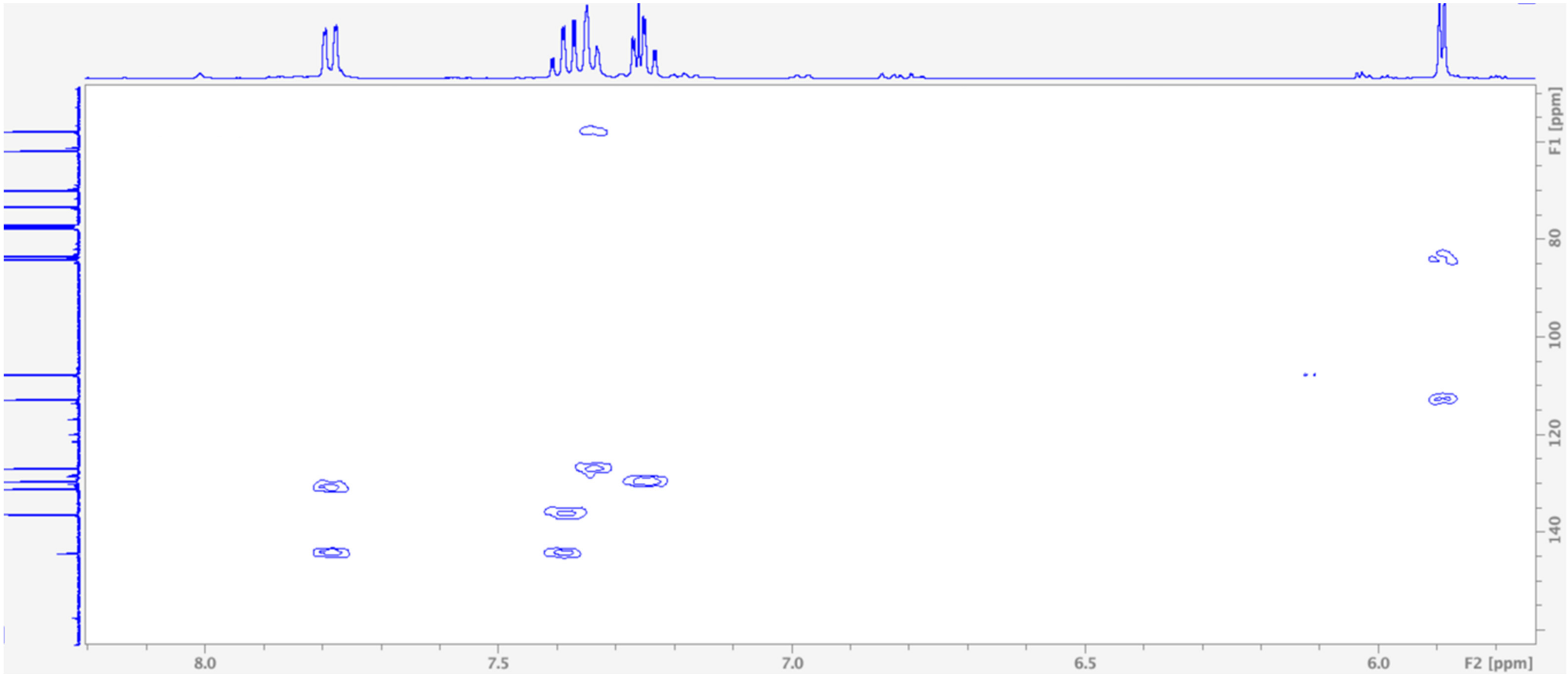
F1 [ppm]
80
100
120
140
8.0
7.5
7.0
6.5
6.0
F2 [ppm]

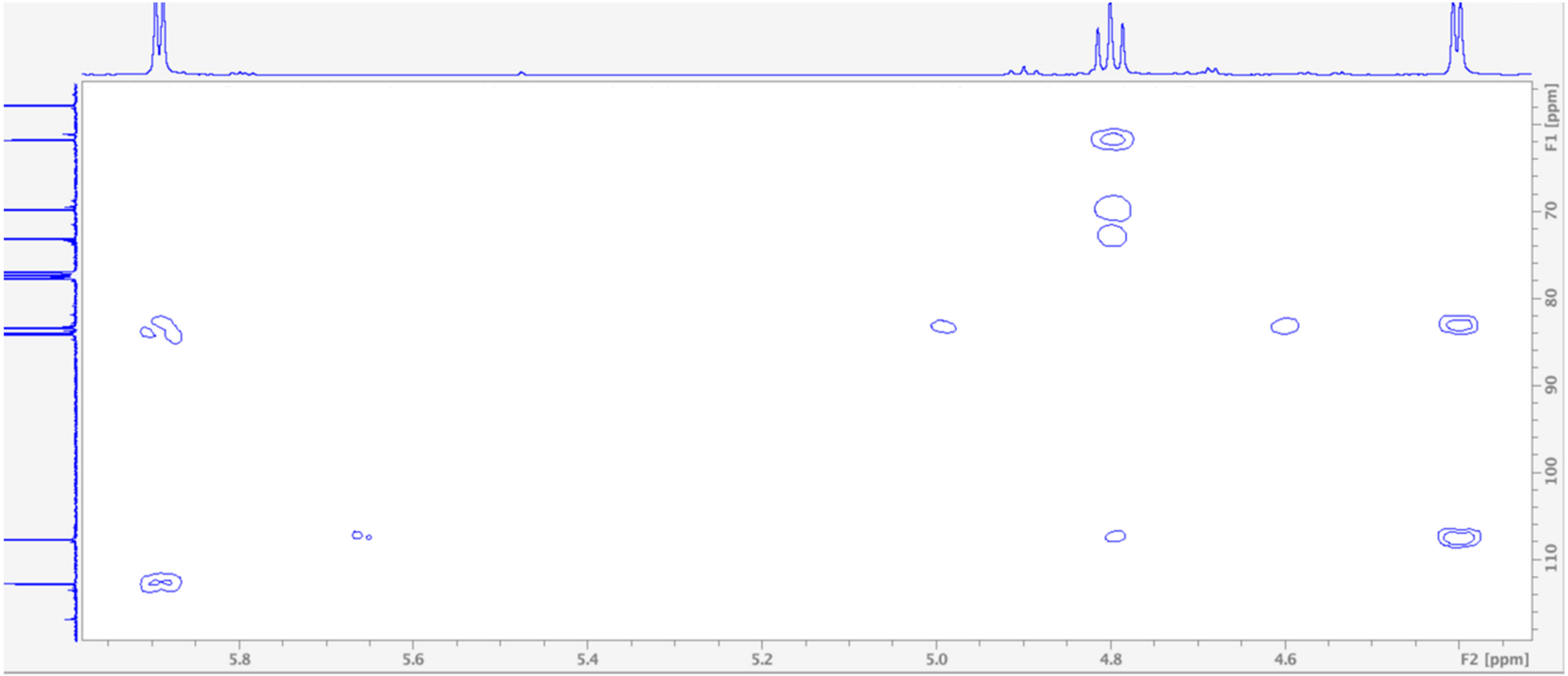
F1 [ppm]
70
80
90
100
110
5.8
5.6
5.4
5.2
5.0
4.8
4.6
F2 [ppm]

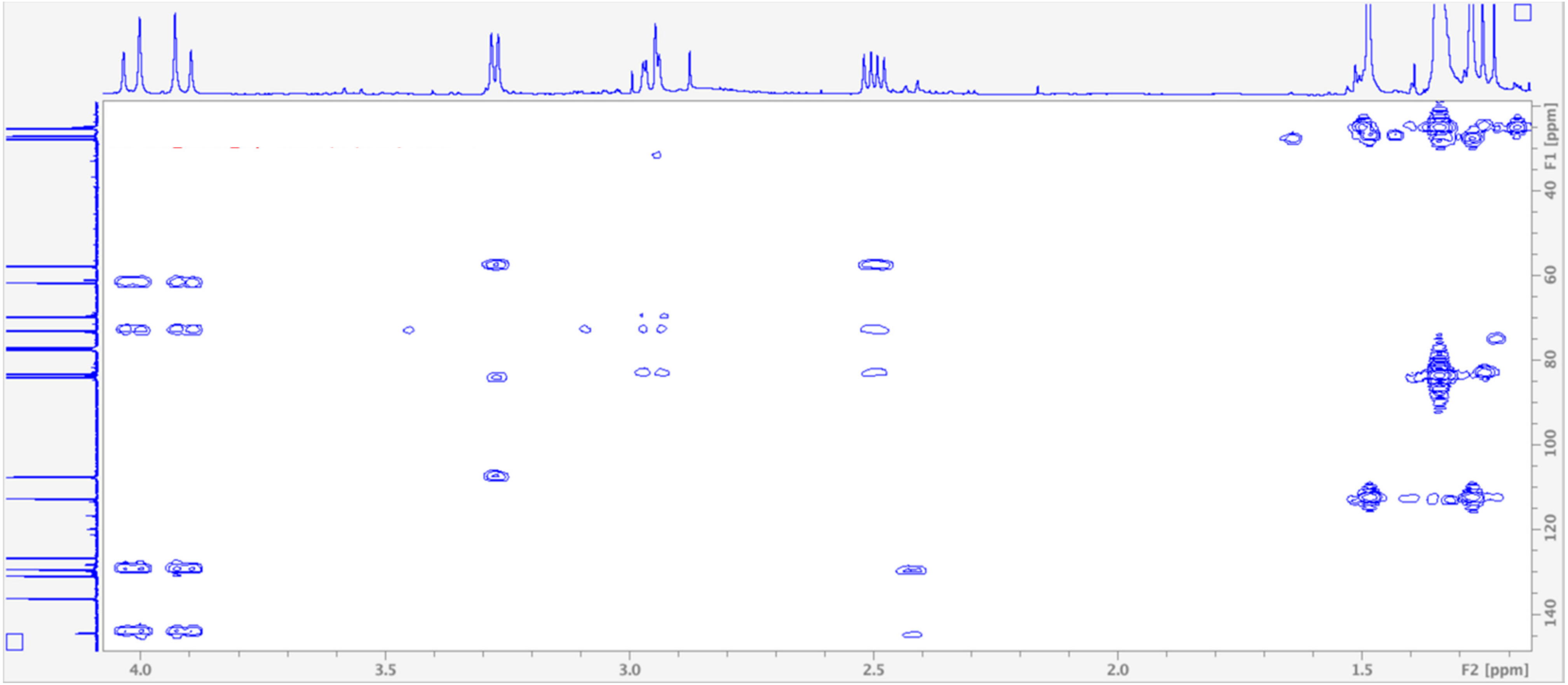

F1 [ppm]
40
60
80
100
120
140
4.0
3.5
3.0
2.5
2.0
1.5
F2 [ppm]

Figure S7. ^{1}H- (400 MHz), ^{13}C-NMR (100 MHz), DEPT, ^{11}B-NMR (128 MHz), COSY, HSQC and HMBC spectra of *N*-(2-methylphenyl boronic acid)-3,6-dideoxy-3,6-imino-D-gulofuranose **ortho 3** in D_2O.

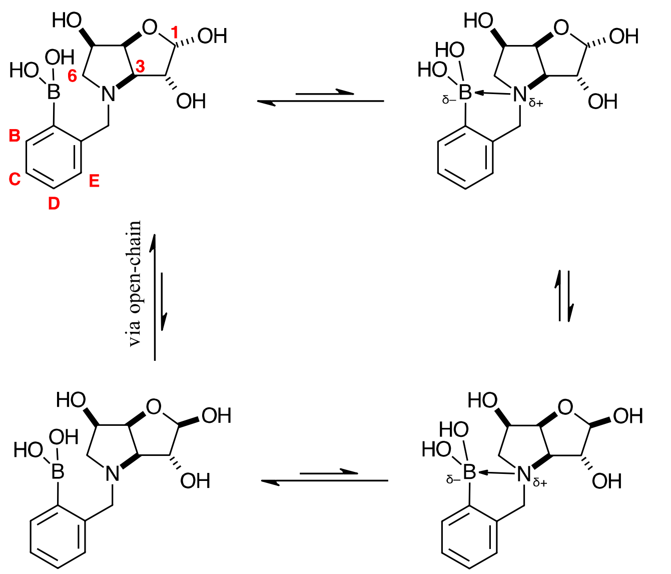

Ratio *α-fur* anomer : *β-fur* anomer, 1.0 : 0.7.

***α-fur* anomer.** δ_H: 7.85 (1H, app-dd, $J_{HA,HB}$ 6.6 Hz, $J_{HA,HC}$ 2.2 Hz, $J_{HA,HD}$ 1.5 Hz, ArH^A), 7.58-7.54 (2Hs, m, overlapping, ArH^B and ArH^C), 7.50 (1H, app-dd, $J_{HD,HC}$ 6.6 Hz, $J_{HD,HB}$ 2.1 Hz, $J_{HD,HA}$ 1.4 Hz, ArH^D), 5.48 (1H, d, $J_{H\text{-}1,H\text{-}2}$ 4.3 Hz, H-1), 5.06-4.99 (1H, m, partially obscured, H-4), 4.76 (1H, d, partially obscured by D_2O signal, ArC*H*^{a}H^b), 4.49-4.44 (1H, obscured, H-5), 4.45 (1H, d, $J_{Hb,Ha}$ 12.8 Hz, ArCH^a*H*^{b}), 4.39-4.28 (1H, m, H-2), 4.31-4.22 (1H, m, H-3), 3.55-3.51 (1Hs, app-s, H-6 and H-6′); δ_C: 135.7 ppm (ArC^A), 134.1 (ArC$_{quat}$), 132.1 (ArC^D), 131.3, 129.6 (ArC^B and ArC^C), 99.5 (C-1, broad signal, width 59 Hz), 79.4 (C-4, broad signal, width 65 Hz), 73.4 (C-2), 72.0 (C-3), 67.2 (C-5), 60.7 ($ArCH_2$, broad signal, width 32 Hz), 60.1 (C-6, broad signal, width 105 Hz); ArC$_{quat}$-B was not discernible.

β-fur anomer. δ_H: 7.88 (1H, app-dd, $J_{HA,HB}$ 6.4 Hz, $J_{HA,HC}$ 2.4 Hz, $J_{HA,HD}$ 1.6 Hz, ArH^A), 7.61-7.56 (2Hs, m, obscured, ArH^B and ArH^C), 7.54 (1H, app-dd, $J_{HD,HC}$ 6.8 Hz, $J_{HD,HB/HA}$ 1.9 Hz, ArH^D), 5.33 (1H, s, H-1), 5.06 (1H, app-t, partially obscured, $J_{H\text{-}4,H\text{-}3}$ 6.1 Hz, H-4), 4.69-4.60 (3H, m, ArC*H*^{a}H^b, ArCH^a*H*^{b} and H-5), 4.18 (1H, d, J 6.4 Hz, H-3), 3.73 (1H, dd, $J_{H\text{-}6,H\text{-}6'}$ 12.4, $J_{H\text{-}6,H\text{-}5}$ 4.5 Hz, H-6), 3.65-3.58 (1H, obscured, H-2), 3.59 (1H, dd, $J_{H\text{-}6',H\text{-}6}$ 12.4, $J_{H\text{-}6',H\text{-}5}$ 5.6 Hz, H-6′); δ_C: 135.9 (ArC^A), 134.3 (ArC$_{quat}$), 132.2 (ArC^D), 131.5, 129.8 (ArC^B and ArC^C), 102.9 (C-1), 81.8 (C-4, slightly broadened, width 29 Hz), 75.6 (C-2), 73.4 (C-3, slightly broadened, width 27 Hz), 67.4 (C-5), 60.1 (C-6, broad, 105 Hz), 60.1 ($ArCH_2$, broad, width 105 Hz); ArC$_{quat}$-B was not discernible.

δ_B: 28.0 (broad, integration: 4.0), 19.3 (sharp, integration: 1.0).

¹H-NMR

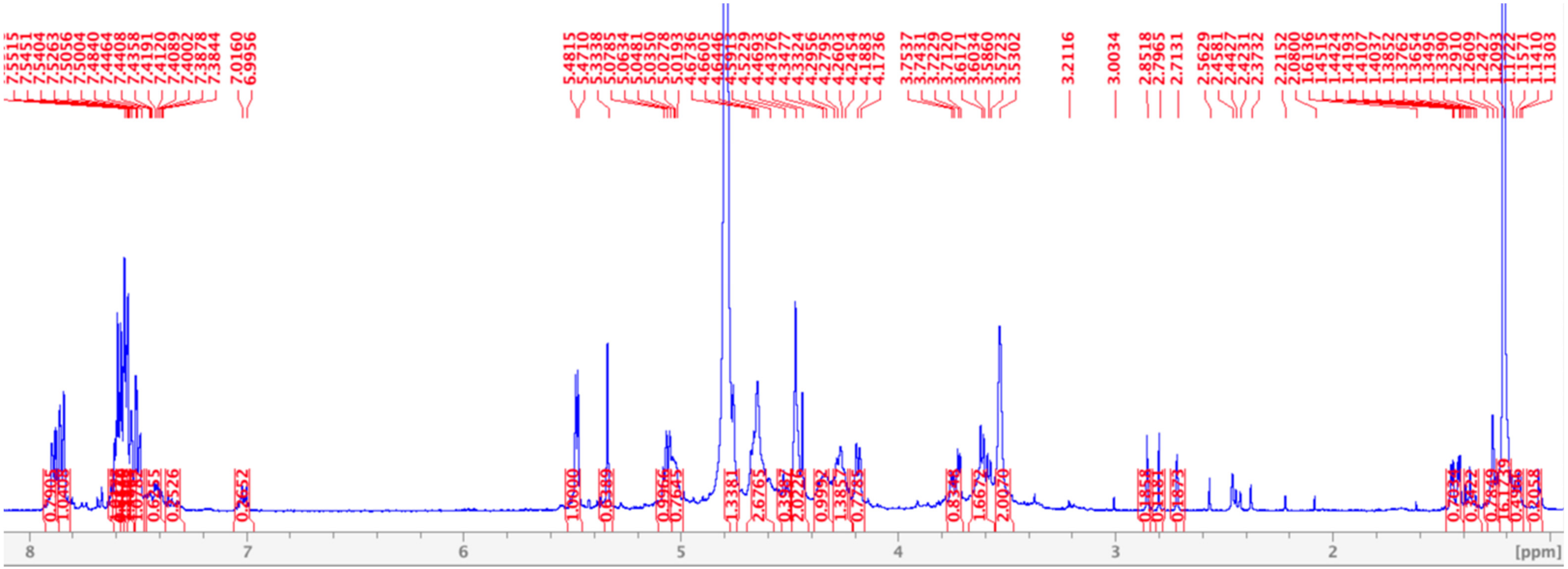

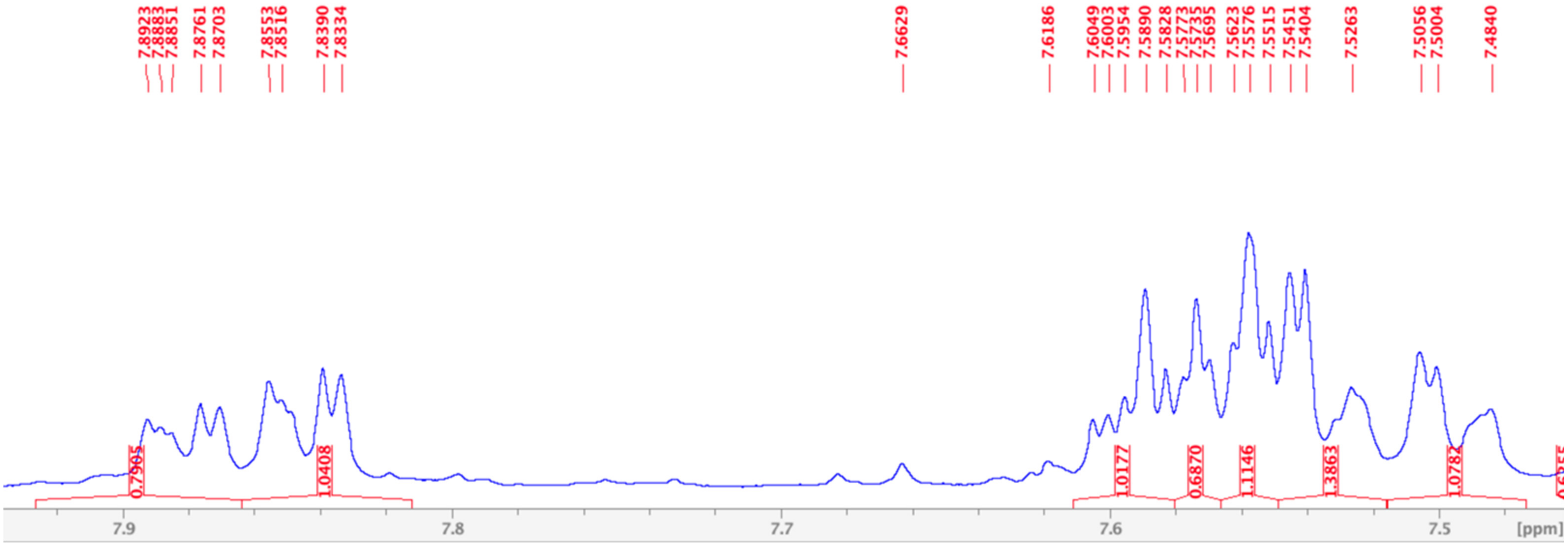

7.8923
7.8883
7.8851
7.8761
7.8703
7.8553
7.8516
7.8390
7.8334
7.6629
7.6186
7.6049
7.6003
7.5954
7.5890
7.5828
7.5773
7.5735
7.5695
7.5623
7.5576
7.5515
7.5451
7.5404
7.5263
7.5056
7.5004
7.4840
0.7905
1.0408
1.0177
0.6870
1.1146
1.3863
1.0782
7.9
7.8
7.7
7.6
7.5
[ppm]

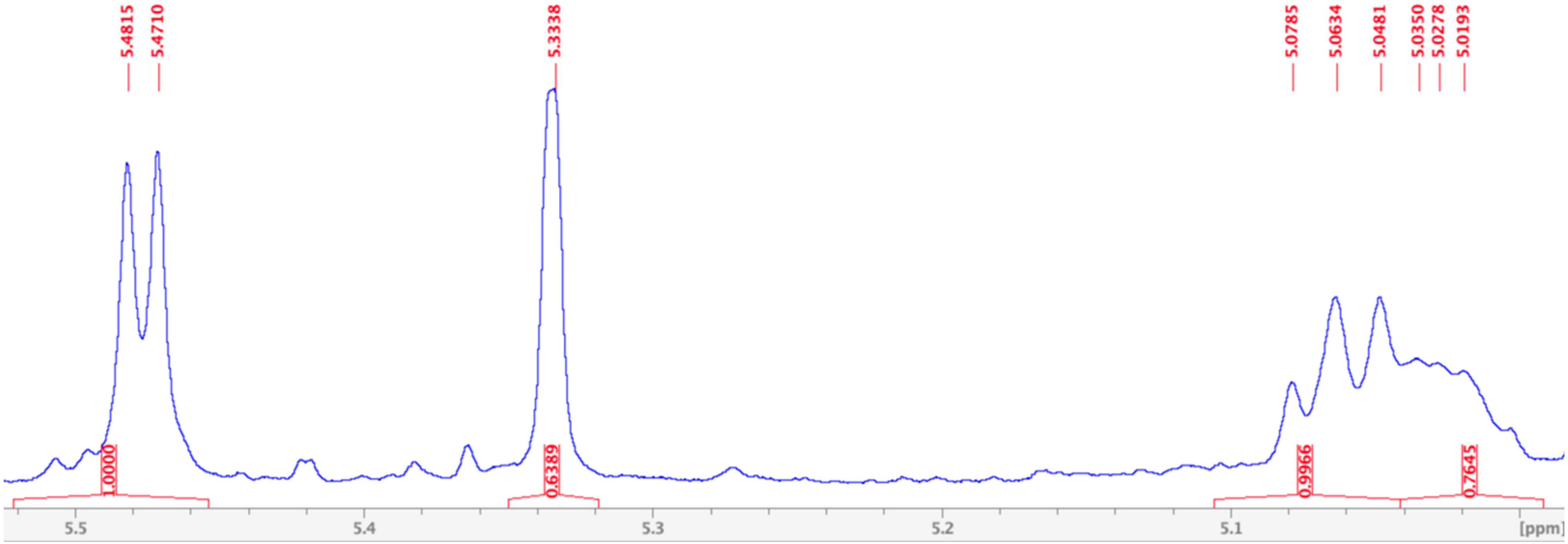

5.4815
5.4710
5.3338
5.0785
5.0634
5.0481
5.0350
5.0278
5.0193
1.0000
0.6389
0.9966
0.7645
5.5
5.4
5.3
5.2
5.1
[ppm]

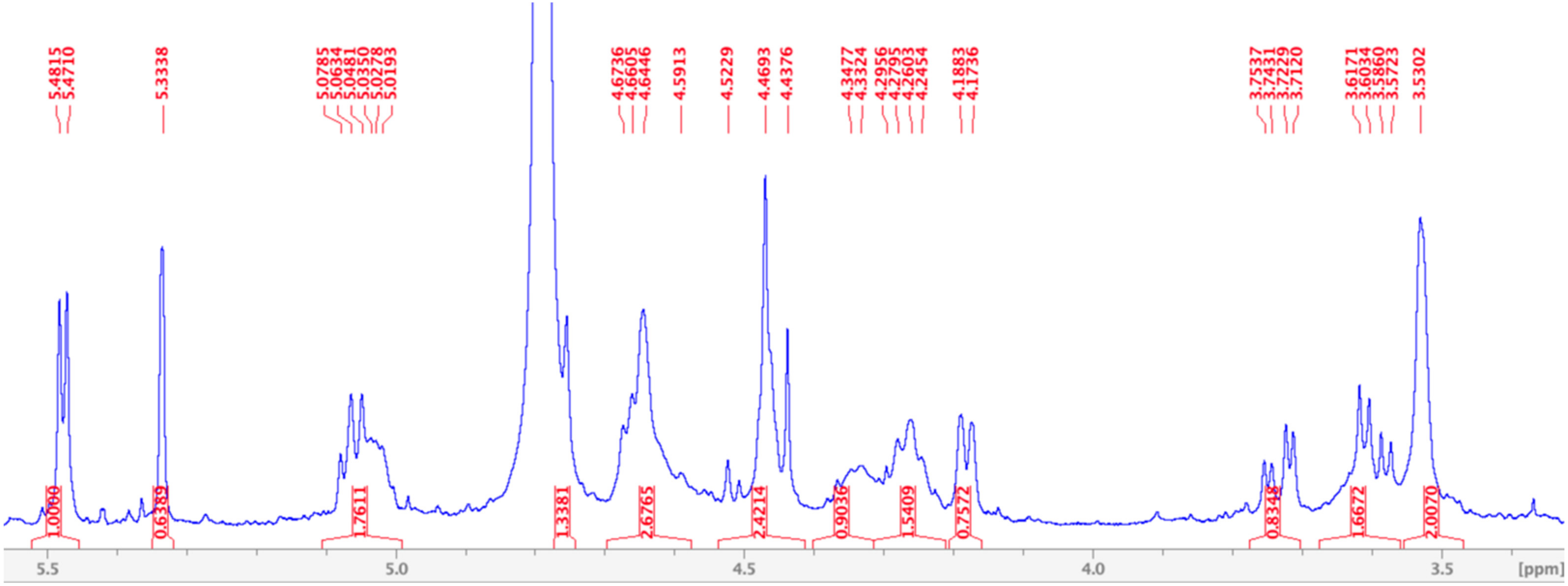
5.4815
5.4710
5.3338
5.0785
5.0634
5.0481
5.0350
5.0278
5.0193
4.6736
4.6605
4.6446
4.5913
4.5229
4.4693
4.4376
4.3477
4.3324
4.2956
4.2795
4.2603
4.2454
4.1883
4.1736
3.7537
3.7431
3.7229
3.7120
3.6171
3.6034
3.5860
3.5723
3.5302
1.0000
0.6389
1.7611
1.3381
2.6765
2.4214
0.9036
1.5409
0.7572
0.8348
1.6672
2.0070
5.5
5.0
4.5
4.0
3.5
[ppm]

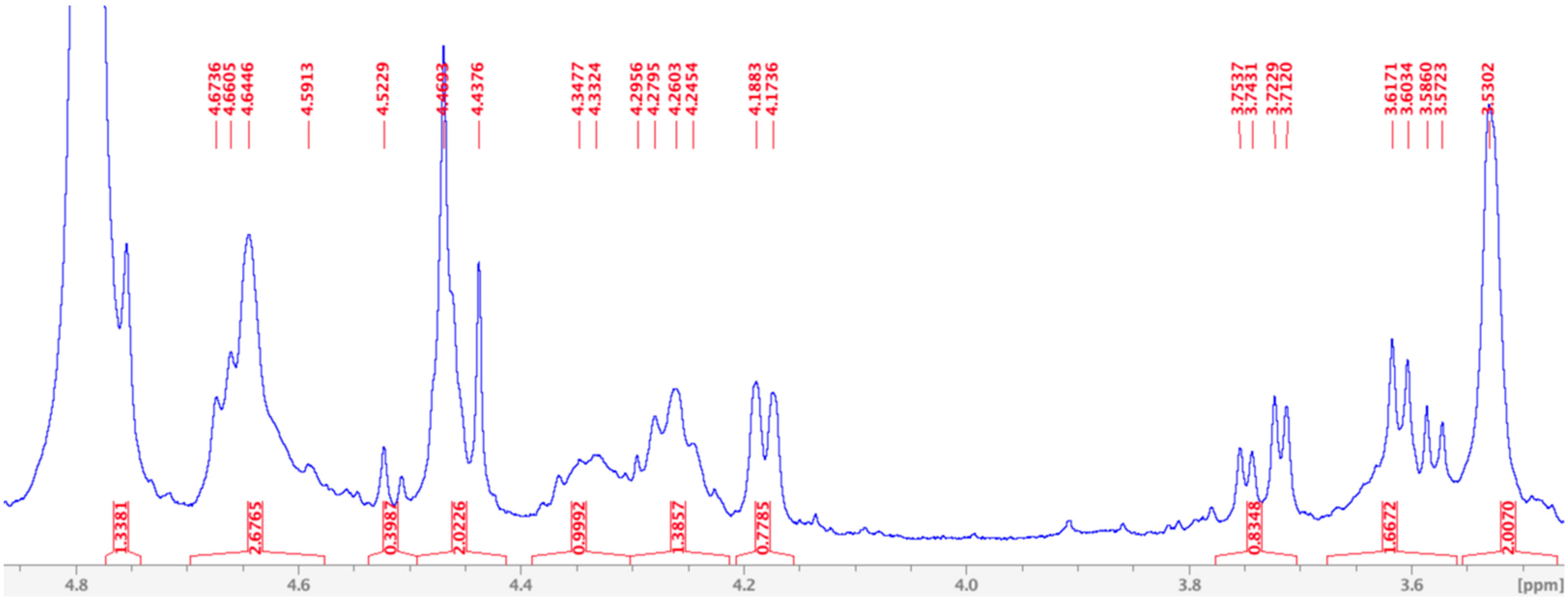
4.6736
4.6605
4.6446
4.5913
4.5229
4.4693
4.4376
4.3477
4.3324
4.2956
4.2795
4.2603
4.2454
4.1883
4.1736
3.7537
3.7431
3.7229
3.7120
3.6171
3.6034
3.5860
3.5723
3.5302
1.3381
2.6765
0.3987
2.0226
0.9992
1.3857
0.7785
0.8348
1.6672
2.0070
4.8
4.6
4.4
4.2
4.0
3.8
3.6
[ppm]

^{13}C-NMR

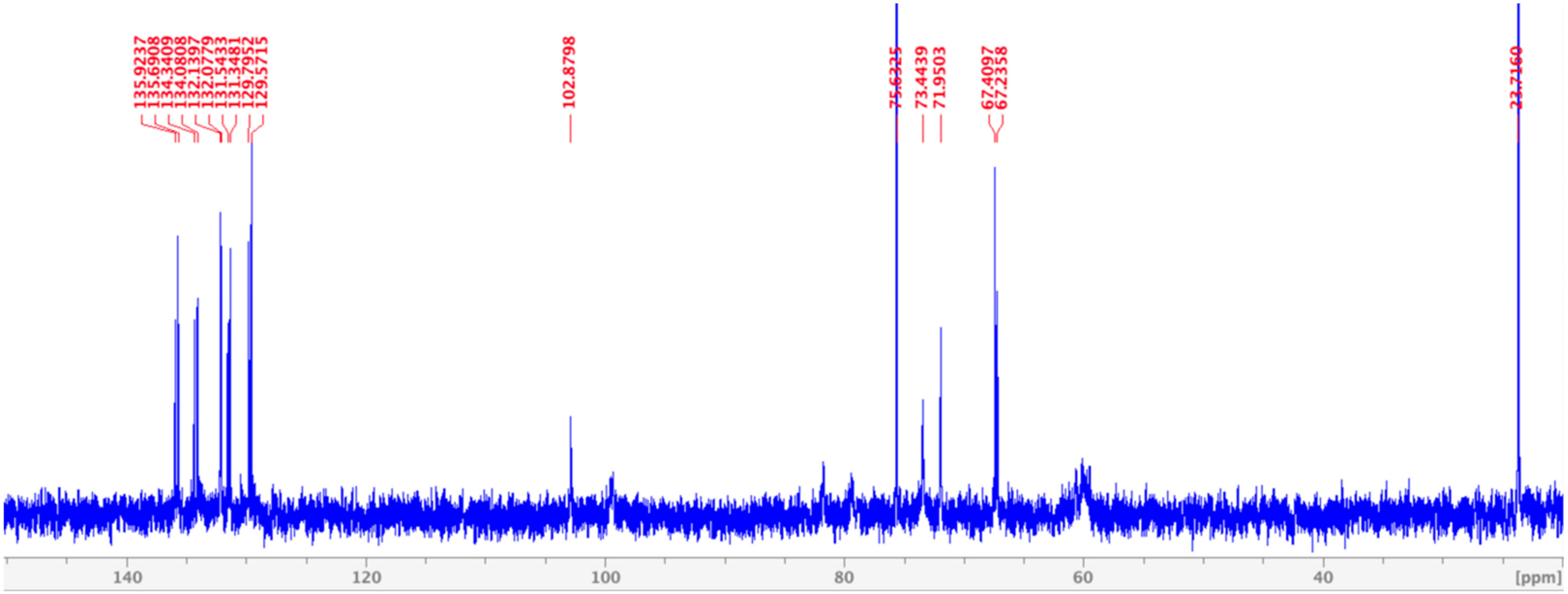

135.9237
135.6908
134.3409
134.0808
132.1397
132.0779
131.5433
131.3481
129.7952
129.5715
102.8798
75.6325
73.4439
71.9503
67.4097
67.2358
23.7160
140
120
100
80
60
40
[ppm]

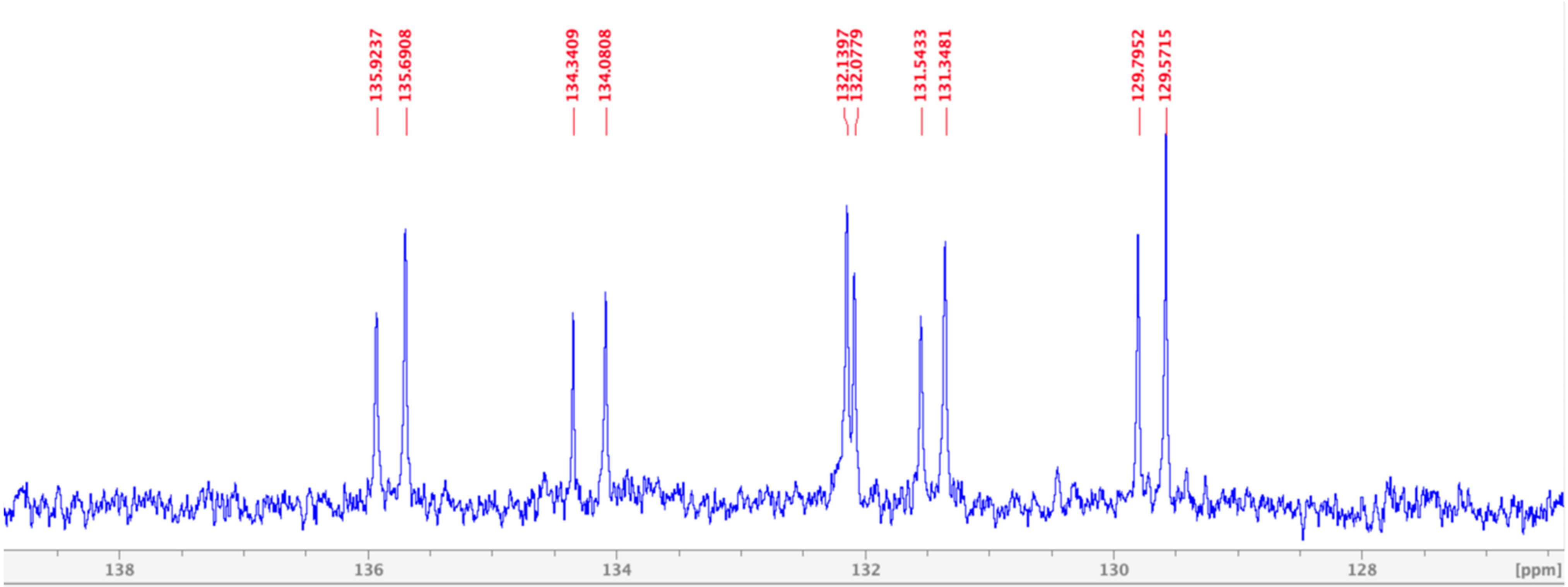
135.9237
135.6908
134.3409
134.0808
132.1397
132.0779
131.5433
131.3481
129.7952
129.5715
138
136
134
132
130
128
[ppm]

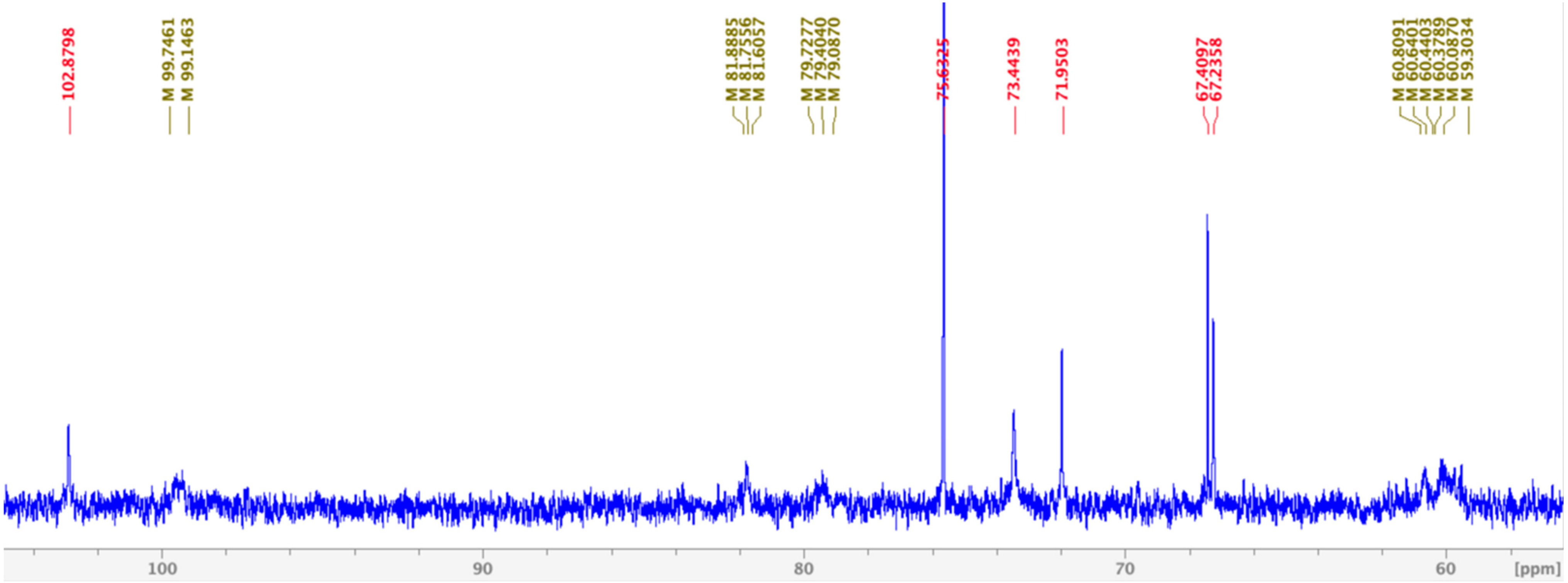
102.8798
M 99.7461
M 99.1463
M 81.8885
M 81.7556
M 81.6057
M 79.7277
M 79.4040
M 79.0870
75.6325
73.4439
71.9503
67.4097
67.2358
M 60.8091
M 60.6401
M 60.4403
M 60.3789
M 60.0870
M 59.3034
100
90
80
70
60
[ppm]

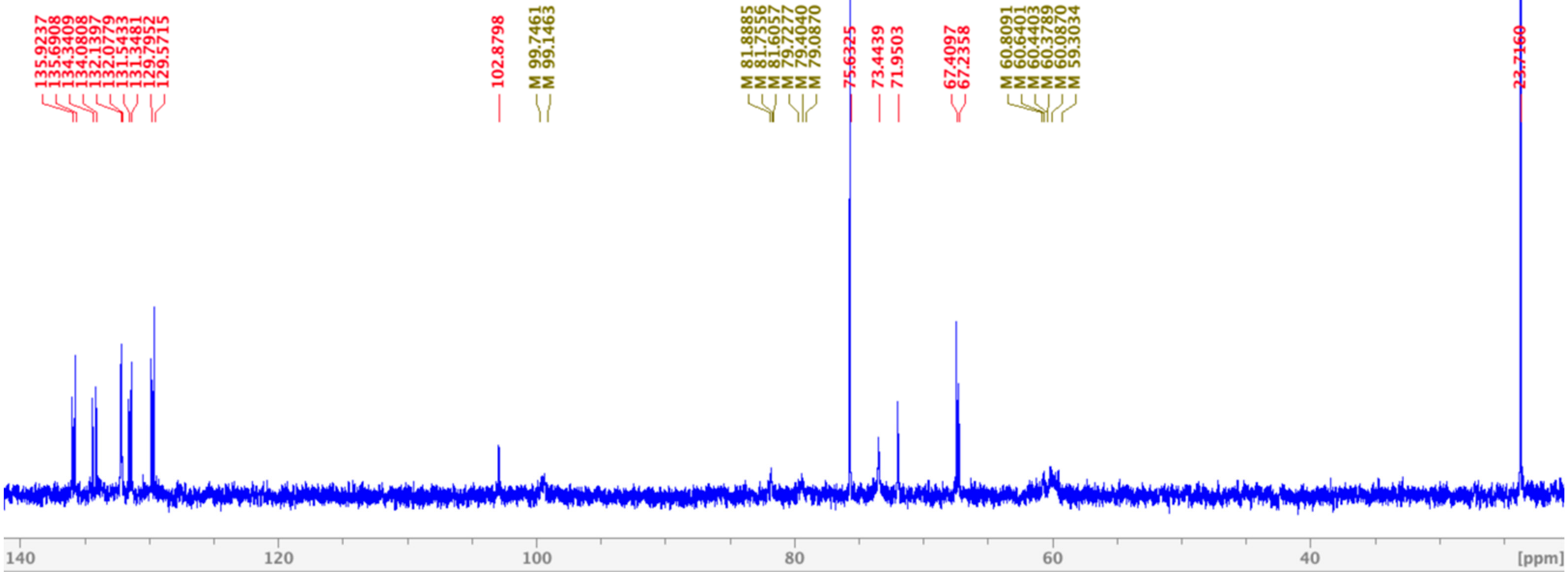
135.9237
135.6908
134.3409
134.0808
132.1397
132.0779
131.5433
131.3481
129.7952
129.5715
102.8798
M 99.7461
M 99.1463
M 81.8885
M 81.7556
M 81.6057
M 79.7277
M 79.4040
M 79.0870
75.6325
73.4439
71.9503
67.4097
67.2358
M 60.8091
M 60.6401
M 60.4403
M 60.3789
M 60.0870
M 59.3034
23.7160
140
120
100
80
60
40
[ppm]

DEPT

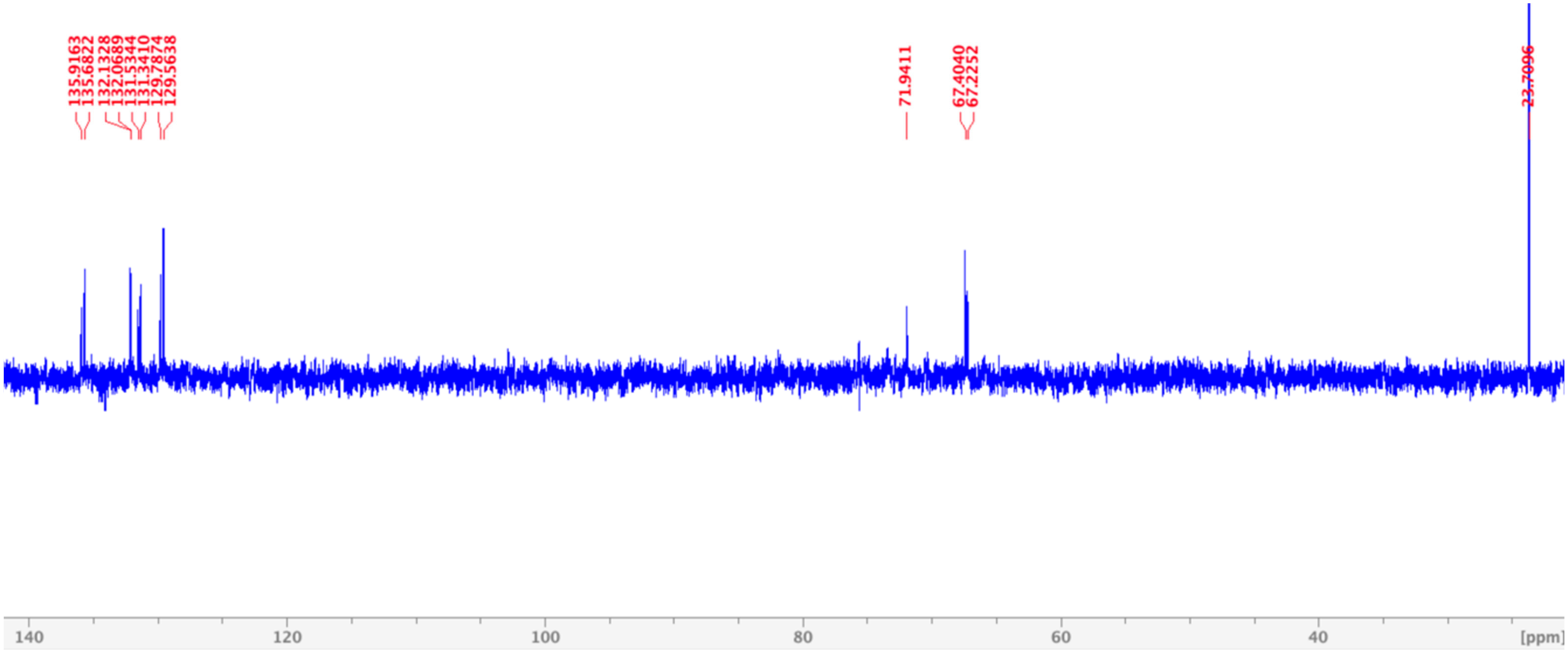

135.9163
135.6822
132.1328
132.0689
131.5344
131.3410
129.7874
129.5638
71.9411
67.4040
67.2252
23.7096
140
120
100
80
60
40
[ppm]

^{11}B-NMR

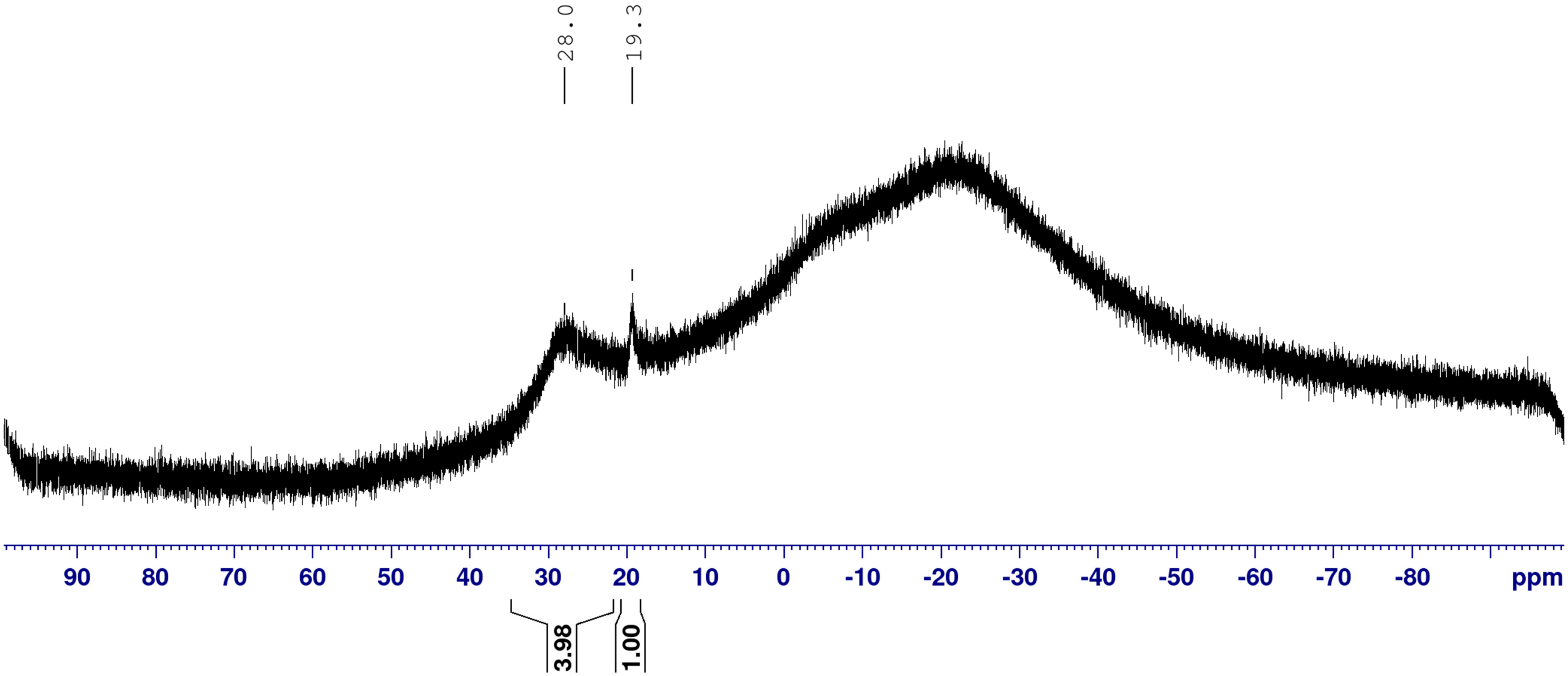
28.0
19.3
90
80
70
60
50
40
30
20
10
0
-10
-20
-30
-40
-50
-60
-70
-80
ppm
3.98
1.00

COSY

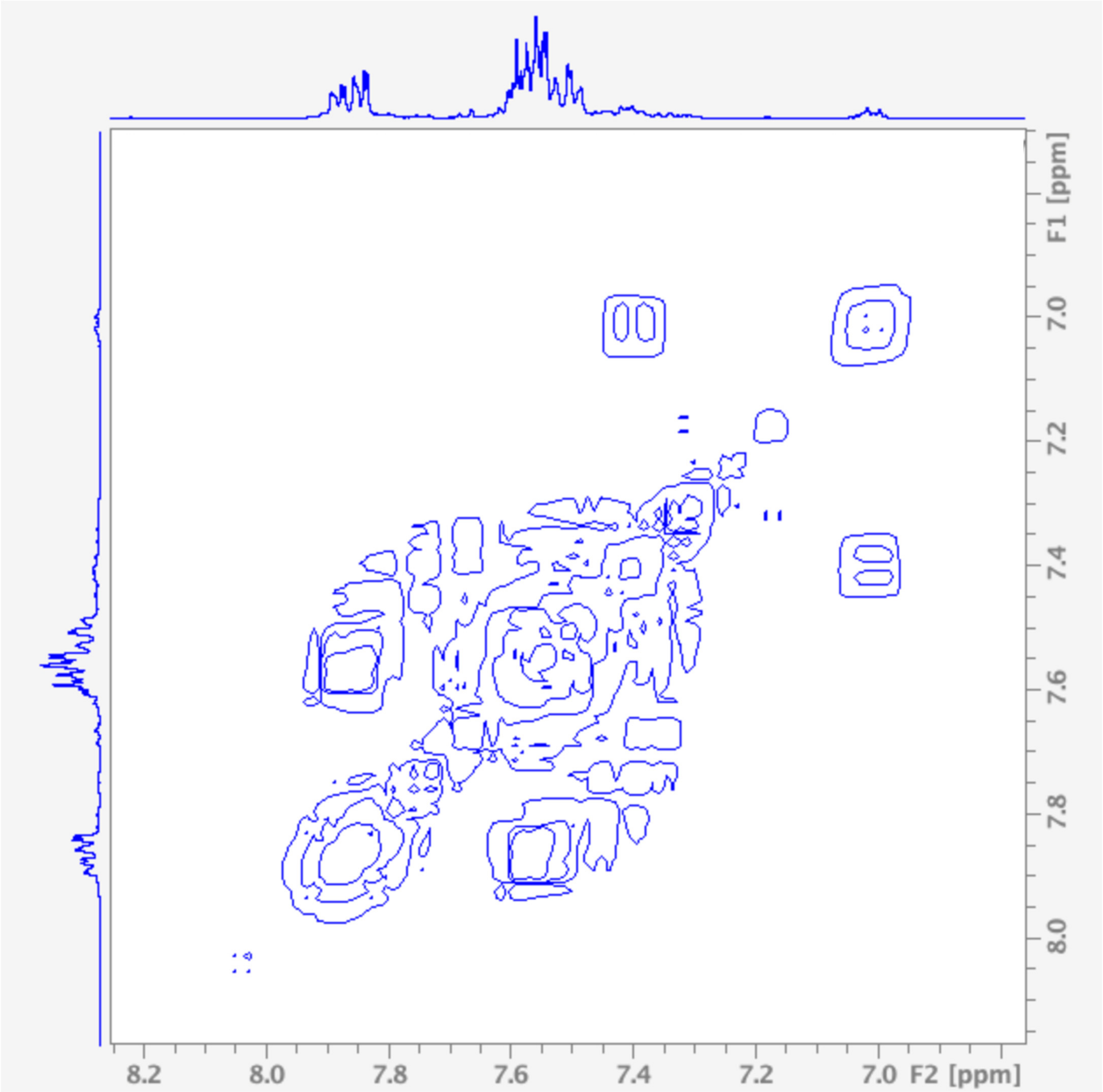

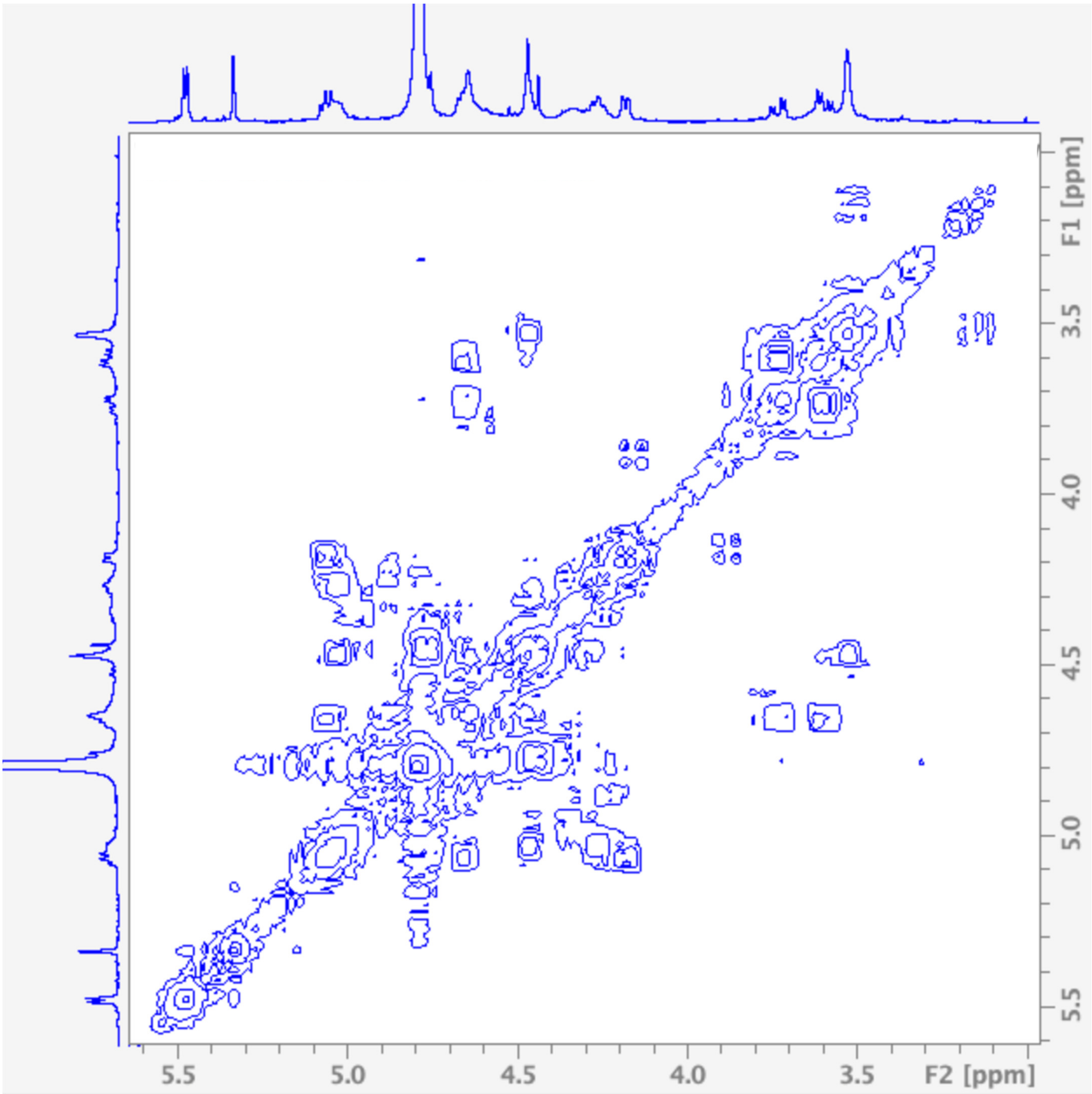
F1 [ppm]
3.5
4.0
4.5
5.0
5.5
5.5
5.0
4.5
4.0
3.5
F2 [ppm]

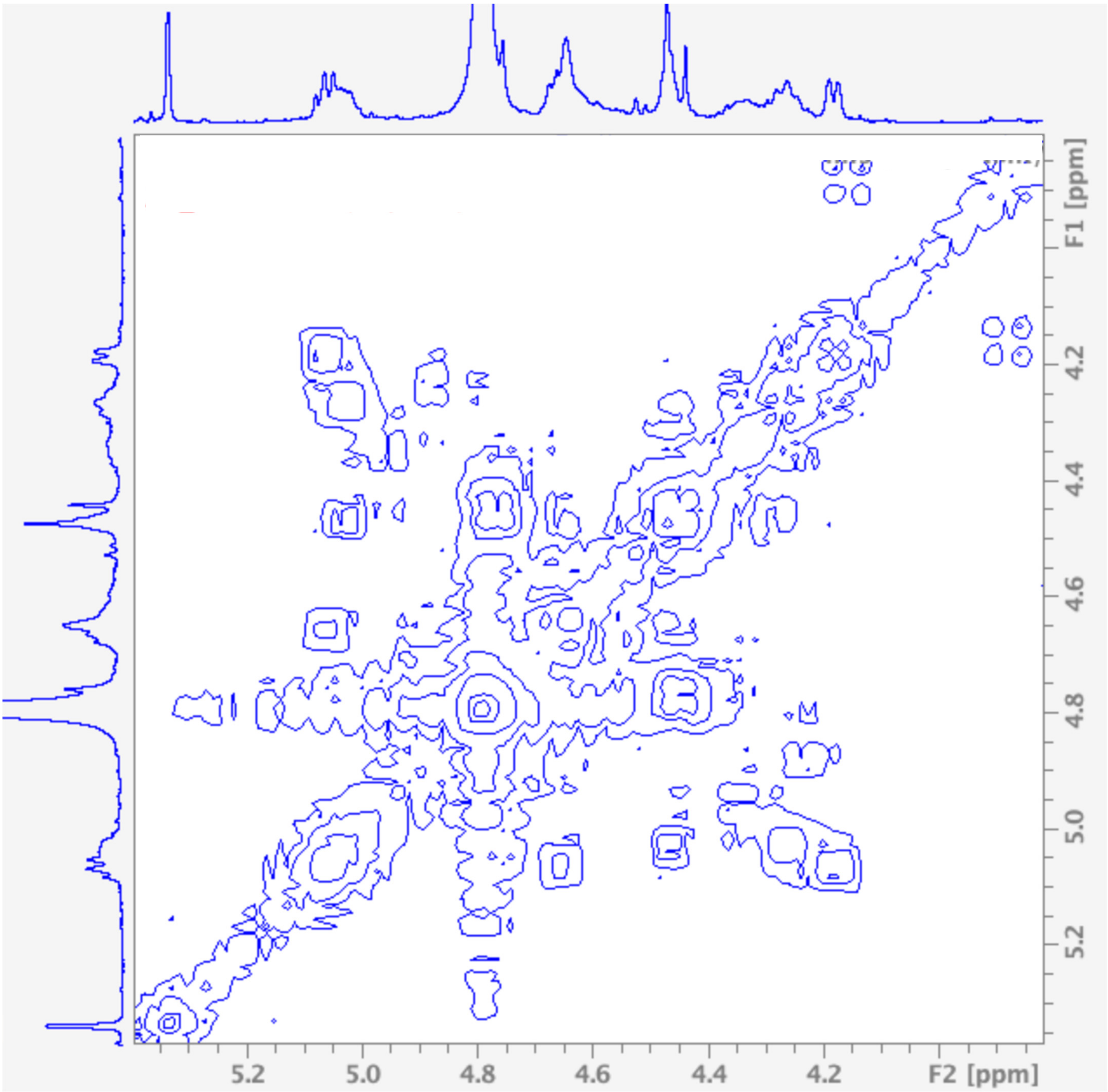

F1 [ppm]
4.2
4.4
4.6
4.8
5.0
5.2
5.2
5.0
4.8
4.6
4.4
4.2
F2 [ppm]

HSQC

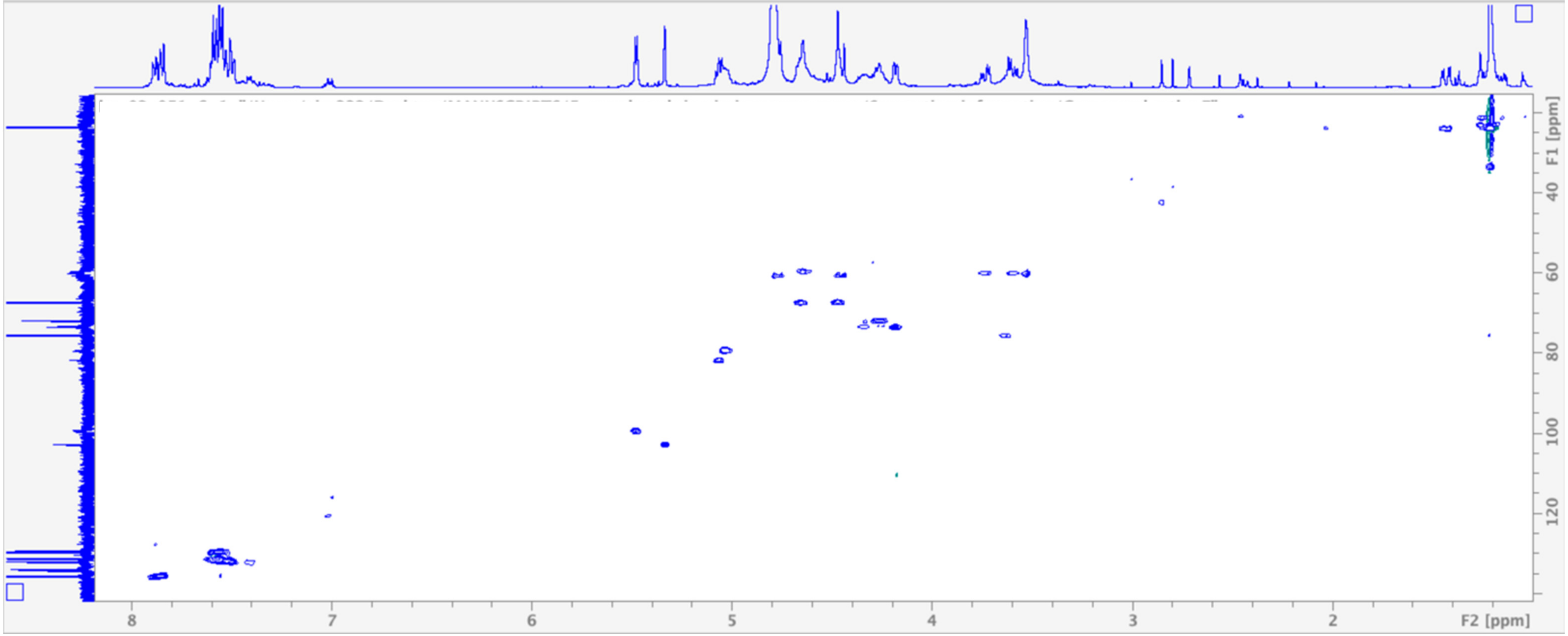

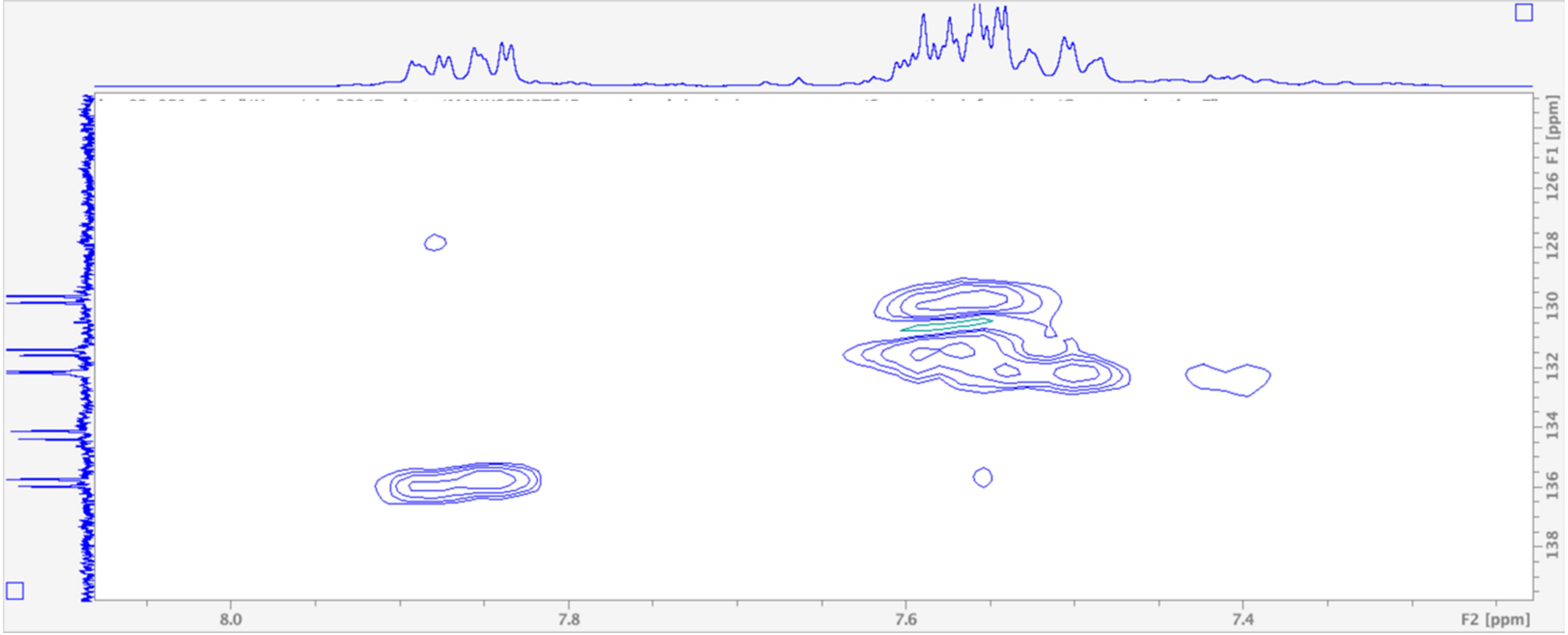
F1 [ppm]
126
128
130
132
134
136
138
8.0
7.8
7.6
7.4
F2 [ppm]

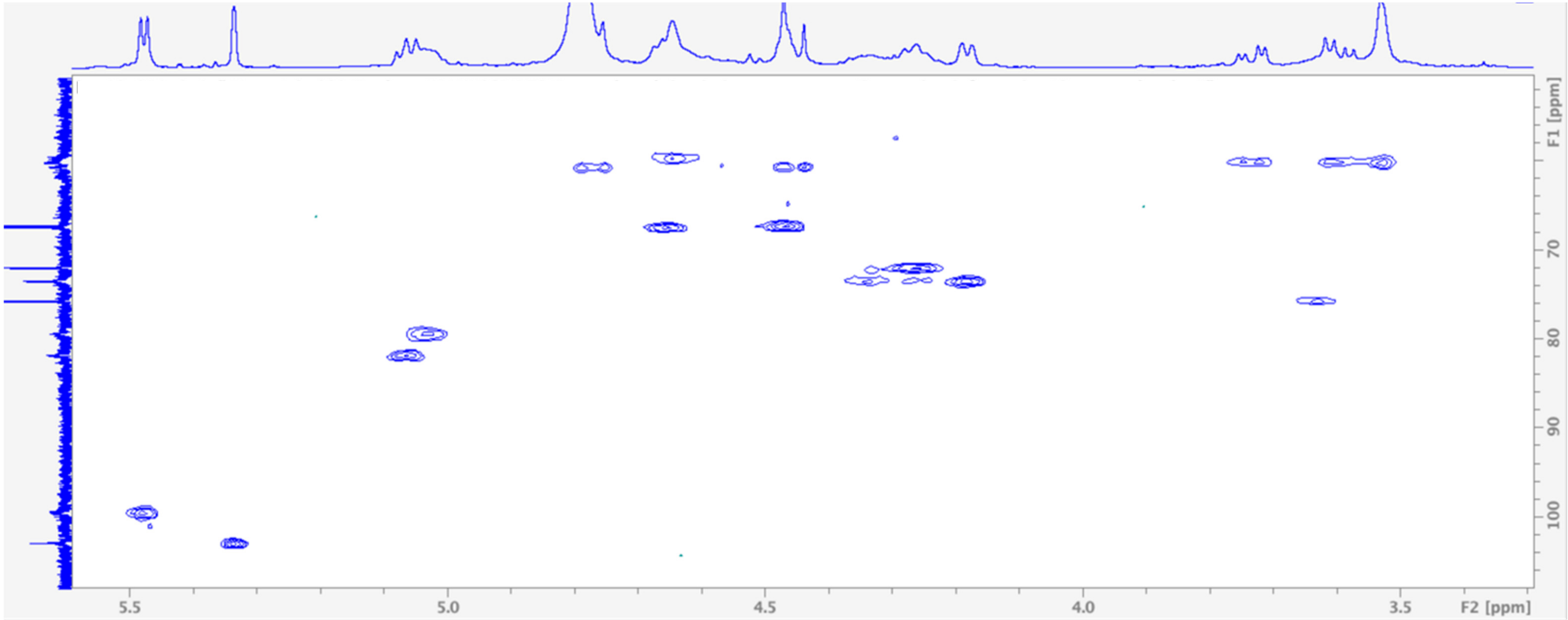

F1 [ppm]
70
80
90
100
5.5
5.0
4.5
4.0
3.5
F2 [ppm]

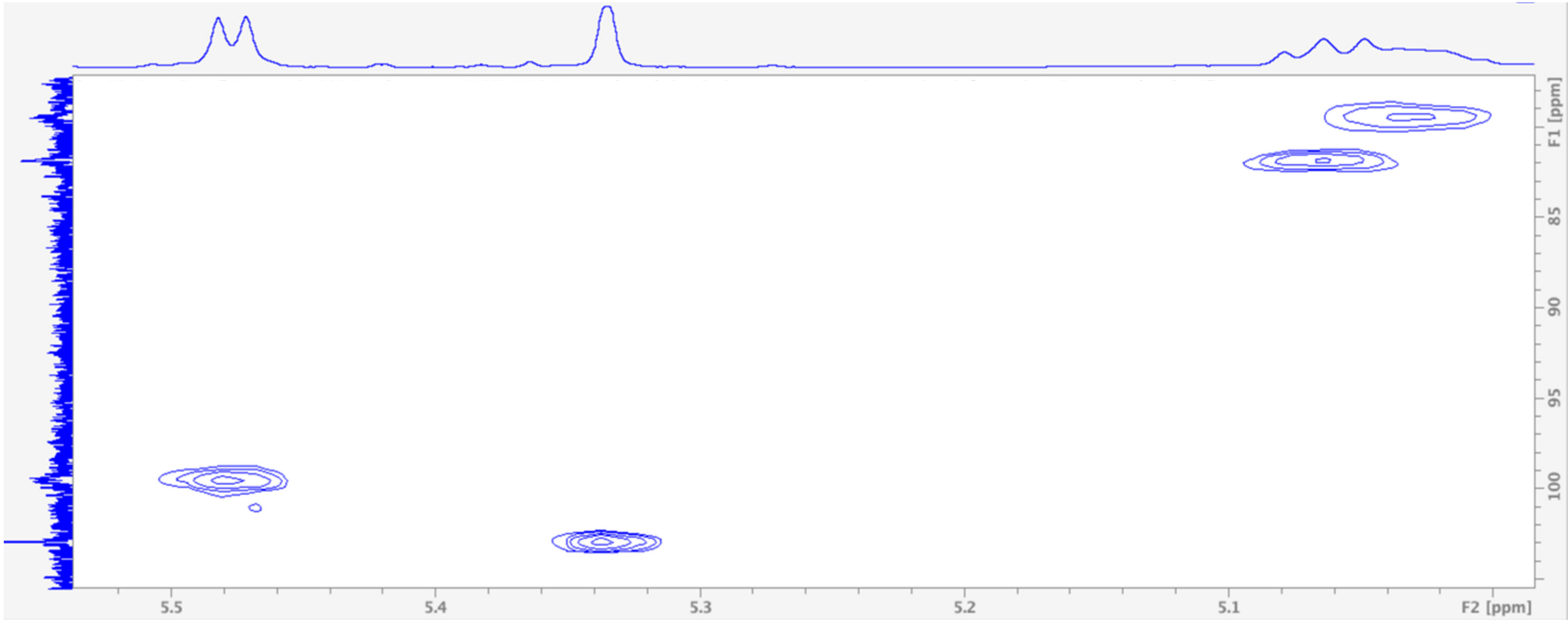

F1 [ppm]
85
90
95
100
5.5
5.4
5.3
5.2
5.1
F2 [ppm]

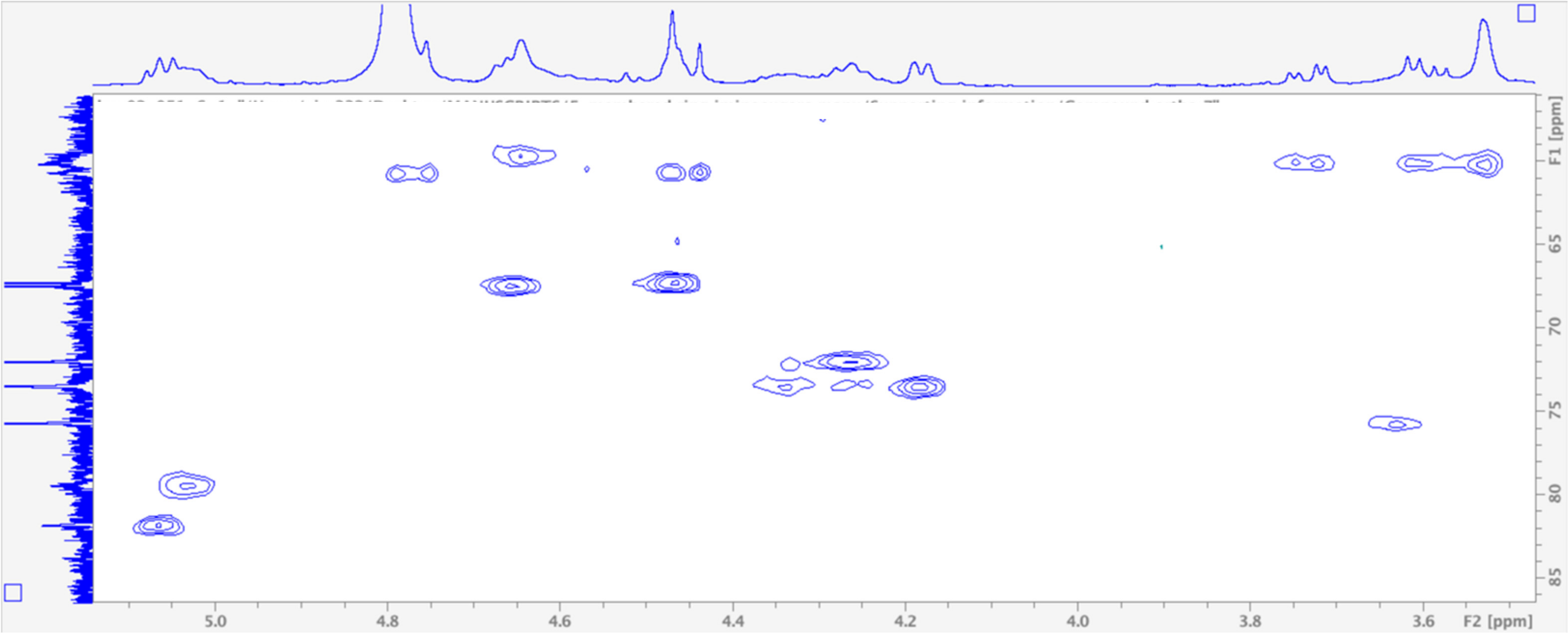
F1 [ppm]
65
70
75
80
85
5.0
4.8
4.6
4.4
4.2
4.0
3.8
3.6
F2 [ppm]

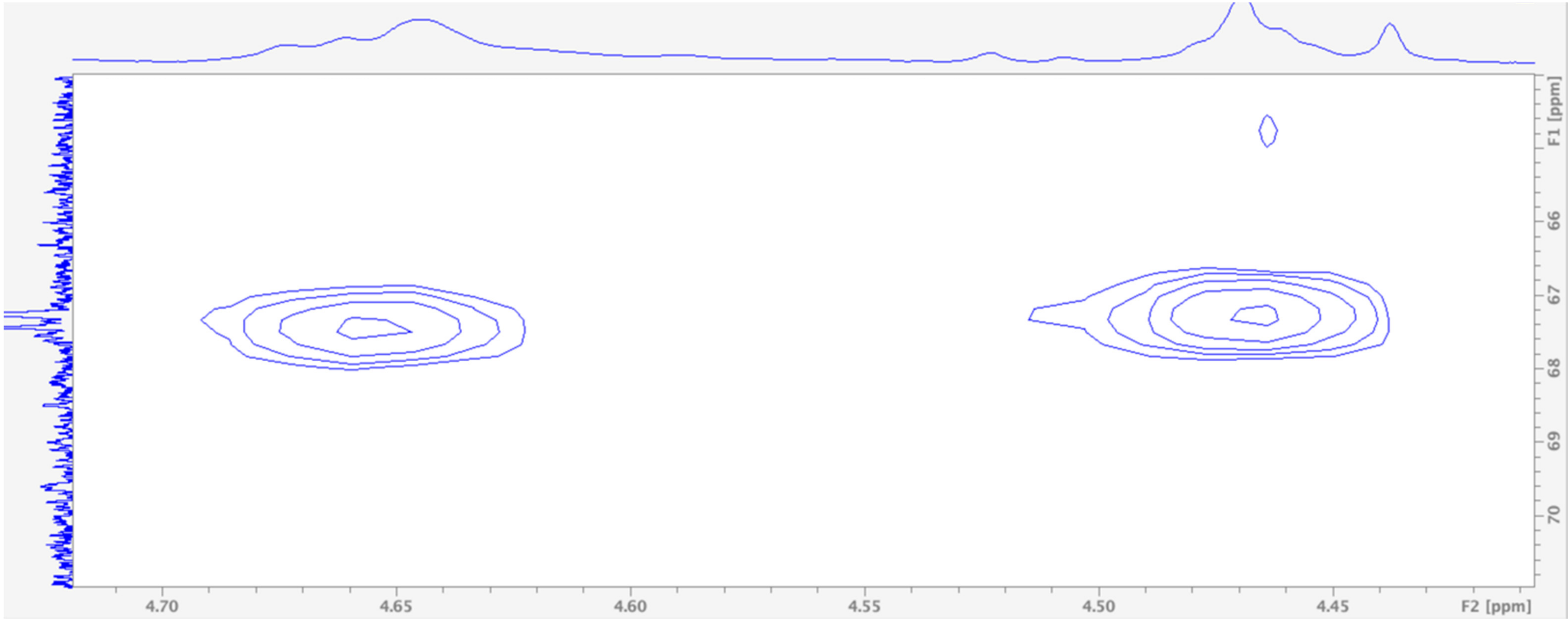
F1 [ppm]
66
67
68
69
70
4.70
4.65
4.60
4.55
4.50
4.45
F2 [ppm]

HMBC

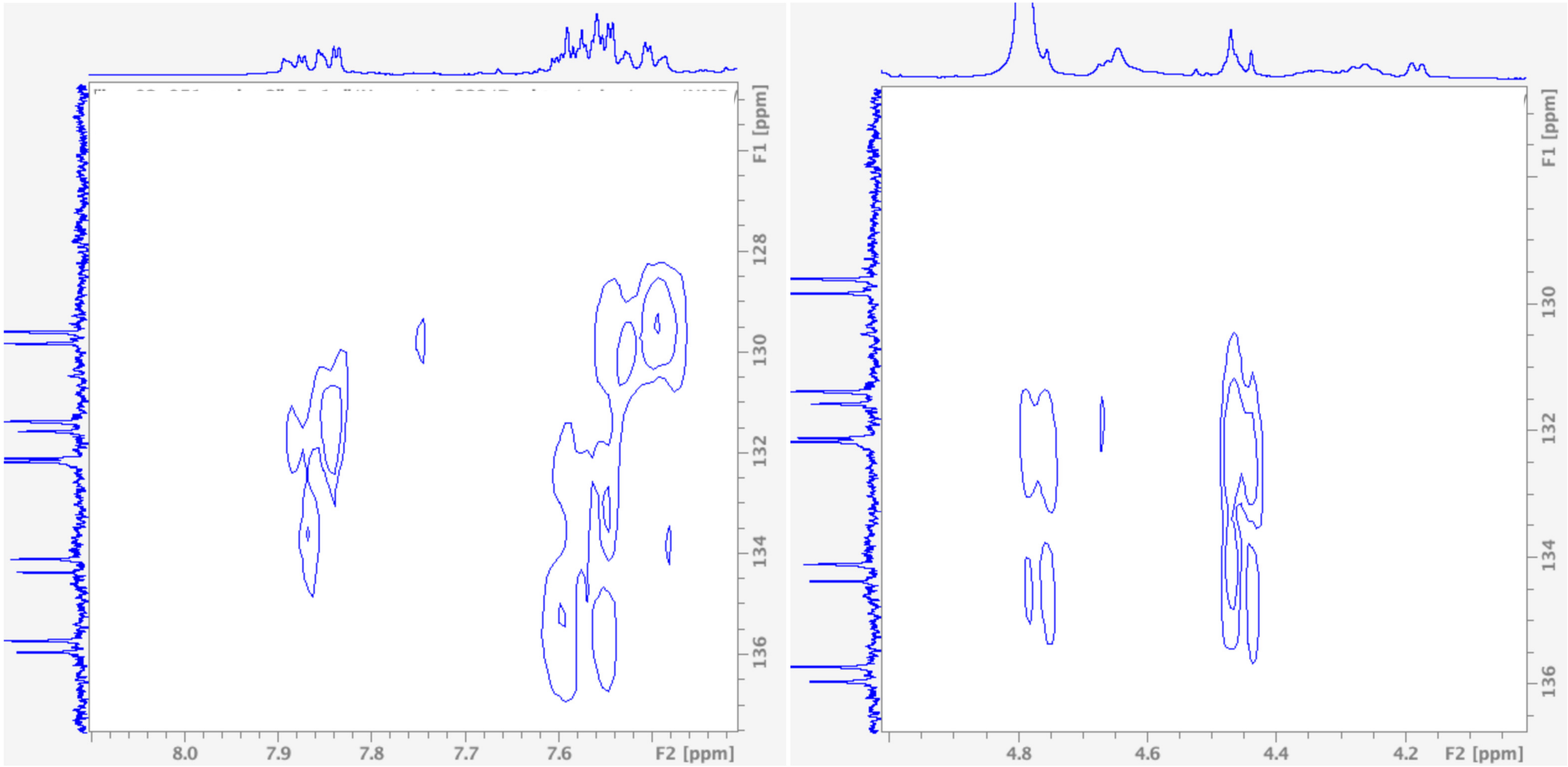

F1 [ppm]
128
130
132
134
136
8.0
7.9
7.8
7.7
7.6
F2 [ppm]
F1 [ppm]
130
132
134
136
4.8
4.6
4.4
4.2
F2 [ppm]

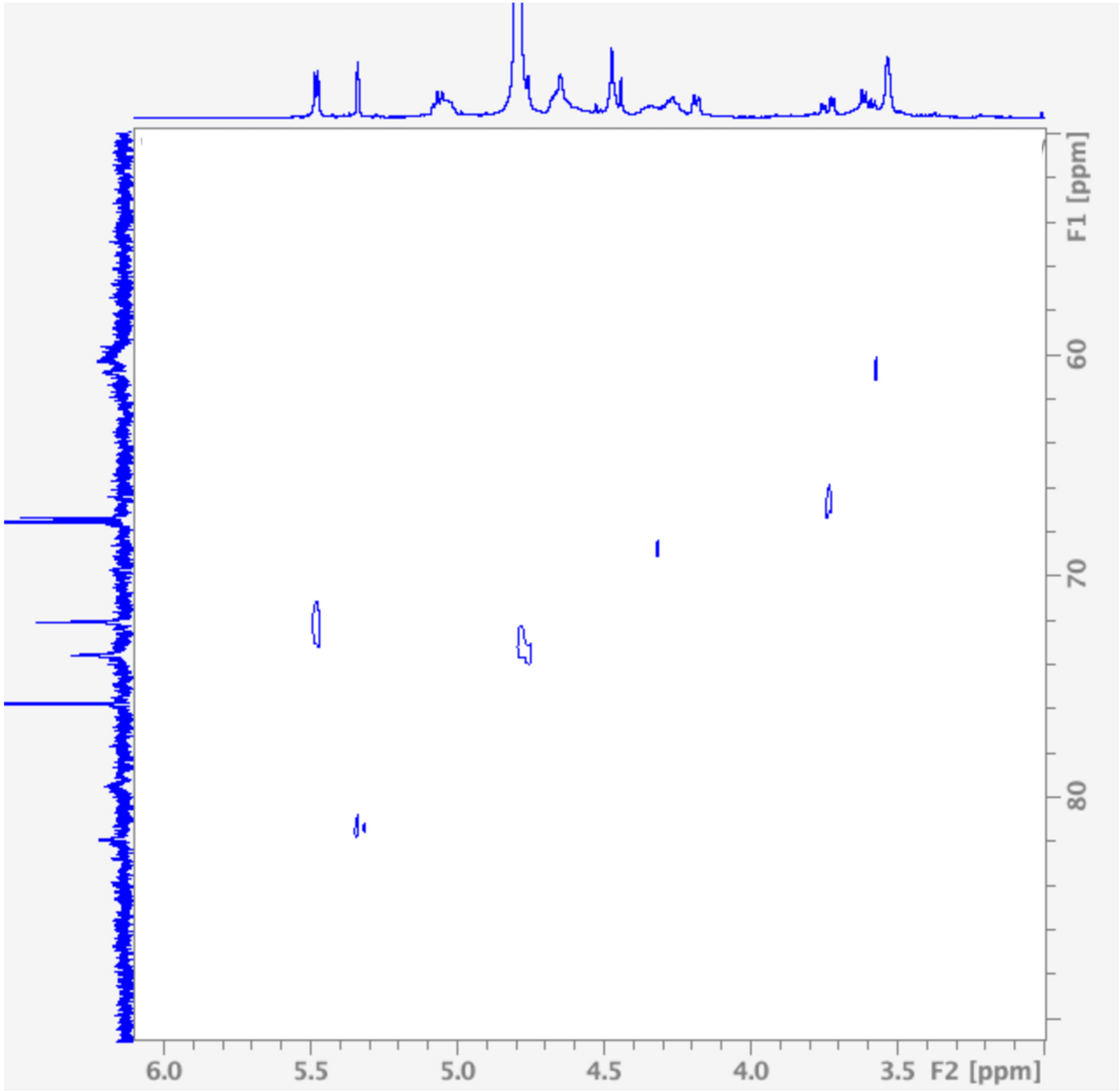
F1 [ppm]
60
70
80
6.0
5.5
5.0
4.5
4.0
3.5
F2 [ppm]

Figure S8. ^{1}H-NMR spectrum (400 MHz, D_2O) of intermediate **ortho 3** with colour-coded signals, highlighting the furanose anomeric forms they belong to, with interpretation of the isolated signals and tentative interpretation of the overlapping ones. Namely, the orange designates the *α-fur* form and indigo designates the *β-fur* form. A) section 7.93 ppm to 7.47 ppm; B) section 5.57 ppm to 4.54 ppm; C) section 4.54 ppm to 3.40 ppm. Highlighted are also the principal COSY correlations to hydrogen atoms within the same spin systems.

A

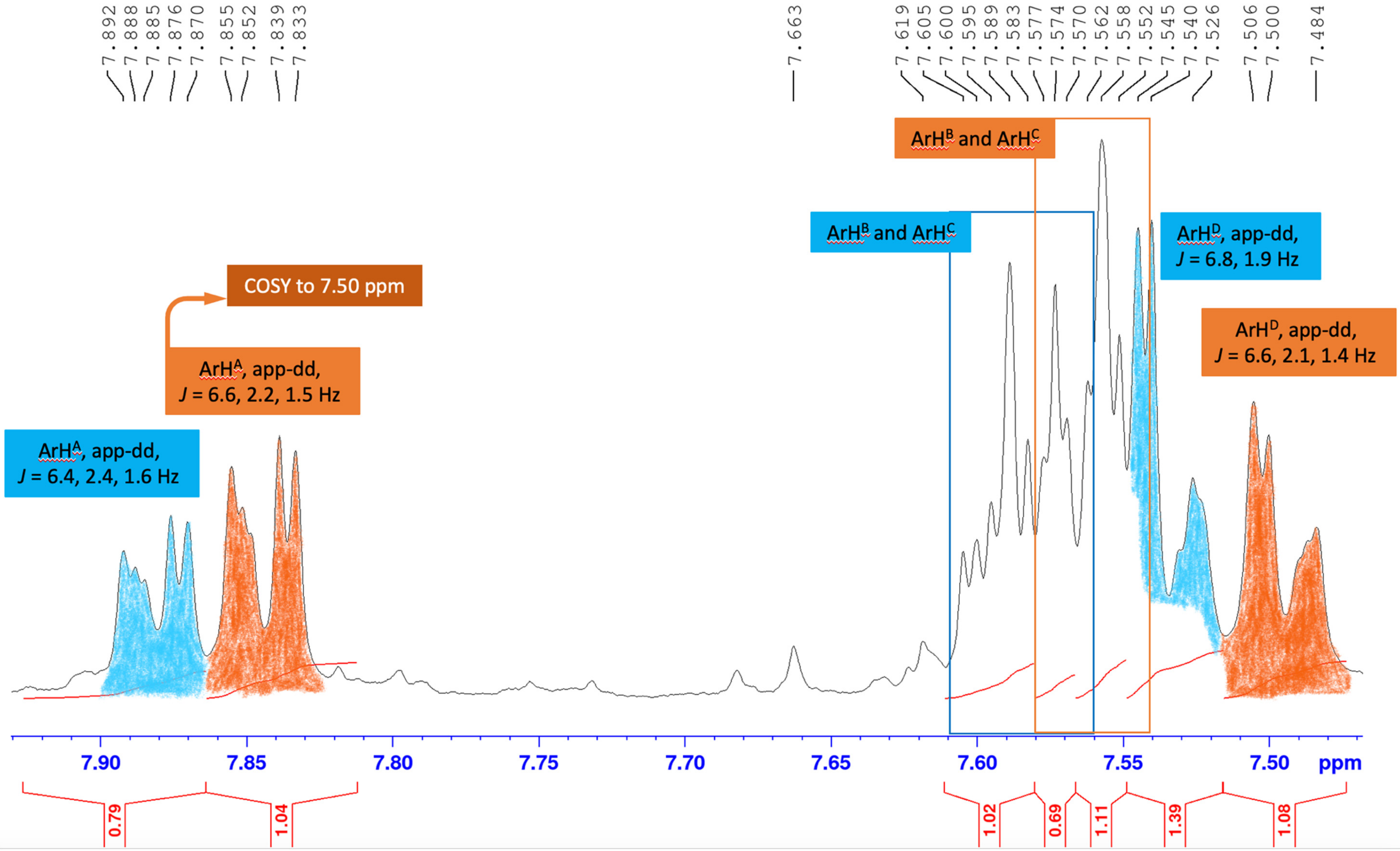

B

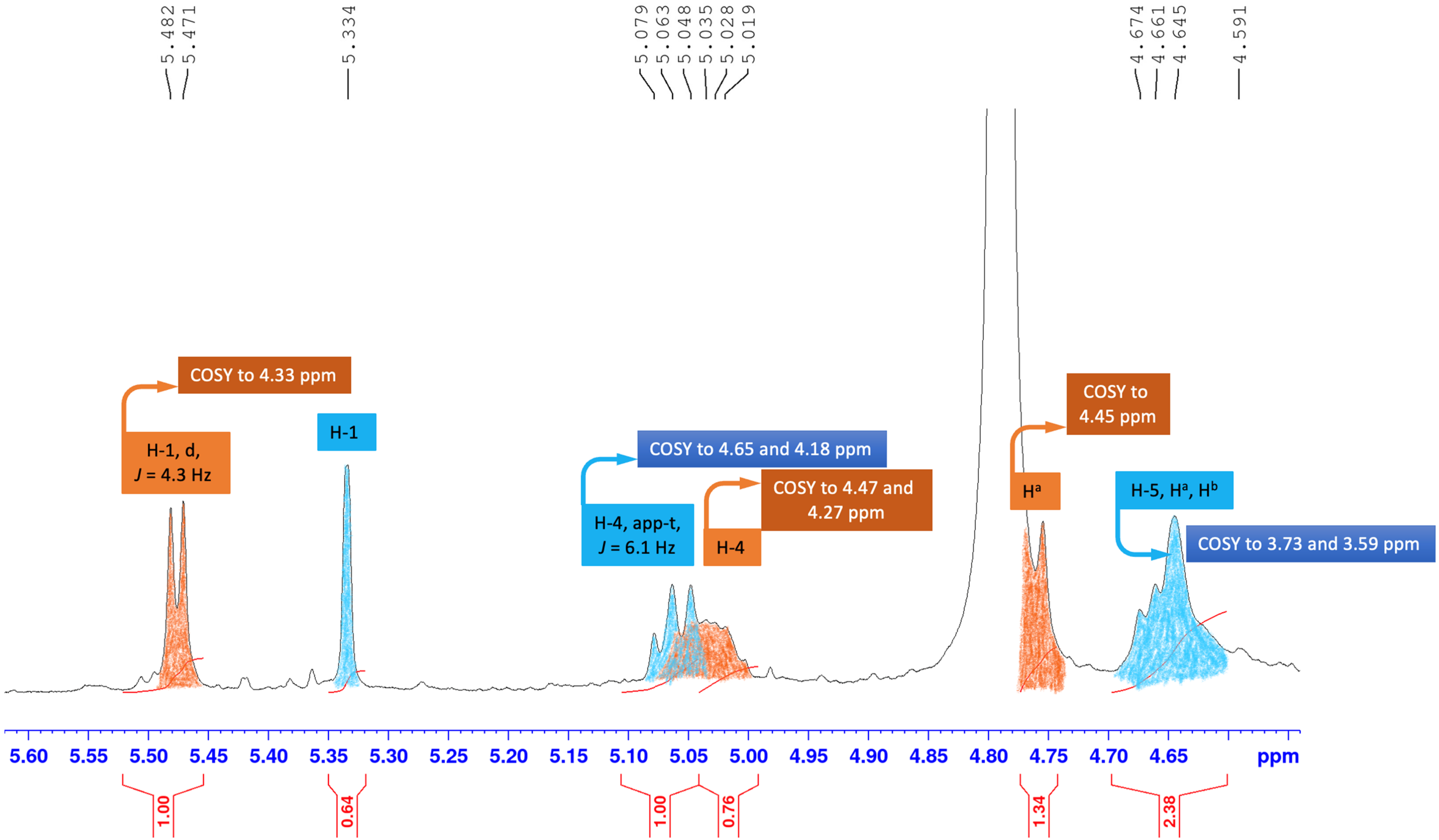
5.482
5.471
5.334
5.079
5.063
5.048
5.035
5.028
5.019
4.674
4.661
4.645
4.591
COSY to 4.33 ppm
H-1, d,
J = 4.3 Hz
H-1
COSY to 4.65 and 4.18 ppm
H-4, app-t,
J = 6.1 Hz
COSY to 4.47 and 4.27 ppm
H-4
COSY to 4.45 ppm
Ha
H-5, Ha, Hb
COSY to 3.73 and 3.59 ppm
5.60 5.55 5.50 5.45 5.40 5.35 5.30 5.25 5.20 5.15 5.10 5.05 5.00 4.95 4.90 4.85 4.80 4.75 4.70 4.65 ppm
1.00
0.64
1.00
0.76
1.34
2.38

c

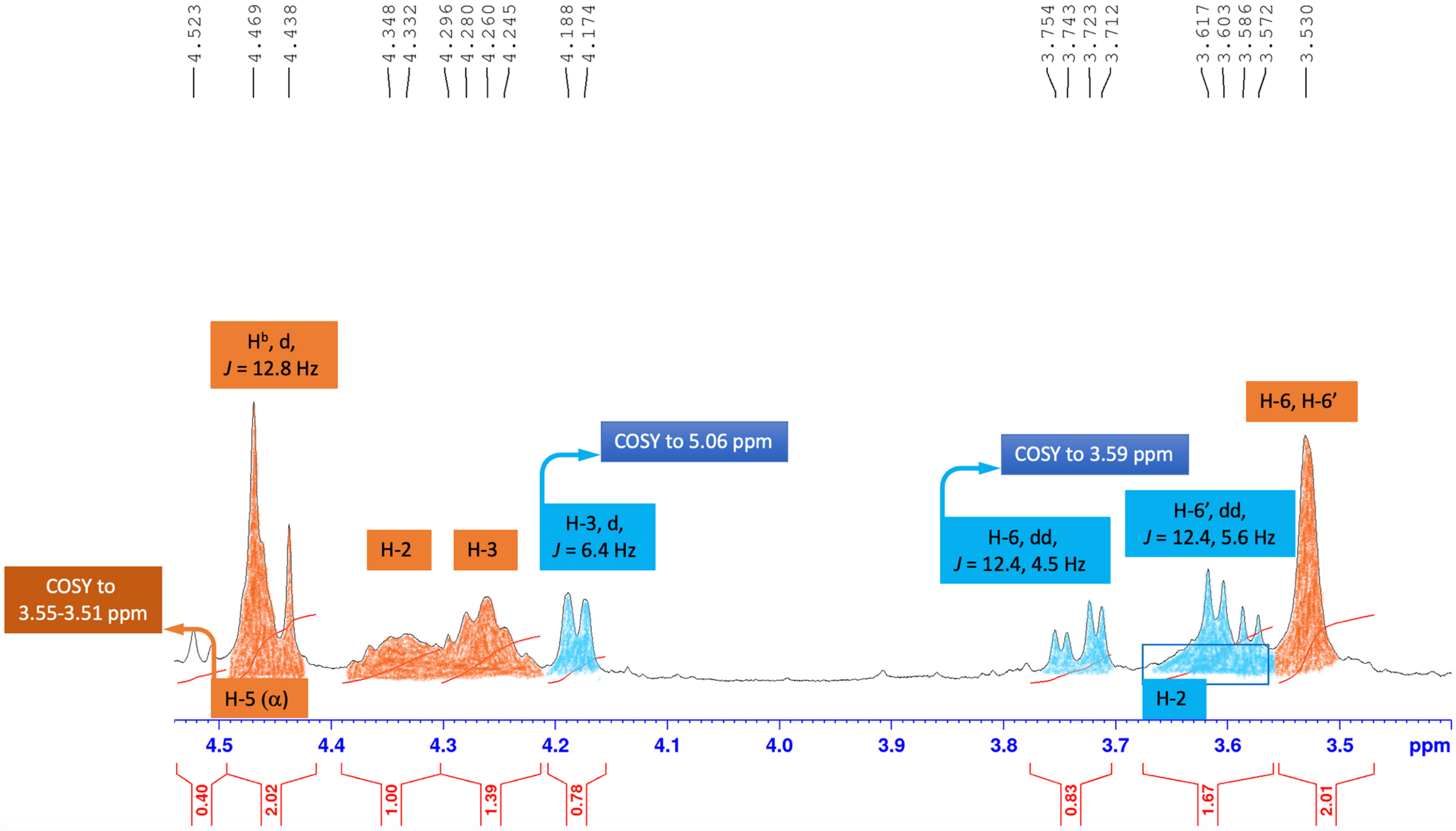
4.523
4.469
4.438
4.348
4.332
4.296
4.280
4.260
4.245
4.188
4.174
3.754
3.743
3.723
3.712
3.617
3.603
3.586
3.572
3.530
Hb, d,
J = 12.8 Hz
COSY to 5.06 ppm
COSY to 3.59 ppm
H-6, H-6'
H-3, d,
J = 6.4 Hz
H-6, dd,
J = 12.4, 4.5 Hz
H-6', dd,
J = 12.4, 5.6 Hz
H-2
H-3
COSY to
3.55-3.51 ppm
H-5 (α)
H-2
4.5
4.4
4.3
4.2
4.1
4.0
3.9
3.8
3.7
3.6
3.5
ppm
0.40
2.02
1.00
1.39
0.78
0.83
1.67
2.01

Figure S9. ^{13}C-NMR spectrum (100 MHz, D_2O) sections of intermediate **ortho 3** with colour-coded signals, highlighting the furanose anomeric forms they belong to, with interpretation of the isolated signals and tentative interpretation of the overlapping ones. Namely, the orange designates the *α-fur* form and indigo designates the *β-fur* form. A) section 100.5 ppm to 79 ppm; B) section 105.0 ppm to 90.0 ppm; C) section 78.0 ppm to 58.0 ppm. Highlighted are also the principal HSQC correlations to hydrogen atoms within the same spin systems.

A

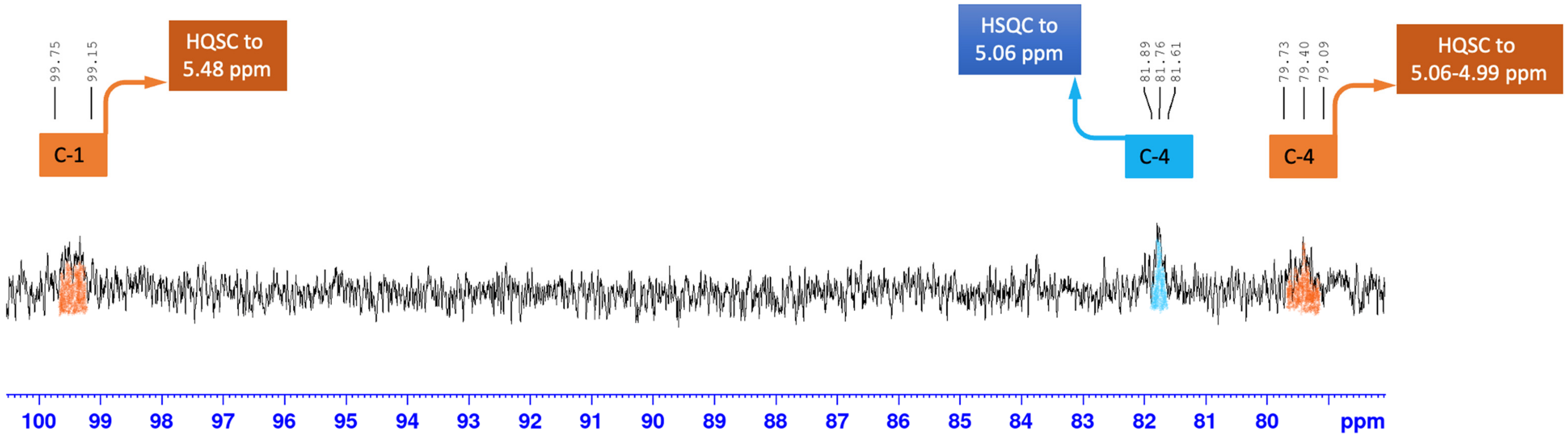

B

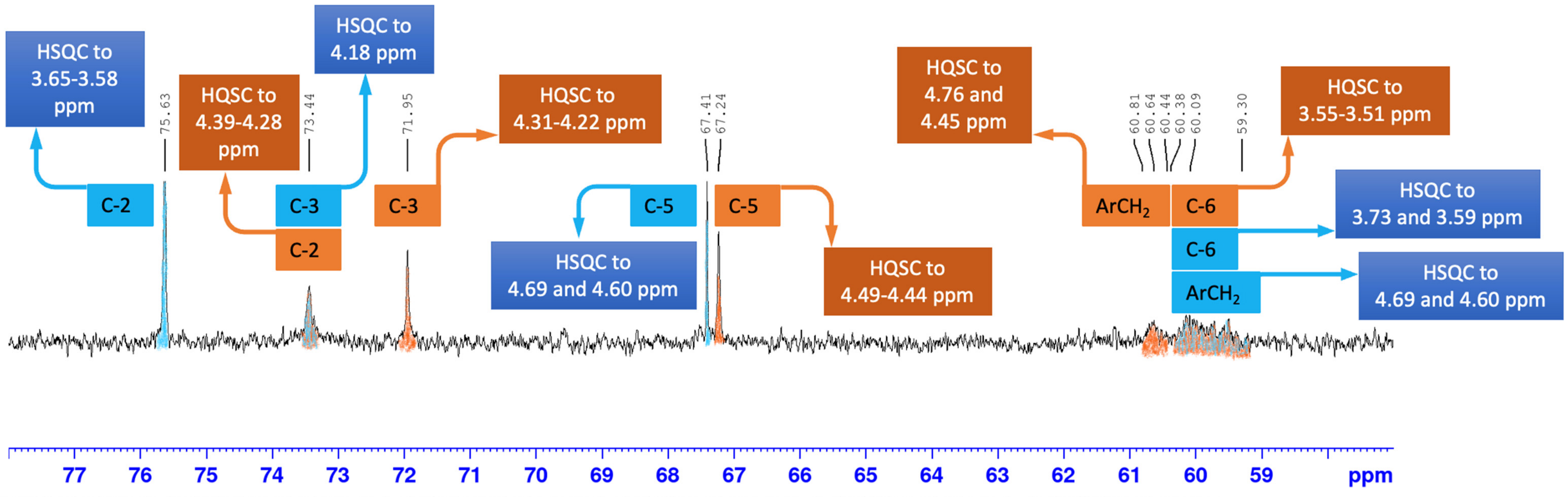
HSQC to 3.65-3.58 ppm
75.63
HQSC to 4.39-4.28 ppm
73.44
HSQC to 4.18 ppm
71.95
HQSC to 4.31-4.22 ppm
67.41
67.24
HQSC to 4.76 and 4.45 ppm
60.81
60.64
60.44
60.38
60.09
59.30
HQSC to 3.55-3.51 ppm
C-2
C-3
C-2
C-3
C-5
C-5
ArCH2
C-6
HSQC to 3.73 and 3.59 ppm
C-6
ArCH2
HSQC to 4.69 and 4.60 ppm
HQSC to 4.49-4.44 ppm
HSQC to 4.69 and 4.60 ppm
77 76 75 74 73 72 71 70 69 68 67 66 65 64 63 62 61 60 59 ppm

Figure S10. ^{1}H- (400 MHz), ^{13}C-NMR (100 MHz), DEPT, ^{11}B-NMR (128 MHz), COSY, HSQC and HMBC spectra of *N*-(2-methylphenyl boronic acid)-1,4-dideoxy-1,4-imino-L-gulitol **ortho 4** in D_2O.

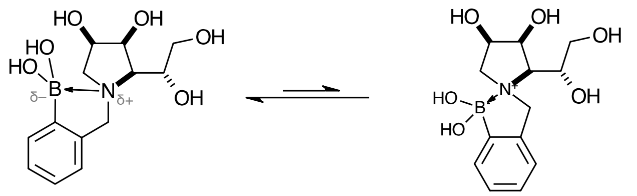

Boronate ammonium species (partially tetrahedral). **δ_H**: 7.48 (1H, app-dd, *J* 5.6, 2.4 Hz, ArH^{B}), 7.33 (1H, ddd, partially obscured, *J* 7.6, 5.6, 2.0 Hz, ArH^{C}), 7.31 (1H, ddd, partially obscured, *J* 7.4, 5.8, 2.1 Hz, ArH^{D}), 7.17 (1H, app-dd, *J* 5.8, 2.2Hz, ArH^{E}), 4.60 (1H, app-dd, *J* 10.9 Hz, $J_{H\text{-}2,H\text{-}1'}$ 5.8 Hz, H-2), 4.53-4.45 (2H, app-dd, partially obscured, *J* 10.7, 5.4 Hz, H-5 and H-3), 4.45 (1H, partially obscured, $J_{Ha,Hb}$ 15.2 Hz, ArC*H*^{a}H^{b}), 4.29 (1H, d, $J_{Hb,Ha}$ 15.2 Hz, ArCH^{a}*H*^{b}), 3.55-3.45 (2H, partially obscured dd and m, $J_{H\text{-}1',H\text{-}1}$ 12.3 Hz, $J_{H\text{-}1',H\text{-}2}$ 6.5 Hz, H-1′ and H-4), 3.25 (2H, app-d, $J_{H\text{-}6,H\text{-}6'=H\text{-}6',H\text{-}6}$ 5.8 Hz, H-6 and H-6′), 3.20 (1H, dd, $J_{H\text{-}1',H\text{-}1}$ 12.4 Hz, $J_{H\text{-}1',H\text{-}2}$ 5.8 Hz, H-1′); **δ_C**: 140.6 (ArC$_{quat}$), 129.4 (ArC^{B}), 128.2 (ArC^{C}), 127.6 (ArC^{D}), 122.7 (ArC^{E}), 72.9 (C-3), 72.7 (C-4), 71.6 (C-2), 70.4 (C-5), 67.3 (ArCH$_2$), 63.8 (C-6), 57.4 (C-1); ArC-B not discernible.
Boronate ammonium species (tetrahedral). **δ_H**: 7.88 (1H, app-dt, *J* 13.0, 8.4, 8.4 Hz, ArH), 7.52 (1H, app-d, partially obscured, *J* 8.3 Hz, ArH), 7.40 (1H, dd, J 7.2, 1.8 Hz, ArH), 7.05-6.95 (1H, m, ArH), 4.50-4.39 (1H, obscured), 4.20-4.10 (1H, m), 3.85-3.75 (1H, m, H-6), 3.72-3.62 (2H, m, H and H-6′), 3.40-3.30 (1H, m), 1.45-1.28 (2H, m, H-1 and H-1′). **δ_C**: 132.2, 130.3, 127.9, 115.9, 72.3 (ArCH$_2$, width 52 Hz), 68.1, 62.9 (C-6), 55.2, 9.9 (C-1).
δ_B: 28.3 (broad, integration: 1.1), 19.4 (sharp, integration: 6.1), 12.3 (sharp and merging with 11.0 ppm signal, integration: 1.2), 11.0 (sharp and merging with 12.3 ppm signal, integration: 1.0).

^{1}H-NMR

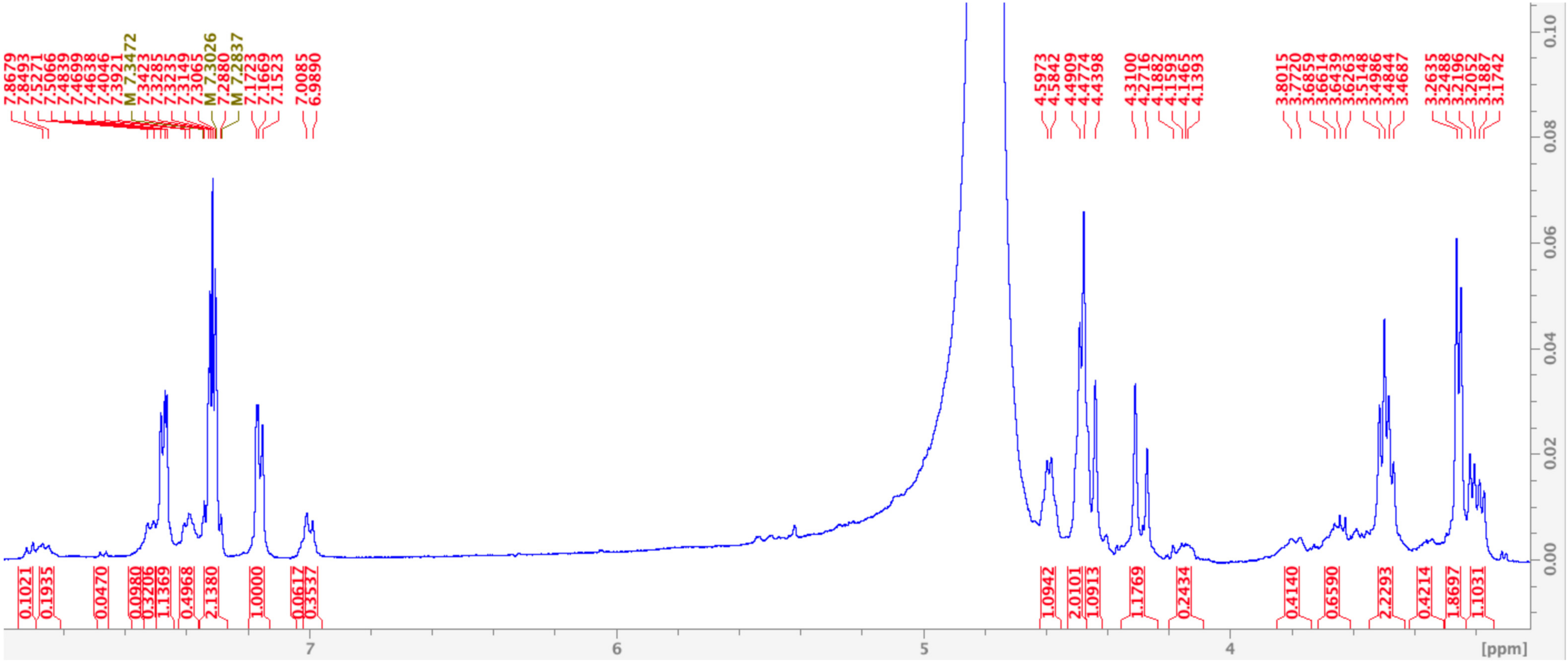

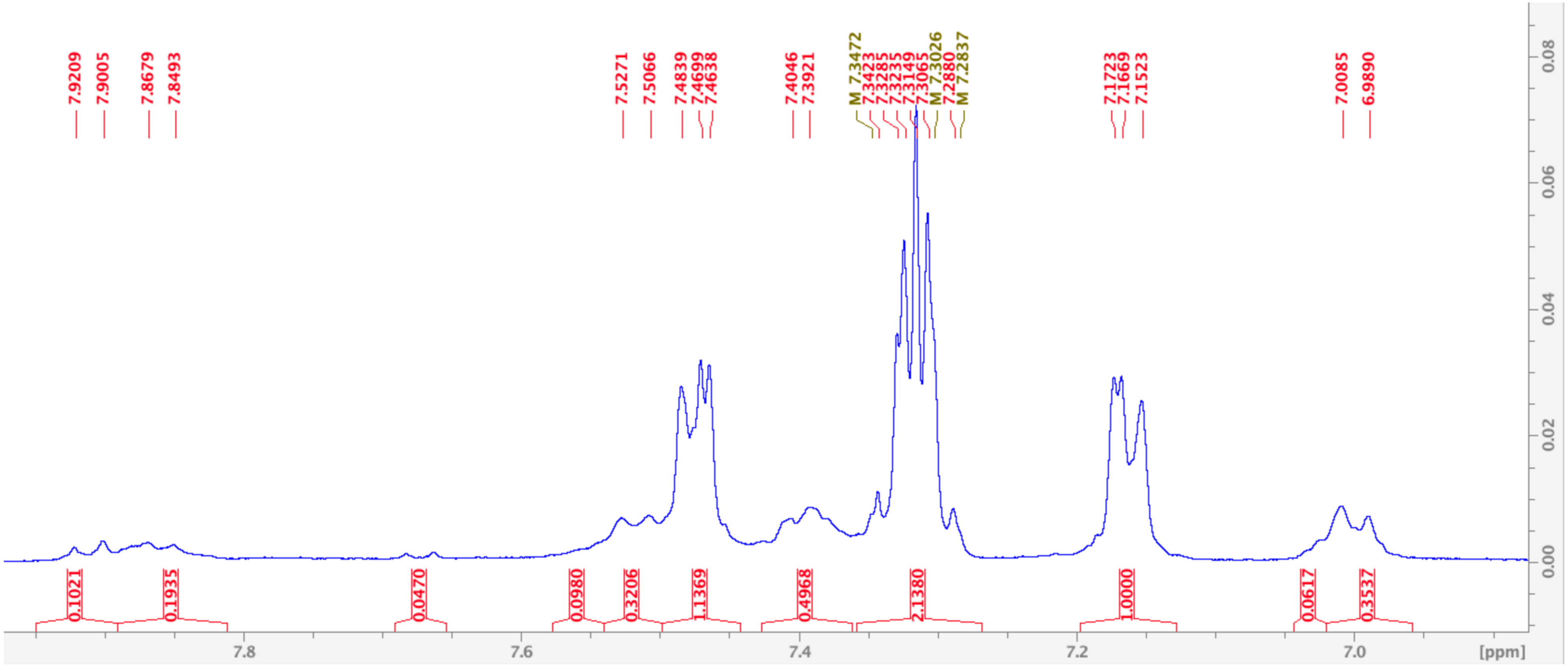

7.9209
7.9005
7.8679
7.8493
7.5271
7.5066
7.4839
7.4699
7.4638
7.4046
7.3921
M 7.3472
7.3423
7.3285
7.3235
7.3149
7.3065
M 7.3026
7.2880
M 7.2837
7.1723
7.1669
7.1523
7.0085
6.9890
0.1021
0.1935
0.0470
0.0980
0.3206
1.1369
0.4968
2.1380
1.0000
0.0617
0.3537
7.8
7.6
7.4
7.2
7.0
[ppm]
0.08
0.06
0.04
0.02
0.00

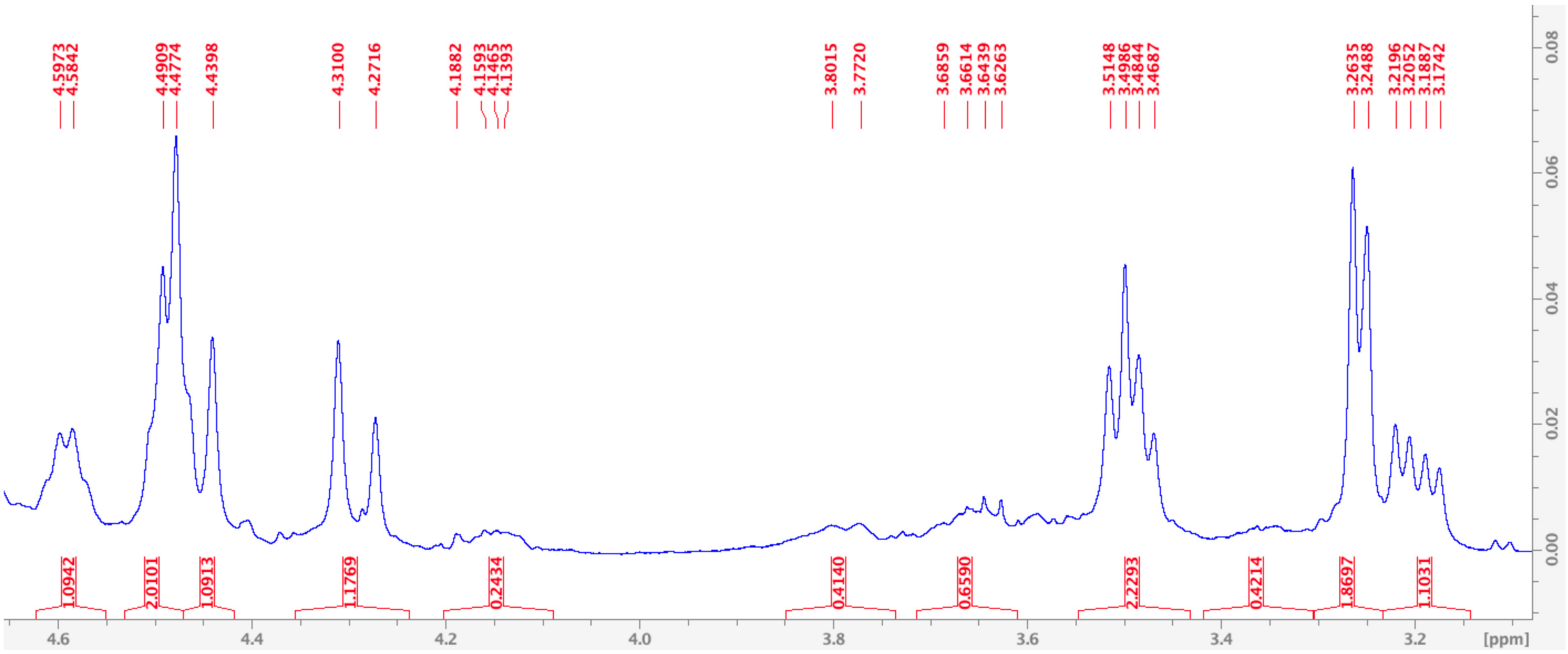
4.5973
4.5842
4.4909
4.4774
4.4398
4.3100
4.2716
4.1882
4.1593
4.1465
4.1393
3.8015
3.7720
3.6859
3.6614
3.6439
3.6263
3.5148
3.4986
3.4844
3.4687
3.2635
3.2488
3.2196
3.2052
3.1887
3.1742
1.0942
2.0101
1.0913
1.1769
0.2434
0.4140
0.6590
2.2293
0.4214
1.8697
1.1031
0.08
0.06
0.04
0.02
0.00
4.6
4.4
4.2
4.0
3.8
3.6
3.4
3.2
[ppm]

^{1}H-NMR (minor species)

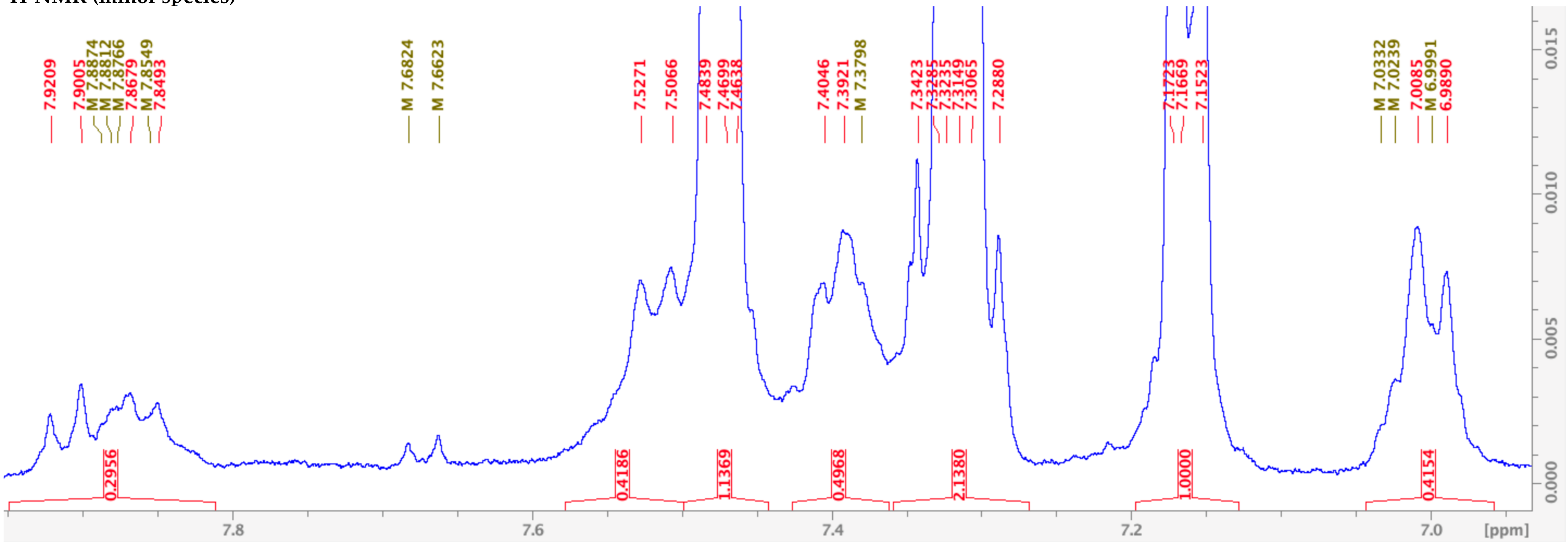

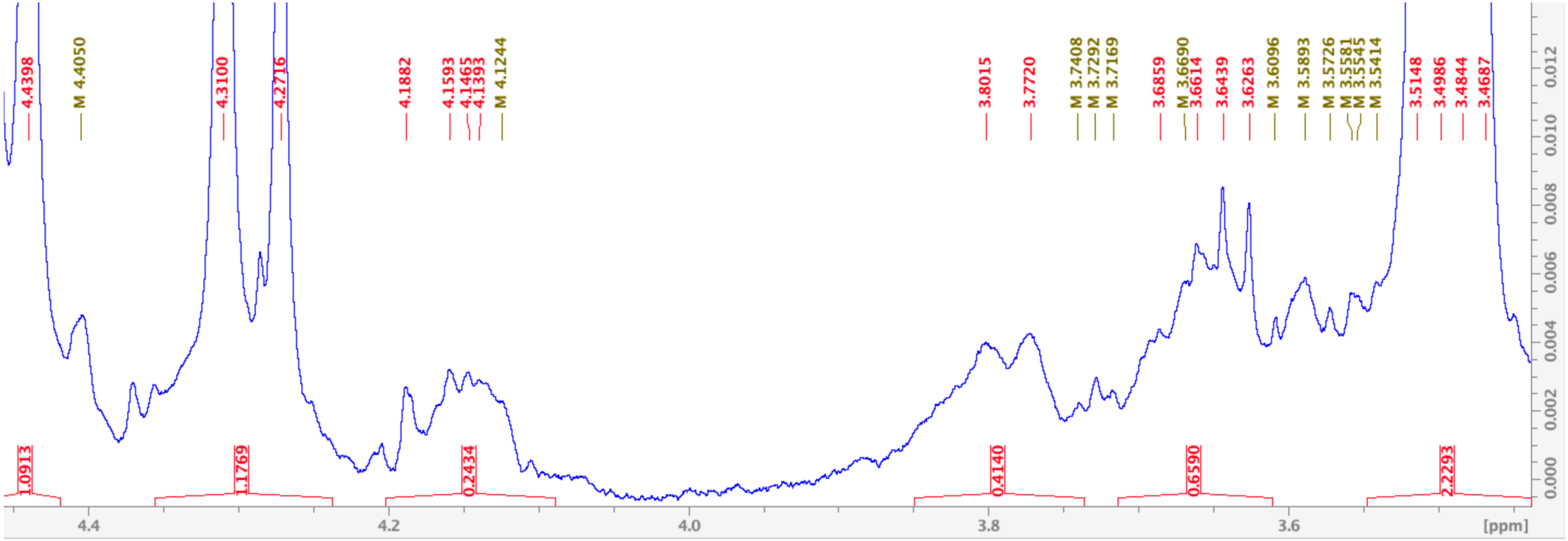
4.4398
M 4.4050
4.3100
4.2716
4.1882
4.1593
4.1465
4.1393
M 4.1244
3.8015
3.7720
M 3.7408
M 3.7292
M 3.7169
3.6859
M 3.6690
3.6614
3.6439
3.6263
M 3.6096
M 3.5893
M 3.5726
M 3.5581
M 3.5545
M 3.5414
3.5148
3.4986
3.4844
3.4687
1.0913
1.1769
0.2434
0.4140
0.6590
2.2293
4.4
4.2
4.0
3.8
3.6
[ppm]
0.012
0.010
0.008
0.006
0.004
0.002
0.000

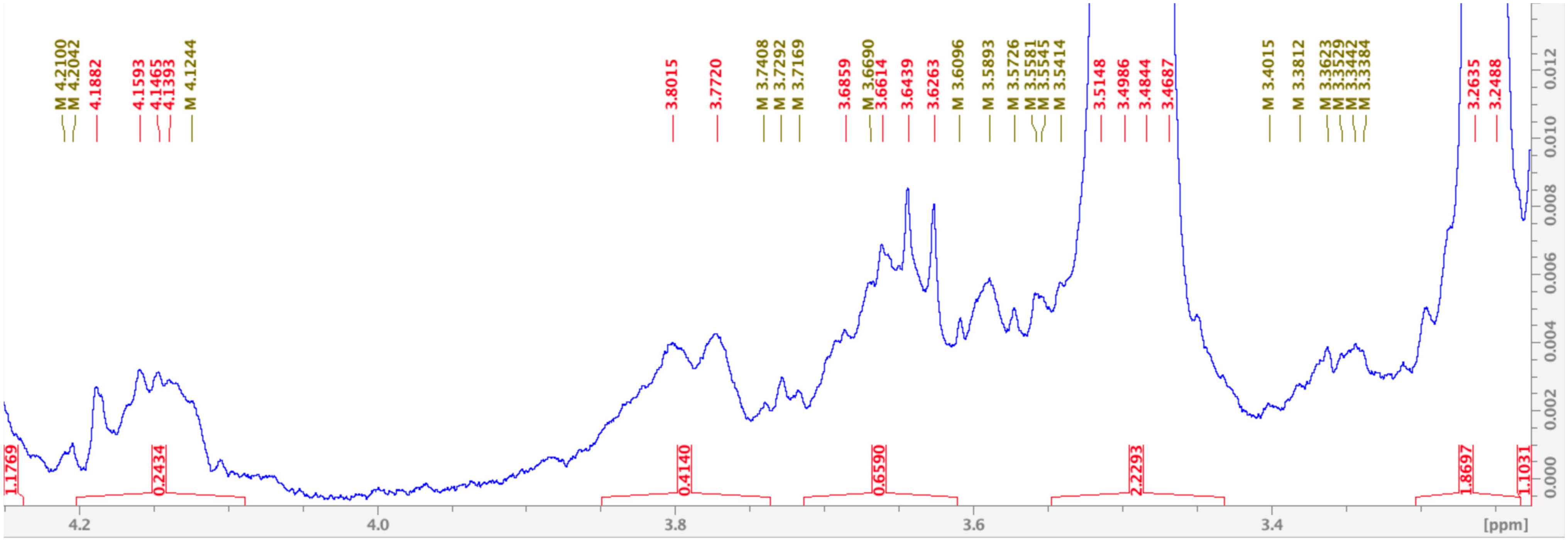
M 4.2100
M 4.2042
4.1882
4.1593
4.1465
4.1393
M 4.1244
3.8015
3.7720
M 3.7408
M 3.7292
M 3.7169
3.6859
M 3.6690
3.6614
3.6439
3.6263
M 3.6096
M 3.5893
M 3.5726
M 3.5581
M 3.5545
M 3.5414
3.5148
3.4986
3.4844
3.4687
M 3.4015
M 3.3812
M 3.3623
M 3.3529
M 3.3442
M 3.3384
3.2635
3.2488
1.1769
0.2434
0.4140
0.6590
2.2293
1.8697
1.1031
4.2
4.0
3.8
3.6
3.4
[ppm]
0.012
0.010
0.008
0.006
0.004
0.002
0.000

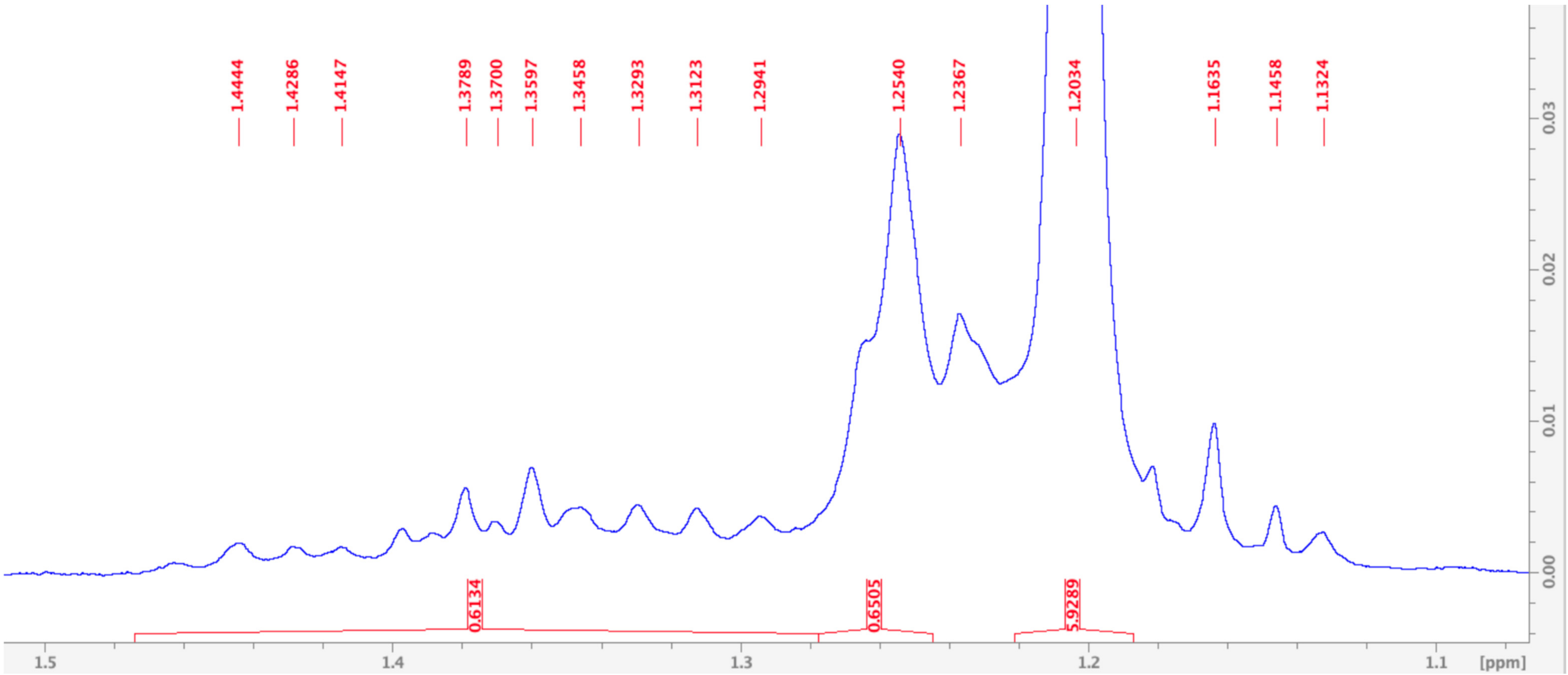
1.4444
1.4286
1.4147
1.3789
1.3700
1.3597
1.3458
1.3293
1.3123
1.2941
1.2540
1.2367
1.2034
1.1635
1.1458
1.1324
0.6134
0.6505
5.9289
1.5
1.4
1.3
1.2
1.1
[ppm]
0.03
0.02
0.01
0.00

^{13}C-NMR

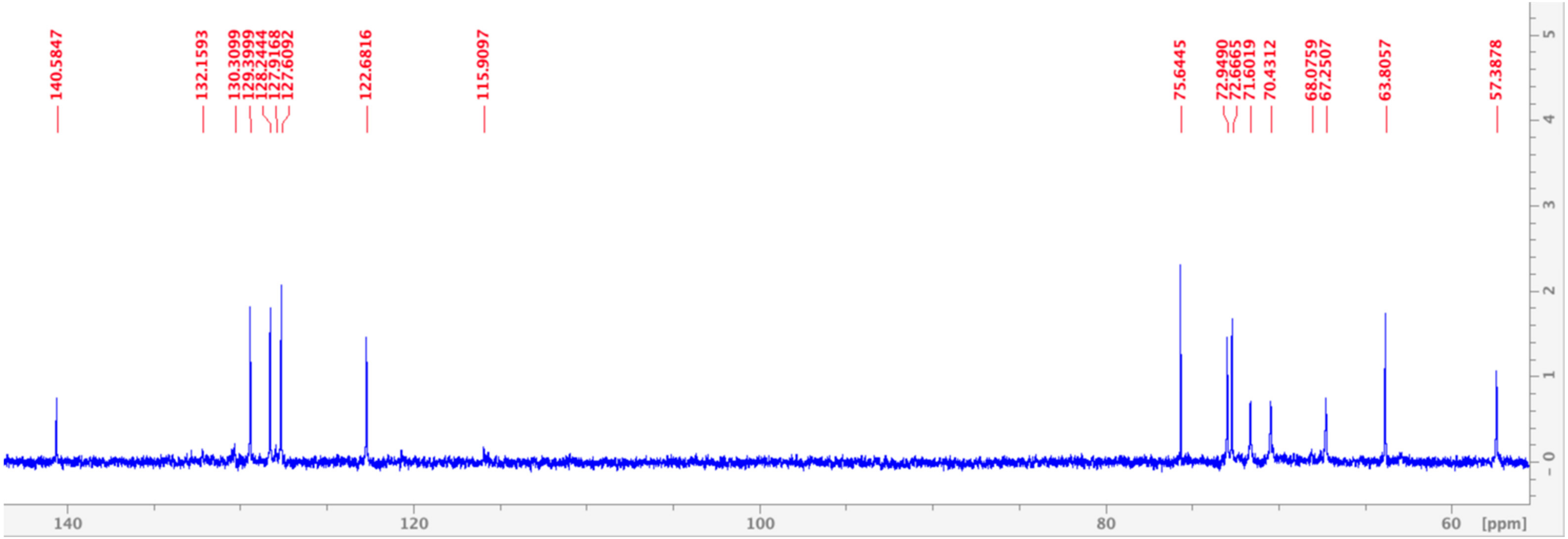

140.5847
132.1593
130.3099
129.3999
128.2444
127.9168
127.6092
122.6816
115.9097
75.6445
72.9490
72.6665
71.6019
70.4312
68.0759
67.2507
63.8057
57.3878
5
4
3
2
1
0
140
120
100
80
60
[ppm]

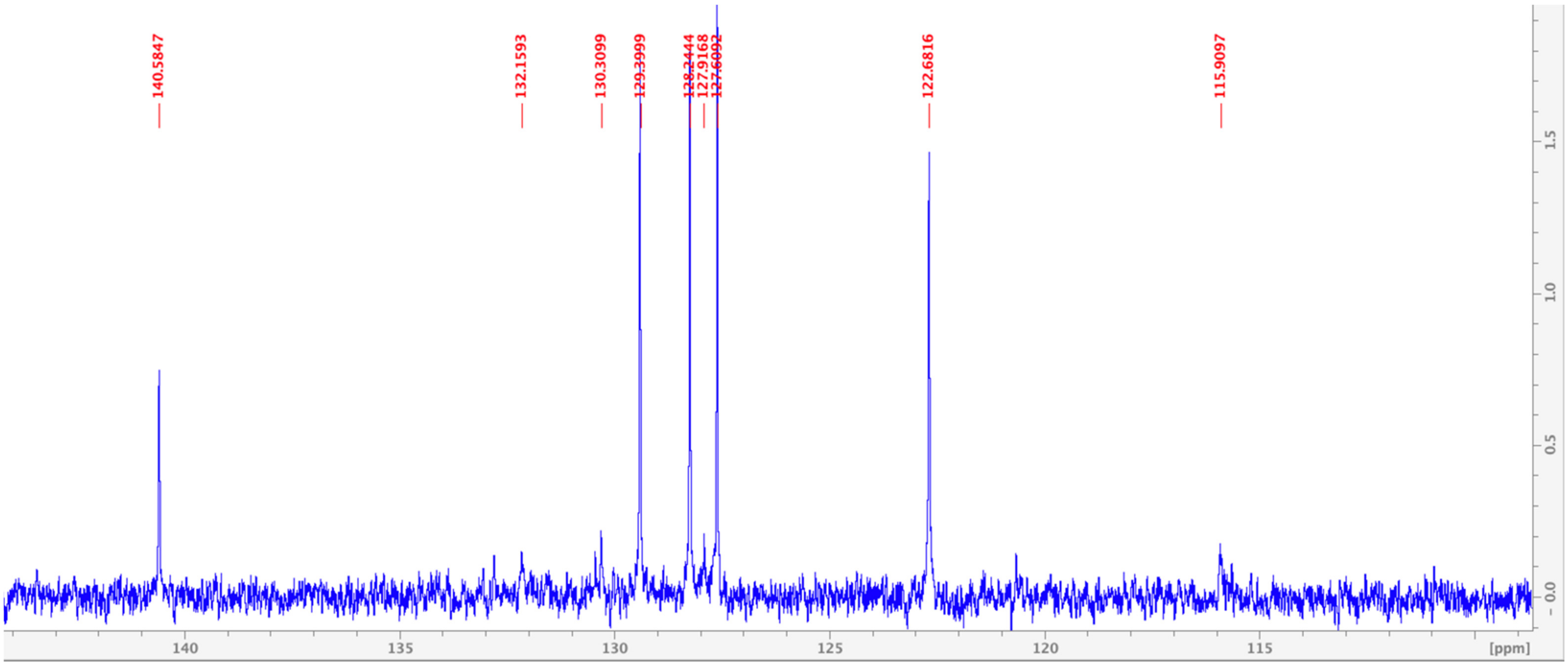
140.5847
132.1593
130.3099
129.3999
128.2444
127.9168
127.6092
122.6816
115.9097
1.5
1.0
0.5
0.0
140
135
130
125
120
115
[ppm]

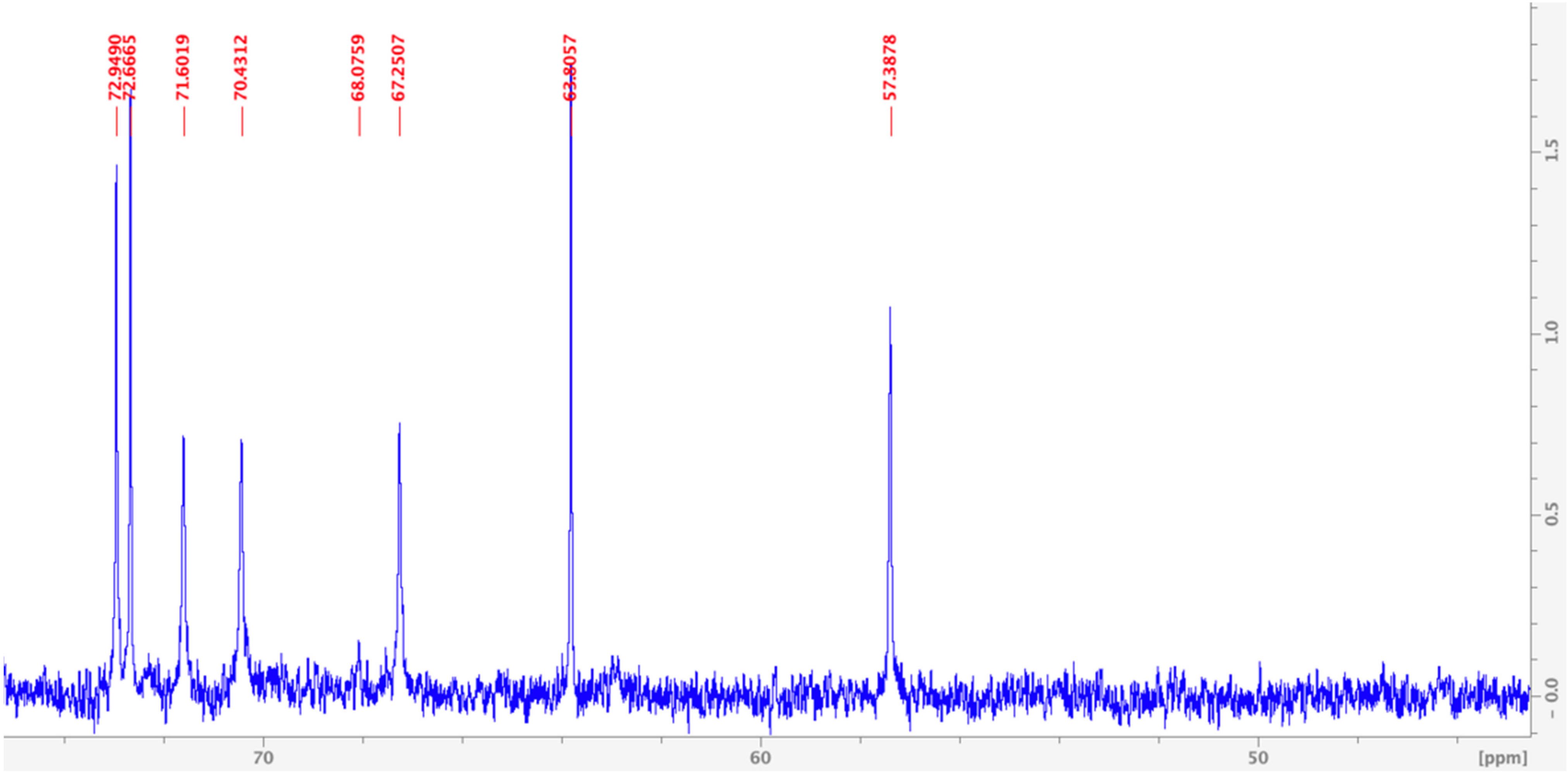
72.9490
72.6665
71.6019
70.4312
68.0759
67.2507
63.8057
57.3878
1.5
1.0
0.5
0.0
70
60
50
[ppm]

DEPT

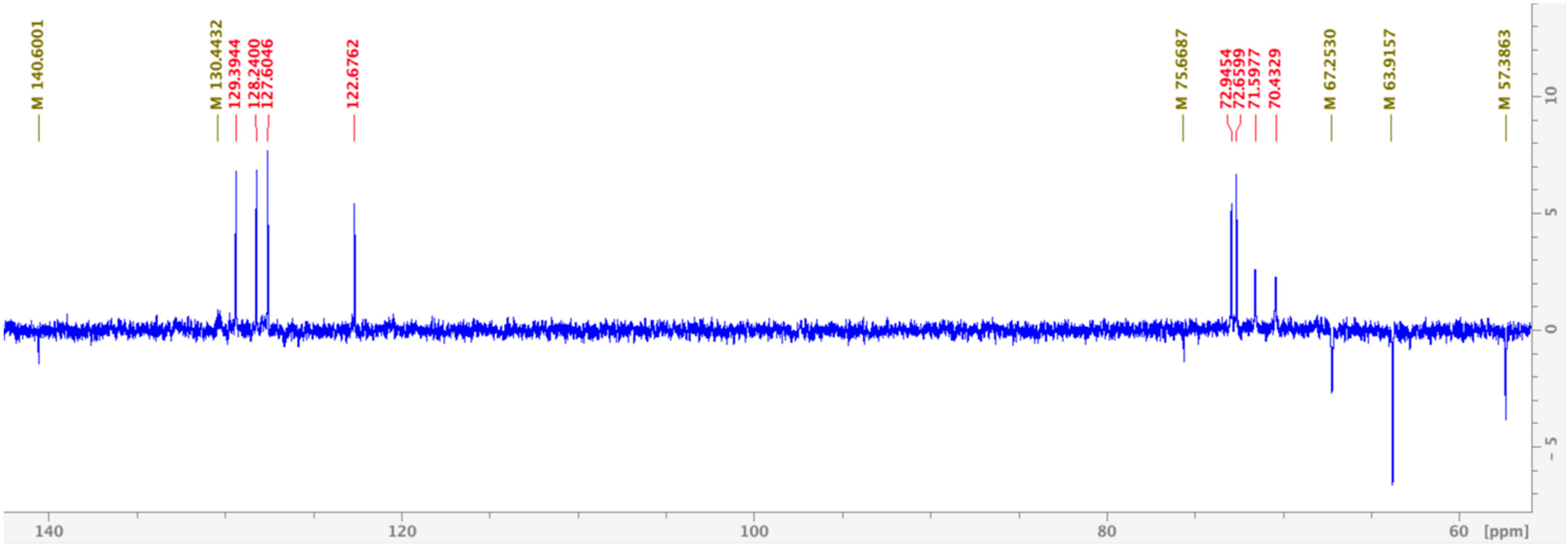

M 140.6001
M 130.4432
129.3944
128.2400
127.6046
122.6762
M 75.6687
72.9454
72.6599
71.5977
70.4329
M 67.2530
M 63.9157
M 57.3863
10
5
0
- 5
140
120
100
80
60
[ppm]

^{11}B-NMR

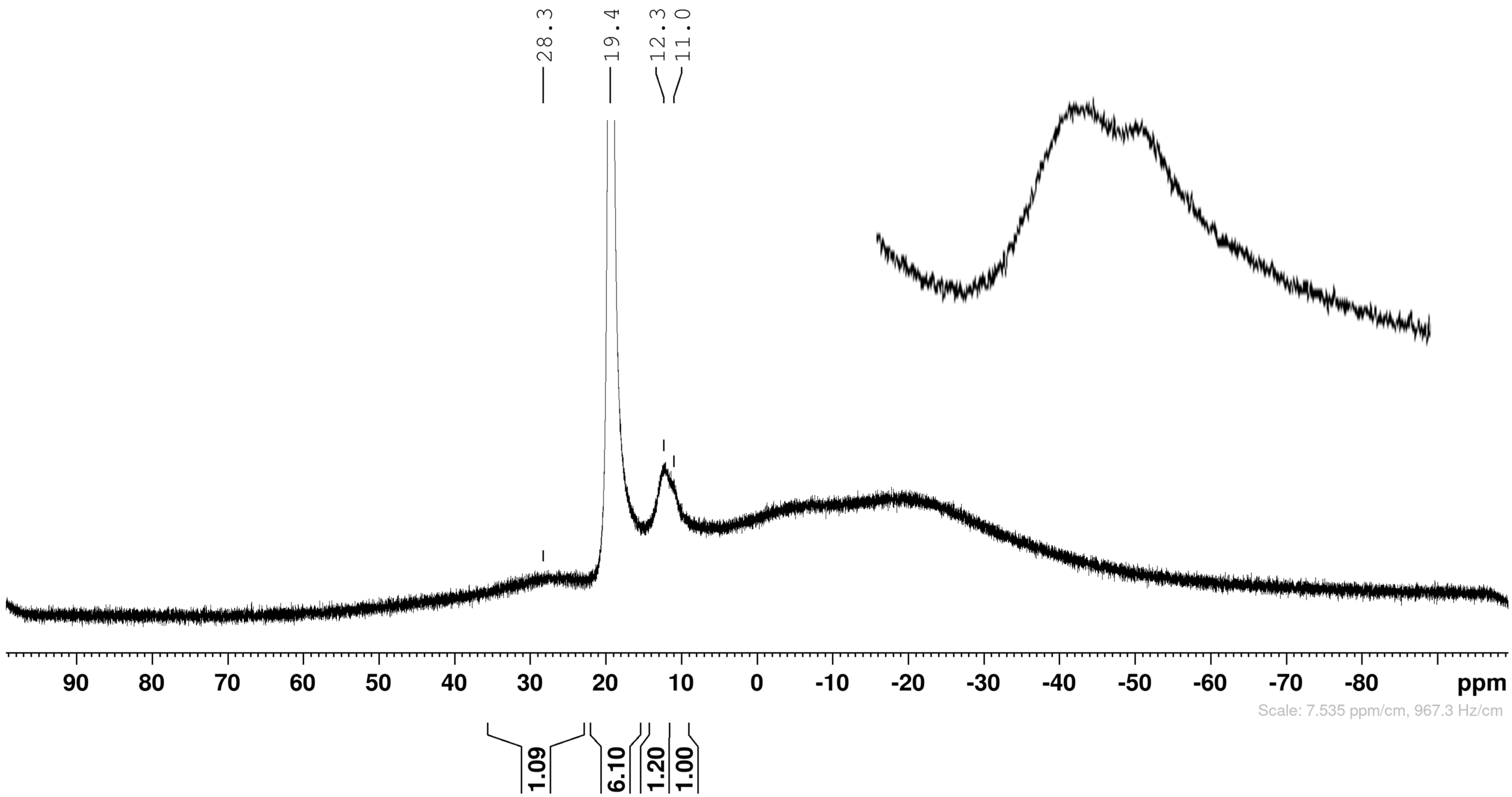

28.3
19.4
12.3
11.0
90 80 70 60 50 40 30 20 10 0 -10 -20 -30 -40 -50 -60 -70 -80 ppm
Scale: 7.535 ppm/cm, 967.3 Hz/cm
1.09
6.10
1.20
1.00

COSY

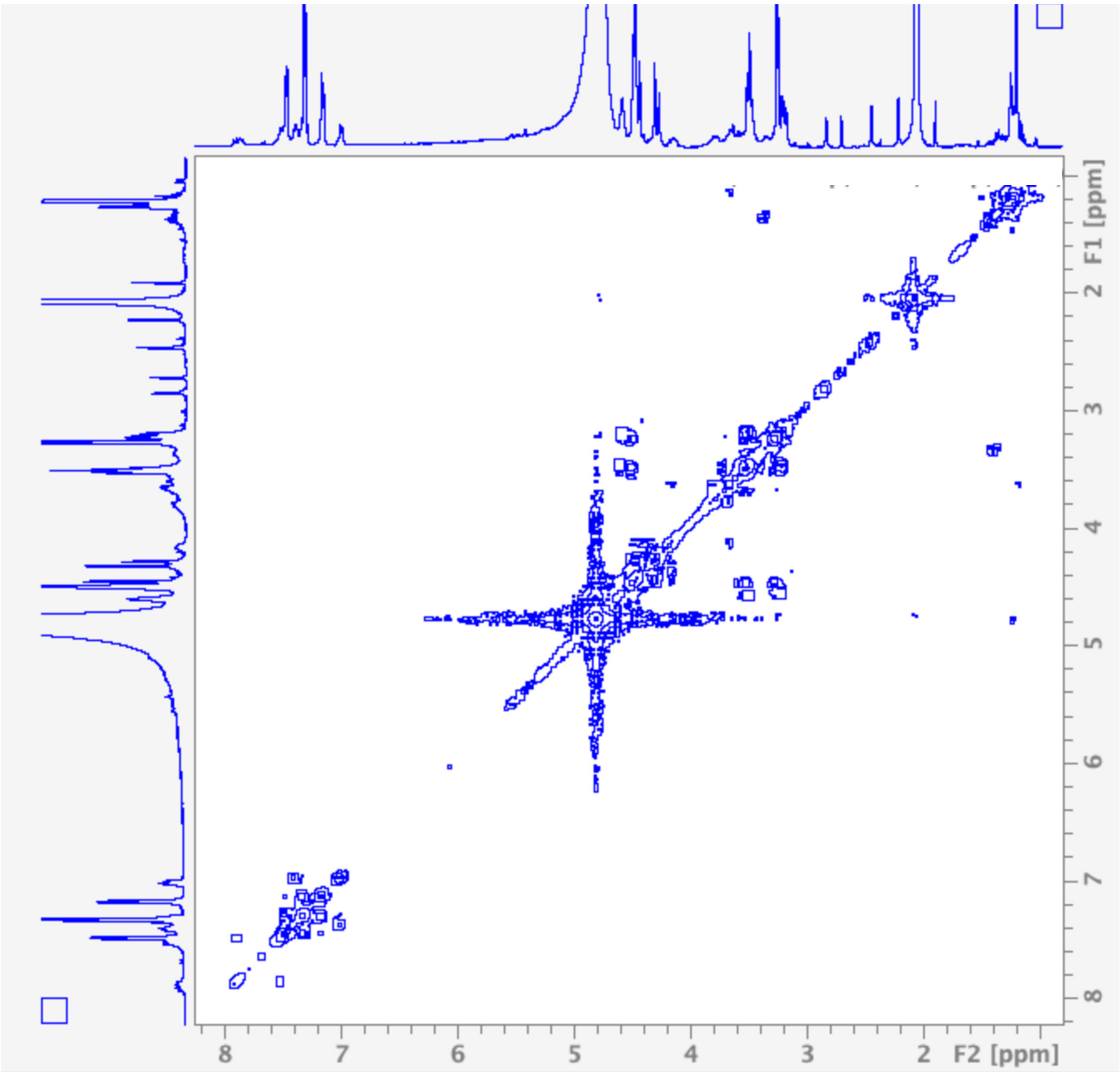

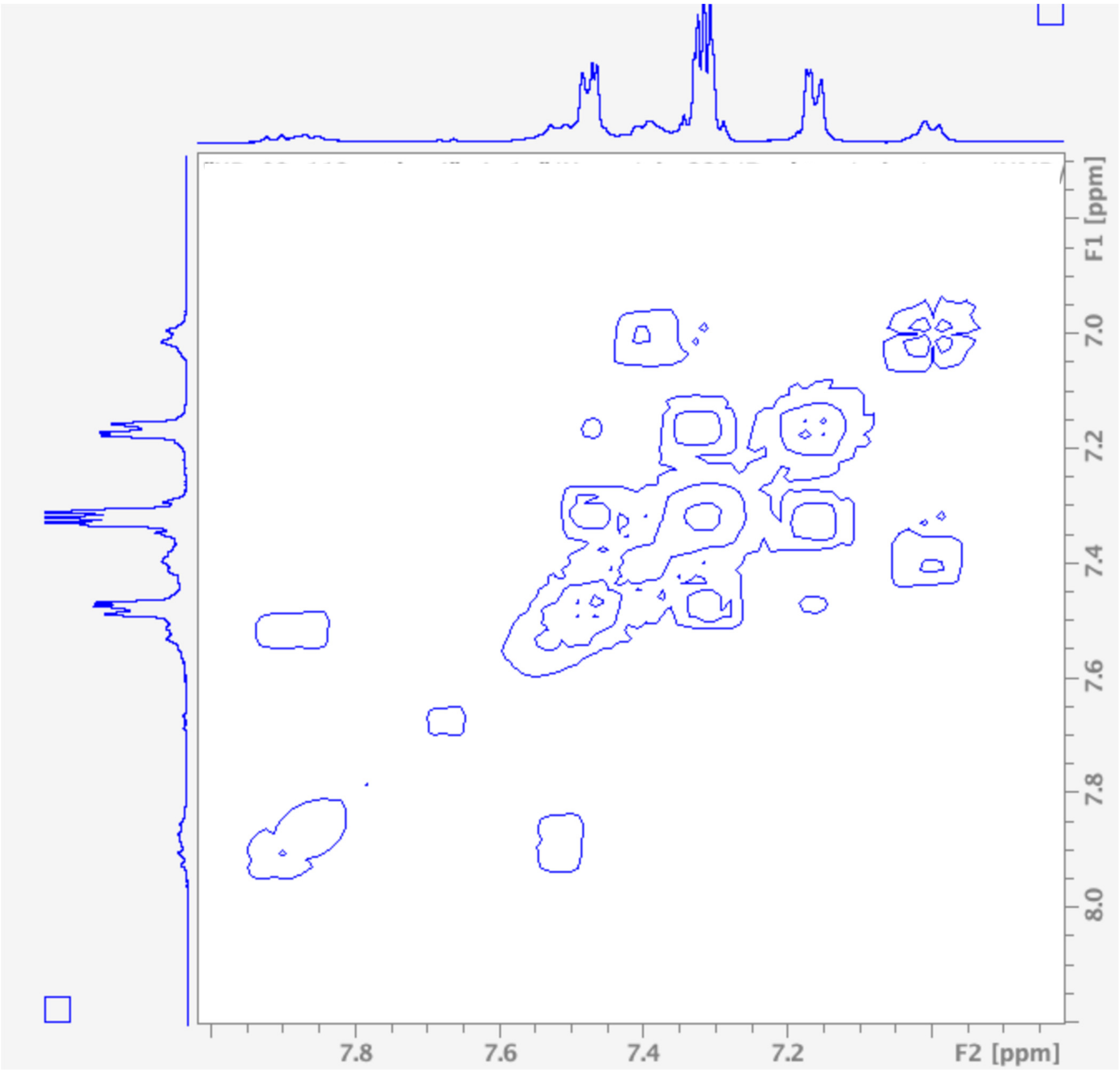
F1 [ppm]
7.0
7.2
7.4
7.6
7.8
8.0
7.8
7.6
7.4
7.2
F2 [ppm]

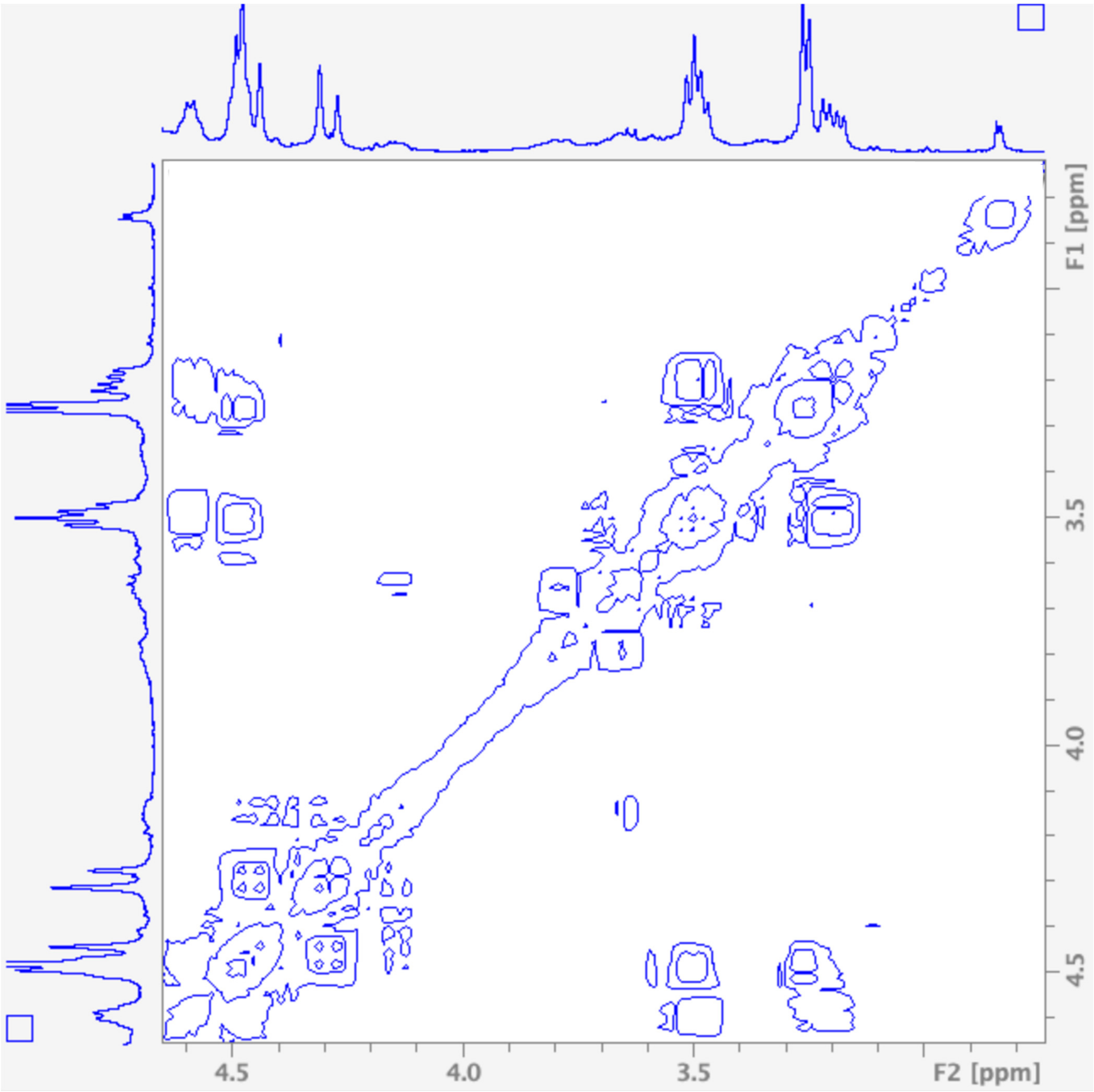
F1 [ppm]
3.5
4.0
4.5
4.5
4.0
3.5
F2 [ppm]

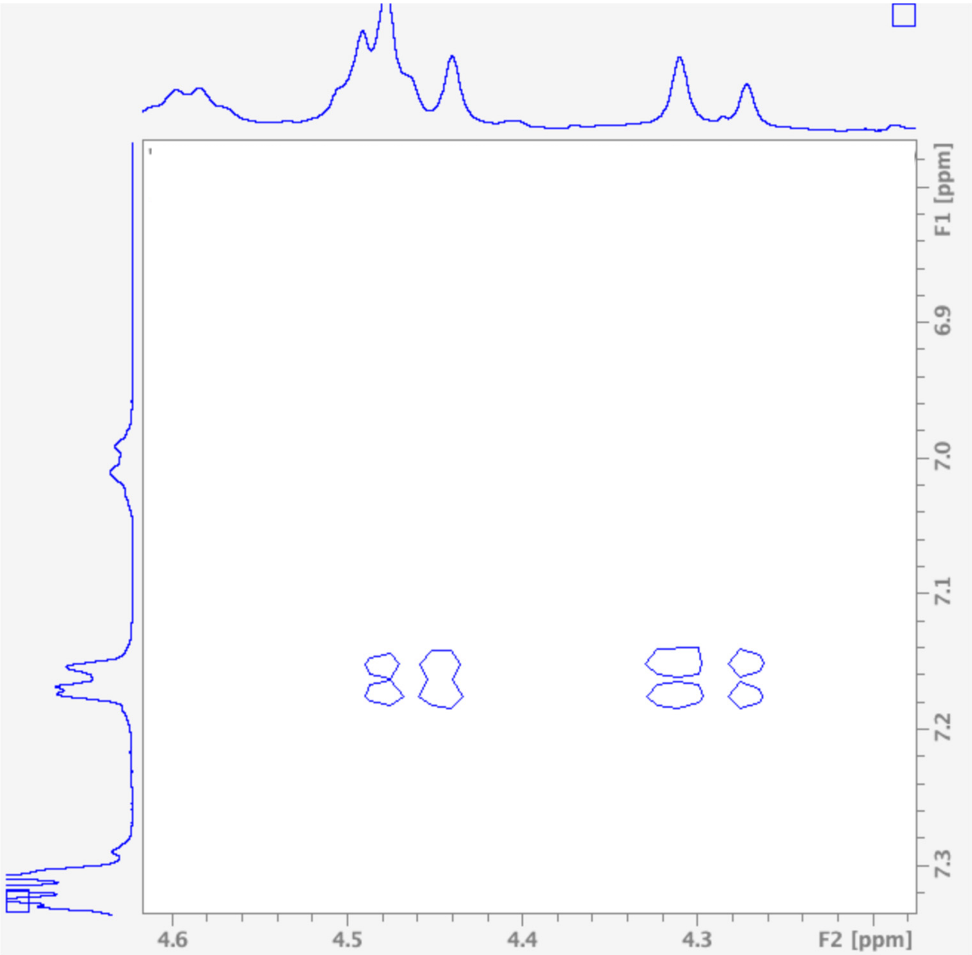
F1 [ppm]
6.9
7.0
7.1
7.2
7.3
4.6
4.5
4.4
4.3
F2 [ppm]

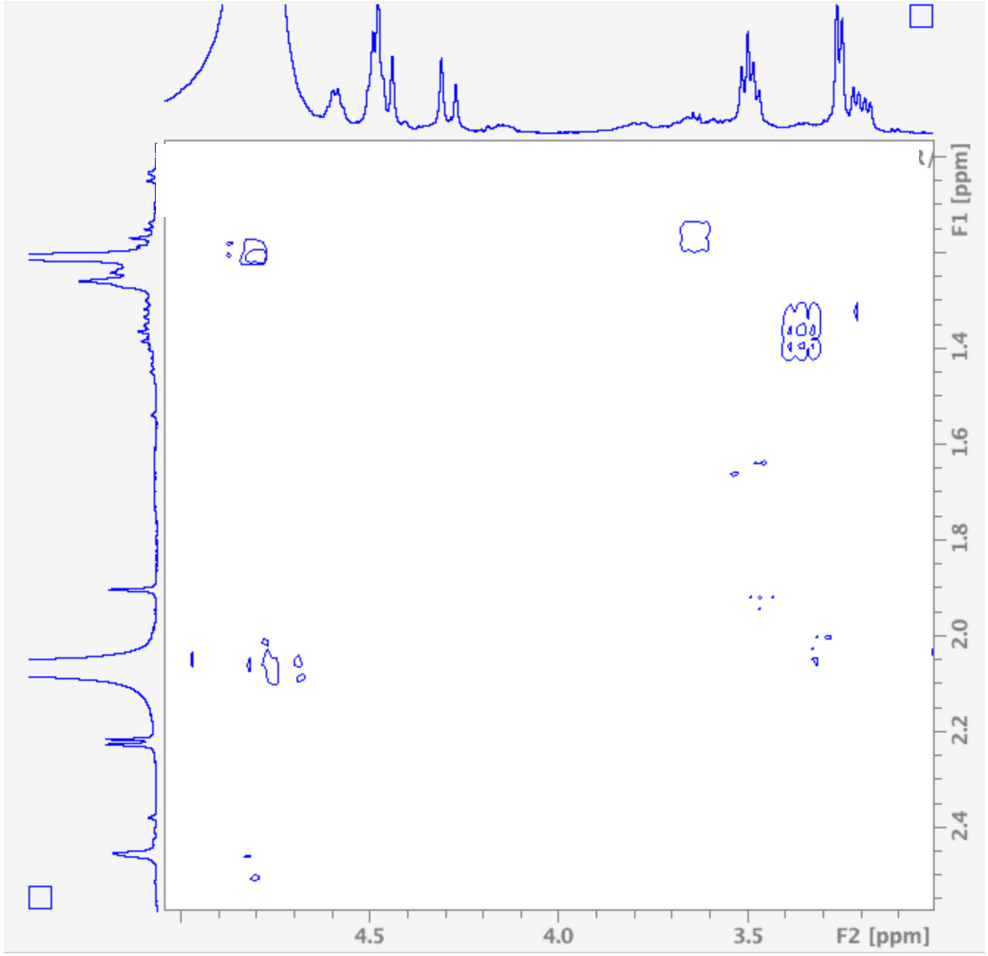
F1 [ppm]
1.4
1.6
1.8
2.0
2.2
2.4
4.5
4.0
3.5
F2 [ppm]

HSQC

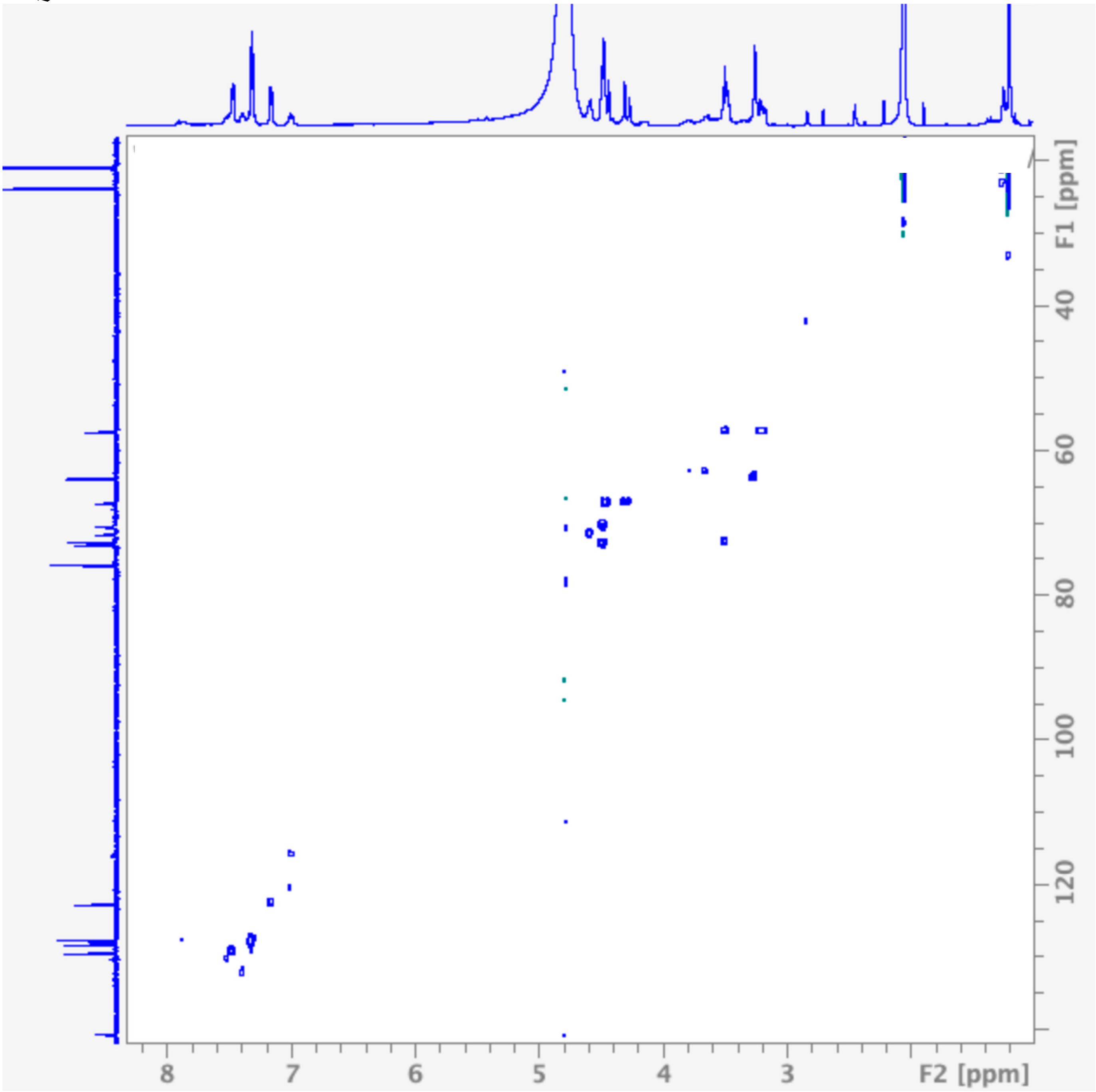

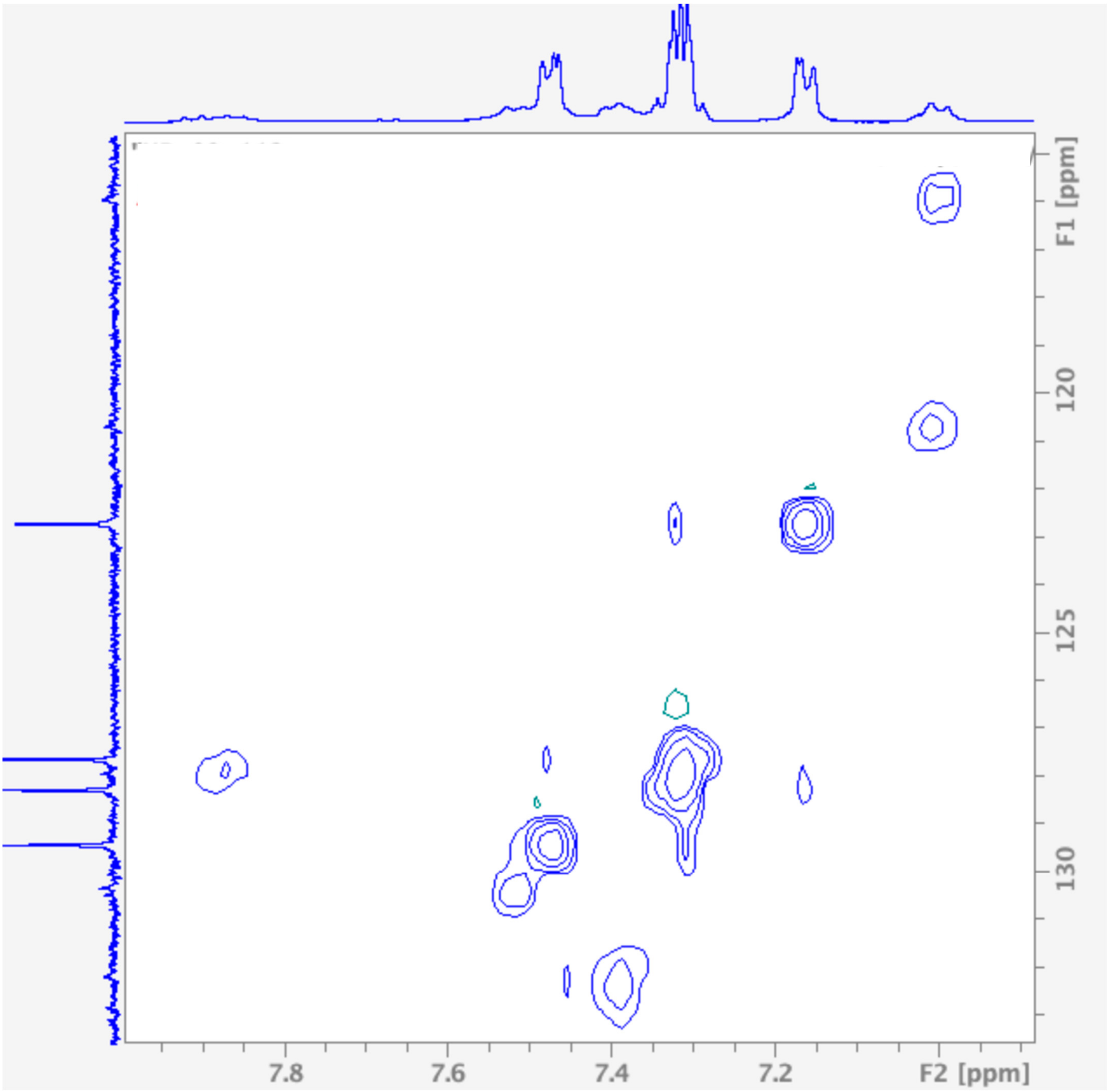
F1 [ppm]
120
125
130
7.8
7.6
7.4
7.2
F2 [ppm]

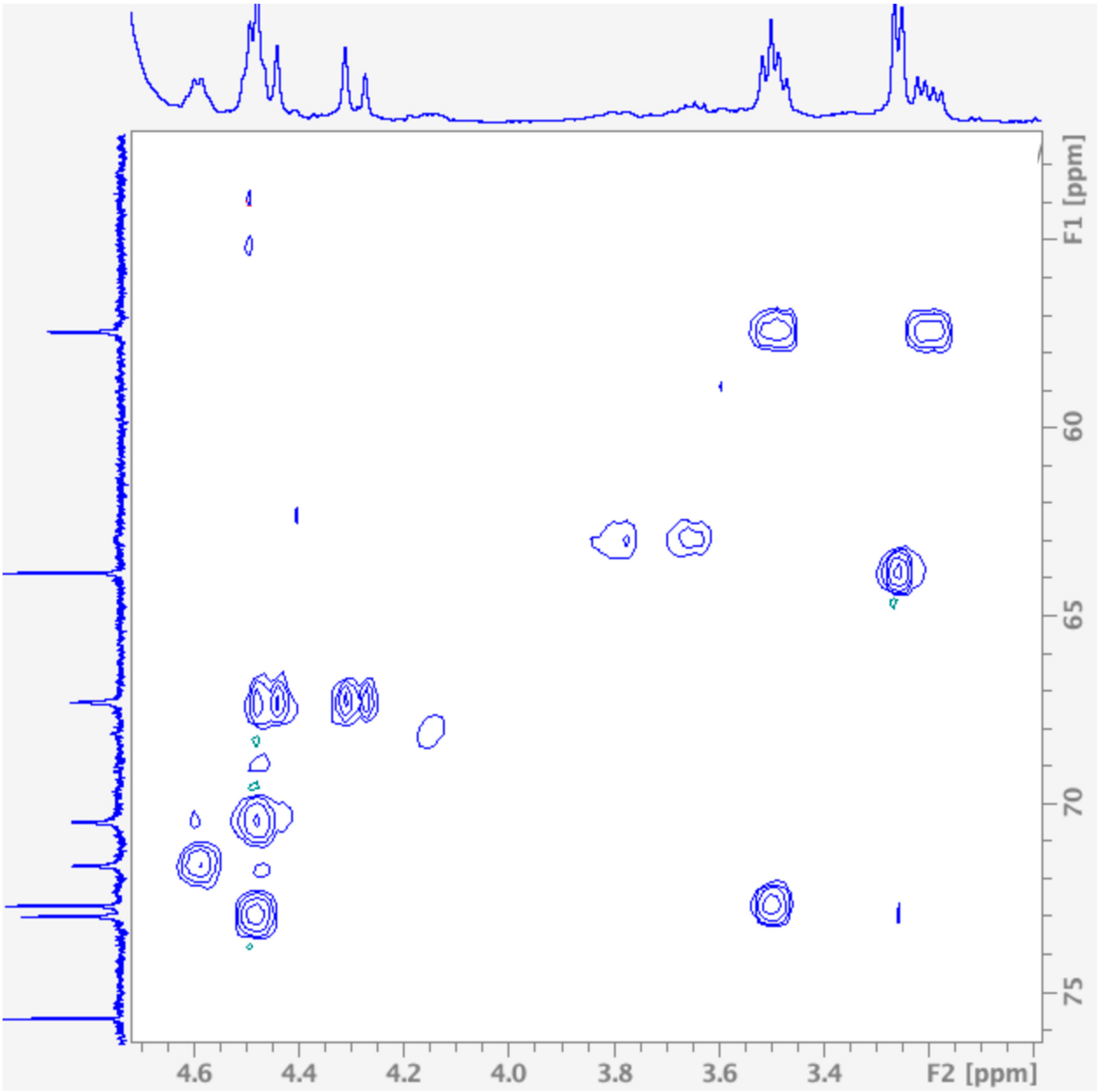

F1 [ppm]
60
65
70
75
4.6
4.4
4.2
4.0
3.8
3.6
3.4
F2 [ppm]

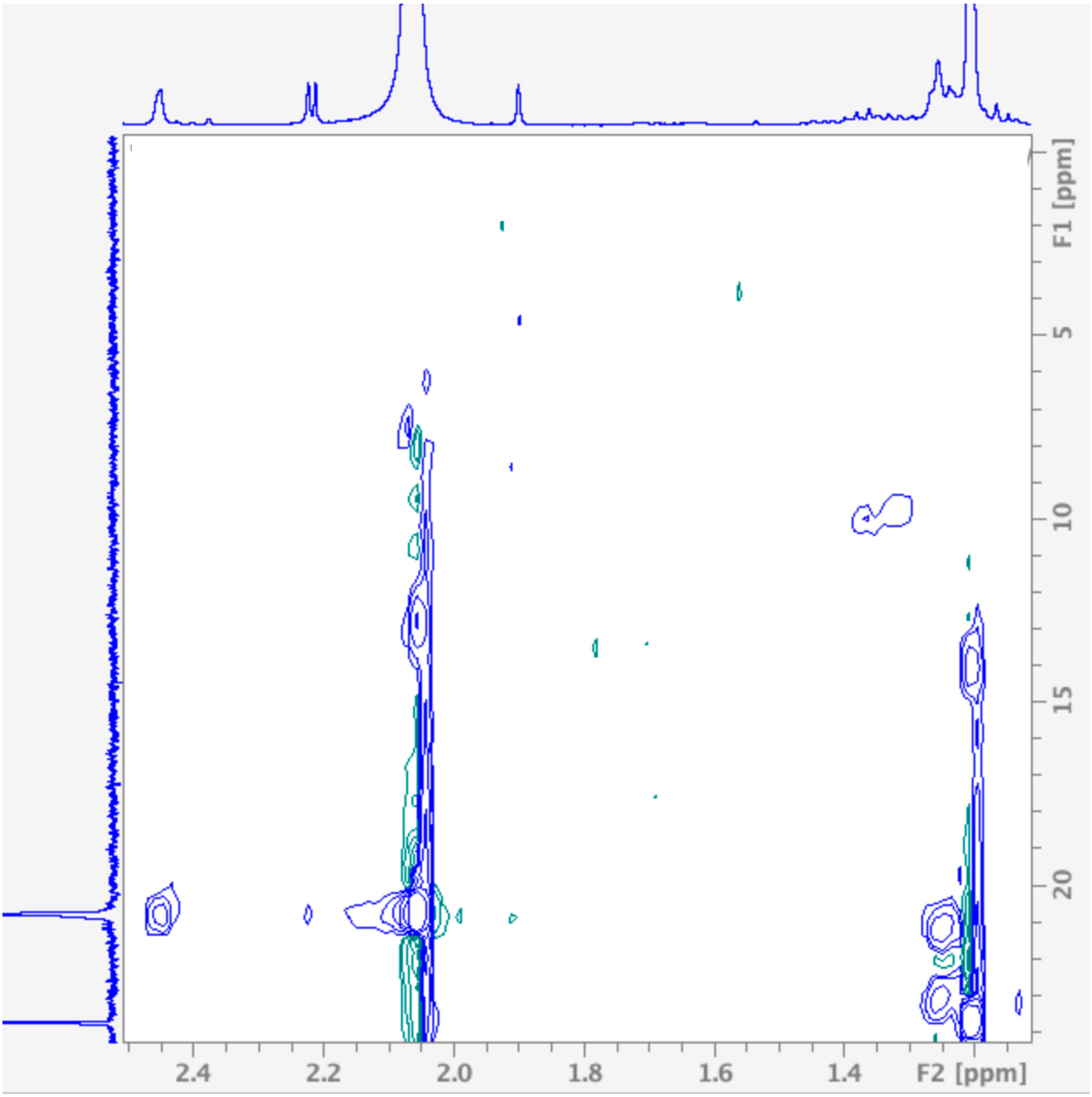

F1 [ppm]
5
10
15
20
2.4
2.2
2.0
1.8
1.6
1.4
F2 [ppm]

HMBC

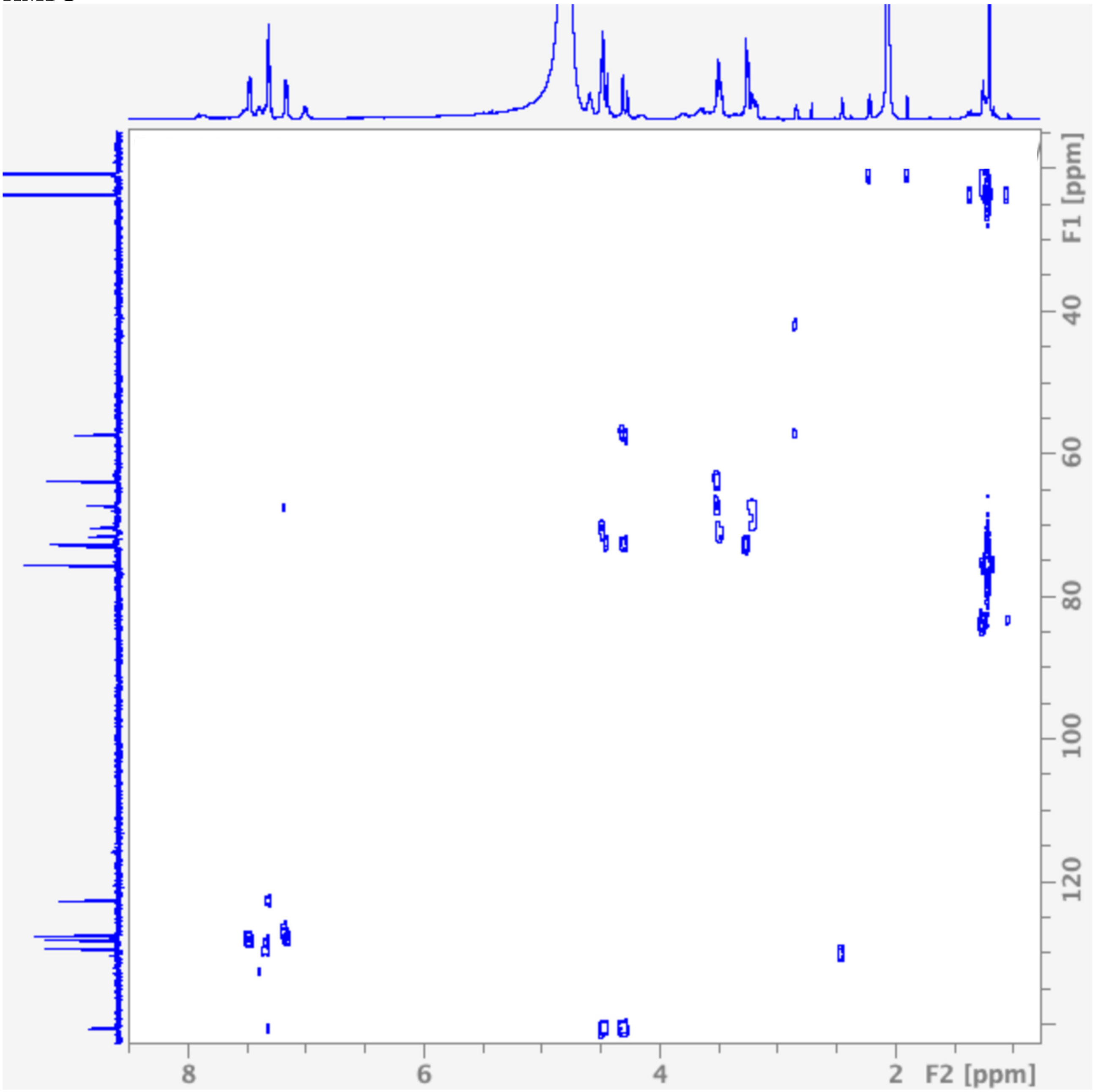

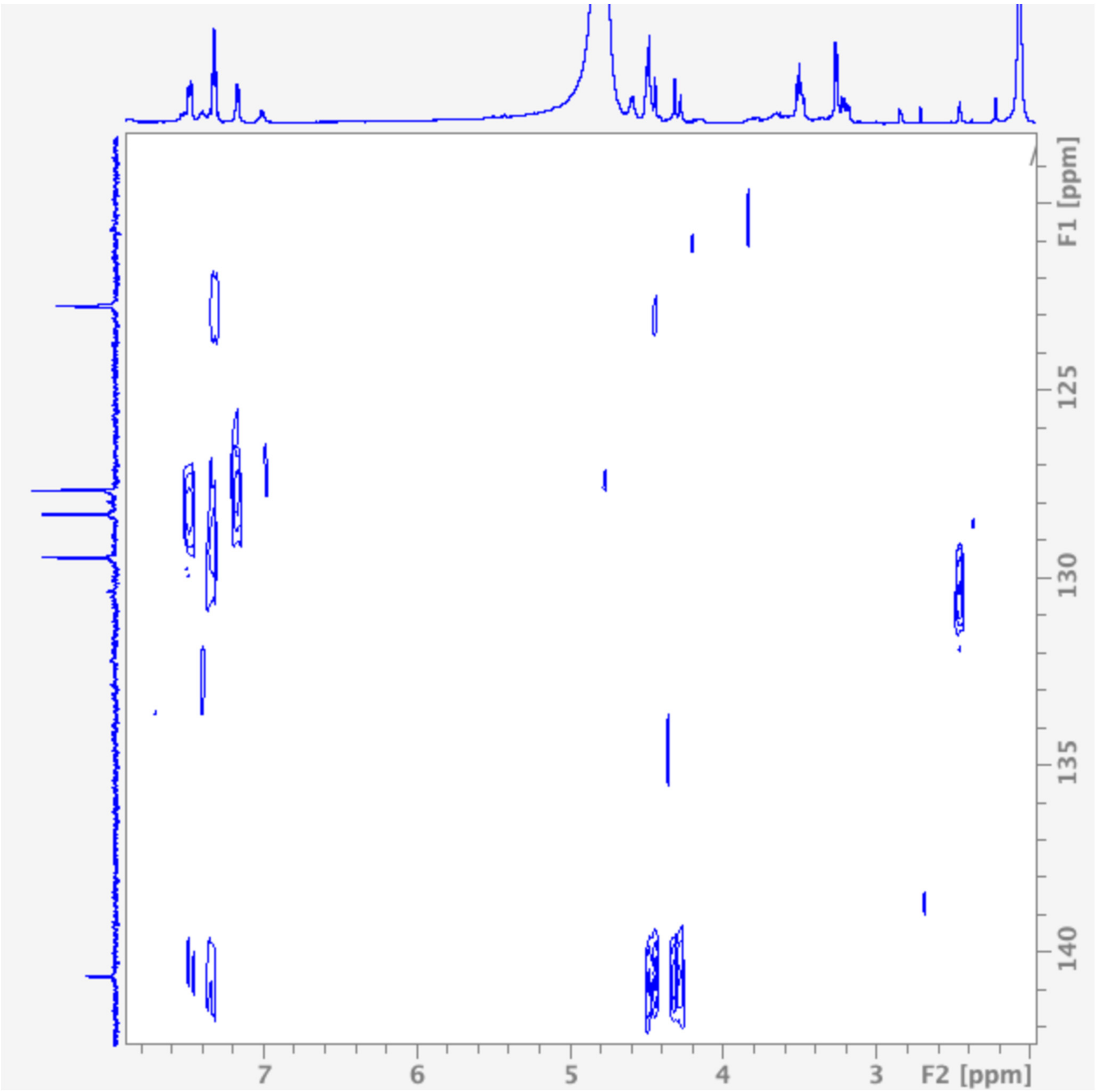

F1 [ppm]
125
130
135
140
7
6
5
4
3
F2 [ppm]

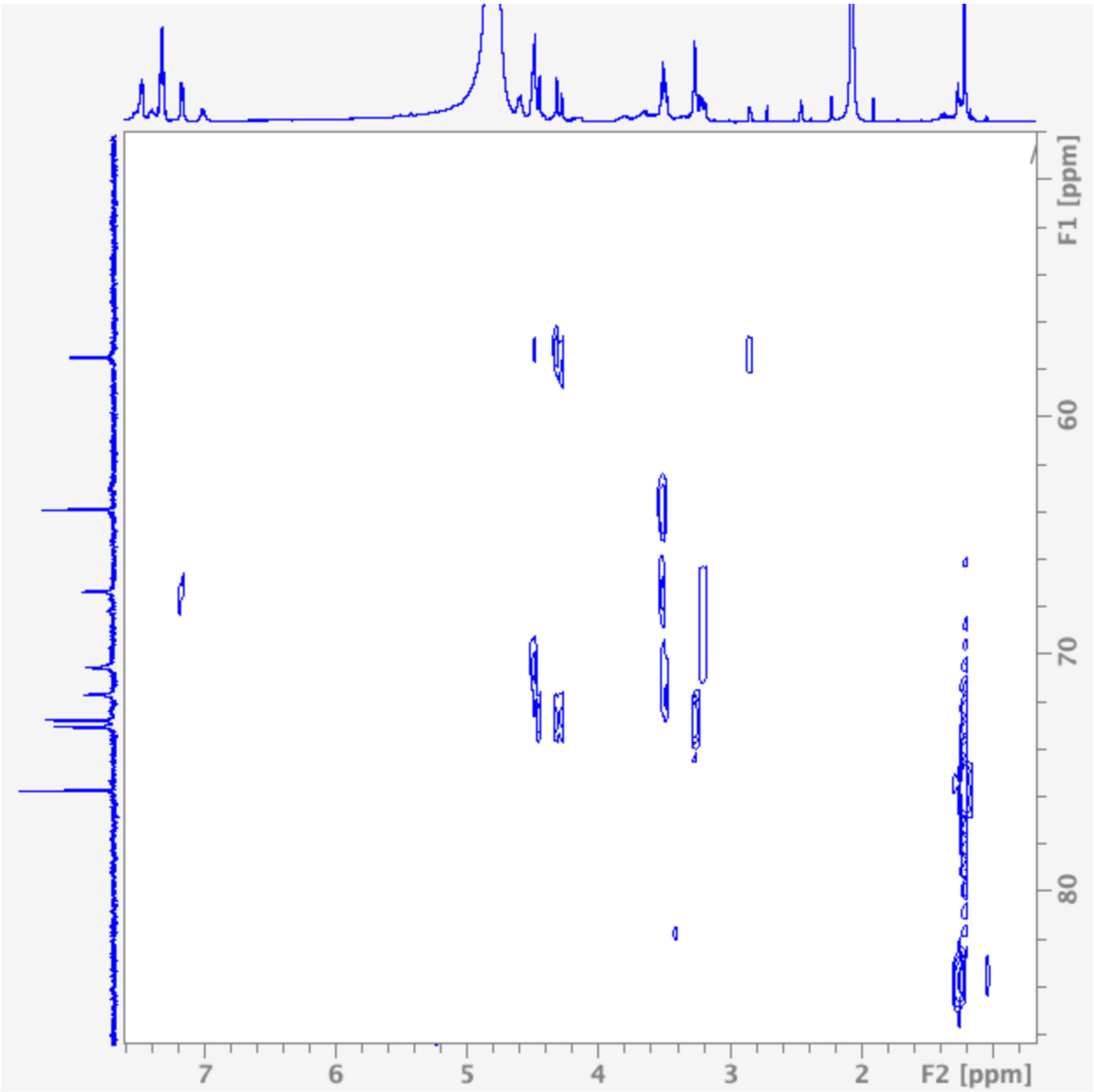
F1 [ppm]
60
70
80
7
6
5
4
3
2
F2 [ppm]

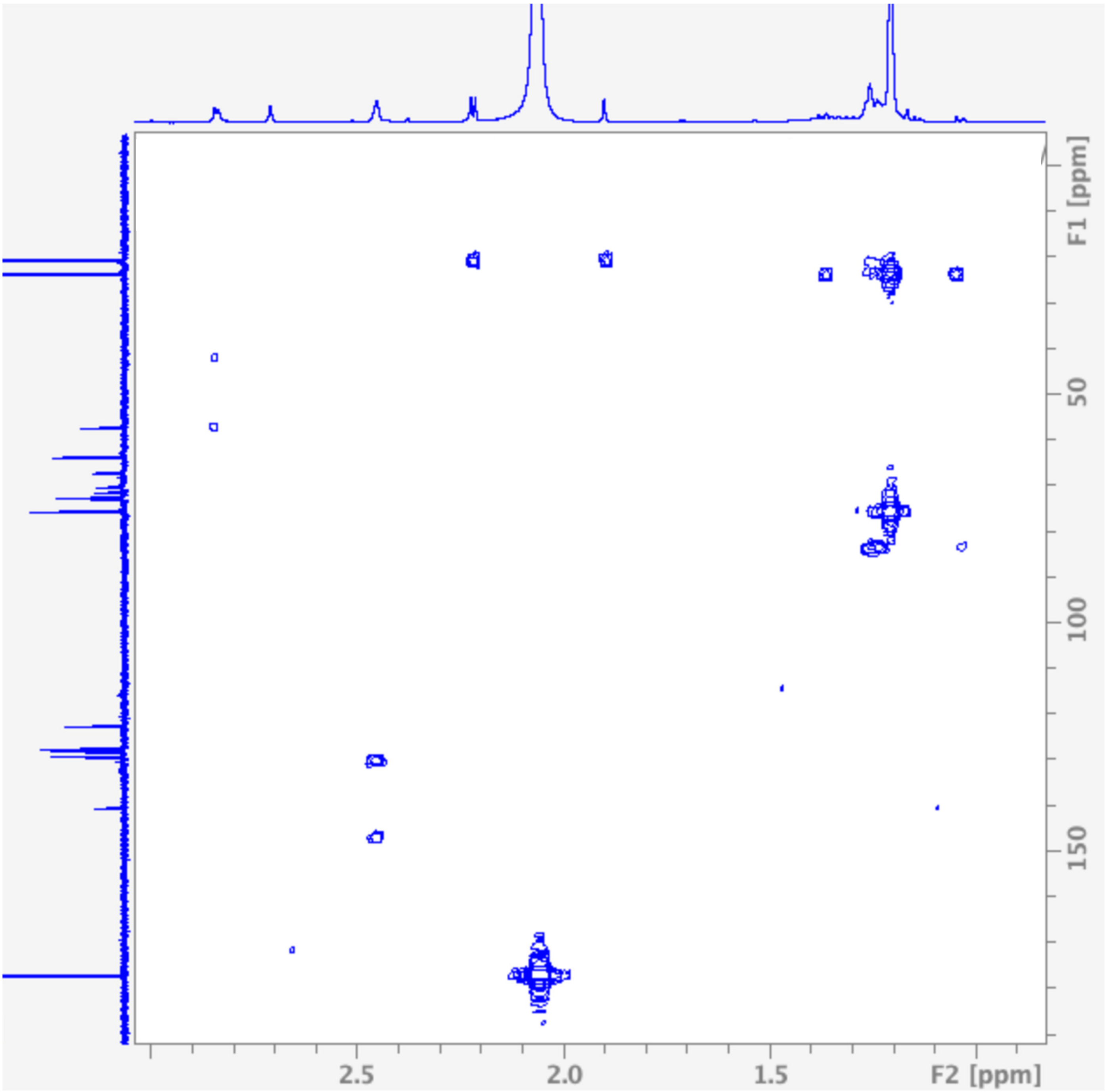

F1 [ppm]
50
100
150
2.5
2.0
1.5
F2 [ppm]

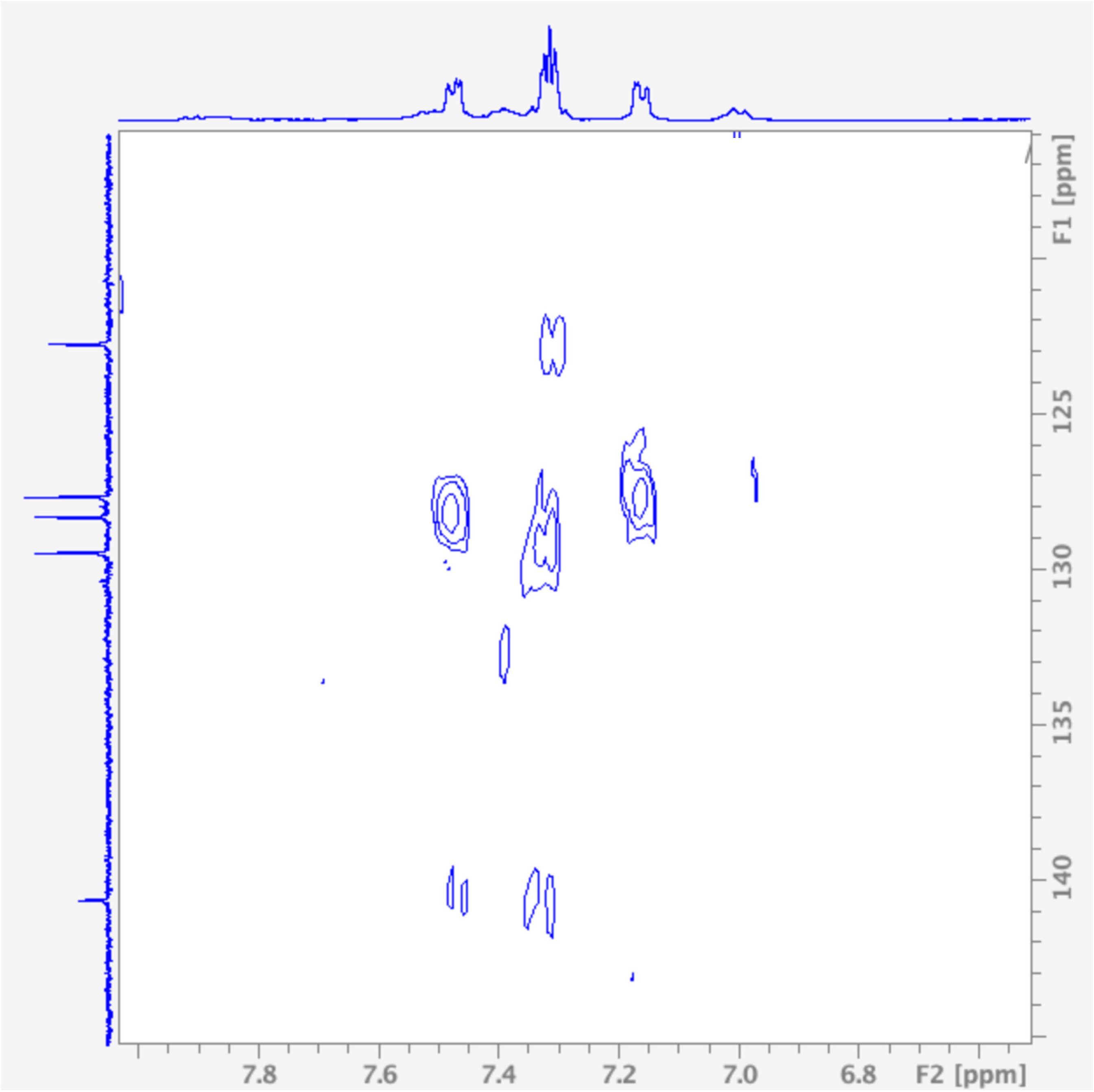
F1 [ppm]
125
130
135
140
7.8
7.6
7.4
7.2
7.0
6.8
F2 [ppm]

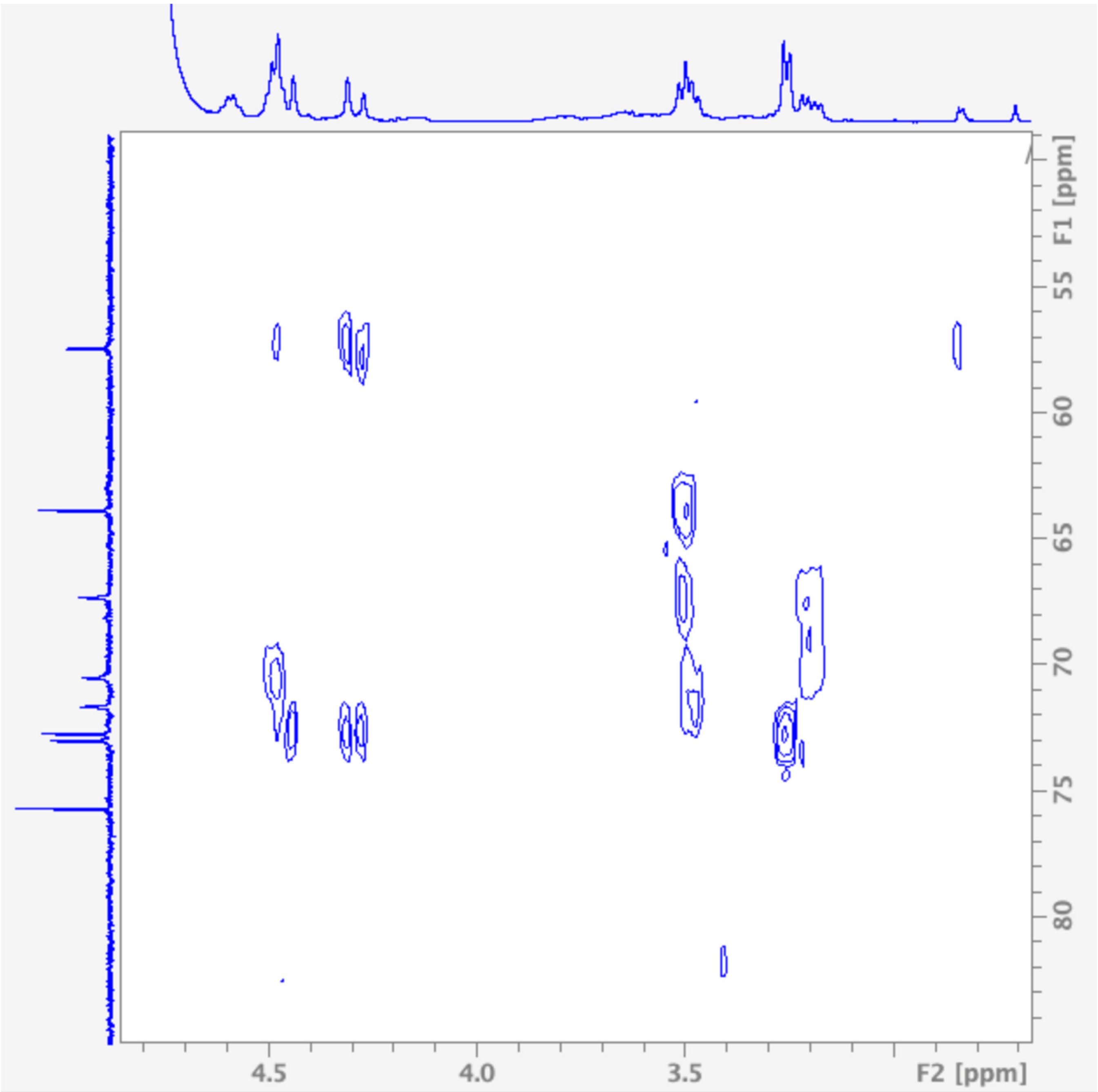

F1 [ppm]
55
60
65
70
75
80
4.5
4.0
3.5
F2 [ppm]

Figure S11. ^{1}H-NMR spectrum (400 MHz, D_2O) of final compound **ortho 4** with colour-coded signals, highlighting the boronic acid and boronate forms they belong to, with interpretation of the isolated signals and tentative interpretation of the overlapping ones. Namely, the orange designates the boronic acid form and indigo designates the boronate form. A) section 8.00 ppm to 6.90 ppm; B) section 4.65 ppm to 3.00 ppm; C) section 1.80 ppm to 1.10 ppm. Highlighted are also the principal COSY correlations to hydrogen atoms within the same spin systems.

A

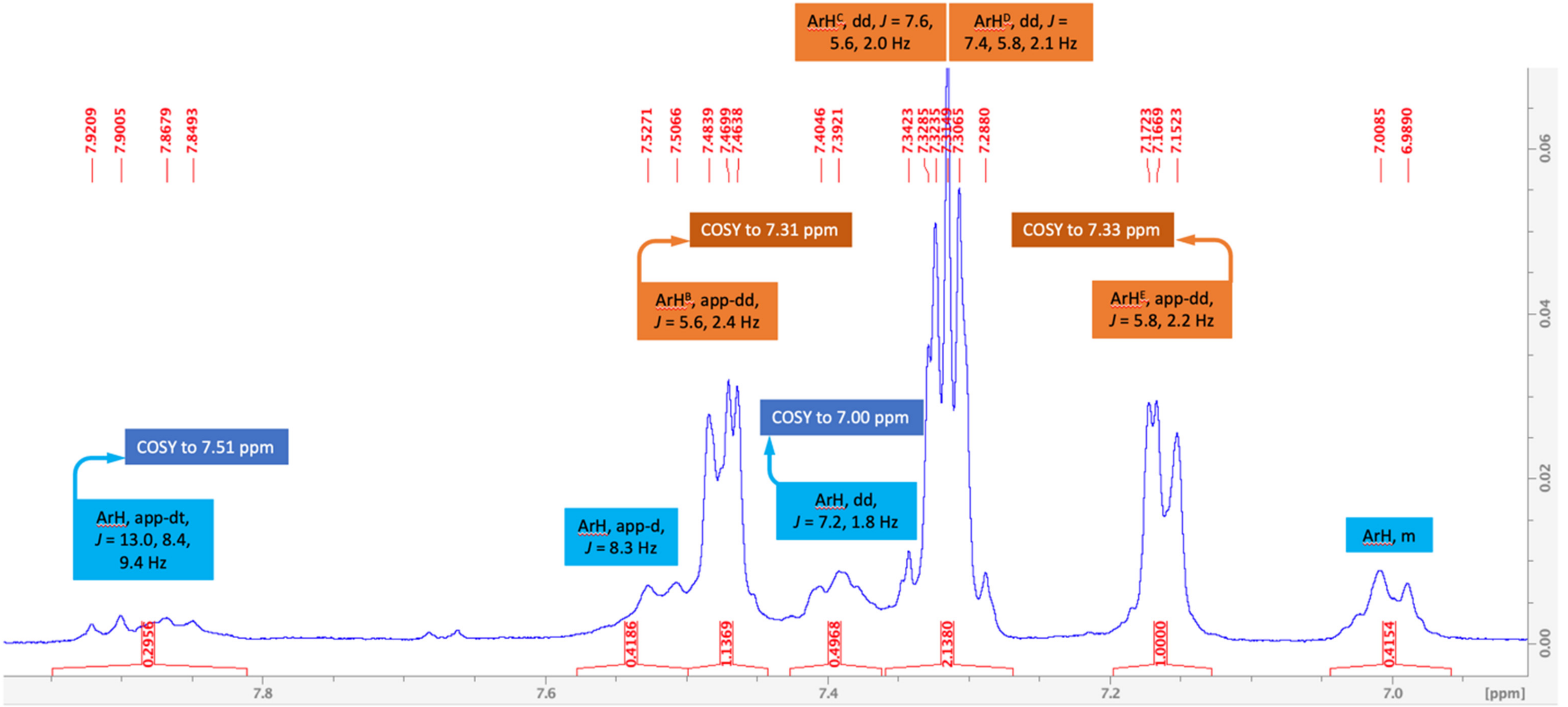

B

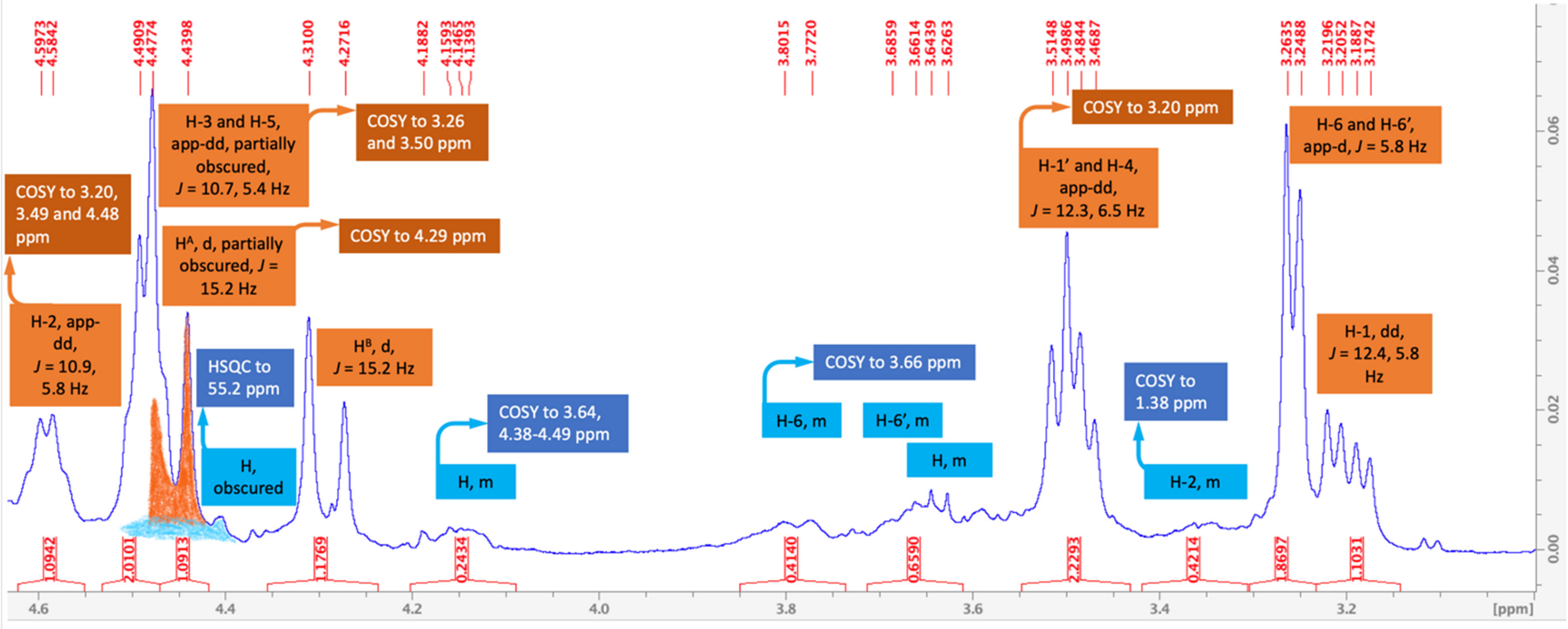

H-3 and H-5, app-dd, partially obscured, J = 10.7, 5.4 Hz
COSY to 3.26 and 3.50 ppm
COSY to 3.20, 3.49 and 4.48 ppm
H^A, d, partially obscured, J = 15.2 Hz
COSY to 4.29 ppm
H-2, app-dd, J = 10.9, 5.8 Hz
HSQC to 55.2 ppm
H, obscured
H^B, d, J = 15.2 Hz
COSY to 3.64, 4.38-4.49 ppm
H, m
COSY to 3.66 ppm
H-6, m
H-6', m
H, m
COSY to 3.20 ppm
H-1' and H-4, app-dd, J = 12.3, 6.5 Hz
COSY to 1.38 ppm
H-2, m
H-6 and H-6', app-d, J = 5.8 Hz
H-1, dd, J = 12.4, 5.8 Hz
[ppm]

C

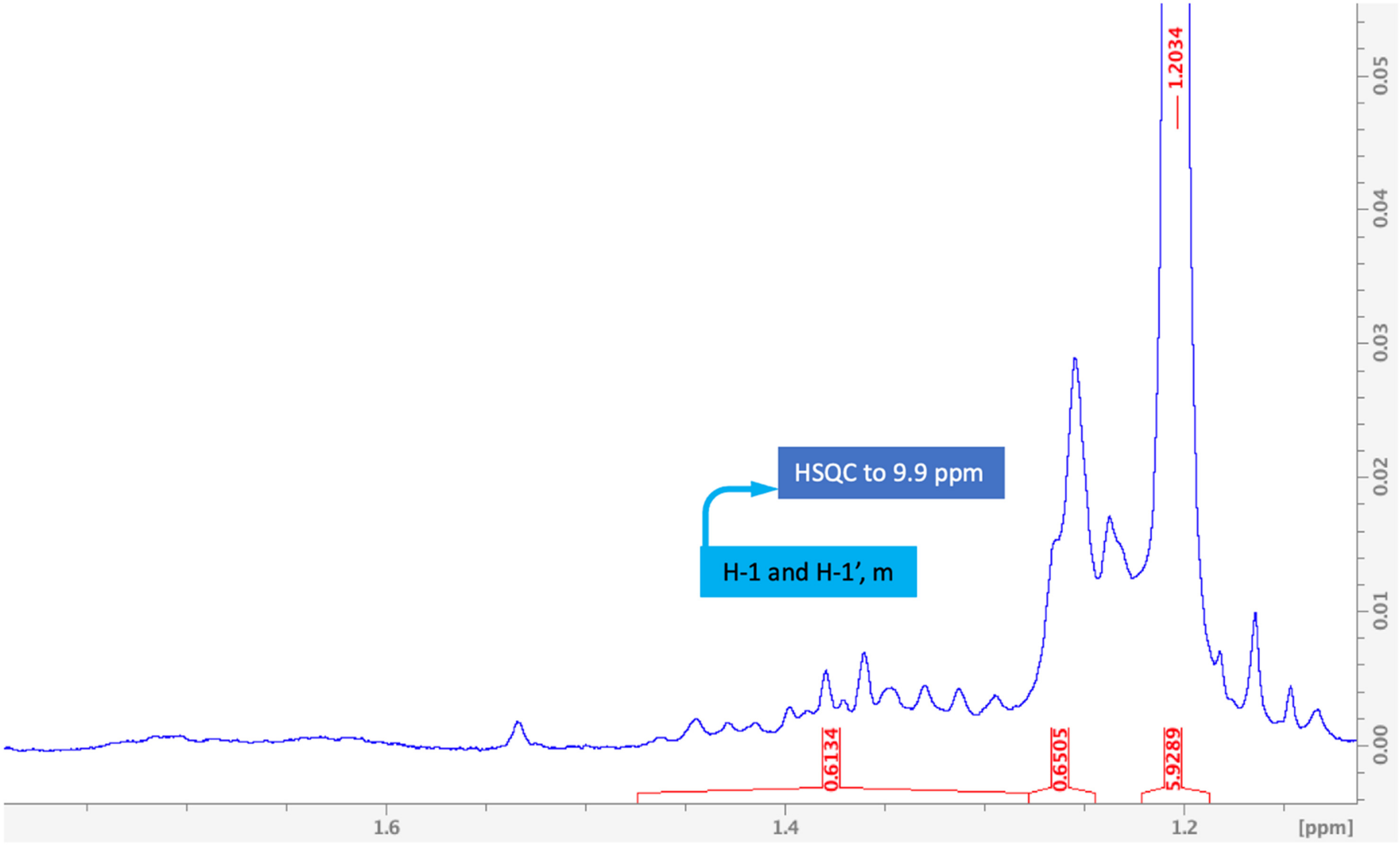
HSQC to 9.9 ppm
H-1 and H-1’, m
1.2034
0.6134
0.6505
5.9289
1.6
1.4
1.2
[ppm]
0.05
0.04
0.03
0.02
0.01
0.00

Figure S12. ^{13}C-NMR spectrum (100 MHz, D_2O) sections of final compound **ortho 4** with colour-coded signals, highlighting the boronic acid and boronate forms they belong to, with interpretation of the isolated signals and tentative interpretation of the overlapping ones. Namely, the orange designates the boronic acid form and indigo designates the boronate form. A) section 144 ppm to 109 ppm; B) section 76 ppm to 44 ppm.

A

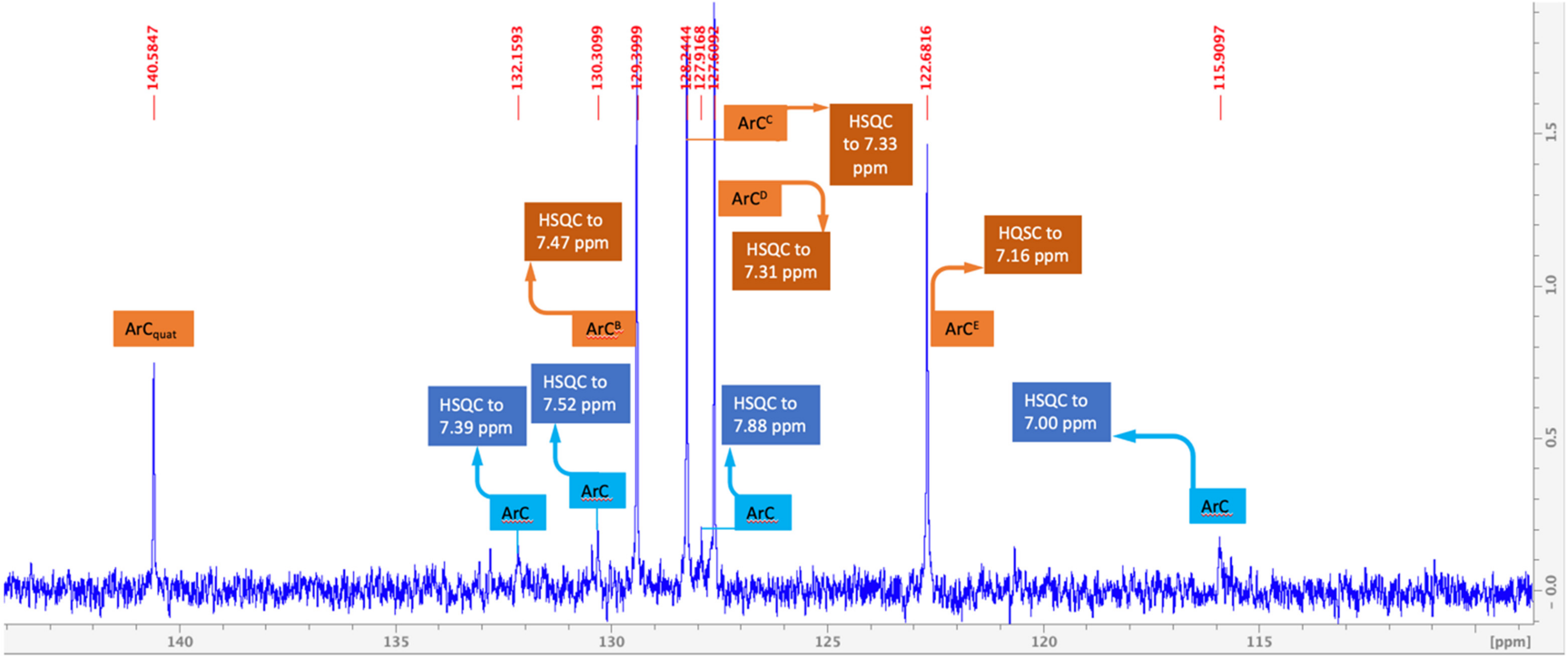

B

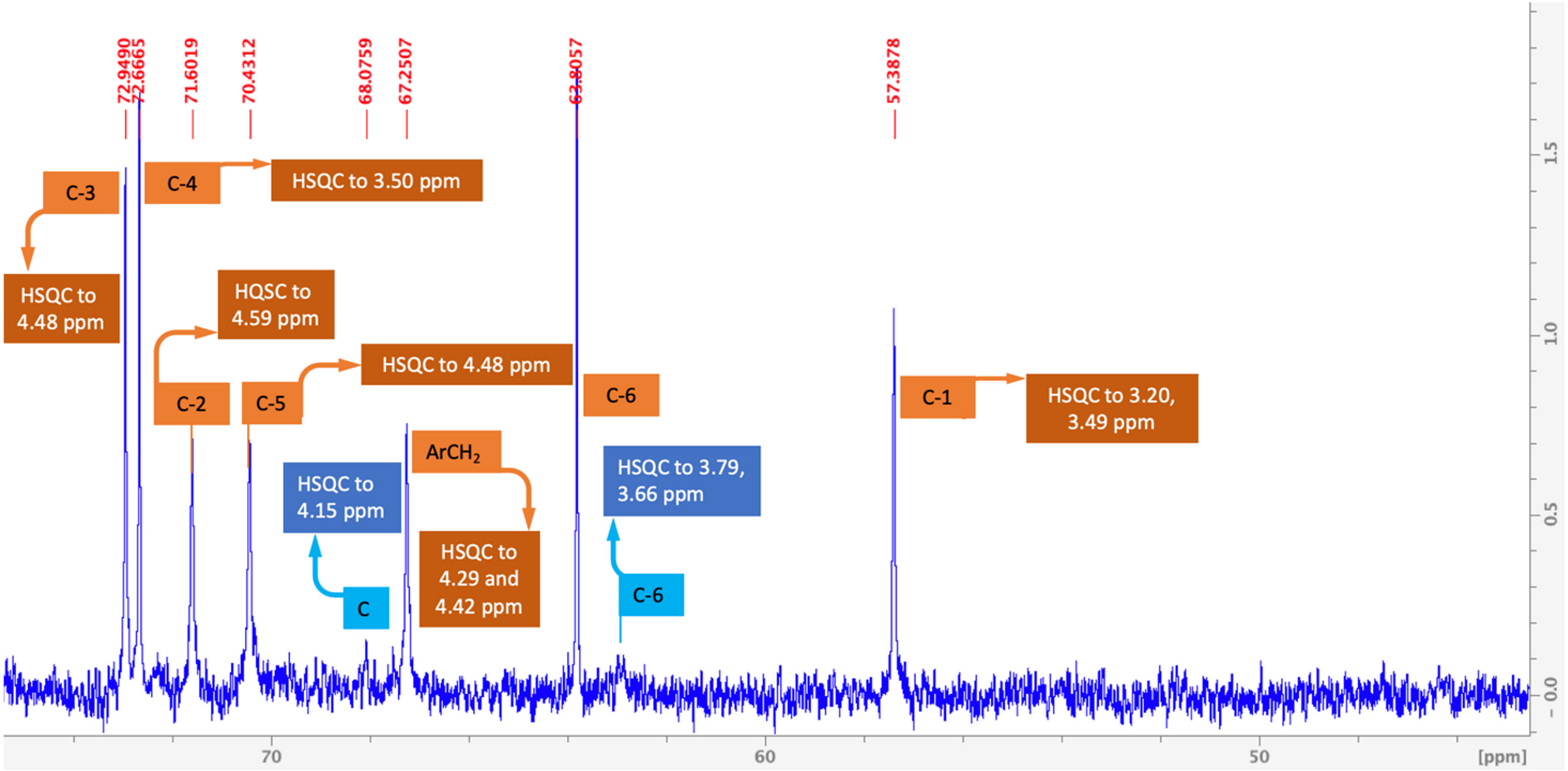

72.9490
72.6665
71.6019
70.4312
68.0759
67.2507
63.8057
57.3878
C-3
HSQC to 4.48 ppm
C-4
HSQC to 3.50 ppm
HQSC to 4.59 ppm
C-2
C-5
HSQC to 4.48 ppm
C-6
C-1
HSQC to 3.20, 3.49 ppm
ArCH$_2$
HSQC to 4.15 ppm
C
HSQC to 4.29 and 4.42 ppm
HSQC to 3.79, 3.66 ppm
C-6
70
60
50
[ppm]
0.0
0.5
1.0
1.5

Figure S13. ^{1}H- (400 MHz), ^{13}C-NMR (100 MHz), ^{11}B-NMR (128 MHz), COSY and HSQC spectra of *N*-(3-methylphenyl boronic acid)-3,6-dideoxy-3,6-imino-1,2-*O*-isopropylidene-*α*-D-gulofuranose **meta 5** in MeOD.

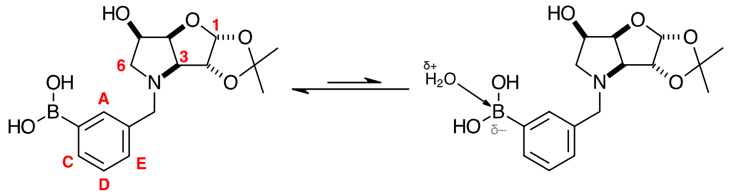

Boronic acid species. **δH**: 7.57 (1H, d, $J_{HA,HE}$ 1.5 Hz, ArH^A), 7.49 (1H, d, $J_{HC,HD}$ 7.2 Hz, ArH^C), 7.37 (1H, dt, $J_{HE,HD}$ 7.6 Hz, $J_{HE,HA}$ 1.5 Hz, ArH^E), 7.32 (1H, app-t, $J_{HD,HC=HD,HE}$ 7.3 Hz, ArH^D), 5.93 (1H, d, $J_{H\text{-}1,H\text{-}2}$ 3.3 Hz, H-1), 4.74 (1H, app-t, $J_{H\text{-}4,H\text{-}3=H\text{-}4,H\text{-}5}$ 5.5 Hz, H-4), 4.46 (1H, d, $J_{H\text{-}2,H\text{-}1}$ 3.3, H-2), 4.13 (1H, ddd, $J_{H\text{-}5,H\text{-}4}$ 5.6 Hz, $J_{H\text{-}5,H\text{-}6}$ 5.5 Hz, $J_{H\text{-}5,H\text{-}6'}$ 3.7 Hz, H-5), 3.91 (1H, d, $J_{Ha,Hb}$ 13.2 Hz, ArC*H*^{a}H^b), 3.59 (1H, d, $J_{Hb,Ha}$ 13.2 Hz, ArCH^a*H*^{b}), 3.30 (1H, d, partially obscured, $J_{H\text{-}3,H\text{-}4}$ 5.7 Hz, H-3), 2.83 (1H, dd, $J_{H\text{-}6',H\text{-}6}$ 10.6 Hz, $J_{H\text{-}6',H\text{-}5}$ 3.5 Hz, H-6′), 2.53 (1H, dd, $J_{H\text{-}6,H\text{-}6'}$ 10.3 Hz, $J_{H\text{-}6,H\text{-}5}$ 5.7 Hz, H-6), 1.44 (3H, s, CH_3 acetonide), 1.27 (3H, s, CH_3 acetonide); **δC**: 138.5 (ArC$_{quat}$), 135.2 (ArC^A), 133.4 (ArC^C), 131.4 (ArC^E), 128.7 (ArC^D), 113.4 (C$_{quat}$ acetonide), 108.8 (C-1), 85.8 (C-2), 84.8 (C-4), 73.4 (C-3), 71.2 (C-5), 60.3 (C-6), 59.9 (CH_2Ar), 27.8, 27.0 (2 x CH_3, acetonide); ArC^B-B was not discernible.

Boronate species (not all signals are visible). **δH**: 7.12 (1H, td, $J_{HC,HD}$ 7.6 Hz, $J_{HC,HE}$ 0.8 Hz, ArH^C), 6.79 (1H, dd, $J_{HE,HD}$ 7.6 Hz, $J_{HE,HA}$ 1.5 Hz, ArH^E), 7.32 (1H, ddd, $J_{HD,HE}$ 8.2 Hz, $J_{HD,HC}$ 2.4 Hz, $J_{HD,HA}$ 0.9 Hz, ArH^D), 5.83 (1H, d, $J_{H\text{-}1,H\text{-}2}$ 3.3 Hz, H-1), 4.54 (1H, d, $J_{H\text{-}4,H\text{-}3}$ 3.5 Hz, H-4), 4.49 (1H, d, $J_{H\text{-}2,H\text{-}1}$ 3.4, H-2), 3.84, 3.83 (2 x 1H, 2 x d, *J* 12.7 Hz, ArCH^{a}H^b), 1.61 (1H, dd, $J_{H\text{-}6,H\text{-}6'}$ 13.0 Hz, $J_{H\text{-}6,H\text{-}5}$ 6.6 Hz, H-6), 1.59 (1H, dd, $J_{H\text{-}6',H\text{-}6}$ 12.8 Hz, $J_{H\text{-}6',H\text{-}5}$ 6.6 Hz, H-6′), 1.44 (3H, s, partially obscured, CH_3 acetonide), 1.32 (3H, s, CH_3 acetonide); **δC**: not discernible.

δB: 28.6 (sharp, integration: 8.6), 18.6 (sharp, integration: 1.0).

^{1}H-NMR

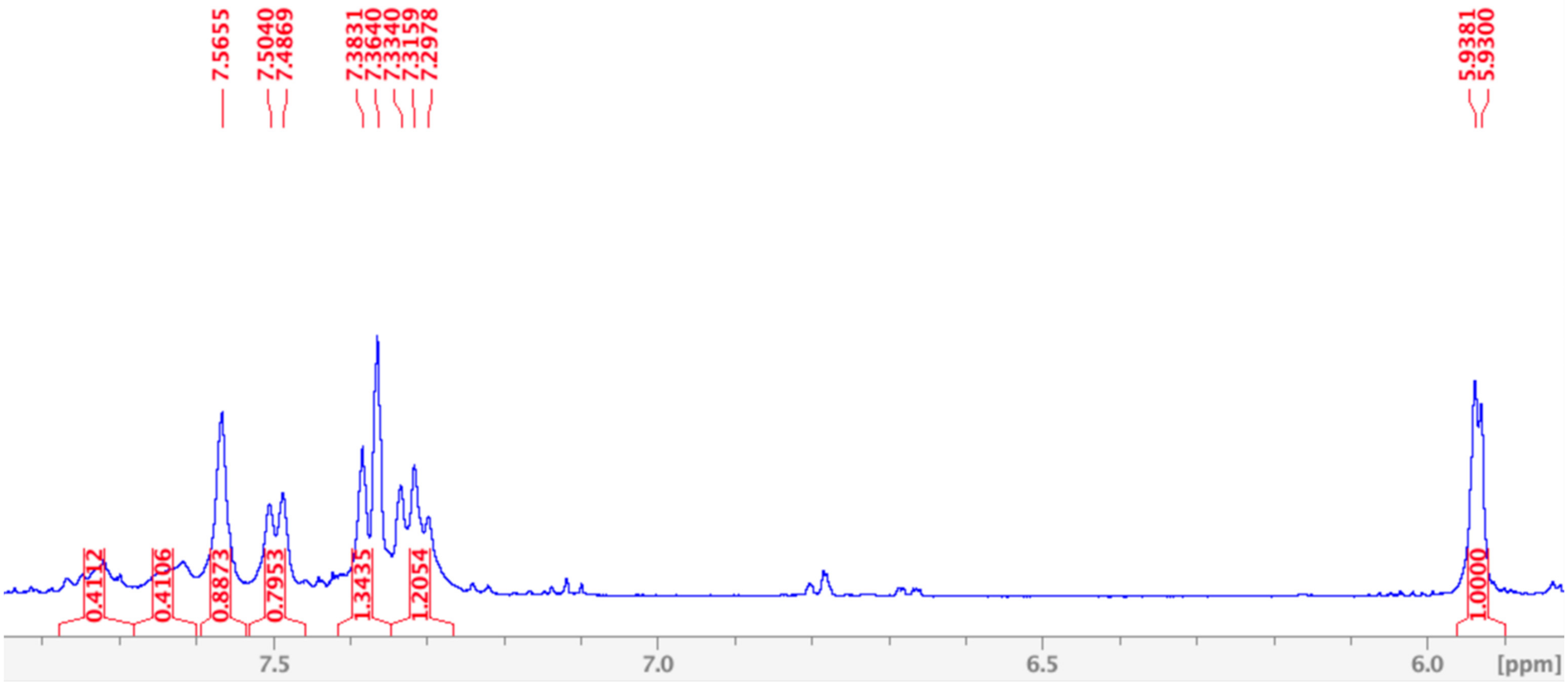

7.5655
7.5040
7.4869
7.3831
7.3640
7.3340
7.3159
7.2978
5.9381
5.9300
0.4112
0.4106
0.8873
0.7953
1.3435
1.2054
1.0000
7.5
7.0
6.5
6.0
[ppm]

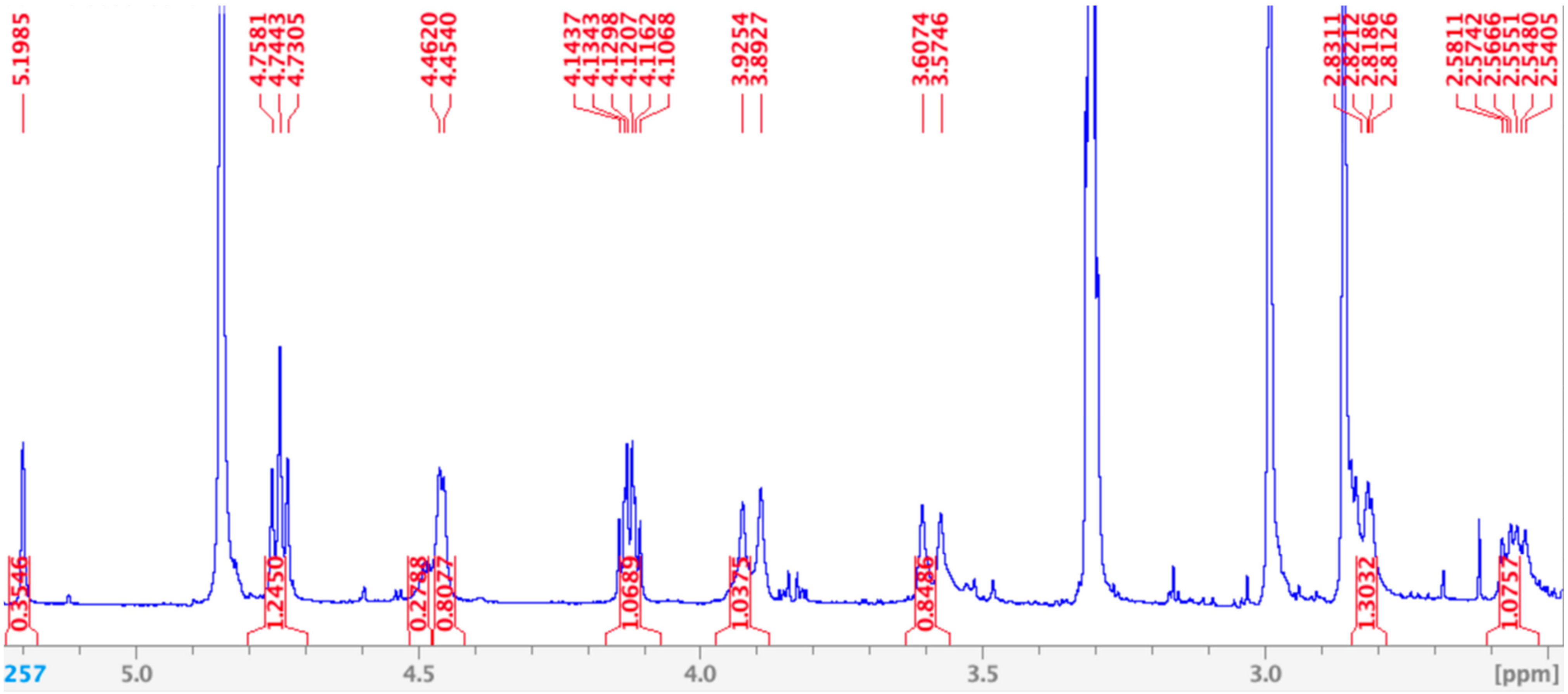
5.1985
4.7581
4.7443
4.7305
4.4620
4.4540
4.1437
4.1343
4.1298
4.1207
4.1162
4.1068
3.9254
3.8927
3.6074
3.5746
2.8311
2.8212
2.8186
2.8126
2.5811
2.5742
2.5666
2.5551
2.5480
2.5405
0.3546
1.2450
0.2788
0.8077
1.0689
1.0375
0.8486
1.3032
1.0757
5.0
4.5
4.0
3.5
3.0
[ppm]

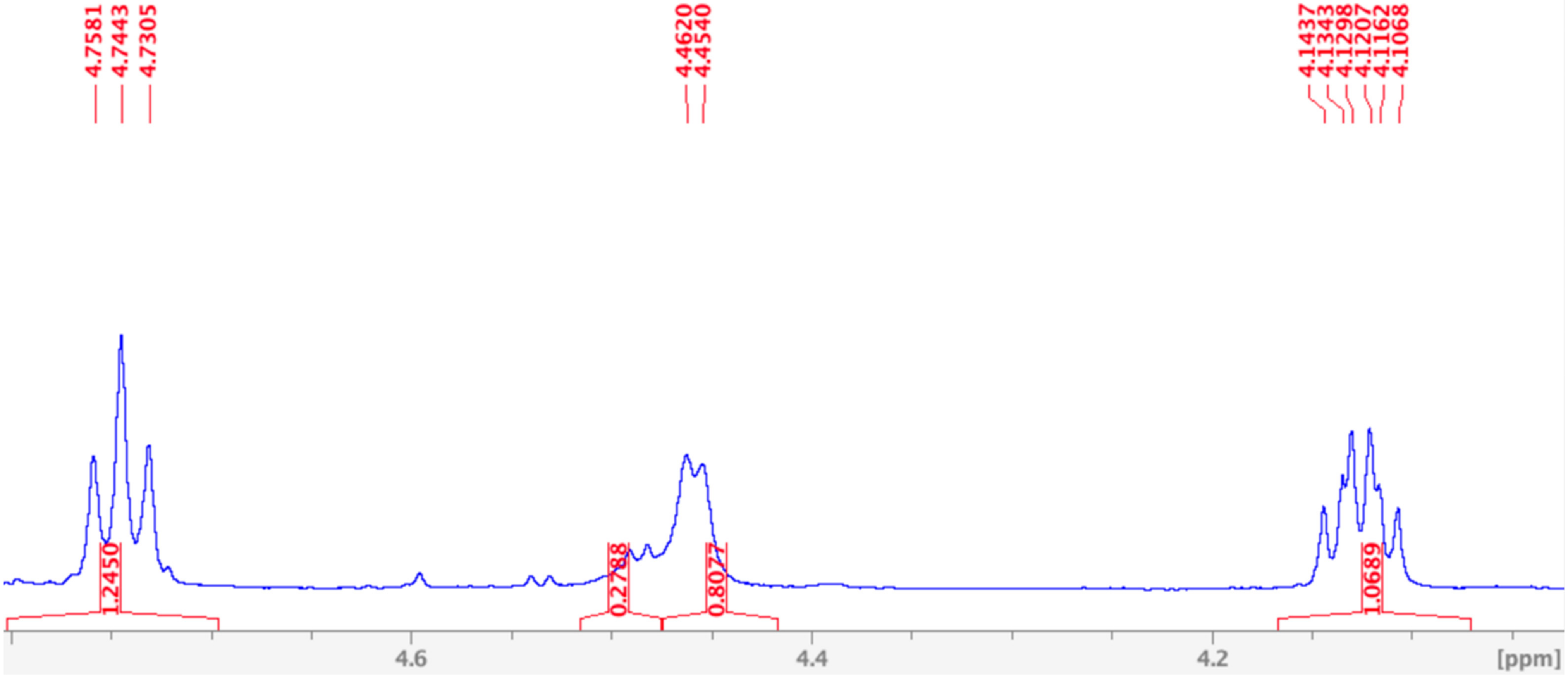
4.7581
4.7443
4.7305
4.4620
4.4540
4.1437
4.1343
4.1298
4.1207
4.1162
4.1068
1.2450
0.2788
0.8077
1.0689
4.6
4.4
4.2
[ppm]

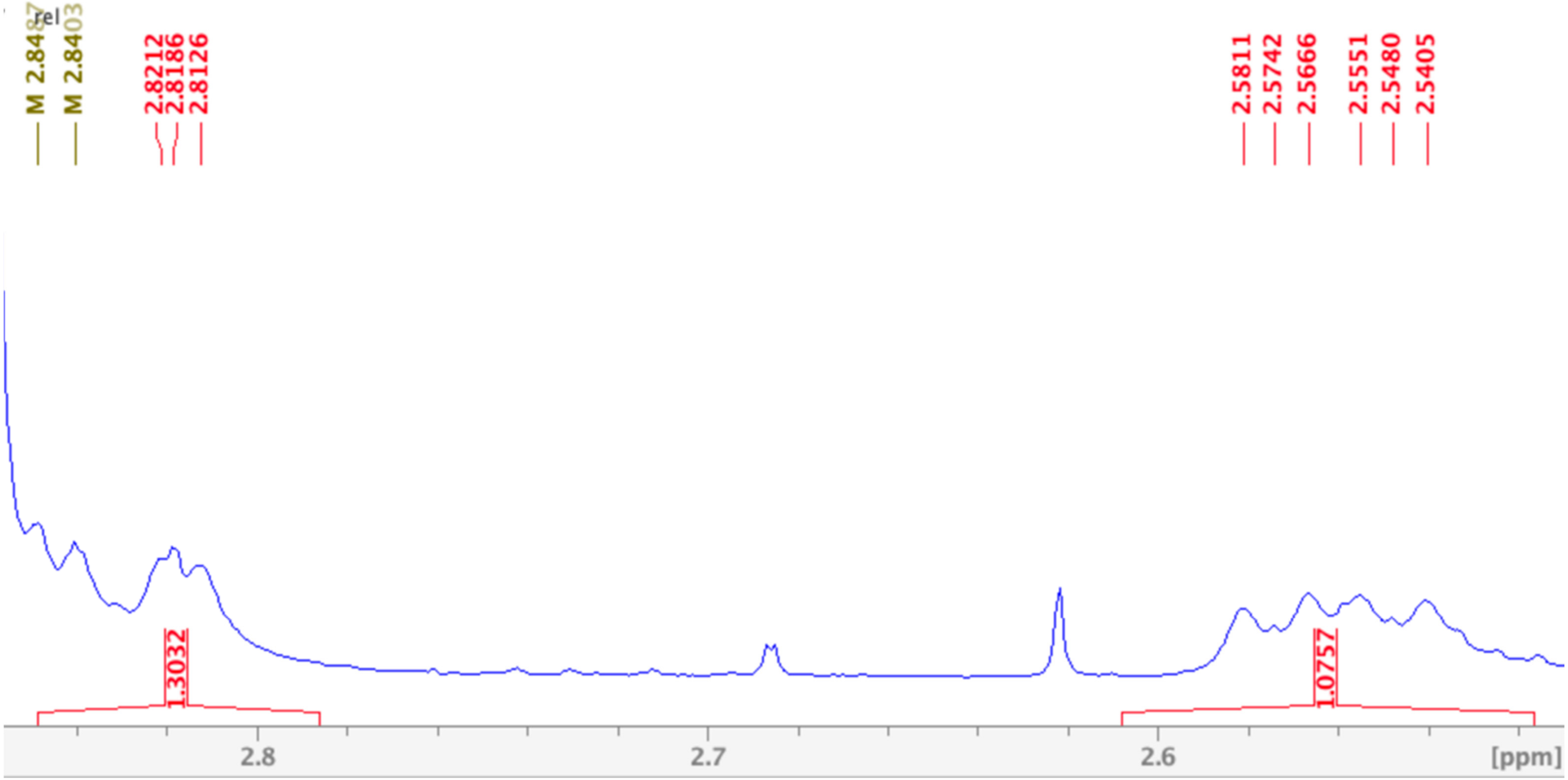

rel
M 2.8487
M 2.8403
2.8212
2.8186
2.8126
2.5811
2.5742
2.5666
2.5551
2.5480
2.5405
1.3032
1.0757
2.8
2.7
2.6
[ppm]

^{13}C-NMR

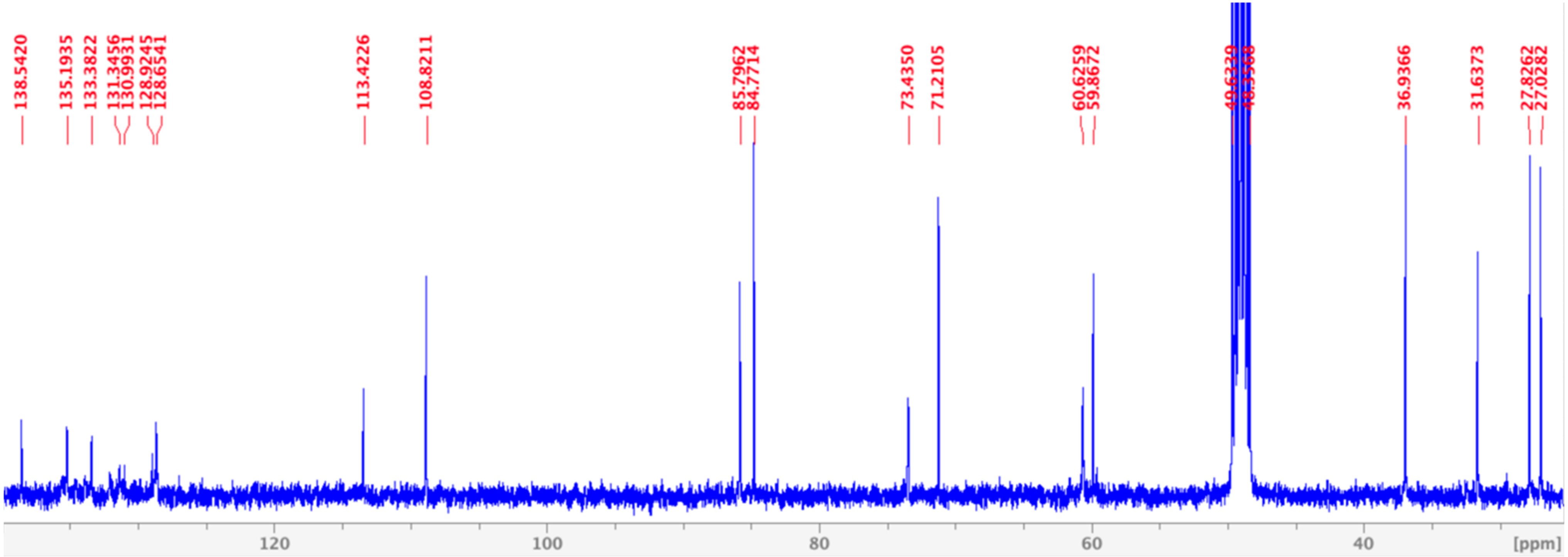
138.5420
135.1935
133.3822
131.3456
130.9931
128.9245
128.6541
113.4226
108.8211
85.7962
84.7714
73.4350
71.2105
60.6259
59.8672
49.6339
48.3568
36.9366
31.6373
27.8262
27.0282
120
100
80
60
40
[ppm]

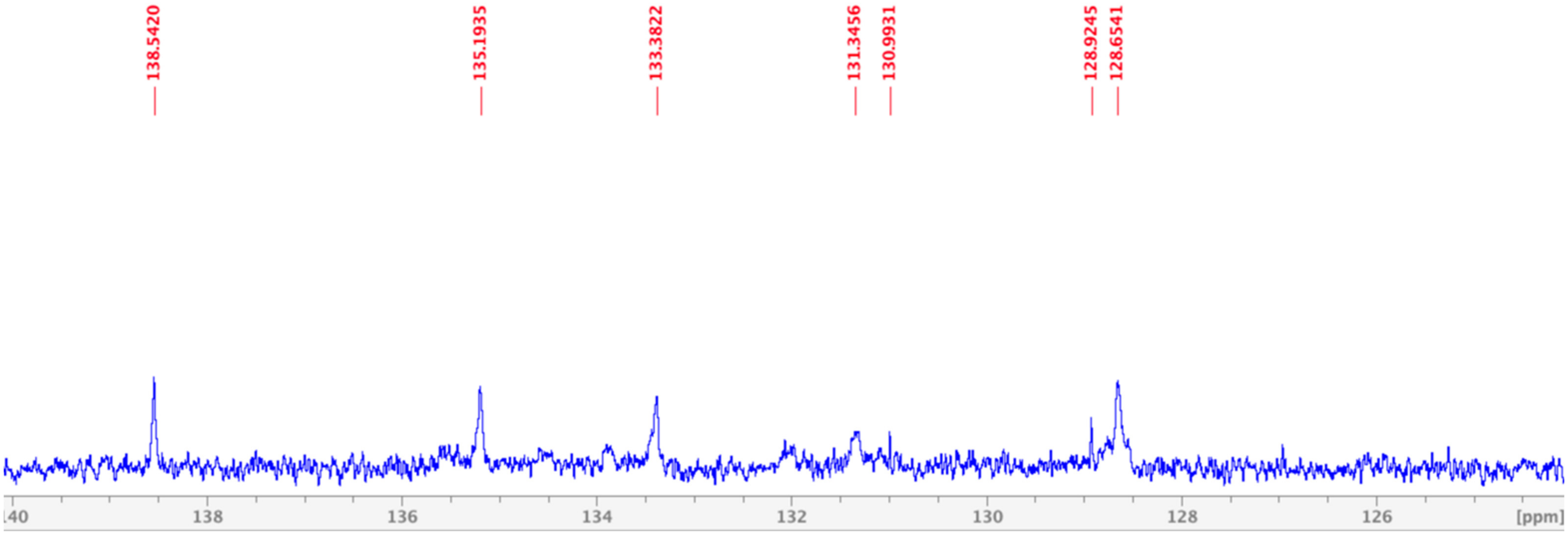
138.5420
135.1935
133.3822
131.3456
130.9931
128.9245
128.6541
140
138
136
134
132
130
128
126
[ppm]

DEPT

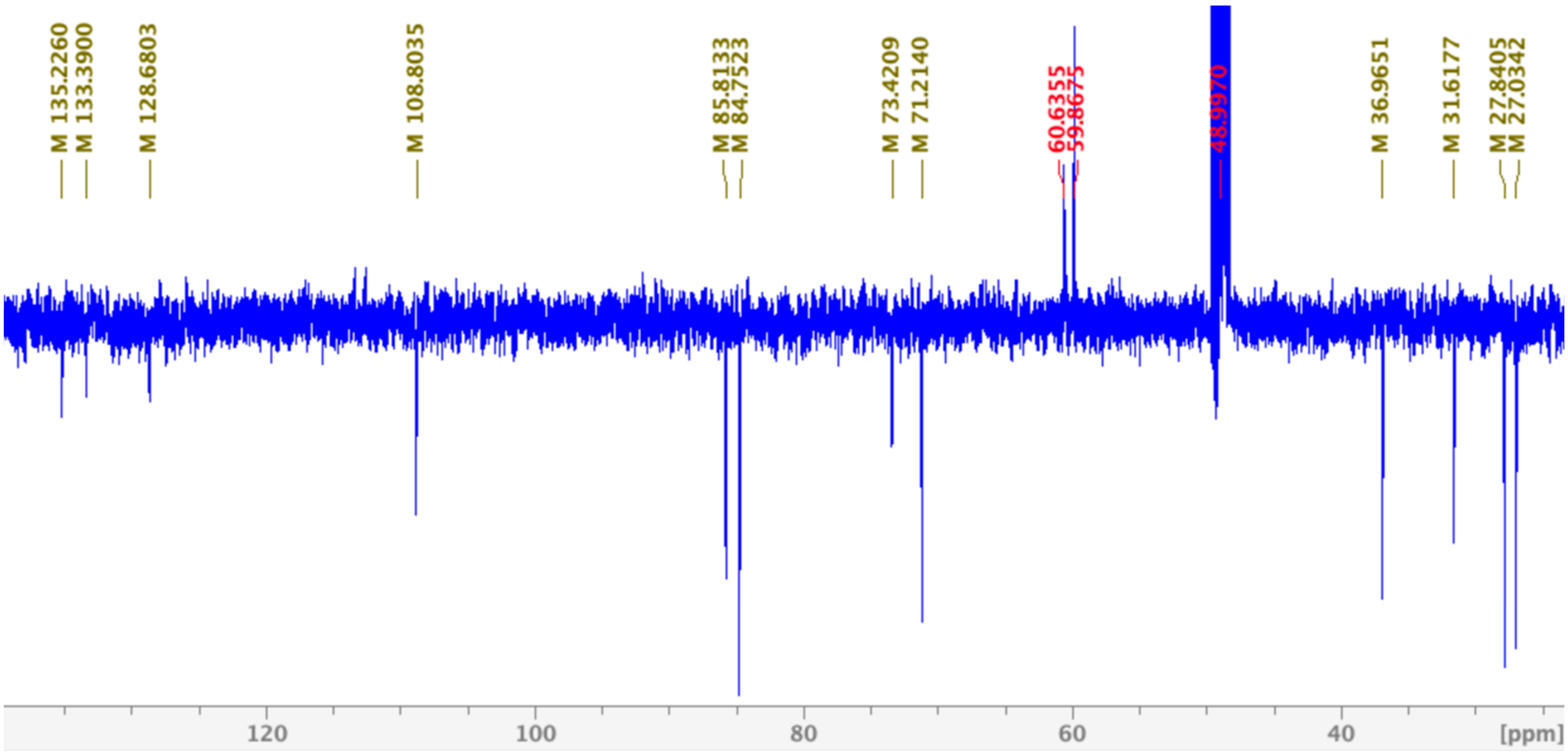
M 135.2260
M 133.3900
M 128.6803
M 108.8035
M 85.8133
M 84.7523
M 73.4209
M 71.2140
60.6355
59.8675
48.9970
M 36.9651
M 31.6177
M 27.8405
M 27.0342
120
100
80
60
40
[ppm]

^{11}B-NMR

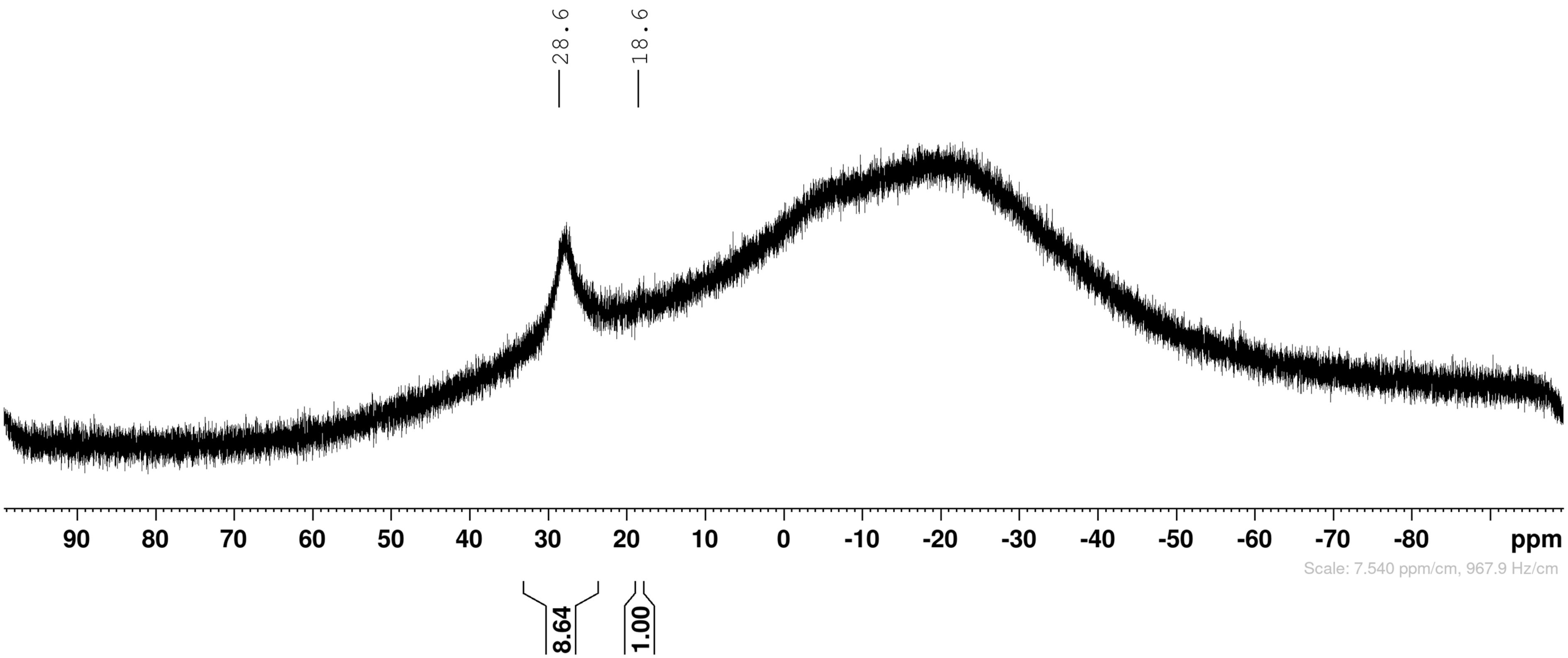

28.6
18.6
90
80
70
60
50
40
30
20
10
0
-10
-20
-30
-40
-50
-60
-70
-80
ppm
Scale: 7.540 ppm/cm, 967.9 Hz/cm
8.64
1.00

COSY

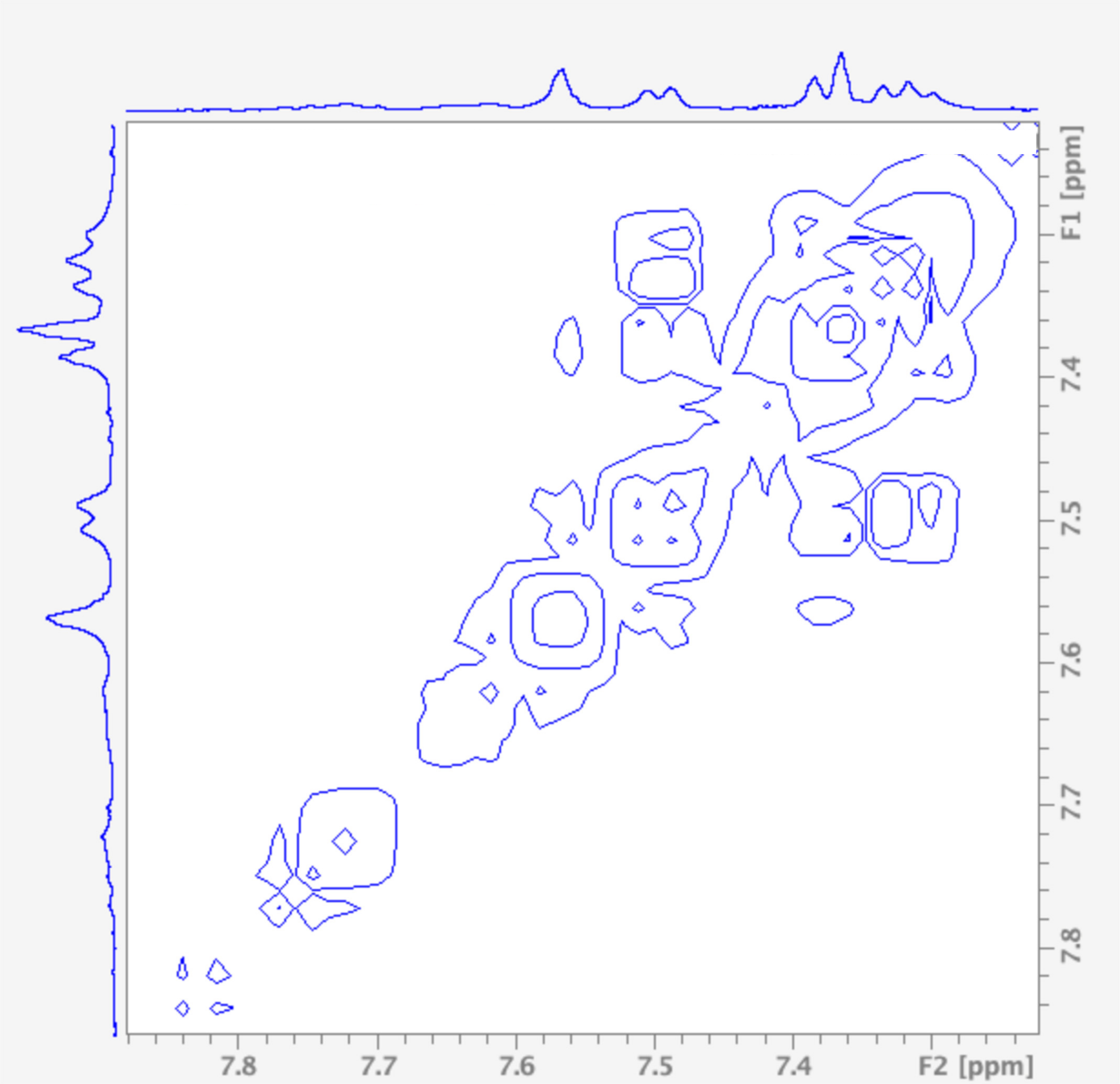

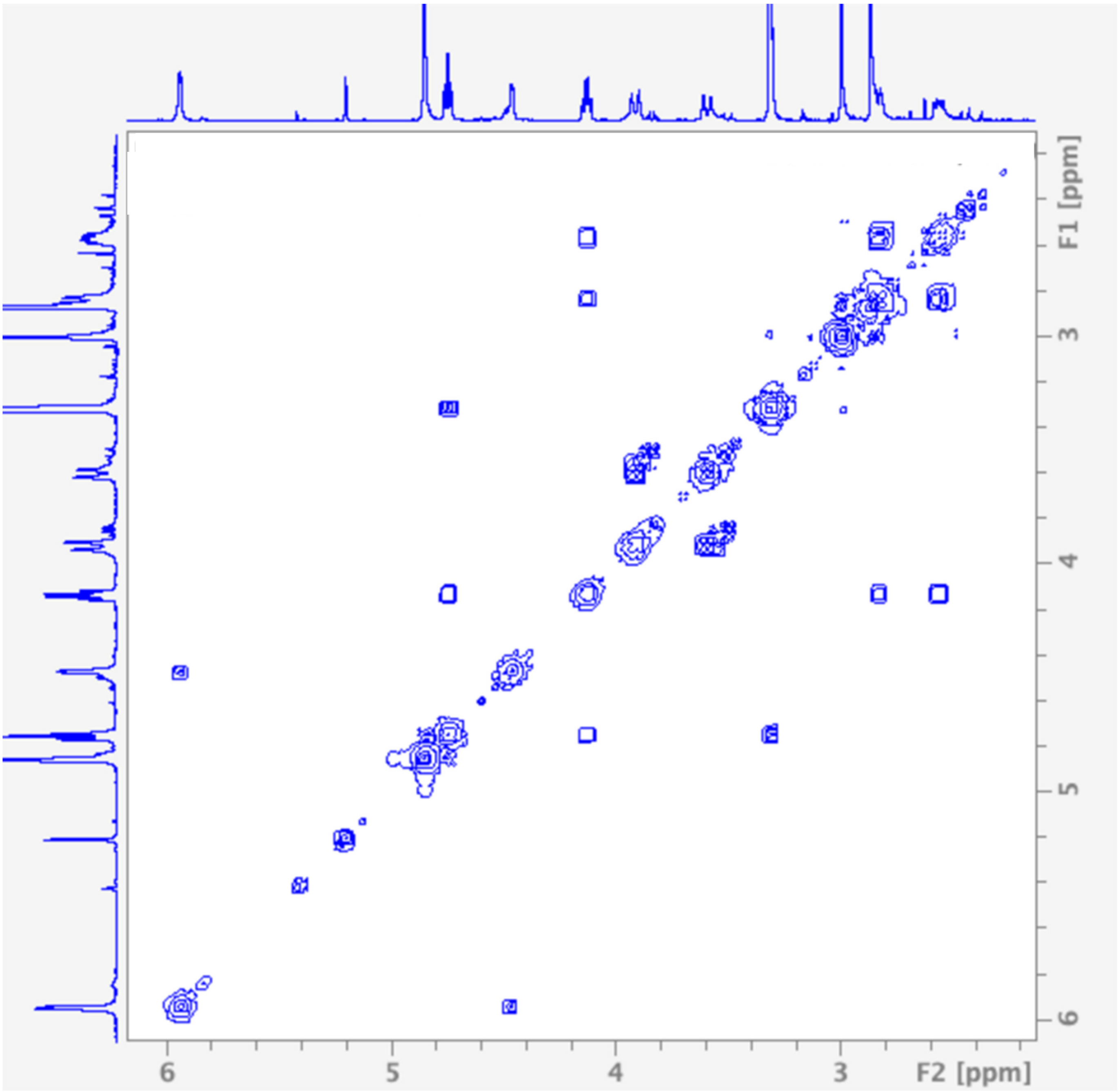

F1 [ppm]
3
4
5
6
6
5
4
3
F2 [ppm]

HSQC

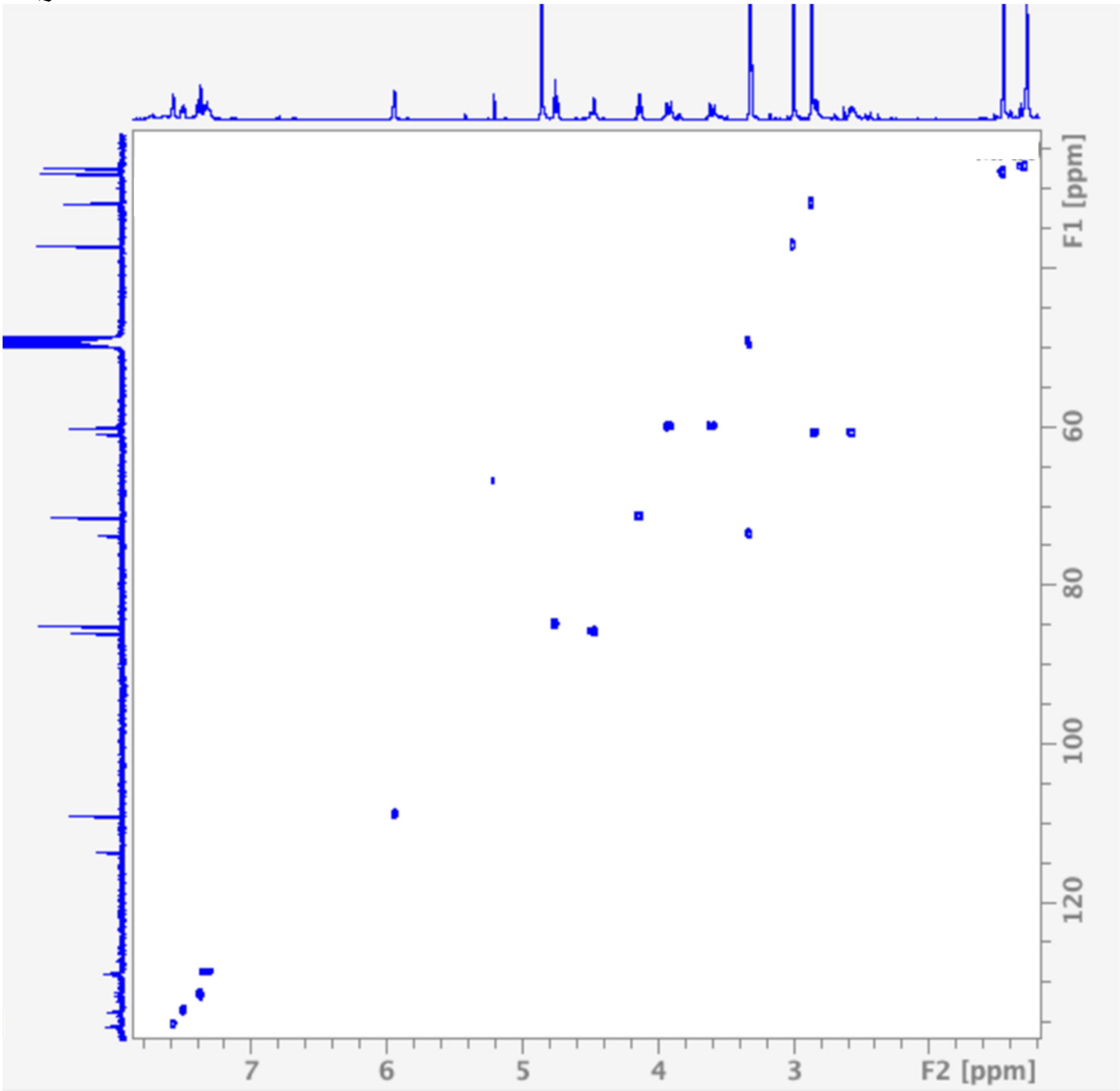

Figure S14. ^{1}H-NMR spectrum (400 MHz, MeOD) of compound **meta 5** with colour-coded signals, highlighting the boronic acid form they belong to, with interpretation of the isolated signals and tentative interpretation of the overlapping ones. Namely, the orange designates the boronic acid form. A) section 7.82 ppm to 7.00 ppm; B) section 6.00 ppm to 4.00 ppm; C) section 4.00 ppm to 2.50 ppm; D) section 1.50 ppm to 1.00 ppm. Highlighted are also the principal COSY correlations to hydrogen atoms within the same spin systems.

A

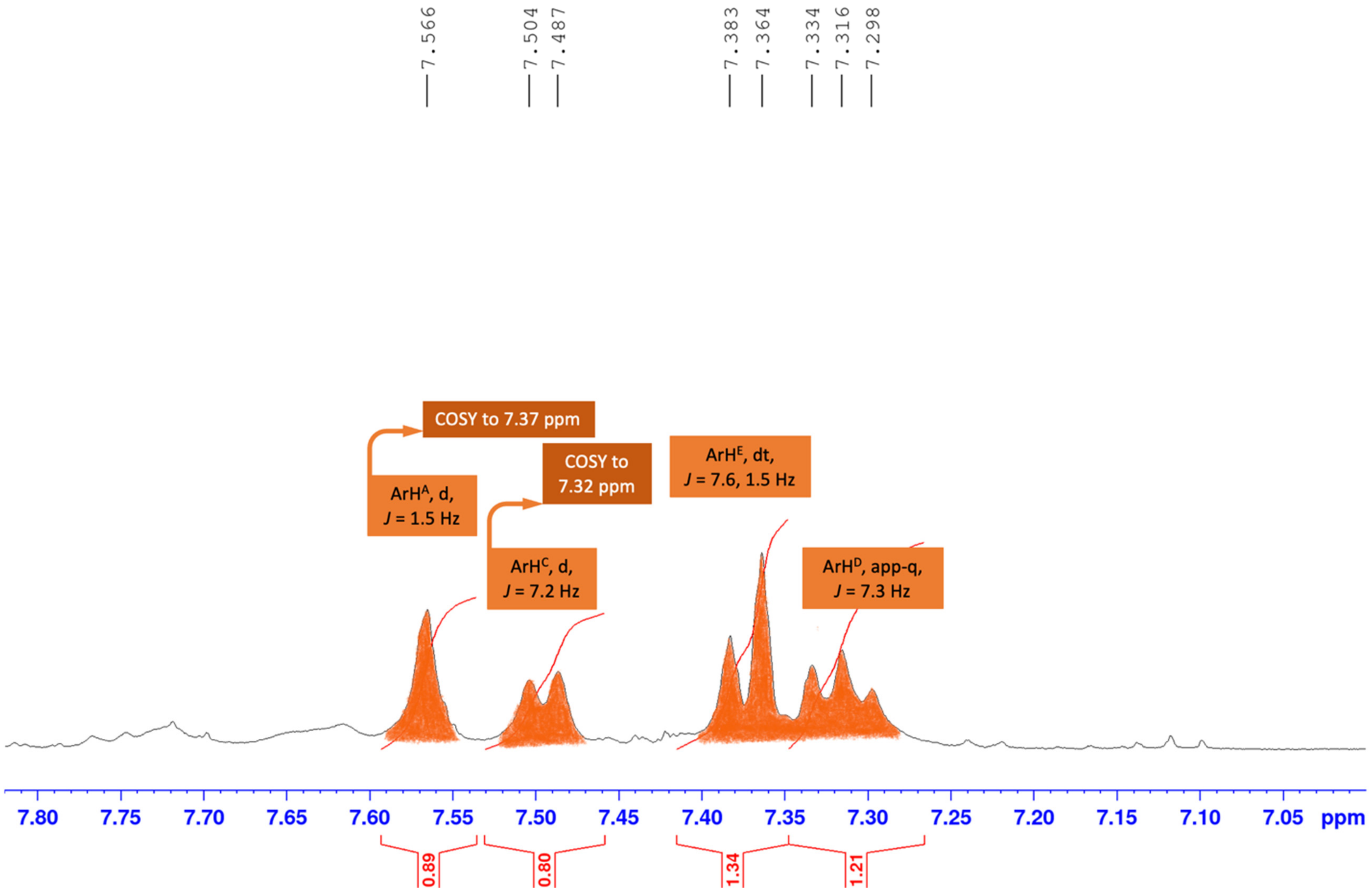

B

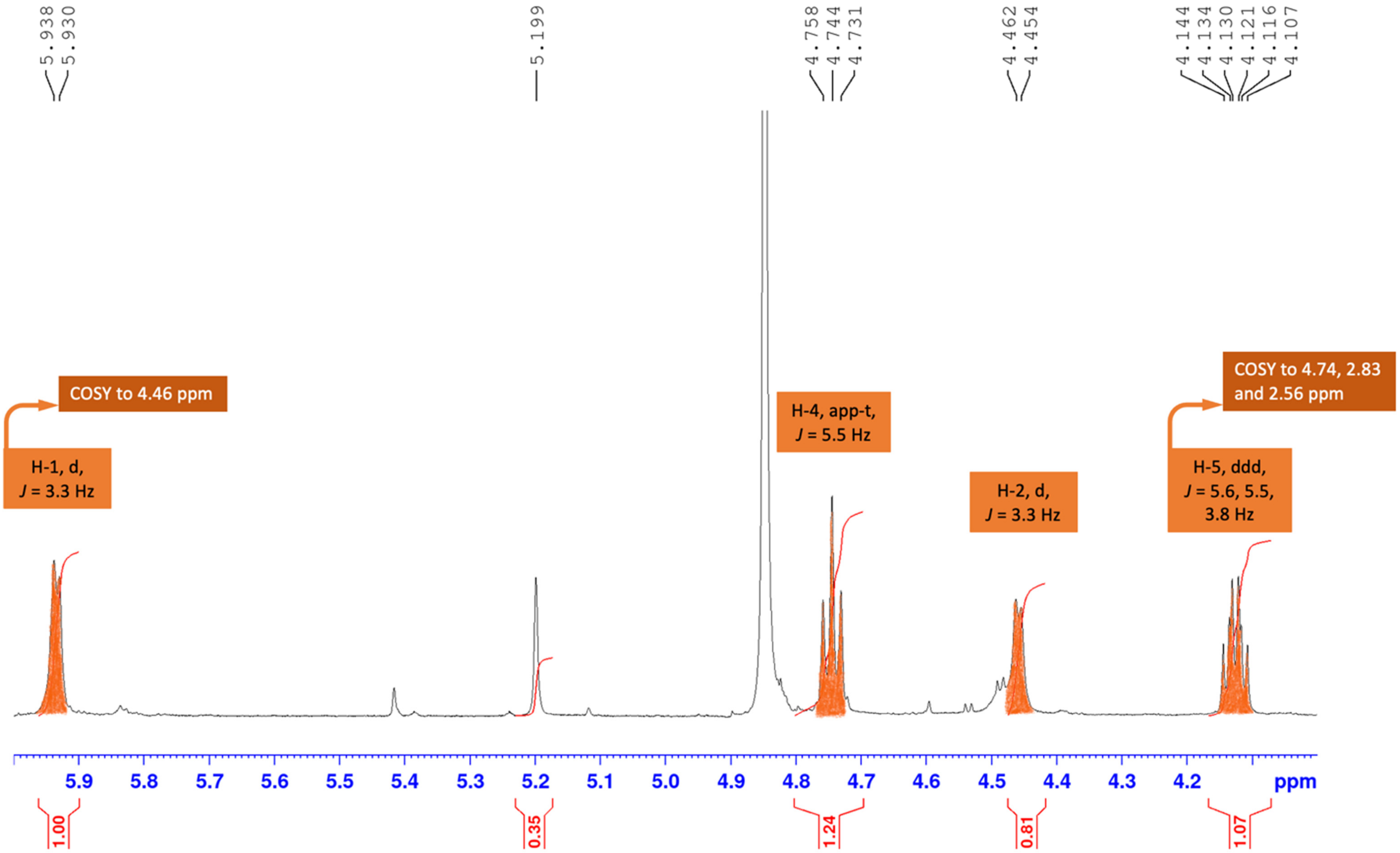
5.938
5.930
5.199
4.758
4.744
4.731
4.462
4.454
4.144
4.134
4.130
4.121
4.116
4.107
COSY to 4.46 ppm
H-1, d,
J = 3.3 Hz
H-4, app-t,
J = 5.5 Hz
H-2, d,
J = 3.3 Hz
COSY to 4.74, 2.83
and 2.56 ppm
H-5, ddd,
J = 5.6, 5.5,
3.8 Hz
5.9
5.8
5.7
5.6
5.5
5.4
5.3
5.2
5.1
5.0
4.9
4.8
4.7
4.6
4.5
4.4
4.3
4.2
ppm
1.00
0.35
1.24
0.81
1.07

C

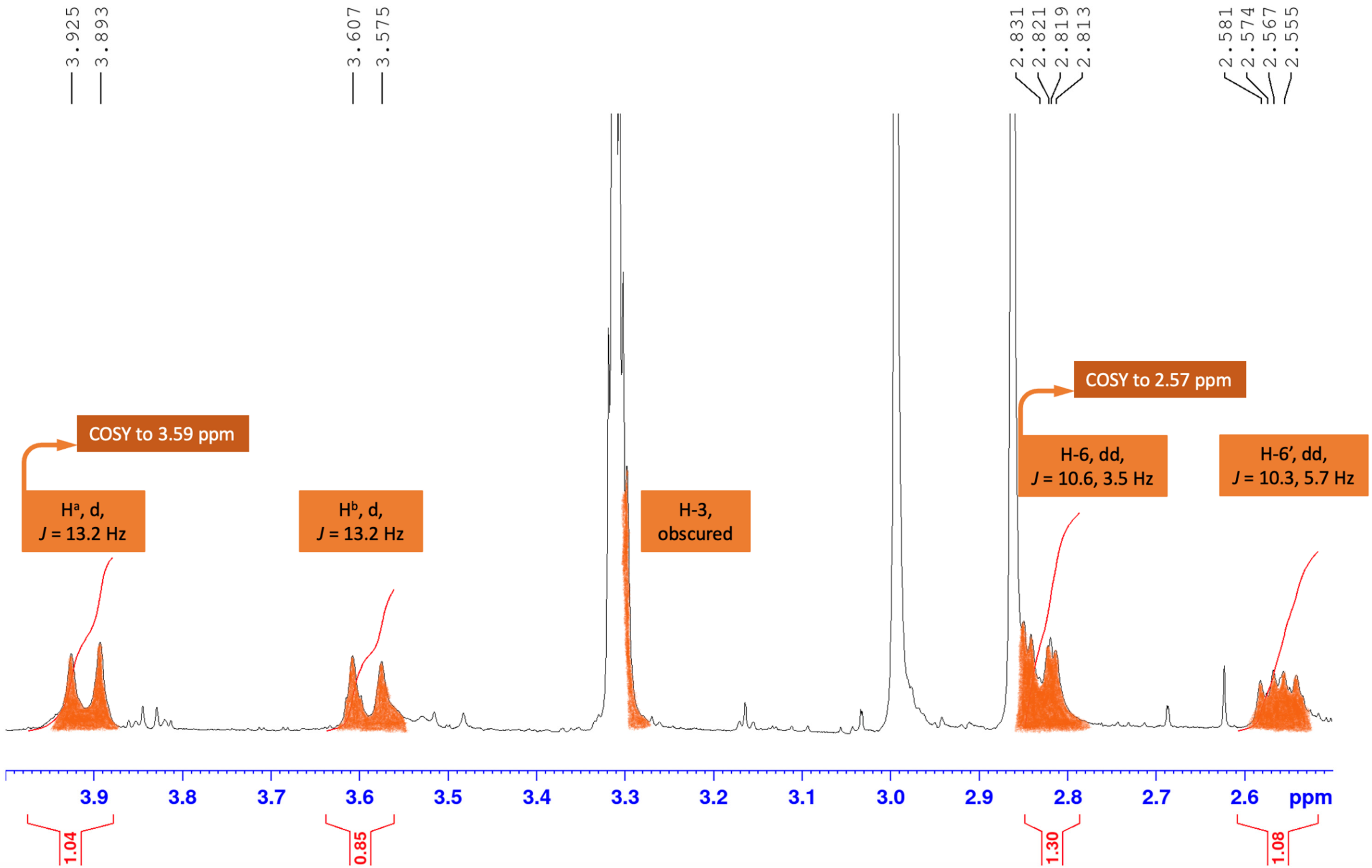

3.925
3.893
3.607
3.575
2.831
2.821
2.819
2.813
2.581
2.574
2.567
2.555
COSY to 3.59 ppm
Hª, d, J = 13.2 Hz
Hᵇ, d, J = 13.2 Hz
H-3, obscured
COSY to 2.57 ppm
H-6, dd, J = 10.6, 3.5 Hz
H-6', dd, J = 10.3, 5.7 Hz
3.9
3.8
3.7
3.6
3.5
3.4
3.3
3.2
3.1
3.0
2.9
2.8
2.7
2.6
ppm
1.04
0.85
1.30
1.08

D

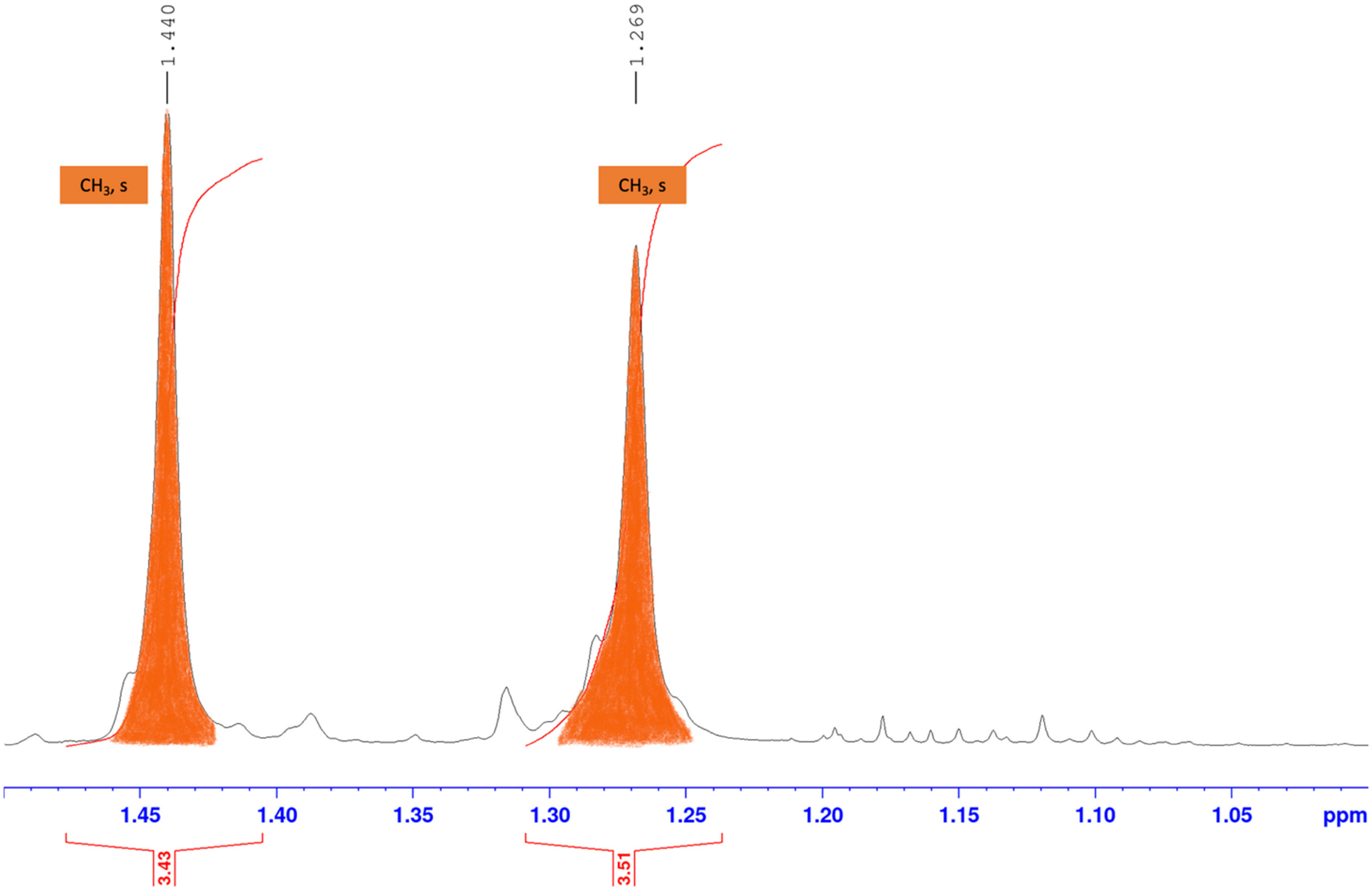
1.440
1.269
CH3, s
CH3, s
1.45
1.40
1.35
1.30
1.25
1.20
1.15
1.10
1.05
ppm
3.43
3.51

Figure S15. ^{13}C-NMR spectrum (100 MHz, MeOD) of compound **meta 5** with colour-coded signals, highlighting the boronic acid form they belong to, with interpretation of the isolated signals and tentative interpretation of the overlapping ones. Namely, the orange designates the boronic acid form. A) section 141 ppm to 25 ppm; B) section 140 ppm to 120 ppm.

A

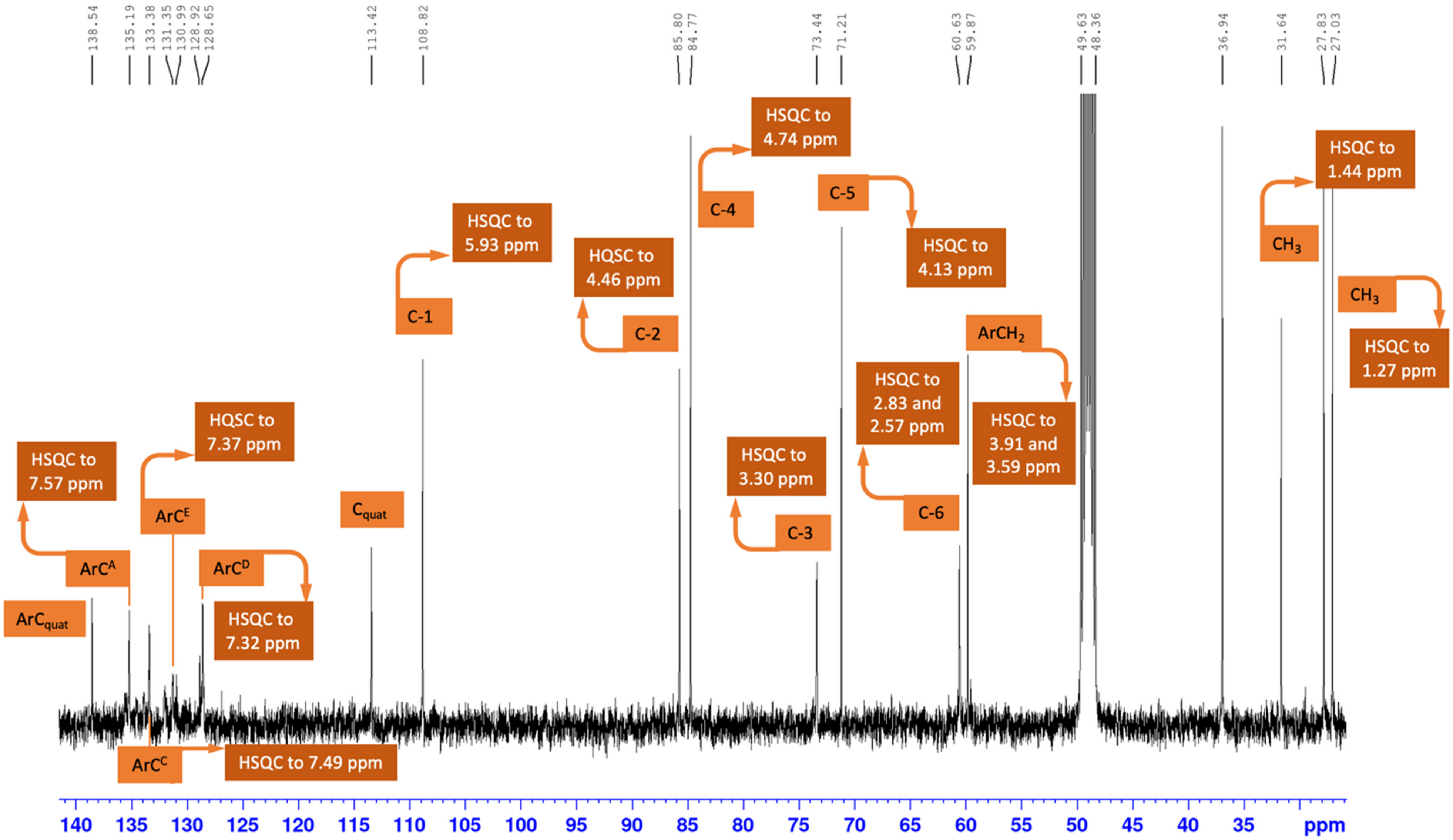

B

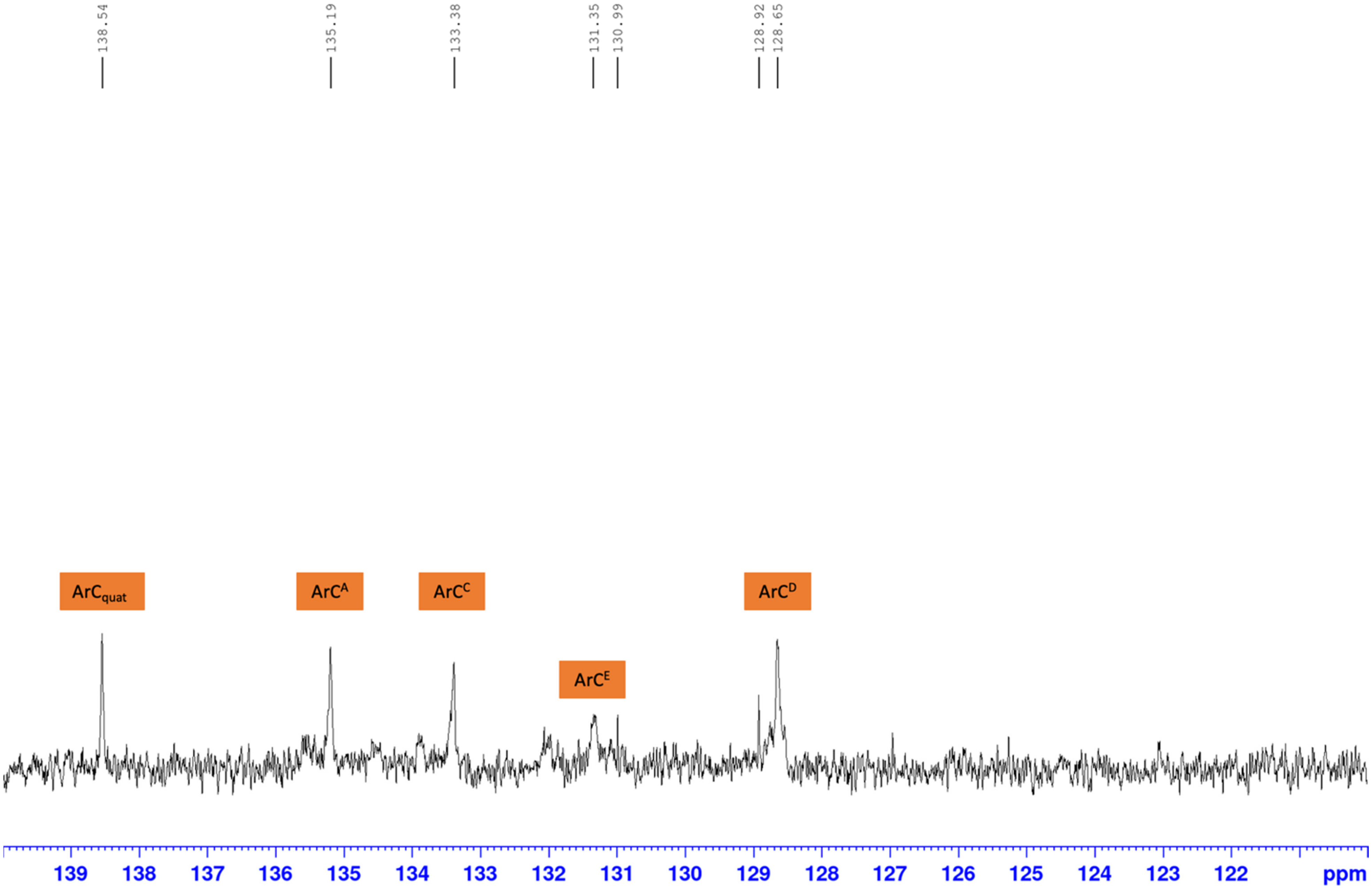

138.54
135.19
133.38
131.35
130.99
128.92
128.65
ArC$_{quat}$
ArC^{A}
ArC^{C}
ArC^{E}
ArC^{D}
139
138
137
136
135
134
133
132
131
130
129
128
127
126
125
124
123
122
ppm

Figure S16. ^{1}H- (400 MHz) and COSY spectra of the minor species for *N*-(3-methylphenyl boronic acid)-3,6-dideoxy-3,6-imino-1,2-*O*-isopropylidene-α-D-gulofuranose **meta 5** in MeOD.

^{1}H-NMR

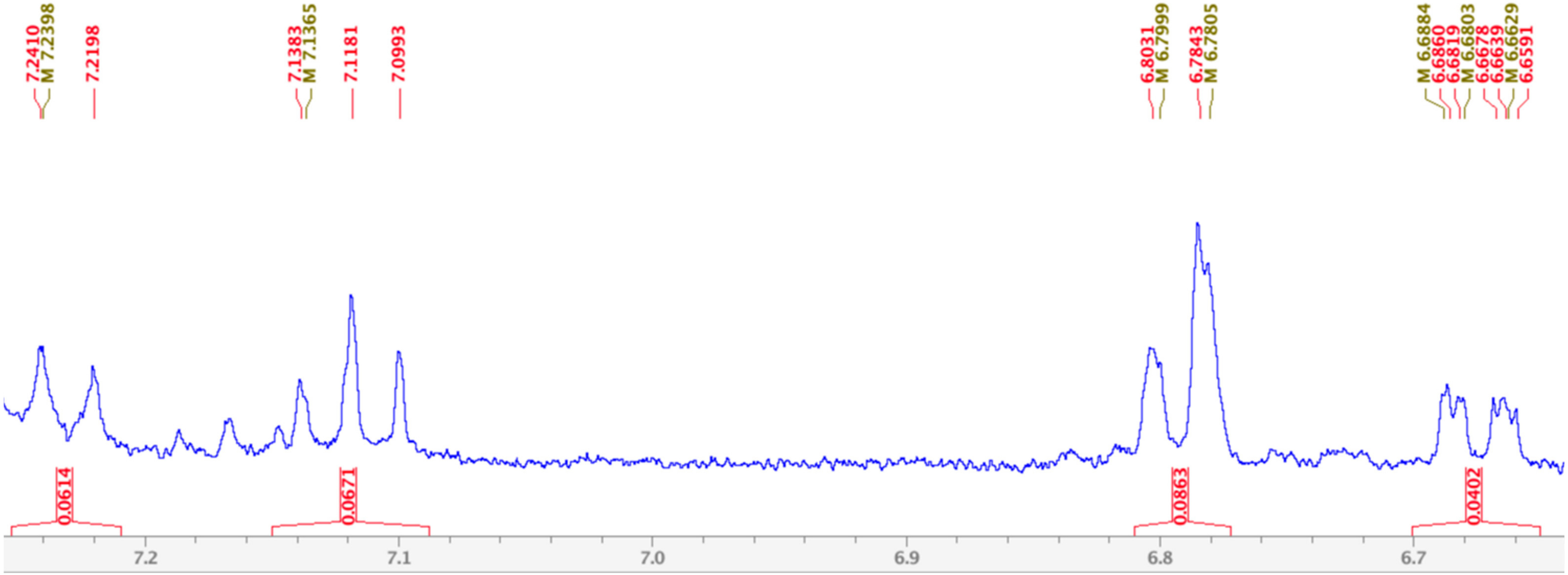

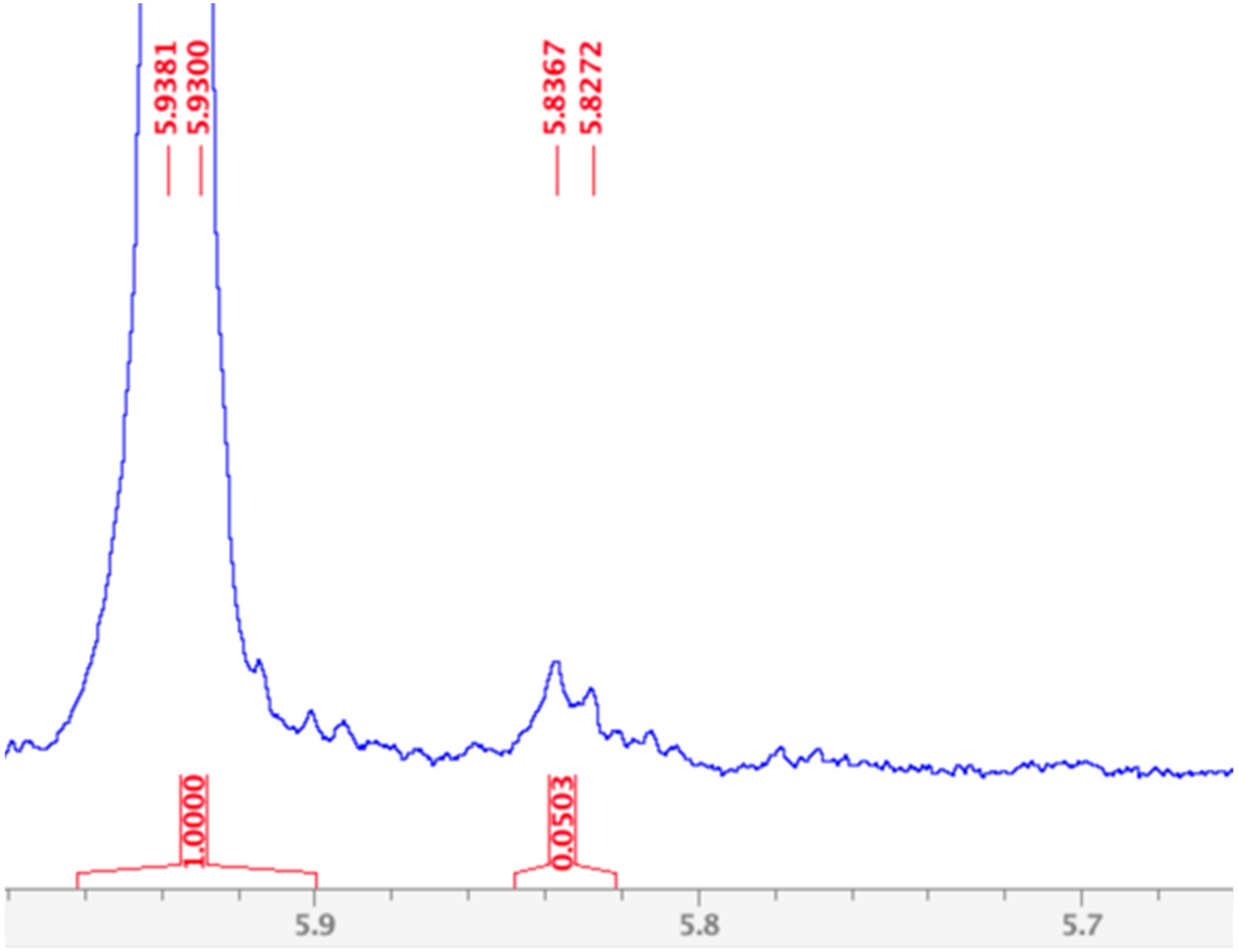
5.9381
5.9300
5.8367
5.8272
1.0000
0.0503
5.9
5.8
5.7

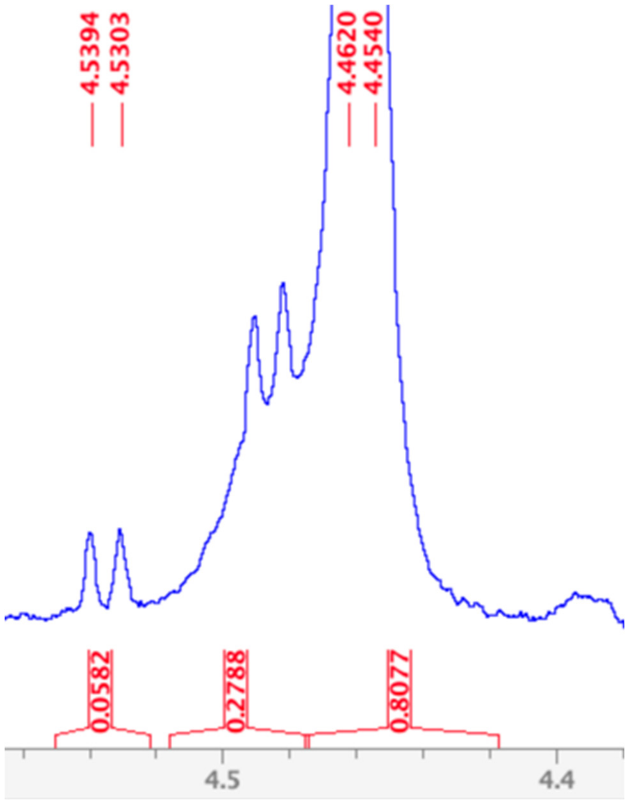
4.5394
4.5303
4.4620
4.4540
0.0582
0.2788
0.8077
4.5
4.4

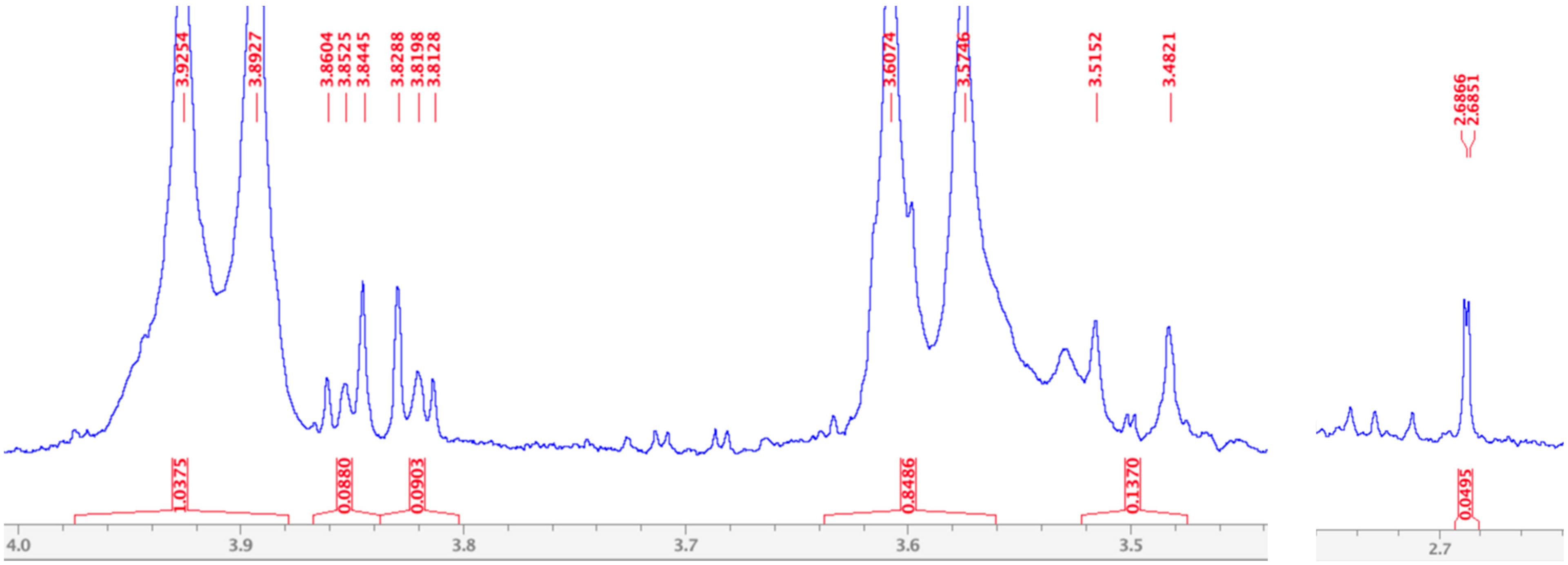
3.9254
3.8927
3.8604
3.8525
3.8445
3.8288
3.8198
3.8128
3.6074
3.5746
3.5152
3.4821
2.6866
2.6851
1.0375
0.0880
0.0903
0.8486
0.1370
0.0495
4.0
3.9
3.8
3.7
3.6
3.5
2.7

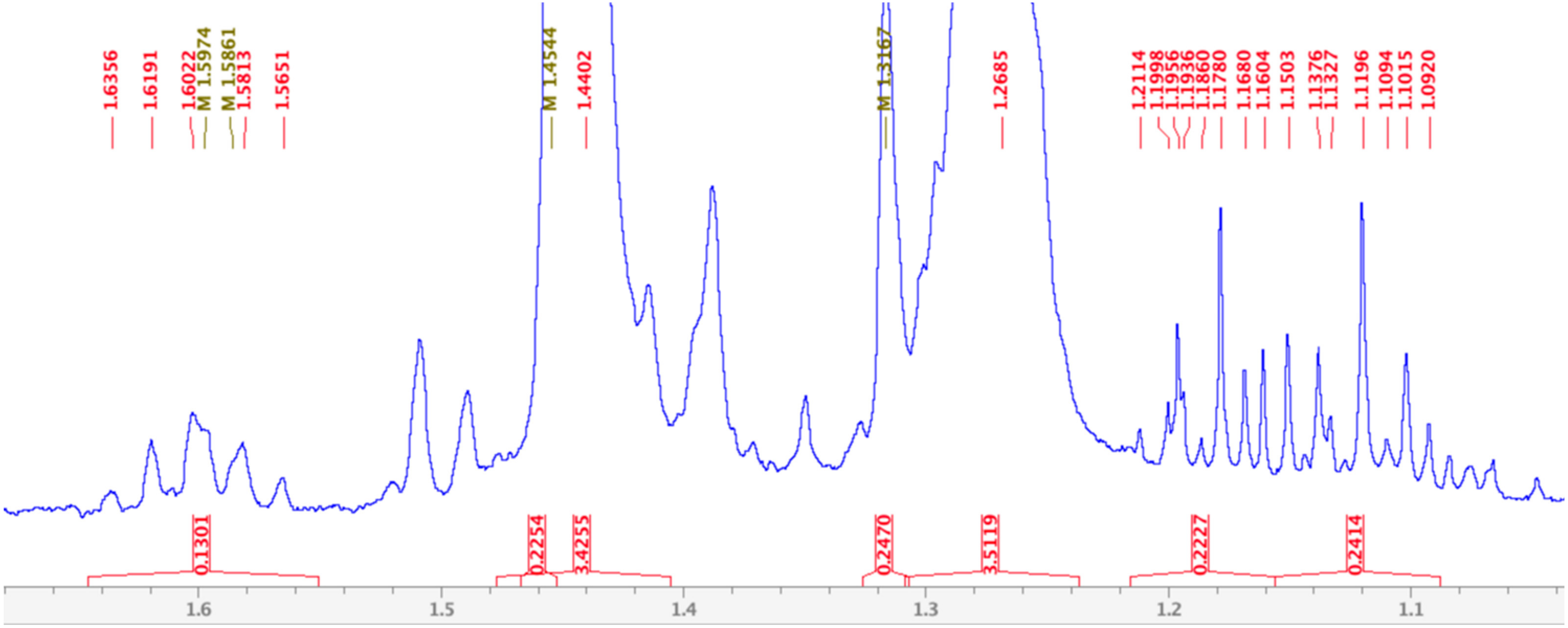
1.6356
1.6191
1.6022
M 1.5974
M 1.5861
1.5813
1.5651
M 1.4544
1.4402
M 1.3167
1.2685
1.2114
1.1998
1.1956
1.1936
1.1860
1.1780
1.1680
1.1604
1.1503
1.1376
1.1327
1.1196
1.1094
1.1015
1.0920
0.1301
0.2254
3.4255
0.2470
3.5119
0.2227
0.2414
1.6
1.5
1.4
1.3
1.2
1.1

COSY

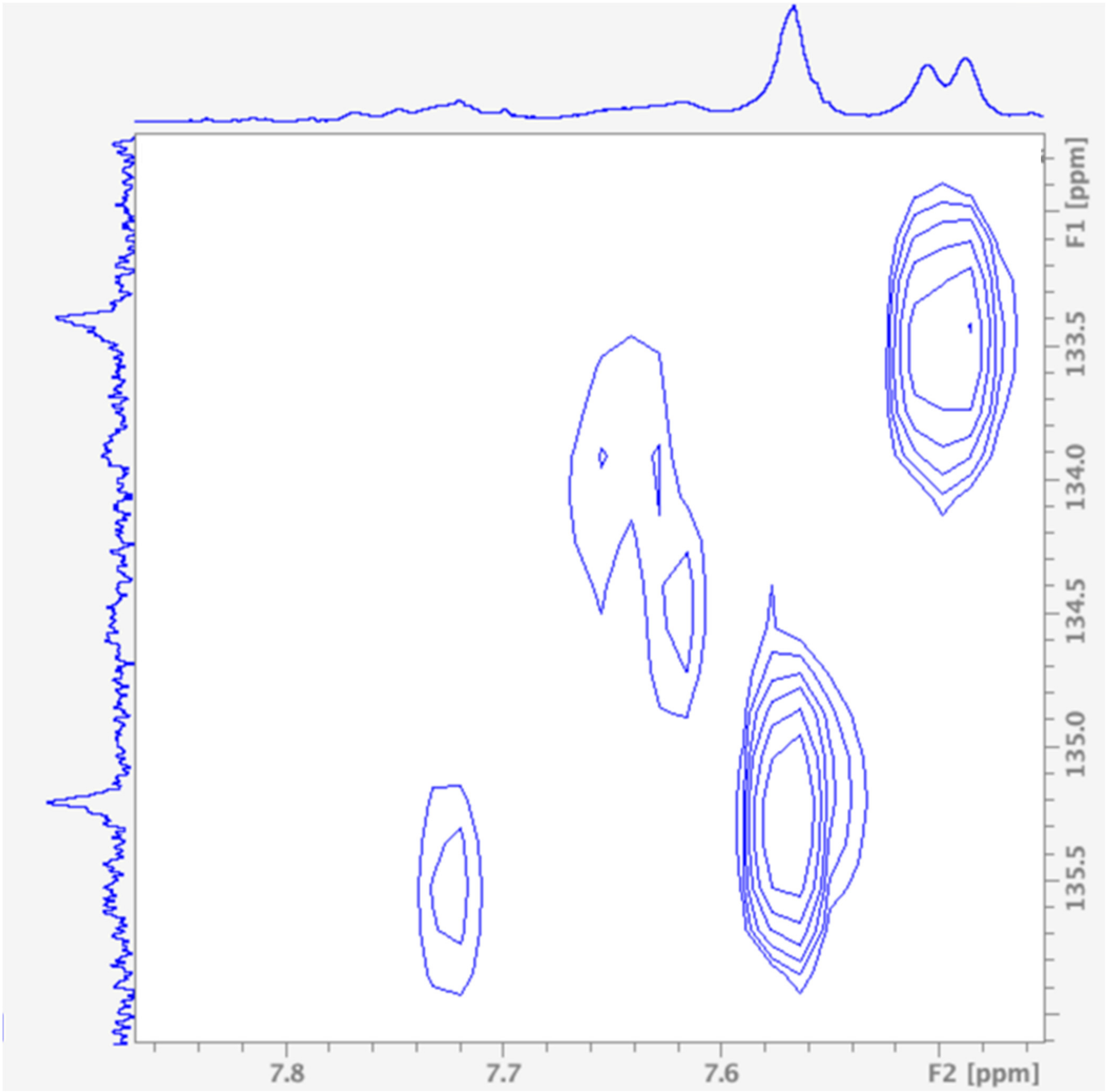

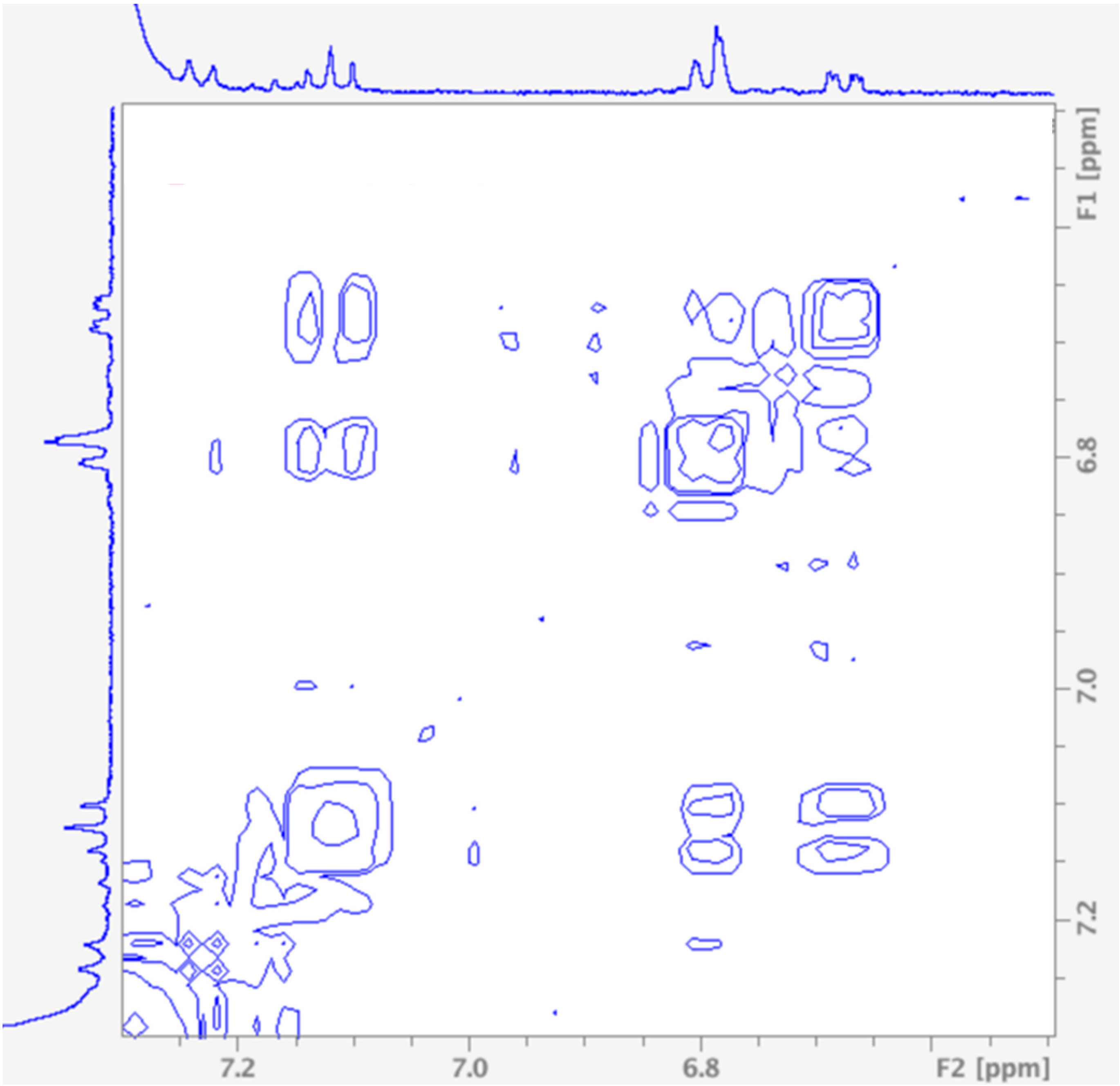

F1 [ppm]
6.8
7.0
7.2
7.2
7.0
6.8
F2 [ppm]

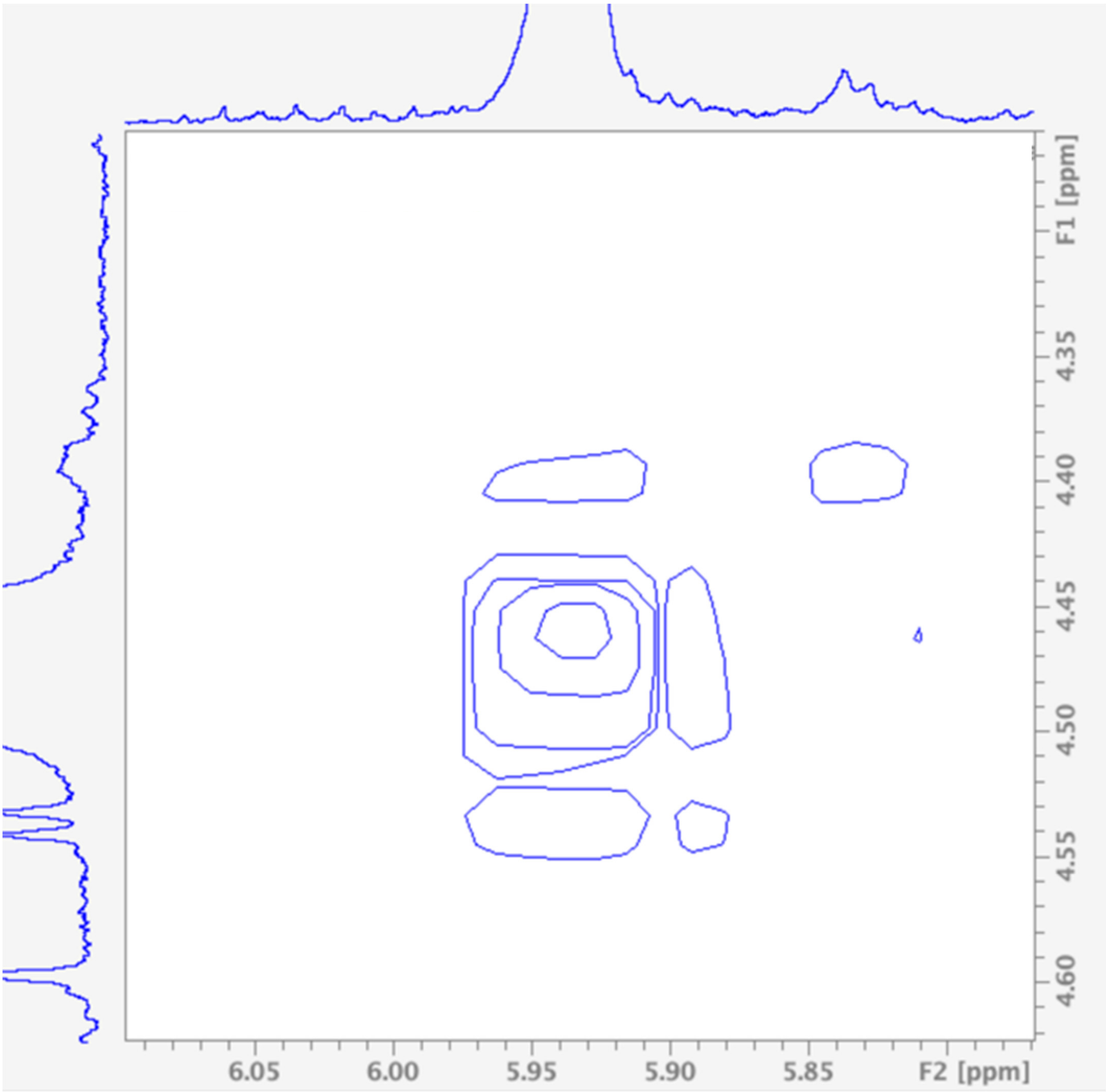

F1 [ppm]
4.35
4.40
4.45
4.50
4.55
4.60
6.05
6.00
5.95
5.90
5.85
F2 [ppm]

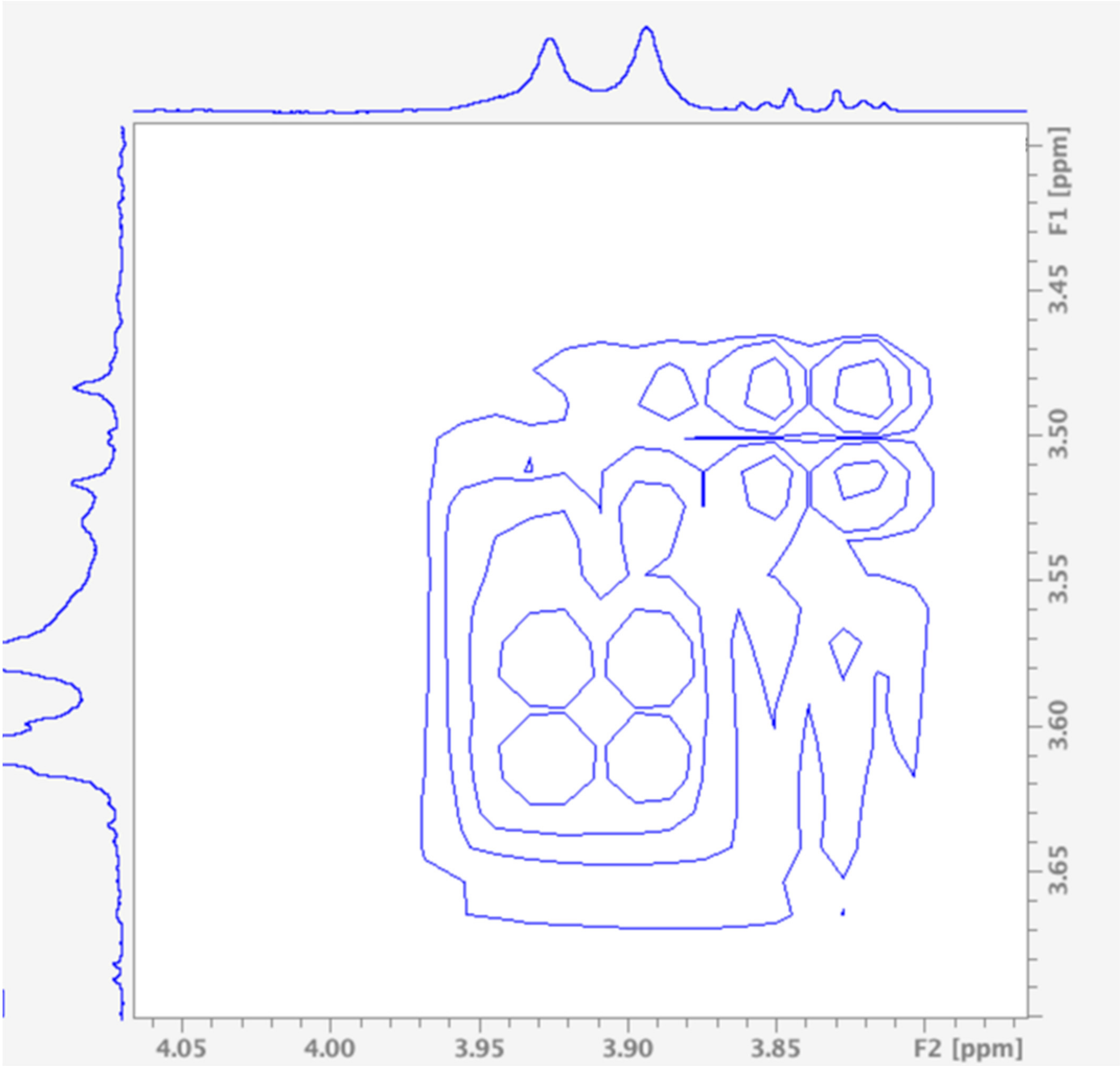
4.05
4.00
3.95
3.90
3.85
F2 [ppm]
F1 [ppm]
3.45
3.50
3.55
3.60
3.65

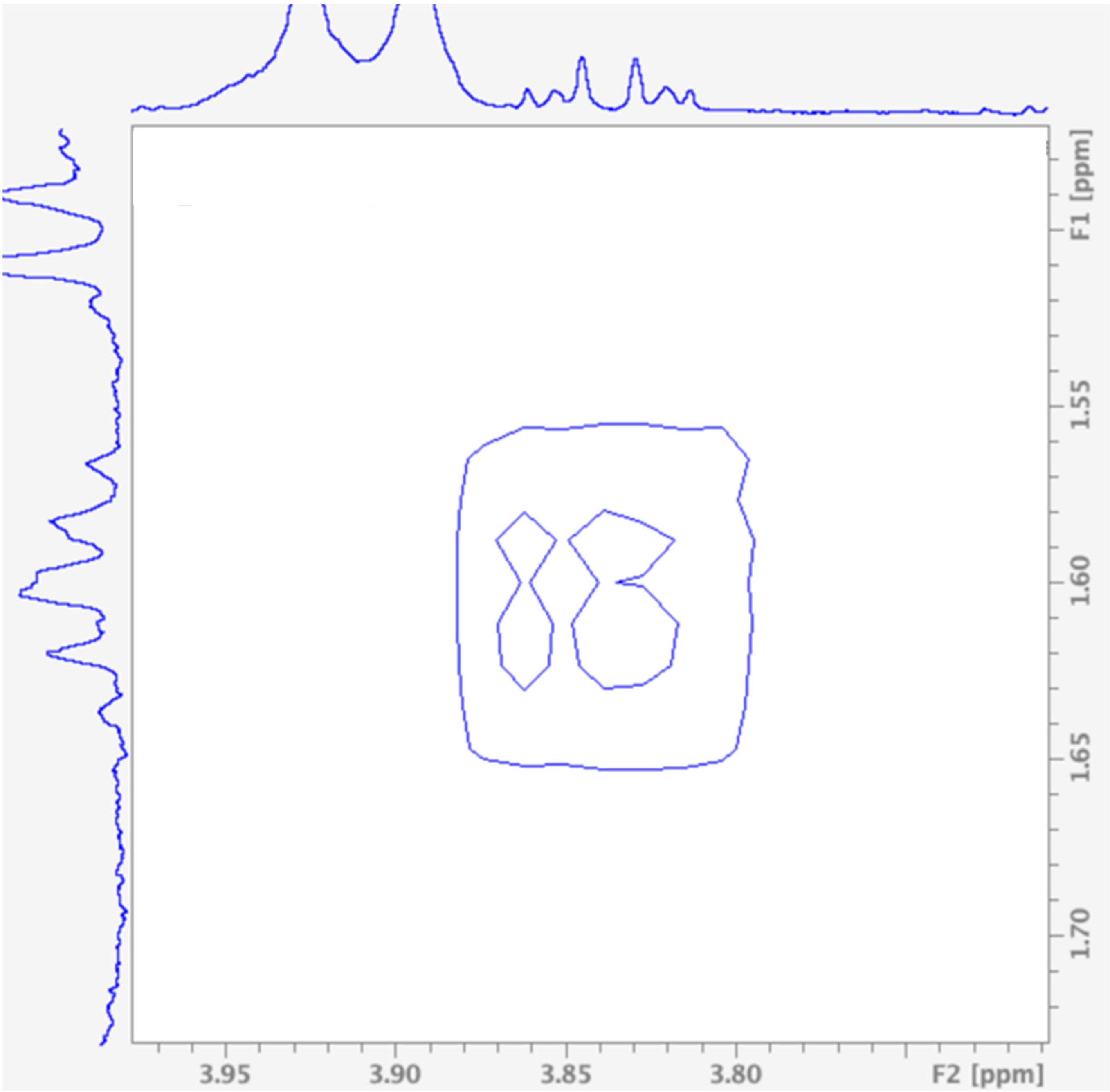
F1 [ppm]
1.55
1.60
1.65
1.70
3.95
3.90
3.85
3.80
F2 [ppm]

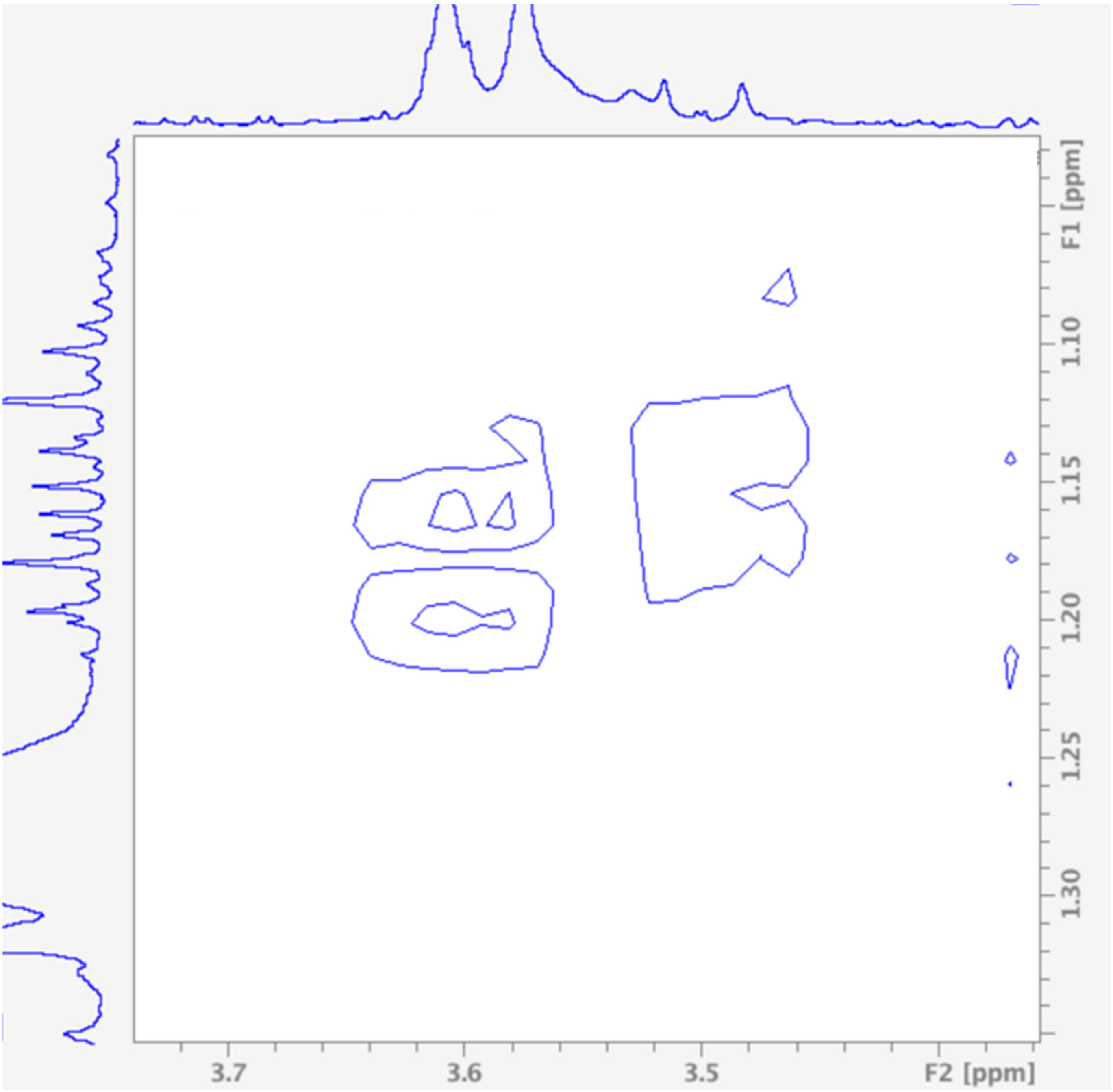

F1 [ppm]
1.10
1.15
1.20
1.25
1.30
3.7
3.6
3.5
F2 [ppm]

Figure S17. ^{1}H- (400 MHz), ^{13}C-NMR (100 MHz), DEPT, HSQC and HMBC spectra of *N*-(3-methylphenyl boronic acid)-3,6-dideoxy-3,6-imino-1,2-*O*-isopropylidene-α-D-gulofuranose **meta 5** in MeOD.

Figure S13. ^{1}H- (400 MHz), ^{13}C-NMR (100 MHz), ^{11}B-NMR (128 MHz), COSY and HSQC spectra of *N*-(3-methylphenyl boronic acid)-3,6-dideoxy-3,6-imino-1,2-*O*-isopropylidene-α-D-gulofuranose **meta 5** in MeOD.

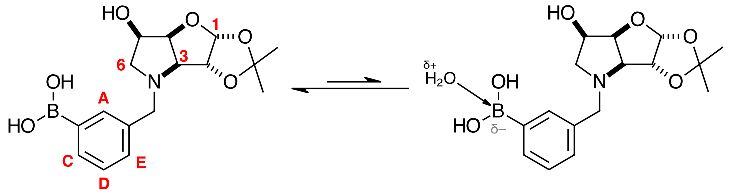

^{1}H-NMR

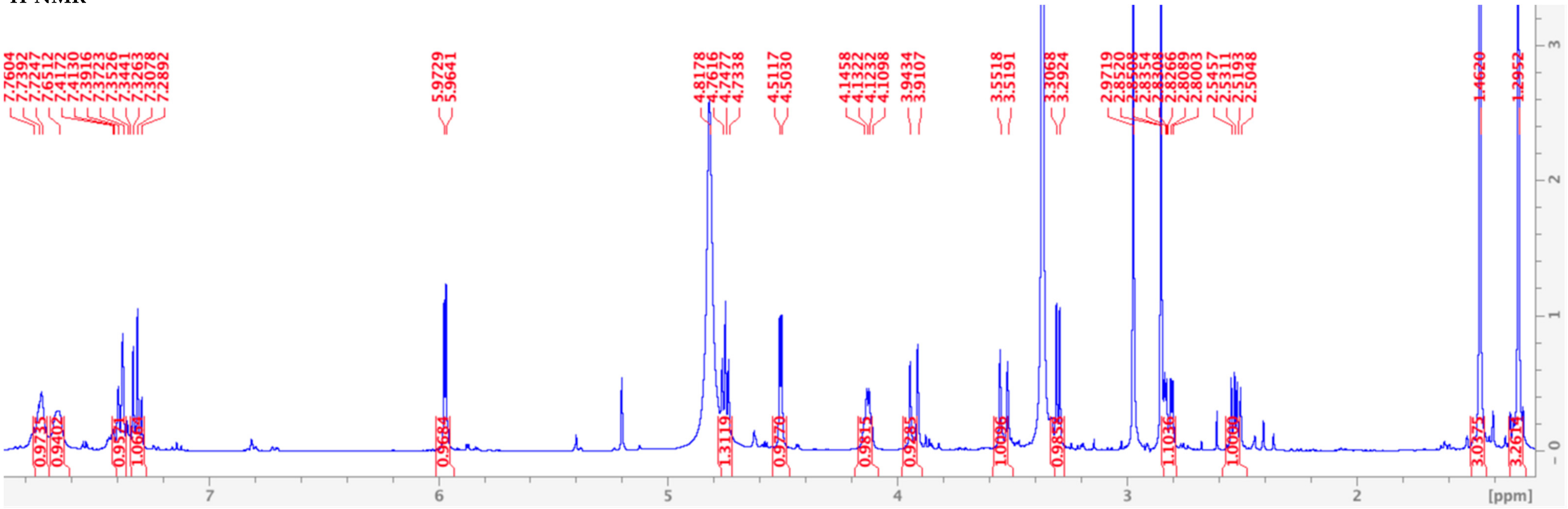

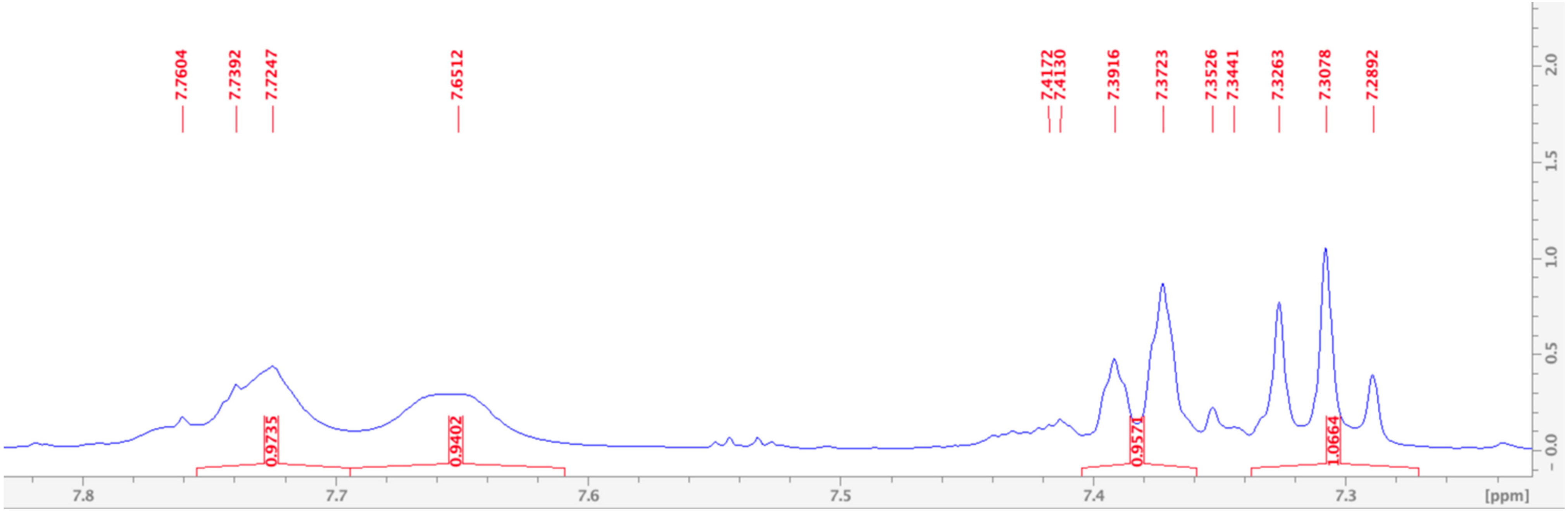
7.7604
7.7392
7.7247
7.6512
7.4172
7.4130
7.3916
7.3723
7.3526
7.3441
7.3263
7.3078
7.2892
0.9735
0.9402
0.9571
1.0664
7.8
7.7
7.6
7.5
7.4
7.3
[ppm]
2.0
1.5
1.0
0.5
0.0

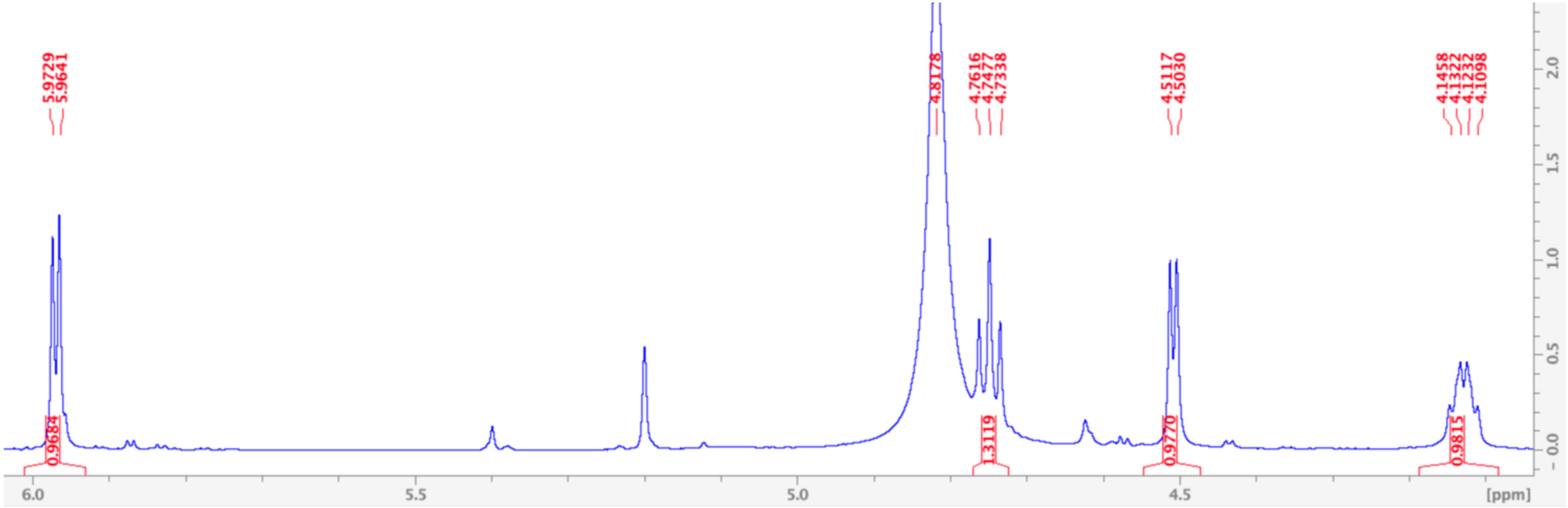
5.9729
5.9641
4.8178
4.7616
4.7477
4.7338
4.5117
4.5030
4.1458
4.1322
4.1232
4.1098
0.9684
1.3119
0.9770
0.9815
6.0
5.5
5.0
4.5
[ppm]
2.0
1.5
1.0
0.5
0.0

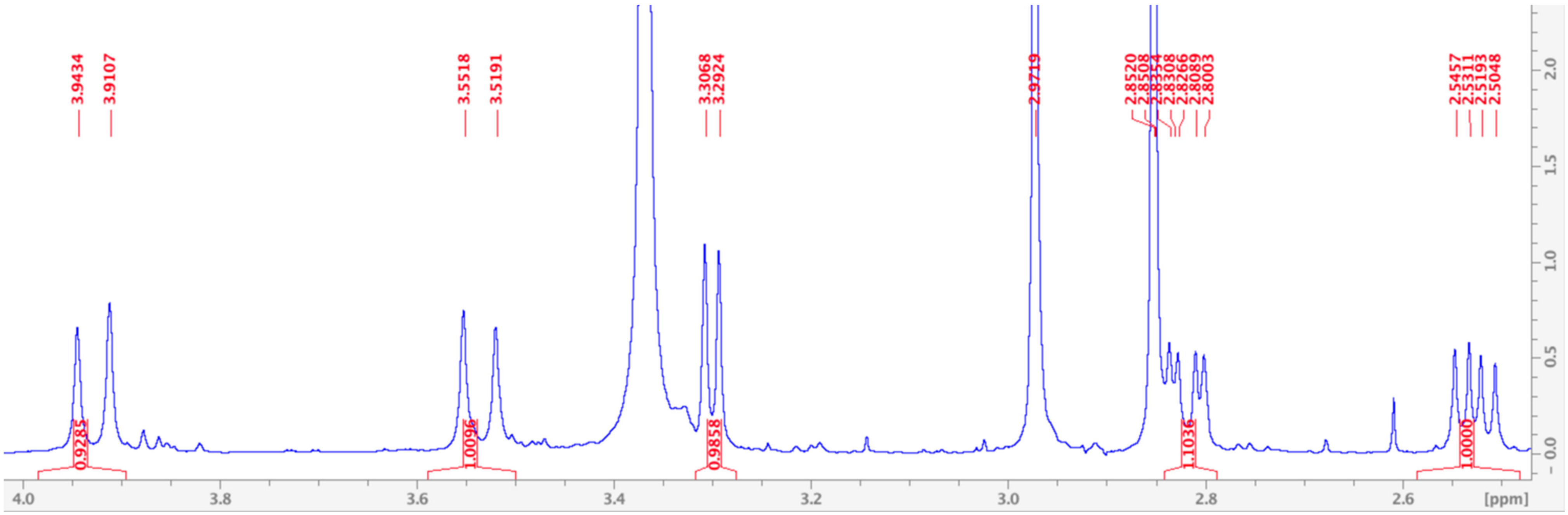
3.9434
3.9107
3.5518
3.5191
3.3068
3.2924
2.9719
2.8520
2.8508
2.8354
2.8308
2.8266
2.8089
2.8003
2.5457
2.5311
2.5193
2.5048
0.9285
1.0096
0.9858
1.1036
1.0000
[ppm]

^{13}C-NMR

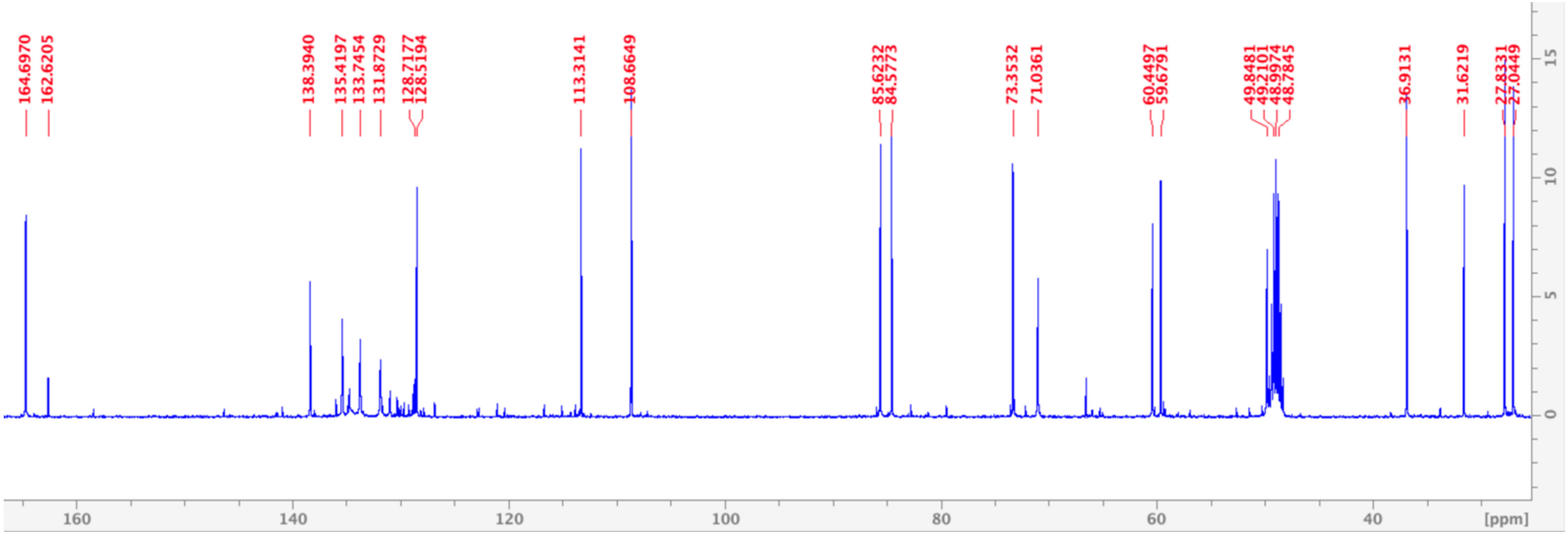
164.6970
162.6205
138.3940
135.4197
133.7454
131.8729
128.7177
128.5194
113.3141
108.6649
85.6232
84.5773
73.3532
71.0361
60.4497
59.6791
49.8481
49.2101
48.9974
48.7845
36.9131
31.6219
27.8331
27.0449
160
140
120
100
80
60
40
[ppm]
15
10
5
0

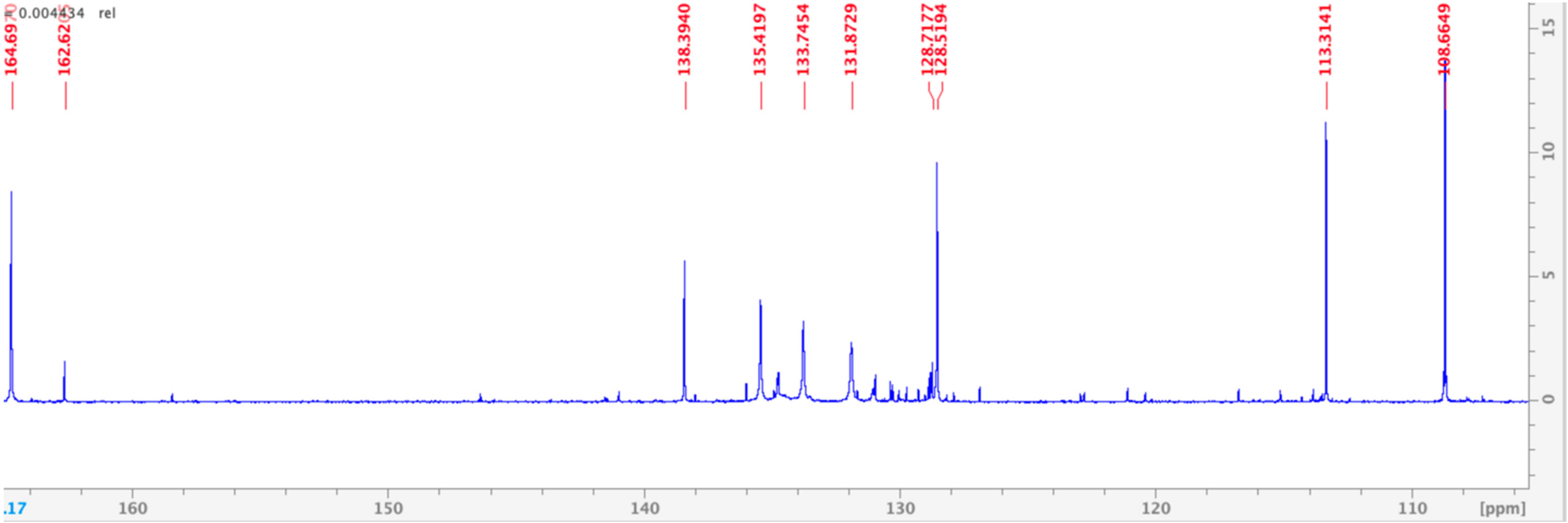

= 0.004434 rel
164.6970
162.6205
138.3940
135.4197
133.7454
131.8729
128.7177
128.5194
113.3141
108.6649
.17
160
150
140
130
120
110
[ppm]
15
10
5
0

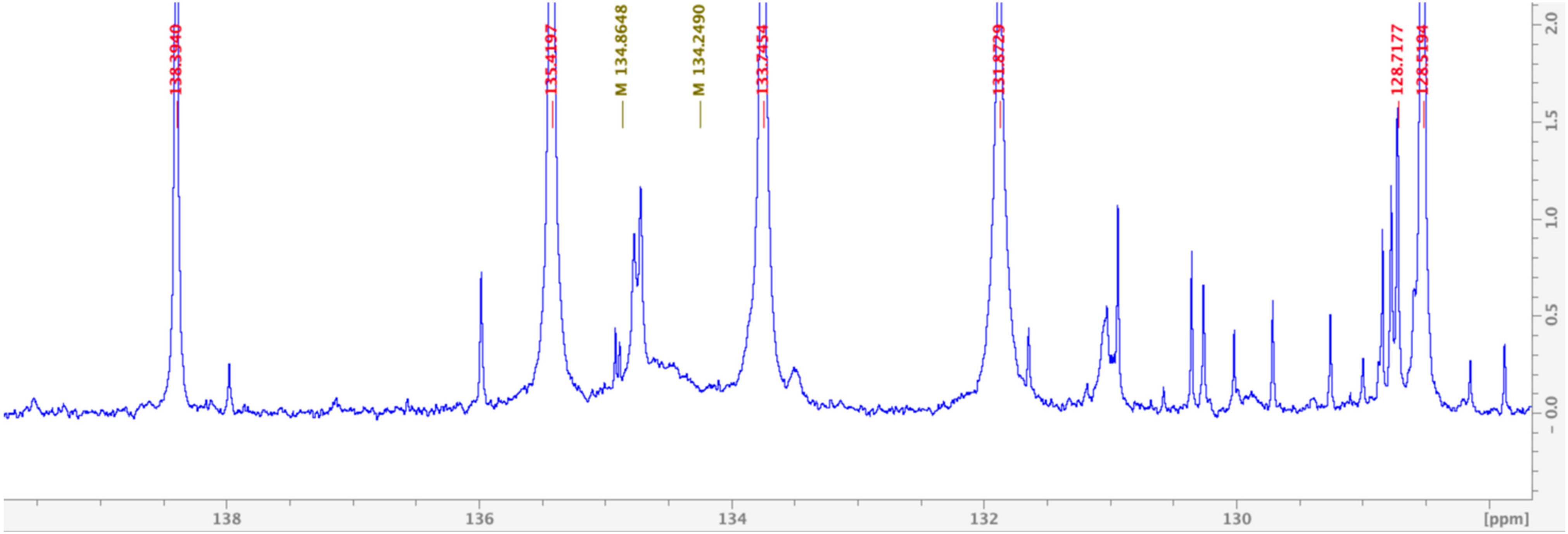
138.3940
135.4197
M 134.8648
M 134.2490
133.7454
131.8729
128.7177
128.5194
138
136
134
132
130
[ppm]
2.0
1.5
1.0
0.5
0.0

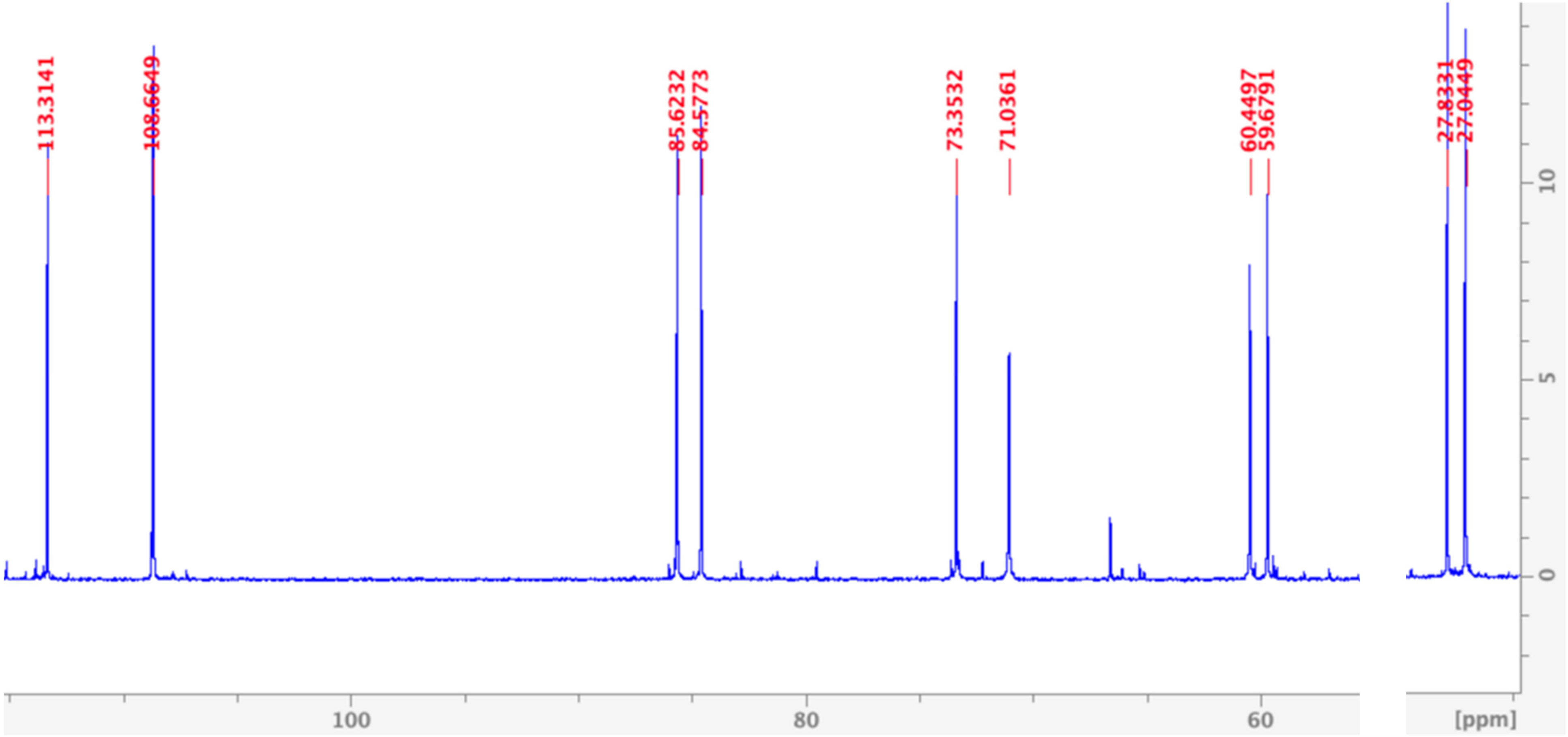
113.3141
108.6649
85.6232
84.5773
73.3532
71.0361
60.4497
59.6791
27.8331
27.0449
100
80
60
[ppm]
10
5
0

DEPT

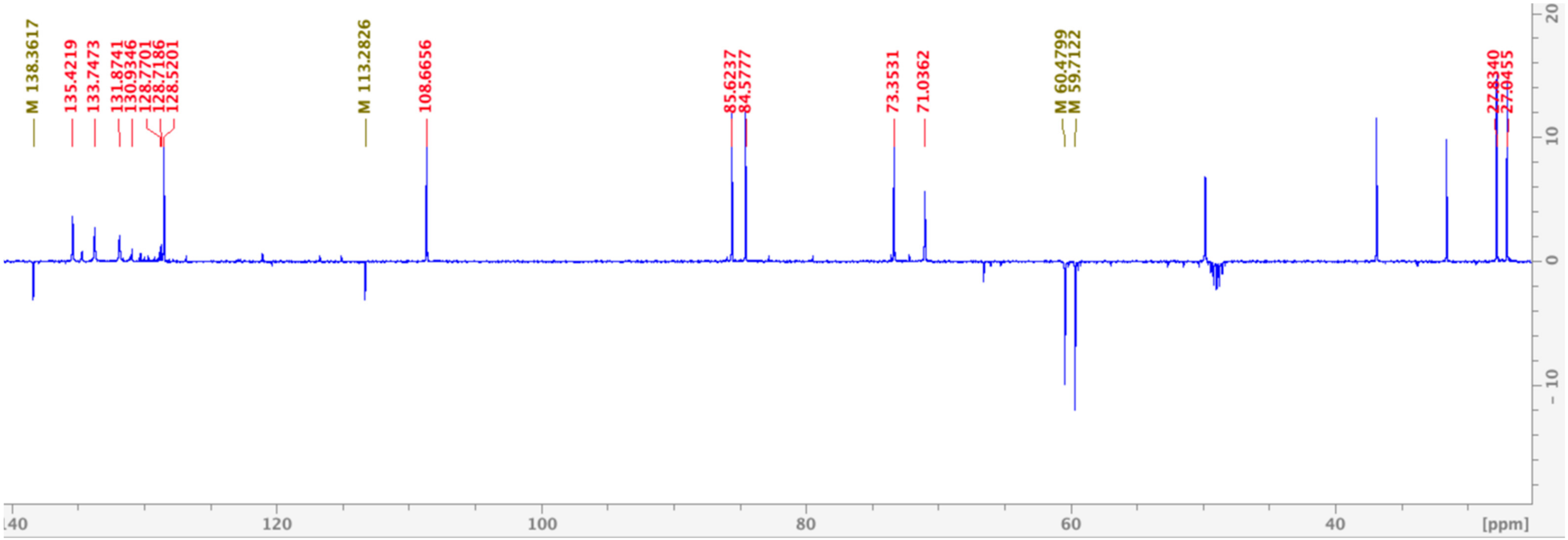
M 138.3617
135.4219
133.7473
131.8741
130.9346
128.7701
128.7186
128.5201
M 113.2826
108.6656
85.6237
84.5777
73.3531
71.0362
M 60.4799
M 59.7122
27.8340
27.0455
140
120
100
80
60
40
[ppm]
20
10
0
- 10

HSQC

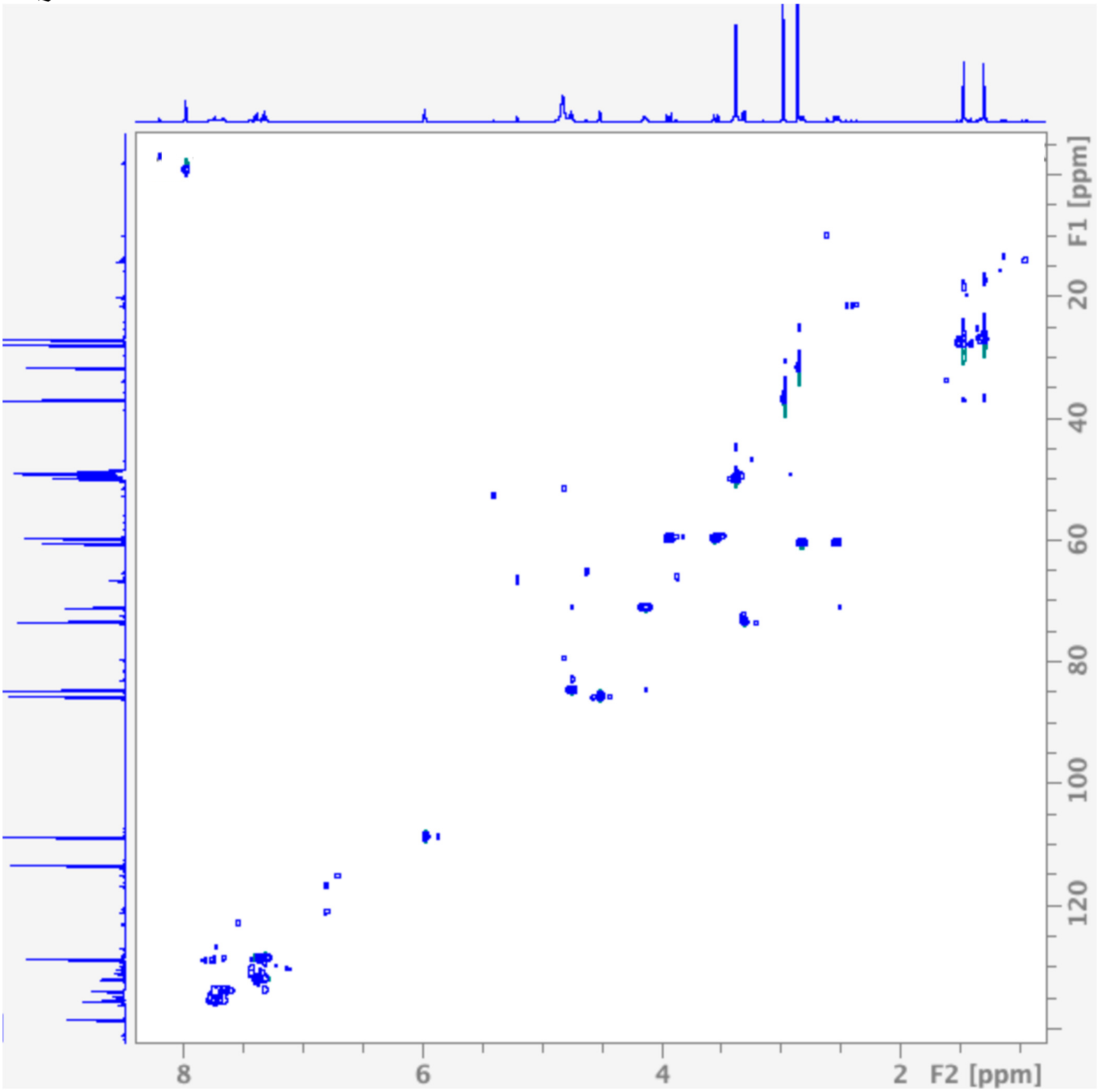

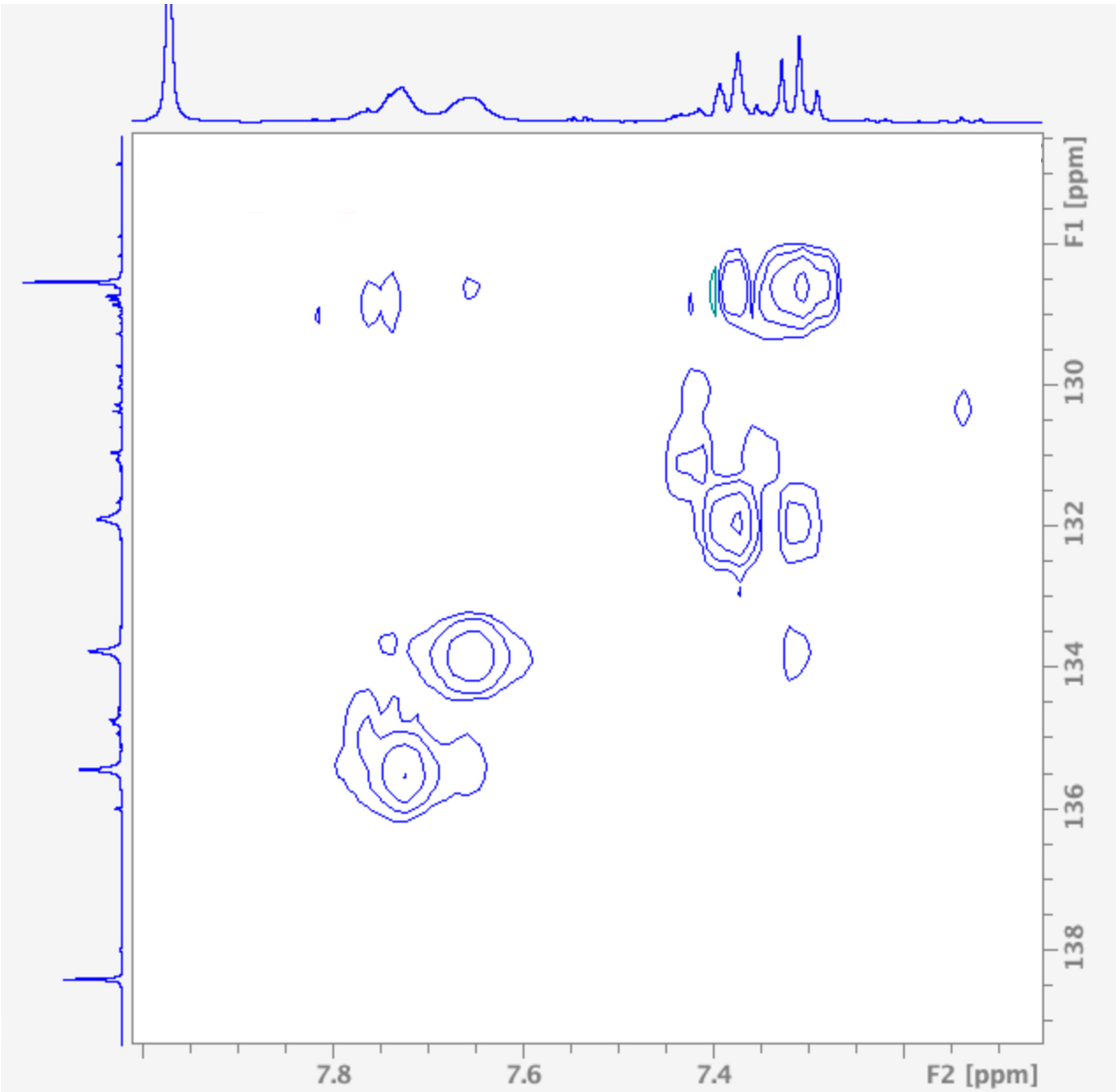
F1 [ppm]
130
132
134
136
138
7.8
7.6
7.4
F2 [ppm]

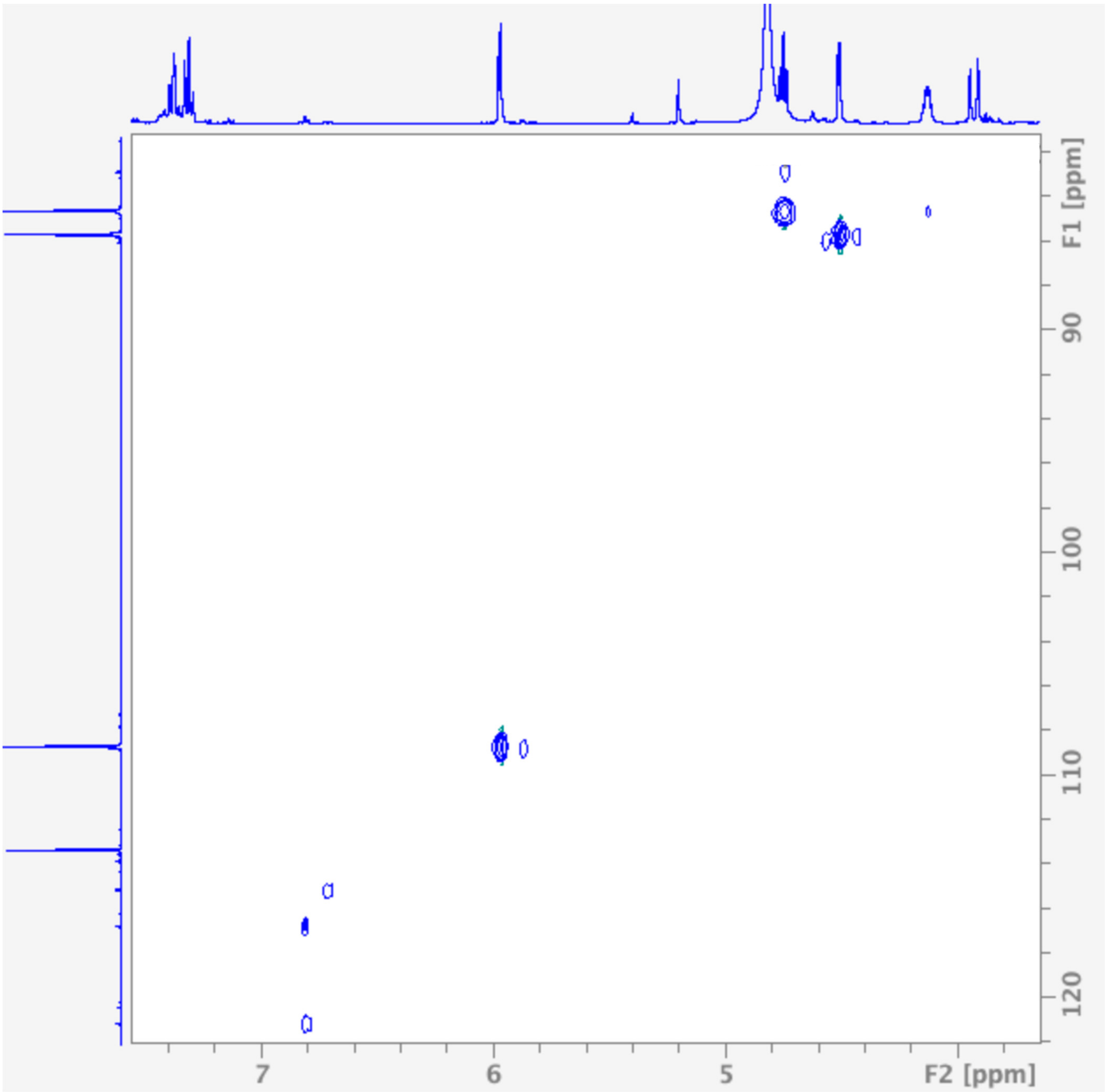

F1 [ppm]
90
100
110
120
7
6
5
F2 [ppm]

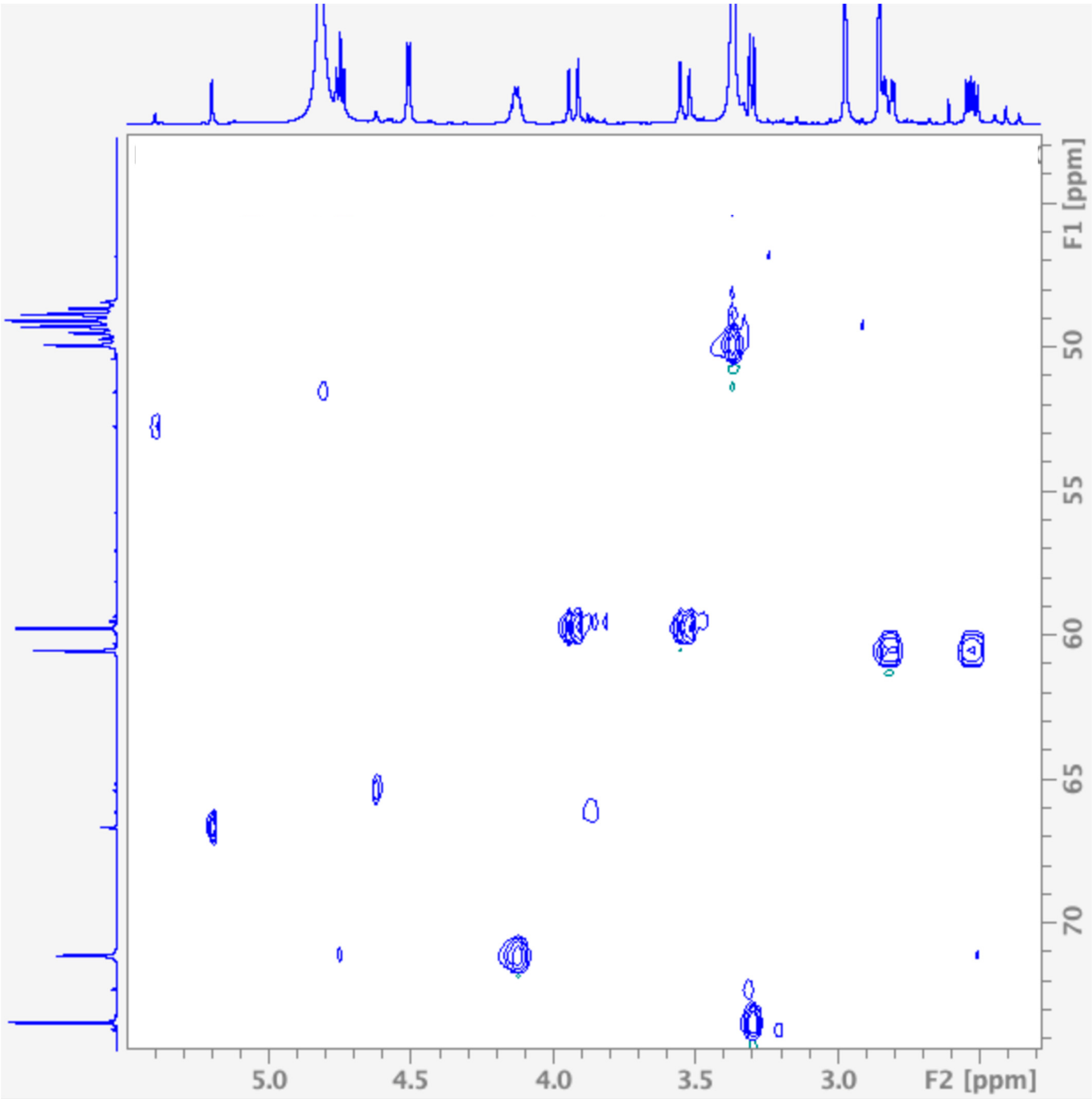

F1 [ppm]
50
55
60
65
70
5.0
4.5
4.0
3.5
3.0
F2 [ppm]

HMBC

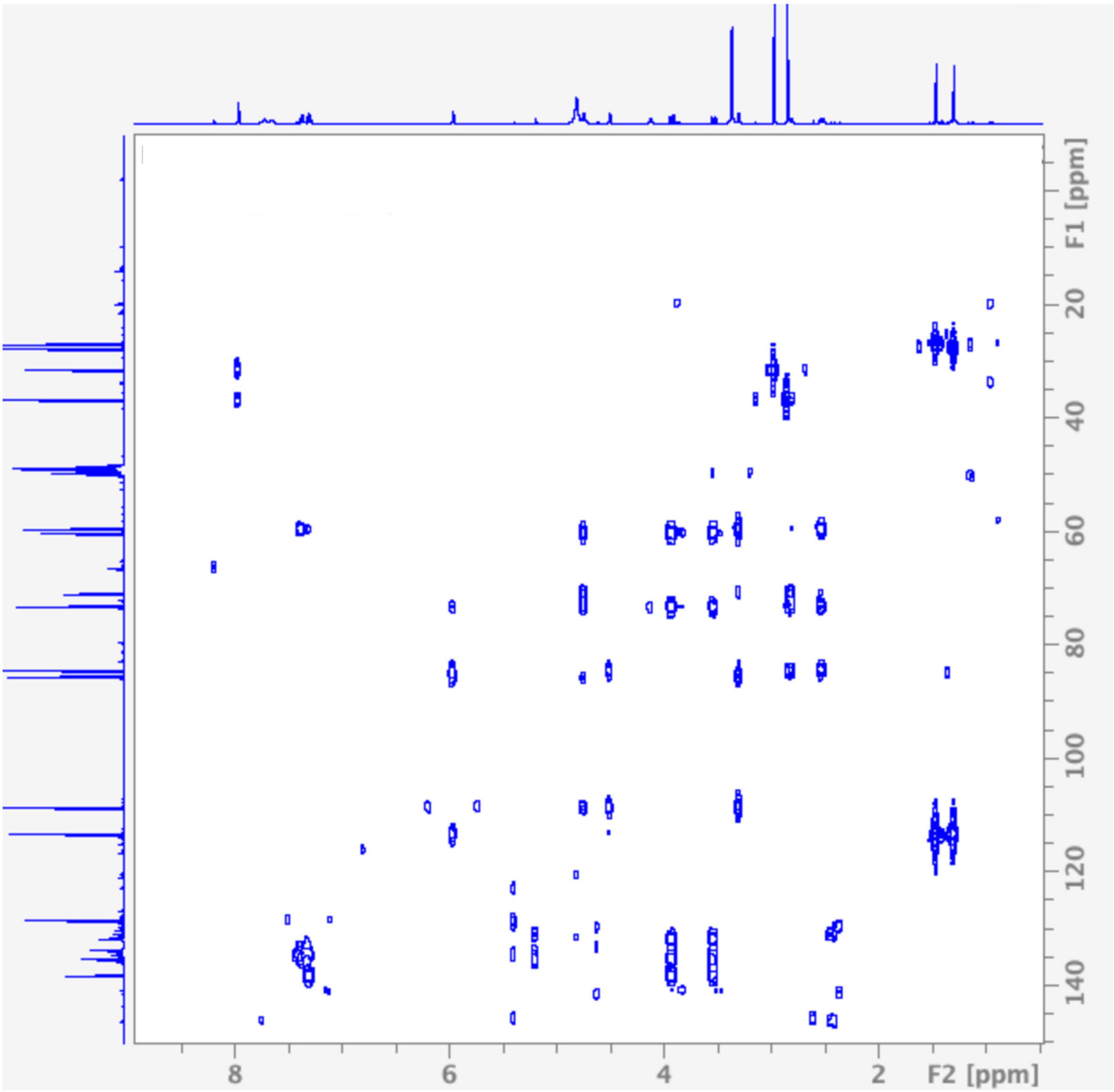

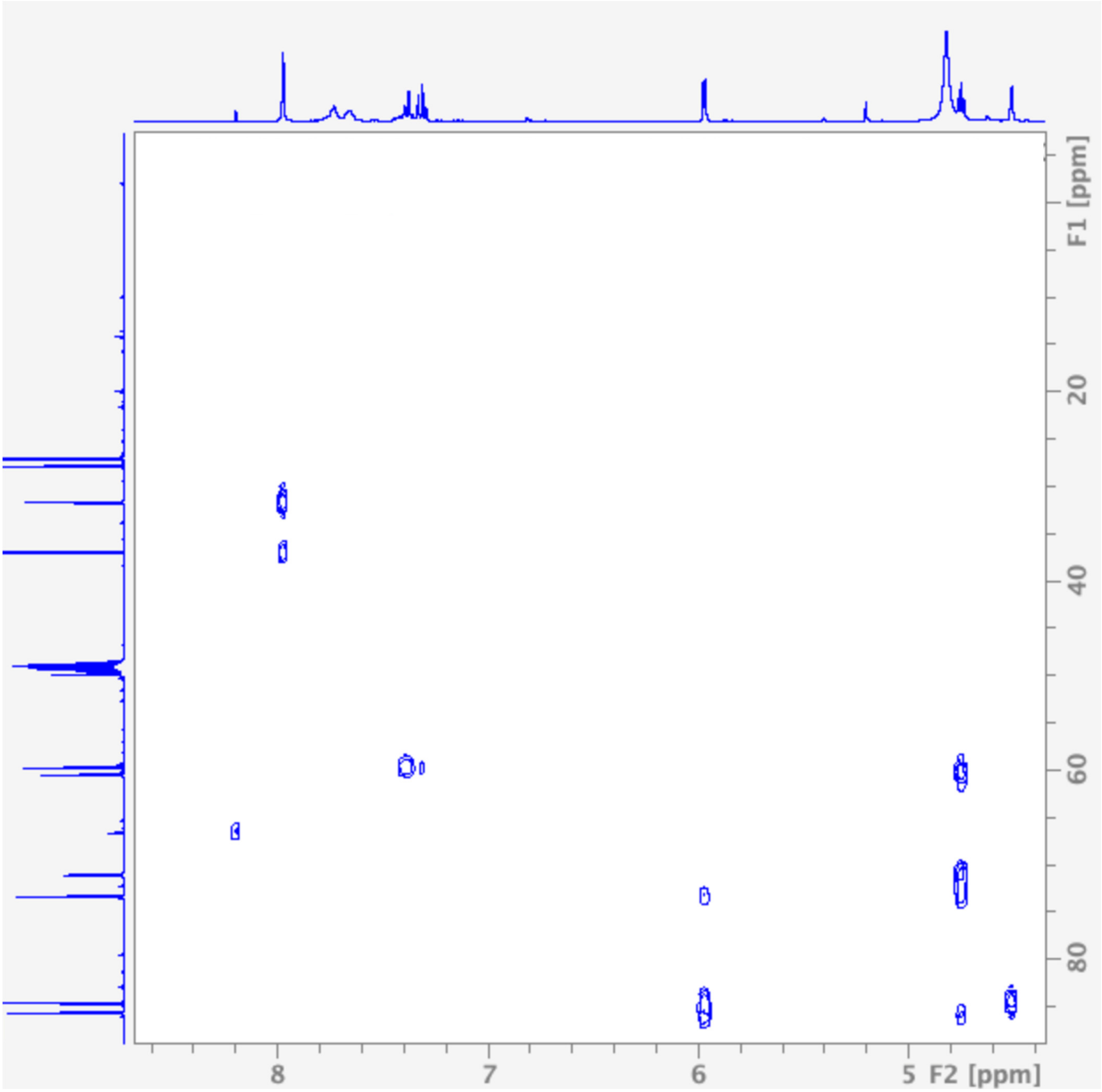
F1 [ppm]
20
40
60
80
8
7
6
5
F2 [ppm]

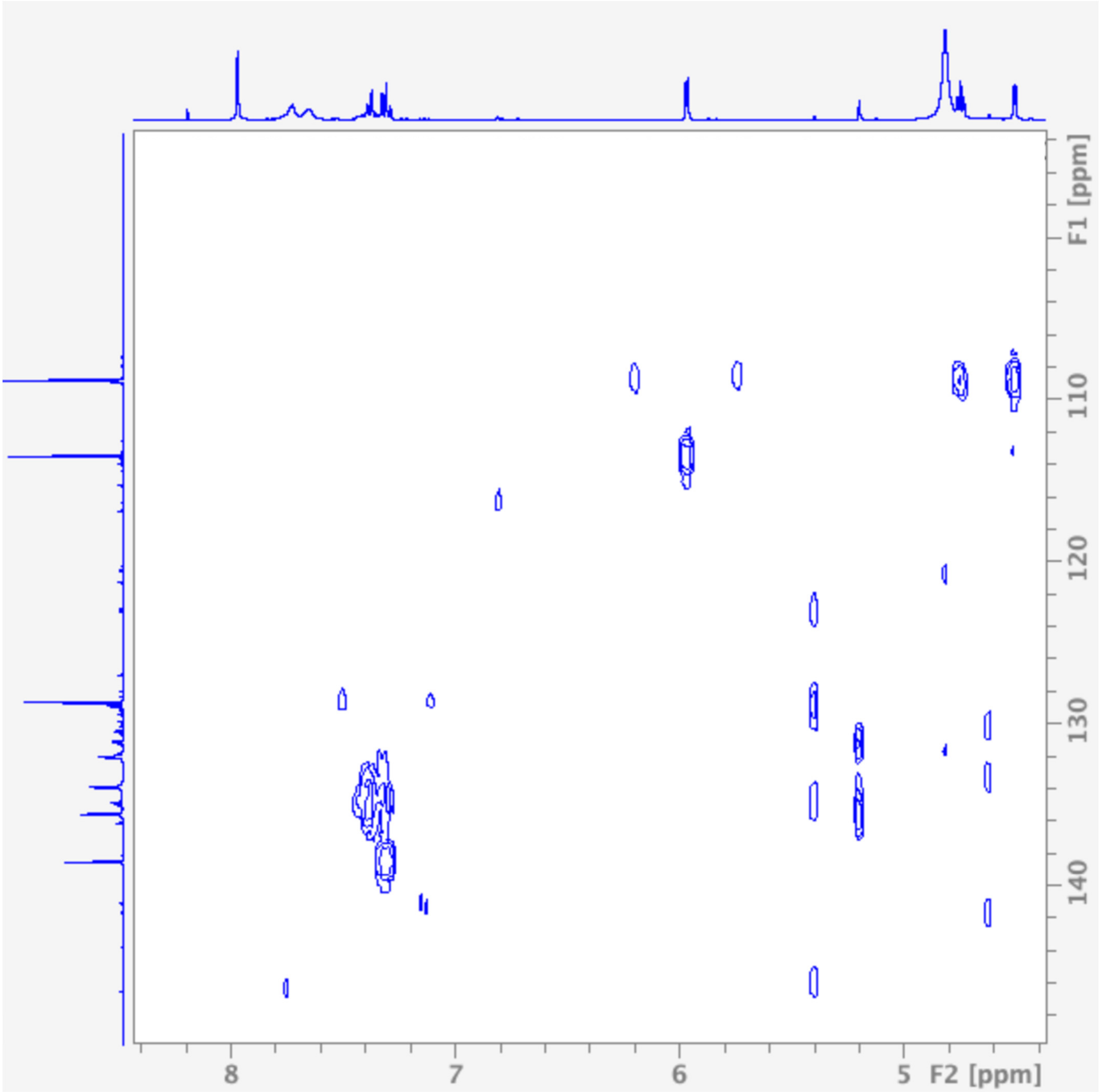
F1 [ppm]
110
120
130
140
8
7
6
5
F2 [ppm]

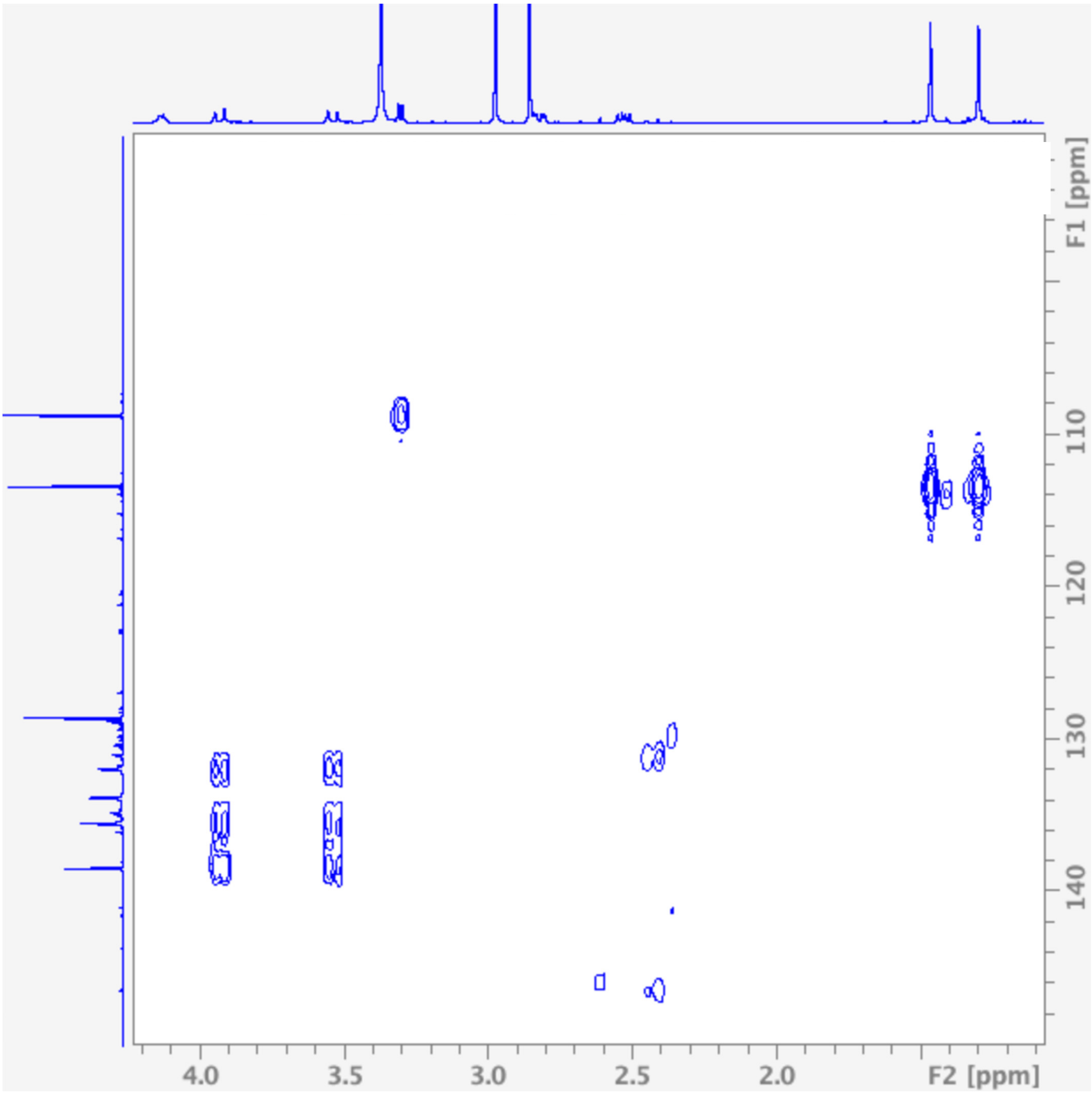

F1 [ppm]
110
120
130
140
4.0
3.5
3.0
2.5
2.0
F2 [ppm]

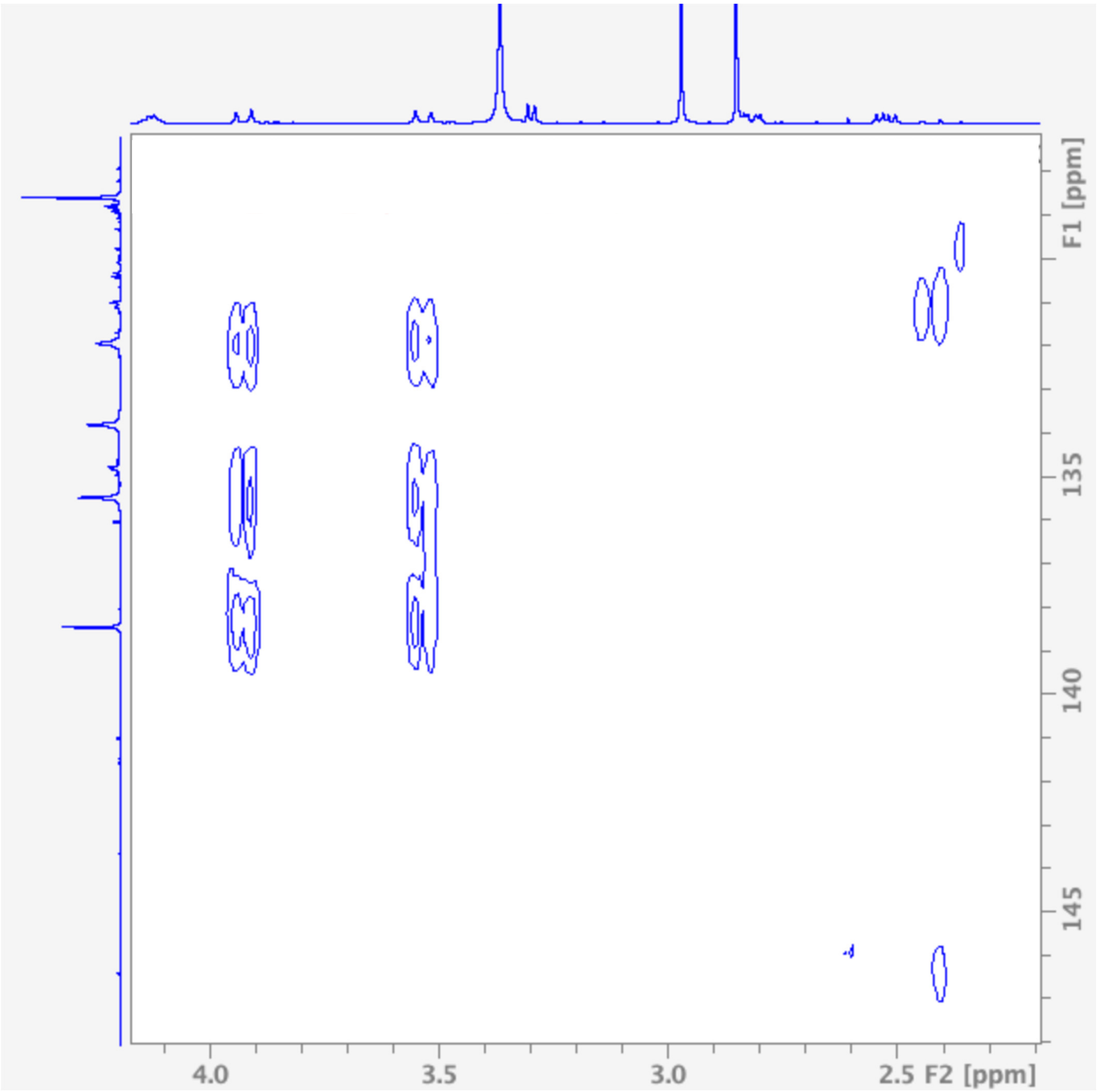
F1 [ppm]
135
140
145
4.0
3.5
3.0
2.5 F2 [ppm]

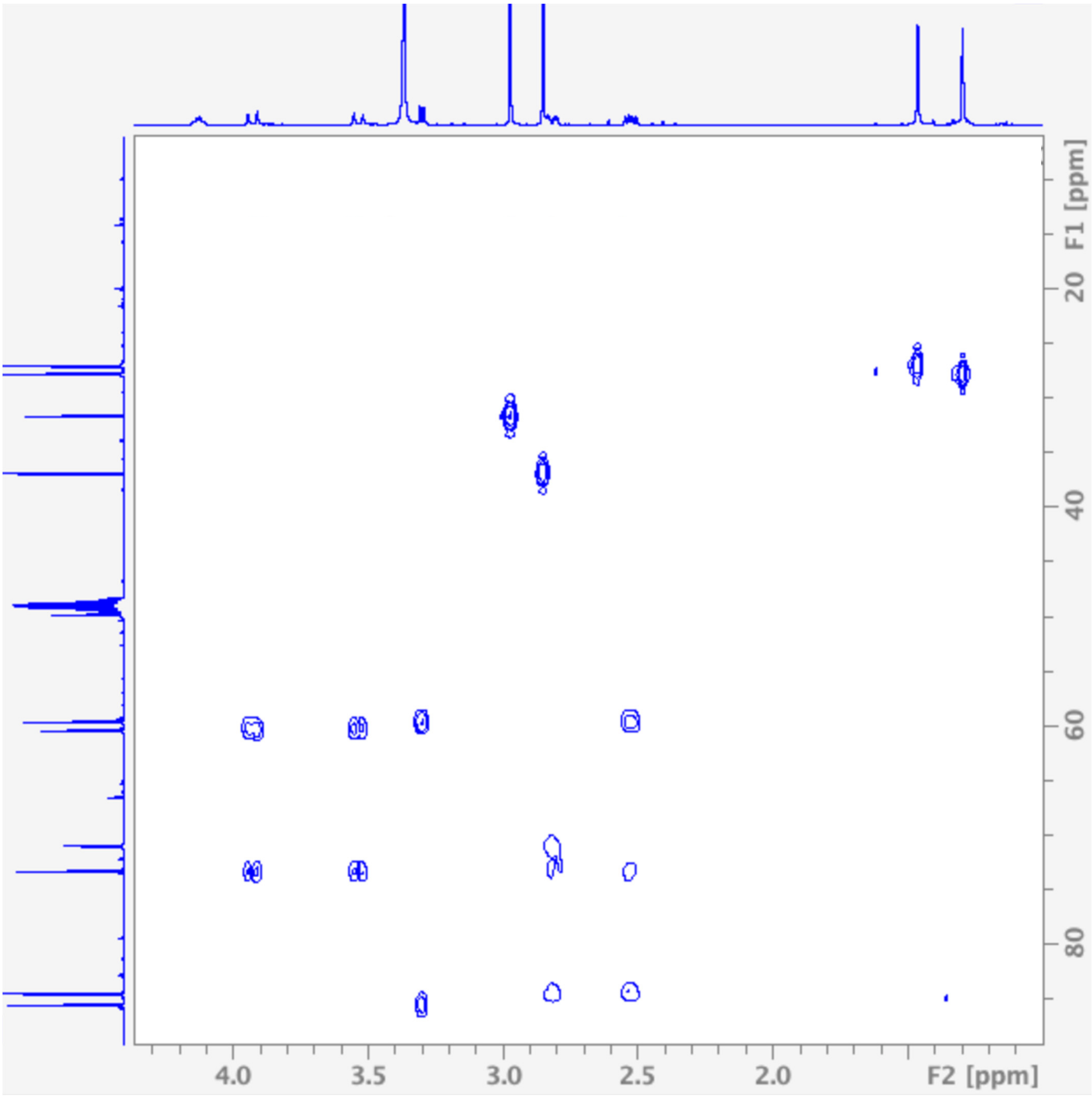

F1 [ppm]
20
40
60
80
4.0
3.5
3.0
2.5
2.0
F2 [ppm]

Figure S18. ^{1}H- (400 MHz, D_2O), ^{13}C-NMR (100 MHz), DEPT, ^{11}B-NMR (128 MHz), COSY and HSQC spectra of *N*-(2-methylphenyl boronic acid)-3,6-dideoxy-3,6-imino-1,2-*O*-isopropylidene-α-D-gulofuranose **ortho 5** in D_2O.

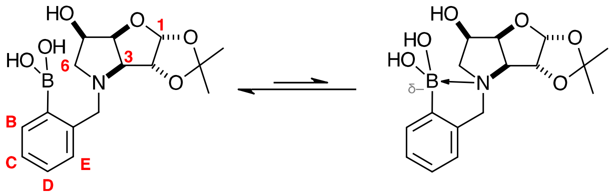

Ratio boronic acid species : boronate species, ~4.2 : 1.0 ratio.

Boronic acid species. **δ_H:** 7.75 (1H, $J_{HB,HD}$ 1.6 Hz, partially obscured, ArH^B), 7.77-7.73 (1H, m, partially obscured, ArH^E), 7.53 (1H, app-dd or partially obscured td, $J_{HD,HC(/HE)}$ 7.6, $J_{HD,HB}$ 1.6 Hz, ArH^D)), 7.48 (1H, app-t, $J_{HC,HB/HD}$ 7.6 Hz, ArH^C), 5.96 (1H, d, $J_{H\text{-}1,H\text{-}2}$ 3.7 Hz, H-1), 4.88 (1H, d, $J_{Ha,Hb}$ 5.5 Hz, ArC*H*^{a}H^b), 4.89-4.83 (1H, m, partially obscured, H-4), 4.36 (1H, d, $J_{H\text{-}2,H\text{-}1}$ 3.7 Hz, H-2) 4.31 (1H, ddd, $J_{H\text{-}5,H\text{-}6'}$ 6.0, $J_{H\text{-}5,H\text{-}6}$ 4.3 Hz, *J* 4.3 Hz, H-5), 3.83 (1H, d, *J* 2.1 Hz, H-3), 3.48 (1H, d, $J_{Hb,Ha}$ 5.7 Hz, ArCH^a*H*^{b}), 2.93, (1H, dd, $J_{H\text{-}6,H\text{-}6'}$ 11.3 Hz, $J_{H\text{-}6,H\text{-}5}$ 4.2 Hz, H-6), 2.83 (1H, dd, $J_{H\text{-}6',H\text{-}6}$ 11.4, $J_{H\text{-}6',H\text{-}5}$ 6.0 Hz, H-6'), 1.49 (3H, s, CH_3), 1.27 (3H, s, CH_3); **δ_C:** 135.9 (ArC$_{quat}$), 135.0 (ArC^E), 133.1 (ArC^B), 132.4 (ArC^D), 128.3 (ArC^C), 113.0 (C$_{quat}$), 106.71 (C-1), 83.9 (C-2), 83.4 (C-4), 71.1 (ArCH$_2$), 69.1 (C-5), 58.8 (C-6), 58.6 (C-3), 26.1, 25.3 (2 x CH_3).

Boronate species. **δ_H:** 7.32 (1H, t, $J_{HD,HE = HD,HC}$ 7.8 Hz, ArH^D), 6.97 (1H, dt, $J_{HE,HD}$ 7.6 Hz, $J_{HE,HC = HE,HB}$ 1.1 Hz, ArH^E), 6.92-6.90 (1H, m, ArH^B), 6.89 (1H, ddd, $J_{HC,HD}$ 8.0 Hz, $J_{HC,HB}$ 2.5 Hz, $J_{HC,HE}$ 0.9 Hz, ArH^C), 5.98 (1H, d, partially obscured, H-1), 4.85-4.80 (1H, obscured, ArC*H*^{a}H^b), 4.40 (1H, d, $J_{H\text{-}2,H\text{-}1}$ 3.7 Hz, H-2), 3.75 (1H, app-d, *J* 3.2 Hz, H-3), 3.46 (1H, d, partially obscured, $J_{Hb,Ha}$ 5.9 Hz, H^a*H*^{b}), 2.96 (1H, app-s, partially obscured, H-5), 2.95-2.90 (1H, obscured, H-6'), 2.84-2.79 (1H, obscured, H-6), 1.49 (3H, s, CH_3 obscured), 1.29 (3H, s, CH_3); **δ_C:** 130.1 (ArC^D), 122.0 (ArC^E), 116.6 (ArC^B), 106.69 (C-1), 71.2 (ArCH$_2$), 58.6 (C-6), 58.4 (C-3), 26.1, 25.2 (2 x CH_3). Other C atoms were not discernible.

δ_B: 29.5 (broad, integration: 4.5), 19.3 (sharp, integration: 1.0).

¹H-NMR

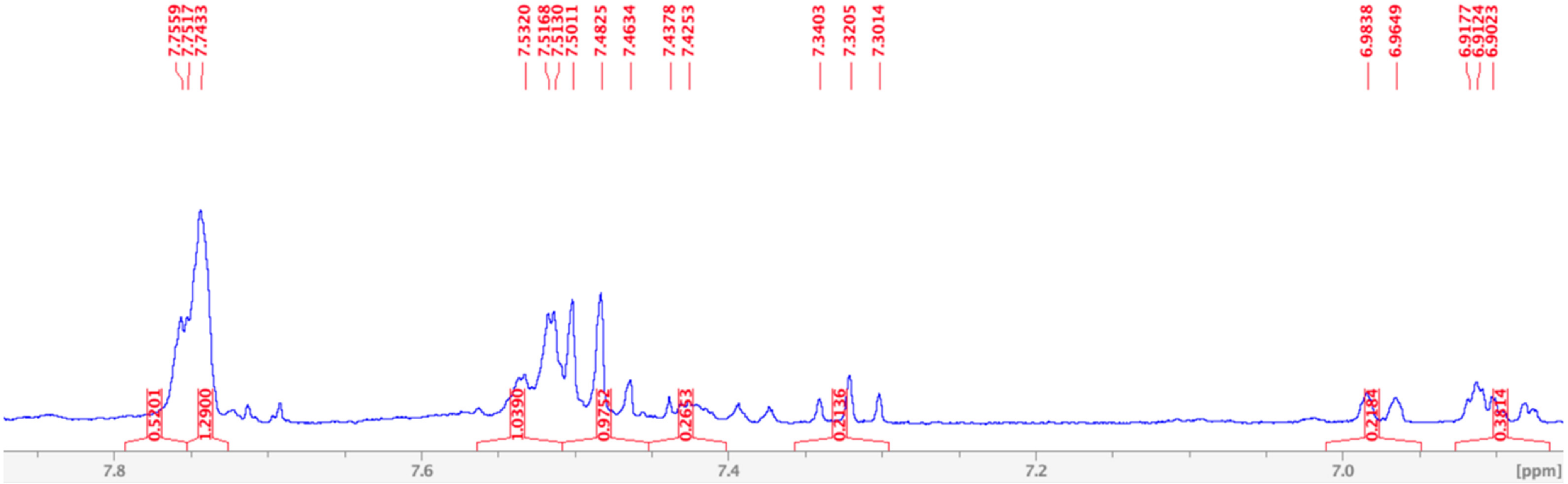
7.7559
7.7517
7.7433
7.5320
7.5168
7.5130
7.5011
7.4825
7.4634
7.4378
7.4253
7.3403
7.3205
7.3014
6.9838
6.9649
6.9177
6.9124
6.9023
0.5201
1.2900
1.0390
0.9752
0.2653
0.2136
0.2184
0.3814
7.8
7.6
7.4
7.2
7.0
[ppm]

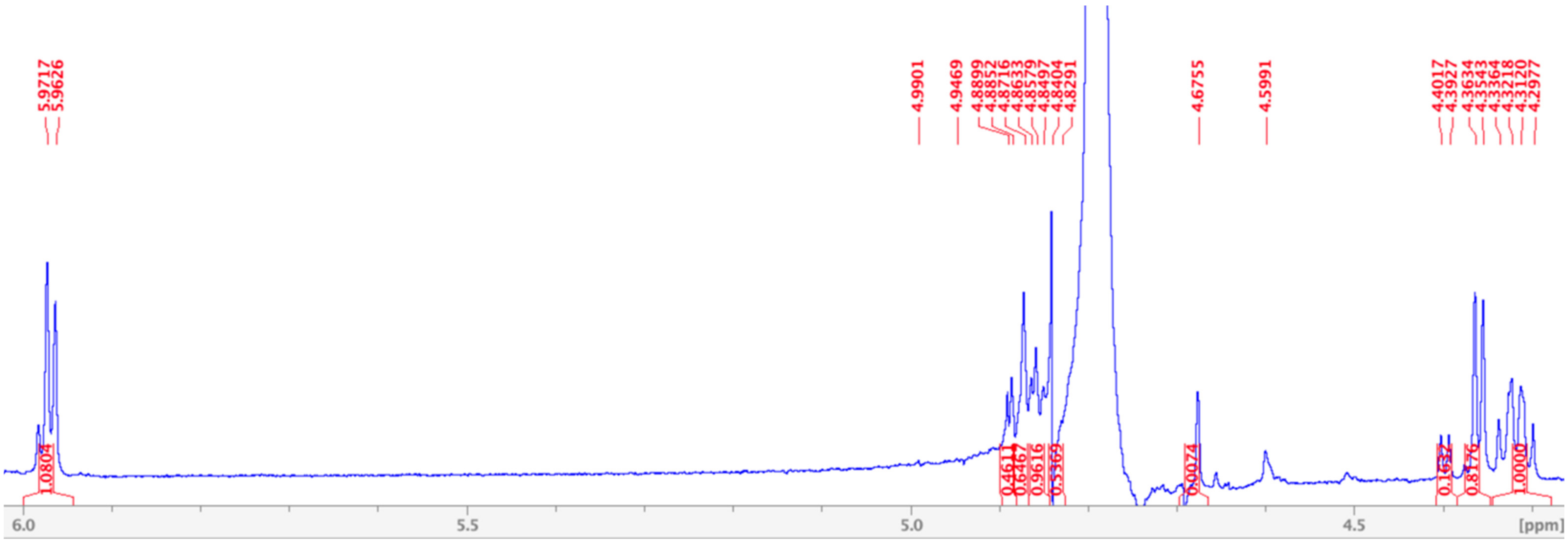
5.9717
5.9626
4.9901
4.9469
4.8899
4.8852
4.8716
4.8633
4.8579
4.8497
4.8404
4.8291
4.6755
4.5991
4.4017
4.3927
4.3634
4.3543
4.3364
4.3218
4.3120
4.2977
1.0804
0.4611
0.6467
0.9616
0.5369
0.0074
0.1632
0.8176
1.0000
6.0
5.5
5.0
4.5
[ppm]

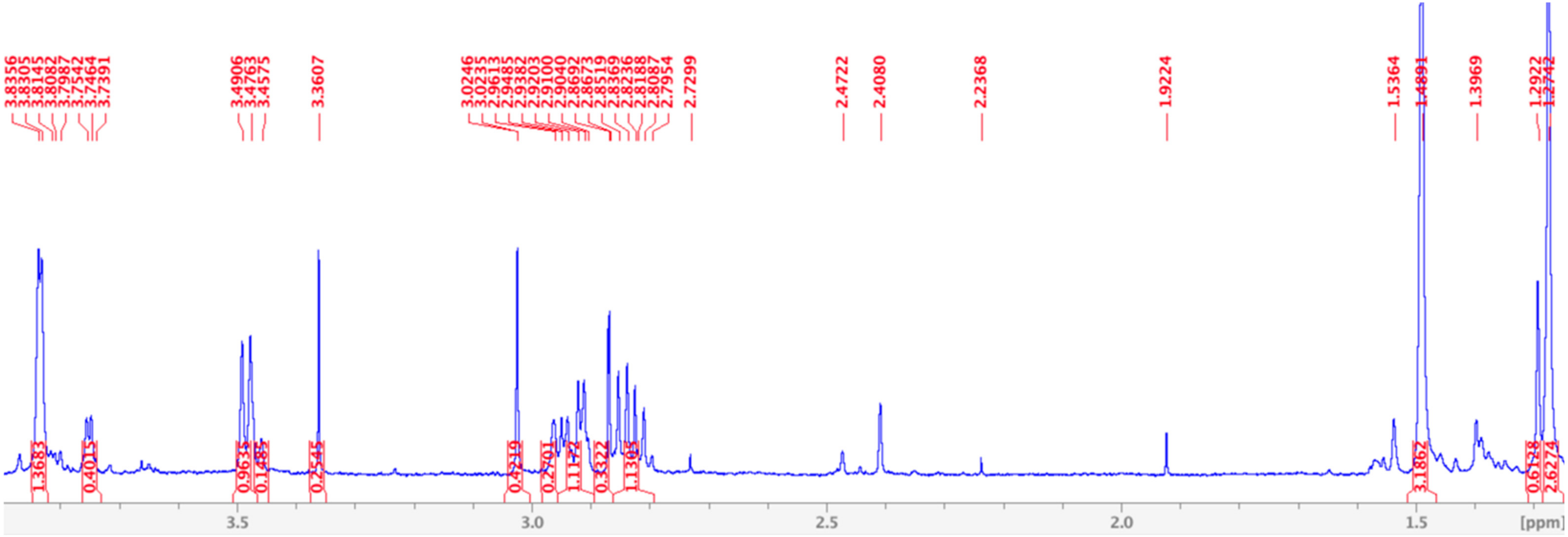

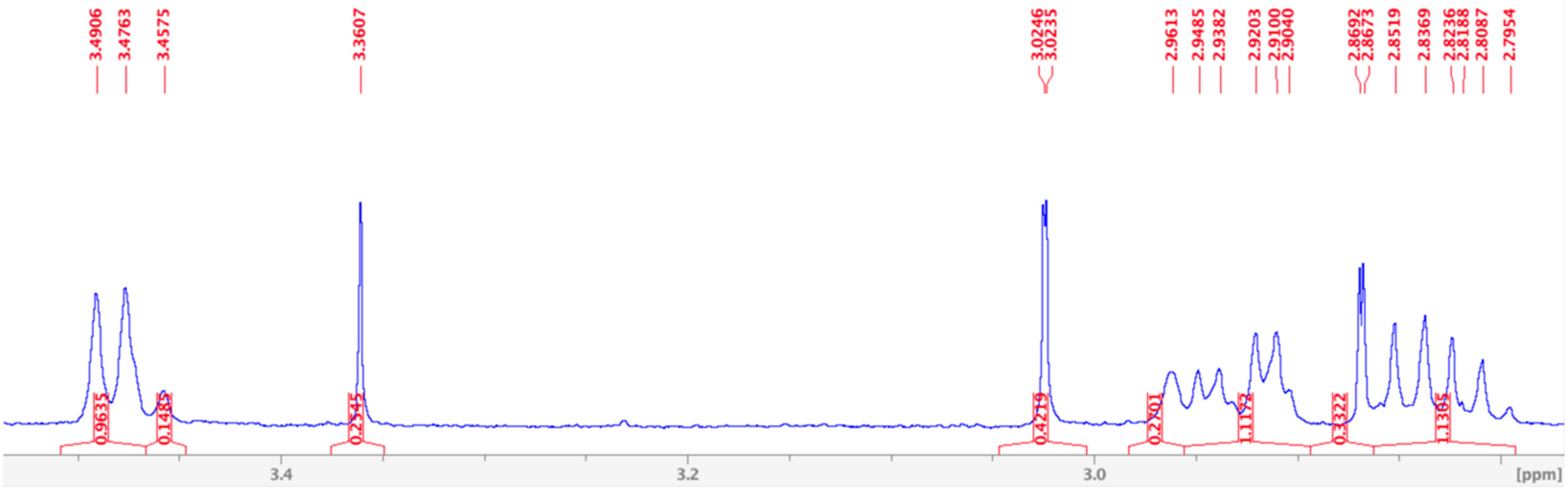

3.4906
3.4763
3.4575
3.3607
3.0246
3.0235
2.9613
2.9485
2.9382
2.9203
2.9100
2.9040
2.8692
2.8673
2.8519
2.8369
2.8236
2.8188
2.8087
2.7954
0.9635
0.1485
0.2545
0.4219
0.2701
1.1174
0.3322
1.1305
3.4
3.2
3.0
[ppm]

^{13}C-NMR

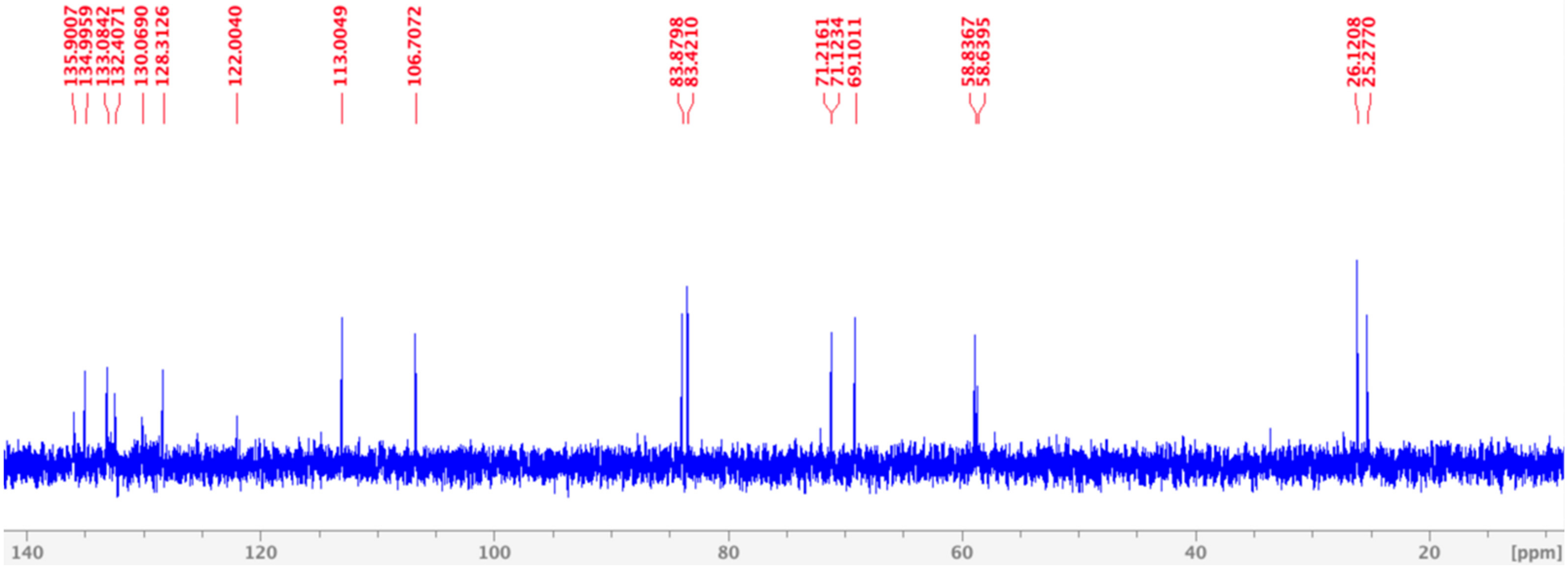
135.9007
134.9959
133.0842
132.4071
130.0690
128.3126
122.0040
113.0049
106.7072
83.8798
83.4210
71.2161
71.1234
69.1011
58.8367
58.6395
26.1208
25.2770
140
120
100
80
60
40
20
[ppm]

DEPT

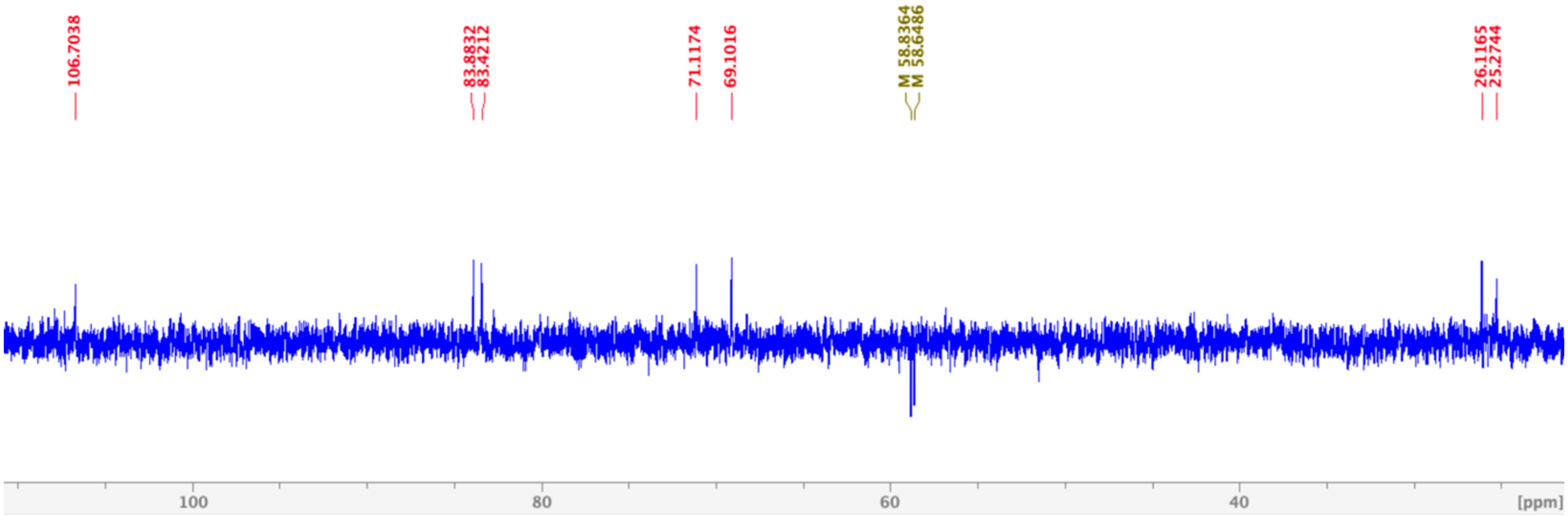
106.7038
83.8832
83.4212
71.1174
69.1016
M 58.8364
M 58.6486
26.1165
25.2744
100
80
60
40
[ppm]

^{11}B-NMR

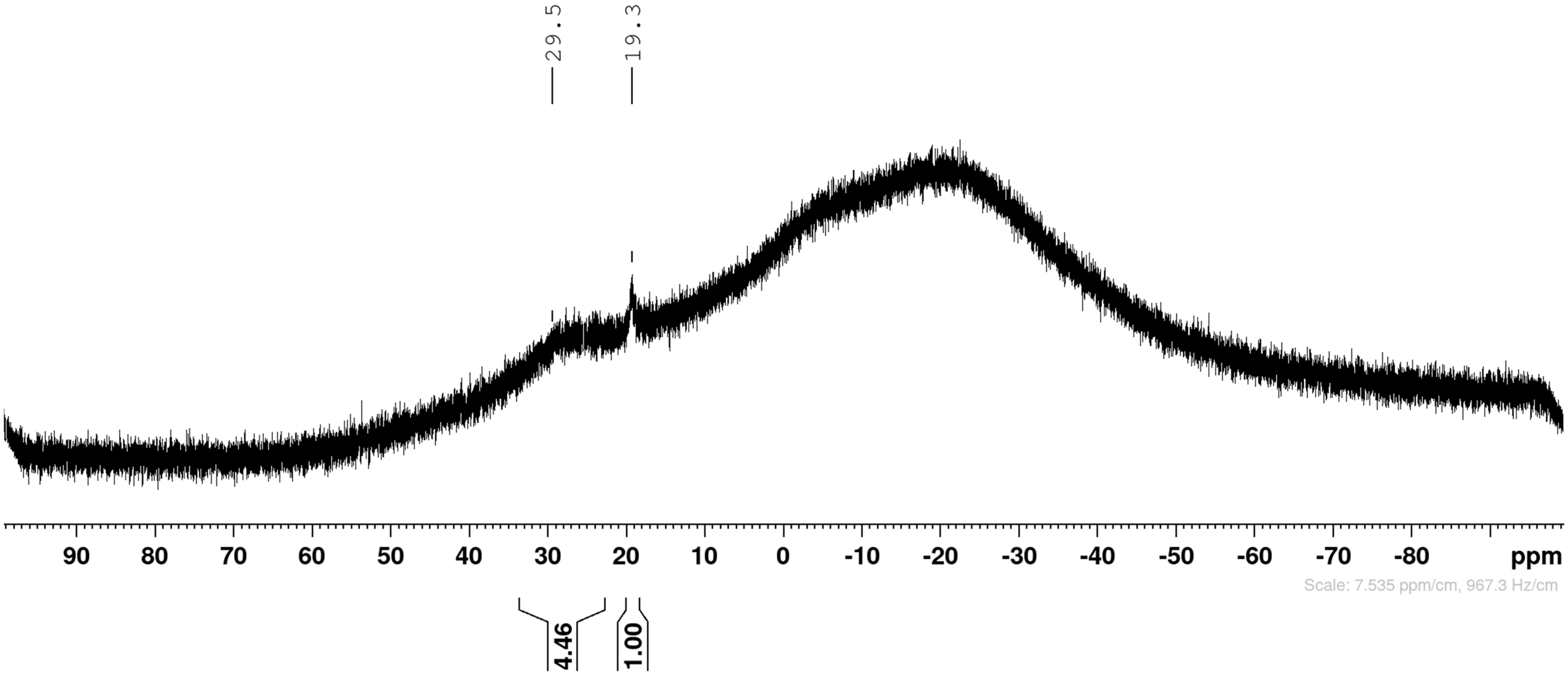

29.5
19.3
90
80
70
60
50
40
30
20
10
0
-10
-20
-30
-40
-50
-60
-70
-80
ppm
Scale: 7.535 ppm/cm, 967.3 Hz/cm
4.46
1.00

COSY

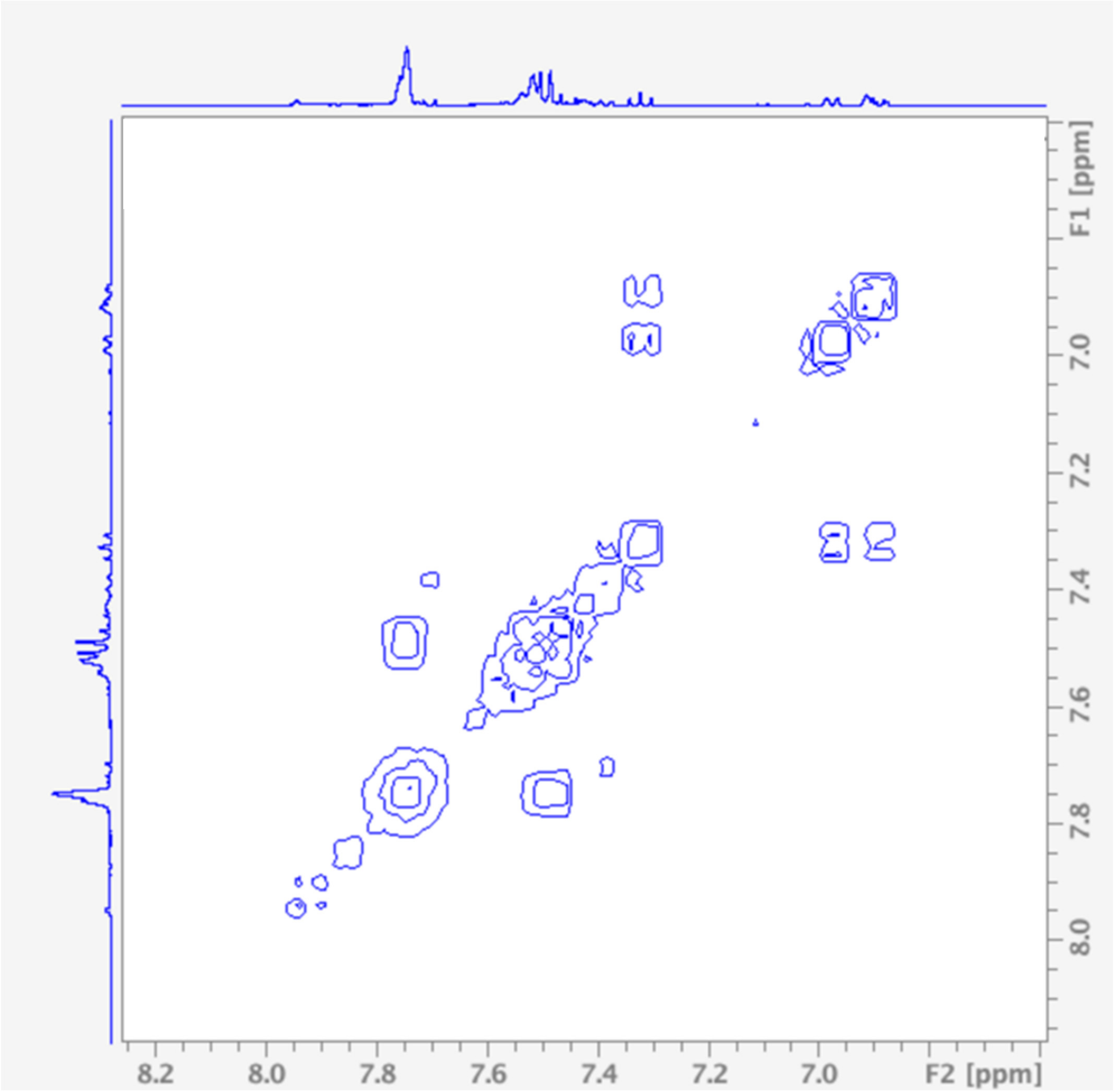

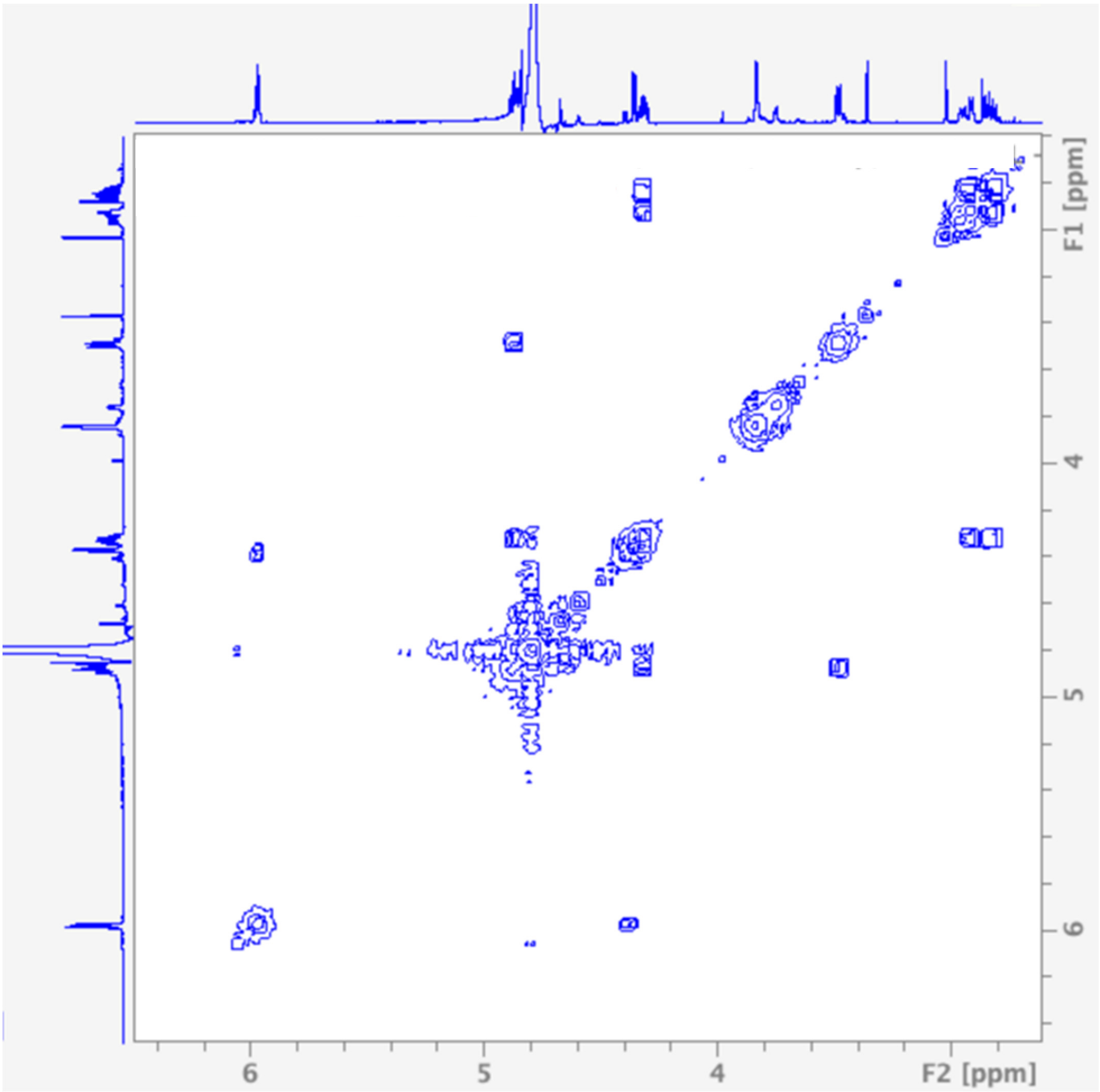
F1 [ppm]
4
5
6
6
5
4
F2 [ppm]

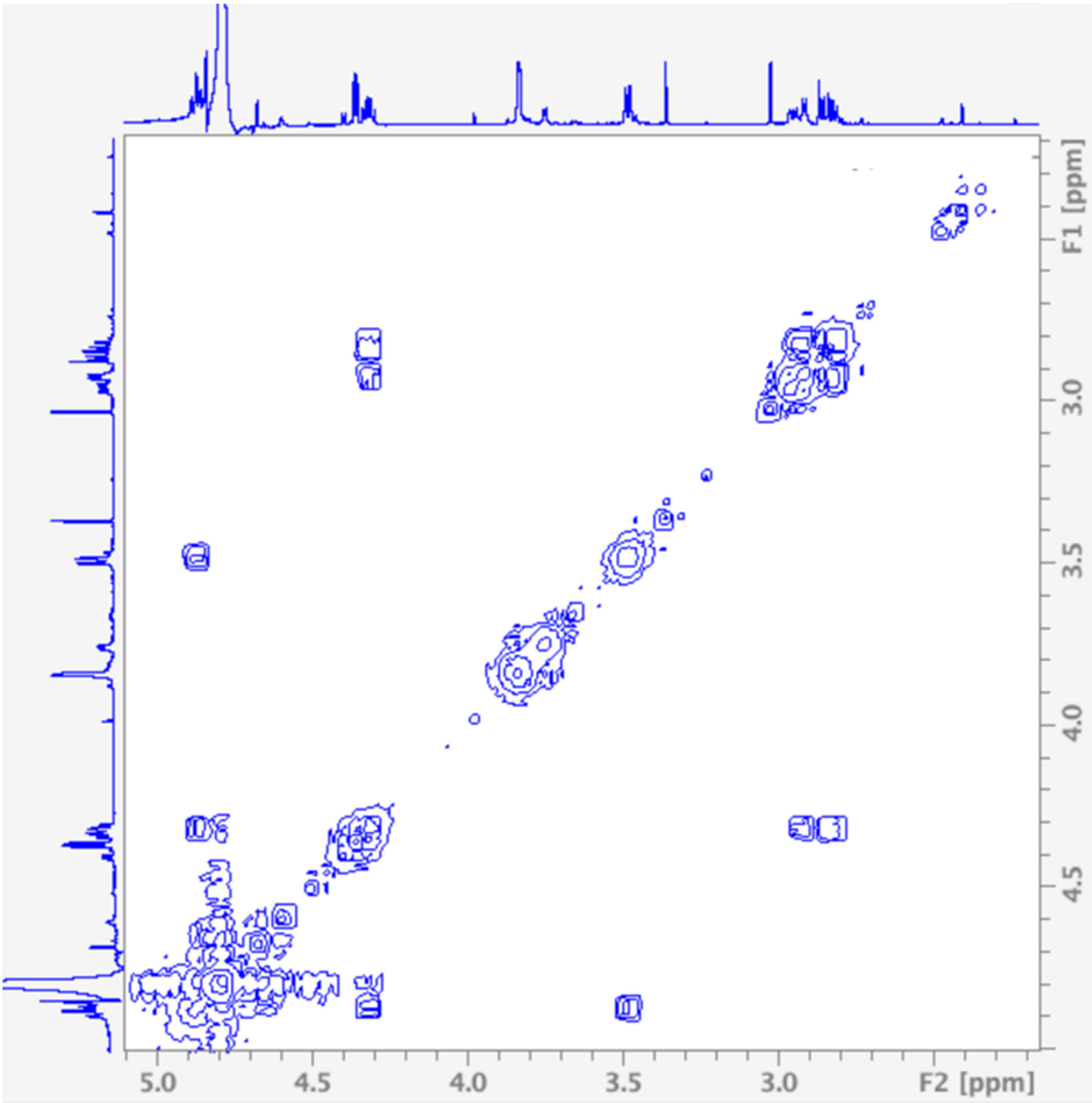
F1 [ppm]
3.0
3.5
4.0
4.5
5.0
4.5
4.0
3.5
3.0
F2 [ppm]

HSQC

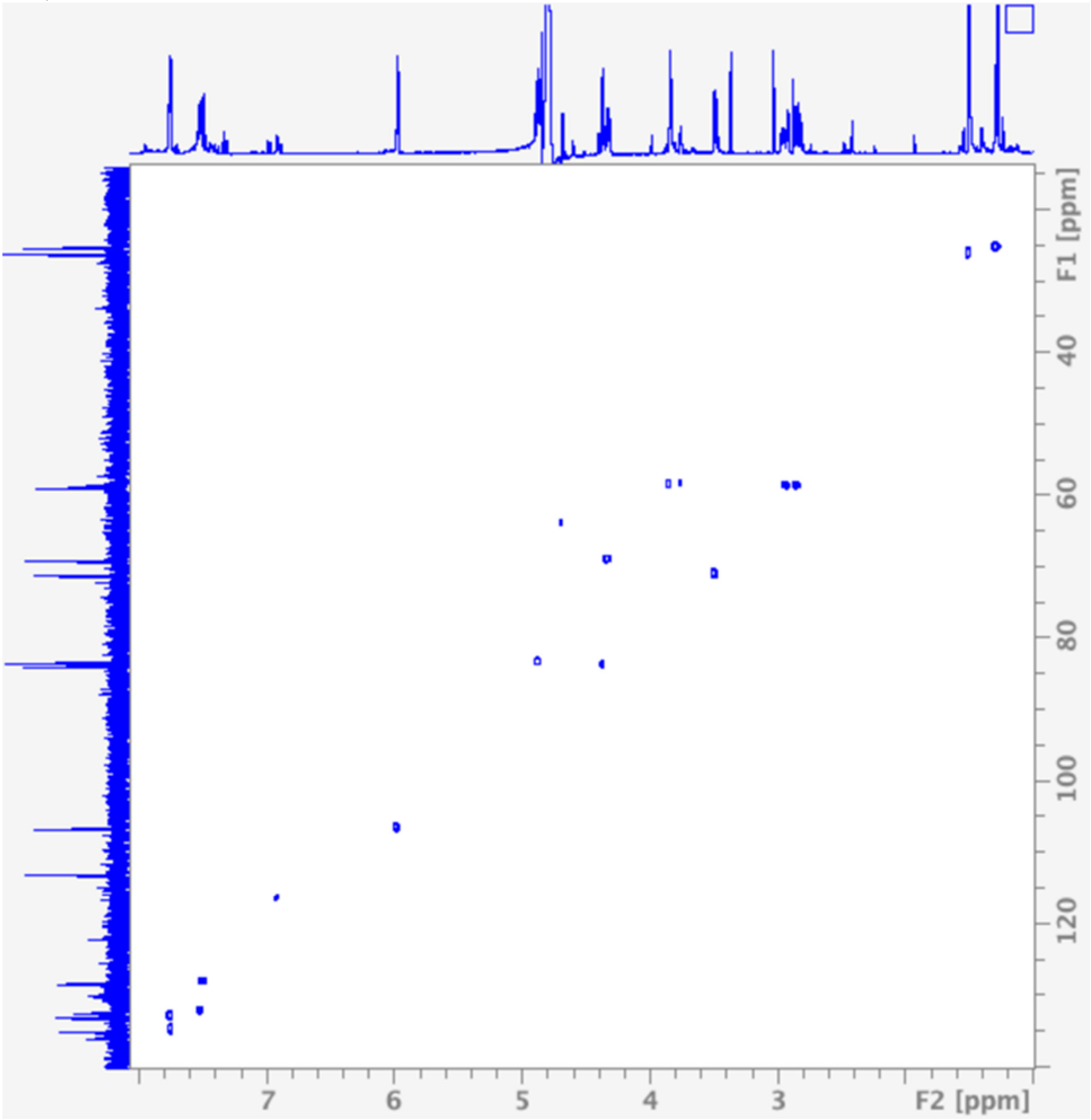

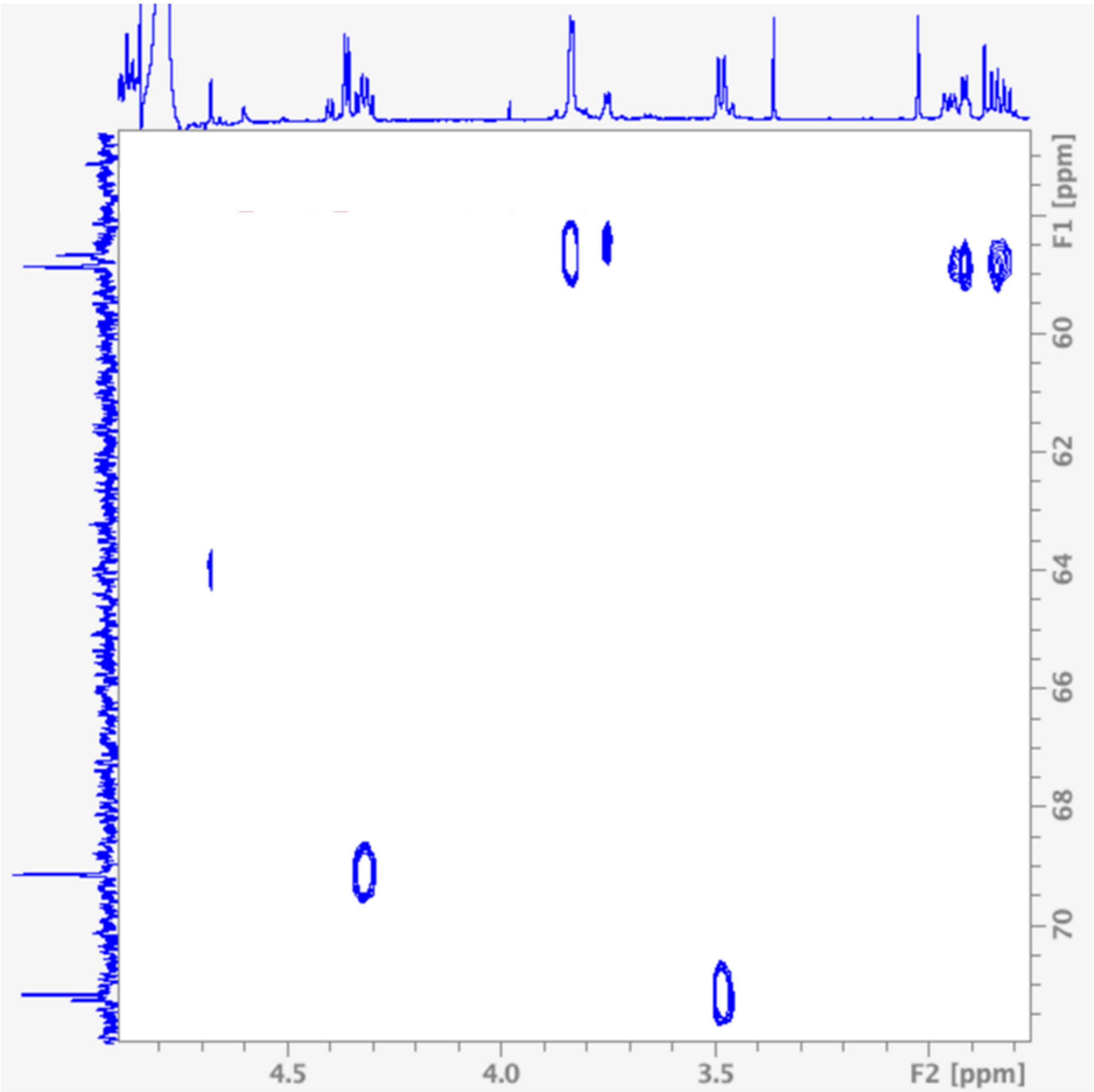
F1 [ppm]
60
62
64
66
68
70
4.5
4.0
3.5
F2 [ppm]

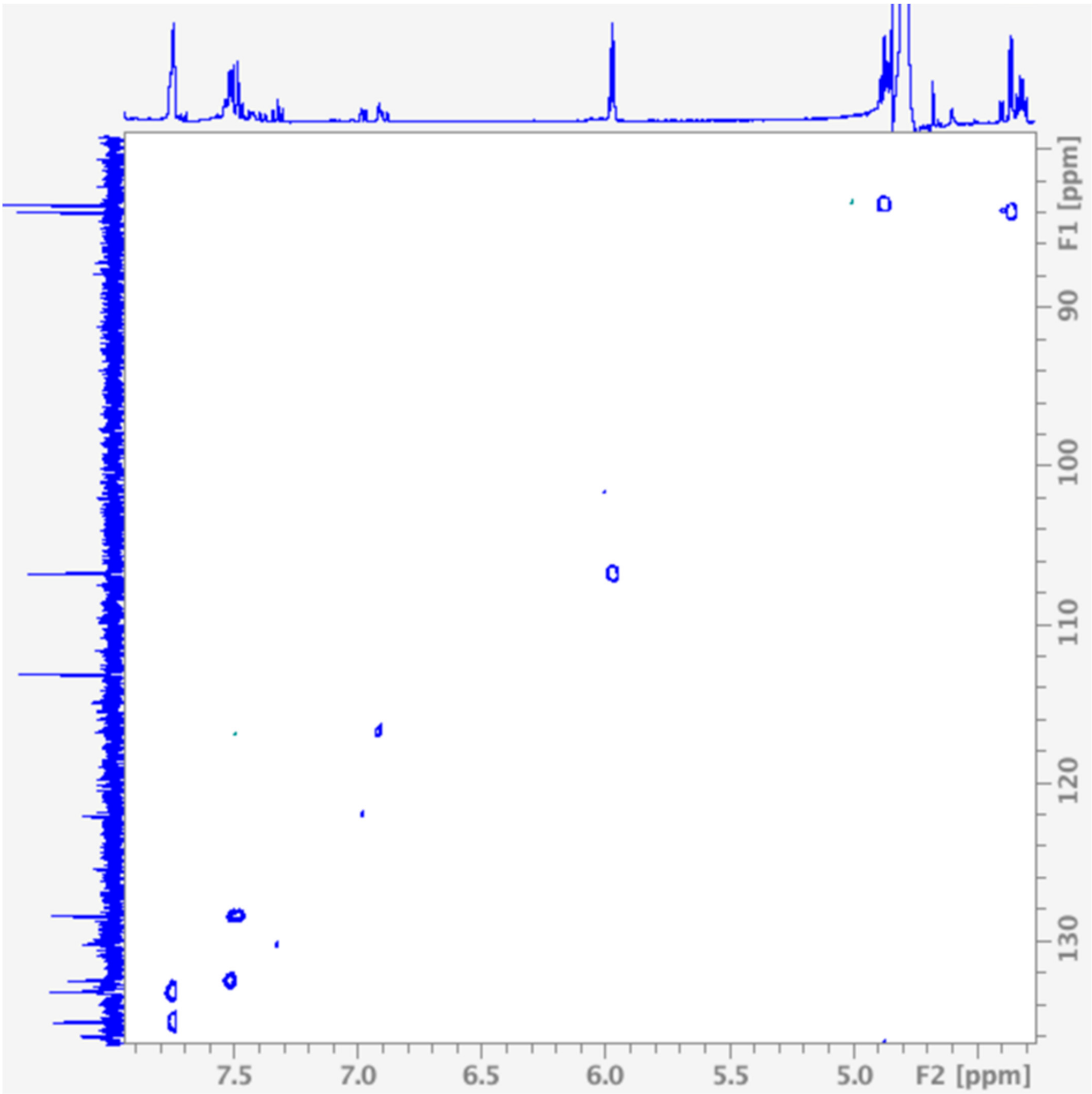
F1 [ppm]
90
100
110
120
130
7.5
7.0
6.5
6.0
5.5
5.0
F2 [ppm]

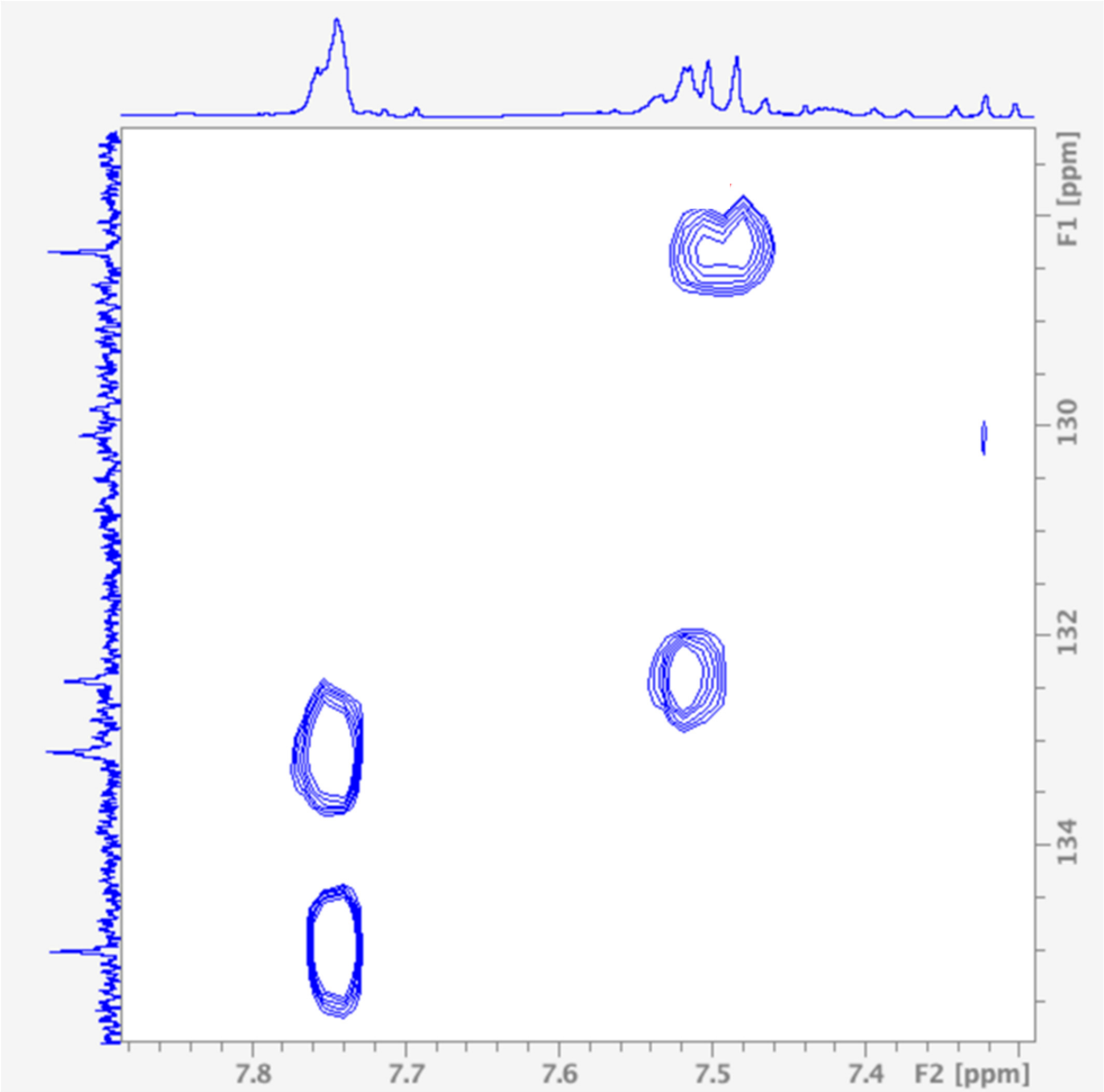
F1 [ppm]
130
132
134
7.8
7.7
7.6
7.5
7.4
F2 [ppm]

Figure S19. ^{1}H-NMR spectrum (400 MHz, D_2O) of compound **ortho 5** with colour-coded signals, highlighting the boronic acid and boronate forms they belong to, with interpretation of the isolated signals and tentative interpretation of the overlapping ones. Namely, the orange designates the boronic acid form and indigo designates the boronate form. A) section 7.80 ppm to 6.80 ppm; B) section 6.10 ppm to 4.20 ppm; C) section 3.90 ppm to 2.70 ppm; D) section 1.60 ppm to 1.25 ppm. Highlighted are also the principal COSY correlations to hydrogen atoms within the same spin systems.

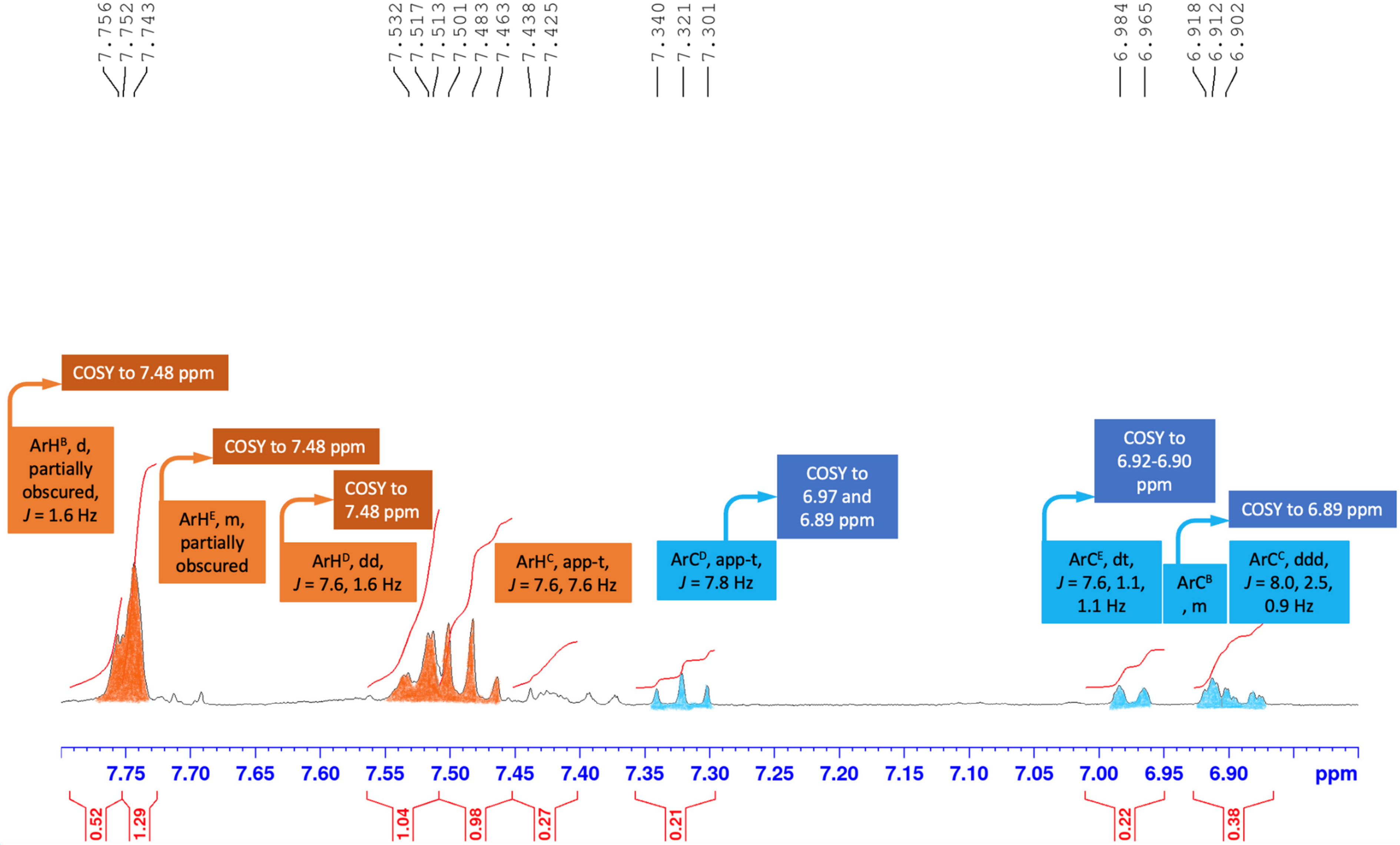

A

B

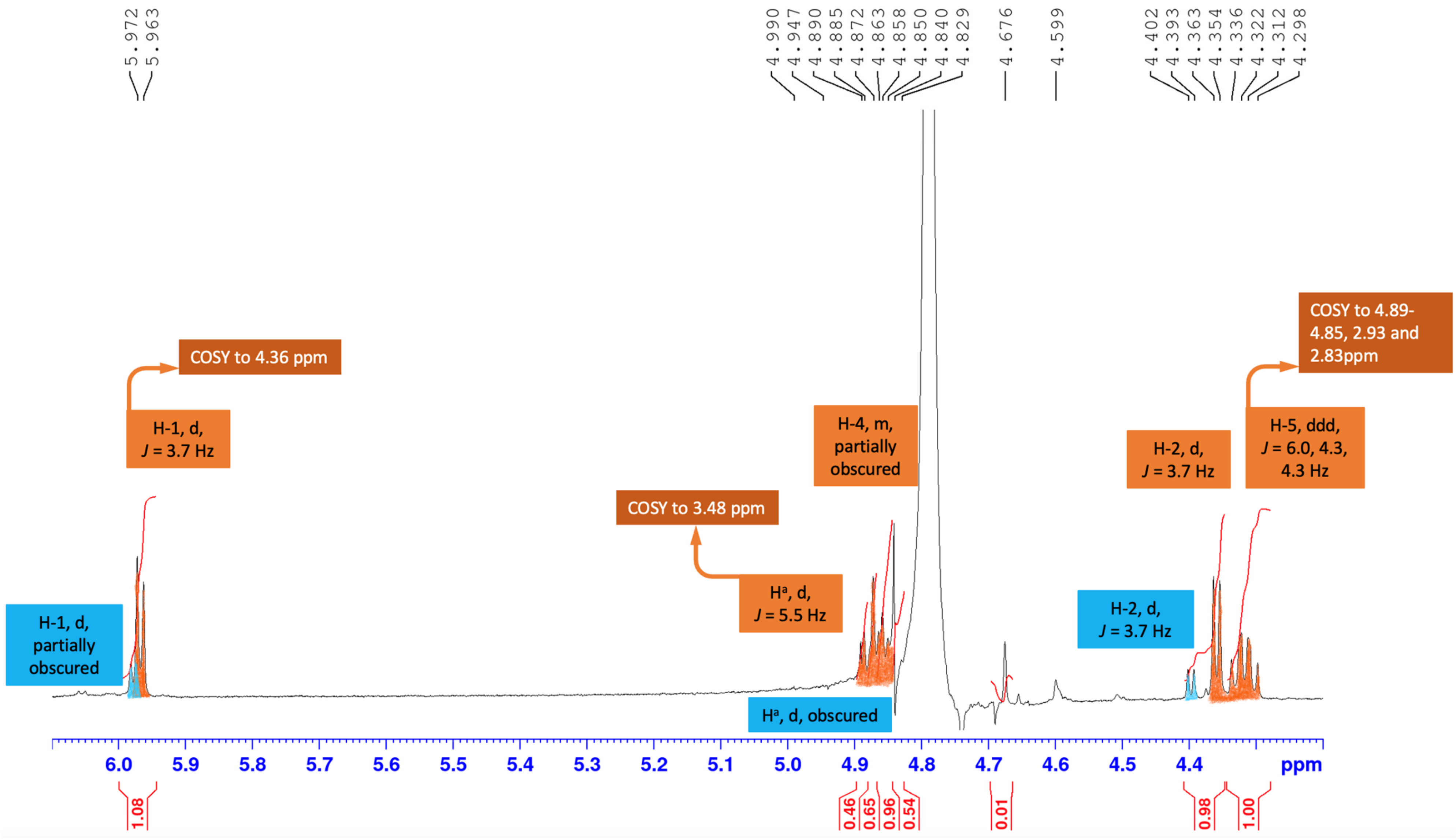
5.972
5.963
4.990
4.947
4.890
4.885
4.872
4.863
4.858
4.850
4.840
4.829
4.676
4.599
4.402
4.393
4.363
4.354
4.336
4.322
4.312
4.298
COSY to 4.36 ppm
H-1, d, J = 3.7 Hz
H-1, d, partially obscured
COSY to 3.48 ppm
Hᵃ, d, J = 5.5 Hz
H-4, m, partially obscured
Hᵃ, d, obscured
COSY to 4.89-4.85, 2.93 and 2.83ppm
H-2, d, J = 3.7 Hz
H-5, ddd, J = 6.0, 4.3, 4.3 Hz
H-2, d, J = 3.7 Hz
6.0
5.9
5.8
5.7
5.6
5.5
5.4
5.3
5.2
5.1
5.0
4.9
4.8
4.7
4.6
4.5
4.4
ppm
1.08
0.46
0.65
0.96
0.54
0.01
0.98
1.00

C

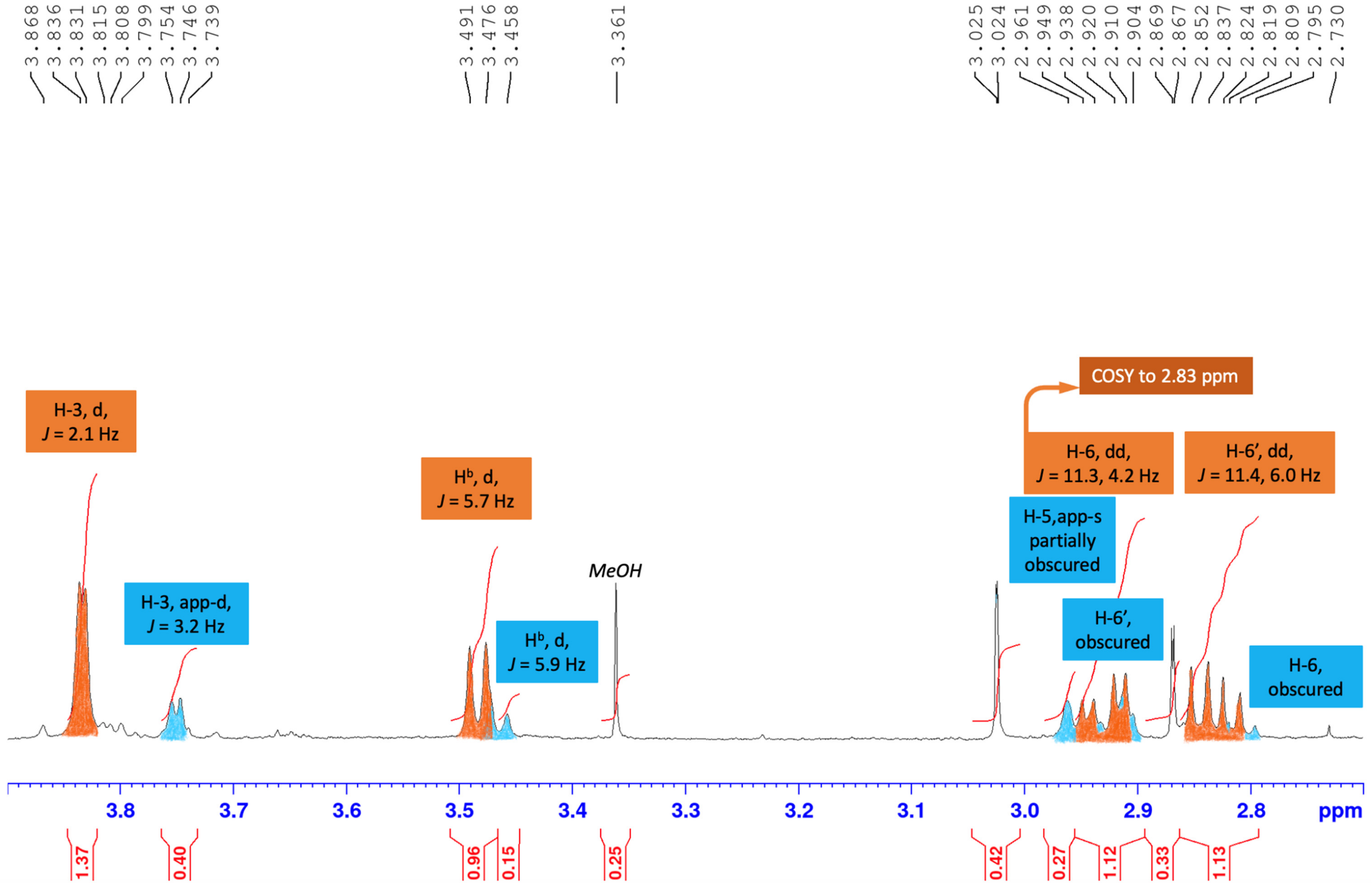
3.868
3.836
3.831
3.815
3.808
3.799
3.754
3.746
3.739
3.491
3.476
3.458
3.361
3.025
3.024
2.961
2.949
2.938
2.920
2.910
2.904
2.869
2.867
2.852
2.837
2.824
2.819
2.809
2.795
2.730
COSY to 2.83 ppm
H-3, d, J = 2.1 Hz
H-3, app-d, J = 3.2 Hz
Hb, d, J = 5.7 Hz
Hb, d, J = 5.9 Hz
MeOH
H-6, dd, J = 11.3, 4.2 Hz
H-6', dd, J = 11.4, 6.0 Hz
H-5, app-s partially obscured
H-6', obscured
H-6, obscured
3.8
3.7
3.6
3.5
3.4
3.3
3.2
3.1
3.0
2.9
2.8
ppm
1.37
0.40
0.96
0.15
0.25
0.42
0.27
1.12
0.33
1.13

D

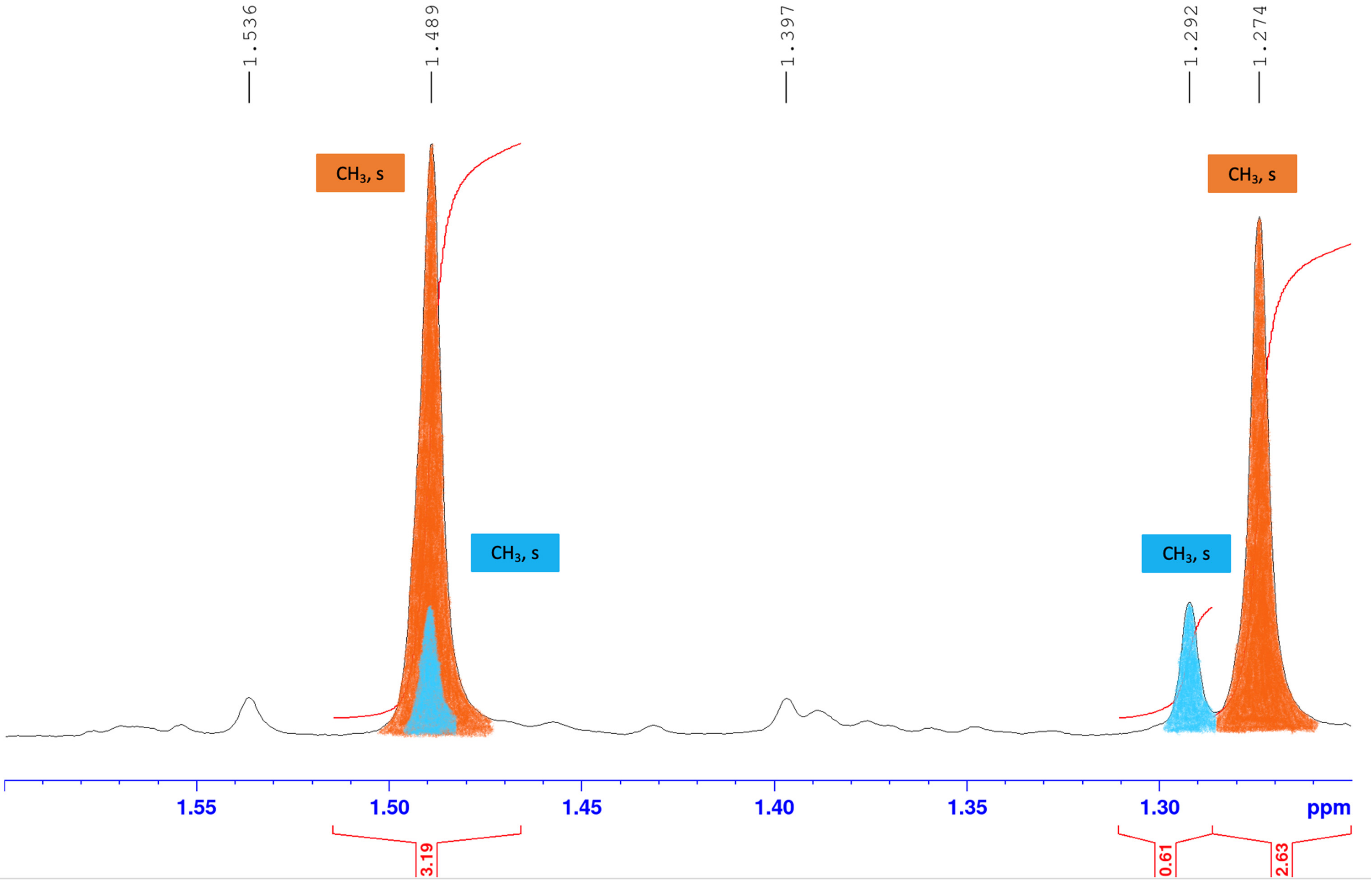
1.536
1.489
1.397
1.292
1.274
CH3, s
CH3, s
CH3, s
CH3, s
1.55
1.50
1.45
1.40
1.35
1.30
ppm
3.19
0.61
2.63

Figure S20. ^{13}C-NMR spectrum (100 MHz, D_2O) of compound **ortho 5** with colour-coded signals, highlighting the boronic acid and boronate forms they belong to, with interpretation of the isolated signals and tentative interpretation of the overlapping ones. Namely, the orange designates the boronic acid form and indigo designates the boronate form. A) section 140 ppm to 23 ppm.

A

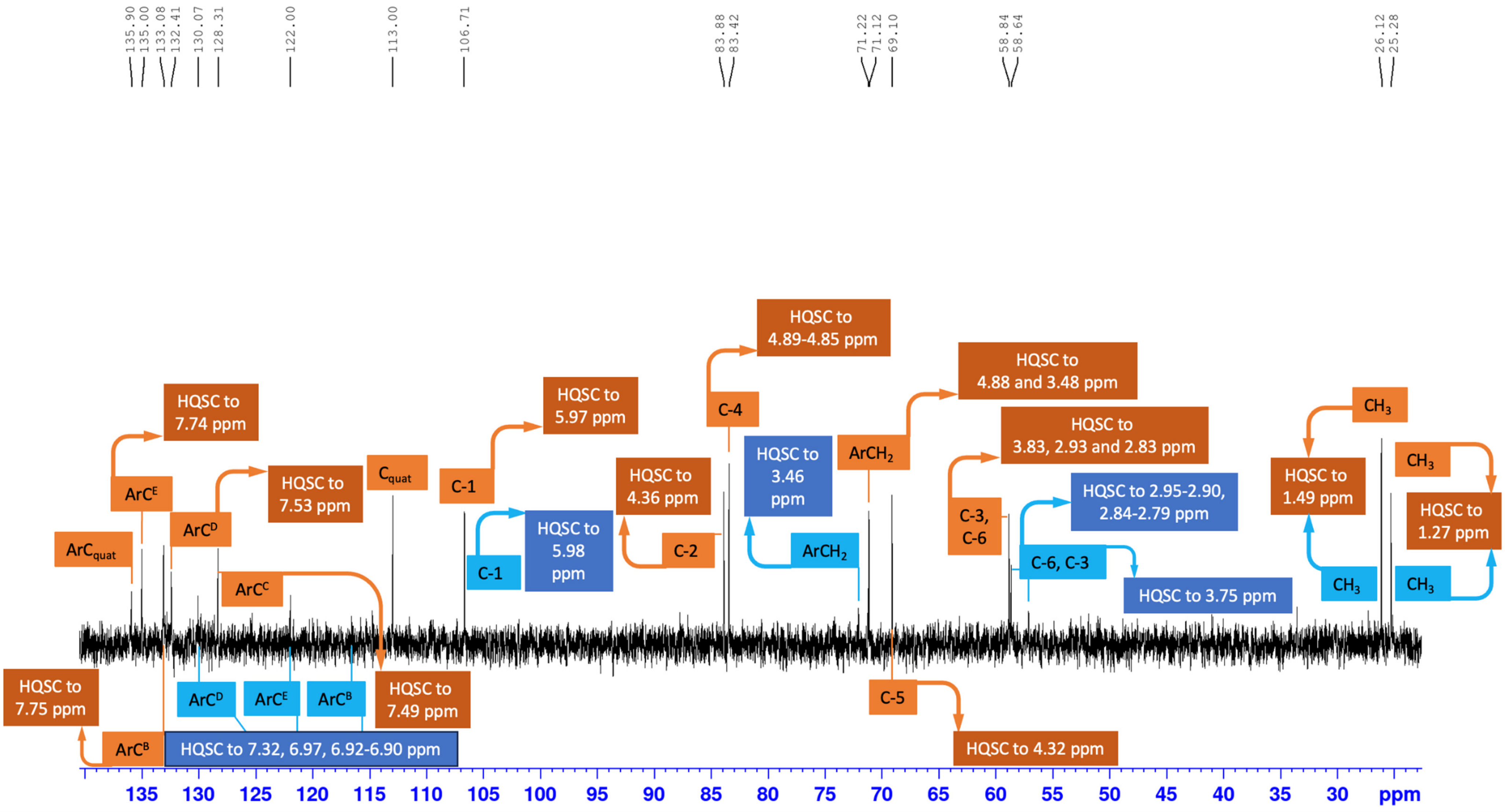

NMR Experimental details

	para 6	para 7	para 8
^{1}H	F2 - Acquisition Parameters<br>Date_ 20171025<br>Time 15.39<br>INSTRUM spect<br>PROBHD 5 mm PABBO BB-<br>PULPROG zg<br>TD 48076<br>SOLVENT CDCl3<br>NS 8<br>DS 2<br>SWH 6002.401 Hz<br>FIDRES 0.124852 Hz<br>AQ 4.0047307 sec<br>RG 23.44<br>DW 83.300 usec<br>DE 16.70 usec<br>TE 298.2 K<br>D1 5.00000000 sec<br>TD0 1<br><br>======== CHANNEL f1 ========<br>SFO1 400.1320007 MHz<br>NUC1 1H<br>P1 15.00 usec<br>PLW1 12.50300026 W<br><br>F2 - Processing parameters<br>SI 32768<br>SF 400.1300096 MHz<br>WDW EM<br>SSB 0<br>LB 0.30 Hz<br>GB 0<br>PC 1.00	F2 - Acquisition Parameters<br>Date_ 20180209<br>Time 10.59<br>INSTRUM spect<br>PROBHD 5 mm PABBO BB-<br>PULPROG zg30<br>TD 65536<br>SOLVENT D2O<br>NS 16<br>DS 2<br>SWH 8012.820 Hz<br>FIDRES 0.122266 Hz<br>AQ 4.0894465 sec<br>RG 86.04<br>DW 62.400 usec<br>DE 6.50 usec<br>TE 298.2 K<br>D1 1.00000000 sec<br>TD0 1<br><br>======== CHANNEL f1 ========<br>SFO1 400.1324710 MHz<br>NUC1 1H<br>P1 15.00 usec<br>PLW1 12.50300026 W<br><br>F2 - Processing parameters<br>SI 65536<br>SF 400.1299656 MHz<br>WDW EM<br>SSB 0<br>LB 0.30 Hz<br>GB 0<br>PC 1.00	F2 - Acquisition Parameters<br>Date_ 20180221<br>Time 11.07<br>INSTRUM spect<br>PROBHD 5 mm PABBO BB-<br>PULPROG zg30<br>TD 65536<br>SOLVENT D2O<br>NS 16<br>DS 2<br>SWH 8012.820 Hz<br>FIDRES 0.122266 Hz<br>AQ 4.0894465 sec<br>RG 33.39<br>DW 62.400 usec<br>DE 6.50 usec<br>TE 298.2 K<br>D1 1.00000000 sec<br>TD0 1<br><br>======== CHANNEL f1 ========<br>SFO1 400.1324710 MHz<br>NUC1 1H<br>P1 15.00 usec<br>PLW1 12.50300026 W<br><br>F2 - Processing parameters<br>SI 65536<br>SF 400.1300000 MHz<br>WDW EM<br>SSB 0<br>LB 0.30 Hz<br>GB 0<br>PC 1.00
^{13}C	F2 - Acquisition Parameters<br>Date_ 20171027<br>Time 21.00<br>INSTRUM spect<br>PROBHD 5 mm PABBO BB-<br>PULPROG zgpg30<br>TD 65536<br>SOLVENT CDCl3<br>NS 4000<br>DS 4<br>SWH 22058.824 Hz<br>FIDRES 0.336591 Hz<br>AQ 1.4854827 sec<br>RG 192.58<br>DW 22.667 usec<br>DE 6.50 usec<br>TE 298.2 K<br>D1 2.00000000 sec<br>D11 0.03000000 sec<br>TD0 1<br><br>======== CHANNEL f1 ========<br>SFO1 100.6223253 MHz<br>NUC1 13C<br>P1 9.80 usec<br>PLW1 60.95399857 W<br><br>======== CHANNEL f2 ========<br>SFO2 400.1316005 MHz<br>NUC2 1H<br>CPDPRG[2 waltz16<br>PCPD2 90.00 usec<br>PLW2 12.50300026 W<br>PLW12 0.34731001 W<br>PLW13 0.28132001 W<br><br>F2 - Processing parameters<br>SI 65536<br>SF 100.6127589 MHz<br>WDW EM<br>SSB 0<br>LB 1.00 Hz<br>GB 0<br>PC 1.40	F2 - Acquisition Parameters<br>Date_ 20180213<br>Time 23.03<br>INSTRUM spect<br>PROBHD 5 mm PABBO BB-<br>PULPROG zgpg30<br>TD 65536<br>SOLVENT D2O<br>NS 3000<br>DS 4<br>SWH 22058.824 Hz<br>FIDRES 0.336591 Hz<br>AQ 1.4854827 sec<br>RG 192.58<br>DW 22.667 usec<br>DE 6.50 usec<br>TE 298.2 K<br>D1 2.00000000 sec<br>D11 0.03000000 sec<br>TD0 1<br><br>======== CHANNEL f1 ========<br>SFO1 100.6223253 MHz<br>NUC1 13C<br>P1 9.80 usec<br>PLW1 60.95399857 W<br><br>======== CHANNEL f2 ========<br>SFO2 400.1316005 MHz<br>NUC2 1H<br>CPDPRG[2 waltz16<br>PCPD2 90.00 usec<br>PLW2 12.50300026 W<br>PLW12 0.34731001 W<br>PLW13 0.28132001 W<br><br>F2 - Processing parameters<br>SI 65536<br>SF 100.6127690 MHz<br>WDW EM<br>SSB 0<br>LB 1.00 Hz<br>GB 0<br>PC 1.40	F2 - Acquisition Parameters<br>Date_ 20180221<br>Time 20.00<br>INSTRUM spect<br>PROBHD 5 mm PABBO BB-<br>PULPROG zgpg30<br>TD 65536<br>SOLVENT D2O<br>NS 3000<br>DS 4<br>SWH 22058.824 Hz<br>FIDRES 0.336591 Hz<br>AQ 1.4854827 sec<br>RG 192.58<br>DW 22.667 usec<br>DE 6.50 usec<br>TE 298.1 K<br>D1 2.00000000 sec<br>D11 0.03000000 sec<br>TD0 1<br><br>======== CHANNEL f1 ========<br>SFO1 100.6223253 MHz<br>NUC1 13C<br>P1 9.80 usec<br>PLW1 60.95399857 W<br><br>======== CHANNEL f2 ========<br>SFO2 400.1316005 MHz<br>NUC2 1H<br>CPDPRG[2 waltz16<br>PCPD2 90.00 usec<br>PLW2 12.50300026 W<br>PLW12 0.34731001 W<br>PLW13 0.28132001 W<br><br>F2 - Processing parameters<br>SI 65536<br>SF 100.6127690 MHz<br>WDW EM<br>SSB 0<br>LB 1.00 Hz<br>GB 0<br>PC 1.40

DEPT

F2 - Acquisition Parameters			
Date_	20171025	20180209	20180221
Time	15.56	15.41	16.18
INSTRUM	spect	spect	spect
PROBHD	5 mm PABBO BB-	5 mm PABBO BB-	5 mm PABBO BB-
PULPROG	deptqgpsp	deptqgpsp	deptqgpsp
TD	65536	65536	65536
SOLVENT	CDCl3	D2O	D2O
NS	256	256	256
DS	4	4	4
SWH	22058.824 Hz	22058.824 Hz	22058.824 Hz
FIDRES	0.336591 Hz	0.336591 Hz	0.336591 Hz
AQ	1.4854827 sec	1.4854827 sec	1.4854827 sec
RG	192.58	192.58	192.58
DW	22.667 usec	22.667 usec	22.667 usec
DE	6.50 usec	6.50 usec	6.50 usec
TE	298.2 K	298.2 K	298.1 K
CNST2	145.0000000	145.0000000	145.0000000
CNST12	1.5000000	1.5000000	1.5000000
D1	2.00000000 sec	2.00000000 sec	2.00000000 sec
D2	0.00344828 sec	0.00344828 sec	0.00344828 sec
D12	0.00002000 sec	0.00002000 sec	0.00002000 sec
D16	0.00020000 sec	0.00020000 sec	0.00020000 sec
TD0	1	1	1
======== CHANNEL f1 ========			
SFO1	100.6223258 MHz	100.6223258 MHz	100.6223258 MHz
NUC1	13C	13C	13C
P1	9.80 usec	9.80 usec	9.80 usec
P13	2000.00 usec	2000.00 usec	2000.00 usec
PLW0	0 W	0 W	0 W
PLW1	60.95399857 W	60.95399857 W	60.95399857 W
SPNAM[5]	Crp60comp.4	Crp60comp.4	Crp60comp.4
SPOAL5	0.500	0.500	0.500
SPOFFS5	0 Hz	0 Hz	0 Hz
SPW5	8.94419956 W	8.94419956 W	8.94419956 W
======== CHANNEL f2 ========			
SFO2	400.1316005 MHz	400.1316005 MHz	400.1316005 MHz
NUC2	1H	1H	1H
CPDPRG[2	waltz16	waltz16	waltz16
P0	22.50 usec	22.50 usec	22.50 usec
P3	15.00 usec	15.00 usec	15.00 usec
P4	30.00 usec	30.00 usec	30.00 usec
PCPD2	90.00 usec	90.00 usec	90.00 usec
PLW2	12.50300026 W	12.50300026 W	12.50300026 W
PLW12	0.34731001 W	0.34731001 W	0.34731001 W
====== GRADIENT CHANNEL =====			
GPNAM[1]	SMSQ10.32	SMSQ10.32	SMSQ10.32
GPNAM[2]	SMSQ10.32	SMSQ10.32	SMSQ10.32
GPNAM[3]	SMSQ10.32	SMSQ10.32	SMSQ10.32
GPZ1	31.00 %	31.00 %	31.00 %
GPZ2	31.00 %	31.00 %	31.00 %
GPZ3	31.00 %	31.00 %	31.00 %
P16	1000.00 usec	1000.00 usec	1000.00 usec
F2 - Processing parameters			
SI	65536	65536	65536
SF	100.6127584 MHz	100.6127690 MHz	100.6127690 MHz
WDW	EM	EM	EM
SSB	0	0	0
LB	1.00 Hz	1.00 Hz	1.00 Hz
GB	0	0	0
PC	1.40	1.40	1.40

^{11}B	F2 - Acquisition Parameters	F2 - Acquisition Parameters	F2 - Acquisition Parameters
	Date_ 20171026	Date_ 20180212	Date_ 20180221
	Time 5.49	Time 5.50	Time 20.24
	INSTRUM spect	INSTRUM spect	INSTRUM spect
	PROBHD 5 mm PABBO BB-	PROBHD 5 mm PABBO BB-	PROBHD 5 mm PABBO BB-
	PULPROG zg	PULPROG zg	PULPROG zg
	TD 65536	TD 65536	TD 65536
	SOLVENT CDCl3	SOLVENT D2O	SOLVENT D2O
	NS 128	NS 128	NS 128
	DS 4	DS 4	DS 4
	SWH 25510.203 Hz	SWH 25510.203 Hz	SWH 25510.203 Hz
	FIDRES 0.389255 Hz	FIDRES 0.389255 Hz	FIDRES 0.389255 Hz
	AQ 1.2845056 sec	AQ 1.2845056 sec	AQ 1.2845056 sec
	RG 192.58	RG 192.58	RG 192.58
	DW 19.600 usec	DW 19.600 usec	DW 19.600 usec
	DE 6.50 usec	DE 6.50 usec	DE 6.50 usec
	TE 298.1 K	TE 298.1 K	TE 298.1 K
	D1 1.00000000 sec	D1 1.00000000 sec	D1 1.00000000 sec
	TD0 1	TD0 1	TD0 1
	======== CHANNEL f1 ========	======== CHANNEL f1 ========	======== CHANNEL f1 ========
	SFO1 128.3776052 MHz	SFO1 128.3776052 MHz	SFO1 128.3776052 MHz
	NUC1 11B	NUC1 11B	NUC1 11B
	P1 17.05 usec	P1 17.05 usec	P1 17.05 usec
	PLW1 11.69499969 W	PLW1 11.69499969 W	PLW1 11.69499969 W
	F2 - Processing parameters	F2 - Processing parameters	F2 - Processing parameters
	SI 32768	SI 32768	SI 32768
	SF 128.3776052 MHz	SF 128.3776052 MHz	SF 128.3776052 MHz
	WDW no	WDW no	WDW no
	SSB 0	SSB 0	SSB 0
	LB 0 Hz	LB 0 Hz	LB 0 Hz
	GB 0	GB 0	GB 0
	PC 1.40	PC 1.40	PC 1.40

**COSY**	F2 - Acquisition Parameters<br>Date_ 20171025<br>Time 15.58<br>INSTRUM spect<br>PROBHD 5 mm PABBO BB-<br>PULPROG cosygpqf<br>TD 2048<br>SOLVENT CDCl3<br>NS 1<br>DS 8<br>SWH 4807.692 Hz<br>FIDRES 2.347506 Hz<br>AQ 0.2129920 sec<br>RG 86.04<br>DW 104.000 usec<br>DE 6.50 usec<br>TE 298.0 K<br>D0 0.00000300 sec<br>D1 1.48689198 sec<br>D13 0.00000400 sec<br>D16 0.00020000 sec<br>IN0 0.00020800 sec<br><br>======== CHANNEL f1 ========<br>SFO1 400.1322007 MHz<br>NUC1 1H<br>P0 15.00 usec<br>P1 15.00 usec<br>PLW1 12.50300026 W<br><br>====== GRADIENT CHANNEL =====<br>GPNAM[1] SMSQ10.100<br>GPZ1 10.00 %<br>P16 1000.00 usec<br><br>F1 - Acquisition parameters<br>TD 128<br>SFO1 400.1322 MHz<br>FIDRES 75.120193 Hz<br>SW 12.015 ppm<br>FnMODE QF<br><br>F2 - Processing parameters<br>SI 1024<br>SF 400.1300631 MHz<br>WDW SINE<br>SSB 0<br>LB 0 Hz<br>GB 0<br>PC 1.40<br><br>F1 - Processing parameters<br>SI 1024<br>MC2 QF<br>SF 400.1300630 MHz<br>WDW SINE<br>SSB 0<br>LB 0 Hz<br>GB 0	F2 - Acquisition Parameters<br>Date_ 20180209<br>Time 15.48<br>INSTRUM spect<br>PROBHD 5 mm PABBO BB-<br>PULPROG cosygpqf<br>TD 2048<br>SOLVENT D2O<br>NS 1<br>DS 8<br>SWH 4807.692 Hz<br>FIDRES 2.347506 Hz<br>AQ 0.2129920 sec<br>RG 192.58<br>DW 104.000 usec<br>DE 6.50 usec<br>TE 298.1 K<br>D0 0.00000300 sec<br>D1 1.48689198 sec<br>D13 0.00000400 sec<br>D16 0.00020000 sec<br>IN0 0.00020800 sec<br><br>======== CHANNEL f1 ========<br>SFO1 400.1322007 MHz<br>NUC1 1H<br>P0 15.00 usec<br>P1 15.00 usec<br>PLW1 12.50300026 W<br><br>====== GRADIENT CHANNEL =====<br>GPNAM[1] SMSQ10.100<br>GPZ1 10.00 %<br>P16 1000.00 usec<br><br>F1 - Acquisition parameters<br>TD 128<br>SFO1 400.1322 MHz<br>FIDRES 75.120193 Hz<br>SW 12.015 ppm<br>FnMODE QF<br><br>F2 - Processing parameters<br>SI 1024<br>SF 400.1299556 MHz<br>WDW SINE<br>SSB 0<br>LB 0 Hz<br>GB 0<br>PC 1.40<br><br>F1 - Processing parameters<br>SI 1024<br>MC2 QF<br>SF 400.1299552 MHz<br>WDW SINE<br>SSB 0<br>LB 0 Hz<br>GB 0	F2 - Acquisition Parameters<br>Date_ 20180221<br>Time 16.25<br>INSTRUM spect<br>PROBHD 5 mm PABBO BB-<br>PULPROG cosygpqf<br>TD 2048<br>SOLVENT D2O<br>NS 1<br>DS 8<br>SWH 4807.692 Hz<br>FIDRES 2.347506 Hz<br>AQ 0.2129920 sec<br>RG 192.58<br>DW 104.000 usec<br>DE 6.50 usec<br>TE 298.1 K<br>D0 0.00000300 sec<br>D1 1.48689198 sec<br>D13 0.00000400 sec<br>D16 0.00020000 sec<br>IN0 0.00020800 sec<br><br>======== CHANNEL f1 ========<br>SFO1 400.1322007 MHz<br>NUC1 1H<br>P0 15.00 usec<br>P1 15.00 usec<br>PLW1 12.50300026 W<br><br>====== GRADIENT CHANNEL =====<br>GPNAM[1] SMSQ10.100<br>GPZ1 10.00 %<br>P16 1000.00 usec<br><br>F1 - Acquisition parameters<br>TD 128<br>SFO1 400.1322 MHz<br>FIDRES 75.120193 Hz<br>SW 12.015 ppm<br>FnMODE QF<br><br>F2 - Processing parameters<br>SI 1024<br>SF 400.1300000 MHz<br>WDW SINE<br>SSB 0<br>LB 0 Hz<br>GB 0<br>PC 1.40<br><br>F1 - Processing parameters<br>SI 1024<br>MC2 QF<br>SF 400.1300000 MHz<br>WDW SINE<br>SSB 0<br>LB 0 Hz<br>GB 0

HSQC			
	F2 - Acquisition Parameters	F2 - Acquisition Parameters	F2 - Acquisition Parameters
	Date_ 20171025	Date_ 20180213	Date_ 20180221
	Time 16.28	Time 23.06	Time 20.03
	INSTRUM spect	INSTRUM spect	INSTRUM spect
	PROBHD 5 mm PABBO BB-	PROBHD 5 mm PABBO BB-	PROBHD 5 mm PABBO BB-
	PULPROG hsqcetgpsisp2.2	PULPROG hsqcetgpsisp2.2	PULPROG hsqcetgpsisp2.2
	TD 2048	TD 2048	TD 2048
	SOLVENT CDCl3	SOLVENT D2O	SOLVENT D2O
	NS 2	NS 2	NS 2
	DS 16	DS 16	DS 16
	SWH 5341.880 Hz	SWH 5341.880 Hz	SWH 5341.880 Hz
	FIDRES 2.608340 Hz	FIDRES 2.608340 Hz	FIDRES 2.608340 Hz
	AQ 0.1916928 sec	AQ 0.1916928 sec	AQ 0.1916928 sec
	RG 192.58	RG 192.58	RG 192.58
	DW 93.600 usec	DW 93.600 usec	DW 93.600 usec
	DE 6.50 usec	DE 6.50 usec	DE 6.50 usec
	TE 298.3 K	TE 298.0 K	TE 298.0 K
	CNST2 145.0000000	CNST2 145.0000000	CNST2 145.0000000
	CNST17 -0.5000000	CNST17 -0.5000000	CNST17 -0.5000000
	D0 0.00000300 sec	D0 0.00000300 sec	D0 0.00000300 sec
	D1 1.50000000 sec	D1 1.50000000 sec	D1 1.50000000 sec
	D4 0.00172414 sec	D4 0.00172414 sec	D4 0.00172414 sec
	D11 0.03000000 sec	D11 0.03000000 sec	D11 0.03000000 sec
	D16 0.00020000 sec	D16 0.00020000 sec	D16 0.00020000 sec
	D24 0.00086207 sec	D24 0.00086207 sec	D24 0.00086207 sec
	IN0 0.00003000 sec	IN0 0.00003000 sec	IN0 0.00003000 sec
	======== CHANNEL f1 ========	======== CHANNEL f1 ========	======== CHANNEL f1 ========
	SFO1 400.1324057 MHz	SFO1 400.1324057 MHz	SFO1 400.1324057 MHz
	NUC1 1H	NUC1 1H	NUC1 1H
	P1 15.00 usec	P1 15.00 usec	P1 15.00 usec
	P2 30.00 usec	P2 30.00 usec	P2 30.00 usec
	P28 1000.00 usec	P28 1000.00 usec	P28 1000.00 usec
	PLW1 12.50300026 W	PLW1 12.50300026 W	PLW1 12.50300026 W
	======== CHANNEL f2 ========	======== CHANNEL f2 ========	======== CHANNEL f2 ========
	SFO2 100.6202713 MHz	SFO2 100.6202713 MHz	SFO2 100.6202713 MHz
	NUC2 13C	NUC2 13C	NUC2 13C
	CPDPRG[2 bi_p5m4sp_4sp.2	CPDPRG[2 bi_p5m4sp_4sp.2	CPDPRG[2 bi_p5m4sp_4sp.2
	P3 9.80 usec	P3 9.80 usec	P3 9.80 usec
	P14 500.00 usec	P14 500.00 usec	P14 500.00 usec
	P24 2000.00 usec	P24 2000.00 usec	P24 2000.00 usec
	P63 1500.00 usec	P63 1500.00 usec	P63 1500.00 usec
	PLW0 0 W	PLW0 0 W	PLW0 0 W
	PLW2 60.95399857 W	PLW2 60.95399857 W	PLW2 60.95399857 W
	PLW12 0.91469002 W	PLW12 0.91469002 W	PLW12 0.91469002 W
	SPNAM[3] Crp60,0.5,20.1	SPNAM[3] Crp60,0.5,20.1	SPNAM[3] Crp60,0.5,20.1
	SPOAL3 0.500	SPOAL3 0.500	SPOAL3 0.500
	SPOFFS3 0 Hz	SPOFFS3 0 Hz	SPOFFS3 0 Hz
	SPW3 8.94419956 W	SPW3 8.94419956 W	SPW3 8.94419956 W
	SPNAM[7] Crp60comp.4	SPNAM[7] Crp60comp.4	SPNAM[7] Crp60comp.4
	SPOAL7 0.500	SPOAL7 0.500	SPOAL7 0.500
	SPOFFS7 0 Hz	SPOFFS7 0 Hz	SPOFFS7 0 Hz
	SPW7 8.94419956 W	SPW7 8.94419956 W	SPW7 8.94419956 W
	SPNAM[14 Crp32,1.5,20.2	SPNAM[14 Crp32,1.5,20.2	SPNAM[14 Crp32,1.5,20.2
	SPOAL14 0.500	SPOAL14 0.500	SPOAL14 0.500
	SPOFFS14 0 Hz	SPOFFS14 0 Hz	SPOFFS14 0 Hz
	SPW14 3.81620002 W	SPW14 3.81620002 W	SPW14 3.81620002 W
	SPNAM[31 Crp32,1.5,20.2	SPNAM[31 Crp32,1.5,20.2	SPNAM[31 Crp32,1.5,20.2
	SPOAL31 0.500	SPOAL31 0.500	SPOAL31 0.500
	SPOFFS31 0 Hz	SPOFFS31 0 Hz	SPOFFS31 0 Hz
	SPW31 0.95405000 W	SPW31 0.95405000 W	SPW31 0.95405000 W
	====== GRADIENT CHANNEL =====	====== GRADIENT CHANNEL =====	====== GRADIENT CHANNEL =====
	GPNAM[1] SMSQ10.100	GPNAM[1] SMSQ10.100	GPNAM[1] SMSQ10.100
	GPNAM[2] SMSQ10.100	GPNAM[2] SMSQ10.100	GPNAM[2] SMSQ10.100
	GPNAM[3] SMSQ10.100	GPNAM[3] SMSQ10.100	GPNAM[3] SMSQ10.100
	GPNAM[4] SMSQ10.100	GPNAM[4] SMSQ10.100	GPNAM[4] SMSQ10.100
	GPZ1 80.00 %	GPZ1 80.00 %	GPZ1 80.00 %
	GPZ2 20.10 %	GPZ2 20.10 %	GPZ2 20.10 %
	GPZ3 11.00 %	GPZ3 11.00 %	GPZ3 11.00 %
	GPZ4 -5.00 %	GPZ4 -5.00 %	GPZ4 -5.00 %
	P16 1000.00 usec	P16 1000.00 usec	P16 1000.00 usec
	P19 600.00 usec	P19 600.00 usec	P19 600.00 usec
	F1 - Acquisition parameters	F1 - Acquisition parameters	F1 - Acquisition parameters
	TD 256	TD 256	TD 256
	SFO1 100.6203 MHz	SFO1 100.6203 MHz	SFO1 100.6203 MHz
	FIDRES 130.208328 Hz	FIDRES 130.208328 Hz	FIDRES 130.208328 Hz
	SW 165.639 ppm	SW 165.639 ppm	SW 165.639 ppm
	FnMODE Echo-Antiecho	FnMODE Echo-Antiecho	FnMODE Echo-Antiecho
	F2 - Processing parameters	F2 - Processing parameters	F2 - Processing parameters
	SI 1024	SI 1024	SI 1024
	SF 400.1300624 MHz	SF 400.1299619 MHz	SF 400.1300000 MHz
	WDW QSINE	WDW QSINE	WDW QSINE
	SSB 2	SSB 2	SSB 2
	LB 0 Hz	LB 0 Hz	LB 0 Hz
	GB 0	GB 0	GB 0
	PC 1.40	PC 1.40	PC 1.40
	F1 - Processing parameters	F1 - Processing parameters	F1 - Processing parameters
	SI 1024	SI 1024	SI 1024
	MC2 echo-antiecho	MC2 echo-antiecho	MC2 echo-antiecho
	SF 100.6127604 MHz	SF 100.6127690 MHz	SF 100.6127690 MHz
	WDW QSINE	WDW QSINE	WDW QSINE
	SSB 2	SSB 2	SSB 2
	LB 0 Hz	LB 0 Hz	LB 0 Hz
	GB 0	GB 0	GB 0

HMBC	Parameter			
	F2 - Acquisition Parameters			
	Date_	20171025	20180209	20180221
	Time	16.11	15.54	16.31
	INSTRUM	spect	spect	spect
	PROBHD	5 mm PABBO BB-	5 mm PABBO BB-	5 mm PABBO BB-
	PULPROG	hmbcgpl2ndqf	hmbcgpl2ndqf	hmbcgpl2ndqf
	TD	2048	2048	2048
	SOLVENT	CDCl3	D2O	D2O
	NS	4	4	4
	DS	16	16	16
	SWH	4807.692 Hz	4807.692 Hz	4807.692 Hz
	FIDRES	2.347506 Hz	2.347506 Hz	2.347506 Hz
	AQ	0.2129920 sec	0.2129920 sec	0.2129920 sec
	RG	192.58	192.58	192.58
	DW	104.000 usec	104.000 usec	104.000 usec
	DE	6.50 usec	6.50 usec	6.50 usec
	TE	298.2 K	298.1 K	298.1 K
	CNST6	125.0000000	125.0000000	125.0000000
	CNST7	165.0000000	165.0000000	165.0000000
	CNST13	7.5000000	7.5000000	7.5000000
	D0	0.00000300 sec	0.00000300 sec	0.00000300 sec
	D1	1.50000000 sec	1.50000000 sec	1.50000000 sec
	D6	0.06666667 sec	0.06666667 sec	0.06666667 sec
	D16	0.00020000 sec	0.00020000 sec	0.00020000 sec
	IN0	0.00002240 sec	0.00002240 sec	0.00002240 sec
	======== CHANNEL f1 ========			
	SFO1	400.1322007 MHz	400.1322007 MHz	400.1322007 MHz
	NUC1	1H	1H	1H
	P1	15.00 usec	15.00 usec	15.00 usec
	P2	30.00 usec	30.00 usec	30.00 usec
	PLW1	12.50300026 W	12.50300026 W	12.50300026 W
	======== CHANNEL f2 ========			
	SFO2	100.6228119 MHz	100.6228119 MHz	100.6228119 MHz
	NUC2	13C	13C	13C
	P3	9.80 usec	9.80 usec	9.80 usec
	PLW2	60.95399857 W	60.95399857 W	60.95399857 W
	====== GRADIENT CHANNEL =====			
	GPNAM[1]	SMSQ10.100	SMSQ10.100	SMSQ10.100
	GPNAM[2]	SMSQ10.100	SMSQ10.100	SMSQ10.100
	GPNAM[3]	SMSQ10.100	SMSQ10.100	SMSQ10.100
	GPNAM[4]	SMSQ10.100	SMSQ10.100	SMSQ10.100
	GPNAM[5]	SMSQ10.100	SMSQ10.100	SMSQ10.100
	GPNAM[6]	SMSQ10.100	SMSQ10.100	SMSQ10.100
	GPZ1	50.00 %	50.00 %	50.00 %
	GPZ2	30.00 %	30.00 %	30.00 %
	GPZ3	40.10 %	40.10 %	40.10 %
	GPZ4	15.00 %	15.00 %	15.00 %
	GPZ5	-10.00 %	-10.00 %	-10.00 %
	GPZ6	-5.00 %	-5.00 %	-5.00 %
	P16	1000.00 usec	1000.00 usec	1000.00 usec
	F1 - Acquisition parameters			
	TD	128	128	128
	SFO1	100.6228 MHz	100.6228 MHz	100.6228 MHz
	FIDRES	348.772308 Hz	348.772308 Hz	348.772308 Hz
	SW	221.833 ppm	221.833 ppm	221.833 ppm
	FnMODE	QF	QF	QF
	F2 - Processing parameters			
	SI	2048	2048	2048
	SF	400.1300628 MHz	400.1299573 MHz	400.1299618 MHz
	WDW	SINE	SINE	SINE
	SSB	0	0	0
	LB	0 Hz	0 Hz	0 Hz
	GB	0	0	0
	PC	1.40	1.40	1.40
	F1 - Processing parameters			
	SI	1024	1024	1024
	MC2	QF	QF	QF
	SF	100.6127463 MHz	100.6127310 MHz	100.6127690 MHz
	WDW	SINE	SINE	SINE
	SSB	0	0	0
	LB	0 Hz	0 Hz	0 Hz
	GB	0	0	0

	meta 2	meta 3
^{1}H	F2 - Acquisition Parameters<br>Date_ 20180405<br>Time 14.40<br>INSTRUM spect<br>PROBHD 5 mm PABBO BB-<br>PULPROG zg30<br>TD 65536<br>SOLVENT CDCl3<br>NS 16<br>DS 2<br>SWH 8012.820 Hz<br>FIDRES 0.122266 Hz<br>AQ 4.0894465 sec<br>RG 33.39<br>DW 62.400 usec<br>DE 6.50 usec<br>TE 298.2 K<br>D1 1.00000000 sec<br>TD0 1<br><br>======== CHANNEL f1 ========<br>SFO1 400.1324710 MHz<br>NUC1 1H<br>P1 15.00 usec<br>PLW1 12.50300026 W<br><br>F2 - Processing parameters<br>SI 65536<br>SF 400.1300095 MHz<br>WDW EM<br>SSB 0<br>LB 0.30 Hz<br>GB 0<br>PC 1.00	F2 - Acquisition Parameters<br>Date_ 20180410<br>Time 5.47<br>INSTRUM spect<br>PROBHD 5 mm PABBO BB-<br>PULPROG zg<br>TD 48076<br>SOLVENT D2O<br>NS 8<br>DS 2<br>SWH 6002.401 Hz<br>FIDRES 0.124852 Hz<br>AQ 4.0047307 sec<br>RG 17.75<br>DW 83.300 usec<br>DE 16.70 usec<br>TE 298.1 K<br>D1 5.00000000 sec<br>TD0 1<br><br>======== CHANNEL f1 ========<br>SFO1 400.1320007 MHz<br>NUC1 1H<br>P1 15.00 usec<br>PLW1 12.50300026 W<br><br>F2 - Processing parameters<br>SI 32768<br>SF 400.1299714 MHz<br>WDW EM<br>SSB 0<br>LB 0.30 Hz<br>GB 0<br>PC 1.00

**DEPT**	F2 - Acquisition Parameters<br>Date_ 20180405<br>Time 15.05<br>INSTRUM spect<br>PROBHD 5 mm PABBO BB-<br>PULPROG deptqgpsp<br>TD 65536<br>SOLVENT CDCl3<br>NS 256<br>DS 4<br>SWH 22058.824 Hz<br>FIDRES 0.336591 Hz<br>AQ 1.4854827 sec<br>RG 192.58<br>DW 22.667 usec<br>DE 6.50 usec<br>TE 298.1 K<br>CNST2 145.0000000<br>CNST12 1.5000000<br>D1 2.00000000 sec<br>D2 0.00344828 sec<br>D12 0.00002000 sec<br>D16 0.00020000 sec<br>TD0 1<br><br>======== CHANNEL f1 ========<br>SFO1 100.6223258 MHz<br>NUC1 13C<br>P1 9.80 usec<br>P13 2000.00 usec<br>PLW0 0 W<br>PLW1 60.95399857 W<br>SPNAM[5] Crp60comp.4<br>SPOAL5 0.500<br>SPOFFS5 0 Hz<br>SPW5 8.94419956 W<br><br>======== CHANNEL f2 ========<br>SFO2 400.1316005 MHz<br>NUC2 1H<br>CPDPRG[2 waltz16<br>P0 22.50 usec<br>P3 15.00 usec<br>P4 30.00 usec<br>PCPD2 90.00 usec<br>PLW2 12.50300026 W<br>PLW12 0.34731001 W<br><br>====== GRADIENT CHANNEL =====<br>GPNAM[1] SMSQ10.32<br>GPNAM[2] SMSQ10.32<br>GPNAM[3] SMSQ10.32<br>GPZ1 31.00 %<br>GPZ2 31.00 %<br>GPZ3 31.00 %<br>P16 1000.00 usec<br><br>F2 - Processing parameters<br>SI 65536<br>SF 100.6127625 MHz<br>WDW EM<br>SSB 0<br>LB 1.00 Hz<br>GB 0<br>PC 1.40	F2 - Acquisition Parameters<br>Date_ 20180410<br>Time 6.04<br>INSTRUM spect<br>PROBHD 5 mm PABBO BB-<br>PULPROG deptqgpsp<br>TD 65536<br>SOLVENT D2O<br>NS 256<br>DS 4<br>SWH 22058.824 Hz<br>FIDRES 0.336591 Hz<br>AQ 1.4854827 sec<br>RG 192.58<br>DW 22.667 usec<br>DE 6.50 usec<br>TE 298.2 K<br>CNST2 145.0000000<br>CNST12 1.5000000<br>D1 2.00000000 sec<br>D2 0.00344828 sec<br>D12 0.00002000 sec<br>D16 0.00020000 sec<br>TD0 1<br><br>======== CHANNEL f1 ========<br>SFO1 100.6223258 MHz<br>NUC1 13C<br>P1 9.80 usec<br>P13 2000.00 usec<br>PLW0 0 W<br>PLW1 60.95399857 W<br>SPNAM[5] Crp60comp.4<br>SPOAL5 0.500<br>SPOFFS5 0 Hz<br>SPW5 8.94419956 W<br><br>======== CHANNEL f2 ========<br>SFO2 400.1316005 MHz<br>NUC2 1H<br>CPDPRG[2 waltz16<br>P0 22.50 usec<br>P3 15.00 usec<br>P4 30.00 usec<br>PCPD2 90.00 usec<br>PLW2 12.50300026 W<br>PLW12 0.34731001 W<br><br>====== GRADIENT CHANNEL =====<br>GPNAM[1] SMSQ10.32<br>GPNAM[2] SMSQ10.32<br>GPNAM[3] SMSQ10.32<br>GPZ1 31.00 %<br>GPZ2 31.00 %<br>GPZ3 31.00 %<br>P16 1000.00 usec<br><br>F2 - Processing parameters<br>SI 65536<br>SF 100.6127690 MHz<br>WDW EM<br>SSB 0<br>LB 1.00 Hz<br>GB 0<br>PC 1.40

^{13}C	F2 - Acquisition Parameters<br>Date_ 20180408<br>Time 7.42<br>INSTRUM spect<br>PROBHD 5 mm PABBO BB-<br>PULPROG zgpg30<br>TD 65536<br>SOLVENT CDCl3<br>NS 3000<br>DS 4<br>SWH 22058.824 Hz<br>FIDRES 0.336591 Hz<br>AQ 1.4854827 sec<br>RG 192.58<br>DW 22.667 usec<br>DE 6.50 usec<br>TE 298.1 K<br>D1 2.00000000 sec<br>D11 0.03000000 sec<br>TD0 1<br><br>======== CHANNEL f1 ========<br>SFO1 100.6223253 MHz<br>NUC1 13C<br>P1 9.80 usec<br>PLW1 60.95399857 W<br><br>======== CHANNEL f2 ========<br>SFO2 400.1316005 MHz<br>NUC2 1H<br>CPDPRG[2 waltz16<br>PCPD2 90.00 usec<br>PLW2 12.50300026 W<br>PLW12 0.34731001 W<br>PLW13 0.28132001 W<br><br>F2 - Processing parameters<br>SI 65536<br>SF 100.6127636 MHz<br>WDW EM<br>SSB 0<br>LB 1.00 Hz<br>GB 0<br>PC 1.40	F2 - Acquisition Parameters<br>Date_ 20180411<br>Time 0.04<br>INSTRUM spect<br>PROBHD 5 mm PABBO BB-<br>PULPROG zgpg30<br>TD 65536<br>SOLVENT D2O<br>NS 3000<br>DS 4<br>SWH 22058.824 Hz<br>FIDRES 0.336591 Hz<br>AQ 1.4854827 sec<br>RG 192.58<br>DW 22.667 usec<br>DE 6.50 usec<br>TE 298.2 K<br>D1 2.00000000 sec<br>D11 0.03000000 sec<br>TD0 1<br><br>======== CHANNEL f1 ========<br>SFO1 100.6223253 MHz<br>NUC1 13C<br>P1 9.80 usec<br>PLW1 60.95399857 W<br><br>======== CHANNEL f2 ========<br>SFO2 400.1316005 MHz<br>NUC2 1H<br>CPDPRG[2 waltz16<br>PCPD2 90.00 usec<br>PLW2 12.50300026 W<br>PLW12 0.34731001 W<br>PLW13 0.28132001 W<br><br>F2 - Processing parameters<br>SI 65536<br>SF 100.6127690 MHz<br>WDW EM<br>SSB 0<br>LB 1.00 Hz<br>GB 0<br>PC 1.40
^{11}B	F2 - Acquisition Parameters<br>Date_ 20180406<br>Time 7.57<br>INSTRUM spect<br>PROBHD 5 mm PABBO BB-<br>PULPROG zg<br>TD 65536<br>SOLVENT CDCl3<br>NS 128<br>DS 4<br>SWH 25510.203 Hz<br>FIDRES 0.389255 Hz<br>AQ 1.2845056 sec<br>RG 192.58<br>DW 19.600 usec<br>DE 6.50 usec<br>TE 298.2 K<br>D1 1.00000000 sec<br>TD0 1<br><br>======== CHANNEL f1 ========<br>SFO1 128.3776052 MHz<br>NUC1 11B<br>P1 17.05 usec<br>PLW1 11.69499969 W<br><br>F2 - Processing parameters<br>SI 32768<br>SF 128.3776052 MHz<br>WDW no<br>SSB 0<br>LB 0 Hz<br>GB 0<br>PC 1.40	F2 - Acquisition Parameters<br>Date_ 20180409<br>Time 14.58<br>INSTRUM spect<br>PROBHD 5 mm PABBO BB-<br>PULPROG zg<br>TD 65536<br>SOLVENT D2O<br>NS 128<br>DS 4<br>SWH 25510.203 Hz<br>FIDRES 0.389255 Hz<br>AQ 1.2845056 sec<br>RG 192.58<br>DW 19.600 usec<br>DE 6.50 usec<br>TE 298.2 K<br>D1 1.00000000 sec<br>TD0 1<br><br>======== CHANNEL f1 ========<br>SFO1 128.3776052 MHz<br>NUC1 11B<br>P1 17.05 usec<br>PLW1 11.69499969 W<br><br>F2 - Processing parameters<br>SI 32768<br>SF 128.3776052 MHz<br>WDW no<br>SSB 0<br>LB 0 Hz<br>GB 0<br>PC 1.40

**COSY**	F2 - Acquisition Parameters<br>Date_ 20180405<br>Time 15.07<br>INSTRUM spect<br>PROBHD 5 mm PABBO BB-<br>PULPROG cosygpqf<br>TD 2048<br>SOLVENT CDCl3<br>NS 1<br>DS 8<br>SWH 4807.692 Hz<br>FIDRES 2.347506 Hz<br>AQ 0.2129920 sec<br>RG 56.54<br>DW 104.000 usec<br>DE 6.50 usec<br>TE 298.0 K<br>D0 0.00000300 sec<br>D1 1.48689198 sec<br>D13 0.00000400 sec<br>D16 0.00020000 sec<br>IN0 0.00020800 sec<br><br>======== CHANNEL f1 ========<br>SFO1 400.1322007 MHz<br>NUC1 1H<br>P0 15.00 usec<br>P1 15.00 usec<br>PLW1 12.50300026 W<br><br>====== GRADIENT CHANNEL =====<br>GPNAM[1] SMSQ10.100<br>GPZ1 10.00 %<br>P16 1000.00 usec<br><br>F1 - Acquisition parameters<br>TD 128<br>SFO1 400.1322 MHz<br>FIDRES 75.120193 Hz<br>SW 12.015 ppm<br>FnMODE QF<br><br>F2 - Processing parameters<br>SI 1024<br>SF 400.1300078 MHz<br>WDW SINE<br>SSB 0<br>LB 0 Hz<br>GB 0<br>PC 1.40<br><br>F1 - Processing parameters<br>SI 1024<br>MC2 QF<br>SF 400.1300092 MHz<br>WDW SINE<br>SSB 0<br>LB 0 Hz<br>GB 0	F2 - Acquisition Parameters<br>Date_ 20180410<br>Time 6.07<br>INSTRUM spect<br>PROBHD 5 mm PABBO BB-<br>PULPROG cosygpqf<br>TD 2048<br>SOLVENT D2O<br>NS 1<br>DS 8<br>SWH 4807.692 Hz<br>FIDRES 2.347506 Hz<br>AQ 0.2129920 sec<br>RG 56.54<br>DW 104.000 usec<br>DE 6.50 usec<br>TE 298.0 K<br>D0 0.00000300 sec<br>D1 1.48689198 sec<br>D13 0.00000400 sec<br>D16 0.00020000 sec<br>IN0 0.00020800 sec<br><br>======== CHANNEL f1 ========<br>SFO1 400.1322007 MHz<br>NUC1 1H<br>P0 15.00 usec<br>P1 15.00 usec<br>PLW1 12.50300026 W<br><br>====== GRADIENT CHANNEL =====<br>GPNAM[1] SMSQ10.100<br>GPZ1 10.00 %<br>P16 1000.00 usec<br><br>F1 - Acquisition parameters<br>TD 128<br>SFO1 400.1322 MHz<br>FIDRES 75.120193 Hz<br>SW 12.015 ppm<br>FnMODE QF<br><br>F2 - Processing parameters<br>SI 1024<br>SF 400.1299700 MHz<br>WDW SINE<br>SSB 0<br>LB 0 Hz<br>GB 0<br>PC 1.40<br><br>F1 - Processing parameters<br>SI 1024<br>MC2 QF<br>SF 400.1299710 MHz<br>WDW SINE<br>SSB 0<br>LB 0 Hz<br>GB 0

**HSQC**	F2 - Acquisition Parameters	F2 - Acquisition Parameters
	Date_ 20180408	Date_ 20180411
	Time 7.45	Time 0.06
	INSTRUM spect	INSTRUM spect
	PROBHD 5 mm PABBO BB-	PROBHD 5 mm PABBO BB-
	PULPROG hsqcetgpsisp2.2	PULPROG hsqcetgpsisp2.2
	TD 2048	TD 2048
	SOLVENT CDCl3	SOLVENT D2O
	NS 2	NS 2
	DS 16	DS 16
	SWH 5341.880 Hz	SWH 5341.880 Hz
	FIDRES 2.608340 Hz	FIDRES 2.608340 Hz
	AQ 0.1916928 sec	AQ 0.1916928 sec
	RG 192.58	RG 192.58
	DW 93.600 usec	DW 93.600 usec
	DE 6.50 usec	DE 6.50 usec
	TE 298.0 K	TE 298.0 K
	CNST2 145.0000000	CNST2 145.0000000
	CNST17 -0.5000000	CNST17 -0.5000000
	D0 0.00000300 sec	D0 0.00000300 sec
	D1 1.50000000 sec	D1 1.50000000 sec
	D4 0.00172414 sec	D4 0.00172414 sec
	D11 0.03000000 sec	D11 0.03000000 sec
	D16 0.00020000 sec	D16 0.00020000 sec
	D24 0.00086207 sec	D24 0.00086207 sec
	IN0 0.00003000 sec	IN0 0.00003000 sec
	======== CHANNEL f1 ========	======== CHANNEL f1 ========
	SFO1 400.1324057 MHz	SFO1 400.1324057 MHz
	NUC1 1H	NUC1 1H
	P1 15.00 usec	P1 15.00 usec
	P2 30.00 usec	P2 30.00 usec
	P28 1000.00 usec	P28 1000.00 usec
	PLW1 12.50300026 W	PLW1 12.50300026 W
	======== CHANNEL f2 ========	======== CHANNEL f2 ========
	SFO2 100.6202713 MHz	SFO2 100.6202713 MHz
	NUC2 13C	NUC2 13C
	CPDPRG[2 bi_p5m4sp_4sp.2	CPDPRG[2 bi_p5m4sp_4sp.2
	P3 9.80 usec	P3 9.80 usec
	P14 500.00 usec	P14 500.00 usec
	P24 2000.00 usec	P24 2000.00 usec
	P63 1500.00 usec	P63 1500.00 usec
	PLW0 0 W	PLW0 0 W
	PLW2 60.95399857 W	PLW2 60.95399857 W
	PLW12 0.91469002 W	PLW12 0.91469002 W
	SPNAM[3] Crp60,0.5,20.1	SPNAM[3] Crp60,0.5,20.1
	SPOAL3 0.500	SPOAL3 0.500
	SPOFFS3 0 Hz	SPOFFS3 0 Hz
	SPW3 8.94419956 W	SPW3 8.94419956 W
	SPNAM[7] Crp60comp.4	SPNAM[7] Crp60comp.4
	SPOAL7 0.500	SPOAL7 0.500
	SPOFFS7 0 Hz	SPOFFS7 0 Hz
	SPW7 8.94419956 W	SPW7 8.94419956 W
	SPNAM[14 Crp32,1.5,20.2	SPNAM[14 Crp32,1.5,20.2
	SPOAL14 0.500	SPOAL14 0.500
	SPOFFS14 0 Hz	SPOFFS14 0 Hz
	SPW14 3.81620002 W	SPW14 3.81620002 W
	SPNAM[31 Crp32,1.5,20.2	SPNAM[31 Crp32,1.5,20.2
	SPOAL31 0.500	SPOAL31 0.500
	SPOFFS31 0 Hz	SPOFFS31 0 Hz
	SPW31 0.95405000 W	SPW31 0.95405000 W
	====== GRADIENT CHANNEL =====	====== GRADIENT CHANNEL =====
	GPNAM[1] SMSQ10.100	GPNAM[1] SMSQ10.100
	GPNAM[2] SMSQ10.100	GPNAM[2] SMSQ10.100
	GPNAM[3] SMSQ10.100	GPNAM[3] SMSQ10.100
	GPNAM[4] SMSQ10.100	GPNAM[4] SMSQ10.100
	GPZ1 80.00 %	GPZ1 80.00 %
	GPZ2 20.10 %	GPZ2 20.10 %
	GPZ3 11.00 %	GPZ3 11.00 %
	GPZ4 -5.00 %	GPZ4 -5.00 %
	P16 1000.00 usec	P16 1000.00 usec
	P19 600.00 usec	P19 600.00 usec
	F1 - Acquisition parameters	F1 - Acquisition parameters
	TD 256	TD 256
	SFO1 100.6203 MHz	SFO1 100.6203 MHz
	FIDRES 130.208328 Hz	FIDRES 130.208328 Hz
	SW 165.639 ppm	SW 165.639 ppm
	FnMODE Echo-Antiecho	FnMODE Echo-Antiecho
	F2 - Processing parameters	F2 - Processing parameters
	SI 1024	SI 1024
	SF 400.1300069 MHz	SF 400.1299725 MHz
	WDW QSINE	WDW QSINE
	SSB 2	SSB 2
	LB 0 Hz	LB 0 Hz
	GB 0	GB 0
	PC 1.40	PC 1.40
	F1 - Processing parameters	F1 - Processing parameters
	SI 1024	SI 1024
	MC2 echo-antiecho	MC2 echo-antiecho
	SF 100.6127690 MHz	SF 100.6127690 MHz
	WDW QSINE	WDW QSINE
	SSB 2	SSB 2
	LB 0 Hz	LB 0 Hz
	GB 0	GB 0

**HMBC**	F2 - Acquisition Parameters	F2 - Acquisition Parameters
	Date_ 20180405	Date_ 20180410
	Time 15.20	Time 6.12
	INSTRUM spect	INSTRUM spect
	PROBHD 5 mm PABBO BB-	PROBHD 5 mm PABBO BB-
	PULPROG hmbcgpl2ndqf	PULPROG hmbcgpl2ndqf
	TD 2048	TD 2048
	SOLVENT CDCl3	SOLVENT D2O
	NS 4	NS 4
	DS 16	DS 16
	SWH 4807.692 Hz	SWH 4807.692 Hz
	FIDRES 2.347506 Hz	FIDRES 2.347506 Hz
	AQ 0.2129920 sec	AQ 0.2129920 sec
	RG 192.58	RG 192.58
	DW 104.000 usec	DW 104.000 usec
	DE 6.50 usec	DE 6.50 usec
	TE 298.2 K	TE 298.1 K
	CNST6 125.0000000	CNST6 125.0000000
	CNST7 165.0000000	CNST7 165.0000000
	CNST13 7.5000000	CNST13 7.5000000
	D0 0.00000300 sec	D0 0.00000300 sec
	D1 1.50000000 sec	D1 1.50000000 sec
	D6 0.06666667 sec	D6 0.06666667 sec
	D16 0.00020000 sec	D16 0.00020000 sec
	IN0 0.00002240 sec	IN0 0.00002240 sec
	======== CHANNEL f1 ========	======== CHANNEL f1 ========
	SFO1 400.1322007 MHz	SFO1 400.1322007 MHz
	NUC1 1H	NUC1 1H
	P1 15.00 usec	P1 15.00 usec
	P2 30.00 usec	P2 30.00 usec
	PLW1 12.50300026 W	PLW1 12.50300026 W
	======== CHANNEL f2 ========	======== CHANNEL f2 ========
	SFO2 100.6228119 MHz	SFO2 100.6228119 MHz
	NUC2 13C	NUC2 13C
	P3 9.80 usec	P3 9.80 usec
	PLW2 60.95399857 W	PLW2 60.95399857 W
	====== GRADIENT CHANNEL =====	====== GRADIENT CHANNEL =====
	GPNAM[1] SMSQ10.100	GPNAM[1] SMSQ10.100
	GPNAM[2] SMSQ10.100	GPNAM[2] SMSQ10.100
	GPNAM[3] SMSQ10.100	GPNAM[3] SMSQ10.100
	GPNAM[4] SMSQ10.100	GPNAM[4] SMSQ10.100
	GPNAM[5] SMSQ10.100	GPNAM[5] SMSQ10.100
	GPNAM[6] SMSQ10.100	GPNAM[6] SMSQ10.100
	GPZ1 50.00 %	GPZ1 50.00 %
	GPZ2 30.00 %	GPZ2 30.00 %
	GPZ3 40.10 %	GPZ3 40.10 %
	GPZ4 15.00 %	GPZ4 15.00 %
	GPZ5 -10.00 %	GPZ5 -10.00 %
	GPZ6 -5.00 %	GPZ6 -5.00 %
	P16 1000.00 usec	P16 1000.00 usec
	F1 - Acquisition parameters	F1 - Acquisition parameters
	TD 128	TD 128
	SFO1 100.6228 MHz	SFO1 100.6228 MHz
	FIDRES 348.772308 Hz	FIDRES 348.772308 Hz
	SW 221.833 ppm	SW 221.833 ppm
	FnMODE QF	FnMODE QF
	F2 - Processing parameters	F2 - Processing parameters
	SI 2048	SI 2048
	SF 400.1300104 MHz	SF 400.1299701 MHz
	WDW SINE	WDW SINE
	SSB 0	SSB 0
	LB 0 Hz	LB 0 Hz
	GB 0	GB 0
	PC 1.40	PC 1.40
	F1 - Processing parameters	F1 - Processing parameters
	SI 1024	SI 1024
	MC2 QF	MC2 QF
	SF 100.6127690 MHz	SF 100.6127690 MHz
	WDW SINE	WDW SINE
	SSB 0	SSB 0
	LB 0 Hz	LB 0 Hz
	GB 0	GB 0

	meta 4	meta 5
^{1}H	F2 - Acquisition Parameters<br>Date_ 20180507<br>Time 16.31<br>INSTRUM spect<br>PROBHD 5 mm PABBO BB-<br>PULPROG zg30<br>TD 65536<br>SOLVENT MeOD<br>NS 16<br>DS 2<br>SWH 8012.820 Hz<br>FIDRES 0.122266 Hz<br>AQ 4.0894465 sec<br>RG 20.83<br>DW 62.400 usec<br>DE 6.50 usec<br>TE 298.1 K<br>D1 1.00000000 sec<br>TD0 1<br><br>======== CHANNEL f1 ========<br>SFO1 400.1324710 MHz<br>NUC1 1H<br>P1 15.00 usec<br>PLW1 12.50300026 W<br><br>F2 - Processing parameters<br>SI 65536<br>SF 400.1300859 MHz<br>WDW EM<br>SSB 0<br>LB 0.30 Hz<br>GB 0<br>PC 1.00	F2 - Acquisition Parameters<br>Date_ 20171006<br>Time 11.40<br>INSTRUM spect<br>PROBHD 5 mm PABBO BB-<br>PULPROG zg<br>TD 48076<br>SOLVENT MeOD<br>NS 8<br>DS 2<br>SWH 6002.401 Hz<br>FIDRES 0.124852 Hz<br>AQ 4.0047307 sec<br>RG 107<br>DW 83.300 usec<br>DE 16.70 usec<br>TE 298.2 K<br>D1 5.00000000 sec<br>TD0 1<br><br>======== CHANNEL f1 ========<br>SFO1 400.1320007 MHz<br>NUC1 1H<br>P1 15.00 usec<br>PLW1 12.50300026 W<br><br>F2 - Processing parameters<br>SI 32768<br>SF 400.1300076 MHz<br>WDW EM<br>SSB 0<br>LB 0.30 Hz<br>GB 0<br>PC 1.00

^{13}C	F2 - Acquisition Parameters	F2 - Acquisition Parameters
	Date_ 20180510	Date_ 20171007
	Time 7.11	Time 5.10
	INSTRUM spect	INSTRUM spect
	PROBHD 5 mm PABBO BB-	PROBHD 5 mm PABBO BB-
	PULPROG zgpg30	PULPROG zgpg30
	TD 65536	TD 65536
	SOLVENT MeOD	SOLVENT MeOD
	NS 3000	NS 4000
	DS 4	DS 4
	SWH 22058.824 Hz	SWH 22058.824 Hz
	FIDRES 0.336591 Hz	FIDRES 0.336591 Hz
	AQ 1.4854827 sec	AQ 1.4854827 sec
	RG 42.64	RG 192.58
	DW 22.667 usec	DW 22.667 usec
	DE 6.50 usec	DE 6.50 usec
	TE 298.2 K	TE 298.2 K
	D1 2.00000000 sec	D1 2.00000000 sec
	D11 0.03000000 sec	D11 0.03000000 sec
	TD0 1	TD0 1
	======== CHANNEL f1 ========	======== CHANNEL f1 ========
	SFO1 100.6223253 MHz	SFO1 100.6223253 MHz
	NUC1 13C	NUC1 13C
	P1 9.80 usec	P1 9.80 usec
	PLW1 60.95399857 W	PLW1 60.95399857 W
	======== CHANNEL f2 ========	======== CHANNEL f2 ========
	SFO2 400.1316005 MHz	SFO2 400.1316005 MHz
	NUC2 1H	NUC2 1H
	CPDPRG[2 waltz16	CPDPRG[2 waltz16
	PCPD2 90.00 usec	PCPD2 90.00 usec
	PLW2 12.50300026 W	PLW2 12.50300026 W
	PLW12 0.34731001 W	PLW12 0.34731001 W
	PLW13 0.28132001 W	PLW13 0.28132001 W
	F2 - Processing parameters	F2 - Processing parameters
	SI 65536	SI 65536
	SF 100.6126421 MHz	SF 100.6126280 MHz
	WDW EM	WDW EM
	SSB 0	SSB 0
	LB 1.00 Hz	LB 1.00 Hz
	GB 0	GB 0
	PC 1.40	PC 1.40

**DEPT**	F2 - Acquisition Parameters	F2 - Acquisition Parameters
	Date_ 20180509	Date_ 20171006
	Time 15.44	Time 12.19
	INSTRUM spect	INSTRUM spect
	PROBHD 5 mm PABBO BB-	PROBHD 5 mm PABBO BB-
	PULPROG deptqgpsp	PULPROG deptqgpsp
	TD 65536	TD 65536
	SOLVENT MeOD	SOLVENT MeOD
	NS 256	NS 256
	DS 4	DS 4
	SWH 22058.824 Hz	SWH 22058.824 Hz
	FIDRES 0.336591 Hz	FIDRES 0.336591 Hz
	AQ 1.4854827 sec	AQ 1.4854827 sec
	RG 192.58	RG 192.58
	DW 22.667 usec	DW 22.667 usec
	DE 6.50 usec	DE 6.50 usec
	TE 298.2 K	TE 298.2 K
	CNST2 145.0000000	CNST2 145.0000000
	CNST12 1.5000000	CNST12 1.5000000
	D1 2.00000000 sec	D1 2.00000000 sec
	D2 0.00344828 sec	D2 0.00344828 sec
	D12 0.00002000 sec	D12 0.00002000 sec
	D16 0.00020000 sec	D16 0.00020000 sec
	TD0 1	TD0 1
	======== CHANNEL f1 ========	======== CHANNEL f1 ========
	SFO1 100.6223258 MHz	SFO1 100.6223258 MHz
	NUC1 13C	NUC1 13C
	P1 9.80 usec	P1 9.80 usec
	P13 2000.00 usec	P13 2000.00 usec
	PLW0 0 W	PLW0 0 W
	PLW1 60.95399857 W	PLW1 60.95399857 W
	SPNAM[5] Crp60comp.4	SPNAM[5] Crp60comp.4
	SPOAL5 0.500	SPOAL5 0.500
	SPOFFS5 0 Hz	SPOFFS5 0 Hz
	SPW5 8.94419956 W	SPW5 8.94419956 W
	======== CHANNEL f2 ========	======== CHANNEL f2 ========
	SFO2 400.1316005 MHz	SFO2 400.1316005 MHz
	NUC2 1H	NUC2 1H
	CPDPRG[2 waltz16	CPDPRG[2 waltz16
	P0 22.50 usec	P0 22.50 usec
	P3 15.00 usec	P3 15.00 usec
	P4 30.00 usec	P4 30.00 usec
	PCPD2 90.00 usec	PCPD2 90.00 usec
	PLW2 12.50300026 W	PLW2 12.50300026 W
	PLW12 0.34731001 W	PLW12 0.34731001 W
	====== GRADIENT CHANNEL =====	====== GRADIENT CHANNEL =====
	GPNAM[1] SMSQ10.32	GPNAM[1] SMSQ10.32
	GPNAM[2] SMSQ10.32	GPNAM[2] SMSQ10.32
	GPNAM[3] SMSQ10.32	GPNAM[3] SMSQ10.32
	GPZ1 31.00 %	GPZ1 31.00 %
	GPZ2 31.00 %	GPZ2 31.00 %
	GPZ3 31.00 %	GPZ3 31.00 %
	P16 1000.00 usec	P16 1000.00 usec
	F2 - Processing parameters	F2 - Processing parameters
	SI 65536	SI 65536
	SF 100.6126418 MHz	SF 100.6126275 MHz
	WDW EM	WDW EM
	SSB 0	SSB 0
	LB 1.00 Hz	LB 1.00 Hz
	GB 0	GB 0
	PC 1.40	PC 1.40

^{11}B		
	F2 - Acquisition Parameters<br>Date_ 20180507<br>Time 16.37<br>INSTRUM spect<br>PROBHD 5 mm PABBO BB-<br>PULPROG zg<br>TD 65536<br>SOLVENT MeOD<br>NS 128<br>DS 4<br>SWH 25510.203 Hz<br>FIDRES 0.389255 Hz<br>AQ 1.2845056 sec<br>RG 192.58<br>DW 19.600 usec<br>DE 6.50 usec<br>TE 298.2 K<br>D1 1.00000000 sec<br>TD0 1<br><br>======== CHANNEL f1 ========<br>SFO1 128.3776052 MHz<br>NUC1 11B<br>P1 17.05 usec<br>PLW1 11.69499969 W<br><br>F2 - Processing parameters<br>SI 32768<br>SF 128.3776052 MHz<br>WDW no<br>SSB 0<br>LB 0 Hz<br>GB 0<br>PC 1.40	F2 - Acquisition Parameters<br>Date_ 20171007<br>Time 5.17<br>INSTRUM spect<br>PROBHD 5 mm PABBO BB-<br>PULPROG zg<br>TD 65536<br>SOLVENT MeOD<br>NS 128<br>DS 4<br>SWH 25510.203 Hz<br>FIDRES 0.389255 Hz<br>AQ 1.2845056 sec<br>RG 192.58<br>DW 19.600 usec<br>DE 6.50 usec<br>TE 298.1 K<br>D1 1.00000000 sec<br>TD0 1<br><br>======== CHANNEL f1 ========<br>SFO1 128.3776052 MHz<br>NUC1 11B<br>P1 17.05 usec<br>PLW1 11.69499969 W<br><br>F2 - Processing parameters<br>SI 32768<br>SF 128.3776052 MHz<br>WDW no<br>SSB 0<br>LB 0 Hz<br>GB 0<br>PC 1.40

**COSY**	F2 - Acquisition Parameters<br>Date_ 20180509<br>Time 15.46<br>INSTRUM spect<br>PROBHD 5 mm PABBO BB-<br>PULPROG cosygpqf<br>TD 2048<br>SOLVENT MeOD<br>NS 1<br>DS 8<br>SWH 4807.692 Hz<br>FIDRES 2.347506 Hz<br>AQ 0.2129920 sec<br>RG 58.62<br>DW 104.000 usec<br>DE 6.50 usec<br>TE 298.0 K<br>D0 0.00000300 sec<br>D1 1.48689198 sec<br>D13 0.00000400 sec<br>D16 0.00020000 sec<br>IN0 0.00020800 sec<br><br>======== CHANNEL f1 ========<br>SFO1 400.1322007 MHz<br>NUC1 1H<br>P0 15.00 usec<br>P1 15.00 usec<br>PLW1 12.50300026 W<br><br>====== GRADIENT CHANNEL =====<br>GPNAM[1] SMSQ10.100<br>GPZ1 10.00 %<br>P16 1000.00 usec<br><br>F1 - Acquisition parameters<br>TD 128<br>SFO1 400.1322 MHz<br>FIDRES 75.120193 Hz<br>SW 12.015 ppm<br>FnMODE QF<br><br>F2 - Processing parameters<br>SI 1024<br>SF 400.1300901 MHz<br>WDW SINE<br>SSB 0<br>LB 0 Hz<br>GB 0<br>PC 1.40<br><br>F1 - Processing parameters<br>SI 1024<br>MC2 QF<br>SF 400.1300897 MHz<br>WDW SINE<br>SSB 0<br>LB 0 Hz<br>GB 0	F2 - Acquisition Parameters<br>Date_ 20171006<br>Time 12.21<br>INSTRUM spect<br>PROBHD 5 mm PABBO BB-<br>PULPROG cosygpqf<br>TD 2048<br>SOLVENT MeOD<br>NS 1<br>DS 8<br>SWH 4807.692 Hz<br>FIDRES 2.347506 Hz<br>AQ 0.2129920 sec<br>RG 192.58<br>DW 104.000 usec<br>DE 6.50 usec<br>TE 298.0 K<br>D0 0.00000300 sec<br>D1 1.48689198 sec<br>D13 0.00000400 sec<br>D16 0.00020000 sec<br>IN0 0.00020800 sec<br><br>======== CHANNEL f1 ========<br>SFO1 400.1322007 MHz<br>NUC1 1H<br>P0 15.00 usec<br>P1 15.00 usec<br>PLW1 12.50300026 W<br><br>====== GRADIENT CHANNEL =====<br>GPNAM[1] SMSQ10.100<br>GPZ1 10.00 %<br>P16 1000.00 usec<br><br>F1 - Acquisition parameters<br>TD 128<br>SFO1 400.1322 MHz<br>FIDRES 75.120193 Hz<br>SW 12.015 ppm<br>FnMODE QF<br><br>F2 - Processing parameters<br>SI 1024<br>SF 400.1300073 MHz<br>WDW SINE<br>SSB 0<br>LB 0 Hz<br>GB 0<br>PC 1.40<br><br>F1 - Processing parameters<br>SI 1024<br>MC2 QF<br>SF 400.1300067 MHz<br>WDW SINE<br>SSB 0<br>LB 0 Hz<br>GB 0

**HSQC**	F2 - Acquisition Parameters<br>Date_ 20180510<br>Time 7.14<br>INSTRUM spect<br>PROBHD 5 mm PABBO BB-<br>PULPROG hsqcetgpsisp2.2<br>TD 2048<br>SOLVENT MeOD<br>NS 2<br>DS 16<br>SWH 5341.880 Hz<br>FIDRES 2.608340 Hz<br>AQ 0.1916928 sec<br>RG 192.58<br>DW 93.600 usec<br>DE 6.50 usec<br>TE 298.0 K<br>CNST2 145.0000000<br>CNST17 -0.5000000<br>D0 0.00000300 sec<br>D1 1.50000000 sec<br>D4 0.00172414 sec<br>D11 0.03000000 sec<br>D16 0.00020000 sec<br>D24 0.00086207 sec<br>IN0 0.00003000 sec<br><br>======== CHANNEL f1 ========<br>SFO1 400.1324057 MHz<br>NUC1 1H<br>P1 15.00 usec<br>P2 30.00 usec<br>P28 1000.00 usec<br>PLW1 12.50300026 W<br><br>======== CHANNEL f2 ========<br>SFO2 100.6202713 MHz<br>NUC2 13C<br>CPDPRG[2 bi_p5m4sp_4sp.2<br>P3 9.80 usec<br>P14 500.00 usec<br>P24 2000.00 usec<br>P63 1500.00 usec<br>PLW0 0 W<br>PLW2 60.95399857 W<br>PLW12 0.91469002 W<br>SPNAM[3] Crp60,0.5,20.1<br>SPOAL3 0.500<br>SPOFFS3 0 Hz<br>SPW3 8.94419956 W<br>SPNAM[7] Crp60comp.4<br>SPOAL7 0.500<br>SPOFFS7 0 Hz<br>SPW7 8.94419956 W<br>SPNAM[14 Crp32,1.5,20.2<br>SPOAL14 0.500<br>SPOFFS14 0 Hz<br>SPW14 3.81620002 W<br>SPNAM[31 Crp32,1.5,20.2<br>SPOAL31 0.500<br>SPOFFS31 0 Hz<br>SPW31 0.95405000 W<br><br>====== GRADIENT CHANNEL =====<br>GPNAM[1] SMSQ10.100<br>GPNAM[2] SMSQ10.100<br>GPNAM[3] SMSQ10.100<br>GPNAM[4] SMSQ10.100<br>GPZ1 80.00 %<br>GPZ2 20.10 %<br>GPZ3 11.00 %<br>GPZ4 -5.00 %<br>P16 1000.00 usec<br>P19 600.00 usec<br><br>F1 - Acquisition parameters<br>TD 256<br>SFO1 100.6203 MHz<br>FIDRES 130.208328 Hz<br>SW 165.639 ppm<br>FnMODE Echo-Antiecho<br><br>F2 - Processing parameters<br>SI 1024<br>SF 400.1300871 MHz<br>WDW QSINE<br>SSB 2<br>LB 0 Hz<br>GB 0<br>PC 1.40<br><br>F1 - Processing parameters<br>SI 1024<br>MC2 echo-antiecho<br>SF 100.6126499 MHz<br>WDW QSINE<br>SSB 2<br>LB 0 Hz<br>GB 0	F2 - Acquisition Parameters<br>Date_ 20171006<br>Time 12.43<br>INSTRUM spect<br>PROBHD 5 mm PABBO BB-<br>PULPROG hsqcetgpsisp2.2<br>TD 2048<br>SOLVENT MeOD<br>NS 2<br>DS 16<br>SWH 5341.880 Hz<br>FIDRES 2.608340 Hz<br>AQ 0.1916928 sec<br>RG 192.58<br>DW 93.600 usec<br>DE 6.50 usec<br>TE 298.3 K<br>CNST2 145.0000000<br>CNST17 -0.5000000<br>D0 0.00000300 sec<br>D1 1.50000000 sec<br>D4 0.00172414 sec<br>D11 0.03000000 sec<br>D16 0.00020000 sec<br>D24 0.00086207 sec<br>IN0 0.00003000 sec<br><br>======== CHANNEL f1 ========<br>SFO1 400.1324057 MHz<br>NUC1 1H<br>P1 15.00 usec<br>P2 30.00 usec<br>P28 1000.00 usec<br>PLW1 12.50300026 W<br><br>======== CHANNEL f2 ========<br>SFO2 100.6202713 MHz<br>NUC2 13C<br>CPDPRG[2 bi_p5m4sp_4sp.2<br>P3 9.80 usec<br>P14 500.00 usec<br>P24 2000.00 usec<br>P63 1500.00 usec<br>PLW0 0 W<br>PLW2 60.95399857 W<br>PLW12 0.91469002 W<br>SPNAM[3] Crp60,0.5,20.1<br>SPOAL3 0.500<br>SPOFFS3 0 Hz<br>SPW3 8.94419956 W<br>SPNAM[7] Crp60comp.4<br>SPOAL7 0.500<br>SPOFFS7 0 Hz<br>SPW7 8.94419956 W<br>SPNAM[14 Crp32,1.5,20.2<br>SPOAL14 0.500<br>SPOFFS14 0 Hz<br>SPW14 3.81620002 W<br>SPNAM[31 Crp32,1.5,20.2<br>SPOAL31 0.500<br>SPOFFS31 0 Hz<br>SPW31 0.95405000 W<br><br>====== GRADIENT CHANNEL =====<br>GPNAM[1] SMSQ10.100<br>GPNAM[2] SMSQ10.100<br>GPNAM[3] SMSQ10.100<br>GPNAM[4] SMSQ10.100<br>GPZ1 80.00 %<br>GPZ2 20.10 %<br>GPZ3 11.00 %<br>GPZ4 -5.00 %<br>P16 1000.00 usec<br>P19 600.00 usec<br><br>F1 - Acquisition parameters<br>TD 256<br>SFO1 100.6203 MHz<br>FIDRES 130.208328 Hz<br>SW 165.639 ppm<br>FnMODE Echo-Antiecho<br><br>F2 - Processing parameters<br>SI 1024<br>SF 400.1300052 MHz<br>WDW QSINE<br>SSB 2<br>LB 0 Hz<br>GB 0<br>PC 1.40<br><br>F1 - Processing parameters<br>SI 1024<br>MC2 echo-antiecho<br>SF 100.6126258 MHz<br>WDW QSINE<br>SSB 2<br>LB 0 Hz<br>GB 0

**HMBC**	F2 - Acquisition Parameters	F2 - Acquisition Parameters
	Date_ 20180509	Date_ 20171006
	Time 15.52	Time 12.26
	INSTRUM spect	INSTRUM spect
	PROBHD 5 mm PABBO BB-	PROBHD 5 mm PABBO BB-
	PULPROG hmbcgpl2ndqf	PULPROG hmbcgpl2ndqf
	TD 2048	TD 2048
	SOLVENT MeOD	SOLVENT MeOD
	NS 4	NS 4
	DS 16	DS 16
	SWH 4807.692 Hz	SWH 4807.692 Hz
	FIDRES 2.347506 Hz	FIDRES 2.347506 Hz
	AQ 0.2129920 sec	AQ 0.2129920 sec
	RG 192.58	RG 192.58
	DW 104.000 usec	DW 104.000 usec
	DE 6.50 usec	DE 6.50 usec
	TE 298.1 K	TE 298.1 K
	CNST6 125.0000000	CNST6 125.0000000
	CNST7 165.0000000	CNST7 165.0000000
	CNST13 7.5000000	CNST13 7.5000000
	D0 0.00000300 sec	D0 0.00000300 sec
	D1 1.50000000 sec	D1 1.50000000 sec
	D6 0.06666667 sec	D6 0.06666667 sec
	D16 0.00020000 sec	D16 0.00020000 sec
	IN0 0.00002240 sec	IN0 0.00002240 sec
	======== CHANNEL f1 ========	======== CHANNEL f1 ========
	SFO1 400.1322007 MHz	SFO1 400.1322007 MHz
	NUC1 1H	NUC1 1H
	P1 15.00 usec	P1 15.00 usec
	P2 30.00 usec	P2 30.00 usec
	PLW1 12.50300026 W	PLW1 12.50300026 W
	======== CHANNEL f2 ========	======== CHANNEL f2 ========
	SFO2 100.6228119 MHz	SFO2 100.6228119 MHz
	NUC2 13C	NUC2 13C
	P3 9.80 usec	P3 9.80 usec
	PLW2 60.95399857 W	PLW2 60.95399857 W
	====== GRADIENT CHANNEL =====	====== GRADIENT CHANNEL =====
	GPNAM[1] SMSQ10.100	GPNAM[1] SMSQ10.100
	GPNAM[2] SMSQ10.100	GPNAM[2] SMSQ10.100
	GPNAM[3] SMSQ10.100	GPNAM[3] SMSQ10.100
	GPNAM[4] SMSQ10.100	GPNAM[4] SMSQ10.100
	GPNAM[5] SMSQ10.100	GPNAM[5] SMSQ10.100
	GPNAM[6] SMSQ10.100	GPNAM[6] SMSQ10.100
	GPZ1 50.00 %	GPZ1 50.00 %
	GPZ2 30.00 %	GPZ2 30.00 %
	GPZ3 40.10 %	GPZ3 40.10 %
	GPZ4 15.00 %	GPZ4 15.00 %
	GPZ5 -10.00 %	GPZ5 -10.00 %
	GPZ6 -5.00 %	GPZ6 -5.00 %
	P16 1000.00 usec	P16 1000.00 usec
	F1 - Acquisition parameters	F1 - Acquisition parameters
	TD 128	TD 128
	SFO1 100.6228 MHz	SFO1 100.6228 MHz
	FIDRES 348.772308 Hz	FIDRES 348.772308 Hz
	SW 221.833 ppm	SW 221.833 ppm
	FnMODE QF	FnMODE QF
	F2 - Processing parameters	F2 - Processing parameters
	SI 2048	SI 2048
	SF 400.1300877 MHz	SF 400.1300000 MHz
	WDW SINE	WDW SINE
	SSB 0	SSB 0
	LB 0 Hz	LB 0 Hz
	GB 0	GB 0
	PC 1.40	PC 1.40
	F1 - Processing parameters	F1 - Processing parameters
	SI 1024	SI 1024
	MC2 QF	MC2 QF
	SF 100.6141285 MHz	SF 100.6127690 MHz
	WDW SINE	WDW SINE
	SSB 0	SSB 0
	LB 0 Hz	LB 0 Hz
	GB 0	GB 0

	ortho 2	ortho 3
^{1}H	F2 - Acquisition Parameters<br>Date_ 20171004<br>Time 15.36<br>INSTRUM spect<br>PROBHD 5 mm PABBO BB-<br>PULPROG zg<br>TD 48076<br>SOLVENT CDCl3<br>NS 8<br>DS 2<br>SWH 6002.401 Hz<br>FIDRES 0.124852 Hz<br>AQ 4.0047307 sec<br>RG 46.86<br>DW 83.300 usec<br>DE 16.70 usec<br>TE 298.1 K<br>D1 5.00000000 sec<br>TD0 1<br><br>======== CHANNEL f1 ========<br>SFO1 400.1320007 MHz<br>NUC1 1H<br>P1 15.00 usec<br>PLW1 12.50300026 W<br><br>F2 - Processing parameters<br>SI 32768<br>SF 400.1300100 MHz<br>WDW EM<br>SSB 0<br>LB 0.30 Hz<br>GB 0<br>PC 1.00	F2 - Acquisition Parameters<br>Date_ 20171009<br>Time 12.05<br>INSTRUM spect<br>PROBHD 5 mm PABBO BB-<br>PULPROG zg30<br>TD 65536<br>SOLVENT D2O<br>NS 16<br>DS 2<br>SWH 8012.820 Hz<br>FIDRES 0.122266 Hz<br>AQ 4.0894465 sec<br>RG 119.51<br>DW 62.400 usec<br>DE 6.50 usec<br>TE 298.2 K<br>D1 1.00000000 sec<br>TD0 1<br><br>======== CHANNEL f1 ========<br>SFO1 400.1324710 MHz<br>NUC1 1H<br>P1 15.00 usec<br>PLW1 12.50300026 W<br><br>F2 - Processing parameters<br>SI 65536<br>SF 400.1299661 MHz<br>WDW EM<br>SSB 0<br>LB 0.30 Hz<br>GB 0<br>PC 1.00

^{13}C	F2 - Acquisition Parameters<br>Date_ 20171004<br>Time 21.00<br>INSTRUM spect<br>PROBHD 5 mm PABBO BB-<br>PULPROG zgpg30<br>TD 65536<br>SOLVENT CDCl3<br>NS 4000<br>DS 4<br>SWH 22058.824 Hz<br>FIDRES 0.336591 Hz<br>AQ 1.4854827 sec<br>RG 192.58<br>DW 22.667 usec<br>DE 6.50 usec<br>TE 298.2 K<br>D1 2.00000000 sec<br>D11 0.03000000 sec<br>TD0 1<br><br>======== CHANNEL f1 ========<br>SFO1 100.6223253 MHz<br>NUC1 13C<br>P1 9.80 usec<br>PLW1 60.95399857 W<br><br>======== CHANNEL f2 ========<br>SFO2 400.1316005 MHz<br>NUC2 1H<br>CPDPRG[2 waltz16<br>PCPD2 90.00 usec<br>PLW2 12.50300026 W<br>PLW12 0.34731001 W<br>PLW13 0.28132001 W<br><br>F2 - Processing parameters<br>SI 65536<br>SF 100.6127573 MHz<br>WDW EM<br>SSB 0<br>LB 1.00 Hz<br>GB 0<br>PC 1.40	F2 - Acquisition Parameters<br>Date_ 20171012<br>Time 7.14<br>INSTRUM spect<br>PROBHD 5 mm PABBO BB-<br>PULPROG zgpg30<br>TD 65536<br>SOLVENT D2O<br>NS 4000<br>DS 4<br>SWH 22058.824 Hz<br>FIDRES 0.336591 Hz<br>AQ 1.4854827 sec<br>RG 192.58<br>DW 22.667 usec<br>DE 6.50 usec<br>TE 298.2 K<br>D1 2.00000000 sec<br>D11 0.03000000 sec<br>TD0 1<br><br>======== CHANNEL f1 ========<br>SFO1 100.6223253 MHz<br>NUC1 13C<br>P1 9.80 usec<br>PLW1 60.95399857 W<br><br>======== CHANNEL f2 ========<br>SFO2 400.1316005 MHz<br>NUC2 1H<br>CPDPRG[2 waltz16<br>PCPD2 90.00 usec<br>PLW2 12.50300026 W<br>PLW12 0.34731001 W<br>PLW13 0.28132001 W<br><br>F2 - Processing parameters<br>SI 65536<br>SF 100.6127690 MHz<br>WDW EM<br>SSB 0<br>LB 1.00 Hz<br>GB 0<br>PC 1.40

**DEPT**	F2 - Acquisition Parameters	F2 - Acquisition Parameters
	Date_ 20171004	Date_ 20171010
	Time 15.53	Time 14.59
	INSTRUM spect	INSTRUM spect
	PROBHD 5 mm PABBO BB-	PROBHD 5 mm PABBO BB-
	PULPROG deptqgpsp	PULPROG deptqgpsp
	TD 65536	TD 65536
	SOLVENT CDCl3	SOLVENT D2O
	NS 256	NS 256
	DS 4	DS 4
	SWH 22058.824 Hz	SWH 22058.824 Hz
	FIDRES 0.336591 Hz	FIDRES 0.336591 Hz
	AQ 1.4854827 sec	AQ 1.4854827 sec
	RG 192.58	RG 192.58
	DW 22.667 usec	DW 22.667 usec
	DE 6.50 usec	DE 6.50 usec
	TE 298.2 K	TE 298.2 K
	CNST2 145.0000000	CNST2 145.0000000
	CNST12 1.5000000	CNST12 1.5000000
	D1 2.00000000 sec	D1 2.00000000 sec
	D2 0.00344828 sec	D2 0.00344828 sec
	D12 0.00002000 sec	D12 0.00002000 sec
	D16 0.00020000 sec	D16 0.00020000 sec
	TD0 1	TD0 1
	======== CHANNEL f1 ========	======== CHANNEL f1 ========
	SFO1 100.6223258 MHz	SFO1 100.6223258 MHz
	NUC1 13C	NUC1 13C
	P1 9.80 usec	P1 9.80 usec
	P13 2000.00 usec	P13 2000.00 usec
	PLW0 0 W	PLW0 0 W
	PLW1 60.95399857 W	PLW1 60.95399857 W
	SPNAM[5] Crp60comp.4	SPNAM[5] Crp60comp.4
	SPOAL5 0.500	SPOAL5 0.500
	SPOFFS5 0 Hz	SPOFFS5 0 Hz
	SPW5 8.94419956 W	SPW5 8.94419956 W
	======== CHANNEL f2 ========	======== CHANNEL f2 ========
	SFO2 400.1316005 MHz	SFO2 400.1316005 MHz
	NUC2 1H	NUC2 1H
	CPDPRG[2 waltz16	CPDPRG[2 waltz16
	P0 22.50 usec	P0 22.50 usec
	P3 15.00 usec	P3 15.00 usec
	P4 30.00 usec	P4 30.00 usec
	PCPD2 90.00 usec	PCPD2 90.00 usec
	PLW2 12.50300026 W	PLW2 12.50300026 W
	PLW12 0.34731001 W	PLW12 0.34731001 W
	====== GRADIENT CHANNEL =====	====== GRADIENT CHANNEL =====
	GPNAM[1] SMSQ10.32	GPNAM[1] SMSQ10.32
	GPNAM[2] SMSQ10.32	GPNAM[2] SMSQ10.32
	GPNAM[3] SMSQ10.32	GPNAM[3] SMSQ10.32
	GPZ1 31.00 %	GPZ1 31.00 %
	GPZ2 31.00 %	GPZ2 31.00 %
	GPZ3 31.00 %	GPZ3 31.00 %
	P16 1000.00 usec	P16 1000.00 usec
	F2 - Processing parameters	F2 - Processing parameters
	SI 65536	SI 65536
	SF 100.6127690 MHz	SF 100.6127690 MHz
	WDW EM	WDW EM
	SSB 0	SSB 0
	LB 1.00 Hz	LB 1.00 Hz
	GB 0	GB 0
	PC 1.40	PC 1.40

^{11}B	F2 - Acquisition Parameters<br>Date_ 20171026<br>Time 12.25<br>INSTRUM spect<br>PROBHD 5 mm PABBO BB-<br>PULPROG zg<br>TD 65536<br>SOLVENT CDCl3<br>NS 128<br>DS 4<br>SWH 25510.203 Hz<br>FIDRES 0.389255 Hz<br>AQ 1.2845056 sec<br>RG 192.58<br>DW 19.600 usec<br>DE 6.50 usec<br>TE 298.2 K<br>D1 1.00000000 sec<br>TD0 1<br><br>======== CHANNEL f1 ========<br>SFO1 128.3776052 MHz<br>NUC1 11B<br>P1 17.05 usec<br>PLW1 11.69499969 W<br><br>F2 - Processing parameters<br>SI 32768<br>SF 128.3776052 MHz<br>WDW no<br>SSB 0<br>LB 0 Hz<br>GB 0<br>PC 1.40	F2 - Acquisition Parameters<br>Date_ 20171011<br>Time 6.57<br>INSTRUM spect<br>PROBHD 5 mm PABBO BB-<br>PULPROG zg<br>TD 65536<br>SOLVENT D2O<br>NS 128<br>DS 4<br>SWH 25510.203 Hz<br>FIDRES 0.389255 Hz<br>AQ 1.2845056 sec<br>RG 192.58<br>DW 19.600 usec<br>DE 6.50 usec<br>TE 298.1 K<br>D1 1.00000000 sec<br>TD0 1<br><br>======== CHANNEL f1 ========<br>SFO1 128.3776052 MHz<br>NUC1 11B<br>P1 17.05 usec<br>PLW1 11.69499969 W<br><br>F2 - Processing parameters<br>SI 32768<br>SF 128.3776052 MHz<br>WDW no<br>SSB 0<br>LB 0 Hz<br>GB 0<br>PC 1.40

COSY		
	F2 - Acquisition Parameters<br>Date_ 20171004<br>Time 15.55<br>INSTRUM spect<br>PROBHD 5 mm PABBO BB-<br>PULPROG cosygpqf<br>TD 2048<br>SOLVENT CDCl3<br>NS 1<br>DS 8<br>SWH 4807.692 Hz<br>FIDRES 2.347506 Hz<br>AQ 0.2129920 sec<br>RG 107<br>DW 104.000 usec<br>DE 6.50 usec<br>TE 298.0 K<br>D0 0.00000300 sec<br>D1 1.48689198 sec<br>D13 0.00000400 sec<br>D16 0.00020000 sec<br>IN0 0.00020800 sec<br><br>======== CHANNEL f1 ========<br>SFO1 400.1322007 MHz<br>NUC1 1H<br>P0 15.00 usec<br>P1 15.00 usec<br>PLW1 12.50300026 W<br><br>====== GRADIENT CHANNEL =====<br>GPNAM[1] SMSQ10.100<br>GPZ1 10.00 %<br>P16 1000.00 usec<br><br>F1 - Acquisition parameters<br>TD 128<br>SFO1 400.1322 MHz<br>FIDRES 75.120193 Hz<br>SW 12.015 ppm<br>FnMODE QF<br><br>F2 - Processing parameters<br>SI 1024<br>SF 400.1300093 MHz<br>WDW SINE<br>SSB 0<br>LB 0 Hz<br>GB 0<br>PC 1.40<br><br>F1 - Processing parameters<br>SI 1024<br>MC2 QF<br>SF 400.1300094 MHz<br>WDW SINE<br>SSB 0<br>LB 0 Hz<br>GB 0	F2 - Acquisition Parameters<br>Date_ 20171010<br>Time 15.01<br>INSTRUM spect<br>PROBHD 5 mm PABBO BB-<br>PULPROG cosygpqf<br>TD 2048<br>SOLVENT D2O<br>NS 1<br>DS 8<br>SWH 4807.692 Hz<br>FIDRES 2.347506 Hz<br>AQ 0.2129920 sec<br>RG 192.58<br>DW 104.000 usec<br>DE 6.50 usec<br>TE 298.0 K<br>D0 0.00000300 sec<br>D1 1.48689198 sec<br>D13 0.00000400 sec<br>D16 0.00020000 sec<br>IN0 0.00020800 sec<br><br>======== CHANNEL f1 ========<br>SFO1 400.1322007 MHz<br>NUC1 1H<br>P0 15.00 usec<br>P1 15.00 usec<br>PLW1 12.50300026 W<br><br>====== GRADIENT CHANNEL =====<br>GPNAM[1] SMSQ10.100<br>GPZ1 10.00 %<br>P16 1000.00 usec<br><br>F1 - Acquisition parameters<br>TD 128<br>SFO1 400.1322 MHz<br>FIDRES 75.120193 Hz<br>SW 12.015 ppm<br>FnMODE QF<br><br>F2 - Processing parameters<br>SI 1024<br>SF 400.1299653 MHz<br>WDW SINE<br>SSB 0<br>LB 0 Hz<br>GB 0<br>PC 1.40<br><br>F1 - Processing parameters<br>SI 1024<br>MC2 QF<br>SF 400.1299652 MHz<br>WDW SINE<br>SSB 0<br>LB 0 Hz<br>GB 0

**HSQC**	F2 - Acquisition Parameters	F2 - Acquisition Parameters
	Date_ 20171004	Date_ 20171010
	Time 16.18	Time 15.41
	INSTRUM spect	INSTRUM spect
	PROBHD 5 mm PABBO BB-	PROBHD 5 mm PABBO BB-
	PULPROG hsqcetgpsisp2.2	PULPROG hsqcetgpsisp2.2
	TD 2048	TD 2048
	SOLVENT CDCl3	SOLVENT D2O
	NS 2	NS 2
	DS 16	DS 16
	SWH 5341.880 Hz	SWH 5341.880 Hz
	FIDRES 2.608340 Hz	FIDRES 2.608340 Hz
	AQ 0.1916928 sec	AQ 0.1916928 sec
	RG 192.58	RG 192.58
	DW 93.600 usec	DW 93.600 usec
	DE 6.50 usec	DE 6.50 usec
	TE 298.3 K	TE 298.2 K
	CNST2 145.0000000	CNST2 145.0000000
	CNST17 -0.5000000	CNST17 -0.5000000
	D0 0.00000300 sec	D0 0.00000300 sec
	D1 1.50000000 sec	D1 1.50000000 sec
	D4 0.00172414 sec	D4 0.00172414 sec
	D11 0.03000000 sec	D11 0.03000000 sec
	D16 0.00020000 sec	D16 0.00020000 sec
	D24 0.00086207 sec	D24 0.00086207 sec
	IN0 0.00003000 sec	IN0 0.00003000 sec
	======== CHANNEL f1 ========	======== CHANNEL f1 ========
	SFO1 400.1324057 MHz	SFO1 400.1324057 MHz
	NUC1 1H	NUC1 1H
	P1 15.00 usec	P1 15.00 usec
	P2 30.00 usec	P2 30.00 usec
	P28 1000.00 usec	P28 1000.00 usec
	PLW1 12.50300026 W	PLW1 12.50300026 W
	======== CHANNEL f2 ========	======== CHANNEL f2 ========
	SFO2 100.6202713 MHz	SFO2 100.6202713 MHz
	NUC2 13C	NUC2 13C
	CPDPRG[2 bi_p5m4sp_4sp.2	CPDPRG[2 bi_p5m4sp_4sp.2
	P3 9.80 usec	P3 9.80 usec
	P14 500.00 usec	P14 500.00 usec
	P24 2000.00 usec	P24 2000.00 usec
	P63 1500.00 usec	P63 1500.00 usec
	PLW0 0 W	PLW0 0 W
	PLW2 60.95399857 W	PLW2 60.95399857 W
	PLW12 0.91469002 W	PLW12 0.91469002 W
	SPNAM[3] Crp60,0.5,20.1	SPNAM[3] Crp60,0.5,20.1
	SPOAL3 0.500	SPOAL3 0.500
	SPOFFS3 0 Hz	SPOFFS3 0 Hz
	SPW3 8.94419956 W	SPW3 8.94419956 W
	SPNAM[7] Crp60comp.4	SPNAM[7] Crp60comp.4
	SPOAL7 0.500	SPOAL7 0.500
	SPOFFS7 0 Hz	SPOFFS7 0 Hz
	SPW7 8.94419956 W	SPW7 8.94419956 W
	SPNAM[14 Crp32,1.5,20.2	SPNAM[14 Crp32,1.5,20.2
	SPOAL14 0.500	SPOAL14 0.500
	SPOFFS14 0 Hz	SPOFFS14 0 Hz
	SPW14 3.81620002 W	SPW14 3.81620002 W
	SPNAM[31 Crp32,1.5,20.2	SPNAM[31 Crp32,1.5,20.2
	SPOAL31 0.500	SPOAL31 0.500
	SPOFFS31 0 Hz	SPOFFS31 0 Hz
	SPW31 0.95405000 W	SPW31 0.95405000 W
	====== GRADIENT CHANNEL =====	====== GRADIENT CHANNEL =====
	GPNAM[1] SMSQ10.100	GPNAM[1] SMSQ10.100
	GPNAM[2] SMSQ10.100	GPNAM[2] SMSQ10.100
	GPNAM[3] SMSQ10.100	GPNAM[3] SMSQ10.100
	GPNAM[4] SMSQ10.100	GPNAM[4] SMSQ10.100
	GPZ1 80.00 %	GPZ1 80.00 %
	GPZ2 20.10 %	GPZ2 20.10 %
	GPZ3 11.00 %	GPZ3 11.00 %
	GPZ4 -5.00 %	GPZ4 -5.00 %
	P16 1000.00 usec	P16 1000.00 usec
	P19 600.00 usec	P19 600.00 usec
	F1 - Acquisition parameters	F1 - Acquisition parameters
	TD 256	TD 256
	SFO1 100.6203 MHz	SFO1 100.6203 MHz
	FIDRES 130.208328 Hz	FIDRES 130.208328 Hz
	SW 165.639 ppm	SW 165.639 ppm
	FnMODE Echo-Antiecho	FnMODE Echo-Antiecho
	F2 - Processing parameters	F2 - Processing parameters
	SI 1024	SI 1024
	SF 400.1300099 MHz	SF 400.1299674 MHz
	WDW QSINE	WDW QSINE
	SSB 2	SSB 2
	LB 0 Hz	LB 0 Hz
	GB 0	GB 0
	PC 1.40	PC 1.40
	F1 - Processing parameters	F1 - Processing parameters
	SI 1024	SI 1024
	MC2 echo-antiecho	MC2 echo-antiecho
	SF 100.6127604 MHz	SF 100.6127649 MHz
	WDW QSINE	WDW QSINE
	SSB 2	SSB 2
	LB 0 Hz	LB 0 Hz
	GB 0	GB 0

**HMBC**	F2 - Acquisition Parameters	F2 - Acquisition Parameters
	Date_ 20171004	Date_ 20171010
	Time 16.01	Time 15.06
	INSTRUM spect	INSTRUM spect
	PROBHD 5 mm PABBO BB-	PROBHD 5 mm PABBO BB-
	PULPROG hmbcgpl2ndqf	PULPROG hmbcgpl2ndqf
	TD 2048	TD 2048
	SOLVENT CDC13	SOLVENT D2O
	NS 4	NS 4
	DS 16	DS 16
	SWH 4807.692 Hz	SWH 4807.692 Hz
	FIDRES 2.347506 Hz	FIDRES 2.347506 Hz
	AQ 0.2129920 sec	AQ 0.2129920 sec
	RG 192.58	RG 192.58
	DW 104.000 usec	DW 104.000 usec
	DE 6.50 usec	DE 6.50 usec
	TE 298.1 K	TE 298.1 K
	CNST6 125.0000000	CNST6 125.0000000
	CNST7 165.0000000	CNST7 165.0000000
	CNST13 7.5000000	CNST13 7.5000000
	D0 0.00000300 sec	D0 0.00000300 sec
	D1 1.50000000 sec	D1 1.50000000 sec
	D6 0.06666667 sec	D6 0.06666667 sec
	D16 0.00020000 sec	D16 0.00020000 sec
	IN0 0.00002240 sec	IN0 0.00002240 sec
	======== CHANNEL f1 ========	======== CHANNEL f1 ========
	SFO1 400.1322007 MHz	SFO1 400.1322007 MHz
	NUC1 1H	NUC1 1H
	P1 15.00 usec	P1 15.00 usec
	P2 30.00 usec	P2 30.00 usec
	PLW1 12.50300026 W	PLW1 12.50300026 W
	======== CHANNEL f2 ========	======== CHANNEL f2 ========
	SFO2 100.6228119 MHz	SFO2 100.6228119 MHz
	NUC2 13C	NUC2 13C
	P3 9.80 usec	P3 9.80 usec
	PLW2 60.95399857 W	PLW2 60.95399857 W
	====== GRADIENT CHANNEL =====	====== GRADIENT CHANNEL =====
	GPNAM[1] SMSQ10.100	GPNAM[1] SMSQ10.100
	GPNAM[2] SMSQ10.100	GPNAM[2] SMSQ10.100
	GPNAM[3] SMSQ10.100	GPNAM[3] SMSQ10.100
	GPNAM[4] SMSQ10.100	GPNAM[4] SMSQ10.100
	GPNAM[5] SMSQ10.100	GPNAM[5] SMSQ10.100
	GPNAM[6] SMSQ10.100	GPNAM[6] SMSQ10.100
	GPZ1 50.00 %	GPZ1 50.00 %
	GPZ2 30.00 %	GPZ2 30.00 %
	GPZ3 40.10 %	GPZ3 40.10 %
	GPZ4 15.00 %	GPZ4 15.00 %
	GPZ5 -10.00 %	GPZ5 -10.00 %
	GPZ6 -5.00 %	GPZ6 -5.00 %
	P16 1000.00 usec	P16 1000.00 usec
	F1 - Acquisition parameters	F1 - Acquisition parameters
	TD 128	TD 128
	SFO1 100.6228 MHz	SFO1 100.6228 MHz
	FIDRES 348.772308 Hz	FIDRES 348.772308 Hz
	SW 221.833 ppm	SW 221.833 ppm
	FnMODE QF	FnMODE QF
	F2 - Processing parameters	F2 - Processing parameters
	SI 2048	SI 2048
	SF 400.1300104 MHz	SF 400.1299655 MHz
	WDW SINE	WDW SINE
	SSB 0	SSB 0
	LB 0 Hz	LB 0 Hz
	GB 0	GB 0
	PC 1.40	PC 1.40
	F1 - Processing parameters	F1 - Processing parameters
	SI 1024	SI 1024
	MC2 QF	MC2 QF
	SF 100.6127690 MHz	SF 100.6127690 MHz
	WDW SINE	WDW SINE
	SSB 0	SSB 0
	LB 0 Hz	LB 0 Hz
	GB 0	GB 0

	ortho 4	ortho 5
^{1}H	F2 - Acquisition Parameters<br>Date_ 20180308<br>Time 15.49<br>INSTRUM spect<br>PROBHD 5 mm PABBO BB-<br>PULPROG zg30<br>TD 65536<br>SOLVENT D2O<br>NS 16<br>DS 2<br>SWH 8012.820 Hz<br>FIDRES 0.122266 Hz<br>AQ 4.0894465 sec<br>RG 23.44<br>DW 62.400 usec<br>DE 6.50 usec<br>TE 298.2 K<br>D1 1.00000000 sec<br>TD0 1<br><br>======== CHANNEL f1 ========<br>SFO1 400.1324710 MHz<br>NUC1 1H<br>P1 15.00 usec<br>PLW1 12.50300026 W<br><br>F2 - Processing parameters<br>SI 65536<br>SF 400.1299640 MHz<br>WDW EM<br>SSB 0<br>LB 0.30 Hz<br>GB 0<br>PC 1.00	F2 - Acquisition Parameters<br>Date_ 20171012<br>Time 11.33<br>INSTRUM spect<br>PROBHD 5 mm PABBO BB-<br>PULPROG zg<br>TD 48076<br>SOLVENT D2O<br>NS 8<br>DS 2<br>SWH 6002.401 Hz<br>FIDRES 0.124852 Hz<br>AQ 4.0047307 sec<br>RG 132.62<br>DW 83.300 usec<br>DE 16.70 usec<br>TE 298.2 K<br>D1 5.00000000 sec<br>TD0 1<br><br>======== CHANNEL f1 ========<br>SFO1 400.1320007 MHz<br>NUC1 1H<br>P1 15.00 usec<br>PLW1 12.50300026 W<br><br>F2 - Processing parameters<br>SI 32768<br>SF 400.1299643 MHz<br>WDW EM<br>SSB 0<br>LB 0.30 Hz<br>GB 0<br>PC 1.00

^{13}C	F2 - Acquisition Parameters<br>Date_ 20180312<br>Time 0.07<br>INSTRUM spect<br>PROBHD 5 mm PABBO BB-<br>PULPROG zgpg30<br>TD 65536<br>SOLVENT D2O<br>NS 3000<br>DS 4<br>SWH 22058.824 Hz<br>FIDRES 0.336591 Hz<br>AQ 1.4854827 sec<br>RG 192.58<br>DW 22.667 usec<br>DE 6.50 usec<br>TE 298.2 K<br>D1 2.00000000 sec<br>D11 0.03000000 sec<br>TD0 1<br><br>======== CHANNEL f1 ========<br>SFO1 100.6223253 MHz<br>NUC1 13C<br>P1 9.80 usec<br>PLW1 60.95399857 W<br><br>======== CHANNEL f2 ========<br>SFO2 400.1316005 MHz<br>NUC2 1H<br>CPDPRG[2 waltz16<br>PCPD2 90.00 usec<br>PLW2 12.50300026 W<br>PLW12 0.34731001 W<br>PLW13 0.28132001 W<br><br>F2 - Processing parameters<br>SI 65536<br>SF 100.6127690 MHz<br>WDW EM<br>SSB 0<br>LB 1.00 Hz<br>GB 0<br>PC 1.40	F2 - Acquisition Parameters<br>Date_ 20171014<br>Time 22.26<br>INSTRUM spect<br>PROBHD 5 mm PABBO BB-<br>PULPROG zgpg30<br>TD 65536<br>SOLVENT D2O<br>NS 4000<br>DS 4<br>SWH 22058.824 Hz<br>FIDRES 0.336591 Hz<br>AQ 1.4854827 sec<br>RG 192.58<br>DW 22.667 usec<br>DE 6.50 usec<br>TE 298.2 K<br>D1 2.00000000 sec<br>D11 0.03000000 sec<br>TD0 1<br><br>======== CHANNEL f1 ========<br>SFO1 100.6223253 MHz<br>NUC1 13C<br>P1 9.80 usec<br>PLW1 60.95399857 W<br><br>======== CHANNEL f2 ========<br>SFO2 400.1316005 MHz<br>NUC2 1H<br>CPDPRG[2 waltz16<br>PCPD2 90.00 usec<br>PLW2 12.50300026 W<br>PLW12 0.34731001 W<br>PLW13 0.28132001 W<br><br>F2 - Processing parameters<br>SI 65536<br>SF 100.6127690 MHz<br>WDW EM<br>SSB 0<br>LB 1.00 Hz<br>GB 0<br>PC 1.40

**DEPT**	F2 - Acquisition Parameters<br>Date_ 20180308<br>Time 17.16<br>INSTRUM spect<br>PROBHD 5 mm PABBO BB-<br>PULPROG deptqgpsp<br>TD 65536<br>SOLVENT D2O<br>NS 256<br>DS 4<br>SWH 22058.824 Hz<br>FIDRES 0.336591 Hz<br>AQ 1.4854827 sec<br>RG 192.58<br>DW 22.667 usec<br>DE 6.50 usec<br>TE 298.2 K<br>CNST2 145.0000000<br>CNST12 1.5000000<br>D1 2.00000000 sec<br>D2 0.00344828 sec<br>D12 0.00002000 sec<br>D16 0.00020000 sec<br>TD0 1<br><br>======== CHANNEL f1 ========<br>SFO1 100.6223258 MHz<br>NUC1 13C<br>P1 9.80 usec<br>P13 2000.00 usec<br>PLW0 0 W<br>PLW1 60.95399857 W<br>SPNAM[5] Crp60comp.4<br>SPOAL5 0.500<br>SPOFFS5 0 Hz<br>SPW5 8.94419956 W<br><br>======== CHANNEL f2 ========<br>SFO2 400.1316005 MHz<br>NUC2 1H<br>CPDPRG[2 waltz16<br>P0 22.50 usec<br>P3 15.00 usec<br>P4 30.00 usec<br>PCPD2 90.00 usec<br>PLW2 12.50300026 W<br>PLW12 0.34731001 W<br><br>====== GRADIENT CHANNEL =====<br>GPNAM[1] SMSQ10.32<br>GPNAM[2] SMSQ10.32<br>GPNAM[3] SMSQ10.32<br>GPZ1 31.00 %<br>GPZ2 31.00 %<br>GPZ3 31.00 %<br>P16 1000.00 usec<br><br>F2 - Processing parameters<br>SI 65536<br>SF 100.6127690 MHz<br>WDW EM<br>SSB 0<br>LB 1.00 Hz<br>GB 0<br>PC 1.40	F2 - Acquisition Parameters<br>Date_ 20171012<br>Time 11.51<br>INSTRUM spect<br>PROBHD 5 mm PABBO BB-<br>PULPROG deptqgpsp<br>TD 65536<br>SOLVENT D2O<br>NS 256<br>DS 4<br>SWH 22058.824 Hz<br>FIDRES 0.336591 Hz<br>AQ 1.4854827 sec<br>RG 192.58<br>DW 22.667 usec<br>DE 6.50 usec<br>TE 298.2 K<br>CNST2 145.0000000<br>CNST12 1.5000000<br>D1 2.00000000 sec<br>D2 0.00344828 sec<br>D12 0.00002000 sec<br>D16 0.00020000 sec<br>TD0 1<br><br>======== CHANNEL f1 ========<br>SFO1 100.6223258 MHz<br>NUC1 13C<br>P1 9.80 usec<br>P13 2000.00 usec<br>PLW0 0 W<br>PLW1 60.95399857 W<br>SPNAM[5] Crp60comp.4<br>SPOAL5 0.500<br>SPOFFS5 0 Hz<br>SPW5 8.94419956 W<br><br>======== CHANNEL f2 ========<br>SFO2 400.1316005 MHz<br>NUC2 1H<br>CPDPRG[2 waltz16<br>P0 22.50 usec<br>P3 15.00 usec<br>P4 30.00 usec<br>PCPD2 90.00 usec<br>PLW2 12.50300026 W<br>PLW12 0.34731001 W<br><br>====== GRADIENT CHANNEL =====<br>GPNAM[1] SMSQ10.32<br>GPNAM[2] SMSQ10.32<br>GPNAM[3] SMSQ10.32<br>GPZ1 31.00 %<br>GPZ2 31.00 %<br>GPZ3 31.00 %<br>P16 1000.00 usec<br><br>F2 - Processing parameters<br>SI 65536<br>SF 100.6127690 MHz<br>WDW EM<br>SSB 0<br>LB 1.00 Hz<br>GB 0<br>PC 1.40

^{11}B	F2 - Acquisition Parameters<br>Date_ 20180312<br>Time 0.31<br>INSTRUM spect<br>PROBHD 5 mm PABBO BB-<br>PULPROG zg<br>TD 65536<br>SOLVENT D2O<br>NS 128<br>DS 4<br>SWH 25510.203 Hz<br>FIDRES 0.389255 Hz<br>AQ 1.2845056 sec<br>RG 192.58<br>DW 19.600 usec<br>DE 6.50 usec<br>TE 298.1 K<br>D1 1.00000000 sec<br>TD0 1<br><br>======== CHANNEL f1 ========<br>SFO1 128.3776052 MHz<br>NUC1 11B<br>P1 17.05 usec<br>PLW1 11.69499969 W<br><br>F2 - Processing parameters<br>SI 32768<br>SF 128.3776052 MHz<br>WDW no<br>SSB 0<br>LB 0 Hz<br>GB 0<br>PC 1.40	F2 - Acquisition Parameters<br>Date_ 20171013<br>Time 7.17<br>INSTRUM spect<br>PROBHD 5 mm PABBO BB-<br>PULPROG zg<br>TD 65536<br>SOLVENT D2O<br>NS 128<br>DS 4<br>SWH 25510.203 Hz<br>FIDRES 0.389255 Hz<br>AQ 1.2845056 sec<br>RG 192.58<br>DW 19.600 usec<br>DE 6.50 usec<br>TE 298.1 K<br>D1 1.00000000 sec<br>TD0 1<br><br>======== CHANNEL f1 ========<br>SFO1 128.3776052 MHz<br>NUC1 11B<br>P1 17.05 usec<br>PLW1 11.69499969 W<br><br>F2 - Processing parameters<br>SI 32768<br>SF 128.3776052 MHz<br>WDW no<br>SSB 0<br>LB 0 Hz<br>GB 0<br>PC 1.40

**COSY**	F2 - Acquisition Parameters<br>Date_ 20180309<br>Time 9.05<br>INSTRUM spect<br>PROBHD 5 mm PABBO BB-<br>PULPROG cosygpqf<br>TD 2048<br>SOLVENT D2O<br>NS 1<br>DS 8<br>SWH 4807.692 Hz<br>FIDRES 2.347506 Hz<br>AQ 0.2129920 sec<br>RG 86.04<br>DW 104.000 usec<br>DE 6.50 usec<br>TE 298.1 K<br>D0 0.00000300 sec<br>D1 1.48689198 sec<br>D13 0.00000400 sec<br>D16 0.00020000 sec<br>IN0 0.00020800 sec<br><br>======== CHANNEL f1 ========<br>SFO1 400.1322007 MHz<br>NUC1 1H<br>P0 15.00 usec<br>P1 15.00 usec<br>PLW1 12.50300026 W<br><br>====== GRADIENT CHANNEL =====<br>GPNAM[1] SMSQ10.100<br>GPZ1 10.00 %<br>P16 1000.00 usec<br><br>F1 - Acquisition parameters<br>TD 128<br>SFO1 400.1322 MHz<br>FIDRES 75.120193 Hz<br>SW 12.015 ppm<br>FnMODE QF<br><br>F2 - Processing parameters<br>SI 1024<br>SF 400.1299639 MHz<br>WDW SINE<br>SSB 0<br>LB 0 Hz<br>GB 0<br>PC 1.40<br><br>F1 - Processing parameters<br>SI 1024<br>MC2 QF<br>SF 400.1299637 MHz<br>WDW SINE<br>SSB 0<br>LB 0 Hz<br>GB 0	F2 - Acquisition Parameters<br>Date_ 20171012<br>Time 11.53<br>INSTRUM spect<br>PROBHD 5 mm PABBO BB-<br>PULPROG cosygpqf<br>TD 2048<br>SOLVENT D2O<br>NS 1<br>DS 8<br>SWH 4807.692 Hz<br>FIDRES 2.347506 Hz<br>AQ 0.2129920 sec<br>RG 192.58<br>DW 104.000 usec<br>DE 6.50 usec<br>TE 298.0 K<br>D0 0.00000300 sec<br>D1 1.48689198 sec<br>D13 0.00000400 sec<br>D16 0.00020000 sec<br>IN0 0.00020800 sec<br><br>======== CHANNEL f1 ========<br>SFO1 400.1322007 MHz<br>NUC1 1H<br>P0 15.00 usec<br>P1 15.00 usec<br>PLW1 12.50300026 W<br><br>====== GRADIENT CHANNEL =====<br>GPNAM[1] SMSQ10.100<br>GPZ1 10.00 %<br>P16 1000.00 usec<br><br>F1 - Acquisition parameters<br>TD 128<br>SFO1 400.1322 MHz<br>FIDRES 75.120193 Hz<br>SW 12.015 ppm<br>FnMODE QF<br><br>F2 - Processing parameters<br>SI 1024<br>SF 400.1299635 MHz<br>WDW SINE<br>SSB 0<br>LB 0 Hz<br>GB 0<br>PC 1.40<br><br>F1 - Processing parameters<br>SI 1024<br>MC2 QF<br>SF 400.1299638 MHz<br>WDW SINE<br>SSB 0<br>LB 0 Hz<br>GB 0

HSQC

```
F2 - Acquisition Parameters
Date_          20180312
Time               0.10
INSTRUM           spect
PROBHD   5 mm PABBO BB-
PULPROG  hsqcetgpsisp2.2
TD                 2048
SOLVENT             D2O
NS                    2
DS                   16
SWH            5341.880 Hz
FIDRES         2.608340 Hz
AQ            0.1916928 sec
RG               192.58
DW               93.600 usec
DE                 6.50 usec
TE                298.0 K
CNST2       145.0000000
CNST17       -0.5000000
D0           0.00000300 sec
D1           1.50000000 sec
D4           0.00172414 sec
D11          0.03000000 sec
D16          0.00020000 sec
D24          0.00086207 sec
IN0          0.00003000 sec

======== CHANNEL f1 ========
SFO1        400.1324057 MHz
NUC1                 1H
P1                15.00 usec
P2                30.00 usec
P28             1000.00 usec
PLW1        12.50300026 W

======== CHANNEL f2 ========
SFO2        100.6202713 MHz
NUC2                13C
CPDPRG[2 bi_p5m4sp_4sp.2
P3                 9.80 usec
P14              500.00 usec
P24             2000.00 usec
P63             1500.00 usec
PLW0                  0 W
PLW2        60.95399857 W
PLW12        0.91469002 W
SPNAM[3] Crp60,0.5,20.1
SPOAL3            0.500
SPOFFS3               0 Hz
SPW3         8.94419956 W
SPNAM[7]    Crp60comp.4
SPOAL7            0.500
SPOFFS7               0 Hz
SPW7         8.94419956 W
SPNAM[14 Crp32,1.5,20.2
SPOAL14           0.500
SPOFFS14              0 Hz
SPW14        3.81620002 W
SPNAM[31 Crp32,1.5,20.2
SPOAL31           0.500
SPOFFS31              0 Hz
SPW31        0.95405000 W

====== GRADIENT CHANNEL =====
GPNAM[1]      SMSQ10.100
GPNAM[2]      SMSQ10.100
GPNAM[3]      SMSQ10.100
GPNAM[4]      SMSQ10.100
GPZ1              80.00 %
GPZ2              20.10 %
GPZ3              11.00 %
GPZ4              -5.00 %
P16             1000.00 usec
P19              600.00 usec

F1 - Acquisition parameters
TD                  256
SFO1           100.6203 MHz
FIDRES       130.208328 Hz
SW              165.639 ppm
FnMODE     Echo-Antiecho

F2 - Processing parameters
SI                 1024
SF          400.1300000 MHz
WDW               QSINE
SSB                   2
LB                    0 Hz
GB                    0
PC                 1.40

F1 - Processing parameters
SI                 1024
MC2        echo-antiecho
SF          100.6127690 MHz
WDW               QSINE
SSB                   2
LB                    0 Hz
GB                    0
```

```
F2 - Acquisition Parameters
Date_          20171012
Time              12.22
INSTRUM           spect
PROBHD   5 mm PABBO BB-
PULPROG  hsqcetgpsisp2.2
TD                 2048
SOLVENT             D2O
NS                    2
DS                   16
SWH            5341.880 Hz
FIDRES         2.608340 Hz
AQ            0.1916928 sec
RG               192.58
DW               93.600 usec
DE                 6.50 usec
TE                298.3 K
CNST2       145.0000000
CNST17       -0.5000000
D0           0.00000300 sec
D1           1.50000000 sec
D4           0.00172414 sec
D11          0.03000000 sec
D16          0.00020000 sec
D24          0.00086207 sec
IN0          0.00003000 sec

======== CHANNEL f1 ========
SFO1        400.1324057 MHz
NUC1                 1H
P1                15.00 usec
P2                30.00 usec
P28             1000.00 usec
PLW1        12.50300026 W

======== CHANNEL f2 ========
SFO2        100.6202713 MHz
NUC2                13C
CPDPRG[2 bi_p5m4sp_4sp.2
P3                 9.80 usec
P14              500.00 usec
P24             2000.00 usec
P63             1500.00 usec
PLW0                  0 W
PLW2        60.95399857 W
PLW12        0.91469002 W
SPNAM[3] Crp60,0.5,20.1
SPOAL3            0.500
SPOFFS3               0 Hz
SPW3         8.94419956 W
SPNAM[7]    Crp60comp.4
SPOAL7            0.500
SPOFFS7               0 Hz
SPW7         8.94419956 W
SPNAM[14 Crp32,1.5,20.2
SPOAL14           0.500
SPOFFS14              0 Hz
SPW14        3.81620002 W
SPNAM[31 Crp32,1.5,20.2
SPOAL31           0.500
SPOFFS31              0 Hz
SPW31        0.95405000 W

====== GRADIENT CHANNEL =====
GPNAM[1]      SMSQ10.100
GPNAM[2]      SMSQ10.100
GPNAM[3]      SMSQ10.100
GPNAM[4]      SMSQ10.100
GPZ1              80.00 %
GPZ2              20.10 %
GPZ3              11.00 %
GPZ4              -5.00 %
P16             1000.00 usec
P19              600.00 usec

F1 - Acquisition parameters
TD                  256
SFO1           100.6203 MHz
FIDRES       130.208328 Hz
SW              165.639 ppm
FnMODE     Echo-Antiecho

F2 - Processing parameters
SI                 1024
SF          400.1299657 MHz
WDW               QSINE
SSB                   2
LB                    0 Hz
GB                    0
PC                 1.40

F1 - Processing parameters
SI                 1024
MC2        echo-antiecho
SF          100.6127690 MHz
WDW               QSINE
SSB                   2
LB                    0 Hz
GB                    0
```

**HMBC**	F2 - Acquisition Parameters	F2 - Acquisition Parameters
	Date_ 20180309	Date_ 20171012
	Time 9.11	Time 11.58
	INSTRUM spect	INSTRUM spect
	PROBHD 5 mm PABBO BB-	PROBHD 5 mm PABBO BB-
	PULPROG hmbcgpl2ndqf	PULPROG hmbcgpl2ndqf
	TD 2048	TD 2048
	SOLVENT D2O	SOLVENT D2O
	NS 4	NS 4
	DS 16	DS 16
	SWH 4807.692 Hz	SWH 4807.692 Hz
	FIDRES 2.347506 Hz	FIDRES 2.347506 Hz
	AQ 0.2129920 sec	AQ 0.2129920 sec
	RG 192.58	RG 192.58
	DW 104.000 usec	DW 104.000 usec
	DE 6.50 usec	DE 6.50 usec
	TE 298.1 K	TE 298.1 K
	CNST6 125.0000000	CNST6 125.0000000
	CNST7 165.0000000	CNST7 165.0000000
	CNST13 7.5000000	CNST13 7.5000000
	D0 0.00000300 sec	D0 0.00000300 sec
	D1 1.50000000 sec	D1 1.50000000 sec
	D6 0.06666667 sec	D6 0.06666667 sec
	D16 0.00020000 sec	D16 0.00020000 sec
	IN0 0.00002240 sec	IN0 0.00002240 sec
	======== CHANNEL f1 ========	======== CHANNEL f1 ========
	SFO1 400.1322007 MHz	SFO1 400.1322007 MHz
	NUC1 1H	NUC1 1H
	P1 15.00 usec	P1 15.00 usec
	P2 30.00 usec	P2 30.00 usec
	PLW1 12.50300026 W	PLW1 12.50300026 W
	======== CHANNEL f2 ========	======== CHANNEL f2 ========
	SFO2 100.6228119 MHz	SFO2 100.6228119 MHz
	NUC2 13C	NUC2 13C
	P3 9.80 usec	P3 9.80 usec
	PLW2 60.95399857 W	PLW2 60.95399857 W
	====== GRADIENT CHANNEL =====	====== GRADIENT CHANNEL =====
	GPNAM[1] SMSQ10.100	GPNAM[1] SMSQ10.100
	GPNAM[2] SMSQ10.100	GPNAM[2] SMSQ10.100
	GPNAM[3] SMSQ10.100	GPNAM[3] SMSQ10.100
	GPNAM[4] SMSQ10.100	GPNAM[4] SMSQ10.100
	GPNAM[5] SMSQ10.100	GPNAM[5] SMSQ10.100
	GPNAM[6] SMSQ10.100	GPNAM[6] SMSQ10.100
	GPZ1 50.00 %	GPZ1 50.00 %
	GPZ2 30.00 %	GPZ2 30.00 %
	GPZ3 40.10 %	GPZ3 40.10 %
	GPZ4 15.00 %	GPZ4 15.00 %
	GPZ5 -10.00 %	GPZ5 -10.00 %
	GPZ6 -5.00 %	GPZ6 -5.00 %
	P16 1000.00 usec	P16 1000.00 usec
	F1 - Acquisition parameters	F1 - Acquisition parameters
	TD 128	TD 128
	SFO1 100.6228 MHz	SFO1 100.6228 MHz
	FIDRES 348.772308 Hz	FIDRES 348.772308 Hz
	SW 221.833 ppm	SW 221.833 ppm
	FnMODE QF	FnMODE QF
	F2 - Processing parameters	F2 - Processing parameters
	SI 2048	SI 2048
	SF 400.1300000 MHz	SF 400.1300000 MHz
	WDW SINE	WDW SINE
	SSB 0	SSB 0
	LB 0 Hz	LB 0 Hz
	GB 0	GB 0
	PC 1.40	PC 1.40
	F1 - Processing parameters	F1 - Processing parameters
	SI 1024	SI 1024
	MC2 QF	MC2 QF
	SF 100.6127690 MHz	SF 100.6127690 MHz
	WDW SINE	WDW SINE
	SSB 0	SSB 0
	LB 0 Hz	LB 0 Hz
	GB 0	GB 0

References

1. Campkin, D.M.; Shimadate, Y.; Bartholomew, B.; Bernhardt, P.V.; Nash, R.J.; Sakoff, J.A.; Kato, A.; Simone, M. Borylated 2,3,4,5-Tetrachlorophthalimide and Their 2,3,4,5-Tetrachlorobenzamide Analogues: Synthesis, Their Glycosidase Inhibition and Anticancer Properties in View to Boron Neutron Capture Therapy. *Molecules* **2022**, *27*, 3447-3475, doi:https://doi.org/10.3390/molecules27113447.
2. Boron Molecular, https://www.boronmolecular.com. A fine chemicals manufacturer.
3. Legge, W.J.; Shimadate, Y.; Sakoff, J.; Houston, T.A.; Kato, A.; Bernhardt, P.V.; Simone, M. Borylated methyl cinnamates: Green synthesis, characterization, crystallographic analysis and biological activities – in glycosidase inhibition and in cancer cells lines. *Beilstein Arch.* **2021**, 20214, doi:https://doi.org/10.3762/bxiv.2021.4.v1.
4. Simone, M. Diastereoselective Synthesis of the Borylated D-Galactose Monosaccharide 3-Boronic-3-Deoxy-D-Galactose and Biological Evaluation in Glycosidase Inhibition and in Cancer for Boron Neutron Capture Therapy (BNCT). *Molecules* **2023**, *28*, 4321-4337, doi:https://doi.org/10.3390/molecules28114321.
5. Simone, M. Borylated Monosaccharide 3-Boronic-3-deoxy-D-galactose: Detailed NMR Spectroscopic Characterisation, and Method for Spectroscopic Analysis of Anomeric and Boron Equilibria. *Internat. J. Mol. Sci.* **2024**, *25*, 12396, doi:https://doi.org/10.3390/ijms252212396.
